# Supplementary material for: A Critical View of the Application of the APEX Software (Aqueous Photochemistry of Environmentally-Occurring Xenobiotics) to Predict Photoreaction Kinetics in Surface Freshwaters
Source: Molecules. 2019 Dec 18;25(1):9. doi: 10.3390/molecules25010009 (PMC7017383; doi:10.3390/molecules25010009)
Supplement: Supplementary file 1 [file molecules-25-00009-s001.zip › 3.2.4_gcc-4.4.0.zip!/3.2.4_gcc-4.4.0/3.2.4_gcc-4.4.0/doc/octave/pdf/octave.pdf]

GNU Octave

A high-level interactive language for numerical computations
Edition 3 for Octave version 3.2.4
July 2007

John W. Eaton
David Bateman
Søren Hauberg

Copyright © 1996, 1997, 1999, 2000, 2001, 2002, 2005, 2006, 2007 John W. Eaton.

This is the third edition of the Octave documentation, and is consistent with version 3.2.4 of Octave.

Permission is granted to make and distribute verbatim copies of this manual provided the copyright notice and this permission notice are preserved on all copies.

Permission is granted to copy and distribute modified versions of this manual under the conditions for verbatim copying, provided that the entire resulting derived work is distributed under the terms of a permission notice identical to this one.

Permission is granted to copy and distribute translations of this manual into another language, under the same conditions as for modified versions.

Portions of this document have been adapted from the `gawk`, `readline`, `gcc`, and C library manuals, published by the Free Software Foundation, Inc., 51 Franklin Street, Fifth Floor, Boston, MA 02110-1301–1307, USA.

Table of Contents

Preface **1**

Acknowledgements 1
How You Can Contribute to Octave 4
Distribution 4

1 A Brief Introduction to Octave **5**

1.1 Running Octave 5
1.2 Simple Examples 5
1.2.1 Elementary Calculations 5
1.2.2 Creating a Matrix 6
1.2.3 Matrix Arithmetic 6
1.2.4 Solving Systems of Linear Equations 6
1.2.5 Integrating Differential Equations 7
1.2.6 Producing Graphical Output 8
1.2.7 Editing What You Have Typed 8
1.2.8 Help and Documentation 8
1.3 Conventions 9
1.3.1 Fonts 9
1.3.2 Evaluation Notation 9
1.3.3 Printing Notation 9
1.3.4 Error Messages 10
1.3.5 Format of Descriptions 10
1.3.5.1 A Sample Function Description 10
1.3.5.2 A Sample Command Description 11
1.3.5.3 A Sample Variable Description 11

2 Getting Started **13**

2.1 Invoking Octave from the Command Line 13
2.1.1 Command Line Options 13
2.1.2 Startup Files 16
2.2 Quitting Octave 17
2.3 Commands for Getting Help 17
2.4 Command Line Editing 19
2.4.1 Cursor Motion 20
2.4.2 Killing and Yanking 21
2.4.3 Commands For Changing Text 21
2.4.4 Letting Readline Type For You 22
2.4.5 Commands For Manipulating The History 22
2.4.6 Customizing `readline` 24
2.4.7 Customizing the Prompt 25
2.4.8 Diary and Echo Commands 26
2.5 How Octave Reports Errors 27

2.6 Executable Octave Programs 28
2.7 Comments in Octave Programs 29
2.7.1 Single Line Comments 29
2.7.2 Block Comments 30
2.7.3 Comments and the Help System 30

3 Data Types 33
3.1 Built-in Data Types 33
3.1.1 Numeric Objects 34
3.1.2 Missing Data 34
3.1.3 String Objects 35
3.1.4 Data Structure Objects 35
3.1.5 Cell Array Objects 35
3.2 User-defined Data Types 35
3.3 Object Sizes 35

4 Numeric Data Types 39
4.1 Matrices 40
4.1.1 Empty Matrices 42
4.2 Ranges 43
4.3 Single Precision Data Types 44
4.4 Integer Data Types 45
4.4.1 Integer Arithmetic 47
4.5 Bit Manipulations 48
4.6 Logical Values 50
4.7 Promotion and Demotion of Data Types 51
4.8 Predicates for Numeric Objects 52

5 Strings 55
5.1 Escape Sequences in string constants 55
5.2 Character Arrays 56
5.3 Creating Strings 57
5.3.1 Concatenating Strings 57
5.3.2 Conversion of Numerical Data to Strings 60
5.4 Comparing Strings 62
5.5 Manipulating Strings 64
5.6 String Conversions 70
5.7 Character Class Functions 75

6 Data Containers 77
6.1 Data Structures 77
6.1.1 Basic Usage and Examples 77
6.1.2 Structure Arrays 80
6.1.3 Creating Structures 81
6.1.4 Manipulating Structures 83
6.1.5 Processing Data in Structures 84
6.2 Cell Arrays 85
6.2.1 Basic Usage of Cell Arrays 85
6.2.2 Creating Cell Array 86
6.2.3 Indexing Cell Arrays 88
6.2.4 Cell Arrays of Strings 90
6.2.5 Processing Data in Cell Arrays 91
6.3 Comma Separated Lists 93
6.3.1 Comma Separated Lists Generated from Cell Arrays 93
6.3.2 Comma Separated Lists Generated from Structure Arrays 94

7 Variables 97
7.1 Global Variables 99
7.2 Persistent Variables 100
7.3 Status of Variables 102

8 Expressions 107
8.1 Index Expressions 107
8.2 Calling Functions 109
8.2.1 Call by Value 110
8.2.2 Recursion 111
8.3 Arithmetic Operators 111
8.4 Comparison Operators 113
8.5 Boolean Expressions 113
8.5.1 Element-by-element Boolean Operators 113
8.5.2 Short-circuit Boolean Operators 114
8.6 Assignment Expressions 115
8.7 Increment Operators 117
8.8 Operator Precedence 118

9 Evaluation 121
9.1 Calling a Function by its Name 121
9.2 Evaluation in a Different Context 122

10 Statements 125

10.1 The if Statement 125
10.2 The switch Statement 127
10.2.1 Notes for the C programmer 128
10.3 The while Statement 129
10.4 The do-until Statement 130
10.5 The for Statement 130
10.5.1 Looping Over Structure Elements 131
10.6 The break Statement 132
10.7 The continue Statement 133
10.8 The unwind_protect Statement 134
10.9 The try Statement 134
10.10 Continuation Lines 135

11 Functions and Scripts 137

11.1 Defining Functions 137
11.2 Multiple Return Values 139
11.3 Variable-length Argument Lists 141
11.4 Variable-length Return Lists 143
11.5 Returning From a Function 143
11.6 Default Arguments 144
11.7 Function Files 145
11.7.1 Manipulating the load path 147
11.7.2 Subfunctions 149
11.7.3 Private Functions 150
11.7.4 Overloading and Autoloading 150
11.7.5 Function Locking 152
11.7.6 Function Precedence 153
11.8 Script Files 153
11.9 Function Handles, Inline Functions, and Anonymous Functions 155
11.9.1 Function Handles 155
11.9.2 Anonymous Functions 156
11.9.3 Inline Functions 156
11.10 Commands 157
11.11 Organization of Functions Distributed with Octave 158

12 Errors and Warnings 161

12.1 Handling Errors 161
12.1.1 Raising Errors 161
12.1.2 Catching Errors 164
12.2 Handling Warnings 166
12.2.1 Issuing Warnings 166
12.2.2 Enabling and Disabling Warnings 167

13 Debugging . . . 171

13.1 Entering Debug Mode . . . 171
13.2 Leavinging Debug Mode . . . 171
13.3 Breakpoints . . . 172
13.4 Debug Mode . . . 173
13.5 Call Stack . . . 174

14 Input and Output . . . 175

14.1 Basic Input and Output . . . 175
14.1.1 Terminal Output . . . 175
14.1.1.1 Paging Screen Output . . . 177
14.1.2 Terminal Input . . . 179
14.1.3 Simple File I/O . . . 180
14.1.3.1 Saving Data on Unexpected Exits . . . 185
14.1.4 Rational Approximations . . . 187
14.2 C-Style I/O Functions . . . 187
14.2.1 Opening and Closing Files . . . 188
14.2.2 Simple Output . . . 189
14.2.3 Line-Oriented Input . . . 190
14.2.4 Formatted Output . . . 190
14.2.5 Output Conversion for Matrices . . . 192
14.2.6 Output Conversion Syntax . . . 192
14.2.7 Table of Output Conversions . . . 193
14.2.8 Integer Conversions . . . 194
14.2.9 Floating-Point Conversions . . . 194
14.2.10 Other Output Conversions . . . 195
14.2.11 Formatted Input . . . 195
14.2.12 Input Conversion Syntax . . . 197
14.2.13 Table of Input Conversions . . . 197
14.2.14 Numeric Input Conversions . . . 198
14.2.15 String Input Conversions . . . 198
14.2.16 Binary I/O . . . 199
14.2.17 Temporary Files . . . 202
14.2.18 End of File and Errors . . . 202
14.2.19 File Positioning . . . 203

15 Plotting . . . 205

15.1 Plotting Basics . . . 205
15.1.1 Two-Dimensional Plots . . . 205
15.1.1.1 Two-dimensional Function Plotting . . . 223
15.1.2 Three-Dimensional Plotting . . . 226
15.1.2.1 Three-dimensional Function Plotting . . . 232
15.1.2.2 Three-dimensional Geometric Shapes . . . 235
15.1.3 Plot Annotations . . . 235
15.1.4 Multiple Plots on One Page . . . 238
15.1.5 Multiple Plot Windows . . . 239
15.1.6 Printing Plots . . . 239

15.1.7 Interacting with plots 242
15.1.8 Test Plotting Functions 242
15.2 Advanced Plotting 243
15.2.1 Graphics Objects 243
15.2.2 Graphics Object Properties 249
15.2.2.1 Root Figure Properties 249
15.2.2.2 Figure Properties 249
15.2.2.3 Axes Properties 249
15.2.2.4 Line Properties 251
15.2.2.5 Text Properties 252
15.2.2.6 Image Properties 255
15.2.2.7 Patch Properties 255
15.2.2.8 Surface Properties 256
15.2.2.9 Searching Properties 256
15.2.3 Managing Default Properties 257
15.2.4 Colors 258
15.2.5 Line Styles 258
15.2.6 Marker Styles 258
15.2.7 Callbacks 259
15.2.8 Object Groups 260
15.2.8.1 Data sources in object groups 263
15.2.8.2 Area series 264
15.2.8.3 Bar series 264
15.2.8.4 Contour groups 265
15.2.8.5 Error bar series 267
15.2.8.6 Line series 267
15.2.8.7 Quiver group 268
15.2.8.8 Scatter group 269
15.2.8.9 Stair group 269
15.2.8.10 Stem Series 270
15.2.8.11 Surface group 271
15.2.9 Graphics backends 271
15.2.9.1 Interaction with `gnuplot` 272

16 Matrix Manipulation 273

16.1 Finding Elements and Checking Conditions 273
16.2 Rearranging Matrices 275
16.3 Applying a Function to an Array 282
16.4 Special Utility Matrices 284
16.5 Famous Matrices 289

17 Arithmetic 293

17.1 Exponents and Logarithms 293
17.2 Complex Arithmetic 294
17.3 Trigonometry 295
17.4 Sums and Products 299
17.5 Utility Functions 301
17.6 Special Functions 307
17.7 Coordinate Transformations 311
17.8 Mathematical Constants 311

18 Linear Algebra 315

18.1 Techniques used for Linear Algebra 315
18.2 Basic Matrix Functions 315
18.3 Matrix Factorizations 320
18.4 Functions of a Matrix 328
18.5 Specialized Solvers 329

19 Nonlinear Equations 331

20 Diagonal and Permutation Matrices 335

20.1 Creating and Manipulating Diagonal and Permutation Matrices 335
20.1.1 Creating Diagonal Matrices 335
20.1.2 Creating Permutation Matrices 336
20.1.3 Explicit and Implicit Conversions 337
20.2 Linear Algebra with Diagonal and Permutation Matrices 337
20.2.1 Expressions Involving Diagonal Matrices 337
20.2.2 Expressions Involving Permutation Matrices 339
20.3 Functions That Are Aware of These Matrices 339
20.3.1 Diagonal Matrix Functions 339
20.3.2 Permutation Matrix Functions 339
20.4 Some Examples of Usage 340
20.5 The Differences in Treatment of Zero Elements 340

21 Sparse Matrices 343

21.1 The Creation and Manipulation of Sparse Matrices 343
21.1.1 Storage of Sparse Matrices 343
21.1.2 Creating Sparse Matrices 344
21.1.3 Finding out Information about Sparse Matrices 349
21.1.4 Basic Operators and Functions on Sparse Matrices 353
21.1.4.1 Sparse Functions 353
21.1.4.2 The Return Types of Operators and Functions 354
21.1.4.3 Mathematical Considerations 355
21.2 Linear Algebra on Sparse Matrices 363
21.3 Iterative Techniques applied to sparse matrices 371
21.4 Real Life Example of the use of Sparse Matrices 376

22 Numerical Integration 381
22.1 Functions of One Variable 381
22.2 Orthogonal Collocation 385
22.3 Functions of Multiple Variables 385

23 Differential Equations 389
23.1 Ordinary Differential Equations 389
23.2 Differential-Algebraic Equations 391

24 Optimization 401
24.1 Linear Programming 401
24.2 Quadratic Programming 407
24.3 Nonlinear Programming 408
24.4 Linear Least Squares 410

25 Statistics 413
25.1 Descriptive Statistics 413
25.2 Basic Statistical Functions 417
25.3 Statistical Plots 420
25.4 Tests 421
25.5 Models 429
25.6 Distributions 429
25.7 Random Number Generation 436

26 Sets 441
26.1 Set Operations 441

27 Polynomial Manipulations 445
27.1 Evaluating Polynomials 445
27.2 Finding Roots 446
27.3 Products of Polynomials 447
27.4 Derivatives and Integrals 449
27.5 Polynomial Interpolation 450
27.6 Miscellaneous Functions 452

28 Interpolation 455
28.1 One-dimensional Interpolation 455
28.2 Multi-dimensional Interpolation 459

29 Geometry 463
29.1 Delaunay Triangulation 463
29.1.1 Plotting the Triangulation 465
29.1.2 Identifying points in Triangulation 466
29.2 Voronoi Diagrams 468
29.3 Convex Hull 471
29.4 Interpolation on Scattered Data 472

30 Signal Processing **475**

31 Image Processing **485**

31.1 Loading and Saving Images 485
31.2 Displaying Images 487
31.3 Representing Images 489
31.4 Plotting on top of Images 493
31.5 Color Conversion 493

32 Audio Processing **495**

33 Object Oriented Programming **497**

33.1 Creating a Class 497
33.2 Manipulating Classes 499
33.3 Indexing Objects 502
33.4 Overloading Objects 506
33.4.1 Function Overloading 506
33.4.2 Operator Overloading 508
33.4.3 Precedence of Objects 508
33.5 Inheritance and Aggregation 510

34 System Utilities **515**

34.1 Timing Utilities 515
34.2 Filesystem Utilities 524
34.3 File Archiving Utilities 530
34.4 Networking Utilities 532
34.5 Controlling Subprocesses 533
34.6 Process, Group, and User IDs 539
34.7 Environment Variables 540
34.8 Current Working Directory 540
34.9 Password Database Functions 541
34.10 Group Database Functions 542
34.11 System Information 542
34.12 Hashing Functions 545

35 Packages **547**

35.1 Installing and Removing Packages 547
35.2 Using Packages 550
35.3 Administrating Packages 550
35.4 Creating Packages 551
35.4.1 The DESCRIPTION File 552
35.4.2 The INDEX file 554
35.4.3 PKG_ADD and PKG_DEL directives 555

Appendix A Dynamically Linked Functions 557

A.1 Oct-Files 557
A.1.1 Getting Started with Oct-Files 557
A.1.2 Matrices and Arrays in Oct-Files 560
A.1.3 Character Strings in Oct-Files 563
A.1.4 Cell Arrays in Oct-Files 564
A.1.5 Structures in Oct-Files 565
A.1.6 Sparse Matrices in Oct-Files 566
A.1.6.1 The Differences between the Array and Sparse Classes 567
A.1.6.2 Creating Sparse Matrices in Oct-Files 568
A.1.6.3 Using Sparse Matrices in Oct-Files 571
A.1.7 Accessing Global Variables in Oct-Files 571
A.1.8 Calling Octave Functions from Oct-Files 572
A.1.9 Calling External Code from Oct-Files 574
A.1.10 Allocating Local Memory in Oct-Files 576
A.1.11 Input Parameter Checking in Oct-Files 577
A.1.12 Exception and Error Handling in Oct-Files 578
A.1.13 Documentation and Test of Oct-Files 579
A.2 Mex-Files 580
A.2.1 Getting Started with Mex-Files 580
A.2.2 Working with Matrices and Arrays in Mex-Files 582
A.2.3 Character Strings in Mex-Files 584
A.2.4 Cell Arrays with Mex-Files 585
A.2.5 Structures with Mex-Files 586
A.2.6 Sparse Matrices with Mex-Files 588
A.2.7 Calling Other Functions in Mex-Files 591
A.3 Standalone Programs 592

Appendix B Test and Demo Functions 597

B.1 Test Functions 597
B.2 Demonstration Functions 601

Appendix C Tips and Standards 605

C.1 Writing Clean Octave Programs 605
C.2 Tips for Making Code Run Faster 605
C.3 Tips on Writing Comments 607
C.4 Conventional Headers for Octave Functions 608
C.5 Tips for Documentation Strings 610

Appendix D Contributing Guidelines 615

D.1 How to Contribute 615
D.2 General Guidelines 616
D.3 Octave Sources (m-files) 617
D.4 C++ Sources 617
D.5 Other Sources 619

Appendix E Known Causes of Trouble 621
E.1 Actual Bugs We Haven't Fixed Yet 621
E.2 Reporting Bugs 621
E.3 Have You Found a Bug? 622
E.4 Where to Report Bugs 622
E.5 How to Report Bugs 622
E.6 Sending Patches for Octave 624
E.7 How To Get Help with Octave 625

Appendix F Installing Octave 627
F.1 Installation Problems 630

Appendix G Emacs Octave Support 633
G.1 Installing EOS 633
G.2 Using Octave Mode 633
G.3 Running Octave From Within Emacs 637
G.4 Using the Emacs Info Reader for Octave 638

Appendix H GNU GENERAL PUBLIC LICENSE 641

Concept Index 653

Function Index 659

Operator Index 671

Preface

Octave was originally intended to be companion software for an undergraduate-level textbook on chemical reactor design being written by James B. Rawlings of the University of Wisconsin-Madison and John G. Ekerdt of the University of Texas.

Clearly, Octave is now much more than just another 'courseware' package with limited utility beyond the classroom. Although our initial goals were somewhat vague, we knew that we wanted to create something that would enable students to solve realistic problems, and that they could use for many things other than chemical reactor design problems.

There are those who would say that we should be teaching the students Fortran instead, because that is the computer language of engineering, but every time we have tried that, the students have spent far too much time trying to figure out why their Fortran code crashes and not enough time learning about chemical engineering. With Octave, most students pick up the basics quickly, and are using it confidently in just a few hours.

Although it was originally intended to be used to teach reactor design, it has been used in several other undergraduate and graduate courses in the Chemical Engineering Department at the University of Texas, and the math department at the University of Texas has been using it for teaching differential equations and linear algebra as well. If you find it useful, please let us know. We are always interested to find out how Octave is being used in other places.

Virtually everyone thinks that the name Octave has something to do with music, but it is actually the name of a former professor of mine who wrote a famous textbook on chemical reaction engineering, and who was also well known for his ability to do quick 'back of the envelope' calculations. We hope that this software will make it possible for many people to do more ambitious computations just as easily.

Everyone is encouraged to share this software with others under the terms of the GNU General Public License (see Appendix H [Copying], page 641). You are also encouraged to help make Octave more useful by writing and contributing additional functions for it, and by reporting any problems you may have.

Acknowledgements

Many people have already contributed to Octave's development. The following people have helped code parts of Octave or aided in various other ways (listed alphabetically).

Ben Abbott, Andy Adler, Joel Andersson
Muthiah Annamalai, Shai Ayal, Roger Banks
Ben Barrowes, Alexander Barth, David Bateman
Heinz Bauschke, Karl Berry, David Billinghurst
Don Bindner, Jakub Bogusz, Moritz Borgmann
Richard Bovey, Marcus Brinkmann, Remy Bruno
Marco Caliari, Daniel Calvelo, John C. Campbell
Jean-Francois Cardoso, Joao Cardoso, Larrie Carr
David Castelow, Vincent Cautaerts, Clinton Chee
Albert Chin-A-Young, Carsten Clark, J. D. Cole
Martin Costabel, Michael Creel, Jeff Cunningham
Martin Dalecki, Jorge Barros de Abreu, Carlo de Falco

Thomas D. Dean
Philippe Defert
Bill Denney
David M. Doolin
Pascal A. Dupuis
John W. Eaton
Dirk Eddelbuettel
Paul Eggert
Stephen Eglen
Peter Ekberg
Rolf Fabian
Stephen Fegan
Ramon Garcia Fernandez
Torsten Finke
Jose Daniel Munoz Frias
Castor Fu
Eduardo Gallestey
Walter Gautschi
Klaus Gebhardt
Driss Ghaddab
Nicolo Giorgetti
Michael Goffioul
Glenn Golden
Tomislav Goles
Keith Goodman
Brian Gough
Steffen Groot
Etienne Grossmann
Peter Gustafson
Kai Habel
William P. Y. Hadisoeseno
Jaroslav Hajek
Benjamin Hall
Kim Hansen
Soren Hauberg
Dave Hawthorne
Daniel Heiserer
Martin Helm
Stefan Hepp
Yozo Hida
Ryan Hinton
Roman Hodek
A. Scottedward Hodel
Richard Allan Holcombe
Tom Holroyd
David Hoover
Kurt Hornik
Christopher Hulbert
Cyril Humbert
Teemu Ikonen
Alan W. Irwin
Geoff Jacobsen
Mats Jansson
Cai Jianming
Steven G. Johnson
Heikki Junes
Atsushi Kajita
Jarkko Kaleva
Mohamed Kamoun
Lute Kamstra
Thomas Kasper
Joel Keay
Mumit Khan
Paul Kienzle
Aaron A. King
Arno J. Klaassen
Geoffrey Knauth
Heine Kolltveit
Ken Kouno
Oyvind Kristiansen
Piotr Krzyzanowski
Volker Kuhlmann
Tetsuro Kurita
Miroslaw Kwasniak
Rafael Laboissiere
Kai Labusch
Claude Lacoursiere
Walter Landry
Bill Lash
Dirk Laurie
Maurice LeBrun
Friedrich Leisch
Timo Lindfors
Benjamin Lindner
Ross Lippert
David Livings
Erik de Castro Lopo
Massimo Lorenzin
Emil Lucretiu
Hoxide Ma
James Macnicol
Jens-Uwe Mager
Ricardo Marranita
Orestes Mas
Makoto Matsumoto
Tatsuro Matsuoka
Laurent Mazet
G. D. McBain
Alexander Mamonov
Christoph Mayer
Thorsten Meyer
Petr Mikulik
Stefan Monnier
Antoine Moreau
Kai P. Mueller
Victor Munoz
Carmen Navarrete
Todd Neal
Al Niessner
Rick Niles
Takuji Nishimura
Kai Noda
Eric Norum
Krzesimir Nowak
Michael O'Brien
Peter O'Gorman
Thorsten Ohl
Arno Onken
Luis F. Ortiz
Scott Pakin
Gabriele Pannocchia
Sylvain Pelissier
Per Persson
Primozz Peterlin
Jim Peterson
Danilo Piazzalunga
Nicholas Piper
Robert Platt
Hans Ekkehard Plesser
Tom Poage
Orion Poplawski
Ondrej Popp
Jef Poskanzer
Francesco Potorti
James B. Rawlings
Eric S. Raymond
Balint Reczey
Michael Reifenberger
Jason Riedy
Petter Risholm
Matthew W. Roberts

Andrew Ross
Ryan Rusaw
Juhani Saastamoinen
Aleksej Saushev
Julian Schnidder
Ludwig Schwardt
Dmitri A. Sergatskov
John Smith
Joerg Specht
Richard Stallman
Jonathan Stickel
John Swensen
Duncan Temple Lang
Thomas Treichl
Stefan van der Walt
Gregory Vanuxem
Olaf Weber
Bob Weigel
Fook Fah Yap
Alex Zvoleff
Mark van Rossum
Olli Saarela
Radek Salac
Alois Schloegl
Nicol N. Schraudolph
Thomas L. Scofield
Baylis Shanks
Julius Smith
Quentin H. Spencer
Russell Standish
Thomas Stuart
Ariel Tankus
Kris Thielemans
Frederick Umminger
Peter Van Wieren
Ivana Varekova
Thomas Weber
Andreas Weingessel
Michael Zeising
Kevin Ruland
Toni Saarela
Ben Sapp
Michel D. Schmid
Sebastian Schubert
Daniel J. Sebald
Joseph P. Skudlarek
Shan G. Smith
Christoph Spiel
Doug Stewart
Ivan Sutoris
Georg Thimm
Olaf Till
Utkarsh Upadhyay
James R. Van Zandt
Thomas Walter
Rik Wehbring
Michael Weitzel
Federico Zenith

Special thanks to the following people and organizations for supporting the development of Octave:

- The United States Department of Energy, through grant number DE-FG02-04ER25635.
- Ashok Krishnamurthy, David Hudak, Juan Carlos Chaves, and Stanley C. Ahalt of the Ohio Supercomputer Center.
- The National Science Foundation, through grant numbers CTS-0105360, CTS-9708497, CTS-9311420, CTS-8957123, and CNS-0540147.
- The industrial members of the Texas-Wisconsin Modeling and Control Consortium (TWMCC).
- The Paul A. Elfers Endowed Chair in Chemical Engineering at the University of Wisconsin-Madison.
- Digital Equipment Corporation, for an equipment grant as part of their External Research Program.
- Sun Microsystems, Inc., for an Academic Equipment grant.
- International Business Machines, Inc., for providing equipment as part of a grant to the University of Texas College of Engineering.
- Texaco Chemical Company, for providing funding to continue the development of this software.
- The University of Texas College of Engineering, for providing a Challenge for Excellence Research Supplement, and for providing an Academic Development Funds grant.
- The State of Texas, for providing funding through the Texas Advanced Technology Program under Grant No. 003658-078.
- Noel Bell, Senior Engineer, Texaco Chemical Company, Austin Texas.

- John A. Turner, Group Leader, Continuum Dynamics (CCS-2), Los Alamos National Laboratory, for registering the `octave.org` domain name.
- James B. Rawlings, Professor, University of Wisconsin-Madison, Department of Chemical and Biological Engineering.
- Richard Stallman, for writing GNU.

This project would not have been possible without the GNU software used in and to produce Octave.

How You Can Contribute to Octave

There are a number of ways that you can contribute to help make Octave a better system. Perhaps the most important way to contribute is to write high-quality code for solving new problems, and to make your code freely available for others to use. See Appendix D [Contributing Guidelines], page 615, for detailed information on contributing new code.

If you find Octave useful, consider providing additional funding to continue its development. Even a modest amount of additional funding could make a significant difference in the amount of time that is available for development and support.

If you cannot provide funding or contribute code, you can still help make Octave better and more reliable by reporting any bugs you find and by offering suggestions for ways to improve Octave. See Appendix E [Trouble], page 621, for tips on how to write useful bug reports.

Distribution

Octave is *free* software. This means that everyone is free to use it and free to redistribute it on certain conditions. Octave is not, however, in the public domain. It is copyrighted and there are restrictions on its distribution, but the restrictions are designed to ensure that others will have the same freedom to use and redistribute Octave that you have. The precise conditions can be found in the GNU General Public License that comes with Octave and that also appears in Appendix H [Copying], page 641.

Octave is available on CD-ROM, with various collections of other free software, from the Free Software Foundation. Ordering a copy of Octave from the Free Software Foundation helps to fund the development of more free software. For more information, write to

Free Software Foundation
51 Franklin Street, Fifth Floor
Boston, MA 02110-1301–1307
USA

Octave can also be downloaded from `http://www.octave.org`, where additional information is available.

1 A Brief Introduction to Octave

GNU Octave is a high-level language, primarily intended for numerical computations. It provides a convenient interactive command line interface for solving linear and nonlinear problems numerically, and for performing other numerical experiments. It may also be used as a batch-oriented language for data processing.

GNU Octave is freely redistributable software. You may redistribute it and/or modify it under the terms of the GNU General Public License as published by the Free Software Foundation. The GPL is included in this manual in Appendix H [Copying], page 641.

This manual provides comprehensive documentation on how to install, run, use, and extend GNU Octave. Additional chapters describe how to report bugs and help contribute code.

This document corresponds to Octave version 3.2.4.

1.1 Running Octave

On most systems, Octave is started with the shell command '`octave`'. Octave displays an initial message and then a prompt indicating it is ready to accept input. You can begin typing Octave commands immediately afterward.

If you get into trouble, you can usually interrupt Octave by typing *Control-C* (written *C-c* for short). *C-c* gets its name from the fact that you type it by holding down CTRL and then pressing C. Doing this will normally return you to Octave's prompt.

To exit Octave, type *quit*, or *exit* at the Octave prompt.

On systems that support job control, you can suspend Octave by sending it a `SIGTSTP` signal, usually by typing *C-z*.

1.2 Simple Examples

The following chapters describe all of Octave's features in detail, but before doing that, it might be helpful to give a sampling of some of its capabilities.

If you are new to Octave, I recommend that you try these examples to begin learning Octave by using it. Lines marked with '`octave:13>`' are lines you type, ending each with a carriage return. Octave will respond with an answer, or by displaying a graph.

1.2.1 Elementary Calculations

Octave can easily be used for basic numerical calculations. Octave knows about arithmetic operations (+,-,*,/), exponentiation (^), natural logarithms/exponents (log, exp), and the trigonometric functions (sin, cos, . . .). Moreover, Octave calculations work on real or imaginary numbers (i,j). In addition, some mathematical constants such as the base of the natural logarithm (e) and the ratio of a circle's circumference to its diameter (pi) are pre-defined.

For example, to verify Euler's Identity,

$$e^{\imath\pi} = -1$$

type the following which will evaluate to `-1` within the tolerance of the calculation.

```
octave:1> exp(i*pi)
```

1.2.2 Creating a Matrix

Vectors and matrices are the basic building blocks for numerical analysis. To create a new matrix and store it in a variable so that you can refer to it later, type the command

```
octave:1> A = [ 1, 1, 2; 3, 5, 8; 13, 21, 34 ]
```

Octave will respond by printing the matrix in neatly aligned columns. Octave uses a comma or space to separate entries in a row, and a semicolon or carriage return to separate one row from the next. Ending a command with a semicolon tells Octave not to print the result of the command. For example,

```
octave:2> B = rand (3, 2);
```

will create a 3 row, 2 column matrix with each element set to a random value between zero and one.

To display the value of a variable, simply type the name of the variable at the prompt. For example, to display the value stored in the matrix `B`, type the command

```
octave:3> B
```

1.2.3 Matrix Arithmetic

Octave has a convenient operator notation for performing matrix arithmetic. For example, to multiply the matrix `A` by a scalar value, type the command

```
octave:4> 2 * A
```

To multiply the two matrices `A` and `B`, type the command

```
octave:5> A * B
```

and to form the matrix product $A^T A$, type the command

```
octave:6> A' * A
```

1.2.4 Solving Systems of Linear Equations

Systems of linear equations are ubiquitous in numerical analysis. To solve the set of linear equations `Ax = b`, use the left division operator, '\':

```
x = A \ b
```

This is conceptually equivalent to $A^{-1}b$, but avoids computing the inverse of a matrix directly.

If the coefficient matrix is singular, Octave will print a warning message and compute a minimum norm solution.

A simple example comes from chemistry and the need to obtain balanced chemical equations. Consider the burning of hydrogen and oxygen to produce water.

$$H_2 + O_2 \rightarrow H_2O$$

The equation above is not accurate. The Law of Conservation of Mass requires that the number of molecules of each type balance on the left- and right-hand sides of the equation. Writing the variable overall reaction with individual equations for hydrogen and oxygen one finds:

$$x_1 H_2 + x_2 O_2 \rightarrow H_2O$$
$$H: \quad 2x_1 + 0x_2 \rightarrow 2$$
$$O: \quad 0x_1 + 2x_2 \rightarrow 1$$

The solution in Octave is found in just three steps.

```
octave:1> A = [ 2, 0; 0, 2 ];
octave:2> b = [ 2; 1 ];
octave:3> x = A \ b
```

1.2.5 Integrating Differential Equations

Octave has built-in functions for solving nonlinear differential equations of the form

$$\frac{dx}{dt} = f(x,t), \qquad x(t = t_0) = x_0$$

For Octave to integrate equations of this form, you must first provide a definition of the function $f(x,t)$. This is straightforward, and may be accomplished by entering the function body directly on the command line. For example, the following commands define the right-hand side function for an interesting pair of nonlinear differential equations. Note that while you are entering a function, Octave responds with a different prompt, to indicate that it is waiting for you to complete your input.

```
octave:1> function xdot = f (x, t)
>
>   r = 0.25;
>   k = 1.4;
>   a = 1.5;
>   b = 0.16;
>   c = 0.9;
>   d = 0.8;
>
>   xdot(1) = r*x(1)*(1 - x(1)/k) - a*x(1)*x(2)/(1 + b*x(1));
>   xdot(2) = c*a*x(1)*x(2)/(1 + b*x(1)) - d*x(2);
>
> endfunction
```

Given the initial condition

```
octave:2> x0 = [1; 2];
```

and the set of output times as a column vector (note that the first output time corresponds to the initial condition given above)

```
octave:3> t = linspace (0, 50, 200)';
```

it is easy to integrate the set of differential equations:

```
octave:4> x = lsode ("f", x0, t);
```

The function `lsode` uses the Livermore Solver for Ordinary Differential Equations, described in A. C. Hindmarsh, *ODEPACK, a Systematized Collection of ODE Solvers*, in: Scientific Computing, R. S. Stepleman et al. (Eds.), North-Holland, Amsterdam, 1983, pages 55–64.

1.2.6 Producing Graphical Output

To display the solution of the previous example graphically, use the command

```
octave:1> plot (t, x)
```

If you are using a graphical user interface, Octave will automatically create a separate window to display the plot.

To save a plot once it has been displayed on the screen, use the print command. For example,

```
print -deps foo.eps
```

will create a file called '`foo.eps`' that contains a rendering of the current plot in Encapsulated PostScript format. The command

```
help print
```

explains more options for the `print` command and provides a list of additional output file formats.

1.2.7 Editing What You Have Typed

At the Octave prompt, you can recall, edit, and reissue previous commands using Emacs- or vi-style editing commands. The default keybindings use Emacs-style commands. For example, to recall the previous command, press *Control-p* (written *C-p* for short). Doing this will normally bring back the previous line of input. *C-n* will bring up the next line of input, *C-b* will move the cursor backward on the line, *C-f* will move the cursor forward on the line, etc.

A complete description of the command line editing capability is given in this manual in Section 2.4 [Command Line Editing], page 19.

1.2.8 Help and Documentation

Octave has an extensive help facility. The same documentation that is available in printed form is also available from the Octave prompt, because both forms of the documentation are created from the same input file.

In order to get good help you first need to know the name of the command that you want to use. This name of the function may not always be obvious, but a good place to start is to just type `help`. This will show you all the operators, reserved words, functions, built-in variables, and function files. An alternative is to search the documentation using the `lookfor` function. This function is described in Section 2.3 [Getting Help], page 17.

Once you know the name of the function you wish to use, you can get more help on the function by simply including the name as an argument to help. For example,

```
help plot
```

will display the help text for the `plot` function.

Octave sends output that is too long to fit on one screen through a pager like `less` or `more`. Type a RET to advance one line, a SPC to advance one page, and Q to exit the pager.

The part of Octave's help facility that allows you to read the complete text of the printed manual from within Octave normally uses a separate program called Info. When you invoke Info you will be put into a menu driven program that contains the entire Octave manual. Help for using Info is provided in this manual in Section 2.3 [Getting Help], page 17.

1.3 Conventions

This section explains the notational conventions that are used in this manual. You may want to skip this section and refer back to it later.

1.3.1 Fonts

Examples of Octave code appear in this font or form: `svd (a)`. Names that represent variables or function arguments appear in this font or form: *first-number*. Commands that you type at the shell prompt appear in this font or form: '`octave --no-init-file`'. Commands that you type at the Octave prompt sometimes appear in this font or form: *`foo --bar --baz`*. Specific keys on your keyboard appear in this font or form: ANY.

1.3.2 Evaluation Notation

In the examples in this manual, results from expressions that you evaluate are indicated with '⇒'. For example,

```
sqrt (2)
     ⇒ 1.4142
```

You can read this as "`sqrt (2)` evaluates to 1.4142".

In some cases, matrix values that are returned by expressions are displayed like this

```
[1, 2; 3, 4] == [1, 3; 2, 4]
     ⇒ [ 1, 0; 0, 1 ]
```

and in other cases, they are displayed like this

```
eye (3)
     ⇒  1  0  0
         0  1  0
         0  0  1
```

in order to clearly show the structure of the result.

Sometimes to help describe one expression, another expression is shown that produces identical results. The exact equivalence of expressions is indicated with '≡'. For example,

```
rot90 ([1, 2; 3, 4], -1)
≡
rot90 ([1, 2; 3, 4], 3)
≡
rot90 ([1, 2; 3, 4], 7)
```

1.3.3 Printing Notation

Many of the examples in this manual print text when they are evaluated. In this manual the printed text resulting from an example is indicated by '⊣'. The value that is returned by evaluating the expression is displayed with '⇒' (`1` in the next example) and follows on a separate line.

```
printf ("foo %s\n", "bar")
     ⊣ foo bar
     ⇒ 1
```

1.3.4 Error Messages

Some examples signal errors. This normally displays an error message on your terminal. Error messages are shown on a line beginning with `error:`.

```
fieldnames ([1, 2; 3, 4])
error: fieldnames: wrong type argument ‘matrix’
```

1.3.5 Format of Descriptions

Functions, commands, and variables are described in this manual in a uniform format. The first line of a description contains the name of the item followed by its arguments, if any. The category—function, variable, or whatever—is printed next to the right margin. The description follows on succeeding lines, sometimes with examples.

1.3.5.1 A Sample Function Description

In a function description, the name of the function being described appears first. It is followed on the same line by a list of parameters. The names used for the parameters are also used in the body of the description.

Here is a description of an imaginary function `foo`:

`foo` (*x*, *y*, . . .) [Function]

The function `foo` subtracts *x* from *y*, then adds the remaining arguments to the result. If *y* is not supplied, then the number 19 is used by default.

```
foo (1, [3, 5], 3, 9)
     ⇒ [ 14, 16 ]
foo (5)
     ⇒ 14
```

More generally,

```
foo (w, x, y, ...)
≡
x - w + y + ...
```

Any parameter whose name contains the name of a type (e.g., *integer* or *matrix*) is expected to be of that type. Parameters named *object* may be of any type. Parameters with other sorts of names (e.g., *new_file*) are discussed specifically in the description of the function. In some sections, features common to parameters of several functions are described at the beginning.

Functions in Octave may be defined in several different ways. The category name for functions may include another name that indicates the way that the function is defined. These additional tags include

Function File
: The function described is defined using Octave commands stored in a text file. See Section 11.7 [Function Files], page 145.

Built-in Function
: The function described is written in a language like C++, C, or Fortran, and is part of the compiled Octave binary.

Loadable Function

The function described is written in a language like C++, C, or Fortran. On systems that support dynamic linking of user-supplied functions, it may be automatically linked while Octave is running, but only if it is needed. See Appendix A [Dynamically Linked Functions], page 557.

Mapping Function

The function described works element-by-element for matrix and vector arguments.

1.3.5.2 A Sample Command Description

Command descriptions have a format similar to function descriptions, except that the word 'Function' is replaced by 'Command'. Commands are functions that may be called without surrounding their arguments in parentheses. For example, here is the description for Octave's `cd` command:

`cd` *dir* [Command]
`chdir` *dir* [Command]

Change the current working directory to *dir*. For example, *`cd ~/octave`* changes the current working directory to '`~/octave`'. If the directory does not exist, an error message is printed and the working directory is not changed.

1.3.5.3 A Sample Variable Description

A *variable* is a name that can hold a value. Although any variable can be set by the user, *built-in variables* typically exist specifically so that users can change them to alter the way Octave behaves (built-in variables are also sometimes called *user options*). Ordinary variables and built-in variables are described using a format like that for functions except that there are no arguments.

Here is a description of the imaginary variable `do_what_i_mean_not_what_i_say`.

`do_what_i_mean_not_what_i_say` [Built-in Variable]

If the value of this variable is nonzero, Octave will do what you actually wanted, even if you have typed a completely different and meaningless list of commands.

Other variable descriptions have the same format, but 'Built-in Variable' is replaced by 'Variable', for ordinary variables, or 'Constant' for symbolic constants whose values cannot be changed.

2 Getting Started

This chapter explains some of Octave's basic features, including how to start an Octave session, get help at the command prompt, edit the command line, and write Octave programs that can be executed as commands from your shell.

2.1 Invoking Octave from the Command Line

Normally, Octave is used interactively by running the program '`octave`' without any arguments. Once started, Octave reads commands from the terminal until you tell it to exit.

You can also specify the name of a file on the command line, and Octave will read and execute the commands from the named file and then exit when it is finished.

You can further control how Octave starts by using the command-line options described in the next section, and Octave itself can remind you of the options available. Type '`octave --help`' to display all available options and briefly describe their use ('`octave -h`' is a shorter equivalent).

2.1.1 Command Line Options

Here is a complete list of the command line options that Octave accepts.

`--debug`
`-d`
: Enter parser debugging mode. Using this option will cause Octave's parser to print a lot of information about the commands it reads, and is probably only useful if you are actually trying to debug the parser.

`--doc-cache-file` *`filename`*
: Specify the name of the doc cache file to use. The value of *filename* specified on the command line will override any value of `OCTAVE_DOC_CACHE_FILE` found in the environment, but not any commands in the system or user startup files that use the `doc_cache_file` function.

`--echo-commands`
`-x`
: Echo commands as they are executed.

`--eval` *`code`*
: Evaluate *code* and exit when finished unless `--persist` is also specified.

`--exec-path` *`path`*
: Specify the path to search for programs to run. The value of *path* specified on the command line will override any value of `OCTAVE_EXEC_PATH` found in the environment, but not any commands in the system or user startup files that set the built-in variable `EXEC_PATH`.

`--help`
`-h`
`-?`
: Print short help message and exit.

`--image-path` *`path`*
: Add path to the head of the search path for images. The value of *path* specified on the command line will override any value of `OCTAVE_IMAGE_PATH` found in

the environment, but not any commands in the system or user startup files that set the built-in variable `IMAGE_PATH`.

`--info-file` *filename*
: Specify the name of the info file to use. The value of *filename* specified on the command line will override any value of `OCTAVE_INFO_FILE` found in the environment, but not any commands in the system or user startup files that use the `info_file` function.

`--info-program` *program*
: Specify the name of the info program to use. The value of *program* specified on the command line will override any value of `OCTAVE_INFO_PROGRAM` found in the environment, but not any commands in the system or user startup files that use the `info_program` function.

`--interactive`
`-i`
: Force interactive behavior. This can be useful for running Octave via a remote shell command or inside an Emacs shell buffer. For another way to run Octave within Emacs, see Appendix G [Emacs Octave Support], page 633.

`--line-editing`
: Force readline use for command-line editing.

`--no-history`
`-H`
: Disable recording of command-line history.

`--no-init-file`
: Don't read the initialization files '`~/.octaverc`' and '`.octaverc`'.

`--no-init-path`
: Don't initialize the search path for function files to include default locations.

`--no-line-editing`
: Disable command-line editing.

`--no-site-file`
: Don't read the site-wide '`octaverc`' initialization files.

`--norc`
`-f`
: Don't read any of the system or user initialization files at startup. This is equivalent to using both of the options `--no-init-file` and `--no-site-file`.

`--path` *path*
`-p` *path*
: Add path to the head of the search path for function files. The value of *path* specified on the command line will override any value of `OCTAVE_PATH` found in the environment, but not any commands in the system or user startup files that set the internal load path through one of the path functions.

`--persist`
: Go to interactive mode after `--eval` or reading from a file named on the command line.

`--silent`
`--quiet`
`-q`
: Don't print the usual greeting and version message at startup.

`--traditional`
`--braindead`
For compatibility with MATLAB, set initial values for user preferences to the following values

```
PS1                         = ">> "
PS2                         = ""
beep_on_error               = true
confirm_recursive_rmdir     = false
crash_dumps_octave_core     = false
default_save_options        = "-mat-binary"
fixed_point_format          = true
history_timestamp_format_string
                            = "%%-- %D %I:%M %p --%%"
page_screen_output          = false
print_empty_dimensions      = false
```

and disable the following warnings

```
Octave:fopen-file-in-path
Octave:function-name-clash
Octave:load-file-in-path
```

`--verbose`
`-V` Turn on verbose output.

`--version`
`-v` Print the program version number and exit.

`file` Execute commands from *file*. Exit when done unless `--persist` is also specified.

Octave also includes several functions which return information about the command line, including the number of arguments and all of the options.

`argv` () [Built-in Function]

Return the command line arguments passed to Octave. For example, if you invoked Octave using the command

```
octave --no-line-editing --silent
```

`argv` would return a cell array of strings with the elements `--no-line-editing` and `--silent`.

If you write an executable Octave script, `argv` will return the list of arguments passed to the script. See Section 2.6 [Executable Octave Programs], page 28, for an example of how to create an executable Octave script.

`program_name ()` [Built-in Function]

Return the last component of the value returned by `program_invocation_name`.

See also: [program_invocation_name], page 15.

`program_invocation_name ()` [Built-in Function]

Return the name that was typed at the shell prompt to run Octave.

If executing a script from the command line (e.g., `octave foo.m`) or using an executable Octave script, the program name is set to the name of the script. See

Section 2.6 [Executable Octave Programs], page 28, for an example of how to create an executable Octave script.

See also: [program_name], page 15.

Here is an example of using these functions to reproduce the command line which invoked Octave.

```
printf ("%s", program_name ());
arg_list = argv ();
for i = 1:nargin
  printf (" %s", arg_list{i});
endfor
printf ("\n");
```

See Section 6.2.3 [Indexing Cell Arrays], page 88, for an explanation of how to retrieve objects from cell arrays, and Section 11.1 [Defining Functions], page 137, for information about the variable `nargin`.

2.1.2 Startup Files

When Octave starts, it looks for commands to execute from the files in the following list. These files may contain any valid Octave commands, including function definitions.

octave-home`/share/octave/site/m/startup/octaverc`
: where *octave-home* is the directory in which Octave is installed (the default is ‘’). This file is provided so that changes to the default Octave environment can be made globally for all users at your site for all versions of Octave you have installed. Care should be taken when making changes to this file since all users of Octave at your site will be affected. The default file may be overridden by the environment variable `OCTAVE_SITE_INITFILE`.

octave-home`/share/octave/`*version*`/m/startup/octaverc`
: where *octave-home* is the directory in which Octave is installed (the default is ‘’), and *version* is the version number of Octave. This file is provided so that changes to the default Octave environment can be made globally for all users of a particular version of Octave. Care should be taken when making changes to this file since all users of Octave at your site will be affected. The default file may be overridden by the environment variable `OCTAVE_VERSION_INITFILE`.

`~/.octaverc`
: This file is used to make personal changes to the default Octave environment.

`.octaverc`
: This file can be used to make changes to the default Octave environment for a particular project. Octave searches for this file in the current directory after it reads ‘`~/.octaverc`’. Any use of the `cd` command in the ‘`~/.octaverc`’ file will affect the directory where Octave searches for ‘`.octaverc`’.

  If you start Octave in your home directory, commands from the file ‘`~/.octaverc`’ will only be executed once.

A message will be displayed as each of the startup files is read if you invoke Octave with the `--verbose` option but without the `--silent` option.

2.2 Quitting Octave

`exit (status)` [Built-in Function]
`quit (status)` [Built-in Function]

Exit the current Octave session. If the optional integer value *status* is supplied, pass that value to the operating system as the Octave's exit status. The default value is zero.

`atexit (fcn)` [Built-in Function]
`atexit (fcn, flag)` [Built-in Function]

Register a function to be called when Octave exits. For example,

```
function last_words ()
  disp ("Bye bye");
endfunction
atexit ("last_words");
```

will print the message "Bye bye" when Octave exits.

The additional argument *flag* will register or unregister *fcn* from the list of functions to be called when Octave exits. If *flag* is true, the function is registered, and if *flag* is false, it is unregistered. For example, after registering the function `last_words` above,

```
atexit ("last_words", false);
```

will remove the function from the list and Octave will not call `last_words` when it exits.

Note that `atexit` only removes the first occurrence of a function from the list, so if a function was placed in the list multiple times with `atexit`, it must also be removed from the list multiple times.

2.3 Commands for Getting Help

The entire text of this manual is available from the Octave prompt via the command *doc*. In addition, the documentation for individual user-written functions and variables is also available via the *help* command. This section describes the commands used for reading the manual and the documentation strings for user-supplied functions and variables. See Section 11.7 [Function Files], page 145, for more information about how to document the functions you write.

`help name` [Command]

Display the help text for *name*. If invoked without any arguments, `help` prints a list of all the available operators and functions.

For example, the command *help help* prints a short message describing the `help` command.

The help command can give you information about operators, but not the comma and semicolons that are used as command separators. To get help for those, you must type *help comma* or *help semicolon*.

See also: [doc], page 18, [lookfor], page 18, [which], page 105.

`doc function_name` [Command]

Display documentation for the function *function_name* directly from an on-line version of the printed manual, using the GNU Info browser. If invoked without any arguments, the manual is shown from the beginning.

For example, the command `doc rand` starts the GNU Info browser at the `rand` node in the on-line version of the manual.

Once the GNU Info browser is running, help for using it is available using the command `C-h`.

See also: [help], page 17.

`lookfor str` [Command]
`lookfor -all str` [Command]
`[func, helpstring] = lookfor (str)` [Function]
`[func, helpstring] = lookfor ('-all', str)` [Function]

Search for the string *str* in all functions found in the current function search path. By default, `lookfor` searches for *str* in the first sentence of the help string of each function found. The entire help text of each function can be searched if the '-all' argument is supplied. All searches are case insensitive.

Called with no output arguments, `lookfor` prints the list of matching functions to the terminal. Otherwise, the output arguments *func* and *helpstring* define the matching functions and the first sentence of each of their help strings.

The ability of `lookfor` to correctly identify the first sentence of the help text is dependent on the format of the function's help. All Octave core functions are correctly formatted, but the same can not be guaranteed for external packages and user-supplied functions. Therefore, the use of the '-all' argument may be necessary to find related functions that are not a part of Octave.

See also: [help], page 17, [doc], page 18, [which], page 105.

To see what is new in the current release of Octave, use the `news` function.

`news ()` [Function File]

Display the current NEWS file for Octave.

`info ()` [Function File]

Display contact information for the GNU Octave community.

`warranty ()` [Built-in Function]

Describe the conditions for copying and distributing Octave.

The following functions can be used to change which programs are used for displaying the documentation, and where the documentation can be found.

`val = info_file ()` [Built-in Function]
`old_val = info_file (new_val)` [Built-in Function]

Query or set the internal variable that specifies the name of the Octave info file. The default value is '`octave-home/info/octave.info`', in which *octave-home* is the root

directory of the Octave installation. The default value may be overridden by the environment variable `OCTAVE_INFO_FILE`, or the command line argument '`--info-file NAME`'.

See also: [info_program], page 19, [doc], page 18, [help], page 17, [makeinfo_program], page 19.

val = `info_program` () [Built-in Function]
old_val = `info_program` (*new_val*) [Built-in Function]

Query or set the internal variable that specifies the name of the info program to run. The default value is '*octave-home*`/libexec/octave/`*version*`/exec/`*arch*`/info`' in which *octave-home* is the root directory of the Octave installation, *version* is the Octave version number, and *arch* is the system type (for example, `i686-pc-linux-gnu`). The default value may be overridden by the environment variable `OCTAVE_INFO_PROGRAM`, or the command line argument '`--info-program NAME`'.

See also: [info_file], page 18, [doc], page 18, [help], page 17, [makeinfo_program], page 19.

val = `makeinfo_program` () [Built-in Function]
old_val = `makeinfo_program` (*new_val*) [Built-in Function]

Query or set the internal variable that specifies the name of the program that Octave runs to format help text containing Texinfo markup commands. The default value is `makeinfo`.

See also: [info_file], page 18, [info_program], page 19, [doc], page 18, [help], page 17.

val = `doc_cache_file` () [Built-in Function]
old_val = `doc_cache_file` (*new_val*) [Built-in Function]

Query or set the internal variable that specifies the name of the Octave documentation cache file. A cache file significantly improves the performance of the `lookfor` command. The default value is '*octave-home*`/share/octave/`*version*`/etc/doc-cache`', in which *octave-home* is the root directory of the Octave installation, and *version* is the Octave version number. The default value may be overridden by the environment variable `OCTAVE_DOC_CACHE_FILE`, or the command line argument '`--doc-cache-file NAME`'.

See also: [lookfor], page 18, [info_program], page 19, [doc], page 18, [help], page 17, [makeinfo_program], page 19.

val = `suppress_verbose_help_message` () [Built-in Function]
old_val = `suppress_verbose_help_message` (*new_val*) [Built-in Function]

Query or set the internal variable that controls whether Octave will add additional help information to the end of the output from the `help` command and usage messages for built-in commands.

2.4 Command Line Editing

Octave uses the GNU Readline library to provide an extensive set of command-line editing and history features. Only the most common features are described in this manual. In

addition, all of the editing functions can be bound to different key strokes at the user's discretion. This manual assumes no changes from the default Emacs bindings. See the GNU Readline Library manual for more information on customizing Readline and for a complete feature list.

To insert printing characters (letters, digits, symbols, etc.), simply type the character. Octave will insert the character at the cursor and advance the cursor forward.

Many of the command-line editing functions operate using control characters. For example, the character `Control-a` moves the cursor to the beginning of the line. To type `C-a`, hold down CTRL and then press A. In the following sections, control characters such as `Control-a` are written as `C-a`.

Another set of command-line editing functions use Meta characters. To type `M-u`, hold down the META key and press U. Depending on the keyboard, the META key may be labeled ALT or even WINDOWS. If your terminal does not have a META key, you can still type Meta characters using two-character sequences starting with `ESC`. Thus, to enter `M-u`, you would type ESC U. The `ESC` character sequences are also allowed on terminals with real Meta keys. In the following sections, Meta characters such as `Meta-u` are written as `M-u`.

2.4.1 Cursor Motion

The following commands allow you to position the cursor.

`C-b` Move back one character.

`C-f` Move forward one character.

DEL Delete the character to the left of the cursor.

`C-d` Delete the character underneath the cursor.

`M-f` Move forward a word.

`M-b` Move backward a word.

`C-a` Move to the start of the line.

`C-e` Move to the end of the line.

`C-l` Clear the screen, reprinting the current line at the top.

`C-_`
`C-/` Undo the last action. You can undo all the way back to an empty line.

`M-r` Undo all changes made to this line. This is like typing the 'undo' command enough times to get back to the beginning.

The above table describes the most basic possible keystrokes that you need in order to do editing of the input line. On most terminals, you can also use the left and right arrow keys in place of `C-f` and `C-b` to move forward and backward.

Notice how `C-f` moves forward a character, while `M-f` moves forward a word. It is a loose convention that control keystrokes operate on characters while meta keystrokes operate on words.

The function `clc` will allow you to clear the screen from within Octave programs.

`clc ()` [Built-in Function]
`home ()` [Built-in Function]

Clear the terminal screen and move the cursor to the upper left corner.

2.4.2 Killing and Yanking

Killing text means to delete the text from the line, but to save it away for later use, usually by *yanking* it back into the line. If the description for a command says that it 'kills' text, then you can be sure that you can get the text back in a different (or the same) place later.

Here is the list of commands for killing text.

C-k Kill the text from the current cursor position to the end of the line.

M-d Kill from the cursor to the end of the current word, or if between words, to the end of the next word.

M-DEL Kill from the cursor to the start of the previous word, or if between words, to the start of the previous word.

C-w Kill from the cursor to the previous whitespace. This is different than *M-DEL* because the word boundaries differ.

And, here is how to *yank* the text back into the line. Yanking means to copy the most-recently-killed text from the kill buffer.

C-y Yank the most recently killed text back into the buffer at the cursor.

M-y Rotate the kill-ring, and yank the new top. You can only do this if the prior command is *C-y* or *M-y*.

When you use a kill command, the text is saved in a *kill-ring*. Any number of consecutive kills save all of the killed text together, so that when you yank it back, you get it in one clean sweep. The kill ring is not line specific; the text that you killed on a previously typed line is available to be yanked back later, when you are typing another line.

2.4.3 Commands For Changing Text

The following commands can be used for entering characters that would otherwise have a special meaning (e.g., TAB, *C-q*, etc.), or for quickly correcting typing mistakes.

C-q
C-v Add the next character that you type to the line verbatim. This is how to insert things like *C-q* for example.

M-TAB Insert a tab character.

C-t Drag the character before the cursor forward over the character at the cursor, also moving the cursor forward. If the cursor is at the end of the line, then transpose the two characters before it.

M-t Drag the word behind the cursor past the word in front of the cursor moving the cursor over that word as well.

M-u Uppercase the characters following the cursor to the end of the current (or following) word, moving the cursor to the end of the word.

M-l Lowercase the characters following the cursor to the end of the current (or following) word, moving the cursor to the end of the word.

M-c Uppercase the character following the cursor (or the beginning of the next word if the cursor is between words), moving the cursor to the end of the word.

2.4.4 Letting Readline Type For You

The following commands allow Octave to complete command and variable names for you.

TAB Attempt to do completion on the text before the cursor. Octave can complete the names of commands and variables.

M-? List the possible completions of the text before the cursor.

val = `completion_append_char ()` [Built-in Function]
old_val = `completion_append_char (`*new_val*`)` [Built-in Function]

Query or set the internal character variable that is appended to successful command-line completion attempts. The default value is `" "` (a single space).

`completion_matches (`*hint*`)` [Built-in Function]

Generate possible completions given *hint*.

This function is provided for the benefit of programs like Emacs which might be controlling Octave and handling user input. The current command number is not incremented when this function is called. This is a feature, not a bug.

2.4.5 Commands For Manipulating The History

Octave normally keeps track of the commands you type so that you can recall previous commands to edit or execute them again. When you exit Octave, the most recent commands you have typed, up to the number specified by the variable `history_size`, are saved in a file. When Octave starts, it loads an initial list of commands from the file named by the variable `history_file`.

Here are the commands for simple browsing and searching the history list.

LFD
RET Accept the current line regardless of where the cursor is. If the line is non-empty, add it to the history list. If the line was a history line, then restore the history line to its original state.

C-p Move 'up' through the history list.

C-n Move 'down' through the history list.

M-< Move to the first line in the history.

M-> Move to the end of the input history, i.e., the line you are entering!

C-r Search backward starting at the current line and moving 'up' through the history as necessary. This is an incremental search.

C-s Search forward starting at the current line and moving 'down' through the history as necessary.

On most terminals, you can also use the up and down arrow keys in place of *C-p* and *C-n* to move through the history list.

In addition to the keyboard commands for moving through the history list, Octave provides three functions for viewing, editing, and re-running chunks of commands from the history list.

`history` *options* [Command]

If invoked with no arguments, `history` displays a list of commands that you have executed. Valid options are:

`-w` *file* Write the current history to the file *file*. If the name is omitted, use the default history file (normally '`~/.octave_hist`').

`-r` *file* Read the file *file*, replacing the current history list with its contents. If the name is omitted, use the default history file (normally '`~/.octave_hist`').

n Display only the most recent *n* lines of history.

`-q` Don't number the displayed lines of history. This is useful for cutting and pasting commands using the X Window System.

For example, to display the five most recent commands that you have typed without displaying line numbers, use the command *history -q 5*.

`edit_history` [*first*] [*last*] [Command]

If invoked with no arguments, `edit_history` allows you to edit the history list using the editor named by the variable `EDITOR`. The commands to be edited are first copied to a temporary file. When you exit the editor, Octave executes the commands that remain in the file. It is often more convenient to use `edit_history` to define functions rather than attempting to enter them directly on the command line. By default, the block of commands is executed as soon as you exit the editor. To avoid executing any commands, simply delete all the lines from the buffer before exiting the editor.

The `edit_history` command takes two optional arguments specifying the history numbers of first and last commands to edit. For example, the command

```
edit_history 13
```

extracts all the commands from the 13th through the last in the history list. The command

```
edit_history 13 169
```

only extracts commands 13 through 169. Specifying a larger number for the first command than the last command reverses the list of commands before placing them in the buffer to be edited. If both arguments are omitted, the previous command in the history list is used.

See also: [run_history], page 23.

`run_history` [*first*] [*last*] [Command]

Similar to `edit_history`, except that the editor is not invoked, and the commands are simply executed as they appear in the history list.

See also: [edit_history], page 23.

Octave also allows you customize the details of when, where, and how history is saved.

val = saving_history () [Built-in Function]
old_val = saving_history (*new_val*) [Built-in Function]

Query or set the internal variable that controls whether commands entered on the command line are saved in the history file.

See also: [history_file], page 24, [history_size], page 24, [history_timestamp_format_string], page 24.

val = history_file () [Built-in Function]
old_val = history_file (*new_val*) [Built-in Function]

Query or set the internal variable that specifies the name of the file used to store command history. The default value is '`~/.octave_hist`', but may be overridden by the environment variable `OCTAVE_HISTFILE`.

See also: [history_size], page 24, [saving_history], page 24, [history_timestamp_format_string], page 24.

val = history_size () [Built-in Function]
old_val = history_size (*new_val*) [Built-in Function]

Query or set the internal variable that specifies how many entries to store in the history file. The default value is `1024`, but may be overridden by the environment variable `OCTAVE_HISTSIZE`.

See also: [history_file], page 24, [history_timestamp_format_string], page 24, [saving_history], page 24.

val = history_timestamp_format_string () [Built-in Function]
old_val = history_timestamp_format_string (*new_val*) [Built-in Function]

Query or set the internal variable that specifies the format string for the comment line that is written to the history file when Octave exits. The format string is passed to `strftime`. The default value is

```
"# Octave VERSION, %a %b %d %H:%M:%S %Y %Z <USER@HOST>"
```

See also: [strftime], page 517, [history_file], page 24, [history_size], page 24, [saving_history], page 24.

val = EDITOR () [Built-in Function]
old_val = EDITOR (*new_val*) [Built-in Function]

Query or set the internal variable that specifies the editor to use with the `edit_history` command. The default value is taken from the environment variable `EDITOR` when Octave starts. If the environment variable is not initialized, `EDITOR` will be set to `"emacs"`.

See also: [edit_history], page 23.

2.4.6 Customizing readline

Octave uses the GNU Readline library for command-line editing and history features. Readline is very flexible and can be modified through a configuration file of commands (See the GNU Readline library for the exact command syntax). The default configuration file is normally '`~/.inputrc`'.

Octave provides two commands for initializing Readline and thereby changing the command line behavior.

`read_readline_init_file (file)` [Built-in Function]

Read the readline library initialization file *file*. If *file* is omitted, read the default initialization file (normally '`~/.inputrc`').

See Section "Readline Init File" in *GNU Readline Library*, for details.

`re_read_readline_init_file ()` [Built-in Function]

Re-read the last readline library initialization file that was read. See Section "Readline Init File" in *GNU Readline Library*, for details.

2.4.7 Customizing the Prompt

The following variables are available for customizing the appearance of the command-line prompts. Octave allows the prompt to be customized by inserting a number of backslash-escaped special characters that are decoded as follows:

'`\t`' The time.

'`\d`' The date.

'`\n`' Begins a new line by printing the equivalent of a carriage return followed by a line feed.

'`\s`' The name of the program (usually just '`octave`').

'`\w`' The current working directory.

'`\W`' The basename of the current working directory.

'`\u`' The username of the current user.

'`\h`' The hostname, up to the first '.'.

'`\H`' The hostname.

'`\#`' The command number of this command, counting from when Octave starts.

'`\!`' The history number of this command. This differs from '`\#`' by the number of commands in the history list when Octave starts.

'`\$`' If the effective UID is 0, a '`#`', otherwise a '`$`'.

'`\nnn`' The character whose character code in octal is *nnn*.

'`\\`' A backslash.

`val = PS1 ()` [Built-in Function]
`old_val = PS1 (new_val)` [Built-in Function]

Query or set the primary prompt string. When executing interactively, Octave displays the primary prompt when it is ready to read a command.

The default value of the primary prompt string is `"\s:\#> "`. To change it, use a command like

```
octave:13> PS1 ("\\u@\\H> ")
```

which will result in the prompt '`boris@kremvax> `' for the user '`boris`' logged in on the host '`kremvax.kgb.su`'. Note that two backslashes are required to enter a backslash into a double-quoted character string. See Chapter 5 [Strings], page 55.

See also: [PS2], page 26, [PS4], page 26.

val = `PS2` () [Built-in Function]
old_val = `PS2` (*new_val*) [Built-in Function]

Query or set the secondary prompt string. The secondary prompt is printed when Octave is expecting additional input to complete a command. For example, if you are typing a `for` loop that spans several lines, Octave will print the secondary prompt at the beginning of each line after the first. The default value of the secondary prompt string is `"> "`.

See also: [PS1], page 25, [PS4], page 26.

val = `PS4` () [Built-in Function]
old_val = `PS4` (*new_val*) [Built-in Function]

Query or set the character string used to prefix output produced when echoing commands is enabled. The default value is `"+ "`. See Section 2.4.8 [Diary and Echo Commands], page 26, for a description of echoing commands.

See also: [echo], page 26, [echo_executing_commands], page 27, [PS1], page 25, [PS2], page 26.

2.4.8 Diary and Echo Commands

Octave's diary feature allows you to keep a log of all or part of an interactive session by recording the input you type and the output that Octave produces in a separate file.

`diary` *options* [Command]

Record a list of all commands *and* the output they produce, mixed together just as you see them on your terminal. Valid options are:

`on` Start recording your session in a file called '`diary`' in your current working directory.

`off` Stop recording your session in the diary file.

`file` Record your session in the file named *file*.

With no arguments, `diary` toggles the current diary state.

Sometimes it is useful to see the commands in a function or script as they are being evaluated. This can be especially helpful for debugging some kinds of problems.

`echo` *options* [Command]

Control whether commands are displayed as they are executed. Valid options are:

`on` Enable echoing of commands as they are executed in script files.

`off` Disable echoing of commands as they are executed in script files.

`on all` Enable echoing of commands as they are executed in script files and functions.

`off all` Disable echoing of commands as they are executed in script files and functions.

With no arguments, `echo` toggles the current echo state.

val = `echo_executing_commands ()` [Built-in Function]
old_val = `echo_executing_commands (`*new_val*`)` [Built-in Function]

Query or set the internal variable that controls the echo state. It may be the sum of the following values:

1 Echo commands read from script files.

2 Echo commands from functions.

4 Echo commands read from command line.

More than one state can be active at once. For example, a value of 3 is equivalent to the command *echo on all*.

The value of `echo_executing_commands` may be set by the *echo* command or the command line option `--echo-commands`.

2.5 How Octave Reports Errors

Octave reports two kinds of errors for invalid programs.

A *parse error* occurs if Octave cannot understand something you have typed. For example, if you misspell a keyword,

```
octave:13> function y = f (x) y = x***2; endfunction
```

Octave will respond immediately with a message like this:

```
parse error:

  syntax error

>>> function y = f (x) y = x***2; endfunction
                              ^
```

For most parse errors, Octave uses a caret ('^') to mark the point on the line where it was unable to make sense of your input. In this case, Octave generated an error message because the keyword for exponentiation (`**`) was misspelled. It marked the error at the third '`*`' because the code leading up to this was correct but the final '`*`' was not understood.

Another class of error message occurs at evaluation time. These errors are called *run-time errors*, or sometimes *evaluation errors*, because they occur when your program is being *run*, or *evaluated*. For example, if after correcting the mistake in the previous function definition, you type

```
octave:13> f ()
```

Octave will respond with

```
error: ‘x’ undefined near line 1 column 24
error: called from:
error:   f at line 1, column 22
```

This error message has several parts, and gives quite a bit of information to help you locate the source of the error. The messages are generated from the point of the innermost error, and provide a traceback of enclosing expressions and function calls.

In the example above, the first line indicates that a variable named ‘x’ was found to be undefined near line 1 and column 24 of some function or expression. For errors occurring within functions, lines are counted from the beginning of the file containing the function definition. For errors occurring outside of an enclosing function, the line number indicates the input line number, which is usually displayed in the primary prompt string.

The second and third lines of the error message indicate that the error occurred within the function `f`. If the function `f` had been called from within another function, for example, `g`, the list of errors would have ended with one more line:

```
error:   g at line 1, column 17
```

These lists of function calls make it fairly easy to trace the path your program took before the error occurred, and to correct the error before trying again.

2.6 Executable Octave Programs

Once you have learned Octave, you may want to write self-contained Octave scripts, using the ‘`#!`’ script mechanism. You can do this on GNU systems and on many Unix systems[1].

Self-contained Octave scripts are useful when you want to write a program which users can invoke without knowing that the program is written in the Octave language. Octave scripts are also used for batch processing of data files. Once an algorithm has been developed and tested in the interactive portion of Octave, it can be committed to an executable script and used again and again on new data files.

As a trivial example of an executable Octave script, you might create a text file named ‘`hello`’, containing the following lines:

```
#! octave-interpreter-name -qf
# a sample Octave program
printf ("Hello, world!\n");
```

(where *octave-interpreter-name* should be replaced with the full path and name of your Octave binary). Note that this will only work if ‘`#!`’ appears at the very beginning of the file. After making the file executable (with the `chmod` command on Unix systems), you can simply type:

```
hello
```

at the shell, and the system will arrange to run Octave as if you had typed:

```
octave hello
```

The line beginning with ‘`#!`’ lists the full path and filename of an interpreter to be run, and an optional initial command line argument to pass to that interpreter. The operating system then runs the interpreter with the given argument and the full argument list of

[1] The ‘`#!`’ mechanism works on Unix systems derived from Berkeley Unix, System V Release 4, and some System V Release 3 systems.

the executed program. The first argument in the list is the full file name of the Octave executable. The rest of the argument list will either be options to Octave, or data files, or both. The '-qf' options are usually specified in stand-alone Octave programs to prevent them from printing the normal startup message, and to keep them from behaving differently depending on the contents of a particular user's '~/.octaverc' file. See Section 2.1 [Invoking Octave from the Command Line], page 13.

Note that some operating systems may place a limit on the number of characters that are recognized after '#!'. Also, the arguments appearing in a '#!' line are parsed differently by various shells/systems. The majority of them group all the arguments together in one string and pass it to the interpreter as a single argument. In this case, the following script:

```
#! octave-interpreter-name -q -f # comment
```

is equivalent to typing at the command line:

```
octave "-q -f # comment"
```

which will produce an error message. Unfortunately, it is not possible for Octave to determine whether it has been called from the command line or from a '#!' script, so some care is needed when using the '#!' mechanism.

Note that when Octave is started from an executable script, the built-in function `argv` returns a cell array containing the command line arguments passed to the executable Octave script, not the arguments passed to the Octave interpreter on the '#!' line of the script. For example, the following program will reproduce the command line that was used to execute the script, not '-qf'.

```
#! /bin/octave -qf
printf ("%s", program_name ());
arg_list = argv ();
for i = 1:nargin
  printf (" %s", arg_list{i});
endfor
printf ("\n");
```

2.7 Comments in Octave Programs

A *comment* is some text that is included in a program for the sake of human readers, and which is NOT an executable part of the program. Comments can explain what the program does, and how it works. Nearly all programming languages have provisions for comments, because programs are typically hard to understand without them.

2.7.1 Single Line Comments

In the Octave language, a comment starts with either the sharp sign character, '#', or the percent symbol '%' and continues to the end of the line. Any text following the sharp sign or percent symbol is ignored by the Octave interpreter and not executed. The following example shows whole line and partial line comments.

```
function countdown
  # Count down for main rocket engines
  disp(3);
  disp(2);
  disp(1);
  disp("Blast Off!");  # Rocket leaves pad
endfunction
```

2.7.2 Block Comments

Entire blocks of code can be commented by enclosing the code between matching '`#{`' and '`#}`' or '`%{`' and '`%}`' markers. For example,

```
function quick_countdown
  # Count down for main rocket engines
  disp(3);
 #{
  disp(2);
  disp(1);
 #}
  disp("Blast Off!");  # Rocket leaves pad
endfunction
```

will produce a very quick countdown from '3' to 'Blast Off' as the lines "`disp(2);`" and "`disp(1);`" won't be executed.

2.7.3 Comments and the Help System

The `help` command (see Section 2.3 [Getting Help], page 17) is able to find the first block of comments in a function and return those as a documentation string. This means that the same commands used to get help on built-in functions are available for properly formatted user-defined functions. For example, after defining the function `f` below,

```
function xdot = f (x, t)

# usage: f (x, t)
#
# This function defines the right-hand
# side functions for a set of nonlinear
# differential equations.

  r = 0.25;
  ...
endfunction
```

the command *`help f`* produces the output

```
 usage: f (x, t)

 This function defines the right-hand
 side functions for a set of nonlinear
 differential equations.
```

Although it is possible to put comment lines into keyboard-composed, throw-away Octave programs, it usually isn't very useful because the purpose of a comment is to help you or another person understand the program at a later time.

The `help` parser currently only recognizes single line comments (see Section 2.7.1 [Single Line Comments], page 29) and not block comments for the initial help text.

3 Data Types

All versions of Octave include a number of built-in data types, including real and complex scalars and matrices, character strings, a data structure type, and an array that can contain all data types.

It is also possible to define new specialized data types by writing a small amount of C++ code. On some systems, new data types can be loaded dynamically while Octave is running, so it is not necessary to recompile all of Octave just to add a new type. See Appendix A [Dynamically Linked Functions], page 557, for more information about Octave's dynamic linking capabilities. Section 3.2 [User-defined Data Types], page 35 describes what you must do to define a new data type for Octave.

`typeinfo` (*expr*) [Built-in Function]

Return the type of the expression *expr*, as a string. If *expr* is omitted, return an array of strings containing all the currently installed data types.

3.1 Built-in Data Types

The standard built-in data types are real and complex scalars and matrices, ranges, character strings, a data structure type, and cell arrays. Additional built-in data types may be added in future versions. If you need a specialized data type that is not currently provided as a built-in type, you are encouraged to write your own user-defined data type and contribute it for distribution in a future release of Octave.

The data type of a variable can be determined and changed through the use of the following functions.

`class` (*expr*) [Built-in Function]
`class` (*s*, *id*) [Built-in Function]
`class` (*s*, *id*, *p*, ...) [Built-in Function]

Return the class of the expression *expr* or create a class with fields from structure *s* and name (string) *id*. Additional arguments name a list of parent classes from which the new class is derived.

`isa` (*x*, *class*) [Function File]

Return true if *x* is a value from the class *class*.

`cast` (*val*, *type*) [Function File]

Convert *val* to data type *type*.

See also: [int8], page 45, [uint8], page 45, [int16], page 45, [uint16], page 45, [int32], page 45, [uint32], page 45, [int64], page 45, [uint64], page 45, [double], page 39.

`typecast` (*x*, *type*) [Loadable Function]

Convert from one datatype to another without changing the underlying data. The argument *type* defines the type of the return argument and must be one of 'uint8', 'uint16', 'uint32', 'uint64', 'int8', 'int16', 'int32', 'int64', 'single' or 'double'.

An example of the use of typecast on a little-endian machine is

```
x = uint16 ([1, 65535]);
typecast (x, 'uint8')
⇒ [   0,   1, 255, 255]
```

See also: [cast], page 33, [swapbytes], page 34.

swapbytes (*x*) [Function File]

Swaps the byte order on values, converting from little endian to big endian and vice versa. For example

```
swapbytes (uint16 (1:4))
⇒ [   256   512   768  1024]
```

See also: [typecast], page 33, [cast], page 33.

3.1.1 Numeric Objects

Octave's built-in numeric objects include real, complex, and integer scalars and matrices. All built-in floating point numeric data is currently stored as double precision numbers. On systems that use the IEEE floating point format, values in the range of approximately 2.2251×10^{-308} to 1.7977×10^{308} can be stored, and the relative precision is approximately 2.2204×10^{-16}. The exact values are given by the variables `realmin`, `realmax`, and `eps`, respectively.

Matrix objects can be of any size, and can be dynamically reshaped and resized. It is easy to extract individual rows, columns, or submatrices using a variety of powerful indexing features. See Section 8.1 [Index Expressions], page 107.

See Chapter 4 [Numeric Data Types], page 39, for more information.

3.1.2 Missing Data

It is possible to represent missing data explicitly in Octave using `NA` (short for "Not Available"). Missing data can only be represented when data is represented as floating point numbers. In this case missing data is represented as a special case of the representation of `NaN`.

NA [Built-in Function]
NA (*n*) [Built-in Function]
NA (*n*, *m*) [Built-in Function]
NA (*n*, *m*, *k*, . . .) [Built-in Function]
NA (. . ., *class*) [Built-in Function]

Return a scalar, matrix, or N-dimensional array whose elements are all equal to the special constant used to designate missing values.

Note that NA always compares not equal to NA (NA != NA). To find NA values, use the `isna` function.

When called with no arguments, return a scalar with the value 'NA'. When called with a single argument, return a square matrix with the dimension specified. When called with more than one scalar argument the first two arguments are taken as the number of rows and columns and any further arguments specify additional matrix dimensions. The optional argument *class* specifies the return type and may be either "double" or "single".

See also: [isna], page 35.

isna (*x*) [Mapping Function]

Return 1 for elements of *x* that are NA (missing) values and zero otherwise. For example,

```
isna ([13, Inf, NA, NaN])
     ⇒ [ 0, 0, 1, 0 ]
```

See also: [isnan], page 274.

3.1.3 String Objects

A character string in Octave consists of a sequence of characters enclosed in either double-quote or single-quote marks. Internally, Octave currently stores strings as matrices of characters. All the indexing operations that work for matrix objects also work for strings.

See Chapter 5 [Strings], page 55, for more information.

3.1.4 Data Structure Objects

Octave's data structure type can help you to organize related objects of different types. The current implementation uses an associative array with indices limited to strings, but the syntax is more like C-style structures.

See Section 6.1 [Data Structures], page 77, for more information.

3.1.5 Cell Array Objects

A Cell Array in Octave is general array that can hold any number of different data types.

See Section 6.2 [Cell Arrays], page 85, for more information.

3.2 User-defined Data Types

Someday I hope to expand this to include a complete description of Octave's mechanism for managing user-defined data types. Until this feature is documented here, you will have to make do by reading the code in the '`ov.h`', '`ops.h`', and related files from Octave's '`src`' directory.

3.3 Object Sizes

The following functions allow you to determine the size of a variable or expression. These functions are defined for all objects. They return −1 when the operation doesn't make sense. For example, Octave's data structure type doesn't have rows or columns, so the `rows` and `columns` functions return −1 for structure arguments.

ndims (*a*) [Built-in Function]

Returns the number of dimensions of array *a*. For any array, the result will always be larger than or equal to 2. Trailing singleton dimensions are not counted.

columns (*a*) [Built-in Function]

Return the number of columns of *a*.

See also: [size], page 36, [numel], page 36, [rows], page 36, [length], page 36, [isscalar], page 52, [isvector], page 52, [ismatrix], page 52.

`rows (a)` [Built-in Function]

Return the number of rows of *a*.

See also: [size], page 36, [numel], page 36, [columns], page 35, [length], page 36, [isscalar], page 52, [isvector], page 52, [ismatrix], page 52.

`numel (a)` [Built-in Function]

Returns the number of elements in the object *a*.

See also: [size], page 36.

`length (a)` [Built-in Function]

Return the 'length' of the object *a*. For matrix objects, the length is the number of rows or columns, whichever is greater (this odd definition is used for compatibility with MATLAB).

`size (a, n)` [Built-in Function]

Return the number rows and columns of *a*.

With one input argument and one output argument, the result is returned in a row vector. If there are multiple output arguments, the number of rows is assigned to the first, and the number of columns to the second, etc. For example,

```
size ([1, 2; 3, 4; 5, 6])
     ⇒ [ 3, 2 ]

[nr, nc] = size ([1, 2; 3, 4; 5, 6])
     ⇒ nr = 3
     ⇒ nc = 2
```

If given a second argument, `size` will return the size of the corresponding dimension. For example

```
size ([1, 2; 3, 4; 5, 6], 2)
     ⇒ 2
```

returns the number of columns in the given matrix.

See also: [numel], page 36.

`isempty (a)` [Built-in Function]

Return 1 if *a* is an empty matrix (either the number of rows, or the number of columns, or both are zero). Otherwise, return 0.

`isnull (x)` [Built-in Function]

Return 1 if *x* is a special null matrix, string or single quoted string. Indexed assignment with such a value as right-hand side should delete array elements. This function should be used when overloading indexed assignment for user-defined classes instead of `isempty`, to distinguish the cases:

`A(I) = []` This should delete elements if `I` is nonempty.

`X = []; A(I) = X`
This should give an error if `I` is nonempty.

`sizeof (val)` [Built-in Function]

Return the size of *val* in bytes

`size_equal (a, b, ...)` [Built-in Function]

Return true if the dimensions of all arguments agree. Trailing singleton dimensions are ignored. Called with a single argument, size_equal returns true.

See also: [size], page 36, [numel], page 36.

`squeeze (x)` [Built-in Function]

Remove singleton dimensions from *x* and return the result. Note that for compatibility with MATLAB, all objects have a minimum of two dimensions and row vectors are left unchanged.

4 Numeric Data Types

A *numeric constant* may be a scalar, a vector, or a matrix, and it may contain complex values.

The simplest form of a numeric constant, a scalar, is a single number that can be an integer, a decimal fraction, a number in scientific (exponential) notation, or a complex number. Note that by default numeric constants are represented within Octave in double-precision floating point format (complex constants are stored as pairs of double-precision floating point values). It is however possible to represent real integers as described in Section 4.4 [Integer Data Types], page 45. Here are some examples of real-valued numeric constants, which all have the same value:

```
105
1.05e+2
1050e-1
```

To specify complex constants, you can write an expression of the form

```
3 + 4i
3.0 + 4.0i
0.3e1 + 40e-1i
```

all of which are equivalent. The letter '`i`' in the previous example stands for the pure imaginary constant, defined as $\sqrt{-1}$.

For Octave to recognize a value as the imaginary part of a complex constant, a space must not appear between the number and the '`i`'. If it does, Octave will print an error message, like this:

```
octave:13> 3 + 4 i

parse error:

  syntax error

>>> 3 + 4 i
          ^
```

You may also use '`j`', '`I`', or '`J`' in place of the '`i`' above. All four forms are equivalent.

`double` (*x*) [Built-in Function]

Convert *x* to double precision type.

See also: [single], page 44.

`complex` (*x*) [Built-in Function]
`complex` (*re*, *im*) [Built-in Function]

Return a complex result from real arguments. With 1 real argument *x*, return the complex result `x + 0i`. With 2 real arguments, return the complex result `re + im`. `complex` can often be more convenient than expressions such as `a + i*b`. For example:

```
complex ([1, 2], [3, 4])
⇒
   1 + 3i   2 + 4i
```

See also: [real], page 295, [imag], page 295, [iscomplex], page 52.

4.1 Matrices

It is easy to define a matrix of values in Octave. The size of the matrix is determined automatically, so it is not necessary to explicitly state the dimensions. The expression

```
a = [1, 2; 3, 4]
```

results in the matrix

$$a = \begin{bmatrix} 1 & 2 \\ 3 & 4 \end{bmatrix}$$

Elements of a matrix may be arbitrary expressions, provided that the dimensions all make sense when combining the various pieces. For example, given the above matrix, the expression

```
[ a, a ]
```

produces the matrix

```
ans =

  1  2  1  2
  3  4  3  4
```

but the expression

```
[ a, 1 ]
```

produces the error

```
error: number of rows must match (1 != 2) near line 13, column 6
```

(assuming that this expression was entered as the first thing on line 13, of course).

Inside the square brackets that delimit a matrix expression, Octave looks at the surrounding context to determine whether spaces and newline characters should be converted into element and row separators, or simply ignored, so an expression like

```
a = [ 1 2
      3 4 ]
```

will work. However, some possible sources of confusion remain. For example, in the expression

```
[ 1 - 1 ]
```

the '`-`' is treated as a binary operator and the result is the scalar 0, but in the expression

```
[ 1 -1 ]
```

the '`-`' is treated as a unary operator and the result is the vector `[ 1, -1 ]`. Similarly, the expression

```
[ sin (pi) ]
```

will be parsed as

```
[ sin, (pi) ]
```

and will result in an error since the `sin` function will be called with no arguments. To get around this, you must omit the space between `sin` and the opening parenthesis, or enclose the expression in a set of parentheses:

```
[ (sin (pi)) ]
```

Whitespace surrounding the single quote character ('`'`', used as a transpose operator and for delimiting character strings) can also cause confusion. Given `a = 1`, the expression

```
[ 1 a' ]
```

results in the single quote character being treated as a transpose operator and the result is the vector `[ 1, 1 ]`, but the expression

```
[ 1 a ' ]
```

produces the error message

```
parse error:

  syntax error

>>> [ 1 a ' ]
              ^
```

because not doing so would cause trouble when parsing the valid expression

```
[ a 'foo' ]
```

For clarity, it is probably best to always use commas and semicolons to separate matrix elements and rows.

When you type a matrix or the name of a variable whose value is a matrix, Octave responds by printing the matrix in with neatly aligned rows and columns. If the rows of the matrix are too large to fit on the screen, Octave splits the matrix and displays a header before each section to indicate which columns are being displayed. You can use the following variables to control the format of the output.

val = `output_max_field_width` () [Built-in Function]
old_val = `output_max_field_width` (*new_val*) [Built-in Function]

Query or set the internal variable that specifies the maximum width of a numeric output field.

See also: [format], page 175, [output_precision], page 41.

val = `output_precision` () [Built-in Function]
old_val = `output_precision` (*new_val*) [Built-in Function]

Query or set the internal variable that specifies the minimum number of significant figures to display for numeric output.

See also: [format], page 175, [output_max_field_width], page 41.

It is possible to achieve a wide range of output styles by using different values of `output_precision` and `output_max_field_width`. Reasonable combinations can be set using the `format` function. See Section 14.1 [Basic Input and Output], page 175.

val = `split_long_rows` () [Built-in Function]
old_val = `split_long_rows` (*new_val*) [Built-in Function]

Query or set the internal variable that controls whether rows of a matrix may be split when displayed to a terminal window. If the rows are split, Octave will display the matrix in a series of smaller pieces, each of which can fit within the limits of your terminal width and each set of rows is labeled so that you can easily see which columns are currently being displayed. For example:

```
octave:13> rand (2,10)
ans =

 Columns 1 through 6:

  0.75883  0.93290  0.40064  0.43818  0.94958  0.16467
  0.75697  0.51942  0.40031  0.61784  0.92309  0.40201

 Columns 7 through 10:

  0.90174  0.11854  0.72313  0.73326
  0.44672  0.94303  0.56564  0.82150
```

Octave automatically switches to scientific notation when values become very large or very small. This guarantees that you will see several significant figures for every value in a matrix. If you would prefer to see all values in a matrix printed in a fixed point format, you can set the built-in variable `fixed_point_format` to a nonzero value. But doing so is not recommended, because it can produce output that can easily be misinterpreted.

val = **fixed_point_format** () [Built-in Function]
old_val = **fixed_point_format** (*new_val*) [Built-in Function]

Query or set the internal variable that controls whether Octave will use a scaled format to print matrix values such that the largest element may be written with a single leading digit with the scaling factor is printed on the first line of output. For example,

```
octave:1> logspace (1, 7, 5)'
ans =

  1.0e+07  *

  0.00000
  0.00003
  0.00100
  0.03162
  1.00000
```

Notice that first value appears to be zero when it is actually 1. For this reason, you should be careful when setting `fixed_point_format` to a nonzero value.

4.1.1 Empty Matrices

A matrix may have one or both dimensions zero, and operations on empty matrices are handled as described by Carl de Boor in *An Empty Exercise*, SIGNUM, Volume 25, pages 2-6, 1990 and C. N. Nett and W. M. Haddad, in *A System-Theoretic Appropriate Realization of the Empty Matrix Concept*, IEEE Transactions on Automatic Control, Volume 38, Number 5, May 1993. Briefly, given a scalar s, an $m \times n$ matrix $M_{m\times n}$, and an $m \times n$ empty

matrix $[\,]_{m\times n}$ (with either one or both dimensions equal to zero), the following are true:

$$s \cdot [\,]_{m\times n} = [\,]_{m\times n} \cdot s = [\,]_{m\times n}$$
$$[\,]_{m\times n} + [\,]_{m\times n} = [\,]_{m\times n}$$
$$[\,]_{0\times m} \cdot M_{m\times n} = [\,]_{0\times n}$$
$$M_{m\times n} \cdot [\,]_{n\times 0} = [\,]_{m\times 0}$$
$$[\,]_{m\times 0} \cdot [\,]_{0\times n} = 0_{m\times n}$$

By default, dimensions of the empty matrix are printed along with the empty matrix symbol, '`[]`'. The built-in variable `print_empty_dimensions` controls this behavior.

val = **print_empty_dimensions** () [Built-in Function]
old_val = **print_empty_dimensions** (*new_val*) [Built-in Function]

Query or set the internal variable that controls whether the dimensions of empty matrices are printed along with the empty matrix symbol, '`[]`'. For example, the expression

```
zeros (3, 0)
```

will print

```
ans = [](3x0)
```

Empty matrices may also be used in assignment statements as a convenient way to delete rows or columns of matrices. See Section 8.6 [Assignment Expressions], page 115.

When Octave parses a matrix expression, it examines the elements of the list to determine whether they are all constants. If they are, it replaces the list with a single matrix constant.

4.2 Ranges

A *range* is a convenient way to write a row vector with evenly spaced elements. A range expression is defined by the value of the first element in the range, an optional value for the increment between elements, and a maximum value which the elements of the range will not exceed. The base, increment, and limit are separated by colons (the '`:`' character) and may contain any arithmetic expressions and function calls. If the increment is omitted, it is assumed to be 1. For example, the range

```
1 : 5
```

defines the set of values '`[ 1, 2, 3, 4, 5 ]`', and the range

```
1 : 3 : 5
```

defines the set of values '`[ 1, 4 ]`'.

Although a range constant specifies a row vector, Octave does *not* convert range constants to vectors unless it is necessary to do so. This allows you to write a constant like '`1 : 10000`' without using 80,000 bytes of storage on a typical 32-bit workstation.

Note that the upper (or lower, if the increment is negative) bound on the range is not always included in the set of values, and that ranges defined by floating point values can produce surprising results because Octave uses floating point arithmetic to compute the values in the range. If it is important to include the endpoints of a range and the number of

elements is known, you should use the `linspace` function instead (see Section 16.4 [Special Utility Matrices], page 284).

When adding a scalar to a range, subtracting a scalar from it (or subtracting a range from a scalar) and multiplying by scalar, Octave will attempt to avoid unpacking the range and keep the result as a range, too, if it can determine that it is safe to do so. For instance, doing

```
a = 2*(1:1e7) - 1;
```

will produce the same result as '`1:2:2e7-1`', but without ever forming a vector with ten million elements.

Using zero as an increment in the colon notation, as '`1:0:1`' is not allowed, because a division by zero would occur in determining the number of range elements. However, ranges with zero increment (i.e., all elements equal) are useful, especially in indexing, and Octave allows them to be constructed using the built-in function *ones*. Note that because a range must be a row vector, '`ones (1, 10)`' produces a range, while '`ones (10, 1)`' does not.

When Octave parses a range expression, it examines the elements of the expression to determine whether they are all constants. If they are, it replaces the range expression with a single range constant.

4.3 Single Precision Data Types

Octave includes support for single precision data types, and most of the functions in Octave accept single precision values and return single precision answers. A single precision variable is created with the `single` function.

`single` (*x*) [Built-in Function]

Convert *x* to single precision type.

See also: [double], page 39.

for example

```
sngl = single (rand (2, 2))
     ⇒ sngl =
        0.37569   0.92982
        0.11962   0.50876
class (sngl)
    ⇒ single
```

Many functions can also return single precision values directly. For example

```
ones (2, 2, "single")
zeros (2, 2, "single")
eye (2, 2,  "single")
rand (2, 2, "single")
NaN (2, 2, "single")
NA (2, 2, "single")
Inf (2, 2, "single")
```

will all return single precision matrices.

4.4 Integer Data Types

Octave supports integer matrices as an alternative to using double precision. It is possible to use both signed and unsigned integers represented by 8, 16, 32, or 64 bits. It should be noted that most computations require floating point data, meaning that integers will often change type when involved in numeric computations. For this reason integers are most often used to store data, and not for calculations.

In general most integer matrices are created by casting existing matrices to integers. The following example shows how to cast a matrix into 32 bit integers.

```
float = rand (2, 2)
     ⇒ float = 0.37569   0.92982
               0.11962   0.50876
integer = int32 (float)
     ⇒ integer = 0  1
                 0  1
```

As can be seen, floating point values are rounded to the nearest integer when converted.

isinteger (*x*) [Built-in Function]

Return true if *x* is an integer object (int8, uint8, int16, etc.). Note that `isinteger (14)` is false because numeric constants in Octave are double precision floating point values.

See also: [isreal], page 52, [isnumeric], page 52, [class], page 33, [isa], page 33.

int8 (*x*) [Built-in Function]

Convert *x* to 8-bit integer type.

uint8 (*x*) [Built-in Function]

Convert *x* to unsigned 8-bit integer type.

int16 (*x*) [Built-in Function]

Convert *x* to 16-bit integer type.

uint16 (*x*) [Built-in Function]

Convert *x* to unsigned 16-bit integer type.

int32 (*x*) [Built-in Function]

Convert *x* to 32-bit integer type.

uint32 (*x*) [Built-in Function]

Convert *x* to unsigned 32-bit integer type.

int64 (*x*) [Built-in Function]

Convert *x* to 64-bit integer type.

uint64 (*x*) [Built-in Function]

Convert *x* to unsigned 64-bit integer type.

intmax (*type*) [Built-in Function]

Return the largest integer that can be represented in an integer type. The variable *type* can be

`int8`	signed 8-bit integer.
`int16`	signed 16-bit integer.
`int32`	signed 32-bit integer.
`int64`	signed 64-bit integer.
`uint8`	unsigned 8-bit integer.
`uint16`	unsigned 16-bit integer.
`uint32`	unsigned 32-bit integer.
`uint64`	unsigned 64-bit integer.

The default for *type* is `uint32`.

See also: [intmin], page 46, [bitmax], page 48.

`intmin` (*type*) [Built-in Function]

Return the smallest integer that can be represented in an integer type. The variable *type* can be

`int8`	signed 8-bit integer.
`int16`	signed 16-bit integer.
`int32`	signed 32-bit integer.
`int64`	signed 64-bit integer.
`uint8`	unsigned 8-bit integer.
`uint16`	unsigned 16-bit integer.
`uint32`	unsigned 32-bit integer.
`uint64`	unsigned 64-bit integer.

The default for *type* is `uint32`.

See also: [intmax], page 45, [bitmax], page 48.

`intwarning` (*action*) [Function File]
`intwarning` (*s*) [Function File]
s = `intwarning` (...) [Function File]

Control the state of the warning for integer conversions and math operations.

"query" The state of the Octave integer conversion and math warnings is queried. If there is no output argument, then the state is printed. Otherwise it is returned in a structure with the fields "identifier" and "state".

```
intwarning ("query")
The state of warning "Octave:int-convert-nan" is "off"
The state of warning "Octave:int-convert-non-int-val" is "off"
The state of warning "Octave:int-convert-overflow" is "off"
The state of warning "Octave:int-math-overflow" is "off"
```

"on" Turn integer conversion and math warnings "on". If there is no output argument, then nothing is printed. Otherwise the original state of the state of the integer conversion and math warnings is returned in a structure array.

"off" Turn integer conversion and math warnings "on". If there is no output argument, then nothing is printed. Otherwise the original state of the state of the integer conversion and math warnings is returned in a structure array.

The original state of the integer warnings can be restored by passing the structure array returned by `intwarning` to a later call to `intwarning`. For example

```
s = intwarning ("off");
...
intwarning (s);
```

See also: [warning], page 166.

4.4.1 Integer Arithmetic

While many numerical computations can't be carried out in integers, Octave does support basic operations like addition and multiplication on integers. The operators `+`, `-`, `.*`, and `./` work on integers of the same type. So, it is possible to add two 32 bit integers, but not to add a 32 bit integer and a 16 bit integer.

The arithmetic operations on integers are performed by casting the integer values to double precision values, performing the operation, and then re-casting the values back to the original integer type. As the double precision type of Octave is only capable of representing integers with up to 53 bits of precision, it is not possible to perform arithmetic with 64 bit integer types.

When doing integer arithmetic one should consider the possibility of underflow and overflow. This happens when the result of the computation can't be represented using the chosen integer type. As an example it is not possible to represent the result of $10 - 20$ when using unsigned integers. Octave makes sure that the result of integer computations is the integer that is closest to the true result. So, the result of $10 - 20$ when using unsigned integers is zero.

When doing integer division Octave will round the result to the nearest integer. This is different from most programming languages, where the result is often floored to the nearest integer. So, the result of `int32(5)./int32(8)` is `1`.

`idivide` (*x*, *y*, *op*) [Function File]

Integer division with different round rules. The standard behavior of the an integer division such as `a ./ b` is to round the result to the nearest integer. This is not always the desired behavior and `idivide` permits integer element-by-element division to be performed with different treatment for the fractional part of the division as determined by the *op* flag. *op* is a string with one of the values:

"fix" Calculate `a ./ b` with the fractional part rounded towards zero.

"round" Calculate `a ./ b` with the fractional part rounded towards the nearest integer.

"floor" Calculate `a ./ b` with the fractional part rounded downwards.

"ceil" Calculate `a ./ b` with the fractional part rounded upwards.

If *op* is not given it is assumed that it is `"fix"`. An example demonstrating these rounding rules is

```
idivide (int8 ([-3, 3]), int8 (4), "fix")
⇒ int8 ([0, 0])
idivide (int8 ([-3, 3]), int8 (4), "round")
⇒ int8 ([-1, 1])
idivide (int8 ([-3, 3]), int8 (4), "ceil")
⇒ int8 ([0, 1])
idivide (int8 ([-3, 3]), int8 (4), "floor")
⇒ int8 ([-1, 0])
```

See also: [ldivide], page 508, [rdivide], page 508.

4.5 Bit Manipulations

Octave provides a number of functions for the manipulation of numeric values on a bit by bit basis. The basic functions to set and obtain the values of individual bits are `bitset` and `bitget`.

x = **bitset** (*a*, *n*) [Function File]
x = **bitset** (*a*, *n*, *v*) [Function File]

Set or reset bit(s) *n* of unsigned integers in *a*. $v = 0$ resets and $v = 1$ sets the bits. The lowest significant bit is: $n = 1$

```
dec2bin (bitset (10, 1))
⇒ 1011
```

See also: [bitand], page 49, [bitor], page 49, [bitxor], page 49, [bitget], page 48, [bitcmp], page 49, [bitshift], page 49, [bitmax], page 48.

X = **bitget** (*a*,*n*) [Function File]

Return the status of bit(s) *n* of unsigned integers in *a* the lowest significant bit is $n = 1$.

```
bitget (100, 8:-1:1)
⇒ 0 1 1 0 0 1 0 0
```

See also: [bitand], page 49, [bitor], page 49, [bitxor], page 49, [bitset], page 48, [bitcmp], page 49, [bitshift], page 49, [bitmax], page 48.

The arguments to all of Octave's bitwise operations can be scalar or arrays, except for `bitcmp`, whose *k* argument must a scalar. In the case where more than one argument is an array, then all arguments must have the same shape, and the bitwise operator is applied to each of the elements of the argument individually. If at least one argument is a scalar and one an array, then the scalar argument is duplicated. Therefore

```
bitget (100, 8:-1:1)
```

is the same as

```
bitget (100 * ones (1, 8), 8:-1:1)
```

It should be noted that all values passed to the bit manipulation functions of Octave are treated as integers. Therefore, even though the example for `bitset` above passes the floating point value `10`, it is treated as the bits `[1, 0, 1, 0]` rather than the bits of the native floating point format representation of `10`.

As the maximum value that can be represented by a number is important for bit manipulation, particularly when forming masks, Octave supplies the function `bitmax`.

bitmax () [Built-in Function]

Return the largest integer that can be represented as a floating point value. On IEEE-754 compatible systems, `bitmax` is `2^53 - 1`.

This is the double precision version of the functions `intmax`, previously discussed.

Octave also includes the basic bitwise 'and', 'or' and 'exclusive or' operators.

bitand (*x*, *y*) [Built-in Function]

Return the bitwise AND of non-negative integers. *x*, *y* must be in the range [0,bitmax]

See also: [bitor], page 49, [bitxor], page 49, [bitset], page 48, [bitget], page 48, [bitcmp], page 49, [bitshift], page 49, [bitmax], page 48.

bitor (*x*, *y*) [Built-in Function]

Return the bitwise OR of non-negative integers. *x*, *y* must be in the range [0,bitmax]

See also: [bitor], page 49, [bitxor], page 49, [bitset], page 48, [bitget], page 48, [bitcmp], page 49, [bitshift], page 49, [bitmax], page 48.

bitxor (*x*, *y*) [Built-in Function]

Return the bitwise XOR of non-negative integers. *x*, *y* must be in the range [0,bitmax]

See also: [bitand], page 49, [bitor], page 49, [bitset], page 48, [bitget], page 48, [bitcmp], page 49, [bitshift], page 49, [bitmax], page 48.

The bitwise 'not' operator is a unary operator that performs a logical negation of each of the bits of the value. For this to make sense, the mask against which the value is negated must be defined. Octave's bitwise 'not' operator is `bitcmp`.

bitcmp (a, *k*) [Function File]

Return the *k*-bit complement of integers in *a*. If *k* is omitted `k = log2 (bitmax) + 1` is assumed.

```
bitcmp(7,4)
⇒ 8
dec2bin(11)
⇒ 1011
dec2bin(bitcmp(11, 6))
⇒ 110100
```

See also: [bitand], page 49, [bitor], page 49, [bitxor], page 49, [bitset], page 48, [bitget], page 48, [bitcmp], page 49, [bitshift], page 49, [bitmax], page 48.

Octave also includes the ability to left-shift and right-shift values bitwise.

bitshift (a, *k*) [Built-in Function]
bitshift (a, *k*, *n*) [Built-in Function]

Return a *k* bit shift of *n*-digit unsigned integers in *a*. A positive *k* leads to a left shift. A negative value to a right shift. If *n* is omitted it defaults to log2(bitmax)+1. *n* must be in the range [1,log2(bitmax)+1] usually [1,33]

```
bitshift (eye (3), 1)
⇒
2 0 0
0 2 0
0 0 2

bitshift (10, [-2, -1, 0, 1, 2])
⇒ 2   5  10  20  40
```

See also: [bitand], page 49, [bitor], page 49, [bitxor], page 49, [bitset], page 48, [bitget], page 48, [bitcmp], page 49, [bitmax], page 48.

Bits that are shifted out of either end of the value are lost. Octave also uses arithmetic shifts, where the sign bit of the value is kept during a right shift. For example

```
bitshift (-10, -1)
⇒ -5
bitshift (int8 (-1), -1)
⇒ -1
```

Note that `bitshift (int8 (-1), -1)` is `-1` since the bit representation of `-1` in the `int8` data type is `[1, 1, 1, 1, 1, 1, 1, 1]`.

4.6 Logical Values

Octave has built-in support for logical values, i.e., variables that are either `true` or `false`. When comparing two variables, the result will be a logical value whose value depends on whether or not the comparison is true.

The basic logical operations are `&`, `|`, and `!`, which correspond to "Logical And", "Logical Or", and "Logical Negation". These operations all follow the usual rules of logic.

It is also possible to use logical values as part of standard numerical calculations. In this case `true` is converted to `1`, and `false` to 0, both represented using double precision floating point numbers. So, the result of `true*22 - false/6` is `22`.

Logical values can also be used to index matrices and cell arrays. When indexing with a logical array the result will be a vector containing the values corresponding to `true` parts of the logical array. The following example illustrates this.

```
data = [ 1, 2; 3, 4 ];
idx = (data <= 2);
data(idx)
     ⇒ ans = [ 1; 2 ]
```

Instead of creating the `idx` array it is possible to replace `data(idx)` with `data( data <= 2 )` in the above code.

Logical values can also be constructed by casting numeric objects to logical values, or by using the `true` or `false` functions.

`logical (`*arg*`)` [Function File]

Convert *arg* to a logical value. For example,

```
logical ([-1, 0, 1])
```

is equivalent to

```
[-1, 0, 1] != 0
```

true (*x*) [Built-in Function]
true (*n*, *m*) [Built-in Function]
true (*n*, *m*, *k*, . . .) [Built-in Function]

Return a matrix or N-dimensional array whose elements are all logical 1. The arguments are handled the same as the arguments for `eye`.

false (*x*) [Built-in Function]
false (*n*, *m*) [Built-in Function]
false (*n*, *m*, *k*, . . .) [Built-in Function]

Return a matrix or N-dimensional array whose elements are all logical 0. The arguments are handled the same as the arguments for `eye`.

4.7 Promotion and Demotion of Data Types

Many operators and functions can work with mixed data types. For example

```
uint8 (1) + 1
    ⇒ 2
```

where the above operator works with an 8-bit integer and a double precision value and returns an 8-bit integer value. Note that the type is demoted to an 8-bit integer, rather than promoted to a double precision value as might be expected. The reason is that if Octave promoted values in expressions like the above with all numerical constants would need to be explicitly cast to the appropriate data type like

```
uint8 (1) + uint8 (1)
    ⇒ 2
```

which becomes difficult for the user to apply uniformly and might allow hard to find bugs to be introduced. The same applies to single precision values where a mixed operation such as

```
single (1) + 1
    ⇒ 2
```

returns a single precision value. The mixed operations that are valid and their returned data types are

Mixed Operation	Result
double OP single	single
double OP integer	integer
double OP char	double
double OP logical	double
single OP integer	integer
single OP char	single
single OP logical	single

The same logic applies to functions with mixed arguments such as

```
min (single (1), 0)
   ⇒ 0
```

where the returned value is single precision.

In the case of mixed type indexed assignments, the type is not changed. For example

```
x = ones (2, 2);
x (1, 1) = single (2)
    ⇒ x = 2   1
          1   1
```

where x remains of the double precision type.

4.8 Predicates for Numeric Objects

Since the type of a variable may change during the execution of a program, it can be necessary to do type checking at run-time. Doing this also allows you to change the behavior of a function depending on the type of the input. As an example, this naive implementation of abs returns the absolute value of the input if it is a real number, and the length of the input if it is a complex number.

```
function a = abs (x)
  if (isreal (x))
    a = sign (x) .* x;
  elseif (iscomplex (x))
    a = sqrt (real(x).^2 + imag(x).^2);
  endif
endfunction
```

The following functions are available for determining the type of a variable.

isnumeric (*x*) [Built-in Function]

Return nonzero if *x* is a numeric object.

isreal (*x*) [Built-in Function]

Return true if *x* is a real-valued numeric object.

isfloat (*x*) [Built-in Function]

Return true if *x* is a floating-point numeric object.

iscomplex (*x*) [Built-in Function]

Return true if *x* is a complex-valued numeric object.

ismatrix (*a*) [Built-in Function]

Return 1 if *a* is a matrix. Otherwise, return 0.

isvector (*a*) [Function File]

Return 1 if *a* is a vector. Otherwise, return 0.

See also: [size], page 36, [rows], page 36, [columns], page 35, [length], page 36, [isscalar], page 52, [ismatrix], page 52.

isscalar (*a*) [Function File]

Return 1 if *a* is a scalar. Otherwise, return 0.

See also: [size], page 36, [rows], page 36, [columns], page 35, [length], page 36, [isscalar], page 52, [ismatrix], page 52.

`issquare (x)` [Function File]

If *x* is a square matrix, then return the dimension of *x*. Otherwise, return 0.

See also: [size], page 36, [rows], page 36, [columns], page 35, [length], page 36, [ismatrix], page 52, [isscalar], page 52, [isvector], page 52.

`issymmetric (x, tol)` [Function File]

If *x* is symmetric within the tolerance specified by *tol*, then return the dimension of *x*. Otherwise, return 0. If *tol* is omitted, use a tolerance equal to the machine precision. Matrix *x* is considered symmetric if `norm (x - x.', inf) / norm (x, inf) < tol`.

See also: [size], page 36, [rows], page 36, [columns], page 35, [length], page 36, [ismatrix], page 52, [isscalar], page 52, [issquare], page 53, [isvector], page 52.

`isdefinite (x, tol)` [Function File]

Return 1 if *x* is symmetric positive definite within the tolerance specified by *tol* or 0 if *x* is symmetric positive semidefinite. Otherwise, return -1. If *tol* is omitted, use a tolerance equal to 100 times the machine precision.

See also: [issymmetric], page 53.

`islogical (x)` [Built-in Function]

Return true if *x* is a logical object.

`isprime (n)` [Function File]

Return true if *n* is a prime number, false otherwise.

Something like the following is much faster if you need to test a lot of small numbers:

```
t = ismember (n, primes (max (n (:))));
```

If max(n) is very large, then you should be using special purpose factorization code.

See also: [primes], page 306, [factor], page 301, [gcd], page 302, [lcm], page 303.

5 Strings

A *string constant* consists of a sequence of characters enclosed in either double-quote or single-quote marks. For example, both of the following expressions

```
"parrot"
'parrot'
```

represent the string whose contents are '`parrot`'. Strings in Octave can be of any length.

Since the single-quote mark is also used for the transpose operator (see Section 8.3 [Arithmetic Ops], page 111) but double-quote marks have no other purpose in Octave, it is best to use double-quote marks to denote strings.

Strings can be concatenated using the notation for defining matrices. For example, the expression

```
[ "foo" , "bar" , "baz" ]
```

produces the string whose contents are '`foobarbaz`'. See Chapter 4 [Numeric Data Types], page 39, for more information about creating matrices.

5.1 Escape Sequences in string constants

In double-quoted strings, the backslash character is used to introduce *escape sequences* that represent other characters. For example, '`\n`' embeds a newline character in a double-quoted string and '`\"`' embeds a double quote character. In single-quoted strings, backslash is not a special character. Here is an example showing the difference:

```
toascii ("\n")
    ⇒ 10
toascii ('\n')
    ⇒ [ 92 110 ]
```

Here is a table of all the escape sequences used in Octave (within double quoted strings). They are the same as those used in the C programming language.

`\\` Represents a literal backslash, '`\`'.

`\"` Represents a literal double-quote character, '`"`'.

`\'` Represents a literal single-quote character, '`'`'.

`\0` Represents the "nul" character, control-@, ASCII code 0.

`\a` Represents the "alert" character, control-g, ASCII code 7.

`\b` Represents a backspace, control-h, ASCII code 8.

`\f` Represents a formfeed, control-l, ASCII code 12.

`\n` Represents a newline, control-j, ASCII code 10.

`\r` Represents a carriage return, control-m, ASCII code 13.

`\t` Represents a horizontal tab, control-i, ASCII code 9.

`\v` Represents a vertical tab, control-k, ASCII code 11.

In a single-quoted string there is only one escape sequence: you may insert a single quote character using two single quote characters in succession. For example,

```
'I can''t escape'
    ⇒ I can't escape
```

5.2 Character Arrays

The string representation used by Octave is an array of characters, so internally the string "dddddddddd" is actually a row vector of length 10 containing the value 100 in all places (100 is the ASCII code of "d"). This lends itself to the obvious generalization to character matrices. Using a matrix of characters, it is possible to represent a collection of same-length strings in one variable. The convention used in Octave is that each row in a character matrix is a separate string, but letting each column represent a string is equally possible.

The easiest way to create a character matrix is to put several strings together into a matrix.

```
collection = [ "String #1"; "String #2" ];
```

This creates a 2-by-9 character matrix.

The function `ischar` can be used to test if an object is a character matrix.

`ischar (a)` [Built-in Function]

Return 1 if *a* is a character array. Otherwise, return 0.

To test if an object is a string (i.e., a character vector and not a character matrix) you can use the `ischar` function in combination with the `isvector` function as in the following example:

```
ischar(collection)
     ⇒ ans = 1

ischar(collection) && isvector(collection)
     ⇒ ans = 0

ischar("my string") && isvector("my string")
     ⇒ ans = 1
```

One relevant question is, what happens when a character matrix is created from strings of different length. The answer is that Octave puts blank characters at the end of strings shorter than the longest string. It is possible to use a different character than the blank character using the `string_fill_char` function.

val = `string_fill_char ()` [Built-in Function]
old_val = `string_fill_char (`*new_val*`)` [Built-in Function]

Query or set the internal variable used to pad all rows of a character matrix to the same length. It must be a single character. The default value is " " (a single space). For example,

```
string_fill_char ("X");
[ "these"; "are"; "strings" ]
     ⇒ "theseXX"
       "areXXXX"
       "strings"
```

This shows a problem with character matrices. It simply isn't possible to represent strings of different lengths. The solution is to use a cell array of strings, which is described in Section 6.2.4 [Cell Arrays of Strings], page 90.

5.3 Creating Strings

The easiest way to create a string is, as illustrated in the introduction, to enclose a text in double-quotes or single-quotes. It is however possible to create a string without actually writing a text. The function `blanks` creates a string of a given length consisting only of blank characters (ASCII code 32).

`blanks (n)` [Function File]

Return a string of *n* blanks, for example:

```
blanks(10);
whos ans;
     ⇒
      Attr Name        Size                     Bytes  Class
      ==== ====        ====                     =====  =====
           ans         1x10                        10  char
```

See also: [repmat], page 285.

5.3.1 Concatenating Strings

It has been shown above that strings can be concatenated using matrix notation (see Chapter 5 [Strings], page 55, Section 5.2 [Character Arrays], page 56). Apart from that, there are several functions to concatenate string objects: `char`, `strvcat`, `strcat` and `cstrcat`. In addition, the general purpose concatenation functions can be used: see [cat], page 277, [horzcat], page 277 and [vertcat], page 277.

- All string concatenation functions except `cstrcat` convert numerical input into character data by taking the corresponding ASCII character for each element, as in the following example:

```
char([98, 97, 110, 97, 110, 97])
     ⇒ ans =
       banana
```

- `char` and `strvcat` concatenate vertically, while `strcat` and `cstrcat` concatenate horizontally. For example:

```
char("an apple", "two pears")
     ⇒ ans =
       an apple
       two pears

strcat("oc", "tave", " is", " good", " for you")
     ⇒ ans =
       octave is good for you
```

- `char` generates an empty row in the output for each empty string in the input. `strvcat`, on the other hand, eliminates empty strings.

```
char("orange", "green", "", "red")
     ⇒ ans =
       orange
       green

       red

strvcat("orange", "green", "", "red")
     ⇒ ans =
       orange
       green
       red
```

- All string concatenation functions except `cstrcat` also accept cell array data (see Section 6.2 [Cell Arrays], page 85). `char` and `strvcat` convert cell arrays into character arrays, while `strcat` concatenates within the cells of the cell arrays:

```
char({"red", "green", "", "blue"})
     ⇒ ans =
       red
       green

       blue

strcat({"abc"; "ghi"}, {"def"; "jkl"})
     ⇒ ans =
       {
         [1,1] = abcdef
         [2,1] = ghijkl
       }
```

- `strcat` removes trailing white space in the arguments (except within cell arrays), while `cstrcat` leaves white space untouched. Both kinds of behavior can be useful as can be seen in the examples:

```
strcat(["dir1";"directory2"], ["/";"/"], ["file1";"file2"])
     ⇒ ans =
       dir1/file1
       directory2/file2

cstrcat(["thirteen apples"; "a banana"], [" 5$";" 1$"])
     ⇒ ans =
       thirteen apples 5$
       a banana        1$
```

  Note that in the above example for `cstrcat`, the white space originates from the internal representation of the strings in a string array (see Section 5.2 [Character Arrays], page 56).

`char` (*x*) [Built-in Function]
`char` (*x*, . . .) [Built-in Function]

`char (s1, s2, ...)` [Built-in Function]
`char (cell_array)` [Built-in Function]

Create a string array from one or more numeric matrices, character matrices, or cell arrays. Arguments are concatenated vertically. The returned values are padded with blanks as needed to make each row of the string array have the same length. Empty input strings are significant and will concatenated in the output.

For numerical input, each element is converted to the corresponding ASCII character. A range error results if an input is outside the ASCII range (0-255).

For cell arrays, each element is concatenated separately. Cell arrays converted through `char` can mostly be converted back with `cellstr`. For example,

```
char ([97, 98, 99], "", {"98", "99", 100}, "str1", ["ha", "lf"])
     ⇒ ["abc    "
        "       "
        "98     "
        "99     "
        "d      "
        "str1   "
        "half   "]
```

See also: [strvcat], page 59, [cellstr], page 90.

`strvcat (x)` [Built-in Function]
`strvcat (x, ...)` [Built-in Function]
`strvcat (s1, s2, ...)` [Built-in Function]
`strvcat (cell_array)` [Built-in Function]

Create a character array from one or more numeric matrices, character matrices, or cell arrays. Arguments are concatenated vertically. The returned values are padded with blanks as needed to make each row of the string array have the same length. Unlike `char`, empty strings are removed and will not appear in the output.

For numerical input, each element is converted to the corresponding ASCII character. A range error results if an input is outside the ASCII range (0-255).

For cell arrays, each element is concatenated separately. Cell arrays converted through `strvcat` can mostly be converted back with `cellstr`. For example,

```
strvcat ([97, 98, 99], "", {"98", "99", 100}, "str1", ["ha", "lf"])
     ⇒ ["abc    "
        "98     "
        "99     "
        "d      "
        "str1   "
        "half   "]
```

See also: [char], page 58, [strcat], page 59, [cstrcat], page 60.

`strcat (s1, s2, ...)` [Function File]

Return a string containing all the arguments concatenated horizontally. If the arguments are cells strings, `strcat` returns a cell string with the individual cells concatenated. For numerical input, each element is converted to the corresponding ASCII character. Trailing white space is eliminated. For example,

```
s = [ "ab"; "cde" ];
strcat (s, s, s)
     ⇒ ans =
        "ab ab ab "
        "cdecdecde"
s = { "ab"; "cde" };
strcat (s, s, s)
     ⇒ ans =
        {
          [1,1] = ababab
          [2,1] = cdecdecde
        }
```

See also: [cstrcat], page 60, [char], page 58, [strvcat], page 59.

`cstrcat` (*s1*, *s2*, . . .) [Function File]

Return a string containing all the arguments concatenated horizontally. Trailing white space is preserved. For example,

```
cstrcat ("ab   ", "cd")
     ⇒ "ab   cd"
s = [ "ab"; "cde" ];
cstrcat (s, s, s)
     ⇒ ans =
        "ab ab ab "
        "cdecdecde"
```

See also: [strcat], page 59, [char], page 58, [strvcat], page 59.

5.3.2 Conversion of Numerical Data to Strings

Apart from the string concatenation functions (see Section 5.3.1 [Concatenating Strings], page 57) which cast numerical data to the corresponding ASCII characters, there are several functions that format numerical data as strings. `mat2str` and `num2str` convert real or complex matrices, while `int2str` converts integer matrices. `int2str` takes the real part of complex values and round fractional values to integer. A more flexible way to format numerical data as strings is the `sprintf` function (see Section 14.2.4 [Formatted Output], page 190, [doc-sprintf], page 191).

s = `mat2str` (*x*, *n*) [Function File]
s = `mat2str` (. . . , *'class'*) [Function File]

Format real/complex numerical matrices as strings. This function returns values that are suitable for the use of the `eval` function.

The precision of the values is given by *n*. If *n* is a scalar then both real and imaginary parts of the matrix are printed to the same precision. Otherwise `n (1)` defines the precision of the real part and `n (2)` defines the precision of the imaginary part. The default for *n* is 17.

If the argument 'class' is given, then the class of *x* is included in the string in such a way that the eval will result in the construction of a matrix of the same class.

```
mat2str ([ -1/3 + i/7; 1/3 - i/7 ], [4 2])
     ⇒ "[-0.3333+0.14i;0.3333-0.14i]"

mat2str ([ -1/3 +i/7; 1/3 -i/7 ], [4 2])
     ⇒ "[-0.3333+0i,0+0.14i;0.3333+0i,-0-0.14i]"

mat2str (int16([1 -1]), 'class')
     ⇒ "int16([1,-1])"
```

See also: [sprintf], page 191, [num2str], page 61, [int2str], page 61.

`num2str` (*x*) [Function File]
`num2str` (*x*, *precision*) [Function File]
`num2str` (*x*, *format*) [Function File]

Convert a number (or array) to a string (or a character array). The optional second argument may either give the number of significant digits (*precision*) to be used in the output or a format template string (*format*) as in `sprintf` (see Section 14.2.4 [Formatted Output], page 190). `num2str` can also handle complex numbers. For example:

```
num2str (123.456)
     ⇒ "123.46"

num2str (123.456, 4)
     ⇒ "123.5"

s = num2str ([1, 1.34; 3, 3.56], "%5.1f")
     ⇒ s =
        1.0  1.3
        3.0  3.6
whos s
     ⇒
      Attr Name        Size                     Bytes  Class
      ==== ====        ====                     =====  =====
           s           2x8                         16  char

num2str (1.234 + 27.3i)
     ⇒ "1.234+27.3i"
```

The `num2str` function is not very flexible. For better control over the results, use `sprintf` (see Section 14.2.4 [Formatted Output], page 190). Note that for complex *x*, the format string may only contain one output conversion specification and nothing else. Otherwise, you will get unpredictable results.

See also: [sprintf], page 191, [int2str], page 61, [mat2str], page 60.

`int2str` (*n*) [Function File]

Convert an integer (or array of integers) to a string (or a character array).

```
int2str (123)
     ⇒ "123"

s = int2str ([1, 2, 3; 4, 5, 6])
     ⇒ s =
        1  2  3
        4  5  6

whos s
     ⇒ s =
       Attr Name        Size                     Bytes  Class
       ==== ====        ====                     =====  =====
            s           2x7                         14  char
```

This function is not very flexible. For better control over the results, use `sprintf` (see Section 14.2.4 [Formatted Output], page 190).

See also: [sprintf], page 191, [num2str], page 61, [mat2str], page 60.

5.4 Comparing Strings

Since a string is a character array, comparisons between strings work element by element as the following example shows:

```
GNU = "GNU's Not UNIX";
spaces = (GNU == " ")
     ⇒ spaces =
       0   0   0   0   0   1   0   0   0   1   0   0   0   0
```

To determine if two strings are identical it is necessary to use the `strcmp` function. It compares complete strings and is case sensitive. `strncmp` compares only the first `N` characters (with `N` given as a parameter). `strcmpi` and `strncmpi` are the corresponding functions for case-insensitive comparison.

`strcmp` (*s1*, *s2*) [Built-in Function]

Return 1 if the character strings *s1* and *s2* are the same, and 0 otherwise.

If either *s1* or *s2* is a cell array of strings, then an array of the same size is returned, containing the values described above for every member of the cell array. The other argument may also be a cell array of strings (of the same size or with only one element), char matrix or character string.

Caution: For compatibility with MATLAB, Octave's strcmp function returns 1 if the character strings are equal, and 0 otherwise. This is just the opposite of the corresponding C library function.

See also: [strcmpi], page 63, [strncmp], page 62, [strncmpi], page 63.

`strncmp` (*s1*, *s2*, *n*) [Built-in Function]

Return 1 if the first *n* characters of strings *s1* and *s2* are the same, and 0 otherwise.

```
strncmp ("abce", "abcd", 3)
     ⇒ 1
```

If either *s1* or *s2* is a cell array of strings, then an array of the same size is returned, containing the values described above for every member of the cell array. The other argument may also be a cell array of strings (of the same size or with only one element), char matrix or character string.

```
strncmp ("abce", {"abcd", "bca", "abc"}, 3)
     ⇒ [1, 0, 1]
```

Caution: For compatibility with MATLAB, Octave's strncmp function returns 1 if the character strings are equal, and 0 otherwise. This is just the opposite of the corresponding C library function.

See also: [strncmpi], page 63, [strcmp], page 62, [strcmpi], page 63.

`strcmpi (`*`s1`*`, `*`s2`*`)` [Function File]

Ignoring case, return 1 if the character strings (or character arrays) *s1* and *s2* are the same, and 0 otherwise.

If either *s1* or *s2* is a cell array of strings, then an array of the same size is returned, containing the values described above for every member of the cell array. The other argument may also be a cell array of strings (of the same size or with only one element), char matrix or character string.

Caution: For compatibility with MATLAB, Octave's strcmpi function returns 1 if the character strings are equal, and 0 otherwise. This is just the opposite of the corresponding C library function.

See also: [strcmp], page 62, [strncmp], page 62, [strncmpi], page 63.

`strncmpi (`*`s1`*`, `*`s2`*`, `*`n`*`)` [Function File]

Ignoring case, return 1 if the first *n* characters of character strings (or character arrays) *s1* and *s2* are the same, and 0 otherwise.

If either *s1* or *s2* is a cell array of strings, then an array of the same size is returned, containing the values described above for every member of the cell array. The other argument may also be a cell array of strings (of the same size or with only one element), char matrix or character string.

Caution: For compatibility with MATLAB, Octave's strncmpi function returns 1 if the character strings are equal, and 0 otherwise. This is just the opposite of the corresponding C library function.

See also: [strcmp], page 62, [strcmpi], page 63, [strncmp], page 62.

`validstr`` = validatestring (`*`str`*`, `*`strarray`*`)` [Function File]
`validstr`` = validatestring (`*`str`*`, `*`strarray`*`, `*`funcname`*`)` [Function File]
`validstr`` = validatestring (`*`str`*`, `*`strarray`*`, `*`funcname`*`, `*`varname`*`)` [Function File]
`validstr`` = validatestring (..., `*`position`*`)` [Function File]

Verify that *str* is a string or substring of an element of *strarray*.

str is a character string to be tested, and *strarray* is a cellstr of valid values. *validstr* will be the validated form of *str* where validation is defined as *str* being a member or substring of *validstr*. If *str* is a substring of *validstr* and there are multiple matches,

the shortest match will be returned if all matches are substrings of each other, and an error will be raised if the matches are not substrings of each other.

All comparisons are case insensitive.

See also: [strcmp], page 62, [strcmpi], page 63.

5.5 Manipulating Strings

Octave supports a wide range of functions for manipulating strings. Since a string is just a matrix, simple manipulations can be accomplished using standard operators. The following example shows how to replace all blank characters with underscores.

```
quote = ...
  "First things first, but not necessarily in that order";
quote( quote == " " ) = "_"
⇒ quote =
    First_things_first,_but_not_necessarily_in_that_order
```

For more complex manipulations, such as searching, replacing, and general regular expressions, the following functions come with Octave.

`deblank` (*s*) [Function File]

Remove trailing blanks and nulls from *s*. If *s* is a matrix, *deblank* trims each row to the length of longest string. If *s* is a cell array, operate recursively on each element of the cell array.

`strtrim` (*s*) [Function File]

Remove leading and trailing blanks and nulls from *s*. If *s* is a matrix, *strtrim* trims each row to the length of longest string. If *s* is a cell array, operate recursively on each element of the cell array. For example:

```
strtrim ("    abc  ")
     ⇒ "abc"

strtrim ([" abc   "; "   def   "])
     ⇒ ["abc  "; "  def"]
```

`strtrunc` (*s*, *n*) [Function File]

Truncate the character string *s* to length *n*. If *s* is a char matrix, then the number of columns is adjusted.

If *s* is a cell array of strings, then the operation is performed on its members and the new cell array is returned.

`findstr` (*s*, *t*, *overlap*) [Function File]

Return the vector of all positions in the longer of the two strings *s* and *t* where an occurrence of the shorter of the two starts. If the optional argument *overlap* is nonzero, the returned vector can include overlapping positions (this is the default). For example,

```
findstr ("ababab", "a")
     ⇒ [1, 3, 5]
findstr ("abababa", "aba", 0)
     ⇒ [1, 5]
```

See also: [strfind], page 65, [strmatch], page 66, [strcmp], page 62, [strncmp], page 62, [strcmpi], page 63, [strncmpi], page 63, [find], page 274.

idx = `strchr` (*str*, *chars*) [Function File]
idx = `strchr` (*str*, *chars*, *n*) [Function File]
idx = `strchr` (*str*, *chars*, *n*, *direction*) [Function File]

Search for the string *str* for occurrences of characters from the set *chars*. The return value, as well as the *n* and *direction* arguments behave identically as in `find`.

This will be faster than using regexp in most cases.

See also: [find], page 274.

`index` (*s*, *t*) [Function File]
`index` (*s*, *t*, *direction*) [Function File]

Return the position of the first occurrence of the string *t* in the string *s*, or 0 if no occurrence is found. For example,

```
index ("Teststring", "t")
     ⇒ 4
```

If *direction* is '`"first"`', return the first element found. If *direction* is '`"last"`', return the last element found. The `rindex` function is equivalent to `index` with *direction* set to '`"last"`'.

Caution: This function does not work for arrays of character strings.

See also: [find], page 274, [rindex], page 65.

`rindex` (*s*, *t*) [Function File]

Return the position of the last occurrence of the character string *t* in the character string *s*, or 0 if no occurrence is found. For example,

```
rindex ("Teststring", "t")
     ⇒ 6
```

Caution: This function does not work for arrays of character strings.

See also: [find], page 274, [index], page 65.

idx = `strfind` (*str*, *pattern*) [Function File]
idx = `strfind` (*cellstr*, *pattern*) [Function File]

Search for *pattern* in the string *str* and return the starting index of every such occurrence in the vector *idx*. If there is no such occurrence, or if *pattern* is longer than *str*, then *idx* is the empty array `[]`.

If the cell array of strings *cellstr* is specified instead of the string *str*, then *idx* is a cell array of vectors, as specified above. Examples:

```
strfind ("abababa", "aba")
     ⇒ [1, 3, 5]

strfind ({"abababa", "bebebe", "ab"}, "aba")
     ⇒ ans =
        {
          [1,1] =

             1   3   5

          [1,2] = [](1x0)
          [1,3] = [](1x0)
        }
```

See also: [findstr], page 64, [strmatch], page 66, [strcmp], page 62, [strncmp], page 62, [strcmpi], page 63, [strncmpi], page 63, [find], page 274.

`strmatch` (*s*, *a*, "*exact*") [Function File]

Return indices of entries of *a* that match the string *s*. The second argument *a* may be a string matrix or a cell array of strings. If the third argument `"exact"` is not given, then *s* only needs to match *a* up to the length of *s*. Nul characters match blanks. Results are returned as a column vector. For example:

```
strmatch ("apple", "apple juice")
     ⇒ 1

strmatch ("apple", ["apple pie"; "apple juice"; "an apple"])
     ⇒ [1; 2]

strmatch ("apple", {"apple pie"; "apple juice"; "tomato"})
     ⇒ [1; 2]
```

See also: [strfind], page 65, [findstr], page 64, [strcmp], page 62, [strncmp], page 62, [strcmpi], page 63, [strncmpi], page 63, [find], page 274.

[*tok*, *rem*] = `strtok` (*str*, *delim*) [Function File]

Find all characters up to but not including the first character which is in the string delim. If *rem* is requested, it contains the remainder of the string, starting at the first delimiter. Leading delimiters are ignored. If *delim* is not specified, space is assumed. For example:

```
strtok ("this is the life")
     ⇒ "this"

[tok, rem] = strtok ("14*27+31", "+-*/")
     ⇒
        tok = 14
        rem = *27+31
```

See also: [index], page 65, [strsplit], page 67.

`[s] = strsplit (p, sep, strip_empty)` [Function File]

Split a single string using one or more delimiters and return a cell array of strings. Consecutive delimiters and delimiters at boundaries result in empty strings, unless *strip_empty* is true. The default value of *strip_empty* is false.

See also: [strtok], page 66.

`strrep (s, x, y)` [Function File]

Replace all occurrences of the substring *x* of the string *s* with the string *y* and return the result. For example,

```
strrep ("This is a test string", "is", "&%$")
     ⇒ "Th&%$ &%$ a test string"
```

See also: [regexprep], page 69, [strfind], page 65, [findstr], page 64.

`substr (s, offset, len)` [Function File]

Return the substring of *s* which starts at character number *offset* and is *len* characters long.

If *offset* is negative, extraction starts that far from the end of the string. If *len* is omitted, the substring extends to the end of S.

For example,

```
substr ("This is a test string", 6, 9)
     ⇒ "is a test"
```

This function is patterned after AWK. You can get the same result by `s(offset : (offset + len - 1))`.

`[s, e, te, m, t, nm] = regexp (str, pat)` [Loadable Function]
`[...] = regexp (str, pat, opts, ...)` [Loadable Function]

Regular expression string matching. Matches *pat* in *str* and returns the position and matching substrings or empty values if there are none.

The matched pattern *pat* can include any of the standard regex operators, including:

`.` Match any character

`* + ? {}` Repetition operators, representing

- `*` Match zero or more times
- `+` Match one or more times
- `?` Match zero or one times
- `{}` Match range operator, which is of the form `{n}` to match exactly *n* times, `{m,}` to match *m* or more times, `{m,n}` to match between *m* and *n* times.

`[...] [^...]`
List operators, where for example `[ab]c` matches `ac` and `bc`

`()` Grouping operator

`|` Alternation operator. Match one of a choice of regular expressions. The alternatives must be delimited by the grouping operator `()` above

`^ $` Anchoring operator. `^` matches the start of the string *str* and `$` the end

In addition the following escaped characters have special meaning. It should be noted that it is recommended to quote *pat* in single quotes rather than double quotes, to avoid the escape sequences being interpreted by Octave before being passed to `regexp`.

`\b` Match a word boundary

`\B` Match within a word

`\w` Matches any word character

`\W` Matches any non word character

`\<` Matches the beginning of a word

`\>` Matches the end of a word

`\s` Matches any whitespace character

`\S` Matches any non whitespace character

`\d` Matches any digit

`\D` Matches any non-digit

The outputs of `regexp` by default are in the order as given below

s The start indices of each of the matching substrings

e The end indices of each matching substring

te The extents of each of the matched token surrounded by `(...)` in *pat*.

m A cell array of the text of each match.

t A cell array of the text of each token matched.

nm A structure containing the text of each matched named token, with the name being used as the fieldname. A named token is denoted as `(?<name>...)`

Particular output arguments or the order of the output arguments can be selected by additional *opts* arguments. These are strings and the correspondence between the output arguments and the optional argument are

'start'	*s*
'end'	*e*
'tokenExtents'	*te*
'match'	*m*
'tokens'	*t*
'names'	*nm*

A further optional argument is 'once', that limits the number of returned matches to the first match. Additional arguments are

matchcase Make the matching case sensitive.

ignorecase Make the matching case insensitive.

stringanchors
: Match the anchor characters at the beginning and end of the string.

lineanchors
: Match the anchor characters at the beginning and end of the line.

dotall
: The character . matches the newline character.

dotexceptnewline
: The character . matches all but the newline character.

freespacing
: The pattern can include arbitrary whitespace and comments starting with `#`.

literalspacing
: The pattern is taken literally.

See also: [regexpi], page 69, [regexprep], page 69.

`[s, e, te, m, t, nm] = regexpi (str, pat)` [Loadable Function]
`[...] = regexpi (str, pat, opts, ...)` [Loadable Function]

Case insensitive regular expression string matching. Matches *pat* in *str* and returns the position and matching substrings or empty values if there are none. See [regexp], page 67, for more details

`string = regexprep (string, pat, repstr, options)` [Loadable Function]

Replace matches of *pat* in *string* with *repstr*.

The replacement can contain `$i`, which substitutes for the ith set of parentheses in the match string. E.g.,

```
regexprep("Bill Dunn",'(\w+) (\w+)','$2, $1')
```

returns "Dunn, Bill"

options may be zero or more of

'`once`'
: Replace only the first occurrence of *pat* in the result.

'`warnings`'
: This option is present for compatibility but is ignored.

'`ignorecase or matchcase`'
: Ignore case for the pattern matching (see `regexpi`). Alternatively, use (?i) or (?-i) in the pattern.

'`lineanchors and stringanchors`'
: Whether characters ^ and $ match the beginning and ending of lines. Alternatively, use (?m) or (?-m) in the pattern.

'`dotexceptnewline and dotall`'
: Whether . matches newlines in the string. Alternatively, use (?s) or (?-s) in the pattern.

'`freespacing` or `literalspacing`'
: Whether whitespace and # comments can be used to make the regular expression more readable. Alternatively, use (?x) or (?-x) in the pattern.

See also: [regexp], page 67, [regexpi], page 69, [strrep], page 67.

`regexptranslate` (*op*, *s*) [Function File]

Translate a string for use in a regular expression. This might include either wildcard replacement or special character escaping. The behavior can be controlled by the *op* that can have the values

"wildcard"
: The wildcard characters `.`, `*` and `?` are replaced with wildcards that are appropriate for a regular expression. For example:

```
regexptranslate ("wildcard", "*.m")
     ⇒ ".*\.m"
```

"escape"
: The characters `$.?[]`, that have special meaning for regular expressions are escaped so that they are treated literally. For example:

```
regexptranslate ("escape", "12.5")
     ⇒ "12\.5"
```

See also: [regexp], page 67, [regexpi], page 69, [regexprep], page 69.

5.6 String Conversions

Octave supports various kinds of conversions between strings and numbers. As an example, it is possible to convert a string containing a hexadecimal number to a floating point number.

```
hex2dec ("FF")
     ⇒ ans = 255
```

`bin2dec` (*s*) [Function File]

Return the decimal number corresponding to the binary number stored in the string *s*. For example,

```
bin2dec ("1110")
     ⇒ 14
```

If *s* is a string matrix, returns a column vector of converted numbers, one per row of *s*. Invalid rows evaluate to NaN.

See also: [dec2hex], page 71, [base2dec], page 72, [dec2base], page 71, [hex2dec], page 71, [dec2bin], page 70.

`dec2bin` (*n*, *len*) [Function File]

Return a binary number corresponding to the non-negative decimal number *n*, as a string of ones and zeros. For example,

```
dec2bin (14)
     ⇒ "1110"
```

If *n* is a vector, returns a string matrix, one row per value, padded with leading zeros to the width of the largest value.

The optional second argument, *len*, specifies the minimum number of digits in the result.

See also: [bin2dec], page 70, [dec2base], page 71, [base2dec], page 72, [hex2dec], page 71, [dec2hex], page 71.

`dec2hex (n, len)` [Function File]

Return the hexadecimal string corresponding to the non-negative integer *n*. For example,

```
dec2hex (2748)
     ⇒ "ABC"
```

If *n* is a vector, returns a string matrix, one row per value, padded with leading zeros to the width of the largest value.

The optional second argument, *len*, specifies the minimum number of digits in the result.

See also: [hex2dec], page 71, [dec2base], page 71, [base2dec], page 72, [bin2dec], page 70, [dec2bin], page 70.

`hex2dec (s)` [Function File]

Return the integer corresponding to the hexadecimal number stored in the string *s*. For example,

```
hex2dec ("12B")
     ⇒ 299
hex2dec ("12b")
     ⇒ 299
```

If *s* is a string matrix, returns a column vector of converted numbers, one per row of *s*. Invalid rows evaluate to NaN.

See also: [dec2hex], page 71, [base2dec], page 72, [dec2base], page 71, [bin2dec], page 70, [dec2bin], page 70.

`dec2base (n, b, len)` [Function File]

Return a string of symbols in base *b* corresponding to the non-negative integer *n*.

```
dec2base (123, 3)
     ⇒ "11120"
```

If *n* is a vector, return a string matrix with one row per value, padded with leading zeros to the width of the largest value.

If *b* is a string then the characters of *b* are used as the symbols for the digits of *n*. Space (' ') may not be used as a symbol.

```
dec2base (123, "aei")
     ⇒ "eeeia"
```

The optional third argument, *len*, specifies the minimum number of digits in the result.

See also: [base2dec], page 72, [dec2bin], page 70, [bin2dec], page 70, [hex2dec], page 71, [dec2hex], page 71.

`base2dec (s, b)` [Function File]

Convert *s* from a string of digits of base *b* into an integer.

```
base2dec ("11120", 3)
     ⇒ 123
```

If *s* is a matrix, returns a column vector with one value per row of *s*. If a row contains invalid symbols then the corresponding value will be NaN. Rows are right-justified before converting so that trailing spaces are ignored.

If *b* is a string, the characters of *b* are used as the symbols for the digits of *s*. Space (' ') may not be used as a symbol.

```
base2dec ("yyyzx", "xyz")
     ⇒ 123
```

See also: [dec2base], page 71, [dec2bin], page 70, [bin2dec], page 70, [hex2dec], page 71, [dec2hex], page 71.

s = `num2hex (n)` [Loadable Function]

Typecast a double precision number or vector to a 16 character hexadecimal string of the IEEE 754 representation of the number. For example

```
num2hex ([-1, 1, e, Inf, NaN, NA]);
⇒ "bff0000000000000
    3ff0000000000000
    4005bf0a8b145769
    7ff0000000000000
    fff8000000000000
    7ff00000000007a2"
```

See also: [hex2num], page 72, [hex2dec], page 71, [dec2hex], page 71.

n = `hex2num (s)` [Loadable Function]

Typecast the 16 character hexadecimal character matrix to an IEEE 754 double precision number. If fewer than 16 characters are given the strings are right padded with '0' characters.

Given a string matrix, `hex2num` treats each row as a separate number.

```
hex2num (["4005bf0a8b145769";"4024000000000000"])
⇒ [2.7183; 10.000]
```

See also: [num2hex], page 72, [hex2dec], page 71, [dec2hex], page 71.

`[num, status, strarray] = str2double (str, cdelim, rdelim, ddelim)` [Function File]

Convert strings into numeric values.

`str2double` can replace `str2num`, but avoids the use of `eval` on unknown data.

str can be the form '`[+-]d[.]dd[[eE][+-]ddd]`' in which '`d`' can be any of digit from 0 to 9, and '`[]`' indicate optional elements.

num is the corresponding numeric value. If the conversion fails, status is -1 and *num* is NaN.

status is 0 if the conversion was successful and -1 otherwise.

strarray is a cell array of strings.

Elements which are not defined or not valid return NaN and the *status* becomes -1.

If *str* is a character array or a cell array of strings, then *num* and *status* return matrices of appropriate size.

str can also contain multiple elements separated by row and column delimiters (*cdelim* and *rdelim*).

The parameters *cdelim*, *rdelim*, and *ddelim* are optional column, row, and decimal delimiters.

The default row-delimiters are newline, carriage return and semicolon (ASCII 10, 13 and 59). The default column-delimiters are tab, space and comma (ASCII 9, 32, and 44). The default decimal delimiter is '`.`' (ASCII 46).

cdelim, *rdelim*, and *ddelim* must contain only nul, newline, carriage return, semicolon, colon, slash, tab, space, comma, or '`()[]{}`' (ASCII 0, 9, 10, 11, 12, 13, 14, 32, 33, 34, 40, 41, 44, 47, 58, 59, 91, 93, 123, 124, 125).

Examples:

```
str2double ("-.1e-5")
⇒ -1.0000e-006

str2double (".314e1, 44.44e-1, .7; -1e+1")
⇒
   3.1400    4.4440    0.7000
 -10.0000       NaN       NaN

line = "200, 300, NaN, -inf, yes, no, 999, maybe, NaN";
[x, status] = str2double (line)
⇒ x =
    200   300   NaN  -Inf   NaN   NaN   999   NaN   NaN
⇒ status =
      0     0     0     0    -1    -1     0    -1     0
```

See also: [str2num], page 73.

`strjust` (*s*, ["*left*"|"*right*"|"*center*"]) [Function File]

Shift the non-blank text of *s* to the left, right or center of the string. If *s* is a string array, justify each string in the array. Null characters are replaced by blanks. If no justification is specified, then all rows are right-justified. For example:

```
strjust (["a"; "ab"; "abc"; "abcd"])
     ⇒ ans =
           a
          ab
         abc
        abcd
```

`str2num` (*s*) [Function File]

Convert the string (or character array) *s* to a number (or an array). Examples:

```
str2num("3.141596")
     ⇒ 3.141596

str2num(["1, 2, 3"; "4, 5, 6"]);
     ⇒ ans =
        1  2  3
        4  5  6
```

Caution: As `str2num` uses the `eval` function to do the conversion, `str2num` will execute any code contained in the string *s*. Use `str2double` instead if you want to avoid the use of `eval`.

See also: [str2double], page 72, [eval], page 121.

`toascii (s)` [Mapping Function]

Return ASCII representation of *s* in a matrix. For example,

```
toascii ("ASCII")
     ⇒ [ 65, 83, 67, 73, 73 ]
```

See also: [char], page 58.

`tolower (s)` [Mapping Function]
`lower (s)` [Mapping Function]

Return a copy of the string or cell string *s*, with each upper-case character replaced by the corresponding lower-case one; non-alphabetic characters are left unchanged. For example,

```
tolower ("MiXeD cAsE 123")
     ⇒ "mixed case 123"
```

See also: [toupper], page 74.

`toupper (s)` [Built-in Function]
`upper (s)` [Built-in Function]

Return a copy of the string or cell string *s*, with each lower-case character replaced by the corresponding upper-case one; non-alphabetic characters are left unchanged. For example,

```
toupper ("MiXeD cAsE 123")
     ⇒ "MIXED CASE 123"
```

See also: [tolower], page 74.

`do_string_escapes (string)` [Built-in Function]

Convert special characters in *string* to their escaped forms.

`undo_string_escapes (s)` [Built-in Function]

Converts special characters in strings back to their escaped forms. For example, the expression

```
bell = "\a";
```

assigns the value of the alert character (control-g, ASCII code 7) to the string variable `bell`. If this string is printed, the system will ring the terminal bell (if it is possible).

This is normally the desired outcome. However, sometimes it is useful to be able to print the original representation of the string, with the special characters replaced by their escape sequences. For example,

```
octave:13> undo_string_escapes (bell)
ans = \a
```

replaces the unprintable alert character with its printable representation.

5.7 Character Class Functions

Octave also provides the following character class test functions patterned after the functions in the standard C library. They all operate on string arrays and return matrices of zeros and ones. Elements that are nonzero indicate that the condition was true for the corresponding character in the string array. For example,

```
isalpha ("!Q@WERT^Y&")
     ⇒ [ 0, 1, 0, 1, 1, 1, 1, 0, 1, 0 ]
```

isalnum (*s*) [Mapping Function]
Return 1 for characters that are letters or digits (**isalpha (*s*)** or **isdigit (*s*)** is true).

isalpha (*s*) [Mapping Function]
isletter (*s*) [Mapping Function]
Return true for characters that are letters (**isupper (*s*)** or **islower (*s*)** is true).

isascii (*s*) [Mapping Function]
Return 1 for characters that are ASCII (in the range 0 to 127 decimal).

iscntrl (*s*) [Mapping Function]
Return 1 for control characters.

isdigit (*s*) [Mapping Function]
Return 1 for characters that are decimal digits.

isgraph (*s*) [Mapping Function]
Return 1 for printable characters (but not the space character).

isletter (*s*) [Function File]
Returns true if *s* is a letter, false otherwise.

See also: [isalpha], page 75.

islower (*s*) [Mapping Function]
Return 1 for characters that are lower case letters.

isprint (*s*) [Mapping Function]
Return 1 for printable characters (including the space character).

ispunct (*s*) [Mapping Function]
Return 1 for punctuation characters.

isspace (*s*) [Mapping Function]

Return 1 for whitespace characters (space, formfeed, newline, carriage return, tab, and vertical tab).

isupper (*s*) [Mapping Function]

Return 1 for upper case letters.

isxdigit (*s*) [Mapping Function]

Return 1 for characters that are hexadecimal digits.

isstrprop (*str*, *pred*) [Function File]

Test character string properties. For example,

```
isstrprop ("abc123", "alpha")
⇒ [1, 1, 1, 0, 0, 0]
```

If *str* is a cell array, `isstrpop` is applied recursively to each element of the cell array.

Numeric arrays are converted to character strings.

The second argument *pred* may be one of

`"alpha"` True for characters that are alphabetic

`"alnum"`
`"alphanum"`
True for characters that are alphabetic or digits.

`"ascii"` True for characters that are in the range of ASCII encoding.

`"cntrl"` True for control characters.

`"digit"` True for decimal digits.

`"graph"`
`"graphic"`
True for printing characters except space.

`"lower"` True for lower-case letters.

`"print"` True for printing characters including space.

`"punct"` True for printing characters except space or letter or digit.

`"space"`
`"wspace"` True for whitespace characters (space, formfeed, newline, carriage return, tab, vertical tab).

`"upper"` True for upper-case letters.

`"xdigit"` True for hexadecimal digits.

See also: [isalnum], page 75, [isalpha], page 75, [isascii], page 75, [iscntrl], page 75, [isdigit], page 75, [isgraph], page 75, [islower], page 75, [isprint], page 75, [ispunct], page 75, [isspace], page 76, [isupper], page 76, [isxdigit], page 76.

6 Data Containers

Octave includes support for two different mechanisms to contain arbitrary data types in the same variable. Structures, which are C-like, and are indexed with named fields, and cell arrays, where each element of the array can have a different data type and or shape. Multiple input arguments and return values of functions are organized as another data container, the comma separated list.

6.1 Data Structures

Octave includes support for organizing data in structures. The current implementation uses an associative array with indices limited to strings, but the syntax is more like C-style structures.

6.1.1 Basic Usage and Examples

Here are some examples of using data structures in Octave.

Elements of structures can be of any value type. For example, the three expressions

```
x.a = 1;
x.b = [1, 2; 3, 4];
x.c = "string";
```

create a structure with three elements. To print the value of the structure, you can type its name, just as for any other variable:

```
x
     ⇒ x =
        {
          a = 1
          b =

            1  2
            3  4

          c = string
        }
```

Note that Octave may print the elements in any order.

Structures may be copied just like any other variable:

```
y = x
     ⇒ y =
        {
          a = 1
          b =

            1  2
            3  4

          c = string
        }
```

Since structures are themselves values, structure elements may reference other structures. The following statements change the value of the element `b` of the structure `x` to be a data structure containing the single element `d`, which has a value of 3.

```
x.b.d = 3;
x.b
     ⇒ ans =
        {
          d = 3
        }


x
     ⇒ x =
        {
          a = 1
          b =
          {
            d = 3
          }

          c = string
        }
```

Note that when Octave prints the value of a structure that contains other structures, only a few levels are displayed. For example,

```
a.b.c.d.e = 1;
a
     ⇒ a =
        {
          b =
          {
            c =
            {
              1x1 struct array containing the fields:

              d: 1x1 struct
            }
          }
        }
```

This prevents long and confusing output from large deeply nested structures. The number of levels to print for nested structures can be set with the function `struct_levels_to_print`:

val = `struct_levels_to_print` () [Built-in Function]
old_val = `struct_levels_to_print` (*new_val*) [Built-in Function]

Query or set the internal variable that specifies the number of structure levels to display.

Functions can return structures. For example, the following function separates the real and complex parts of a matrix and stores them in two elements of the same structure variable.

```
function y = f (x)
  y.re = real (x);
  y.im = imag (x);
endfunction
```

When called with a complex-valued argument, `f` returns the data structure containing the real and imaginary parts of the original function argument.

```
f (rand (2) + rand (2) * I)
     ⇒ ans =
        {
          im =

            0.26475  0.14828
            0.18436  0.83669

          re =

            0.040239  0.242160
            0.238081  0.402523

        }
```

Function return lists can include structure elements, and they may be indexed like any other variable. For example,

```
[ x.u, x.s(2:3,2:3), x.v ] = svd ([1, 2; 3, 4]);
x
     ⇒ x =
        {
          u =

            -0.40455  -0.91451
            -0.91451   0.40455

          s =

             0.00000   0.00000   0.00000
             0.00000   5.46499   0.00000
             0.00000   0.00000   0.36597

          v =

            -0.57605   0.81742
            -0.81742  -0.57605

        }
```

It is also possible to cycle through all the elements of a structure in a loop, using a special form of the for statement (see Section 10.5.1 [Looping Over Structure Elements], page 131).

6.1.2 Structure Arrays

A structure array is a particular instance of a structure, where each of the fields of the structure is represented by a cell array. Each of these cell arrays has the same dimensions. Conceptually, a structure array can also be seen as an array of structures with identical fields. An example of the creation of a structure array is

```
x(1).a = "string1";
x(2).a = "string2";
x(1).b = 1;
x(2).b = 2;
```

which creates a 2-by-1 structure array with two fields. Another way to create a structure array is with the struct function (see Section 6.1.3 [Creating Structures], page 81). As previously, to print the value of the structure array, you can type its name:

```
x
     ⇒ x =
        {
          1x2 struct array containing the fields:

            a
            b
        }
```

Individual elements of the structure array can be returned by indexing the variable like x(1), which returns a structure with two fields:

```
x(1)
     ⇒ ans =
        {
          a = string1
          b =  1
        }
```

Furthermore, the structure array can return a comma separated list of field values (see Section 6.3 [Comma Separated Lists], page 93), if indexed by one of its own field names. For example

```
x.a
     ⇒
        ans = string1
        ans = string2
```

Here is another example, using this comma separated list on the left-hand side of an assignment:

```
[x.a] = deal("new string1", "new string2");
 x(1).a
     ⇒ ans = new string1
 x(2).a
     ⇒ ans = new string2
```

Just as for numerical arrays, it is possible to use vectors as indices (see Section 8.1 [Index Expressions], page 107):

```
x(3:4) = x(1:2);
[x([1,3]).a] = deal("other string1", "other string2");
x.a
     ⇒
        ans = other string1
        ans = new string2
        ans = other string2
        ans = new string2
```

The function `size` will return the size of the structure. For the example above

```
size(x)
     ⇒ ans =

          1   4
```

Elements can be deleted from a structure array in a similar manner to a numerical array, by assigning the elements to an empty matrix. For example

```
in = struct ("call1", {x, Inf, "last"},
             "call2", {x, Inf, "first"})
     ⇒ in =
        {
          1x3 struct array containing the fields:

            call1
            call2
        }

in(1) = [];
in.call1
     ⇒
       ans = Inf
       ans = last
```

6.1.3 Creating Structures

As well as indexing a structure with ".", Octave can create a structure with the `struct` command. `struct` takes pairs of arguments, where the first argument in the pair is the fieldname to include in the structure and the second is a scalar or cell array, representing the values to include in the structure or structure array. For example

```
struct ("field1", 1, "field2", 2)
⇒ ans =
      {
        field1 =  1
        field2 =  2
      }
```

If the values passed to `struct` are a mix of scalar and cell arrays, then the scalar arguments are expanded to create a structure array with a consistent dimension. For example

```
s = struct ("field1", {1, "one"}, "field2", {2, "two"},
        "field3", 3);
s.field1
     ⇒
        ans =  1
        ans = one

s.field2
     ⇒
        ans =  2
        ans = two

s.field3
     ⇒
        ans =  3
        ans =  3
```

If you want to create a struct which contains a cell array as an individual field, you have to put it into another cell array like in the following example:

```
struct ("field1", {{1, "one"}}, "field2", 2)
     ⇒ ans =
        {
          field1 =

        {
          [1,1] =  1
          [1,2] = one
        }

          field2 =  2
        }
```

`struct` ("*field*", *value*, "*field*", *value*, ...) [Built-in Function]

Create a structure and initialize its value.

If the values are cell arrays, create a structure array and initialize its values. The dimensions of each cell array of values must match. Singleton cells and non-cell values are repeated so that they fill the entire array. If the cells are empty, create an empty structure array with the specified field names.

If the argument is an object, return the underlying struct.

The function `isstruct` can be used to test if an object is a structure or a structure array.

`isstruct (`*expr*`)` [Built-in Function]

Return 1 if the value of the expression *expr* is a structure.

6.1.4 Manipulating Structures

Other functions that can manipulate the fields of a structure are given below.

`rmfield (`*s*, *f*`)` [Built-in Function]

Remove field *f* from the structure *s*. If *f* is a cell array of character strings or a character array, remove the named fields.

See also: [cellstr], page 90, [iscellstr], page 91, [setfield], page 83.

`[`*k1*`, ..., `*v1*`] = setfield (`*s*, *k1*, *v1*, ...`)` [Function File]

Set field members in a structure.

```
oo(1,1).f0 = 1;
oo = setfield (oo, {1,2}, "fd", {3}, "b", 6);
oo(1,2).fd(3).b == 6
⇒ ans = 1
```

Note that this function could be written

```
i1 = {1,2}; i2 = "fd"; i3 = {3}; i4 = "b";
oo(i1{:}).(i2)(i3{:}).(i4) == 6;
```

See also: [getfield], page 83, [rmfield], page 83, [isfield], page 83, [isstruct], page 83, [fieldnames], page 83, [struct], page 82.

`[`*t*`, `*p*`] = orderfields (`*s1*, *s2*`)` [Function File]

Return a struct with fields arranged alphabetically or as specified by *s2* and a corresponding permutation vector.

Given one struct, arrange field names in *s1* alphabetically.

Given two structs, arrange field names in *s1* as they appear in *s2*. The second argument may also specify the order in a permutation vector or a cell array of strings.

See also: [getfield], page 83, [rmfield], page 83, [isfield], page 83, [isstruct], page 83, [fieldnames], page 83, [struct], page 82.

`fieldnames (`*struct*`)` [Built-in Function]

Return a cell array of strings naming the elements of the structure *struct*. It is an error to call `fieldnames` with an argument that is not a structure.

`isfield (`*expr*, *name*`)` [Built-in Function]

Return true if the expression *expr* is a structure and it includes an element named *name*. The first argument must be a structure and the second must be a string.

`[`*v1*`, ...] = getfield (`*s*, *key*, ...`)` [Function File]

Extract fields from a structure. For example

```
ss(1,2).fd(3).b = 5;
getfield (ss, {1,2}, "fd", {3}, "b")
⇒ ans = 5
```

Note that the function call in the previous example is equivalent to the expression

```
i1 = {1,2}; i2 = "fd"; i3 = {3}; i4= "b";
ss(i1{:}).(i2)(i3{:}).(i4)
```

See also: [setfield], page 83, [rmfield], page 83, [isfield], page 83, [isstruct], page 83, [fieldnames], page 83, [struct], page 82.

`substruct` (*type*, *subs*, ...) [Function File]

Create a subscript structure for use with `subsref` or `subsasgn`.

See also: [subsref], page 502, [subsasgn], page 504.

6.1.5 Processing Data in Structures

The simplest way to process data in a structure is within a `for` loop (see Section 10.5.1 [Looping Over Structure Elements], page 131). A similar effect can be achieved with the `structfun` function, where a user defined function is applied to each field of the structure.

`structfun` (*func*, *s*) [Function File]
`[a, b] = structfun` (...) [Function File]
`structfun` (..., "*ErrorHandler*", *errfunc*) [Function File]
`structfun` (..., "*UniformOutput*", *val*) [Function File]

Evaluate the function named *name* on the fields of the structure *s*. The fields of *s* are passed to the function *func* individually.

`structfun` accepts an arbitrary function *func* in the form of an inline function, function handle, or the name of a function (in a character string). In the case of a character string argument, the function must accept a single argument named *x*, and it must return a string value. If the function returns more than one argument, they are returned as separate output variables.

If the parameter "UniformOutput" is set to true (the default), then the function must return a single element which will be concatenated into the return value. If "UniformOutput" is false, the outputs placed in a structure with the same fieldnames as the input structure.

```
s.name1 = "John Smith";
s.name2 = "Jill Jones";
structfun (@(x) regexp (x, '(\w+)$', "matches"){1}, s,
           "UniformOutput", false)
```

Given the parameter "ErrorHandler", then *errfunc* defines a function to call in case *func* generates an error. The form of the function is

```
function [...] = errfunc (se, ...)
```

where there is an additional input argument to *errfunc* relative to *func*, given by *se*. This is a structure with the elements "identifier", "message" and "index", giving respectively the error identifier, the error message, and the index into the input arguments of the element that caused the error.

See also: [cellfun], page 91, [arrayfun], page 282.

Alternatively, to process the data in a structure, the structure might be converted to another type of container before being treated.

`struct2cell (S)` [Built-in Function]

Create a new cell array from the objects stored in the struct object. If *f* is the number of fields in the structure, the resulting cell array will have a dimension vector corresponding to `[F size(S)]`.

See also: [cell2struct], page 93, [fieldnames], page 83.

6.2 Cell Arrays

It can be both necessary and convenient to store several variables of different size or type in one variable. A cell array is a container class able to do just that. In general cell arrays work just like N-dimensional arrays with the exception of the use of '`{`' and '`}`' as allocation and indexing operators.

6.2.1 Basic Usage of Cell Arrays

As an example, the following code creates a cell array containing a string and a 2-by-2 random matrix

```
c = {"a string", rand(2, 2)};
```

To access the elements of a cell array, it can be indexed with the `{` and `}` operators. Thus, the variable created in the previous example can be indexed like this:

```
c{1}
     ⇒ ans = a string
```

As with numerical arrays several elements of a cell array can be extracted by indexing with a vector of indexes

```
c{1:2}
     ⇒ ans =

               (,
                 [1] = a string
                 [2] =

                    0.593993   0.627732
                    0.377037   0.033643

               ,)
```

The indexing operators can also be used to insert or overwrite elements of a cell array. The following code inserts the scalar 3 on the third place of the previously created cell array

```
c{3} = 3
     ⇒ c =

         {
           [1,1] = a string
           [1,2] =

              0.593993   0.627732
              0.377037   0.033643

           [1,3] =  3
         }
```

Details on indexing cell arrays are explained in Section 6.2.3 [Indexing Cell Arrays], page 88.

In general nested cell arrays are displayed hierarchically as in the previous example. In some circumstances it makes sense to reference them by their index, and this can be performed by the `celldisp` function.

`celldisp` (*c*, *name*) [Function File]

Recursively display the contents of a cell array. By default the values are displayed with the name of the variable *c*. However, this name can be replaced with the variable *name*.

See also: [disp], page 175.

To test if an object is a cell array, use the `iscell` function. For example:

```
iscell(c)
     ⇒ ans = 1

iscell(3)
     ⇒ ans = 0
```

`iscell` (*x*) [Built-in Function]

Return true if *x* is a cell array object. Otherwise, return false.

6.2.2 Creating Cell Array

The introductory example (see Section 6.2.1 [Basic Usage of Cell Arrays], page 85) showed how to create a cell array containing currently available variables. In many situations, however, it is useful to create a cell array and then fill it with data.

The `cell` function returns a cell array of a given size, containing empty matrices. This function is similar to the `zeros` function for creating new numerical arrays. The following example creates a 2-by-2 cell array containing empty matrices

```
c = cell(2,2)
     ⇒ c =

         {
           [1,1] = [](0x0)
           [2,1] = [](0x0)
           [1,2] = [](0x0)
           [2,2] = [](0x0)
         }
```

Just like numerical arrays, cell arrays can be multidimensional. The `cell` function accepts any number of positive integers to describe the size of the returned cell array. It is also possible to set the size of the cell array through a vector of positive integers. In the following example two cell arrays of equal size are created, and the size of the first one is displayed

```
c1 = cell(3, 4, 5);
c2 = cell( [3, 4, 5] );
size(c1)
     ⇒ ans =
         3   4   5
```

As can be seen, the [doc-size], page 36 function also works for cell arrays. As do other functions describing the size of an object, such as [doc-length], page 36, [doc-numel], page 36, [doc-rows], page 36, and [doc-columns], page 35.

`cell` (*x*) [Built-in Function]
`cell` (*n*, *m*) [Built-in Function]

Create a new cell array object. If invoked with a single scalar argument, `cell` returns a square cell array with the dimension specified. If you supply two scalar arguments, `cell` takes them to be the number of rows and columns. If given a vector with two elements, `cell` uses the values of the elements as the number of rows and columns, respectively.

As an alternative to creating empty cell arrays, and then filling them, it is possible to convert numerical arrays into cell arrays using the `num2cell` and `mat2cell` functions.

c = `num2cell` (*m*) [Loadable Function]
c = `num2cell` (*m*, *dim*) [Loadable Function]

Convert the matrix *m* to a cell array. If *dim* is defined, the value *c* is of dimension 1 in this dimension and the elements of *m* are placed in slices in *c*.

See also: [mat2cell], page 87.

b = `mat2cell` (a, *m*, *n*) [Loadable Function]
b = `mat2cell` (a, *d1*, *d2*, . . .) [Loadable Function]
b = `mat2cell` (a, *r*) [Loadable Function]

Convert the matrix *a* to a cell array. If *a* is 2-D, then it is required that `sum (m) == size (a, 1)` and `sum (n) == size (a, 2)`. Similarly, if *a* is a multi-dimensional and the number of dimensional arguments is equal to the dimensions of *a*, then it is required that `sum (di) == size (a, i)`.

Given a single dimensional argument *r*, the other dimensional arguments are assumed to equal `size (a,i)`.

An example of the use of mat2cell is

```
mat2cell (reshape(1:16,4,4),[3,1],[3,1])
⇒ {
  [1,1] =

     1   5   9
     2   6  10
     3   7  11

  [2,1] =

     4   8  12

  [1,2] =

    13
    14
    15

  [2,2] = 16
}
```

See also: [num2cell], page 87, [cell2mat], page 92.

6.2.3 Indexing Cell Arrays

As shown in see Section 6.2.1 [Basic Usage of Cell Arrays], page 85 elements can be extracted from cell arrays using the '`{`' and '`}`' operators. If you want to extract or access subarrays which are still cell arrays, you need to use the '`(`' and '`)`' operators. The following example illustrates the difference:

```
c = {"1", "2", "3"; "a", "b", "c"; "4", "5", "6"};
c{2,3}
     ⇒ ans = c

c(2,3)
     ⇒ ans =
        {
          [1,1] = c
        }
```

So with '`{}`' you access elements of a cell array, while with '`()`' you access a sub array of a cell array.

Using the '`(`' and '`)`' operators, indexing works for cell arrays like for multidimensional arrays. As an example, all the rows of the first and third column of a cell array can be set to `0` with the following command:

```
c(:, [1, 3]) = {0}
     ⇒  =
        {
          [1,1] = 0
          [2,1] = 0
          [3,1] = 0
          [1,2] = 2
          [2,2] =  10
          [3,2] =  20
          [1,3] = 0
          [2,3] = 0
          [3,3] = 0
        }
```

Note, that the above can also be achieved like this:

```
c(:, [1, 3]) = 0;
```

Here, the scalar '0' is automatically promoted to cell array '{0}' and then assigned to the subarray of c.

To give another example for indexing cell arrays with '()', you can exchange the first and the second row of a cell array as in the following command:

```
c = {1, 2, 3; 4, 5, 6};
c([1, 2], :) = c([2, 1], :)
     ⇒ =
        {
          [1,1] =  4
          [2,1] =  1
          [1,2] =  5
          [2,2] =  2
          [1,3] =  6
          [2,3] =  3
        }
```

Accessing multiple elements of a cell array with the '{' and '}' operators will result in a comma-separated list of all the requested elements (see Section 6.3 [Comma Separated Lists], page 93). Using the '{' and '}' operators the first two rows in the above example can be swapped back like this:

```
[c{[1,2], :}] = deal(c{[2, 1], :})
     ⇒ =
        {
          [1,1] =  1
          [2,1] =  4
          [1,2] =  2
          [2,2] =  5
          [1,3] =  3
          [2,3] =  6
        }
```

As for struct arrays and numerical arrays, the empty matrix '[]' can be used to delete elements from a cell array:

```
x = {"1", "2"; "3", "4"};
x(1, :) = []
     ⇒ x =
        {
          [1,1] = 3
          [1,2] = 4
        }
```

The following example shows how to just remove the contents of cell array elements but not delete the space for them:

```
x = {"1", "2"; "3", "4"};
x{1, :} = []
⇒ x =
      {
        [1,1] = [](0x0)
        [2,1] = 3
        [1,2] = [](0x0)
        [2,2] = 4
      }
```

6.2.4 Cell Arrays of Strings

One common use of cell arrays is to store multiple strings in the same variable. It is also possible to store multiple strings in a character matrix by letting each row be a string. This, however, introduces the problem that all strings must be of equal length. Therefore, it is recommended to use cell arrays to store multiple strings. For cases, where the character matrix representation is required for an operation, there are several functions that convert a cell array of strings to a character array and back. `char` and `strvcat` convert cell arrays to a character array (see Section 5.3.1 [Concatenating Strings], page 57), while the function `cellstr` converts a character array to a cell array of strings:

```
a = ["hello"; "world"];
c = cellstr (a)
     ⇒ c =
         {
           [1,1] = hello
           [2,1] = world
         }
```

`cellstr` (*string*) [Built-in Function]

Create a new cell array object from the elements of the string array *string*.

One further advantage of using cell arrays to store multiple strings is that most functions for string manipulations included with Octave support this representation. As an example, it is possible to compare one string with many others using the `strcmp` function. If one of the arguments to this function is a string and the other is a cell array of strings, each element of the cell array will be compared to the string argument:

```
c = {"hello", "world"};
strcmp ("hello", c)
     ⇒ ans =
        1   0
```

The following string functions support cell arrays of strings: `char`, `strvcat`, `strcat` (see Section 5.3.1 [Concatenating Strings], page 57), `strcmp`, `strncmp`, `strcmpi`, `strncmpi` (see Section 5.4 [Comparing Strings], page 62), `str2double`, `deblank`, `strtrim`, `strtrunc`, `strfind`, `strmatch`, , `regexp`, `regexpi` (see Section 5.5 [Manipulating Strings], page 64) and `str2double` (see Section 5.6 [String Conversions], page 70).

The function `iscellstr` can be used to test if an object is a cell array of strings.

`iscellstr` (*cell*) [Built-in Function]

Return true if every element of the cell array *cell* is a character string

[*idxvec*, *errmsg*] = `cellidx` (*listvar*, *strlist*) [Function File]

Return indices of string entries in *listvar* that match strings in *strlist*.

Both *listvar* and *strlist* may be passed as strings or string matrices. If they are passed as string matrices, each entry is processed by `deblank` prior to searching for the entries.

The first output is the vector of indices in *listvar*.

If *strlist* contains a string not in *listvar*, then an error message is returned in *errmsg*. If only one output argument is requested, then *cellidx* prints *errmsg* to the screen and exits with an error.

6.2.5 Processing Data in Cell Arrays

Data that is stored in a cell array can be processed in several ways depending on the actual data. The simplest way to process that data is to iterate through it using one or more `for` loops. The same idea can be implemented more easily through the use of the `cellfun` function that calls a user-specified function on all elements of a cell array.

`cellfun` (*name*, *c*) [Loadable Function]
`cellfun` ("*size*", *c*, *k*) [Loadable Function]
`cellfun` ("*isclass*", *c*, *class*) [Loadable Function]
`cellfun` (*func*, *c*) [Loadable Function]
`cellfun` (*func*, *c*, *d*) [Loadable Function]
[*a*, *b*] = `cellfun` (...) [Loadable Function]
`cellfun` (..., *'ErrorHandler'*, *errfunc*) [Loadable Function]
`cellfun` (..., *'UniformOutput'*, *val*) [Loadable Function]

Evaluate the function named *name* on the elements of the cell array *c*. Elements in *c* are passed on to the named function individually. The function *name* can be one of the functions

`isempty` Return 1 for empty elements.

`islogical`
Return 1 for logical elements.

`isreal` Return 1 for real elements.

`length` Return a vector of the lengths of cell elements.

`ndims` Return the number of dimensions of each element.

`prodofsize`
Return the product of dimensions of each element.

`size` Return the size along the *k*-th dimension.

`isclass` Return 1 for elements of *class*.

Additionally, `cellfun` accepts an arbitrary function *func* in the form of an inline function, function handle, or the name of a function (in a character string). In the case of a character string argument, the function must accept a single argument named *x*, and it must return a string value. The function can take one or more arguments, with the inputs args given by *c*, *d*, etc. Equally the function can return one or more output arguments. For example

```
cellfun (@atan2, {1, 0}, {0, 1})
⇒ans = [1.57080   0.00000]
```

Note that the default output argument is an array of the same size as the input arguments.

If the parameter 'UniformOutput' is set to true (the default), then the function must return a single element which will be concatenated into the return value. If 'UniformOutput' is false, the outputs are concatenated in a cell array. For example

```
cellfun ("tolower(x)", {"Foo", "Bar", "FooBar"},
         "UniformOutput",false)
⇒ ans = {"foo", "bar", "foobar"}
```

Given the parameter 'ErrorHandler', then *errfunc* defines a function to call in case *func* generates an error. The form of the function is

```
function [...] = errfunc (s, ...)
```

where there is an additional input argument to *errfunc* relative to *func*, given by *s*. This is a structure with the elements 'identifier', 'message' and 'index', giving respectively the error identifier, the error message, and the index into the input arguments of the element that caused the error. For example

```
function y = foo (s, x), y = NaN; endfunction
cellfun (@factorial, {-1,2},'ErrorHandler',@foo)
⇒ ans = [NaN 2]
```

See also: [isempty], page 36, [islogical], page 53, [isreal], page 52, [length], page 36, [ndims], page 35, [numel], page 36, [size], page 36.

An alternative is to convert the data to a different container, such as a matrix or a data structure. Depending on the data this is possible using the `cell2mat` and `cell2struct` functions.

m = `cell2mat` (*c*) [Function File]

Convert the cell array *c* into a matrix by concatenating all elements of *c* into a hyperrectangle. Elements of *c* must be numeric, logical or char, and `cat` must be able to concatenate them together.

See also: [mat2cell], page 87, [num2cell], page 87.

`cell2struct` (*cell*, *fields*, *dim*) [Built-in Function]

Convert *cell* to a structure. The number of fields in *fields* must match the number of elements in *cell* along dimension *dim*, that is `numel (fields) == size (cell, dim)`.

```
A = cell2struct ({'Peter', 'Hannah', 'Robert';
                   185, 170, 168},
                  {'Name','Height'}, 1);
A(1)
⇒ ans =
      {
        Height = 185
        Name   = Peter
      }
```

6.3 Comma Separated Lists

Comma separated lists[1] are the basic argument type to all Octave functions - both for input and return arguments. In the example

```
max (a, b)
```

'`a, b`' is a comma separated list. Comma separated lists can appear on both the right and left hand side of an assignment. For example

```
x = [1 0 1 0 0 1 1; 0 0 0 0 0 0 7];
[i, j] = find (x, 2, "last");
```

Here, '`x, 2, "last"`' is a comma separated list constituting the input arguments of `find`. `find` returns a comma separated list of output arguments which is assigned element by element to the comma separated list '`i, j`'.

Another example of where comma separated lists are used is in the creation of a new array with `[]` (see Section 4.1 [Matrices], page 40) or the creation of a cell array with `{}` (see Section 6.2.1 [Basic Usage of Cell Arrays], page 85). In the expressions

```
a = [1, 2, 3, 4];
c = {4, 5, 6, 7};
```

both '`1, 2, 3, 4`' and '`4, 5, 6, 7`' are comma separated lists.

Comma separated lists cannot be directly manipulated by the user. However, both structure arrays and cell arrays can be converted into comma separated lists, and thus used in place of explicitly written comma separated lists. This feature is useful in many ways, as will be shown in the following subsections.

6.3.1 Comma Separated Lists Generated from Cell Arrays

As has been mentioned above (see Section 6.2.3 [Indexing Cell Arrays], page 88), elements of a cell array can be extracted into a comma separated list with the `{` and `}` operators. By surrounding this list with `[` and `]`, it can be concatenated into an array. For example:

[1] Comma-separated lists are also sometimes informally referred to as *cs-lists*.

```
a = {1, [2, 3], 4, 5, 6};
b = [a{1:4}]
     ⇒ b =
         1   2   3   4
```

Similarly, it is possible to create a new cell array containing cell elements selected with `{}`. By surrounding the list with '`{`' and '`}`' a new cell array will be created, as the following example illustrates:

```
a = {1, rand(2, 2), "three"};
b = { a{ [1, 3] } }
     ⇒ b =
         {
           [1,1] =  1
           [1,2] = three
         }
```

Furthermore, cell elements (accessed by `{}`) can be passed directly to a function. The list of elements from the cell array will be passed as an argument list to a given function as if it is called with the elements as individual arguments. The two calls to `printf` in the following example are identical but the latter is simpler and can handle cell arrays of an arbitrary size:

```
c = {"GNU", "Octave", "is", "Free", "Software"};
printf ("%s ", c{1}, c{2}, c{3}, c{4}, c{5});
     ⊣ GNU Octave is Free Software
printf ("%s ", c{:});
     ⊣ GNU Octave is Free Software
```

If used on the left-hand side of an assignment, a comma separated list generated with `{}` can be assigned to. An example is

```
in{1} = [10, 20, 30, 40, 50, 60, 70, 80, 90];
in{2} = inf;
in{3} = "last";
in{4} = "first";
out = cell (4, 1);
[out{1:3}] = find (in{1 : 3});
[out{4:6}] = find (in{[1, 2, 4]})
     ⇒ out =
        {
          [1,1] = 1
          [2,1] = 9
          [3,1] = 90
          [4,1] = 1
          [3,1] = 1
          [4,1] = 10
        }
```

6.3.2 Comma Separated Lists Generated from Structure Arrays

Structure arrays can equally be used to create comma separated lists. This is done by addressing one of the fields of a structure array. For example

```
x = ceil (randn (10, 1));
in = struct ("call1", {x, 3, "last"},
             "call2", {x, inf, "first"});
out = struct ("call1", cell (2, 1), "call2", cell (2, 1));
[out.call1] = find (in.call1);
[out.call2] = find (in.call2);
```

7 Variables

Variables let you give names to values and refer to them later. You have already seen variables in many of the examples. The name of a variable must be a sequence of letters, digits and underscores, but it may not begin with a digit. Octave does not enforce a limit on the length of variable names, but it is seldom useful to have variables with names longer than about 30 characters. The following are all valid variable names

```
x
x15
__foo_bar_baz__
fucnrdthsucngtagdjb
```

However, names like `__foo_bar_baz__` that begin and end with two underscores are understood to be reserved for internal use by Octave. You should not use them in code you write, except to access Octave's documented internal variables and built-in symbolic constants.

Case is significant in variable names. The symbols `a` and `A` are distinct variables.

A variable name is a valid expression by itself. It represents the variable's current value. Variables are given new values with *assignment operators* and *increment operators*. See Section 8.6 [Assignment Expressions], page 115.

There is one built-in variable with a special meaning. The `ans` variable always contains the result of the last computation, where the output wasn't assigned to any variable. The code `a = cos (pi)` will assign the value -1 to the variable `a`, but will not change the value of `ans`. However, the code `cos (pi)` will set the value of `ans` to -1.

Variables in Octave do not have fixed types, so it is possible to first store a numeric value in a variable and then to later use the same name to hold a string value in the same program. Variables may not be used before they have been given a value. Doing so results in an error.

`ans` [Automatic Variable]

The most recently computed result that was not explicitly assigned to a variable. For example, after the expression

```
3^2 + 4^2
```

is evaluated, the value returned by `ans` is 25.

`isvarname (`*name*`)` [Built-in Function]

Return true if *name* is a valid variable name

varname `= genvarname (`*str*`)` [Function File]
varname `= genvarname (`*str*, *exclusions*`)` [Function File]

Create unique variable(s) from *str*. If *exclusions* is given, then the variable(s) will be unique to each other and to *exclusions* (*exclusions* may be either a string or a cellstr).

If *str* is a cellstr, then a unique variable is created for each cell in *str*.

```
x = 3.141;
genvarname ("x", who ())
⇒ x1
```

If *wanted* is a cell array, genvarname will make sure the returned strings are distinct:

```
genvarname ({"foo", "foo"})
⇒
{
  [1,1] = foo
  [1,2] = foo1
}
```

Note that the result is a char array/cell array of strings, not the variables themselves. To define a variable, `eval()` can be used. The following trivial example sets `x` to `42`.

```
name = genvarname ("x");
eval([name " = 42"]);
⇒ x =  42
```

Also, this can be useful for creating unique struct field names.

```
x = struct ();
for i = 1:3
  x.(genvarname ("a", fieldnames (x))) = i;
endfor
⇒
x =
{
  a =  1
  a1 =  2
  a2 =  3
}
```

Since variable names may only contain letters, digits and underscores, genvarname replaces any sequence of disallowed characters with an underscore. Also, variables may not begin with a digit; in this case an underscore is added before the variable name.

Variable names beginning and ending with two underscores "__" are valid but they are used internally by octave and should generally be avoided, therefore genvarname will not generate such names.

genvarname will also make sure that returned names do not clash with keywords such as "for" and "if". A number will be appended if necessary. Note, however, that this does **not** include function names, such as "sin". Such names should be included in *avoid* if necessary.

See also: [isvarname], page 97, [exist], page 104, [tmpnam], page 202, [eval], page 121.

`namelengthmax` () [Function File]

Returns the MATLAB compatible maximum variable name length. Octave is capable of storing strings up to $2^{31} - 1$ in length. However for MATLAB compatibility all variable, function and structure field names should be shorter than the length supplied by `namelengthmax`. In particular variables stored to a MATLAB file format will have their names truncated to this length.

7.1 Global Variables

A variable that has been declared *global* may be accessed from within a function body without having to pass it as a formal parameter.

A variable may be declared global using a `global` declaration statement. The following statements are all global declarations.

```
global a
global a b
global c = 2
global d = 3 e f = 5
```

A global variable may only be initialized once in a `global` statement. For example, after executing the following code

```
global gvar = 1
global gvar = 2
```

the value of the global variable `gvar` is 1, not 2. Issuing a '`clear gvar`' command does not change the above behavior, but '`clear all`' does.

It is necessary declare a variable as global within a function body in order to access it. For example,

```
global x
function f ()
  x = 1;
endfunction
f ()
```

does *not* set the value of the global variable `x` to 1. In order to change the value of the global variable `x`, you must also declare it to be global within the function body, like this

```
function f ()
  global x;
  x = 1;
endfunction
```

Passing a global variable in a function parameter list will make a local copy and not modify the global value. For example, given the function

```
function f (x)
  x = 0
endfunction
```

and the definition of `x` as a global variable at the top level,

```
global x = 13
```

the expression

```
f (x)
```

will display the value of `x` from inside the function as 0, but the value of `x` at the top level remains unchanged, because the function works with a *copy* of its argument.

`isglobal` (*name*) [Built-in Function]

Return 1 if *name* is globally visible. Otherwise, return 0. For example,

```
global x
isglobal ("x")
     ⇒ 1
```

7.2 Persistent Variables

A variable that has been declared *persistent* within a function will retain its contents in memory between subsequent calls to the same function. The difference between persistent variables and global variables is that persistent variables are local in scope to a particular function and are not visible elsewhere.

The following example uses a persistent variable to create a function that prints the number of times it has been called.

```
function count_calls ()
  persistent calls = 0;
  printf ("'count_calls' has been called %d times\n",
          ++calls);
endfunction

for i = 1:3
  count_calls ();
endfor

⊣ 'count_calls' has been called 1 times
⊣ 'count_calls' has been called 2 times
⊣ 'count_calls' has been called 3 times
```

As the example shows, a variable may be declared persistent using a `persistent` declaration statement. The following statements are all persistent declarations.

```
persistent a
persistent a b
persistent c = 2
persistent d = 3 e f = 5
```

The behavior of persistent variables is equivalent to the behavior of static variables in C. The command `static` in Octave is also recognized and is equivalent to `persistent`.

Like global variables, a persistent variable may only be initialized once. For example, after executing the following code

```
persistent pvar = 1
persistent pvar = 2
```

the value of the persistent variable `pvar` is 1, not 2.

If a persistent variable is declared but not initialized to a specific value, it will contain an empty matrix. So, it is also possible to initialize a persistent variable by checking whether it is empty, as the following example illustrates.

```
function count_calls ()
  persistent calls;
  if (isempty (calls))
    calls = 0;
  endif
  printf ("'count_calls' has been called %d times\n",
          ++calls);
endfunction
```

This implementation behaves in exactly the same way as the previous implementation of `count_calls`.

The value of a persistent variable is kept in memory until it is explicitly cleared. Assuming that the implementation of `count_calls` is saved on disk, we get the following behavior.

```
for i = 1:2
  count_calls ();
endfor
⊣ 'count_calls' has been called 1 times
⊣ 'count_calls' has been called 2 times

clear
for i = 1:2
  count_calls();
endfor
⊣ 'count_calls' has been called 3 times
⊣ 'count_calls' has been called 4 times

clear all
for i = 1:2
  count_calls();
endfor
⊣ 'count_calls' has been called 1 times
⊣ 'count_calls' has been called 2 times

clear count_calls
for i = 1:2
  count_calls();
endfor
⊣ 'count_calls' has been called 1 times
⊣ 'count_calls' has been called 2 times
```

That is, the persistent variable is only removed from memory when the function containing the variable is removed. Note that if the function definition is typed directly into the Octave prompt, the persistent variable will be cleared by a simple `clear` command as the entire function definition will be removed from memory. If you do not want a persistent variable to be removed from memory even if the function is cleared, you should use the `mlock` function as described in See Section 11.7.5 [Function Locking], page 152.

7.3 Status of Variables

When creating simple one-shot programs it can be very convenient to see which variables are available at the prompt. The function `who` and its siblings `whos` and `whos_line_format` will show different information about what is in memory, as the following shows.

```
str = "A random string";
who -variables
     -| *** local user variables:
     -|
     -| __nargin__  str
```

`who` [Command]
`who` *pattern* ... [Command]
`who` *option pattern* ... [Command]
`C` = *who*("*pattern*", ...) [Command]

List currently defined variables matching the given patterns. Valid pattern syntax is the same as described for the `clear` command. If no patterns are supplied, all variables are listed. By default, only variables visible in the local scope are displayed.

The following are valid options but may not be combined.

`global` List variables in the global scope rather than the current scope.

`-regexp` The patterns are considered to be regular expressions when matching the variables to display. The same pattern syntax accepted by the `regexp` function is used.

`-file` The next argument is treated as a filename. All variables found within the specified file are listed. No patterns are accepted when reading variables from a file.

If called as a function, return a cell array of defined variable names matching the given patterns.

See also: [whos], page 102, [regexp], page 67.

`whos` [Command]
`whos` *pattern* ... [Command]
`whos` *option pattern* ... [Command]
`S` = *whos*("*pattern*", ...) [Command]

Provide detailed information on currently defined variables matching the given patterns. Options and pattern syntax are the same as for the `who` command. Extended information about each variable is summarized in a table with the following default entries.

Attr Attributes of the listed variable. Possible attributes are:

blank Variable in local scope

`g` Variable with global scope

`p` Persistent variable

Name The name of the variable.

Size — The logical size of the variable. A scalar is 1x1, a vector is 1xN or Nx1, a 2-D matrix is MxN.

Bytes — The amount of memory currently used to store the variable.

Class — The class of the variable. Examples include double, single, char, uint16, cell, and struct.

The table can be customized to display more or less information through the function `whos_line_format`.

If `whos` is called as a function, return a struct array of defined variable names matching the given patterns. Fields in the structure describing each variable are: name, size, bytes, class, global, sparse, complex, nesting, persistent.

See also: [who], page 102, [whos_line_format], page 103.

val = `whos_line_format ()` [Built-in Function]
old_val = `whos_line_format (`*new_val*`)` [Built-in Function]

Query or set the format string used by the command `whos`.

A full format string is:

```
%[modifier]<command>[:width[:left-min[:balance]]];
```

The following command sequences are available:

`%a` — Prints attributes of variables (g=global, p=persistent, f=formal parameter, a=automatic variable).

`%b` — Prints number of bytes occupied by variables.

`%c` — Prints class names of variables.

`%e` — Prints elements held by variables.

`%n` — Prints variable names.

`%s` — Prints dimensions of variables.

`%t` — Prints type names of variables.

Every command may also have an alignment modifier:

`l` — Left alignment.

`r` — Right alignment (default).

`c` — Column-aligned (only applicable to command %s).

The `width` parameter is a positive integer specifying the minimum number of columns used for printing. No maximum is needed as the field will auto-expand as required.

The parameters `left-min` and `balance` are only available when the column-aligned modifier is used with the command '`%s`'. `balance` specifies the column number within the field width which will be aligned between entries. Numbering starts from 0 which indicates the leftmost column. `left-min` specifies the minimum field width to the left of the specified balance column.

The default format is `" %a:4; %ln:6; %cs:16:6:1; %rb:12; %lc:-1;\n"`.

See also: [whos], page 102.

Instead of displaying which variables are in memory, it is possible to determine if a given variable is available. That way it is possible to alter the behavior of a program depending on the existence of a variable. The following example illustrates this.

```
if (! exist ("meaning", "var"))
  disp ("The program has no 'meaning'");
endif
```

`exist` (*name*, *type*) [Built-in Function]

Return 1 if the name exists as a variable, 2 if the name is an absolute file name, an ordinary file in Octave's `path`, or (after appending '`.m`') a function file in Octave's `path`, 3 if the name is a '`.oct`' or '`.mex`' file in Octave's `path`, 5 if the name is a built-in function, 7 if the name is a directory, or 103 if the name is a function not associated with a file (entered on the command line).

Otherwise, return 0.

This function also returns 2 if a regular file called *name* exists in Octave's search path. If you want information about other types of files, you should use some combination of the functions `file_in_path` and `stat` instead.

If the optional argument *type* is supplied, check only for symbols of the specified type. Valid types are

'`"var"`' Check only for variables.

'`"builtin"`'
Check only for built-in functions.

'`"file"`' Check only for files.

'`"dir"`' Check only for directories.

Usually Octave will manage the memory, but sometimes it can be practical to remove variables from memory manually. This is usually needed when working with large variables that fill a substantial part of the memory. On a computer that uses the IEEE floating point format, the following program allocates a matrix that requires around 128 MB memory.

```
large_matrix = zeros (4000, 4000);
```

Since having this variable in memory might slow down other computations, it can be necessary to remove it manually from memory. The `clear` function allows this.

`clear` [*options*] *pattern* . . . [Command]

Delete the names matching the given patterns from the symbol table. The pattern may contain the following special characters:

`?` Match any single character.

`*` Match zero or more characters.

`[ list ]` Match the list of characters specified by *list*. If the first character is `!` or `^`, match all characters except those specified by *list*. For example, the pattern '`[a-zA-Z]`' will match all lower and upper case alphabetic characters.

For example, the command

```
clear foo b*r
```

clears the name `foo` and all names that begin with the letter `b` and end with the letter `r`.

If `clear` is called without any arguments, all user-defined variables (local and global) are cleared from the symbol table. If `clear` is called with at least one argument, only the visible names matching the arguments are cleared. For example, suppose you have defined a function `foo`, and then hidden it by performing the assignment `foo = 2`. Executing the command *clear foo* once will clear the variable definition and restore the definition of `foo` as a function. Executing *clear foo* a second time will clear the function definition.

The following options are available in both long and short form

`-all, -a` Clears all local and global user-defined variables and all functions from the symbol table.

`-exclusive, -x`
Clears the variables that don't match the following pattern.

`-functions, -f`
Clears the function names and the built-in symbols names.

`-global, -g`
Clears the global symbol names.

`-variables, -v`
Clears the local variable names.

`-classes, -c`
Clears the class structure table and clears all objects.

`-regexp, -r`
The arguments are treated as regular expressions as any variables that match will be cleared.

With the exception of `exclusive`, all long options can be used without the dash as well.

Information about a function or variable such as its location in the file system can also be acquired from within Octave. This is usually only useful during development of programs, and not within a program.

`type options` *name* . . . [Command]

Display the definition of each *name* that refers to a function.

Normally also displays whether each *name* is user-defined or built-in; the `-q` option suppresses this behavior.

If an output argument is requested nothing is displayed. Instead, a cell array of strings is returned, where each element corresponds to the definition of each requested function.

`which` *name* . . . [Command]

Display the type of each *name*. If *name* is defined from a function file, the full name of the file is also displayed.

See also: [help], page 17, [lookfor], page 18.

`what` [Command]
`what` *`dir`* [Command]
`w = what (`*`dir`*`)` [Function File]

List the Octave specific files in a directory. If the variable *dir* is given then check that directory rather than the current directory. If a return argument is requested, the files found are returned in the structure *w*.

See also: [which], page 105.

8 Expressions

Expressions are the basic building block of statements in Octave. An expression evaluates to a value, which you can print, test, store in a variable, pass to a function, or assign a new value to a variable with an assignment operator.

An expression can serve as a statement on its own. Most other kinds of statements contain one or more expressions which specify data to be operated on. As in other languages, expressions in Octave include variables, array references, constants, and function calls, as well as combinations of these with various operators.

8.1 Index Expressions

An *index expression* allows you to reference or extract selected elements of a matrix or vector.

Indices may be scalars, vectors, ranges, or the special operator ':', which may be used to select entire rows or columns.

Vectors are indexed using a single index expression. Matrices may be indexed using one or two indices. When using a single index expression, the elements of the matrix are taken in column-first order; the dimensions of the output match those of the index expression. For example,

```
a (2)       # a scalar
a (1:2)     # a row vector
a ([1; 2])  # a column vector
```

As a special case, when a colon is used as a single index, the output is a column vector containing all the elements of the vector or matrix. For example

```
a (:)       # a column vector
```

Given the matrix

```
a = [1, 2; 3, 4]
```

all of the following expressions are equivalent

```
a (1, [1, 2])
a (1, 1:2)
a (1, :)
```

and select the first row of the matrix.

In general, an array with 'n' dimensions can be indexed using 'm' indices. If `n == m`, each index corresponds to its respective dimension. The set of index tuples determining the result is formed by the Cartesian product of the index vectors (or ranges or scalars). If `n < m`, then the array is padded by trailing singleton dimensions. If `n > m`, the last `n-m+1` dimensions are folded into a single dimension with extent equal to product of extents of the original dimensions.

Indexing a scalar with a vector of ones can be used to create a vector the same size as the index vector, with each element equal to the value of the original scalar. For example, the following statements

```
a = 13;
a (ones (1, 4))
```

produce a vector whose four elements are all equal to 13.

Similarly, indexing a scalar with two vectors of ones can be used to create a matrix. For example the following statements

```
a = 13;
a (ones (1, 2), ones (1, 3))
```

create a 2 by 3 matrix with all elements equal to 13.

The last example could also be written as

```
13 (ones (2, 3))
```

It should be, noted that `ones (1, n)` (a row vector of ones) results in a range (with zero increment), and is therefore more efficient when used in index expression than other forms of *ones*. In particular, when '`r`' is a row vector, the expressions

```
r(ones (1, n), :)

r(ones (n, 1), :)
```

will produce identical results, but the first one will be significantly faster, at least for '`r`' and '`n`' large enough. The reason is that in the first case the index is kept in a compressed form, which allows Octave to choose a more efficient algorithm to handle the expression.

In general, for an user unaware of these subtleties, it is best to use the function *repmat* for spreading arrays into bigger ones.

It is also possible to create a matrix with different values. The following example creates a 10 dimensional row vector a containing the values $a_i = \sqrt{i}$.

```
for i = 1:10
  a(i) = sqrt (i);
endfor
```

Note that it is quite inefficient to create a vector using a loop like the one shown in the example above. In this particular case, it would have been much more efficient to use the expression

```
a = sqrt (1:10);
```

thus avoiding the loop entirely. In cases where a loop is still required, or a number of values must be combined to form a larger matrix, it is generally much faster to set the size of the matrix first, and then insert elements using indexing commands. For example, given a matrix `a`,

```
[nr, nc] = size (a);
x = zeros (nr, n * nc);
for i = 1:n
  x(:,(i-1)*nc+1:i*nc) = a;
endfor
```

is considerably faster than

```
x = a;
for i = 1:n-1
  x = [x, a];
endfor
```

particularly for large matrices because Octave does not have to repeatedly resize the result.

ind = sub2ind (*dims*, *i*, *j*) [Function File]
ind = sub2ind (*dims*, *s1*, *s2*, . . ., *sN*) [Function File]

Convert subscripts into a linear index.

The following example shows how to convert the two-dimensional index (2,3) of a 3-by-3 matrix to a linear index. The matrix is linearly indexed moving from one column to next, filling up all rows in each column.

```
linear_index = sub2ind ([3, 3], 2, 3)
⇒ 8
```

See also: [ind2sub], page 109.

[*s1*, *s2*, ..., *sN*] = ind2sub (*dims*, *ind*) [Function File]

Convert a linear index into subscripts.

The following example shows how to convert the linear index 8 in a 3-by-3 matrix into a subscript. The matrix is linearly indexed moving from one column to next, filling up all rows in each column.

```
[r, c] = ind2sub ([3, 3], 8)
⇒ r =  2
c =  3
```

See also: [sub2ind], page 108.

8.2 Calling Functions

A *function* is a name for a particular calculation. Because it has a name, you can ask for it by name at any point in the program. For example, the function sqrt computes the square root of a number.

A fixed set of functions are *built-in*, which means they are available in every Octave program. The sqrt function is one of these. In addition, you can define your own functions. See Chapter 11 [Functions and Scripts], page 137, for information about how to do this.

The way to use a function is with a *function call* expression, which consists of the function name followed by a list of *arguments* in parentheses. The arguments are expressions which give the raw materials for the calculation that the function will do. When there is more than one argument, they are separated by commas. If there are no arguments, you can omit the parentheses, but it is a good idea to include them anyway, to clearly indicate that a function call was intended. Here are some examples:

```
sqrt (x^2 + y^2)      # One argument
ones (n, m)           # Two arguments
rand ()               # No arguments
```

Each function expects a particular number of arguments. For example, the sqrt function must be called with a single argument, the number to take the square root of:

```
sqrt (argument)
```

Some of the built-in functions take a variable number of arguments, depending on the particular usage, and their behavior is different depending on the number of arguments supplied.

Like every other expression, the function call has a value, which is computed by the function based on the arguments you give it. In this example, the value of sqrt (*argument*)

is the square root of the argument. A function can also have side effects, such as assigning the values of certain variables or doing input or output operations.

Unlike most languages, functions in Octave may return multiple values. For example, the following statement

```
[u, s, v] = svd (a)
```

computes the singular value decomposition of the matrix `a` and assigns the three result matrices to `u`, `s`, and `v`.

The left side of a multiple assignment expression is itself a list of expressions, and is allowed to be a list of variable names or index expressions. See also Section 8.1 [Index Expressions], page 107, and Section 8.6 [Assignment Ops], page 115.

8.2.1 Call by Value

In Octave, unlike Fortran, function arguments are passed by value, which means that each argument in a function call is evaluated and assigned to a temporary location in memory before being passed to the function. There is currently no way to specify that a function parameter should be passed by reference instead of by value. This means that it is impossible to directly alter the value of a function parameter in the calling function. It can only change the local copy within the function body. For example, the function

```
function f (x, n)
  while (n-- > 0)
    disp (x);
  endwhile
endfunction
```

displays the value of the first argument *n* times. In this function, the variable *n* is used as a temporary variable without having to worry that its value might also change in the calling function. Call by value is also useful because it is always possible to pass constants for any function parameter without first having to determine that the function will not attempt to modify the parameter.

The caller may use a variable as the expression for the argument, but the called function does not know this: it only knows what value the argument had. For example, given a function called as

```
foo = "bar";
fcn (foo)
```

you should not think of the argument as being "the variable `foo`." Instead, think of the argument as the string value, `"bar"`.

Even though Octave uses pass-by-value semantics for function arguments, values are not copied unnecessarily. For example,

```
x = rand (1000);
f (x);
```

does not actually force two 1000 by 1000 element matrices to exist *unless* the function `f` modifies the value of its argument. Then Octave must create a copy to avoid changing the value outside the scope of the function `f`, or attempting (and probably failing!) to modify the value of a constant or the value of a temporary result.

8.2.2 Recursion

With some restrictions[1], recursive function calls are allowed. A *recursive function* is one which calls itself, either directly or indirectly. For example, here is an inefficient[2] way to compute the factorial of a given integer:

```
function retval = fact (n)
  if (n > 0)
    retval = n * fact (n-1);
  else
    retval = 1;
  endif
endfunction
```

This function is recursive because it calls itself directly. It eventually terminates because each time it calls itself, it uses an argument that is one less than was used for the previous call. Once the argument is no longer greater than zero, it does not call itself, and the recursion ends.

The built-in variable `max_recursion_depth` specifies a limit to the recursion depth and prevents Octave from recursing infinitely.

val = `max_recursion_depth` () [Built-in Function]
old_val = `max_recursion_depth` (*new_val*) [Built-in Function]

Query or set the internal limit on the number of times a function may be called recursively. If the limit is exceeded, an error message is printed and control returns to the top level.

8.3 Arithmetic Operators

The following arithmetic operators are available, and work on scalars and matrices.

x + *y*
: Addition. If both operands are matrices, the number of rows and columns must both agree. If one operand is a scalar, its value is added to all the elements of the other operand.

x .+ *y*
: Element by element addition. This operator is equivalent to `+`.

x - *y*
: Subtraction. If both operands are matrices, the number of rows and columns of both must agree.

x .- *y*
: Element by element subtraction. This operator is equivalent to `-`.

x * *y*
: Matrix multiplication. The number of columns of *x* must agree with the number of rows of *y*.

x .* *y*
: Element by element multiplication. If both operands are matrices, the number of rows and columns must both agree.

---

[1] Some of Octave's functions are implemented in terms of functions that cannot be called recursively. For example, the ODE solver `lsode` is ultimately implemented in a Fortran subroutine that cannot be called recursively, so `lsode` should not be called either directly or indirectly from within the user-supplied function that `lsode` requires. Doing so will result in an error.

[2] It would be much better to use `prod (1:n)`, or `gamma (n+1)` instead, after first checking to ensure that the value `n` is actually a positive integer.

x / *y* Right division. This is conceptually equivalent to the expression

```
(inverse (y') * x')'
```

but it is computed without forming the inverse of *y'*.

If the system is not square, or if the coefficient matrix is singular, a minimum norm solution is computed.

x ./ *y* Element by element right division.

x \ *y* Left division. This is conceptually equivalent to the expression

```
inverse (x) * y
```

but it is computed without forming the inverse of *x*.

If the system is not square, or if the coefficient matrix is singular, a minimum norm solution is computed.

x .\ *y* Element by element left division. Each element of *y* is divided by each corresponding element of *x*.

x ^ *y*

x ** *y* Power operator. If *x* and *y* are both scalars, this operator returns *x* raised to the power *y*. If *x* is a scalar and *y* is a square matrix, the result is computed using an eigenvalue expansion. If *x* is a square matrix, the result is computed by repeated multiplication if *y* is an integer, and by an eigenvalue expansion if *y* is not an integer. An error results if both *x* and *y* are matrices.

The implementation of this operator needs to be improved.

x .^ *y*

x .** *y* Element by element power operator. If both operands are matrices, the number of rows and columns must both agree.

-*x* Negation.

+*x* Unary plus. This operator has no effect on the operand.

x' Complex conjugate transpose. For real arguments, this operator is the same as the transpose operator. For complex arguments, this operator is equivalent to the expression

```
conj (x.')
```

x.' Transpose.

Note that because Octave's element by element operators begin with a '.', there is a possible ambiguity for statements like

```
1./m
```

because the period could be interpreted either as part of the constant or as part of the operator. To resolve this conflict, Octave treats the expression as if you had typed

```
(1) ./ m
```

and not

```
(1.) / m
```

Although this is inconsistent with the normal behavior of Octave's lexer, which usually prefers to break the input into tokens by preferring the longest possible match at any given point, it is more useful in this case.

8.4 Comparison Operators

Comparison operators compare numeric values for relationships such as equality. They are written using *relational operators*.

All of Octave's comparison operators return a value of 1 if the comparison is true, or 0 if it is false. For matrix values, they all work on an element-by-element basis. For example,

```
[1, 2; 3, 4] == [1, 3; 2, 4]
     ⇒  1  0
        0  1
```

If one operand is a scalar and the other is a matrix, the scalar is compared to each element of the matrix in turn, and the result is the same size as the matrix.

`x < y` True if *x* is less than *y*.

`x <= y` True if *x* is less than or equal to *y*.

`x == y` True if *x* is equal to *y*.

`x >= y` True if *x* is greater than or equal to *y*.

`x > y` True if *x* is greater than *y*.

`x != y`
`x ~= y` True if *x* is not equal to *y*.

String comparisons may also be performed with the `strcmp` function, not with the comparison operators listed above. See Chapter 5 [Strings], page 55.

`isequal (x1, x2, ...)` [Function File]

Return true if all of *x1*, *x2*, ... are equal.

See also: [isequalwithequalnans], page 113.

`isequalwithequalnans (x1, x2, ...)` [Function File]

Assuming NaN == NaN, return true if all of *x1*, *x2*, ... are equal.

See also: [isequal], page 113.

8.5 Boolean Expressions

8.5.1 Element-by-element Boolean Operators

An *element-by-element boolean expression* is a combination of comparison expressions using the boolean operators "or" ('`|`'), "and" ('`&`'), and "not" ('`!`'), along with parentheses to control nesting. The truth of the boolean expression is computed by combining the truth values of the corresponding elements of the component expressions. A value is considered to be false if it is zero, and true otherwise.

Element-by-element boolean expressions can be used wherever comparison expressions can be used. They can be used in `if` and `while` statements. However, a matrix value used as the condition in an `if` or `while` statement is only true if *all* of its elements are nonzero.

Like comparison operations, each element of an element-by-element boolean expression also has a numeric value (1 if true, 0 if false) that comes into play if the result of the boolean expression is stored in a variable, or used in arithmetic.

Here are descriptions of the three element-by-element boolean operators.

boolean1 & *boolean2*
Elements of the result are true if both corresponding elements of *boolean1* and *boolean2* are true.

boolean1 | *boolean2*
Elements of the result are true if either of the corresponding elements of *boolean1* or *boolean2* is true.

! *boolean*
~ *boolean*
Each element of the result is true if the corresponding element of *boolean* is false.

For matrix operands, these operators work on an element-by-element basis. For example, the expression

```
[1, 0; 0, 1] & [1, 0; 2, 3]
```

returns a two by two identity matrix.

For the binary operators, the dimensions of the operands must conform if both are matrices. If one of the operands is a scalar and the other a matrix, the operator is applied to the scalar and each element of the matrix.

For the binary element-by-element boolean operators, both subexpressions *boolean1* and *boolean2* are evaluated before computing the result. This can make a difference when the expressions have side effects. For example, in the expression

```
a & b++
```

the value of the variable *b* is incremented even if the variable *a* is zero.

This behavior is necessary for the boolean operators to work as described for matrix-valued operands.

8.5.2 Short-circuit Boolean Operators

Combined with the implicit conversion to scalar values in `if` and `while` conditions, Octave's element-by-element boolean operators are often sufficient for performing most logical operations. However, it is sometimes desirable to stop evaluating a boolean expression as soon as the overall truth value can be determined. Octave's *short-circuit* boolean operators work this way.

boolean1 && *boolean2*
The expression *boolean1* is evaluated and converted to a scalar using the equivalent of the operation `all (boolean1(:))`. If it is false, the result of the overall expression is 0. If it is true, the expression *boolean2* is evaluated and converted to a scalar using the equivalent of the operation `all (boolean1(:))`. If it is true, the result of the overall expression is 1. Otherwise, the result of the overall expression is 0.

Warning: there is one exception to the rule of evaluating `all (boolean1(:))`, which is when `boolean1` is the empty matrix. The truth value of an empty matrix is always `false` so `[] && true` evaluates to `false` even though `all ([])` is `true`.

boolean1 || *boolean2*
: The expression *boolean1* is evaluated and converted to a scalar using the equivalent of the operation `all (boolean1(:))`. If it is true, the result of the overall expression is 1. If it is false, the expression *boolean2* is evaluated and converted to a scalar using the equivalent of the operation `all (boolean1(:))`. If it is true, the result of the overall expression is 1. Otherwise, the result of the overall expression is 0.

  Warning: the truth value of an empty matrix is always `false`, see the previous list item for details.

The fact that both operands may not be evaluated before determining the overall truth value of the expression can be important. For example, in the expression

```
a && b++
```

the value of the variable *b* is only incremented if the variable *a* is nonzero.

This can be used to write somewhat more concise code. For example, it is possible write

```
function f (a, b, c)
  if (nargin > 2 && ischar (c))
    ...
```

instead of having to use two `if` statements to avoid attempting to evaluate an argument that doesn't exist. For example, without the short-circuit feature, it would be necessary to write

```
function f (a, b, c)
  if (nargin > 2)
    if (ischar (c))
      ...
```

Writing

```
function f (a, b, c)
  if (nargin > 2 & ischar (c))
    ...
```

would result in an error if `f` were called with one or two arguments because Octave would be forced to try to evaluate both of the operands for the operator '`&`'.

8.6 Assignment Expressions

An *assignment* is an expression that stores a new value into a variable. For example, the following expression assigns the value 1 to the variable `z`:

```
z = 1
```

After this expression is executed, the variable `z` has the value 1. Whatever old value `z` had before the assignment is forgotten. The '`=`' sign is called an *assignment operator*.

Assignments can store string values also. For example, the following expression would store the value `"this food is good"` in the variable `message`:

```
thing = "food"
predicate = "good"
message = [ "this " , thing , " is " , predicate ]
```

(This also illustrates concatenation of strings.)

Most operators (addition, concatenation, and so on) have no effect except to compute a value. If you ignore the value, you might as well not use the operator. An assignment operator is different. It does produce a value, but even if you ignore the value, the assignment still makes itself felt through the alteration of the variable. We call this a *side effect*.

The left-hand operand of an assignment need not be a variable (see Chapter 7 [Variables], page 97). It can also be an element of a matrix (see Section 8.1 [Index Expressions], page 107) or a list of return values (see Section 8.2 [Calling Functions], page 109). These are all called *lvalues*, which means they can appear on the left-hand side of an assignment operator. The right-hand operand may be any expression. It produces the new value which the assignment stores in the specified variable, matrix element, or list of return values.

It is important to note that variables do *not* have permanent types. The type of a variable is simply the type of whatever value it happens to hold at the moment. In the following program fragment, the variable `foo` has a numeric value at first, and a string value later on:

```
octave:13> foo = 1
foo = 1
octave:13> foo = "bar"
foo = bar
```

When the second assignment gives `foo` a string value, the fact that it previously had a numeric value is forgotten.

Assignment of a scalar to an indexed matrix sets all of the elements that are referenced by the indices to the scalar value. For example, if `a` is a matrix with at least two columns,

```
a(:, 2) = 5
```

sets all the elements in the second column of `a` to 5.

Assigning an empty matrix '`[]`' works in most cases to allow you to delete rows or columns of matrices and vectors. See Section 4.1.1 [Empty Matrices], page 42. For example, given a 4 by 5 matrix A, the assignment

```
A (3, :) = []
```

deletes the third row of A, and the assignment

```
A (:, 1:2:5) = []
```

deletes the first, third, and fifth columns.

An assignment is an expression, so it has a value. Thus, `z = 1` as an expression has the value 1. One consequence of this is that you can write multiple assignments together:

```
x = y = z = 0
```

stores the value 0 in all three variables. It does this because the value of `z = 0`, which is 0, is stored into `y`, and then the value of `y = z = 0`, which is 0, is stored into `x`.

This is also true of assignments to lists of values, so the following is a valid expression

```
[a, b, c] = [u, s, v] = svd (a)
```

that is exactly equivalent to

```
[u, s, v] = svd (a)
a = u
b = s
c = v
```

In expressions like this, the number of values in each part of the expression need not match. For example, the expression

```
[a, b] = [u, s, v] = svd (a)
```

is equivalent to

```
[u, s, v] = svd (a)
a = u
b = s
```

The number of values on the left side of the expression can, however, not exceed the number of values on the right side. For example, the following will produce an error.

```
[a, b, c, d] = [u, s, v] = svd (a);
⊣ error: element number 4 undefined in return list
```

A very common programming pattern is to increment an existing variable with a given value, like this

```
a = a + 2;
```

This can be written in a clearer and more condensed form using the `+=` operator

```
a += 2;
```

Similar operators also exist for subtraction (`-=`), multiplication (`*=`), and division (`/=`). An expression of the form

```
expr1 op= expr2
```

is evaluated as

```
expr1 = (expr1) op (expr2)
```

where *op* can be either `+`, `-`, `*`, or `/`. So, the expression

```
a *= b+1
```

is evaluated as

```
a = a * (b+1)
```

and *not*

```
a = a * b + 1
```

You can use an assignment anywhere an expression is called for. For example, it is valid to write `x != (y = 1)` to set `y` to 1 and then test whether `x` equals 1. But this style tends to make programs hard to read. Except in a one-shot program, you should rewrite it to get rid of such nesting of assignments. This is never very hard.

8.7 Increment Operators

Increment operators increase or decrease the value of a variable by 1. The operator to increment a variable is written as '`++`'. It may be used to increment a variable either before or after taking its value.

For example, to pre-increment the variable *x*, you would write `++x`. This would add one to *x* and then return the new value of *x* as the result of the expression. It is exactly the same as the expression `x = x + 1`.

To post-increment a variable *x*, you would write `x++`. This adds one to the variable *x*, but returns the value that *x* had prior to incrementing it. For example, if *x* is equal to 2, the result of the expression `x++` is 2, and the new value of *x* is 3.

For matrix and vector arguments, the increment and decrement operators work on each element of the operand.

Here is a list of all the increment and decrement expressions.

`++x`
: This expression increments the variable *x*. The value of the expression is the *new* value of *x*. It is equivalent to the expression `x = x + 1`.

`--x`
: This expression decrements the variable *x*. The value of the expression is the *new* value of *x*. It is equivalent to the expression `x = x - 1`.

`x++`
: This expression causes the variable *x* to be incremented. The value of the expression is the *old* value of *x*.

`x--`
: This expression causes the variable *x* to be decremented. The value of the expression is the *old* value of *x*.

8.8 Operator Precedence

Operator precedence determines how operators are grouped, when different operators appear close by in one expression. For example, '`*`' has higher precedence than '`+`'. Thus, the expression `a + b * c` means to multiply `b` and `c`, and then add `a` to the product (i.e., `a + (b * c)`).

You can overrule the precedence of the operators by using parentheses. You can think of the precedence rules as saying where the parentheses are assumed if you do not write parentheses yourself. In fact, it is wise to use parentheses whenever you have an unusual combination of operators, because other people who read the program may not remember what the precedence is in this case. You might forget as well, and then you too could make a mistake. Explicit parentheses will help prevent any such mistake.

When operators of equal precedence are used together, the leftmost operator groups first, except for the assignment and exponentiation operators, which group in the opposite order. Thus, the expression `a - b + c` groups as `(a - b) + c`, but the expression `a = b = c` groups as `a = (b = c)`.

The precedence of prefix unary operators is important when another operator follows the operand. For example, `-x^2` means `-(x^2)`, because '`-`' has lower precedence than '`^`'.

Here is a table of the operators in Octave, in order of increasing precedence.

`statement separators`
: '`;`', '`,`'.

`assignment`
: '`=`', '`+=`', '`-=`', '`*=`','`/=`'. This operator groups right to left.

`logical "or" and "and"`
: '`||`', '`&&`'.

`element-wise "or" and "and"`
: '`|`', '`&`'.

`relational`
: '`<`', '`<=`', '`==`', '`>=`', '`>`', '`!=`', '`~=`'.

`colon`
: '`:`'.

`add, subtract`
: '+', '-'.

`multiply, divide`
: '*', '/', '\', '.\', '.*', './'.

`transpose`
: ''', '.''

`unary plus, minus, increment, decrement, and ``not''`
: '+', '-', '++', '--', '!', '~'.

`exponentiation`
: '^', '**', '.^', '.**'.

9 Evaluation

Normally, you evaluate expressions simply by typing them at the Octave prompt, or by asking Octave to interpret commands that you have saved in a file.

Sometimes, you may find it necessary to evaluate an expression that has been computed and stored in a string, which is exactly what the `eval` function lets you do.

`eval (`*`try`*`, `*`catch`*`)` [Built-in Function]

Parse the string *try* and evaluate it as if it were an Octave program. If that fails, evaluate the optional string *catch*. The string *try* is evaluated in the current context, so any results remain available after `eval` returns.

The following example makes the variable *a* with the approximate value 3.1416 available.

```
eval("a = acos(-1);");
```

If an error occurs during the evaluation of *try* the *catch* string is evaluated, as the following example shows:

```
eval ('error ("This is a bad example");',
      'printf ("This error occurred:\n%s\n", lasterr ());');
     ⊣ This error occurred:
       This is a bad example
```

9.1 Calling a Function by its Name

The `feval` function allows you to call a function from a string containing its name. This is useful when writing a function that needs to call user-supplied functions. The `feval` function takes the name of the function to call as its first argument, and the remaining arguments are given to the function.

The following example is a simple-minded function using `feval` that finds the root of a user-supplied function of one variable using Newton's method.

```
function result = newtroot (fname, x)

# usage: newtroot (fname, x)
#
#   fname : a string naming a function f(x).
#   x     : initial guess

  delta = tol = sqrt (eps);
  maxit = 200;
  fx = feval (fname, x);
  for i = 1:maxit
    if (abs (fx) < tol)
      result = x;
      return;
    else
      fx_new = feval (fname, x + delta);
      deriv = (fx_new - fx) / delta;
```

```
      x = x - fx / deriv;
      fx = fx_new;
    endif
  endfor

  result = x;

endfunction
```

Note that this is only meant to be an example of calling user-supplied functions and should not be taken too seriously. In addition to using a more robust algorithm, any serious code would check the number and type of all the arguments, ensure that the supplied function really was a function, etc. See Section 4.8 [Predicates for Numeric Objects], page 52, for example, for a list of predicates for numeric objects, and see Section 7.3 [Status of Variables], page 102, for a description of the `exist` function.

`feval` (*name*, ...) [Built-in Function]

Evaluate the function named *name*. Any arguments after the first are passed on to the named function. For example,

```
feval ("acos", -1)
     ⇒ 3.1416
```

calls the function `acos` with the argument '`-1`'.

The function `feval` is necessary in order to be able to write functions that call user-supplied functions, because Octave does not have a way to declare a pointer to a function (like C) or to declare a special kind of variable that can be used to hold the name of a function (like `EXTERNAL` in Fortran). Instead, you must refer to functions by name, and use `feval` to call them.

A similar function `run` exists for calling user script files, that are not necessarily on the user path

`run` (*f*) [Function File]
`run` *f* [Command]

Run scripts in the current workspace that are not necessarily on the path. If *f* is the script to run, including its path, then `run` change the directory to the directory where *f* is found. `run` then executes the script, and returns to the original directory.

See also: [system], page 533.

9.2 Evaluation in a Different Context

Before you evaluate an expression you need to substitute the values of the variables used in the expression. These are stored in the symbol table. Whenever the interpreter starts a new function it saves the current symbol table and creates a new one, initializing it with the list of function parameters and a couple of predefined variables such as `nargin`. Expressions inside the function use the new symbol table.

Sometimes you want to write a function so that when you call it, it modifies variables in your own context. This allows you to use a pass-by-name style of function, which is similar to using a pointer in programming languages such as C.

Consider how you might write `save` and `load` as m-files. For example,

```
function create_data
  x = linspace (0, 10, 10);
  y = sin (x);
  save mydata x y
endfunction
```

With `evalin`, you could write `save` as follows:

```
function save (file, name1, name2)
  f = open_save_file (file);
  save_var(f, name1, evalin ("caller", name1));
  save_var(f, name2, evalin ("caller", name2));
endfunction
```

Here, '`caller`' is the `create_data` function and `name1` is the string `"x"`, which evaluates simply as the value of `x`.

You later want to load the values back from `mydata` in a different context:

```
function process_data
  load mydata
  ... do work ...
endfunction
```

With `assignin`, you could write `load` as follows:

```
function load (file)
  f = open_load_file (file);
  [name, val] = load_var (f);
  assignin ("caller", name, val);
  [name, val] = load_var (f);
  assignin ("caller", name, val);
endfunction
```

Here, '`caller`' is the `process_data` function.

You can set and use variables at the command prompt using the context '`base`' rather than '`caller`'.

These functions are rarely used in practice. One example is the `fail ('code', 'pattern')` function which evaluates '`code`' in the caller's context and checks that the error message it produces matches the given pattern. Other examples such as `save` and `load` are written in C++ where all Octave variables are in the '`caller`' context and `evalin` is not needed.

`evalin (`*`context`*`, `*`try`*`, `*`catch`*`)` [Built-in Function]

Like `eval`, except that the expressions are evaluated in the context *context*, which may be either `"caller"` or `"base"`.

`assignin (`*`context`*`, `*`varname`*`, `*`value`*`)` [Built-in Function]

Assign *value* to *varname* in context *context*, which may be either `"base"` or `"caller"`.

10 Statements

Statements may be a simple constant expression or a complicated list of nested loops and conditional statements.

Control statements such as `if`, `while`, and so on control the flow of execution in Octave programs. All the control statements start with special keywords such as `if` and `while`, to distinguish them from simple expressions. Many control statements contain other statements; for example, the `if` statement contains another statement which may or may not be executed.

Each control statement has a corresponding *end* statement that marks the end of the control statement. For example, the keyword `endif` marks the end of an `if` statement, and `endwhile` marks the end of a `while` statement. You can use the keyword `end` anywhere a more specific end keyword is expected, but using the more specific keywords is preferred because if you use them, Octave is able to provide better diagnostics for mismatched or missing end tokens.

The list of statements contained between keywords like `if` or `while` and the corresponding end statement is called the *body* of a control statement.

10.1 The `if` Statement

The `if` statement is Octave's decision-making statement. There are three basic forms of an `if` statement. In its simplest form, it looks like this:

```
if (condition)
  then-body
endif
```

condition is an expression that controls what the rest of the statement will do. The *then-body* is executed only if *condition* is true.

The condition in an `if` statement is considered true if its value is non-zero, and false if its value is zero. If the value of the conditional expression in an `if` statement is a vector or a matrix, it is considered true only if it is non-empty and *all* of the elements are non-zero.

The second form of an if statement looks like this:

```
if (condition)
  then-body
else
  else-body
endif
```

If *condition* is true, *then-body* is executed; otherwise, *else-body* is executed.

Here is an example:

```
if (rem (x, 2) == 0)
  printf ("x is even\n");
else
  printf ("x is odd\n");
endif
```

In this example, if the expression `rem (x, 2) == 0` is true (that is, the value of `x` is divisible by 2), then the first `printf` statement is evaluated, otherwise the second `printf` statement is evaluated.

The third and most general form of the `if` statement allows multiple decisions to be combined in a single statement. It looks like this:

```
if (condition)
  then-body
elseif (condition)
  elseif-body
else
  else-body
endif
```

Any number of `elseif` clauses may appear. Each condition is tested in turn, and if one is found to be true, its corresponding *body* is executed. If none of the conditions are true and the `else` clause is present, its body is executed. Only one `else` clause may appear, and it must be the last part of the statement.

In the following example, if the first condition is true (that is, the value of `x` is divisible by 2), then the first `printf` statement is executed. If it is false, then the second condition is tested, and if it is true (that is, the value of `x` is divisible by 3), then the second `printf` statement is executed. Otherwise, the third `printf` statement is performed.

```
if (rem (x, 2) == 0)
  printf ("x is even\n");
elseif (rem (x, 3) == 0)
  printf ("x is odd and divisible by 3\n");
else
  printf ("x is odd\n");
endif
```

Note that the `elseif` keyword must not be spelled `else if`, as is allowed in Fortran. If it is, the space between the `else` and `if` will tell Octave to treat this as a new `if` statement within another `if` statement's `else` clause. For example, if you write

```
if (c1)
  body-1
else if (c2)
  body-2
endif
```

Octave will expect additional input to complete the first `if` statement. If you are using Octave interactively, it will continue to prompt you for additional input. If Octave is reading this input from a file, it may complain about missing or mismatched `end` statements, or, if you have not used the more specific `end` statements (`endif`, `endfor`, etc.), it may simply produce incorrect results, without producing any warning messages.

It is much easier to see the error if we rewrite the statements above like this,

```
if (c1)
  body-1
else
  if (c2)
    body-2
  endif
```

using the indentation to show how Octave groups the statements. See Chapter 11 [Functions and Scripts], page 137.

10.2 The switch Statement

It is very common to take different actions depending on the value of one variable. This is possible using the `if` statement in the following way

```
if (X == 1)
  do_something ();
elseif (X == 2)
  do_something_else ();
else
  do_something_completely_different ();
endif
```

This kind of code can however be very cumbersome to both write and maintain. To overcome this problem Octave supports the `switch` statement. Using this statement, the above example becomes

```
switch (X)
  case 1
    do_something ();
  case 2
    do_something_else ();
  otherwise
    do_something_completely_different ();
endswitch
```

This code makes the repetitive structure of the problem more explicit, making the code easier to read, and hence maintain. Also, if the variable `X` should change its name, only one line would need changing compared to one line per case when `if` statements are used.

The general form of the `switch` statement is

```
switch expression
  case label
    command_list
  case label
    command_list
  ...

  otherwise
    command_list
endswitch
```

where *label* can be any expression. However, duplicate *label* values are not detected, and only the *command_list* corresponding to the first match will be executed. For the `switch` statement to be meaningful at least one `case label command_list` clause must be present, while the `otherwise command_list` clause is optional.

If *label* is a cell array the corresponding *command_list* is executed if *any* of the elements of the cell array match *expression*. As an example, the following program will print '`Variable is either 6 or 7`'.

```
A = 7;
switch A
  case { 6, 7 }
    printf ("variable is either 6 or 7\n");
  otherwise
    printf ("variable is neither 6 nor 7\n");
endswitch
```

As with all other specific `end` keywords, `endswitch` may be replaced by `end`, but you can get better diagnostics if you use the specific forms.

One advantage of using the `switch` statement compared to using `if` statements is that the *label*s can be strings. If an `if` statement is used it is *not* possible to write

```
if (X == "a string") # This is NOT valid
```

since a character-to-character comparison between `X` and the string will be made instead of evaluating if the strings are equal. This special-case is handled by the `switch` statement, and it is possible to write programs that look like this

```
switch (X)
  case "a string"
    do_something
  ...
endswitch
```

10.2.1 Notes for the C programmer

The `switch` statement is also available in the widely used C programming language. There are, however, some differences between the statement in Octave and C

- Cases are exclusive, so they don't 'fall through' as do the cases in the `switch` statement of the C language.
- The *command_list* elements are not optional. Making the list optional would have meant requiring a separator between the label and the command list. Otherwise, things like

  ```
  switch (foo)
    case (1) -2
    ...
  ```

  would produce surprising results, as would

  ```
  switch (foo)
    case (1)
    case (2)
      doit ();
    ...
  ```

particularly for C programmers. If `doit()` should be executed if *foo* is either `1` or `2`, the above code should be written with a cell array like this

```
switch (foo)
  case { 1, 2 }
    doit ();
  ...
```

10.3 The `while` Statement

In programming, a *loop* means a part of a program that is (or at least can be) executed two or more times in succession.

The `while` statement is the simplest looping statement in Octave. It repeatedly executes a statement as long as a condition is true. As with the condition in an `if` statement, the condition in a `while` statement is considered true if its value is non-zero, and false if its value is zero. If the value of the conditional expression in a `while` statement is a vector or a matrix, it is considered true only if it is non-empty and *all* of the elements are non-zero.

Octave's `while` statement looks like this:

```
while (condition)
  body
endwhile
```

Here *body* is a statement or list of statements that we call the *body* of the loop, and *condition* is an expression that controls how long the loop keeps running.

The first thing the `while` statement does is test *condition*. If *condition* is true, it executes the statement *body*. After *body* has been executed, *condition* is tested again, and if it is still true, *body* is executed again. This process repeats until *condition* is no longer true. If *condition* is initially false, the body of the loop is never executed.

This example creates a variable `fib` that contains the first ten elements of the Fibonacci sequence.

```
fib = ones (1, 10);
i = 3;
while (i <= 10)
  fib (i) = fib (i-1) + fib (i-2);
  i++;
endwhile
```

Here the body of the loop contains two statements.

The loop works like this: first, the value of `i` is set to 3. Then, the `while` tests whether `i` is less than or equal to 10. This is the case when `i` equals 3, so the value of the `i`-th element of `fib` is set to the sum of the previous two values in the sequence. Then the `i++` increments the value of `i` and the loop repeats. The loop terminates when `i` reaches 11.

A newline is not required between the condition and the body; but using one makes the program clearer unless the body is very simple.

10.4 The do-until Statement

The `do-until` statement is similar to the `while` statement, except that it repeatedly executes a statement until a condition becomes true, and the test of the condition is at the end of the loop, so the body of the loop is always executed at least once. As with the condition in an `if` statement, the condition in a `do-until` statement is considered true if its value is non-zero, and false if its value is zero. If the value of the conditional expression in a `do-until` statement is a vector or a matrix, it is considered true only if it is non-empty and *all* of the elements are non-zero.

Octave's `do-until` statement looks like this:

```
do
  body
until (condition)
```

Here *body* is a statement or list of statements that we call the *body* of the loop, and *condition* is an expression that controls how long the loop keeps running.

This example creates a variable `fib` that contains the first ten elements of the Fibonacci sequence.

```
fib = ones (1, 10);
i = 2;
do
  i++;
  fib (i) = fib (i-1) + fib (i-2);
until (i == 10)
```

A newline is not required between the `do` keyword and the body; but using one makes the program clearer unless the body is very simple.

10.5 The for Statement

The `for` statement makes it more convenient to count iterations of a loop. The general form of the `for` statement looks like this:

```
for var = expression
  body
endfor
```

where *body* stands for any statement or list of statements, *expression* is any valid expression, and *var* may take several forms. Usually it is a simple variable name or an indexed variable. If the value of *expression* is a structure, *var* may also be a vector with two elements. See Section 10.5.1 [Looping Over Structure Elements], page 131, below.

The assignment expression in the `for` statement works a bit differently than Octave's normal assignment statement. Instead of assigning the complete result of the expression, it assigns each column of the expression to *var* in turn. If *expression* is a range, a row vector, or a scalar, the value of *var* will be a scalar each time the loop body is executed. If *var* is a column vector or a matrix, *var* will be a column vector each time the loop body is executed.

The following example shows another way to create a vector containing the first ten elements of the Fibonacci sequence, this time using the `for` statement:

```
fib = ones (1, 10);
for i = 3:10
  fib (i) = fib (i-1) + fib (i-2);
endfor
```

This code works by first evaluating the expression `3:10`, to produce a range of values from 3 to 10 inclusive. Then the variable `i` is assigned the first element of the range and the body of the loop is executed once. When the end of the loop body is reached, the next value in the range is assigned to the variable `i`, and the loop body is executed again. This process continues until there are no more elements to assign.

Within Octave is it also possible to iterate over matrices or cell arrays using the `for` statement. For example consider

```
disp("Loop over a matrix")
for i = [1,3;2,4]
  i
endfor
disp("Loop over a cell array")
for i = {1,"two";"three",4}
  i
endfor
```

In this case the variable `i` takes on the value of the columns of the matrix or cell matrix. So the first loop iterates twice, producing two column vectors `[1;2]`, followed by `[3;4]`, and likewise for the loop over the cell array. This can be extended to loops over multidimensional arrays. For example

```
a = [1,3;2,4]; b = cat(3, a, 2*a);
for i = c
  i
endfor
```

In the above case, the multidimensional matrix *c* is reshaped to a two-dimensional matrix as `reshape (c, rows(c), prod(size(c)(2:end)))` and then the same behavior as a loop over a two dimensional matrix is produced.

Although it is possible to rewrite all `for` loops as `while` loops, the Octave language has both statements because often a `for` loop is both less work to type and more natural to think of. Counting the number of iterations is very common in loops and it can be easier to think of this counting as part of looping rather than as something to do inside the loop.

10.5.1 Looping Over Structure Elements

A special form of the `for` statement allows you to loop over all the elements of a structure:

```
for [ val, key ] = expression
  body
endfor
```

In this form of the `for` statement, the value of *expression* must be a structure. If it is, *key* and *val* are set to the name of the element and the corresponding value in turn, until there are no more elements. For example,

```
x.a = 1
x.b = [1, 2; 3, 4]
x.c = "string"
for [val, key] = x
  key
  val
endfor

     ⊣ key = a
     ⊣ val = 1
     ⊣ key = b
     ⊣ val =
     ⊣
     ⊣   1  2
     ⊣   3  4
     ⊣
     ⊣ key = c
     ⊣ val = string
```

The elements are not accessed in any particular order. If you need to cycle through the list in a particular way, you will have to use the function `fieldnames` and sort the list yourself.

The *key* variable may also be omitted. If it is, the brackets are also optional. This is useful for cycling through the values of all the structure elements when the names of the elements do not need to be known.

10.6 The `break` Statement

The `break` statement jumps out of the innermost `for` or `while` loop that encloses it. The `break` statement may only be used within the body of a loop. The following example finds the smallest divisor of a given integer, and also identifies prime numbers:

```
num = 103;
div = 2;
while (div*div <= num)
  if (rem (num, div) == 0)
    break;
  endif
  div++;
endwhile
if (rem (num, div) == 0)
  printf ("Smallest divisor of %d is %d\n", num, div)
else
  printf ("%d is prime\n", num);
endif
```

When the remainder is zero in the first `while` statement, Octave immediately *breaks out* of the loop. This means that Octave proceeds immediately to the statement following

the loop and continues processing. (This is very different from the `exit` statement which stops the entire Octave program.)

Here is another program equivalent to the previous one. It illustrates how the *condition* of a `while` statement could just as well be replaced with a `break` inside an `if`:

```
num = 103;
div = 2;
while (1)
  if (rem (num, div) == 0)
    printf ("Smallest divisor of %d is %d\n", num, div);
    break;
  endif
  div++;
  if (div*div > num)
    printf ("%d is prime\n", num);
    break;
  endif
endwhile
```

10.7 The continue Statement

The `continue` statement, like `break`, is used only inside `for` or `while` loops. It skips over the rest of the loop body, causing the next cycle around the loop to begin immediately. Contrast this with `break`, which jumps out of the loop altogether. Here is an example:

```
# print elements of a vector of random
# integers that are even.

# first, create a row vector of 10 random
# integers with values between 0 and 100:

vec = round (rand (1, 10) * 100);

# print what we're interested in:

for x = vec
  if (rem (x, 2) != 0)
    continue;
  endif
  printf ("%d\n", x);
endfor
```

If one of the elements of *vec* is an odd number, this example skips the print statement for that element, and continues back to the first statement in the loop.

This is not a practical example of the `continue` statement, but it should give you a clear understanding of how it works. Normally, one would probably write the loop like this:

```
for x = vec
  if (rem (x, 2) == 0)
    printf ("%d\n", x);
  endif
endfor
```

10.8 The unwind_protect Statement

Octave supports a limited form of exception handling modelled after the unwind-protect form of Lisp.

The general form of an `unwind_protect` block looks like this:

```
unwind_protect
  body
unwind_protect_cleanup
  cleanup
end_unwind_protect
```

where *body* and *cleanup* are both optional and may contain any Octave expressions or commands. The statements in *cleanup* are guaranteed to be executed regardless of how control exits *body*.

This is useful to protect temporary changes to global variables from possible errors. For example, the following code will always restore the original value of the global variable `frobnosticate` even if an error occurs in the first part of the `unwind_protect` block.

```
save_frobnosticate = frobnosticate;
unwind_protect
  frobnosticate = true;
  ...
unwind_protect_cleanup
  frobnosticate = save_frobnosticate;
end_unwind_protect
```

Without `unwind_protect`, the value of *frobnosticate* would not be restored if an error occurs while evaluating the first part of the `unwind_protect` block because evaluation would stop at the point of the error and the statement to restore the value would not be executed.

10.9 The try Statement

In addition to unwind_protect, Octave supports another limited form of exception handling.

The general form of a `try` block looks like this:

```
try
  body
catch
  cleanup
end_try_catch
```

where *body* and *cleanup* are both optional and may contain any Octave expressions or commands. The statements in *cleanup* are only executed if an error occurs in *body*.

No warnings or error messages are printed while *body* is executing. If an error does occur during the execution of *body*, *cleanup* can use the function `lasterr` to access the

text of the message that would have been printed. This is the same as `eval (try, catch)` but it is more efficient since the commands do not need to be parsed each time the *try* and *catch* statements are evaluated. See Chapter 12 [Errors and Warnings], page 161, for more information about the `lasterr` function.

10.10 Continuation Lines

In the Octave language, most statements end with a newline character and you must tell Octave to ignore the newline character in order to continue a statement from one line to the next. Lines that end with the characters `...` or `\` are joined with the following line before they are divided into tokens by Octave's parser. For example, the lines

```
x = long_variable_name ...
    + longer_variable_name \
    - 42
```

form a single statement. The backslash character on the second line above is interpreted as a continuation character, *not* as a division operator.

For continuation lines that do not occur inside string constants, whitespace and comments may appear between the continuation marker and the newline character. For example, the statement

```
x = long_variable_name ...     # comment one
    + longer_variable_name \   # comment two
    - 42                       # last comment
```

is equivalent to the one shown above. Inside string constants, the continuation marker must appear at the end of the line just before the newline character.

Input that occurs inside parentheses can be continued to the next line without having to use a continuation marker. For example, it is possible to write statements like

```
if (fine_dining_destination == on_a_boat
    || fine_dining_destination == on_a_train)
  seuss (i, will, not, eat, them, sam, i, am, i,
         will, not, eat, green, eggs, and, ham);
endif
```

without having to add to the clutter with continuation markers.

11 Functions and Scripts

Complicated Octave programs can often be simplified by defining functions. Functions can be defined directly on the command line during interactive Octave sessions, or in external files, and can be called just like built-in functions.

11.1 Defining Functions

In its simplest form, the definition of a function named *name* looks like this:

```
function name
  body
endfunction
```

A valid function name is like a valid variable name: a sequence of letters, digits and underscores, not starting with a digit. Functions share the same pool of names as variables.

The function *body* consists of Octave statements. It is the most important part of the definition, because it says what the function should actually *do*.

For example, here is a function that, when executed, will ring the bell on your terminal (assuming that it is possible to do so):

```
function wakeup
  printf ("\a");
endfunction
```

The `printf` statement (see Chapter 14 [Input and Output], page 175) simply tells Octave to print the string `"\a"`. The special character '`\a`' stands for the alert character (ASCII 7). See Chapter 5 [Strings], page 55.

Once this function is defined, you can ask Octave to evaluate it by typing the name of the function.

Normally, you will want to pass some information to the functions you define. The syntax for passing parameters to a function in Octave is

```
function name (arg-list)
  body
endfunction
```

where *arg-list* is a comma-separated list of the function's arguments. When the function is called, the argument names are used to hold the argument values given in the call. The list of arguments may be empty, in which case this form is equivalent to the one shown above.

To print a message along with ringing the bell, you might modify the `wakeup` to look like this:

```
function wakeup (message)
  printf ("\a%s\n", message);
endfunction
```

Calling this function using a statement like this

```
wakeup ("Rise and shine!");
```

will cause Octave to ring your terminal's bell and print the message '`Rise and shine!`', followed by a newline character (the '`\n`' in the first argument to the `printf` statement).

In most cases, you will also want to get some information back from the functions you define. Here is the syntax for writing a function that returns a single value:

```
function ret-var = name (arg-list)
  body
endfunction
```

The symbol *ret-var* is the name of the variable that will hold the value to be returned by the function. This variable must be defined before the end of the function body in order for the function to return a value.

Variables used in the body of a function are local to the function. Variables named in *arg-list* and *ret-var* are also local to the function. See Section 7.1 [Global Variables], page 99, for information about how to access global variables inside a function.

For example, here is a function that computes the average of the elements of a vector:

```
function retval = avg (v)
  retval = sum (v) / length (v);
endfunction
```

If we had written `avg` like this instead,

```
function retval = avg (v)
  if (isvector (v))
    retval = sum (v) / length (v);
  endif
endfunction
```

and then called the function with a matrix instead of a vector as the argument, Octave would have printed an error message like this:

```
error: value on right hand side of assignment is undefined
```

because the body of the `if` statement was never executed, and `retval` was never defined. To prevent obscure errors like this, it is a good idea to always make sure that the return variables will always have values, and to produce meaningful error messages when problems are encountered. For example, `avg` could have been written like this:

```
function retval = avg (v)
  retval = 0;
  if (isvector (v))
    retval = sum (v) / length (v);
  else
    error ("avg: expecting vector argument");
  endif
endfunction
```

There is still one additional problem with this function. What if it is called without an argument? Without additional error checking, Octave will probably print an error message that won't really help you track down the source of the error. To allow you to catch errors like this, Octave provides each function with an automatic variable called `nargin`. Each time a function is called, `nargin` is automatically initialized to the number of arguments that have actually been passed to the function. For example, we might rewrite the `avg` function like this:

```
function retval = avg (v)
  retval = 0;
  if (nargin != 1)
    usage ("avg (vector)");
  endif
  if (isvector (v))
    retval = sum (v) / length (v);
  else
    error ("avg: expecting vector argument");
  endif
endfunction
```

Although Octave does not automatically report an error if you call a function with more arguments than expected, doing so probably indicates that something is wrong. Octave also does not automatically report an error if a function is called with too few arguments, but any attempt to use a variable that has not been given a value will result in an error. To avoid such problems and to provide useful messages, we check for both possibilities and issue our own error message.

nargin () [Built-in Function]
nargin (*fcn_name*) [Built-in Function]

Within a function, return the number of arguments passed to the function. At the top level, return the number of command line arguments passed to Octave. If called with the optional argument *fcn_name*, return the maximum number of arguments the named function can accept, or -1 if the function accepts a variable number of arguments.

See also: [nargout], page 140, [varargin], page 141, [varargout], page 141.

inputname (*n*) [Function File]

Return the text defining *n*-th input to the function.

val = **silent_functions** () [Built-in Function]
old_val = **silent_functions** (*new_val*) [Built-in Function]

Query or set the internal variable that controls whether internal output from a function is suppressed. If this option is disabled, Octave will display the results produced by evaluating expressions within a function body that are not terminated with a semicolon.

11.2 Multiple Return Values

Unlike many other computer languages, Octave allows you to define functions that return more than one value. The syntax for defining functions that return multiple values is

```
function [ret-list] = name (arg-list)
  body
endfunction
```

where *name*, *arg-list*, and *body* have the same meaning as before, and *ret-list* is a comma-separated list of variable names that will hold the values returned from the function. The list of return values must have at least one element. If *ret-list* has only one element, this form of the `function` statement is equivalent to the form described in the previous section.

Here is an example of a function that returns two values, the maximum element of a vector and the index of its first occurrence in the vector.

```
function [max, idx] = vmax (v)
  idx = 1;
  max = v (idx);
  for i = 2:length (v)
    if (v (i) > max)
      max = v (i);
      idx = i;
    endif
  endfor
endfunction
```

In this particular case, the two values could have been returned as elements of a single array, but that is not always possible or convenient. The values to be returned may not have compatible dimensions, and it is often desirable to give the individual return values distinct names.

In addition to setting `nargin` each time a function is called, Octave also automatically initializes `nargout` to the number of values that are expected to be returned. This allows you to write functions that behave differently depending on the number of values that the user of the function has requested. The implicit assignment to the built-in variable `ans` does not figure in the count of output arguments, so the value of `nargout` may be zero.

The `svd` and `lu` functions are examples of built-in functions that behave differently depending on the value of `nargout`.

It is possible to write functions that only set some return values. For example, calling the function

```
function [x, y, z] = f ()
  x = 1;
  z = 2;
endfunction
```

as

```
[a, b, c] = f ()
```

produces:

```
a = 1

b = [](0x0)

c = 2
```

along with a warning.

`nargout ()` [Built-in Function]
`nargout (`*`fcn_name`*`)` [Built-in Function]

Within a function, return the number of values the caller expects to receive. If called with the optional argument *fcn_name*, return the maximum number of values the named function can produce, or -1 if the function can produce a variable number of values.

For example,

```
f ()
```

will cause `nargout` to return 0 inside the function `f` and

```
[s, t] = f ()
```

will cause `nargout` to return 2 inside the function `f`.

At the top level, `nargout` is undefined.

See also: [nargin], page 139, [varargin], page 141, [varargout], page 141.

msgstr = nargchk (*minargs*, *maxargs*, *nargs*) [Function File]
msgstr = nargchk (*minargs*, *maxargs*, *nargs*, "*string*") [Function File]
msgstruct = nargchk (*minargs*, *maxargs*, *nargs*, "*struct*") [Function File]

Return an appropriate error message string (or structure) if the number of inputs requested is invalid.

This is useful for checking to see that the number of input arguments supplied to a function is within an acceptable range.

See also: [nargoutchk], page 141, [error], page 161, [nargin], page 139, [nargout], page 140.

msgstr = nargoutchk (*minargs*, *maxargs*, *nargs*) [Function File]
msgstr = nargoutchk (*minargs*, *maxargs*, *nargs*, "*string*") [Function File]
msgstruct = nargoutchk (*minargs*, *maxargs*, *nargs*, "*struct*") [Function File]

Return an appropriate error message string (or structure) if the number of outputs requested is invalid.

This is useful for checking to see that the number of output arguments supplied to a function is within an acceptable range.

See also: [nargchk], page 141, [error], page 161, [nargout], page 140, [nargin], page 139.

11.3 Variable-length Argument Lists

Sometimes the number of input arguments is not known when the function is defined. As an example think of a function that returns the smallest of all its input arguments. For example,

```
a = smallest (1, 2, 3);
b = smallest (1, 2, 3, 4);
```

In this example both `a` and `b` would be 1. One way to write the `smallest` function is

```
function val = smallest (arg1, arg2, arg3, arg4, arg5)
  body
endfunction
```

and then use the value of `nargin` to determine which of the input arguments should be considered. The problem with this approach is that it can only handle a limited number of input arguments.

If the special parameter name `varargin` appears at the end of a function parameter list it indicates that the function takes a variable number of input arguments. Using `varargin` the function looks like this

```
function val = smallest (varargin)
  body
endfunction
```

In the function body the input arguments can be accessed through the variable `varargin`. This variable is a cell array containing all the input arguments. See Section 6.2 [Cell Arrays], page 85, for details on working with cell arrays. The `smallest` function can now be defined like this

```
function val = smallest (varargin)
  val = min ([varargin{:}]);
endfunction
```

This implementation handles any number of input arguments, but it's also a very simple solution to the problem.

A slightly more complex example of `varargin` is a function `print_arguments` that prints all input arguments. Such a function can be defined like this

```
function print_arguments (varargin)
  for i = 1:length (varargin)
    printf ("Input argument %d: ", i);
    disp (varargin{i});
  endfor
endfunction
```

This function produces output like this

```
print_arguments (1, "two", 3);
     ⊣ Input argument 1:  1
     ⊣ Input argument 2: two
     ⊣ Input argument 3:  3
```

`[`*reg*`,` *prop*`] = parseparams (`*params*`)` [Function File]

Return in *reg* the cell elements of *param* up to the first string element and in *prop* all remaining elements beginning with the first string element. For example

```
[reg, prop] = parseparams ({1, 2, "linewidth", 10})
reg =
{
  [1,1] = 1
  [1,2] = 2
}
prop =
{
  [1,1] = linewidth
  [1,2] = 10
}
```

The parseparams function may be used to separate 'regular' arguments and additional arguments given as property/value pairs of the *varargin* cell array.

See also: [varargin], page 141.

11.4 Variable-length Return Lists

It is possible to return a variable number of output arguments from a function using a syntax that's similar to the one used with the special `varargin` parameter name. To let a function return a variable number of output arguments the special output parameter name `varargout` is used. As with `varargin`, `varargout` is a cell array that will contain the requested output arguments.

As an example the following function sets the first output argument to 1, the second to 2, and so on.

```
function varargout = one_to_n ()
  for i = 1:nargout
    varargout{i} = i;
  endfor
endfunction
```

When called this function returns values like this

```
[a, b, c] = one_to_n ()
     ⇒ a =  1
     ⇒ b =  2
     ⇒ c =  3
```

If `varargin` (`varargout`) does not appear as the last element of the input (output) parameter list, then it is not special, and is handled the same as any other parameter name.

`[r1, r2, ..., rn] = deal (a)` [Function File]
`[r1, r2, ..., rn] = deal (a1, a2, ..., an)` [Function File]

Copy the input parameters into the corresponding output parameters. If only one input parameter is supplied, its value is copied to each of the outputs.

For example,

```
[a, b, c] = deal (x, y, z);
```

is equivalent to

```
a = x;
b = y;
c = z;
```

and

```
[a, b, c] = deal (x);
```

is equivalent to

```
a = b = c = x;
```

11.5 Returning From a Function

The body of a user-defined function can contain a `return` statement. This statement returns control to the rest of the Octave program. It looks like this:

```
return
```

Unlike the `return` statement in C, Octave's `return` statement cannot be used to return a value from a function. Instead, you must assign values to the list of return variables that

are part of the `function` statement. The `return` statement simply makes it easier to exit a function from a deeply nested loop or conditional statement.

Here is an example of a function that checks to see if any elements of a vector are nonzero.

```
function retval = any_nonzero (v)
  retval = 0;
  for i = 1:length (v)
    if (v (i) != 0)
      retval = 1;
      return;
    endif
  endfor
  printf ("no nonzero elements found\n");
endfunction
```

Note that this function could not have been written using the `break` statement to exit the loop once a nonzero value is found without adding extra logic to avoid printing the message if the vector does contain a nonzero element.

`return` [Keyword]

When Octave encounters the keyword `return` inside a function or script, it returns control to the caller immediately. At the top level, the return statement is ignored. A `return` statement is assumed at the end of every function definition.

11.6 Default Arguments

Since Octave supports variable number of input arguments, it is very useful to assign default values to some input arguments. When an input argument is declared in the argument list it is possible to assign a default value to the argument like this

```
function name (arg1 = val1, ...)
  body
endfunction
```

If no value is assigned to *arg1* by the user, it will have the value *val1*.

As an example, the following function implements a variant of the classic "Hello, World" program.

```
function hello (who = "World")
  printf ("Hello, %s!\n", who);
endfunction
```

When called without an input argument the function prints the following

```
hello ();
     ⊣ Hello, World!
```

and when it's called with an input argument it prints the following

```
hello ("Beautiful World of Free Software");
     ⊣ Hello, Beautiful World of Free Software!
```

Sometimes it is useful to explicitly tell Octave to use the default value of an input argument. This can be done writing a '`:`' as the value of the input argument when calling the function.

```
hello (:);
     ⊣ Hello, World!
```

11.7 Function Files

Except for simple one-shot programs, it is not practical to have to define all the functions you need each time you need them. Instead, you will normally want to save them in a file so that you can easily edit them, and save them for use at a later time.

Octave does not require you to load function definitions from files before using them. You simply need to put the function definitions in a place where Octave can find them.

When Octave encounters an identifier that is undefined, it first looks for variables or functions that are already compiled and currently listed in its symbol table. If it fails to find a definition there, it searches a list of directories (the *path*) for files ending in '`.m`' that have the same base name as the undefined identifier.[1] Once Octave finds a file with a name that matches, the contents of the file are read. If it defines a *single* function, it is compiled and executed. See Section 11.8 [Script Files], page 153, for more information about how you can define more than one function in a single file.

When Octave defines a function from a function file, it saves the full name of the file it read and the time stamp on the file. If the time stamp on the file changes, Octave may reload the file. When Octave is running interactively, time stamp checking normally happens at most once each time Octave prints the prompt. Searching for new function definitions also occurs if the current working directory changes.

Checking the time stamp allows you to edit the definition of a function while Octave is running, and automatically use the new function definition without having to restart your Octave session.

To avoid degrading performance unnecessarily by checking the time stamps on functions that are not likely to change, Octave assumes that function files in the directory tree '*octave-home*`/share/octave/`*version*`/m`' will not change, so it doesn't have to check their time stamps every time the functions defined in those files are used. This is normally a very good assumption and provides a significant improvement in performance for the function files that are distributed with Octave.

If you know that your own function files will not change while you are running Octave, you can improve performance by calling `ignore_function_time_stamp ("all")`, so that Octave will ignore the time stamps for all function files. Passing `"system"` to this function resets the default behavior.

`edit` *name* [Command]
`edit` *field value* [Command]
value `= edit` *get field* [Command]

Edit the named function, or change editor settings.

If `edit` is called with the name of a file or function as its argument it will be opened in a text editor.

- If the function *name* is available in a file on your path and that file is modifiable, then it will be edited in place. If it is a system function, then it will first be

[1] The '`.m`' suffix was chosen for compatibility with MATLAB.

copied to the directory HOME (see further down) and then edited. If no file is found, then the m-file variant, ending with ".m", will be considered. If still no file is found, then variants with a leading "@" and then with both a leading "@" and trailing ".m" will be considered.

- If *name* is the name of a function defined in the interpreter but not in an m-file, then an m-file will be created in HOME to contain that function along with its current definition.
- If `name.cc` is specified, then it will search for `name.cc` in the path and try to modify it, otherwise it will create a new '`.cc`' file in HOME. If *name* happens to be an m-file or interpreter defined function, then the text of that function will be inserted into the .cc file as a comment.
- If *name.ext* is on your path then it will be edited, otherwise the editor will be started with '`HOME/name.ext`' as the filename. If '`name.ext`' is not modifiable, it will be copied to HOME before editing.

  WARNING! You may need to clear name before the new definition is available. If you are editing a .cc file, you will need to mkoctfile '`name.cc`' before the definition will be available.

If `edit` is called with *field* and *value* variables, the value of the control field *field* will be *value*. If an output argument is requested and the first argument is `get` then `edit` will return the value of the control field *field*. If the control field does not exist, edit will return a structure containing all fields and values. Thus, `edit get all` returns a complete control structure. The following control fields are used:

'`editor`' This is the editor to use to modify the functions. By default it uses Octave's `EDITOR` built-in function, which comes from `getenv("EDITOR")` and defaults to `emacs`. Use `%s` In place of the function name. For example,

'`[EDITOR, " %s"]`'
Use the editor which Octave uses for `bug_report`.

'`"xedit %s &"`'
pop up simple X11 editor in a separate window

'`"gnudoit -q \"(find-file \\\"%s\\\")\""`'
Send it to current Emacs; must have `(gnuserv-start)` in '`.emacs`'.

See also field 'mode', which controls how the editor is run by Octave.

On Cygwin, you will need to convert the Cygwin path to a Windows path if you are using a native Windows editor. For example

```
'"C:/Program Files/Good Editor/Editor.exe" "$(cygpath -wa %s)"'
```

'`home`' This is the location of user local m-files. Be be sure it is in your path. The default is '`~/octave`'.

'`author`' This is the name to put after the "## Author:" field of new functions. By default it guesses from the `gecos` field of password database.

'`email`' This is the e-mail address to list after the name in the author field. By default it guesses `<$LOGNAME@$HOSTNAME>`, and if `$HOSTNAME` is not de-

fined it uses `uname -n`. You probably want to override this. Be sure to use `<user@host>` as your format.

'license'

- 'gpl' GNU General Public License (default).
- 'bsd' BSD-style license without advertising clause.
- 'pd' Public domain.
- '"text"' Your own default copyright and license.

Unless you specify 'pd', edit will prepend the copyright statement with "Copyright (C) yyyy Function Author".

'mode' This value determines whether the editor should be started in async mode (editor is started in the background and Octave continues) or sync mode (Octave waits until the editor exits). Set it to "async" to start the editor in async mode. The default is "sync" (see also "system").

'editinplace'
Determines whether files should be edited in place, without regard to whether they are modifiable or not. The default is `false`.

`mfilename ()` [Built-in Function]
`mfilename ("fullpath")` [Built-in Function]
`mfilename ("fullpathext")` [Built-in Function]

Return the name of the currently executing file. At the top-level, return the empty string. Given the argument `"fullpath"`, include the directory part of the file name, but not the extension. Given the argument `"fullpathext"`, include the directory part of the file name and the extension.

val = `ignore_function_time_stamp ()` [Built-in Function]
old_val = `ignore_function_time_stamp (`*new_val*`)` [Built-in Function]

Query or set the internal variable that controls whether Octave checks the time stamp on files each time it looks up functions defined in function files. If the internal variable is set to `"system"`, Octave will not automatically recompile function files in subdirectories of '*octave-home*/lib/*version*' if they have changed since they were last compiled, but will recompile other function files in the search path if they change. If set to `"all"`, Octave will not recompile any function files unless their definitions are removed with `clear`. If set to "none", Octave will always check time stamps on files to determine whether functions defined in function files need to recompiled.

11.7.1 Manipulating the load path

When a function is called, Octave searches a list of directories for a file that contains the function declaration. This list of directories is known as the load path. By default the load path contains a list of directories distributed with Octave plus the current working directory. To see your current load path call the `path` function without any input or output arguments.

It is possible to add or remove directories to or from the load path using `addpath` and `rmpath`. As an example, the following code adds '~/Octave' to the load path.

```
addpath("~/Octave")
```

After this the directory '`~/Octave`' will be searched for functions.

`addpath` (*dir1*, ...) [Built-in Function]
`addpath` (*dir1*, ..., *option*) [Built-in Function]

Add *dir1*, ... to the current function search path. If *option* is '`"-begin"`' or 0 (the default), prepend the directory name to the current path. If *option* is '`"-end"`' or 1, append the directory name to the current path. Directories added to the path must exist.

See also: [path], page 148, [rmpath], page 148, [genpath], page 148, [pathdef], page 148, [savepath], page 148, [pathsep], page 149.

`genpath` (*dir*) [Built-in Function]

Return a path constructed from *dir* and all its subdirectories.

`rmpath` (*dir1*, ...) [Built-in Function]

Remove *dir1*, ... from the current function search path.

See also: [path], page 148, [addpath], page 148, [genpath], page 148, [pathdef], page 148, [savepath], page 148, [pathsep], page 149.

`savepath` (*file*) [Function File]

Save the portion of the current function search path, that is not set during Octave's initialization process, to *file*. If *file* is omitted, '`~/.octaverc`' is used. If successful, `savepath` returns 0.

See also: [path], page 148, [addpath], page 148, [rmpath], page 148, [genpath], page 148, [pathdef], page 148, [pathsep], page 149.

`path` (...) [Built-in Function]

Modify or display Octave's load path.

If *nargin* and *nargout* are zero, display the elements of Octave's load path in an easy to read format.

If *nargin* is zero and nargout is greater than zero, return the current load path.

If *nargin* is greater than zero, concatenate the arguments, separating them with `pathsep()`. Set the internal search path to the result and return it.

No checks are made for duplicate elements.

See also: [addpath], page 148, [rmpath], page 148, [genpath], page 148, [pathdef], page 148, [savepath], page 148, [pathsep], page 149.

val = `pathdef` () [Function File]

Return the default path for Octave. The path information is extracted from one of three sources. In order of preference, those are;

1. '`~/.octaverc`'
2. '`<octave-home>/.../<version>/m/startup/octaverc`'
3. Octave's path prior to changes by any octaverc.

See also: [path], page 148, [addpath], page 148, [rmpath], page 148, [genpath], page 148, [savepath], page 148, [pathsep], page 149.

val = pathsep () [Built-in Function]
old_val = pathsep (*new_val*) [Built-in Function]

Query or set the character used to separate directories in a path.

See also: [filesep], page 529, [dir], page 540, [ls], page 540.

rehash () [Built-in Function]

Reinitialize Octave's load path directory cache.

file_in_loadpath (*file*) [Built-in Function]
file_in_loadpath (*file*, "*all*") [Built-in Function]

Return the absolute name of *file* if it can be found in the list of directories specified by `path`. If no file is found, return an empty matrix.

If the first argument is a cell array of strings, search each directory of the loadpath for element of the cell array and return the first that matches.

If the second optional argument `"all"` is supplied, return a cell array containing the list of all files that have the same name in the path. If no files are found, return an empty cell array.

See also: [file_in_path], page 528, [path], page 148.

restoredefaultpath (...) [Built-in Function]

Restore Octave's path to it's initial state at startup.

See also: [path], page 148, [addpath], page 148, [rmpath], page 148, [genpath], page 148, [pathdef], page 148, [savepath], page 148, [pathsep], page 149.

command_line_path (...) [Built-in Function]

Return the command line path variable.

See also: [path], page 148, [addpath], page 148, [rmpath], page 148, [genpath], page 148, [pathdef], page 148, [savepath], page 148, [pathsep], page 149.

find_dir_in_path (*dir*) [Built-in Function]

Return the full name of the path element matching *dir*. The match is performed at the end of each path element. For example, if *dir* is `"foo/bar"`, it matches the path element `"/some/dir/foo/bar"`, but not `"/some/dir/foo/bar/baz"` or `"/some/dir/allfoo/bar"`.

11.7.2 Subfunctions

A function file may contain secondary functions called *subfunctions*. These secondary functions are only visible to the other functions in the same function file. For example, a file '`f.m`' containing

```
function f ()
  printf ("in f, calling g\n");
  g ()
endfunction
function g ()
  printf ("in g, calling h\n");
  h ()
endfunction
function h ()
  printf ("in h\n")
endfunction
```

defines a main function f and two subfunctions. The subfunctions g and h may only be called from the main function f or from the other subfunctions, but not from outside the file 'f.m'.

11.7.3 Private Functions

In many cases one function needs to access one or more helper functions. If the helper function is limited to the scope of a single function, then subfunctions as discussed above might be used. However, if a single helper function is used by more than one function, then this is no longer possible. In this case the helper functions might be placed in a subdirectory, called "private", of the directory in which the functions needing access to this helper function are found.

As a simple example, consider a function func1, that calls a helper function func2 to do much of the work. For example

```
function y = func1 (x)
  y = func2 (x);
endfunction
```

Then if the path to func1 is <directory>/func1.m, and if func2 is found in the directory <directory>/private/func2.m, then func2 is only available for use of the functions, like func1, that are found in <directory>.

11.7.4 Overloading and Autoloading

The dispatch function can be used to alias one function name to another. It can be used to alias all calls to a particular function name to another function, or the alias can be limited to only a particular variable type. Consider the example

```
function y = spsin (x)
  printf ("Calling spsin\n");
  fflush(stdout);
  y = spfun ("sin", x);
endfunction

dispatch ("sin", "spsin", "sparse matrix");
y0 = sin(eye(3));
y1 = sin(speye(3));
```

which aliases the user-defined function `spsin` to `sin`, but only for real sparse matrices. Note that the builtin `sin` already correctly treats sparse matrices and so this example is only illustrative.

`dispatch (f, r, type)` [Loadable Function]

Replace the function *f* with a dispatch so that function *r* is called when *f* is called with the first argument of the named *type*. If the type is *any* then call *r* if no other type matches. The original function *f* is accessible using `builtin (f, ...)`.

If *r* is omitted, clear dispatch function associated with *type*.

If both *r* and *type* are omitted, list dispatch functions for *f*.

See also: [builtin], page 151.

`[...] builtin (f, ...)` [Loadable Function]

Call the base function *f* even if *f* is overloaded to some other function for the given type signature.

See also: [dispatch], page 151.

A single dynamically linked file might define several functions. However, as Octave searches for functions based on the functions filename, Octave needs a manner in which to find each of the functions in the dynamically linked file. On operating systems that support symbolic links, it is possible to create a symbolic link to the original file for each of the functions which it contains.

However, there is at least one well known operating system that doesn't support symbolic links. Making copies of the original file for each of the functions is undesirable as it increases the amount of disk space used by Octave. Instead Octave supplies the `autoload` function, that permits the user to define in which file a certain function will be found.

`autoload (function, file)` [Built-in Function]

Define *function* to autoload from *file*.

The second argument, *file*, should be an absolute file name or a file name in the same directory as the function or script from which the autoload command was run. *file* should not depend on the Octave load path.

Normally, calls to `autoload` appear in PKG_ADD script files that are evaluated when a directory is added to the Octave's load path. To avoid having to hardcode directory names in *file*, if *file* is in the same directory as the PKG_ADD script then

```
autoload ("foo", "bar.oct");
```

will load the function `foo` from the file `bar.oct`. The above when `bar.oct` is not in the same directory or uses like

```
autoload ("foo", file_in_loadpath ("bar.oct"))
```

are strongly discouraged, as their behavior might be unpredictable.

With no arguments, return a structure containing the current autoload map.

See also: [PKG_ADD], page 551.

11.7.5 Function Locking

It is sometime desirable to lock a function into memory with the `mlock` function. This is typically used for dynamically linked functions in Oct-files or mex-files that contain some initialization, and it is desirable that calling `clear` does not remove this initialization.

As an example,

```
mlock ("my_function");
```

prevents `my_function` from being removed from memory, even if `clear` is called. It is possible to determine if a function is locked into memory with the `mislocked`, and to unlock a function with `munlock`, which the following illustrates.

```
mlock ("my_function");
mislocked ("my_function")
⇒ ans = 1
munlock ("my_function");
mislocked ("my_function")
⇒ ans = 0
```

A common use of `mlock` is to prevent persistent variables from being removed from memory, as the following example shows:

```
function count_calls()
  persistent calls = 0;
  printf ("'count_calls' has been called %d times\n",
          ++calls);
endfunction
mlock ("count_calls");

count_calls ();
⊣ 'count_calls' has been called 1 times

clear count_calls
count_calls ();
⊣ 'count_calls' has been called 2 times
```

It is, however, often inconvenient to lock a function from the prompt, so it is also possible to lock a function from within its body. This is simply done by calling `mlock` from within the function.

```
function count_calls ()
  mlock ();
  persistent calls = 0;
  printf ("'count_calls' has been called %d times\n",
          ++calls);
endfunction
```

`mlock` might equally be used to prevent changes to a function from having effect in Octave, though a similar effect can be had with the `ignore_function_time_stamp` function.

`mlock ()` [Built-in Function]

Lock the current function into memory so that it can't be cleared.

See also: [munlock], page 153, [mislocked], page 153, [persistent], page 100.

`munlock (fcn)` [Built-in Function]

Unlock the named function. If no function is named then unlock the current function.

See also: [mlock], page 152, [mislocked], page 153, [persistent], page 100.

`mislocked (fcn)` [Built-in Function]

Return true if the named function is locked. If no function is named then return true if the current function is locked.

See also: [mlock], page 152, [munlock], page 153, [persistent], page 100.

11.7.6 Function Precedence

Given the numerous different ways that Octave can define a function, it is possible and even likely that multiple versions of a function, might be defined within a particular scope. The precedence of which function will be used within a particular scope is given by

1. Subfunction A subfunction with the required function name in the given scope.
2. Private function A function defined within a private directory of the directory which contains the current function.
3. Class constructor A function that constuctors a user class as defined in chapter Chapter 33 [Object Oriented Programming], page 497.
4. Class method An overloaded function of a class as in chapter Chapter 33 [Object Oriented Programming], page 497.
5. Legacy Dispatch An overloaded function as defined by See [doc-dispatch], page 151.
6. Command-line Function A function that has been defined on the command-line.
7. Autoload function A function that is marked as autoloaded with See [doc-autoload], page 151.
8. A Function on the Path A function that can be found on the users load-path. There can also be Oct-file, mex-file or m-file versions of this function and the precedence between these versions are in that order.
9. Built-in function A function that is builtin to Octave itself such as `numel`, `size`, etc.

11.8 Script Files

A script file is a file containing (almost) any sequence of Octave commands. It is read and evaluated just as if you had typed each command at the Octave prompt, and provides a convenient way to perform a sequence of commands that do not logically belong inside a function.

Unlike a function file, a script file must *not* begin with the keyword `function`. If it does, Octave will assume that it is a function file, and that it defines a single function that should be evaluated as soon as it is defined.

A script file also differs from a function file in that the variables named in a script file are not local variables, but are in the same scope as the other variables that are visible on the command line.

Even though a script file may not begin with the `function` keyword, it is possible to define more than one function in a single script file and load (but not execute) all of them at once. To do this, the first token in the file (ignoring comments and other white space)

must be something other than `function`. If you have no other statements to evaluate, you can use a statement that has no effect, like this:

```
# Prevent Octave from thinking that this
# is a function file:

1;

# Define function one:

function one ()
  ...
```

To have Octave read and compile these functions into an internal form, you need to make sure that the file is in Octave's load path (accessible through the `path` function), then simply type the base name of the file that contains the commands. (Octave uses the same rules to search for script files as it does to search for function files.)

If the first token in a file (ignoring comments) is `function`, Octave will compile the function and try to execute it, printing a message warning about any non-whitespace characters that appear after the function definition.

Note that Octave does not try to look up the definition of any identifier until it needs to evaluate it. This means that Octave will compile the following statements if they appear in a script file, or are typed at the command line,

```
# not a function file:
1;
function foo ()
  do_something ();
endfunction
function do_something ()
  do_something_else ();
endfunction
```

even though the function `do_something` is not defined before it is referenced in the function `foo`. This is not an error because Octave does not need to resolve all symbols that are referenced by a function until the function is actually evaluated.

Since Octave doesn't look for definitions until they are needed, the following code will always print '`bar = 3`' whether it is typed directly on the command line, read from a script file, or is part of a function body, even if there is a function or script file called '`bar.m`' in Octave's path.

```
eval ("bar = 3");
bar
```

Code like this appearing within a function body could fool Octave if definitions were resolved as the function was being compiled. It would be virtually impossible to make Octave clever enough to evaluate this code in a consistent fashion. The parser would have to be able to perform the call to `eval` at compile time, and that would be impossible unless all the references in the string to be evaluated could also be resolved, and requiring that would be too restrictive (the string might come from user input, or depend on things that are not known until the function is evaluated).

Although Octave normally executes commands from script files that have the name '*file*.m', you can use the function `source` to execute commands from any file.

`source (`*`file`*`)` [Built-in Function]

Parse and execute the contents of *file*. This is equivalent to executing commands from a script file, but without requiring the file to be named '*file*.m'.

11.9 Function Handles, Inline Functions, and Anonymous Functions

It can be very convenient store a function in a variable so that it can be passed to a different function. For example, a function that performs numerical minimization needs access to the function that should be minimized.

11.9.1 Function Handles

A function handle is a pointer to another function and is defined with the syntax

```
@function-name
```

For example

```
f = @sin;
```

Creates a function handle called `f` that refers to the function `sin`.

Function handles are used to call other functions indirectly, or to pass a function as an argument to another function like `quad` or `fsolve`. For example

```
f = @sin;
quad (f, 0, pi)
    ⇒ 2
```

You may use `feval` to call a function using function handle, or simply write the name of the function handle followed by an argument list. If there are no arguments, you must use an empty argument list '`()`'. For example

```
f = @sin;
feval (f, pi/4)
    ⇒ 0.70711
f (pi/4)
    ⇒ 0.70711
```

`functions (`*`fcn_handle`*`)` [Built-in Function]

Return a struct containing information about the function handle *fcn_handle*.

`func2str (`*`fcn_handle`*`)` [Built-in Function]

Return a string containing the name of the function referenced by the function handle *fcn_handle*.

`str2func (`*`fcn_name`*`)` [Built-in Function]

Return a function handle constructed from the string *fcn_name*.

11.9.2 Anonymous Functions

Anonymous functions are defined using the syntax

```
@(argument-list) expression
```

Any variables that are not found in the argument list are inherited from the enclosing scope. Anonymous functions are useful for creating simple unnamed functions from expressions or for wrapping calls to other functions to adapt them for use by functions like `quad`. For example,

```
f = @(x) x.^2;
quad (f, 0, 10)
    ⇒ 333.33
```

creates a simple unnamed function from the expression `x.^2` and passes it to `quad`,

```
quad (@(x) sin (x), 0, pi)
    ⇒ 2
```

wraps another function, and

```
a = 1;
b = 2;
quad (@(x) betainc (x, a, b), 0, 0.4)
    ⇒ 0.13867
```

adapts a function with several parameters to the form required by `quad`. In this example, the values of *a* and *b* that are passed to `betainc` are inherited from the current environment.

11.9.3 Inline Functions

An inline function is created from a string containing the function body using the `inline` function. The following code defines the function $f(x) = x^2 + 2$.

```
f = inline("x^2 + 2");
```

After this it is possible to evaluate f at any x by writing `f(x)`.

inline (*str*) [Built-in Function]
inline (*str*, *arg1*, ...) [Built-in Function]
inline (*str*, *n*) [Built-in Function]

Create an inline function from the character string *str*. If called with a single argument, the arguments of the generated function are extracted from the function itself. The generated function arguments will then be in alphabetical order. It should be noted that i, and j are ignored as arguments due to the ambiguity between their use as a variable or their use as an inbuilt constant. All arguments followed by a parenthesis are considered to be functions.

If the second and subsequent arguments are character strings, they are the names of the arguments of the function.

If the second argument is an integer *n*, the arguments are `"x"`, `"P1"`, ..., `"PN"`.

See also: [argnames], page 156, [formula], page 157, [vectorize], page 157.

argnames (*fun*) [Built-in Function]

Return a cell array of character strings containing the names of the arguments of the inline function *fun*.

See also: [inline], page 156, [formula], page 157, [vectorize], page 157.

formula (*fun*) [Built-in Function]

Return a character string representing the inline function *fun*. Note that `char (fun)` is equivalent to `formula (fun)`.

See also: [argnames], page 156, [inline], page 156, [vectorize], page 157.

vectorize (*fun*) [Built-in Function]

Create a vectorized version of the inline function *fun* by replacing all occurrences of `*`, `/`, etc., with `.*`, `./`, etc.

symvar (*s*) [Function File]

Identifies the argument names in the function defined by a string. Common constant names such as `pi`, `NaN`, `Inf`, `eps`, `i` or `j` are ignored. The arguments that are found are returned in a cell array of strings. If no variables are found then the returned cell array is empty.

11.10 Commands

Commands are a special class of functions that only accept string input arguments. A command can be called as an ordinary function, but it can also be called without the parentheses like the following example shows

```
my_command hello world
```

which is the same as

```
my_command("hello", "world")
```

The general form of a command call is

```
name arg1 arg2 ...
```

which translates directly to

```
name ("arg1", "arg2", ...)
```

A function can be used as a command if it accepts string input arguments. To do this, the function must be marked as a command, which can be done with the `mark_as_command` command like this

```
mark_as_command name
```

where `name` is the function to be marked as a command.

One difficulty of commands occurs when one of the string input arguments are stored in a variable. Since Octave can't tell the difference between a variable name, and an ordinary string, it is not possible to pass a variable as input to a command. In such a situation a command must be called as a function.

mark_as_command (*name*) [Built-in Function]

This function is obsolete and will be removed from a future version of Octave.

unmark_command (*name*) [Built-in Function]

This function is obsolete and will be removed from a future version of Octave.

iscommand (*name*) [Built-in Function]

This function is obsolete and will be removed from a future version of Octave.

`mark_as_rawcommand` (*name*) [Built-in Function]

This function is obsolete and will be removed from a future version of Octave.

`unmark_rawcommand` (*name*) [Built-in Function]

This function is obsolete and will be removed from a future version of Octave.

`israwcommand` (*name*) [Built-in Function]

This function is obsolete and will be removed from a future version of Octave.

11.11 Organization of Functions Distributed with Octave

Many of Octave's standard functions are distributed as function files. They are loosely organized by topic, in subdirectories of '*octave-home*/lib/octave/*version*/m', to make it easier to find them.

The following is a list of all the function file subdirectories, and the types of functions you will find there.

'`audio`' Functions for playing and recording sounds.

'`control`' Functions for design and simulation of automatic control systems.

'`elfun`' Elementary functions.

'`finance`' Functions for computing interest payments, investment values, and rates of return.

'`general`' Miscellaneous matrix manipulations, like `flipud`, `rot90`, and `triu`, as well as other basic functions, like `ismatrix`, `nargchk`, etc.

'`image`' Image processing tools. These functions require the X Window System.

'`io`' Input-output functions.

'`linear-algebra`'
Functions for linear algebra.

'`miscellaneous`'
Functions that don't really belong anywhere else.

'`optimization`'
Minimization of functions.

'`path`' Functions to manage the directory path Octave uses to find functions.

'`pkg`' Install external packages of functions in Octave.

'`plot`' Functions for displaying and printing two- and three-dimensional graphs.

'`polynomial`'
Functions for manipulating polynomials.

'`set`' Functions for creating and manipulating sets of unique values.

'`signal`' Functions for signal processing applications.

'`sparse`' Functions for handling sparse matrices.

'`specfun`' Special functions.

'special-matrix'
: Functions that create special matrix forms.

'startup'
: Octave's system-wide startup file.

'statistics'
: Statistical functions.

'strings'
: Miscellaneous string-handling functions.

'testfun'
: Perform unit tests on other functions.

'time'
: Functions related to time keeping.

12 Errors and Warnings

Octave includes several functions for printing error and warning messages. When you write functions that need to take special action when they encounter abnormal conditions, you should print the error messages using the functions described in this chapter.

Since many of Octave's functions use these functions, it is also useful to understand them, so that errors and warnings can be handled.

12.1 Handling Errors

An error is something that occurs when a program is in a state where it doesn't make sense to continue. An example is when a function is called with too few input arguments. In this situation the function should abort with an error message informing the user of the lacking input arguments.

Since an error can occur during the evaluation of a program, it is very convenient to be able to detect that an error occurred, so that the error can be fixed. This is possible with the `try` statement described in Section 10.9 [The `try` Statement], page 134.

12.1.1 Raising Errors

The most common use of errors is for checking input arguments to functions. The following example calls the `error` function if the function `f` is called without any input arguments.

```
function f (arg1)
  if (nargin == 0)
    error("not enough input arguments");
  endif
endfunction
```

When the `error` function is called, it prints the given message and returns to the Octave prompt. This means that no code following a call to `error` will be executed.

`error` (*template*, ...) [Built-in Function]
`error` (*id*, *template*, ...) [Built-in Function]

Format the optional arguments under the control of the template string *template* using the same rules as the `printf` family of functions (see Section 14.2.4 [Formatted Output], page 190) and print the resulting message on the `stderr` stream. The message is prefixed by the character string '`error: `'.

Calling `error` also sets Octave's internal error state such that control will return to the top level without evaluating any more commands. This is useful for aborting from functions or scripts.

If the error message does not end with a new line character, Octave will print a traceback of all the function calls leading to the error. For example, given the following function definitions:

```
function f () g (); end
function g () h (); end
function h () nargin == 1 || error ("nargin != 1"); end
```

calling the function `f` will result in a list of messages that can help you to quickly locate the exact location of the error:

```
f ()
error: nargin != 1
error: called from:
error:   error at line -1, column -1
error:   h at line 1, column 27
error:   g at line 1, column 15
error:   f at line 1, column 15
```

If the error message ends in a new line character, Octave will print the message but will not display any traceback messages as it returns control to the top level. For example, modifying the error message in the previous example to end in a new line causes Octave to only print a single message:

```
function h () nargin == 1 || error ("nargin != 1\n"); end
f ()
error: nargin != 1
```

Since it is common to use errors when there is something wrong with the input to a function, Octave supports functions to simplify such code. When the `print_usage` function is called, it reads the help text of the function calling `print_usage`, and presents a useful error. If the help text is written in Texinfo it is possible to present an error message that only contains the function prototypes as described by the `@deftypefn` parts of the help text. When the help text isn't written in Texinfo, the error message contains the entire help message.

Consider the following function.

```
## -*- texinfo -*-
## @deftypefn {Function File} f (@var{arg1})
## Function help text goes here...
## @end deftypefn
function f (arg1)
  if (nargin == 0)
    print_usage ();
  endif
endfunction
```

When it is called with no input arguments it produces the following error.

```
f ()

-| error: Invalid call to f.  Correct usage is:
-|
-|  -- Function File: f (ARG1)
-|
-|
-| Additional help for built-in functions and operators is
-| available in the on-line version of the manual.  Use the command
-| ‘doc <topic>’ to search the manual index.
-|
-| Help and information about Octave is also available on the WWW
-| at http://www.octave.org and via the help@octave.org
-| mailing list.
```

`print_usage ()` [Function File]
`print_usage (`*name*`)` [Function File]

Print the usage message for a function. When called with no input arguments the `print_usage` function displays the usage message of the currently executing function.

See also: [help], page 17.

`usage (`*msg*`)` [Built-in Function]

Print the message *msg*, prefixed by the string '`usage: `', and set Octave's internal error state such that control will return to the top level without evaluating any more commands. This is useful for aborting from functions.

After `usage` is evaluated, Octave will print a traceback of all the function calls leading to the usage message.

You should use this function for reporting problems errors that result from an improper call to a function, such as calling a function with an incorrect number of arguments, or with arguments of the wrong type. For example, most functions distributed with Octave begin with code like this

```
if (nargin != 2)
  usage ("foo (a, b)");
endif
```

to check for the proper number of arguments.

`beep ()` [Function File]

Produce a beep from the speaker (or visual bell).

See also: [puts], page 190, [fputs], page 189, [printf], page 190, [fprintf], page 191.

val `= beep_on_error ()` [Built-in Function]
old_val `= beep_on_error (`*new_val*`)` [Built-in Function]

Query or set the internal variable that controls whether Octave will try to ring the terminal bell before printing an error message.

12.1.2 Catching Errors

When an error occurs, it can be detected and handled using the `try` statement as described in Section 10.9 [The try Statement], page 134. As an example, the following piece of code counts the number of errors that occurs during a `for` loop.

```
number_of_errors = 0;
for n = 1:100
  try
    ...
  catch
    number_of_errors++;
  end_try_catch
endfor
```

The above example treats all errors the same. In many situations it can however be necessary to discriminate between errors, and take different actions depending on the error. The `lasterror` function returns a structure containing information about the last error that occurred. As an example, the code above could be changed to count the number of errors related to the '`*`' operator.

```
number_of_errors = 0;
for n = 1:100
  try
    ...
  catch
    msg = lasterror.message;
    if (strfind (msg, "operator *"))
      number_of_errors++;
    endif
  end_try_catch
endfor
```

err = `lasterror` (*err*) [Built-in Function]
`lasterror` (*'reset'*) [Built-in Function]

Returns or sets the last error message. Called without any arguments returns a structure containing the last error message, as well as other information related to this error. The elements of this structure are:

'message' The text of the last error message

'identifier' The message identifier of this error message

'stack' A structure containing information on where the message occurred. This might be an empty structure if this in the case where this information cannot be obtained. The fields of this structure are:

- 'file' The name of the file where the error occurred
- 'name' The name of function in which the error occurred
- 'line' The line number at which the error occurred
- 'column' An optional field with the column number at which the error occurred

The *err* structure may also be passed to `lasterror` to set the information about the last error. The only constraint on *err* in that case is that it is a scalar structure. Any fields of *err* that match the above are set to the value passed in *err*, while other fields are set to their default values.

If `lasterror` is called with the argument 'reset', all values take their default values.

[*msg*, *msgid*] = lasterr (*msg*, *msgid*) [Built-in Function]

Without any arguments, return the last error message. With one argument, set the last error message to *msg*. With two arguments, also set the last message identifier.

When an error has been handled it is possible to raise it again. This can be useful when an error needs to be detected, but the program should still abort. This is possible using the `rethrow` function. The previous example can now be changed to count the number of errors related to the '`*`' operator, but still abort if another kind of error occurs.

```
number_of_errors = 0;
for n = 1:100
  try
    ...
  catch
    msg = lasterror.message;
    if (strfind (msg, "operator *"))
      number_of_errors++;
    else
      rethrow (lasterror);
    endif
  end_try_catch
endfor
```

rethrow (*err*) [Built-in Function]

Reissues a previous error as defined by *err*. *err* is a structure that must contain at least the 'message' and 'identifier' fields. *err* can also contain a field 'stack' that gives information on the assumed location of the error. Typically *err* is returned from `lasterror`.

See also: [lasterror], page 164, [lasterr], page 165, [error], page 161.

err = errno () [Built-in Function]
err = errno (*val*) [Built-in Function]
err = errno (*name*) [Built-in Function]

Return the current value of the system-dependent variable errno, set its value to *val* and return the previous value, or return the named error code given *name* as a character string, or -1 if *name* is not found.

errno_list () [Built-in Function]

Return a structure containing the system-dependent errno values.

12.2 Handling Warnings

Like an error, a warning is issued when something unexpected happens. Unlike an error, a warning doesn't abort the currently running program. A simple example of a warning is when a number is divided by zero. In this case Octave will issue a warning and assign the value `Inf` to the result.

```
a = 1/0
     ⊣ warning: division by zero
     ⇒ a = Inf
```

12.2.1 Issuing Warnings

It is possible to issue warnings from any code using the `warning` function. In its most simple form, the `warning` function takes a string describing the warning as its input argument. As an example, the following code controls if the variable '`a`' is non-negative, and if not issues a warning and sets '`a`' to zero.

```
a = -1;
if (a < 0)
  warning ("'a' must be non-negative.  Setting 'a' to zero.");
  a = 0;
endif
     ⊣ 'a' must be non-negative.  Setting 'a' to zero.
```

Since warnings aren't fatal to a running program, it is not possible to catch a warning using the `try` statement or something similar. It is however possible to access the last warning as a string using the `lastwarn` function.

It is also possible to assign an identification string to a warning. If a warning has such an ID the user can enable and disable this warning as will be described in the next section. To assign an ID to a warning, simply call `warning` with two string arguments, where the first is the identification string, and the second is the actual warning.

warning (*template*, . . .) [Built-in Function]
warning (*id*, *template*, . . .) [Built-in Function]

Format the optional arguments under the control of the template string *template* using the same rules as the `printf` family of functions (see Section 14.2.4 [Formatted Output], page 190) and print the resulting message on the `stderr` stream. The message is prefixed by the character string '`warning: `'. You should use this function when you want to notify the user of an unusual condition, but only when it makes sense for your program to go on.

The optional message identifier allows users to enable or disable warnings tagged by *id*. The special identifier '`"all"`' may be used to set the state of all warnings.

warning ("*on*", *id*) [Built-in Function]
warning ("*off*", *id*) [Built-in Function]
warning ("*error*", *id*) [Built-in Function]
warning ("*query*", *id*) [Built-in Function]

Set or query the state of a particular warning using the identifier *id*. If the identifier is omitted, a value of '`"all"`' is assumed. If you set the state of a warning to '`"error"`', the warning named by *id* is handled as if it were an error instead.

See also: [warning_ids], page 167.

[*msg*, *msgid*] = lastwarn (*msg*, *msgid*) [Built-in Function]

Without any arguments, return the last warning message. With one argument, set the last warning message to *msg*. With two arguments, also set the last message identifier.

12.2.2 Enabling and Disabling Warnings

The `warning` function also allows you to control which warnings are actually printed to the screen. If the `warning` function is called with a string argument that is either `"on"` or `"off"` all warnings will be enabled or disabled.

It is also possible to enable and disable individual warnings through their string identifications. The following code will issue a warning

```
warning ("non-negative-variable",
         "'a' must be non-negative.  Setting 'a' to zero.");
```

while the following won't issue a warning

```
warning ("off", "non-negative-variable");
warning ("non-negative-variable",
         "'a' must be non-negative.  Setting 'a' to zero.");
```

The functions distributed with Octave can issue one of the following warnings.

`Octave:array-to-scalar`
: If the `Octave:array-to-scalar` warning is enabled, Octave will warn when an implicit conversion from an array to a scalar value is attempted. By default, the `Octave:array-to-scalar` warning is disabled.

`Octave:array-to-vector`
: If the `Octave:array-to-vector` warning is enabled, Octave will warn when an implicit conversion from an array to a vector value is attempted. By default, the `Octave:array-to-vector` warning is disabled.

`Octave:assign-as-truth-value`
: If the `Octave:assign-as-truth-value` warning is enabled, a warning is issued for statements like

```
if (s = t)
  ...
```

since such statements are not common, and it is likely that the intent was to write

```
if (s == t)
  ...
```

instead.

There are times when it is useful to write code that contains assignments within the condition of a `while` or `if` statement. For example, statements like

```
while (c = getc())
  ...
```

are common in C programming.

It is possible to avoid all warnings about such statements by disabling the `Octave:assign-as-truth-value` warning, but that may also let real errors like

```
if (x = 1)  # intended to test (x == 1)!
  ...
```

slip by.

In such cases, it is possible suppress errors for specific statements by writing them with an extra set of parentheses. For example, writing the previous example as

```
while ((c = getc()))
  ...
```

will prevent the warning from being printed for this statement, while allowing Octave to warn about other assignments used in conditional contexts.

By default, the `Octave:assign-as-truth-value` warning is enabled.

`Octave:associativity-change`
: If the `Octave:associativity-change` warning is enabled, Octave will warn about possible changes in the meaning of some code due to changes in associativity for some operators. Associativity changes have typically been made for MATLAB compatibility. By default, the `Octave:associativity-change` warning is enabled.

`Octave:divide-by-zero`
: If the `Octave:divide-by-zero` warning is enabled, a warning is issued when Octave encounters a division by zero. By default, the `Octave:divide-by-zero` warning is enabled.

`Octave:empty-list-elements`
: If the `Octave:empty-list-elements` warning is enabled, a warning is issued when an empty matrix is found in a matrix list. For example,

```
a = [1, [], 3, [], 5]
```

By default, the `Octave:empty-list-elements` warning is enabled.

`Octave:fortran-indexing`
: If the `Octave:fortran-indexing` warning is enabled, a warning is printed for expressions which select elements of a two-dimensional matrix using a single index. By default, the `Octave:fortran-indexing` warning is disabled.

`Octave:function-name-clash`
: If the `Octave:function-name-clash` warning is enabled, a warning is issued when Octave finds that the name of a function defined in a function file differs from the name of the file. (If the names disagree, the name declared inside the file is ignored.) By default, the `Octave:function-name-clash` warning is enabled.

`Octave:future-time-stamp`
: If the `Octave:future-time-stamp` warning is enabled, Octave will print a warning if it finds a function file with a time stamp that is in the future. By default, the `Octave:future-time-stamp` warning is enabled.

`Octave:imag-to-real`
If the `Octave:imag-to-real` warning is enabled, a warning is printed for implicit conversions of complex numbers to real numbers. By default, the `Octave:imag-to-real` warning is disabled.

`Octave:matlab-incompatible`
Print warnings for Octave language features that may cause compatibility problems with MATLAB.

`Octave:missing-semicolon`
If the `Octave:missing-semicolon` warning is enabled, Octave will warn when statements in function definitions don't end in semicolons. By default the `Octave:missing-semicolon` warning is disabled.

`Octave:neg-dim-as-zero`
If the `Octave:neg-dim-as-zero` warning is enabled, print a warning for expressions like

```
eye (-1)
```

By default, the `Octave:neg-dim-as-zero` warning is disabled.

`Octave:num-to-str`
If the `Octave:num-to-str` warning is enable, a warning is printed for implicit conversions of numbers to their ASCII character equivalents when strings are constructed using a mixture of strings and numbers in matrix notation. For example,

```
[ "f", 111, 111 ]
     ⇒ "foo"
```

elicits a warning if the `Octave:num-to-str` warning is enabled. By default, the `Octave:num-to-str` warning is enabled.

`Octave:precedence-change`
If the `Octave:precedence-change` warning is enabled, Octave will warn about possible changes in the meaning of some code due to changes in precedence for some operators. Precedence changes have typically been made for MATLAB compatibility. By default, the `Octave:precedence-change` warning is enabled.

`Octave:reload-forces-clear`
If several functions have been loaded from the same file, Octave must clear all the functions before any one of them can be reloaded. If the `Octave:reload-forces-clear` warning is enabled, Octave will warn you when this happens, and print a list of the additional functions that it is forced to clear. By default, the `Octave:reload-forces-clear` warning is enabled.

`Octave:resize-on-range-error`
If the `Octave:resize-on-range-error` warning is enabled, print a warning when a matrix is resized by an indexed assignment with indices outside the current bounds. By default, the `Octave:resize-on-range-error` warning is disabled.

`Octave:separator-insert`
: Print warning if commas or semicolons might be inserted automatically in literal matrices.

`Octave:single-quote-string`
: Print warning if a single quote character is used to introduce a string constant.

`Octave:str-to-num`
: If the `Octave:str-to-num` warning is enabled, a warning is printed for implicit conversions of strings to their numeric ASCII equivalents. For example,

```
"abc" + 0
     ⇒ 97 98 99
```

elicits a warning if the `Octave:str-to-num` warning is enabled. By default, the `Octave:str-to-num` warning is disabled.

`Octave:string-concat`
: If the `Octave:string-concat` warning is enabled, print a warning when concatenating a mixture of double and single quoted strings. By default, the `Octave:string-concat` warning is disabled.

`Octave:undefined-return-values`
: If the `Octave:undefined-return-values` warning is disabled, print a warning if a function does not define all the values in the return list which are expected. By default, the `Octave:undefined-return-values` warning is enabled.

`Octave:variable-switch-label`
: If the `Octave:variable-switch-label` warning is enabled, Octave will print a warning if a switch label is not a constant or constant expression. By default, the `Octave:variable-switch-label` warning is disabled.

13 Debugging

Octave includes a built-in debugger to aid in the development of scripts. This can be used to interrupt the execution of an Octave script at a certain point, or when certain conditions are met. Once execution has stopped, and debug mode is entered, the symbol table at the point where execution has stopped can be examined and modified to check for errors.

The normal command-line editing and history functions are available in debug mode.

13.1 Entering Debug Mode

There are two basic means of interrupting the execution of an Octave script. These are breakpoints see Section 13.3 [Breakpoints], page 172, discussed in the next section and interruption based on some condition.

Octave supports three means to stop execution based on the values set in the functions `debug_on_interrupt`, `debug_on_warning` and `debug_on_error`.

val = `debug_on_interrupt ()` [Built-in Function]
old_val = `debug_on_interrupt (`*new_val*`)` [Built-in Function]

Query or set the internal variable that controls whether Octave will try to enter debugging mode when it receives an interrupt signal (typically generated with *C-c*). If a second interrupt signal is received before reaching the debugging mode, a normal interrupt will occur.

val = `debug_on_warning ()` [Built-in Function]
old_val = `debug_on_warning (`*new_val*`)` [Built-in Function]

Query or set the internal variable that controls whether Octave will try to enter the debugger when a warning is encountered.

val = `debug_on_error ()` [Built-in Function]
old_val = `debug_on_error (`*new_val*`)` [Built-in Function]

Query or set the internal variable that controls whether Octave will try to enter the debugger when an error is encountered. This will also inhibit printing of the normal traceback message (you will only see the top-level error message).

13.2 Leavinging Debug Mode

To leave the debug mode, use either `dbcont` or `return`.

`dbcont ()` [Command]

In debugging mode, quit debugging mode and continue execution.

See also: [dbstep], page 174, [dbstep], page 174.

To quit debug mode and return directly to the prompt `dbquit` should be used instead

`dbquit ()` [Command]

In debugging mode, quit debugging mode and return to the top level.

See also: [dbstep], page 174, [dbcont], page 171.

Finally, typing `exit` or `quit` at the debug prompt will result in Octave terminating normally.

13.3 Breakpoints

Breakpoints can be set in any Octave function, using the `dbstop` function.

rline = dbstop (*func*, *line*, . . .) [Loadable Function]

Set a breakpoint in a function

`func` String representing the function name. When already in debug mode this should be left out and only the line should be given.

`line` Line number you would like the breakpoint to be set on. Multiple lines might be given as separate arguments or as a vector.

The rline returned is the real line that the breakpoint was set at.

See also: [dbclear], page 172, [dbstatus], page 172, [dbstep], page 174.

Note that breakpoints cannot be set in built-in functions (e.g., `sin`, etc.) or dynamically loaded function (i.e., oct-files). To set a breakpoint immediately on entering a function, the breakpoint should be set to line 1. The leading comment block will be ignored and the breakpoint will be set to the first executable statement in the function. For example

```
dbstop ("asind", 1)
⇒ 27
```

Note that the return value of `27` means that the breakpoint was effectively set to line 27. The status of breakpoints in a function can be queried with the `dbstatus` function.

lst = dbstatus (*func*) [Loadable Function]

Return a vector containing the lines on which a function has breakpoints set.

`func` String representing the function name. When already in debug mode this should be left out.

See also: [dbclear], page 172, [dbwhere], page 173.

Taking the above as an example, `dbstatus ("asind")` should return 27. The breakpoints can then be cleared with the `dbclear` function

dbclear (*func*, *line*, . . .) [Loadable Function]

Delete a breakpoint in a function

`func` String representing the function name. When already in debug mode this should be left out and only the line should be given.

`line` Line number where you would like to remove the breakpoint. Multiple lines might be given as separate arguments or as a vector.

No checking is done to make sure that the line you requested is really a breakpoint. If you get the wrong line nothing will happen.

See also: [dbstop], page 172, [dbstatus], page 172, [dbwhere], page 173.

These functions can be used to clear all the breakpoints in a function. For example,

```
dbclear ("asind", dbstatus ("asind"));
```

A breakpoint can be set in a subfunction. For example if a file contains the functions

```
function y = func1 (x)
  y = func2 (x);
endfunction
function y = func2 (x)
  y = x + 1;
endfunction
```

then a breakpoint can be set at the start of the subfunction directly with

```
dbstop (["func1", filemarker(), "func2"])
⇒ 5
```

Note that `filemarker` returns a character that marks the subfunctions from the file containing them.

Another simple way of setting a breakpoint in an Octave script is the use of the `keyboard` function.

`keyboard` () [Built-in Function]
`keyboard` (*prompt*) [Built-in Function]

This function is normally used for simple debugging. When the `keyboard` function is executed, Octave prints a prompt and waits for user input. The input strings are then evaluated and the results are printed. This makes it possible to examine the values of variables within a function, and to assign new values if necessary. To leave the prompt and return to normal execution type '`return`' or '`dbcont`'. The `keyboard` function does not return an exit status.

If `keyboard` is invoked without arguments, a default prompt of '`debug> `' is used.

See also: [dbcont], page 171, [dbquit], page 171.

The `keyboard` function is typically placed in a script at the point where the user desires that the execution is stopped. It automatically sets the running script into the debug mode.

13.4 Debug Mode

There are two additional support functions that allow the user to interrogate where in the execution of a script Octave entered the debug mode and to print the code in the script surrounding the point where Octave entered debug mode.

`dbwhere` () [Loadable Function]

Show where we are in the code

See also: [dbclear], page 172, [dbstatus], page 172, [dbstop], page 172.

`dbtype` () [Loadable Function]

List script file with line numbers.

See also: [dbclear], page 172, [dbstatus], page 172, [dbstop], page 172.

You may also use `isdebugmode` to determine whether the debugger is currently active.

`isdebugmode` () [Command]

Return true if debug mode is on, otherwise false.

See also: [dbstack], page 174, [dbclear], page 172, [dbstop], page 172, [dbstatus], page 172.

Debug mode also allows single line stepping through a function using the commands `dbstep`.

`dbstep` *n* [Command]
`dbstep` *in* [Command]
`dbstep` *out* [Command]

In debugging mode, execute the next *n* lines of code. If *n* is omitted execute the next line of code. If the next line of code is itself defined in terms of an m-file remain in the existing function.

Using `dbstep in` will cause execution of the next line to step into any m-files defined on the next line. Using `dbstep out` with cause execution to continue until the current function returns.

See also: [dbcont], page 171, [dbquit], page 171.

13.5 Call Stack

`[`*stack*`,` *idx*`] dbstack (`*n*`)` [Loadable Function]

Print or return current stack information. With optional argument *n*, omit the *n* innermost stack frames.

See also: [dbclear], page 172, [dbstatus], page 172, [dbstop], page 172.

`dbup (`*n*`)` [Loadable Function]

In debugging mode, move up the execution stack *n* frames. If *n* is omitted, move up one frame.

See also: [dbstack], page 174.

`dbdown (`*n*`)` [Loadable Function]

In debugging mode, move down the execution stack *n* frames. If *n* is omitted, move down one frame.

See also: [dbstack], page 174.

14 Input and Output

Octave supports several ways of reading and writing data to or from the prompt or a file. The simplest functions for data Input and Output (I/O) are easy to use, but only provides limited control of how data is processed. For more control, a set of functions modelled after the C standard library are also provided by Octave.

14.1 Basic Input and Output

14.1.1 Terminal Output

Since Octave normally prints the value of an expression as soon as it has been evaluated, the simplest of all I/O functions is a simple expression. For example, the following expression will display the value of '`pi`'

```
pi
     ⊣ pi = 3.1416
```

This works well as long as it is acceptable to have the name of the variable (or '`ans`') printed along with the value. To print the value of a variable without printing its name, use the function `disp`.

The `format` command offers some control over the way Octave prints values with `disp` and through the normal echoing mechanism.

`disp` (*x*) [Built-in Function]

Display the value of *x*. For example,

```
disp ("The value of pi is:"), disp (pi)

     ⊣ the value of pi is:
     ⊣ 3.1416
```

Note that the output from `disp` always ends with a newline.

If an output value is requested, `disp` prints nothing and returns the formatted output in a string.

See also: [fdisp], page 184.

`format` [Command]
`format` *options* [Command]

Reset or specify the format of the output produced by `disp` and Octave's normal echoing mechanism. This command only affects the display of numbers but not how they are stored or computed. To change the internal representation from the default double use one of the conversion functions such as `single`, `uint8`, `int64`, etc.

By default, Octave displays 5 significant digits in a human readable form (option '`short`' paired with '`loose`' format for matrices). If `format` is invoked without any options, this default format is restored.

Valid formats for floating point numbers are listed in the following table.

`short` Fixed point format with 5 significant figures in a field that is a maximum of 10 characters wide. (default).

If Octave is unable to format a matrix so that columns line up on the decimal point and all numbers fit within the maximum field width then it switches to an exponential '`e`' format.

`long` Fixed point format with 15 significant figures in a field that is a maximum of 20 characters wide.

As with the '`short`' format, Octave will switch to an exponential '`e`' format if it is unable to format a matrix properly using the current format.

`short e`
`long e` Exponential format. The number to be represented is split between a mantissa and an exponent (power of 10). The mantissa has 5 significant digits in the short format and 15 digits in the long format. For example, with the '`short e`' format, `pi` is displayed as `3.1416e+00`.

`short E`
`long E` Identical to '`short e`' or '`long e`' but displays an uppercase '`E`' to indicate the exponent. For example, with the '`long E`' format, `pi` is displayed as `3.14159265358979E+00`.

`short g`
`long g` Optimally choose between fixed point and exponential format based on the magnitude of the number. For example, with the '`short g`' format, `pi .^ [2; 4; 8; 16; 32]` is displayed as

```
ans =

      9.8696
      97.409
      9488.5
  9.0032e+07
  8.1058e+15
```

`long G`
`short G` Identical to '`short g`' or '`long g`' but displays an uppercase '`E`' to indicate the exponent.

`free`
`none` Print output in free format, without trying to line up columns of matrices on the decimal point. This also causes complex numbers to be formatted as numeric pairs like this '`(0.60419, 0.60709)`' instead of like this '`0.60419 + 0.60709i`'.

The following formats affect all numeric output (floating point and integer types).

`+`
`+ chars`
`plus`
`plus chars`
Print a '`+`' symbol for nonzero matrix elements and a space for zero matrix elements. This format can be very useful for examining the structure of a large sparse matrix.

The optional argument *chars* specifies a list of 3 characters to use for printing values greater than zero, less than zero and equal to zero. For example, with the '`+ "+-."`' format, `[1, 0, -1; -1, 0, 1]` is displayed as

```
ans =

+.-
-.+
```

`bank` Print in a fixed format with two digits to the right of the decimal point.

`native-hex`
: Print the hexadecimal representation of numbers as they are stored in memory. For example, on a workstation which stores 8 byte real values in IEEE format with the least significant byte first, the value of `pi` when printed in `native-hex` format is `400921fb54442d18`.

`hex` The same as `native-hex`, but always print the most significant byte first.

`native-bit`
: Print the bit representation of numbers as stored in memory. For example, the value of `pi` is

```
01000000000010010010000111111011
01010100010001000010110100011000
```

(shown here in two 32 bit sections for typesetting purposes) when printed in native-bit format on a workstation which stores 8 byte real values in IEEE format with the least significant byte first.

`bit` The same as `native-bit`, but always print the most significant bits first.

`rat` Print a rational approximation, i.e., values are approximated as the ratio of small integers. For example, with the '`rat`' format, `pi` is displayed as `355/113`.

The following two options affect the display of all matrices.

`compact` Remove extra blank space around column number labels producing more compact output with more data per page.

`loose` Insert blank lines above and below column number labels to produce a more readable output with less data per page. (default).

14.1.1.1 Paging Screen Output

When running interactively, Octave normally sends any output intended for your terminal that is more than one screen long to a paging program, such as `less` or `more`. This avoids the problem of having a large volume of output stream by before you can read it. With `less` (and some versions of `more`) you can also scan forward and backward, and search for specific items.

Normally, no output is displayed by the pager until just before Octave is ready to print the top level prompt, or read from the standard input (for example, by using the `fscanf` or `scanf` functions). This means that there may be some delay before any output appears on

your screen if you have asked Octave to perform a significant amount of work with a single command statement. The function `fflush` may be used to force output to be sent to the pager (or any other stream) immediately.

You can select the program to run as the pager using the `PAGER` function, and you can turn paging off by using the function `more`.

`more` [Command]
`more` *on* [Command]
`more` *off* [Command]

Turn output pagination on or off. Without an argument, `more` toggles the current state. The current state can be determined via `page_screen_output`.

val = `PAGER ()` [Built-in Function]
old_val = `PAGER (`*new_val*`)` [Built-in Function]

Query or set the internal variable that specifies the program to use to display terminal output on your system. The default value is normally `"less"`, `"more"`, or `"pg"`, depending on what programs are installed on your system. See Appendix F [Installation], page 627.

See also: [more], page 178, [page_screen_output], page 178, [page_output_immediately], page 178, [PAGER_FLAGS], page 178.

val = `PAGER_FLAGS ()` [Built-in Function]
old_val = `PAGER_FLAGS (`*new_val*`)` [Built-in Function]

Query or set the internal variable that specifies the options to pass to the pager.

See also: [PAGER], page 178.

val = `page_screen_output ()` [Built-in Function]
old_val = `page_screen_output (`*new_val*`)` [Built-in Function]

Query or set the internal variable that controls whether output intended for the terminal window that is longer than one page is sent through a pager. This allows you to view one screenful at a time. Some pagers (such as `less`—see Appendix F [Installation], page 627) are also capable of moving backward on the output.

val = `page_output_immediately ()` [Built-in Function]
val = `page_output_immediately (`*new_val*`)` [Built-in Function]

Query or set the internal variable that controls whether Octave sends output to the pager as soon as it is available. Otherwise, Octave buffers its output and waits until just before the prompt is printed to flush it to the pager.

`fflush (`*fid*`)` [Built-in Function]

Flush output to *fid*. This is useful for ensuring that all pending output makes it to the screen before some other event occurs. For example, it is always a good idea to flush the standard output stream before calling `input`.

`fflush` returns 0 on success and an OS dependent error value (−1 on unix) on error.

See also: [fopen], page 188, [fclose], page 189.

14.1.2 Terminal Input

Octave has three functions that make it easy to prompt users for input. The `input` and `menu` functions are normally used for managing an interactive dialog with a user, and the `keyboard` function is normally used for doing simple debugging.

`input` (*prompt*) [Built-in Function]
`input` (*prompt*, "*s*") [Built-in Function]

Print a prompt and wait for user input. For example,

```
input ("Pick a number, any number! ")
```

prints the prompt

```
Pick a number, any number!
```

and waits for the user to enter a value. The string entered by the user is evaluated as an expression, so it may be a literal constant, a variable name, or any other valid expression.

Currently, `input` only returns one value, regardless of the number of values produced by the evaluation of the expression.

If you are only interested in getting a literal string value, you can call `input` with the character string `"s"` as the second argument. This tells Octave to return the string entered by the user directly, without evaluating it first.

Because there may be output waiting to be displayed by the pager, it is a good idea to always call `fflush (stdout)` before calling `input`. This will ensure that all pending output is written to the screen before your prompt. See Chapter 14 [Input and Output], page 175.

`menu` (*title*, *opt1*, ...) [Function File]

Print a title string followed by a series of options. Each option will be printed along with a number. The return value is the number of the option selected by the user. This function is useful for interactive programs. There is no limit to the number of options that may be passed in, but it may be confusing to present more than will fit easily on one screen.

See also: [disp], page 175, [printf], page 190, [input], page 179.

`yes_or_no` (*prompt*) [Built-in Function]

Ask the user a yes-or-no question. Return 1 if the answer is yes. Takes one argument, which is the string to display to ask the question. It should end in a space; '`yes-or-no-p`' adds '`(yes or no) `' to it. The user must confirm the answer with RET and can edit it until it has been confirmed.

For `input`, the normal command line history and editing functions are available at the prompt.

Octave also has a function that makes it possible to get a single character from the keyboard without requiring the user to type a carriage return.

`kbhit` () [Built-in Function]

Read a single keystroke from the keyboard. If called with one argument, don't wait for a keypress. For example,

```
x = kbhit ();
```

will set *x* to the next character typed at the keyboard as soon as it is typed.

```
x = kbhit (1);
```

identical to the above example, but don't wait for a keypress, returning the empty string if no key is available.

14.1.3 Simple File I/O

The `save` and `load` commands allow data to be written to and read from disk files in various formats. The default format of files written by the `save` command can be controlled using the functions `default_save_options` and `save_precision`.

As an example the following code creates a 3-by-3 matrix and saves it to the file '`myfile.mat`'.

```
A = [ 1:3; 4:6; 7:9 ];
save myfile.mat A
```

Once one or more variables have been saved to a file, they can be read into memory using the `load` command.

```
load myfile.mat
A
     ⊣ A =
     ⊣
     ⊣    1   2   3
     ⊣    4   5   6
     ⊣    7   8   9
```

`save` *file* [Command]
`save` *options file* [Command]
`save` *options file* `v1 v2` ... [Command]
`save` *options file -struct* `STRUCT f1 f2` ... [Command]

Save the named variables *v1*, *v2*, ..., in the file *file*. The special filename '`-`' may be used to write output to the terminal. If no variable names are listed, Octave saves all the variables in the current scope. Otherwise, full variable names or pattern syntax can be used to specify the variables to save. If the `-struct` modifier is used, fields *f1 f2* ... of the scalar structure *STRUCT* are saved as if they were variables with corresponding names. Valid options for the `save` command are listed in the following table. Options that modify the output format override the format specified by `default_save_options`.

If save is invoked using the functional form

```
save ("-option1", ..., "file", "v1", ...)
```

then the *options*, *file*, and variable name arguments (*v1*, ...) must be specified as character strings.

`-ascii` Save a single matrix in a text file without header or any other information.

`-binary` Save the data in Octave's binary data format.

`-float-binary`
: Save the data in Octave's binary data format but only using single precision. Only use this format if you know that all the values to be saved can be represented in single precision.

`-hdf5`
: Save the data in HDF5 format. (HDF5 is a free, portable binary format developed by the National Center for Supercomputing Applications at the University of Illinois.)

`-float-hdf5`
: Save the data in HDF5 format but only using single precision. Only use this format if you know that all the values to be saved can be represented in single precision.

`-V7`
`-v7`
`-7`
`-mat7-binary`
: Save the data in MATLAB's v7 binary data format.

`-V6`
`-v6`
`-6`
`-mat`
`-mat-binary`
: Save the data in MATLAB's v6 binary data format.

`-V4`
`-v4`
`-4`
`-mat4-binary`
: Save the data in the binary format written by MATLAB version 4.

`-text`
: Save the data in Octave's text data format. (default).

`-zip`
`-z`
: Use the gzip algorithm to compress the file. This works equally on files that are compressed with gzip outside of octave, and gzip can equally be used to convert the files for backward compatibility.

The list of variables to save may use wildcard patterns containing the following special characters:

`?`
: Match any single character.

`*`
: Match zero or more characters.

`[ list ]`
: Match the list of characters specified by *list*. If the first character is `!` or `^`, match all characters except those specified by *list*. For example, the pattern `[a-zA-Z]` will match all lower and upper case alphabetic characters.

: Wildcards may also be used in the field name specifications when using the `-struct` modifier (but not in the struct name itself).

Except when using the MATLAB binary data file format or the '-ascii' format, saving global variables also saves the global status of the variable. If the variable is restored at a later time using 'load', it will be restored as a global variable.

The command

```
save -binary data a b*
```

saves the variable 'a' and all variables beginning with 'b' to the file 'data' in Octave's binary format.

See also: [load], page 182, [default_save_options], page 183, [dlmread], page 185, [csvread], page 185, [fread], page 199.

`load` *file* [Command]
`load` *options file* [Command]
`load` *options file v1 v2 . . .* [Command]
`S` = *load*("*options*", "*file*", "*v1*", "*v2*", . . .) [Command]

Load the named variables *v1*, *v2*, . . ., from the file *file*. If no variables are specified then all variables found in the file will be loaded. As with `save`, the list of variables to extract can be full names or use a pattern syntax. The format of the file is automatically detected but may be overridden by supplying the appropriate option.

If load is invoked using the functional form

```
load ("-option1", ..., "file", "v1", ...)
```

then the *options*, *file*, and variable name arguments (*v1*, . . .) must be specified as character strings.

If a variable that is not marked as global is loaded from a file when a global symbol with the same name already exists, it is loaded in the global symbol table. Also, if a variable is marked as global in a file and a local symbol exists, the local symbol is moved to the global symbol table and given the value from the file.

If invoked with a single output argument, Octave returns data instead of inserting variables in the symbol table. If the data file contains only numbers (TAB- or space-delimited columns), a matrix of values is returned. Otherwise, `load` returns a structure with members corresponding to the names of the variables in the file.

The `load` command can read data stored in Octave's text and binary formats, and MATLAB's binary format. If compiled with zlib support, it can also load gzip-compressed files. It will automatically detect the type of file and do conversion from different floating point formats (currently only IEEE big and little endian, though other formats may be added in the future).

Valid options for `load` are listed in the following table.

`-force` This option is accepted for backward compatibility but is ignored. Octave now overwrites variables currently in memory with those of the same name found in the file.

`-ascii` Force Octave to assume the file contains columns of numbers in text format without any header or other information. Data in the file will be loaded as a single numeric matrix with the name of the variable derived from the name of the file.

`-binary` Force Octave to assume the file is in Octave's binary format.

`-hdf5` Force Octave to assume the file is in HDF5 format. (HDF5 is a free, portable binary format developed by the National Center for Supercomputing Applications at the University of Illinois.) Note that Octave can read HDF5 files not created by itself, but may skip some datasets in formats that it cannot support.

`-import` This option is accepted for backward compatibility but is ignored. Octave can now support multi-dimensional HDF data and automatically modifies variable names if they are invalid Octave identifiers.

`-mat`
`-mat-binary`
`-6`
`-v6`
`-7`
`-v7` Force Octave to assume the file is in MATLAB's version 6 or 7 binary format.

`-mat4-binary`
`-4`
`-v4`
`-V4` Force Octave to assume the file is in the binary format written by MATLAB version 4.

`-text` Force Octave to assume the file is in Octave's text format.

See also: [save], page 180, [dlmwrite], page 184, [csvwrite], page 185, [fwrite], page 201.

There are three functions that modify the behavior of `save`.

val = `default_save_options` () [Built-in Function]
old_val = `default_save_options` (*new_val*) [Built-in Function]

Query or set the internal variable that specifies the default options for the `save` command, and defines the default format. Typical values include `"-ascii"`, `"-text -zip"`. The default value is `-text`.

See also: [save], page 180.

val = `save_precision` () [Built-in Function]
old_val = `save_precision` (*new_val*) [Built-in Function]

Query or set the internal variable that specifies the number of digits to keep when saving data in text format.

val = `save_header_format_string` () [Built-in Function]
old_val = `save_header_format_string` (*new_val*) [Built-in Function]

Query or set the internal variable that specifies the format string used for the comment line written at the beginning of text-format data files saved by Octave. The format string is passed to `strftime` and should begin with the character '`#`' and contain no newline characters. If the value of `save_header_format_string` is the empty string, the header comment is omitted from text-format data files. The default value is

```
"# Created by Octave VERSION, %a %b %d %H:%M:%S %Y %Z <USER@HOST>"
```

See also: [strftime], page 517, [save], page 180.

native_float_format () [Built-in Function]

Return the native floating point format as a string

It is possible to write data to a file in a similar way to the `disp` function for writing data to the screen. The `fdisp` works just like `disp` except its first argument is a file pointer as created by `fopen`. As an example, the following code writes to data '`myfile.txt`'.

```
fid = fopen ("myfile.txt", "w");
fdisp (fid, "3/8 is ");
fdisp (fid, 3/8);
fclose (fid);
```

See Section 14.2.1 [Opening and Closing Files], page 188, for details on how to use `fopen` and `fclose`.

fdisp (*fid*, *x*) [Built-in Function]

Display the value of *x* on the stream *fid*. For example,

```
fdisp (stdout, "The value of pi is:"), fdisp (stdout, pi)

     ⊣ the value of pi is:
     ⊣ 3.1416
```

Note that the output from `fdisp` always ends with a newline.

See also: [disp], page 175.

Octave can also read and write matrices text files such as comma separated lists.

dlmwrite (*file*, *a*) [Function File]
dlmwrite (*file*, *a*, *delim*, *r*, *c*) [Function File]
dlmwrite (*file*, *a*, *key*, *val* . . .) [Function File]
dlmwrite (*file*, *a*, "*-append*", . . .) [Function File]

Write the matrix *a* to the named file using delimiters.

The parameter *delim* specifies the delimiter to use to separate values on a row.

The value of *r* specifies the number of delimiter-only lines to add to the start of the file.

The value of *c* specifies the number of delimiters to prepend to each line of data.

If the argument `"-append"` is given, append to the end of the *file*.

In addition, the following keyword value pairs may appear at the end of the argument list:

`"append"` Either '`"on"`' or '`"off"`'. See '`"-append"`' above.

`"delimiter"`
See *delim* above.

`"newline"`
The character(s) to use to separate each row. Three special cases exist for this option. '`"unix"`' is changed into '\n', '`"pc"`' is changed into '\r\n',

and '"mac"' is changed into '\r'. Other values for this option are kept as is.

"roffset"
: See *r* above.

"coffset"
: See *c* above.

"precision"
: The precision to use when writing the file. It can either be a format string (as used by fprintf) or a number of significant digits.

```
dlmwrite ("file.csv", reshape (1:16, 4, 4));
dlmwrite ("file.tex", a, "delimiter", "&", "newline", "\\n")
```

See also: [dlmread], page 185, [csvread], page 185, [csvwrite], page 185.

data = dlmread (*file*) [Loadable Function]
data = dlmread (*file*, *sep*) [Loadable Function]
data = dlmread (*file*, *sep*, *r0*, *c0*) [Loadable Function]
data = dlmread (*file*, *sep*, *range*) [Loadable Function]

Read the matrix *data* from a text file. If not defined the separator between fields is determined from the file itself. Otherwise the separation character is defined by *sep*.

Given two scalar arguments *r0* and *c0*, these define the starting row and column of the data to be read. These values are indexed from zero, such that the first row corresponds to an index of zero.

The *range* parameter must be a 4 element vector containing the upper left and lower right corner [*R0*,*C0*,*R1*,*C1*] or a spreadsheet style range such as 'A2..Q15'. The lowest index value is zero.

x = csvwrite (*filename*, *x*) [Function File]

Write the matrix *x* to a file.

This function is equivalent to

```
dlmwrite (filename, x, ",", ...)
```

See also: [dlmread], page 185, [dlmwrite], page 184, [csvread], page 185.

x = csvread (*filename*) [Function File]

Read the matrix *x* from a file.

This function is equivalent to

```
dlmread (filename, "," , ...)
```

See also: [dlmread], page 185, [dlmwrite], page 184, [csvwrite], page 185.

14.1.3.1 Saving Data on Unexpected Exits

If Octave for some reason exits unexpectedly it will by default save the variables available in the workspace to a file in the current directory. By default this file is named 'octave-core' and can be loaded into memory with the `load` command. While the default behavior most often is reasonable it can be changed through the following functions.

val = `crash_dumps_octave_core` () [Built-in Function]
old_val = `crash_dumps_octave_core` (*new_val*) [Built-in Function]

Query or set the internal variable that controls whether Octave tries to save all current variables to the file "octave-core" if it crashes or receives a hangup, terminate or similar signal.

See also: [octave_core_file_limit], page 186, [octave_core_file_name], page 186, [octave_core_file_options], page 186.

val = `sighup_dumps_octave_core` () [Built-in Function]
old_val = `sighup_dumps_octave_core` (*new_val*) [Built-in Function]

Query or set the internal variable that controls whether Octave tries to save all current variables to the file "octave-core" if it receives a hangup signal.

val = `sigterm_dumps_octave_core` () [Built-in Function]
old_val = `sigterm_dumps_octave_core` (*new_val*) [Built-in Function]

Query or set the internal variable that controls whether Octave tries to save all current variables to the file "octave-core" if it receives a terminate signal.

val = `octave_core_file_options` () [Built-in Function]
old_val = `octave_core_file_options` (*new_val*) [Built-in Function]

Query or set the internal variable that specifies the options used for saving the workspace data if Octave aborts. The value of `octave_core_file_options` should follow the same format as the options for the `save` function. The default value is Octave's binary format.

See also: [crash_dumps_octave_core], page 185, [octave_core_file_name], page 186, [octave_core_file_limit], page 186.

val = `octave_core_file_limit` () [Built-in Function]
old_val = `octave_core_file_limit` (*new_val*) [Built-in Function]

Query or set the internal variable that specifies the maximum amount of memory (in kilobytes) of the top-level workspace that Octave will attempt to save when writing data to the crash dump file (the name of the file is specified by *octave_core_file_name*). If *octave_core_file_options* flags specify a binary format, then *octave_core_file_limit* will be approximately the maximum size of the file. If a text file format is used, then the file could be much larger than the limit. The default value is -1 (unlimited)

See also: [crash_dumps_octave_core], page 185, [octave_core_file_name], page 186, [octave_core_file_options], page 186.

val = `octave_core_file_name` () [Built-in Function]
old_val = `octave_core_file_name` (*new_val*) [Built-in Function]

Query or set the internal variable that specifies the name of the file used for saving data from the top-level workspace if Octave aborts. The default value is `"octave-core"`

See also: [crash_dumps_octave_core], page 185, [octave_core_file_name], page 186, [octave_core_file_options], page 186.

14.1.4 Rational Approximations

s = rat (*x*, *tol*) [Function File]
[*n*, *d*] = rat (*x*, *tol*) [Function File]

Find a rational approximation to *x* within the tolerance defined by *tol* using a continued fraction expansion. For example,

```
rat(pi) = 3 + 1/(7 + 1/16) = 355/113
rat(e) = 3 + 1/(-4 + 1/(2 + 1/(5 + 1/(-2 + 1/(-7)))))
       = 1457/536
```

Called with two arguments returns the numerator and denominator separately as two matrices.

See also: [rats], page 187.

rats (*x*, *len*) [Built-in Function]

Convert *x* into a rational approximation represented as a string. You can convert the string back into a matrix as follows:

```
r = rats(hilb(4));
x = str2num(r)
```

The optional second argument defines the maximum length of the string representing the elements of *x*. By default *len* is 9.

See also: [format], page 175, [rat], page 187.

14.2 C-Style I/O Functions

Octave's C-style input and output functions provide most of the functionality of the C programming language's standard I/O library. The argument lists for some of the input functions are slightly different, however, because Octave has no way of passing arguments by reference.

In the following, *file* refers to a file name and `fid` refers to an integer file number, as returned by `fopen`.

There are three files that are always available. Although these files can be accessed using their corresponding numeric file ids, you should always use the symbolic names given in the table below, since it will make your programs easier to understand.

stdin () [Built-in Function]

Return the numeric value corresponding to the standard input stream. When Octave is used interactively, this is filtered through the command line editing functions.

See also: [stdout], page 187, [stderr], page 188.

stdout () [Built-in Function]

Return the numeric value corresponding to the standard output stream. Data written to the standard output is normally filtered through the pager.

See also: [stdin], page 187, [stderr], page 188.

`stderr ()` [Built-in Function]

Return the numeric value corresponding to the standard error stream. Even if paging is turned on, the standard error is not sent to the pager. It is useful for error messages and prompts.

See also: [stdin], page 187, [stdout], page 187.

14.2.1 Opening and Closing Files

When reading data from a file it must be opened for reading first, and likewise when writing to a file. The `fopen` function returns a pointer to an open file that is ready to be read or written. Once all data has been read from or written to the opened file it should be closed. The `fclose` function does this. The following code illustrates the basic pattern for writing to a file, but a very similar pattern is used when reading a file.

```
filename = "myfile.txt";
fid = fopen (filename, "w");
# Do the actual I/O here...
fclose (fid);
```

`[`*fid*`, `*msg*`] = fopen (`*name*`, `*mode*`, `*arch*`)` [Built-in Function]
fid_list `= fopen ("`*all*`")` [Built-in Function]
`[`*file*`, `*mode*`, `*arch*`] = fopen (`*fid*`)` [Built-in Function]

The first form of the `fopen` function opens the named file with the specified mode (read-write, read-only, etc.) and architecture interpretation (IEEE big endian, IEEE little endian, etc.), and returns an integer value that may be used to refer to the file later. If an error occurs, *fid* is set to −1 and *msg* contains the corresponding system error message. The *mode* is a one or two character string that specifies whether the file is to be opened for reading, writing, or both.

The second form of the `fopen` function returns a vector of file ids corresponding to all the currently open files, excluding the `stdin`, `stdout`, and `stderr` streams.

The third form of the `fopen` function returns information about the open file given its file id.

For example,

```
myfile = fopen ("splat.dat", "r", "ieee-le");
```

opens the file '`splat.dat`' for reading. If necessary, binary numeric values will be read assuming they are stored in IEEE format with the least significant bit first, and then converted to the native representation.

Opening a file that is already open simply opens it again and returns a separate file id. It is not an error to open a file several times, though writing to the same file through several different file ids may produce unexpected results.

The possible values '`mode`' may have are

'`r`' Open a file for reading.

'`w`' Open a file for writing. The previous contents are discarded.

'`a`' Open or create a file for writing at the end of the file.

'`r+`' Open an existing file for reading and writing.

'w+' Open a file for reading or writing. The previous contents are discarded.

'a+' Open or create a file for reading or writing at the end of the file.

Append a "t" to the mode string to open the file in text mode or a "b" to open in binary mode. On Windows and Macintosh systems, text mode reading and writing automatically converts linefeeds to the appropriate line end character for the system (carriage-return linefeed on Windows, carriage-return on Macintosh). The default if no mode is specified is binary mode.

Additionally, you may append a "z" to the mode string to open a gzipped file for reading or writing. For this to be successful, you must also open the file in binary mode.

The parameter *arch* is a string specifying the default data format for the file. Valid values for *arch* are:

'native' The format of the current machine (this is the default).

'ieee-be' IEEE big endian format.

'ieee-le' IEEE little endian format.

'vaxd' VAX D floating format.

'vaxg' VAX G floating format.

'cray' Cray floating format.

however, conversions are currently only supported for 'native' 'ieee-be', and 'ieee-le' formats.

See also: [fclose], page 189, [fgets], page 190, [fputs], page 189, [fread], page 199, [fseek], page 203, [ferror], page 203, [fprintf], page 191, [fscanf], page 195, [ftell], page 203, [fwrite], page 201.

`fclose (fid)` [Built-in Function]

Closes the specified file. If successful, `fclose` returns 0, otherwise, it returns -1.

See also: [fopen], page 188, [fseek], page 203, [ftell], page 203.

14.2.2 Simple Output

Once a file has been opened for writing a string can be written to the file using the `fputs` function. The following example shows how to write the string 'Free Software is needed for Free Science' to the file 'free.txt'.

```
filename = "free.txt";
fid = fopen (filename, "w");
fputs (fid, "Free Software is needed for Free Science");
fclose (fid);
```

`fputs (fid, string)` [Built-in Function]

Write a string to a file with no formatting.

Return a non-negative number on success and EOF on error.

See also: [scanf], page 196, [sscanf], page 196, [fread], page 199, [fprintf], page 191, [fgets], page 190, [fscanf], page 195.

A function much similar to `fputs` is available for writing data to the screen. The `puts` function works just like `fputs` except it doesn't take a file pointer as its input.

`puts` (*string*) [Built-in Function]

Write a string to the standard output with no formatting.

Return a non-negative number on success and EOF on error.

14.2.3 Line-Oriented Input

To read from a file it must be opened for reading using `fopen`. Then a line can be read from the file using `fgetl` as the following code illustrates

```
fid = fopen ("free.txt");
txt = fgetl (fid)
     ⊣ Free Software is needed for Free Science
fclose (fid);
```

This of course assumes that the file '`free.txt`' exists and contains the line '`Free Software is needed for Free Science`'.

`fgetl` (*fid*, *len*) [Built-in Function]

Read characters from a file, stopping after a newline, or EOF, or *len* characters have been read. The characters read, excluding the possible trailing newline, are returned as a string.

If *len* is omitted, `fgetl` reads until the next newline character.

If there are no more characters to read, `fgetl` returns −1.

See also: [fread], page 199, [fscanf], page 195.

`fgets` (*fid*, *len*) [Built-in Function]

Read characters from a file, stopping after a newline, or EOF, or *len* characters have been read. The characters read, including the possible trailing newline, are returned as a string.

If *len* is omitted, `fgets` reads until the next newline character.

If there are no more characters to read, `fgets` returns −1.

See also: [fputs], page 189, [fopen], page 188, [fread], page 199, [fscanf], page 195.

14.2.4 Formatted Output

This section describes how to call `printf` and related functions.

The following functions are available for formatted output. They are modelled after the C language functions of the same name, but they interpret the format template differently in order to improve the performance of printing vector and matrix values.

`printf` (*template*, ...) [Built-in Function]

Print optional arguments under the control of the template string *template* to the stream `stdout` and return the number of characters printed.

See also: [fprintf], page 191, [sprintf], page 191, [scanf], page 196.

fprintf (*fid*, *template*, ...) [Built-in Function]

This function is just like `printf`, except that the output is written to the stream *fid* instead of `stdout`. If *fid* is omitted, the output is written to `stdout`.

See also: [printf], page 190, [sprintf], page 191, [fread], page 199, [fscanf], page 195, [fopen], page 188, [fclose], page 189.

sprintf (*template*, ...) [Built-in Function]

This is like `printf`, except that the output is returned as a string. Unlike the C library function, which requires you to provide a suitably sized string as an argument, Octave's `sprintf` function returns the string, automatically sized to hold all of the items converted.

See also: [printf], page 190, [fprintf], page 191, [sscanf], page 196.

The `printf` function can be used to print any number of arguments. The template string argument you supply in a call provides information not only about the number of additional arguments, but also about their types and what style should be used for printing them.

Ordinary characters in the template string are simply written to the output stream as-is, while *conversion specifications* introduced by a '%' character in the template cause subsequent arguments to be formatted and written to the output stream. For example,

```
pct = 37;
filename = "foo.txt";
printf ("Processed %d%% of '%s'.\nPlease be patient.\n",
        pct, filename);
```

produces output like

```
Processed 37% of 'foo.txt'.
Please be patient.
```

This example shows the use of the '%d' conversion to specify that a scalar argument should be printed in decimal notation, the '%s' conversion to specify printing of a string argument, and the '%%' conversion to print a literal '%' character.

There are also conversions for printing an integer argument as an unsigned value in octal, decimal, or hexadecimal radix ('%o', '%u', or '%x', respectively); or as a character value ('%c').

Floating-point numbers can be printed in normal, fixed-point notation using the '%f' conversion or in exponential notation using the '%e' conversion. The '%g' conversion uses either '%e' or '%f' format, depending on what is more appropriate for the magnitude of the particular number.

You can control formatting more precisely by writing *modifiers* between the '%' and the character that indicates which conversion to apply. These slightly alter the ordinary behavior of the conversion. For example, most conversion specifications permit you to specify a minimum field width and a flag indicating whether you want the result left- or right-justified within the field.

The specific flags and modifiers that are permitted and their interpretation vary depending on the particular conversion. They're all described in more detail in the following sections.

14.2.5 Output Conversion for Matrices

When given a matrix value, Octave's formatted output functions cycle through the format template until all the values in the matrix have been printed. For example,

```
printf ("%4.2f %10.2e %8.4g\n", hilb (3));

     ⊣ 1.00   5.00e-01   0.3333
     ⊣ 0.50   3.33e-01     0.25
     ⊣ 0.33   2.50e-01      0.2
```

If more than one value is to be printed in a single call, the output functions do not return to the beginning of the format template when moving on from one value to the next. This can lead to confusing output if the number of elements in the matrices are not exact multiples of the number of conversions in the format template. For example,

```
printf ("%4.2f %10.2e %8.4g\n", [1, 2], [3, 4]);

     ⊣ 1.00   2.00e+00        3
     ⊣ 4.00
```

If this is not what you want, use a series of calls instead of just one.

14.2.6 Output Conversion Syntax

This section provides details about the precise syntax of conversion specifications that can appear in a `printf` template string.

Characters in the template string that are not part of a conversion specification are printed as-is to the output stream.

The conversion specifications in a `printf` template string have the general form:

% *flags width* [. *precision*] *type conversion*

For example, in the conversion specifier '`%-10.8ld`', the '`-`' is a flag, '`10`' specifies the field width, the precision is '`8`', the letter '`l`' is a type modifier, and '`d`' specifies the conversion style. (This particular type specifier says to print a numeric argument in decimal notation, with a minimum of 8 digits left-justified in a field at least 10 characters wide.)

In more detail, output conversion specifications consist of an initial '`%`' character followed in sequence by:

- Zero or more *flag characters* that modify the normal behavior of the conversion specification.
- An optional decimal integer specifying the *minimum field width*. If the normal conversion produces fewer characters than this, the field is padded with spaces to the specified width. This is a *minimum* value; if the normal conversion produces more characters than this, the field is *not* truncated. Normally, the output is right-justified within the field.

  You can also specify a field width of '`*`'. This means that the next argument in the argument list (before the actual value to be printed) is used as the field width. The value is rounded to the nearest integer. If the value is negative, this means to set the '`-`' flag (see below) and to use the absolute value as the field width.

- An optional *precision* to specify the number of digits to be written for the numeric conversions. If the precision is specified, it consists of a period ('.') followed optionally by a decimal integer (which defaults to zero if omitted).

  You can also specify a precision of '*'. This means that the next argument in the argument list (before the actual value to be printed) is used as the precision. The value must be an integer, and is ignored if it is negative.
- An optional *type modifier character*. This character is ignored by Octave's `printf` function, but is recognized to provide compatibility with the C language `printf`.
- A character that specifies the conversion to be applied.

The exact options that are permitted and how they are interpreted vary between the different conversion specifiers. See the descriptions of the individual conversions for information about the particular options that they use.

14.2.7 Table of Output Conversions

Here is a table summarizing what all the different conversions do:

'%d', '%i' Print an integer as a signed decimal number. See Section 14.2.8 [Integer Conversions], page 194, for details. '%d' and '%i' are synonymous for output, but are different when used with `scanf` for input (see Section 14.2.13 [Table of Input Conversions], page 197).

'%o' Print an integer as an unsigned octal number. See Section 14.2.8 [Integer Conversions], page 194, for details.

'%u' Print an integer as an unsigned decimal number. See Section 14.2.8 [Integer Conversions], page 194, for details.

'%x', '%X' Print an integer as an unsigned hexadecimal number. '%x' uses lower-case letters and '%X' uses upper-case. See Section 14.2.8 [Integer Conversions], page 194, for details.

'%f' Print a floating-point number in normal (fixed-point) notation. See Section 14.2.9 [Floating-Point Conversions], page 194, for details.

'%e', '%E' Print a floating-point number in exponential notation. '%e' uses lower-case letters and '%E' uses upper-case. See Section 14.2.9 [Floating-Point Conversions], page 194, for details.

'%g', '%G' Print a floating-point number in either normal (fixed-point) or exponential notation, whichever is more appropriate for its magnitude. '%g' uses lower-case letters and '%G' uses upper-case. See Section 14.2.9 [Floating-Point Conversions], page 194, for details.

'%c' Print a single character. See Section 14.2.10 [Other Output Conversions], page 195.

'%s' Print a string. See Section 14.2.10 [Other Output Conversions], page 195.

'%%' Print a literal '%' character. See Section 14.2.10 [Other Output Conversions], page 195.

If the syntax of a conversion specification is invalid, unpredictable things will happen, so don't do this. If there aren't enough function arguments provided to supply values for all the conversion specifications in the template string, or if the arguments are not of the correct types, the results are unpredictable. If you supply more arguments than conversion specifications, the extra argument values are simply ignored; this is sometimes useful.

14.2.8 Integer Conversions

This section describes the options for the '%d', '%i', '%o', '%u', '%x', and '%X' conversion specifications. These conversions print integers in various formats.

The '%d' and '%i' conversion specifications both print an numeric argument as a signed decimal number; while '%o', '%u', and '%x' print the argument as an unsigned octal, decimal, or hexadecimal number (respectively). The '%X' conversion specification is just like '%x' except that it uses the characters 'ABCDEF' as digits instead of 'abcdef'.

The following flags are meaningful:

'-' Left-justify the result in the field (instead of the normal right-justification).

'+' For the signed '%d' and '%i' conversions, print a plus sign if the value is positive.

' ' For the signed '%d' and '%i' conversions, if the result doesn't start with a plus or minus sign, prefix it with a space character instead. Since the '+' flag ensures that the result includes a sign, this flag is ignored if you supply both of them.

'#' For the '%o' conversion, this forces the leading digit to be '0', as if by increasing the precision. For '%x' or '%X', this prefixes a leading '0x' or '0X' (respectively) to the result. This doesn't do anything useful for the '%d', '%i', or '%u' conversions.

'0' Pad the field with zeros instead of spaces. The zeros are placed after any indication of sign or base. This flag is ignored if the '-' flag is also specified, or if a precision is specified.

If a precision is supplied, it specifies the minimum number of digits to appear; leading zeros are produced if necessary. If you don't specify a precision, the number is printed with as many digits as it needs. If you convert a value of zero with an explicit precision of zero, then no characters at all are produced.

14.2.9 Floating-Point Conversions

This section discusses the conversion specifications for floating-point numbers: the '%f', '%e', '%E', '%g', and '%G' conversions.

The '%f' conversion prints its argument in fixed-point notation, producing output of the form [-]*ddd*.*ddd*, where the number of digits following the decimal point is controlled by the precision you specify.

The '%e' conversion prints its argument in exponential notation, producing output of the form [-]*d*.*ddd*e[+|-]*dd*. Again, the number of digits following the decimal point is controlled by the precision. The exponent always contains at least two digits. The '%E' conversion is similar but the exponent is marked with the letter 'E' instead of 'e'.

The '%g' and '%G' conversions print the argument in the style of '%e' or '%E' (respectively) if the exponent would be less than -4 or greater than or equal to the precision; otherwise

they use the '%f' style. Trailing zeros are removed from the fractional portion of the result and a decimal-point character appears only if it is followed by a digit.

The following flags can be used to modify the behavior:

'-' Left-justify the result in the field. Normally the result is right-justified.

'+' Always include a plus or minus sign in the result.

' ' If the result doesn't start with a plus or minus sign, prefix it with a space instead. Since the '+' flag ensures that the result includes a sign, this flag is ignored if you supply both of them.

'#' Specifies that the result should always include a decimal point, even if no digits follow it. For the '%g' and '%G' conversions, this also forces trailing zeros after the decimal point to be left in place where they would otherwise be removed.

'0' Pad the field with zeros instead of spaces; the zeros are placed after any sign. This flag is ignored if the '-' flag is also specified.

The precision specifies how many digits follow the decimal-point character for the '%f', '%e', and '%E' conversions. For these conversions, the default precision is 6. If the precision is explicitly 0, this suppresses the decimal point character entirely. For the '%g' and '%G' conversions, the precision specifies how many significant digits to print. Significant digits are the first digit before the decimal point, and all the digits after it. If the precision is 0 or not specified for '%g' or '%G', it is treated like a value of 1. If the value being printed cannot be expressed precisely in the specified number of digits, the value is rounded to the nearest number that fits.

14.2.10 Other Output Conversions

This section describes miscellaneous conversions for printf.

The '%c' conversion prints a single character. The '-' flag can be used to specify left-justification in the field, but no other flags are defined, and no precision or type modifier can be given. For example:

```
printf ("%c%c%c%c%c", "h", "e", "l", "l", "o");
```

prints 'hello'.

The '%s' conversion prints a string. The corresponding argument must be a string. A precision can be specified to indicate the maximum number of characters to write; otherwise characters in the string up to but not including the terminating null character are written to the output stream. The '-' flag can be used to specify left-justification in the field, but no other flags or type modifiers are defined for this conversion. For example:

```
printf ("%3s%-6s", "no", "where");
```

prints ' nowhere ' (note the leading and trailing spaces).

14.2.11 Formatted Input

Octave provides the scanf, fscanf, and sscanf functions to read formatted input. There are two forms of each of these functions. One can be used to extract vectors of data from a file, and the other is more 'C-like'.

`[val, count] = fscanf (fid, template, size)` [Built-in Function]
`[v1, v2, ..., count] = fscanf (fid, template, "C")` [Built-in Function]

In the first form, read from *fid* according to *template*, returning the result in the matrix *val*.

The optional argument *size* specifies the amount of data to read and may be one of

`Inf` Read as much as possible, returning a column vector.

`nr` Read up to *nr* elements, returning a column vector.

`[nr, Inf]` Read as much as possible, returning a matrix with *nr* rows. If the number of elements read is not an exact multiple of *nr*, the last column is padded with zeros.

`[nr, nc]` Read up to `nr * nc` elements, returning a matrix with *nr* rows. If the number of elements read is not an exact multiple of *nr*, the last column is padded with zeros.

If *size* is omitted, a value of `Inf` is assumed.

A string is returned if *template* specifies only character conversions.

The number of items successfully read is returned in *count*.

In the second form, read from *fid* according to *template*, with each conversion specifier in *template* corresponding to a single scalar return value. This form is more 'C-like', and also compatible with previous versions of Octave. The number of successful conversions is returned in *count*

See also: [scanf], page 196, [sscanf], page 196, [fread], page 199, [fprintf], page 191, [fgets], page 190, [fputs], page 189.

`[val, count] = scanf (template, size)` [Built-in Function]
`[v1, v2, ..., count]] = scanf (template, "C")` [Built-in Function]

This is equivalent to calling `fscanf` with *fid* = `stdin`.

It is currently not useful to call `scanf` in interactive programs.

See also: [fscanf], page 195, [sscanf], page 196, [printf], page 190.

`[val, count] = sscanf (string, template, size)` [Built-in Function]
`[v1, v2, ..., count] = sscanf (string, template, "C")` [Built-in Function]

This is like `fscanf`, except that the characters are taken from the string *string* instead of from a stream. Reaching the end of the string is treated as an end-of-file condition.

See also: [fscanf], page 195, [scanf], page 196, [sprintf], page 191.

Calls to `scanf` are superficially similar to calls to `printf` in that arbitrary arguments are read under the control of a template string. While the syntax of the conversion specifications in the template is very similar to that for `printf`, the interpretation of the template is oriented more towards free-format input and simple pattern matching, rather than fixed-field formatting. For example, most `scanf` conversions skip over any amount of "white space" (including spaces, tabs, and newlines) in the input file, and there is no concept of precision for the numeric input conversions as there is for the corresponding output conversions. Ordinarily, non-whitespace characters in the template are expected to match characters in the input stream exactly.

When a *matching failure* occurs, `scanf` returns immediately, leaving the first non-matching character as the next character to be read from the stream, and `scanf` returns all the items that were successfully converted.

The formatted input functions are not used as frequently as the formatted output functions. Partly, this is because it takes some care to use them properly. Another reason is that it is difficult to recover from a matching error.

14.2.12 Input Conversion Syntax

A `scanf` template string is a string that contains ordinary multibyte characters interspersed with conversion specifications that start with '`%`'.

Any whitespace character in the template causes any number of whitespace characters in the input stream to be read and discarded. The whitespace characters that are matched need not be exactly the same whitespace characters that appear in the template string. For example, write '` , `' in the template to recognize a comma with optional whitespace before and after.

Other characters in the template string that are not part of conversion specifications must match characters in the input stream exactly; if this is not the case, a matching failure occurs.

The conversion specifications in a `scanf` template string have the general form:

```
% flags width type conversion
```

In more detail, an input conversion specification consists of an initial '`%`' character followed in sequence by:

- An optional *flag character* '`*`', which says to ignore the text read for this specification. When `scanf` finds a conversion specification that uses this flag, it reads input as directed by the rest of the conversion specification, but it discards this input, does not return any value, and does not increment the count of successful assignments.
- An optional decimal integer that specifies the *maximum field width*. Reading of characters from the input stream stops either when this maximum is reached or when a non-matching character is found, whichever happens first. Most conversions discard initial whitespace characters, and these discarded characters don't count towards the maximum field width. Conversions that do not discard initial whitespace are explicitly documented.
- An optional type modifier character. This character is ignored by Octave's `scanf` function, but is recognized to provide compatibility with the C language `scanf`.
- A character that specifies the conversion to be applied.

The exact options that are permitted and how they are interpreted vary between the different conversion specifiers. See the descriptions of the individual conversions for information about the particular options that they allow.

14.2.13 Table of Input Conversions

Here is a table that summarizes the various conversion specifications:

'`%d`' Matches an optionally signed integer written in decimal. See Section 14.2.14 [Numeric Input Conversions], page 198.

'%i' Matches an optionally signed integer in any of the formats that the C language defines for specifying an integer constant. See Section 14.2.14 [Numeric Input Conversions], page 198.

'%o' Matches an unsigned integer written in octal radix. See Section 14.2.14 [Numeric Input Conversions], page 198.

'%u' Matches an unsigned integer written in decimal radix. See Section 14.2.14 [Numeric Input Conversions], page 198.

'%x', '%X' Matches an unsigned integer written in hexadecimal radix. See Section 14.2.14 [Numeric Input Conversions], page 198.

'%e', '%f', '%g', '%E', '%G'
Matches an optionally signed floating-point number. See Section 14.2.14 [Numeric Input Conversions], page 198.

'%s' Matches a string containing only non-whitespace characters. See Section 14.2.15 [String Input Conversions], page 198.

'%c' Matches a string of one or more characters; the number of characters read is controlled by the maximum field width given for the conversion. See Section 14.2.15 [String Input Conversions], page 198.

'%%' This matches a literal '%' character in the input stream. No corresponding argument is used.

If the syntax of a conversion specification is invalid, the behavior is undefined. If there aren't enough function arguments provided to supply addresses for all the conversion specifications in the template strings that perform assignments, or if the arguments are not of the correct types, the behavior is also undefined. On the other hand, extra arguments are simply ignored.

14.2.14 Numeric Input Conversions

This section describes the `scanf` conversions for reading numeric values.

The '%d' conversion matches an optionally signed integer in decimal radix.

The '%i' conversion matches an optionally signed integer in any of the formats that the C language defines for specifying an integer constant.

For example, any of the strings '10', '0xa', or '012' could be read in as integers under the '%i' conversion. Each of these specifies a number with decimal value `10`.

The '%o', '%u', and '%x' conversions match unsigned integers in octal, decimal, and hexadecimal radices, respectively.

The '%X' conversion is identical to the '%x' conversion. They both permit either uppercase or lowercase letters to be used as digits.

Unlike the C language `scanf`, Octave ignores the 'h', 'l', and 'L' modifiers.

14.2.15 String Input Conversions

This section describes the `scanf` input conversions for reading string and character values: '%s' and '%c'.

The '%c' conversion is the simplest: it matches a fixed number of characters, always. The maximum field with says how many characters to read; if you don't specify the maximum, the default is 1. This conversion does not skip over initial whitespace characters. It reads precisely the next *n* characters, and fails if it cannot get that many.

The '%s' conversion matches a string of non-whitespace characters. It skips and discards initial whitespace, but stops when it encounters more whitespace after having read something.

For example, reading the input:

```
hello, world
```

with the conversion '%10c' produces `" hello, wo"`, but reading the same input with the conversion '%10s' produces `"hello,"`.

14.2.16 Binary I/O

Octave can read and write binary data using the functions `fread` and `fwrite`, which are patterned after the standard C functions with the same names. They are able to automatically swap the byte order of integer data and convert among the supported floating point formats as the data are read.

`[val, count] = fread (fid, size, precision, skip, arch)` [Built-in Function]

Read binary data of type *precision* from the specified file ID *fid*.

The optional argument *size* specifies the amount of data to read and may be one of

`Inf` Read as much as possible, returning a column vector.

nr Read up to *nr* elements, returning a column vector.

`[nr, Inf]` Read as much as possible, returning a matrix with *nr* rows. If the number of elements read is not an exact multiple of *nr*, the last column is padded with zeros.

`[nr, nc]` Read up to `nr * nc` elements, returning a matrix with *nr* rows. If the number of elements read is not an exact multiple of *nr*, the last column is padded with zeros.

If *size* is omitted, a value of `Inf` is assumed.

The optional argument *precision* is a string specifying the type of data to read and may be one of

`"schar"`
`"signed char"`
Signed character.

`"uchar"`
`"unsigned char"`
Unsigned character.

`"int8"`
`"integer*1"`
8-bit signed integer.

"int16"
"integer*2"
: 16-bit signed integer.

"int32"
"integer*4"
: 32-bit signed integer.

"int64"
"integer*8"
: 64-bit signed integer.

"uint8" 8-bit unsigned integer.

"uint16" 16-bit unsigned integer.

"uint32" 32-bit unsigned integer.

"uint64" 64-bit unsigned integer.

"single"
"float32"
"real*4" 32-bit floating point number.

"double"
"float64"
"real*8" 64-bit floating point number.

"char"
"char*1" Single character.

"short" Short integer (size is platform dependent).

"int" Integer (size is platform dependent).

"long" Long integer (size is platform dependent).

"ushort"
"unsigned short"
: Unsigned short integer (size is platform dependent).

"uint"
"unsigned int"
: Unsigned integer (size is platform dependent).

"ulong"
"unsigned long"
: Unsigned long integer (size is platform dependent).

"float" Single precision floating point number (size is platform dependent).

The default precision is "uchar".

The *precision* argument may also specify an optional repeat count. For example, '32*single' causes fread to read a block of 32 single precision floating point numbers. Reading in blocks is useful in combination with the *skip* argument.

The *precision* argument may also specify a type conversion. For example, '`int16=>int32`' causes `fread` to read 16-bit integer values and return an array of 32-bit integer values. By default, `fread` returns a double precision array. The special form '`*TYPE`' is shorthand for '`TYPE=>TYPE`'.

The conversion and repeat counts may be combined. For example, the specification '`32*single=>single`' causes `fread` to read blocks of single precision floating point values and return an array of single precision values instead of the default array of double precision values.

The optional argument *skip* specifies the number of bytes to skip after each element (or block of elements) is read. If it is not specified, a value of 0 is assumed. If the final block read is not complete, the final skip is omitted. For example,

```
fread (f, 10, "3*single=>single", 8)
```

will omit the final 8-byte skip because the last read will not be a complete block of 3 values.

The optional argument *arch* is a string specifying the data format for the file. Valid values are

`"native"` The format of the current machine.

`"ieee-be"`
IEEE big endian.

`"ieee-le"`
IEEE little endian.

`"vaxd"` VAX D floating format.

`"vaxg"` VAX G floating format.

`"cray"` Cray floating format.

Conversions are currently only supported for `"ieee-be"` and `"ieee-le"` formats.

The data read from the file is returned in *val*, and the number of values read is returned in `count`

See also: [fwrite], page 201, [fopen], page 188, [fclose], page 189.

count = **fwrite** (*fid*, *data*, *precision*, *skip*, *arch*) [Built-in Function]

Write data in binary form of type *precision* to the specified file ID *fid*, returning the number of values successfully written to the file.

The argument *data* is a matrix of values that are to be written to the file. The values are extracted in column-major order.

The remaining arguments *precision*, *skip*, and *arch* are optional, and are interpreted as described for `fread`.

The behavior of `fwrite` is undefined if the values in *data* are too large to fit in the specified precision.

See also: [fread], page 199, [fopen], page 188, [fclose], page 189.

14.2.17 Temporary Files

Sometimes one needs to write data to a file that is only temporary. This is most commonly used when an external program launched from within Octave needs to access data. When Octave exits all temporary files will be deleted, so this step need not be executed manually.

[*fid*, *name*, *msg*] = `mkstemp` (*template*, *delete*) [Built-in Function]

Return the file ID corresponding to a new temporary file with a unique name created from *template*. The last six characters of *template* must be `XXXXXX` and these are replaced with a string that makes the filename unique. The file is then created with mode read/write and permissions that are system dependent (on GNU/Linux systems, the permissions will be 0600 for versions of glibc 2.0.7 and later). The file is opened with the `O_EXCL` flag.

If the optional argument *delete* is supplied and is true, the file will be deleted automatically when Octave exits, or when the function `purge_tmp_files` is called.

If successful, *fid* is a valid file ID, *name* is the name of the file, and *msg* is an empty string. Otherwise, *fid* is -1, *name* is empty, and *msg* contains a system-dependent error message.

See also: [tmpfile], page 202, [tmpnam], page 202, [P_tmpdir], page 530.

[*fid*, *msg*] = `tmpfile` () [Built-in Function]

Return the file ID corresponding to a new temporary file with a unique name. The file is opened in binary read/write (`"w+b"`) mode. The file will be deleted automatically when it is closed or when Octave exits.

If successful, *fid* is a valid file ID and *msg* is an empty string. Otherwise, *fid* is -1 and *msg* contains a system-dependent error message.

See also: [tmpnam], page 202, [mkstemp], page 202, [P_tmpdir], page 530.

`tmpnam` (*dir*, *prefix*) [Built-in Function]

Return a unique temporary file name as a string.

If *prefix* is omitted, a value of `"oct-"` is used. If *dir* is also omitted, the default directory for temporary files is used. If *dir* is provided, it must exist, otherwise the default directory for temporary files is used. Since the named file is not opened, by `tmpnam`, it is possible (though relatively unlikely) that it will not be available by the time your program attempts to open it.

See also: [tmpfile], page 202, [mkstemp], page 202, [P_tmpdir], page 530.

14.2.18 End of File and Errors

Once a file has been opened its status can be acquired. As an example the `feof` functions determines if the end of the file has been reached. This can be very useful when reading small parts of a file at a time. The following example shows how to read one line at a time from a file until the end has been reached.

```
filename = "myfile.txt";
fid = fopen (filename, "r");
while (! feof (fid) )
  text_line = fgetl (fid);
endwhile
fclose (fid);
```

Note that in some situations it is more efficient to read the entire contents of a file and then process it, than it is to read it line by line. This has the potential advantage of removing the loop in the above code.

`feof` (*fid*) [Built-in Function]

Return 1 if an end-of-file condition has been encountered for a given file and 0 otherwise. Note that it will only return 1 if the end of the file has already been encountered, not if the next read operation will result in an end-of-file condition.

See also: [fread], page 199, [fopen], page 188, [fclose], page 189.

`ferror` (*fid*) [Built-in Function]

Return 1 if an error condition has been encountered for a given file and 0 otherwise. Note that it will only return 1 if an error has already been encountered, not if the next operation will result in an error condition.

`fclear` (*fid*) [Built-in Function]

Clear the stream state for the specified file.

`freport` () [Built-in Function]

Print a list of which files have been opened, and whether they are open for reading, writing, or both. For example,

```
freport ()

     ⊣  number  mode  name
     ⊣
     ⊣       0     r  stdin
     ⊣       1     w  stdout
     ⊣       2     w  stderr
     ⊣       3     r  myfile
```

14.2.19 File Positioning

Three functions are available for setting and determining the position of the file pointer for a given file.

`ftell` (*fid*) [Built-in Function]

Return the position of the file pointer as the number of characters from the beginning of the file *fid*.

See also: [fseek], page 203, [fopen], page 188, [fclose], page 189.

`fseek` (*fid*, *offset*, *origin*) [Built-in Function]

Set the file pointer to any location within the file *fid*.

The pointer is positioned *offset* characters from the *origin*, which may be one of the predefined variables `SEEK_CUR` (current position), `SEEK_SET` (beginning), or `SEEK_END` (end of file) or strings `"cof"`, `"bof"` or `"eof"`. If *origin* is omitted, `SEEK_SET` is assumed. The offset must be zero, or a value returned by `ftell` (in which case *origin* must be `SEEK_SET`).

Return 0 on success and -1 on error.

See also: [ftell], page 203, [fopen], page 188, [fclose], page 189.

`SEEK_SET ()` [Built-in Function]
`SEEK_CUR ()` [Built-in Function]
`SEEK_END ()` [Built-in Function]

Return the value required to request that `fseek` perform one of the following actions:

`SEEK_SET` Position file relative to the beginning.

`SEEK_CUR` Position file relative to the current position.

`SEEK_END` Position file relative to the end.

`frewind (`*`fid`*`)` [Built-in Function]

Move the file pointer to the beginning of the file *fid*, returning 0 for success, and -1 if an error was encountered. It is equivalent to `fseek (fid, 0, SEEK_SET)`.

The following example stores the current file position in the variable `marker`, moves the pointer to the beginning of the file, reads four characters, and then returns to the original position.

```
marker = ftell (myfile);
frewind (myfile);
fourch = fgets (myfile, 4);
fseek (myfile, marker, SEEK_SET);
```

15 Plotting

15.1 Plotting Basics

Octave makes it easy to create many different types of two- and three-dimensional plots using a few high-level functions.

If you need finer control over graphics, see Section 15.2 [Advanced Plotting], page 243.

15.1.1 Two-Dimensional Plots

The `plot` function allows you to create simple x-y plots with linear axes. For example,

```
x = -10:0.1:10;
plot (x, sin (x));
```

displays a sine wave shown in Figure 15.1. On most systems, this command will open a separate plot window to display the graph.

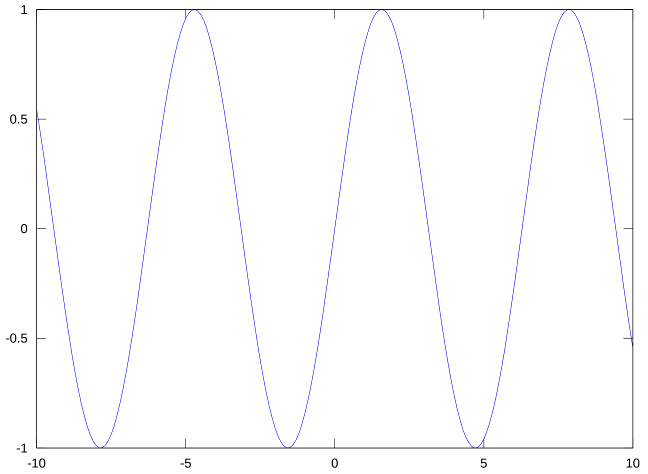

Figure 15.1: Simple Two-Dimensional Plot.

plot (*y*) [Function File]
plot (*x*, *y*) [Function File]
plot (*x*, *y*, *property*, *value*, ...) [Function File]
plot (*x*, *y*, *fmt*) [Function File]
plot (*h*, ...) [Function File]

Produces two-dimensional plots. Many different combinations of arguments are possible. The simplest form is

```
plot (y)
```

where the argument is taken as the set of *y* coordinates and the *x* coordinates are taken to be the indices of the elements, starting with 1.

To save a plot, in one of several image formats such as PostScript or PNG, use the `print` command.

If more than one argument is given, they are interpreted as

```
plot (y, property, value, ...)
```

or

```
plot (x, y, property, value, ...)
```

or

```
plot (x, y, fmt, ...)
```

and so on. Any number of argument sets may appear. The *x* and *y* values are interpreted as follows:

- If a single data argument is supplied, it is taken as the set of *y* coordinates and the *x* coordinates are taken to be the indices of the elements, starting with 1.
- If the *x* is a vector and *y* is a matrix, then the columns (or rows) of *y* are plotted versus *x*. (using whichever combination matches, with columns tried first.)
- If the *x* is a matrix and *y* is a vector, *y* is plotted versus the columns (or rows) of *x*. (using whichever combination matches, with columns tried first.)
- If both arguments are vectors, the elements of *y* are plotted versus the elements of *x*.
- If both arguments are matrices, the columns of *y* are plotted versus the columns of *x*. In this case, both matrices must have the same number of rows and columns and no attempt is made to transpose the arguments to make the number of rows match.

  If both arguments are scalars, a single point is plotted.

Multiple property-value pairs may be specified, but they must appear in pairs. These arguments are applied to the lines drawn by `plot`.

If the *fmt* argument is supplied, it is interpreted as follows. If *fmt* is missing, the default gnuplot line style is assumed.

'`-`' Set lines plot style (default).

'`.`' Set dots plot style.

'*`n`*' Interpreted as the plot color if *n* is an integer in the range 1 to 6.

'*`nm`*' If *nm* is a two digit integer and *m* is an integer in the range 1 to 6, *m* is interpreted as the point style. This is only valid in combination with the `@` or `-@` specifiers.

'`c`' If *c* is one of `"k"` (black), `"r"` (red), `"g"` (green), `"b"` (blue), `"m"` (magenta), `"c"` (cyan), or `"w"` (white), it is interpreted as the line plot color.

'`";title;"`'
Here `"title"` is the label for the key.

'`+`'
'`*`'
'`o`'
'`x`'
'`^`' Used in combination with the points or linespoints styles, set the point style.

The *fmt* argument may also be used to assign key titles. To do so, include the desired title between semi-colons after the formatting sequence described above, e.g., "+3;Key Title;" Note that the last semi-colon is required and will generate an error if it is left out.

Here are some plot examples:

```
plot (x, y, "@12", x, y2, x, y3, "4", x, y4, "+")
```

This command will plot `y` with points of type 2 (displayed as '+') and color 1 (red), `y2` with lines, `y3` with lines of color 4 (magenta) and `y4` with points displayed as '+'.

```
plot (b, "*", "markersize", 3)
```

This command will plot the data in the variable `b`, with points displayed as '*' with a marker size of 3.

```
t = 0:0.1:6.3;
plot (t, cos(t), "-;cos(t);", t, sin(t), "+3;sin(t);");
```

This will plot the cosine and sine functions and label them accordingly in the key.

If the first argument is an axis handle, then plot into these axes, rather than the current axis handle returned by `gca`.

See also: [semilogx], page 208, [semilogy], page 208, [loglog], page 208, [polar], page 218, [mesh], page 227, [contour], page 213, [bar], page 208, [stairs], page 210, [errorbar], page 216, [xlabel], page 236, [ylabel], page 236, [title], page 236, [print], page 239.

The `plotyy` function may be used to create a plot with two independent y axes.

`plotyy` (*x1*, *y1*, *x2*, *y2*) [Function File]
`plotyy` (. . ., *fun*) [Function File]
`plotyy` (. . ., *fun1*, *fun2*) [Function File]
`plotyy` (*h*, . . .) [Function File]
[`ax`, *h1*, *h2*] = `plotyy` (. . .) [Function File]

Plots two sets of data with independent y-axes. The arguments *x1* and *y1* define the arguments for the first plot and *x1* and *y2* for the second.

By default the arguments are evaluated with `feval (@plot, x, y)`. However the type of plot can be modified with the *fun* argument, in which case the plots are generated by `feval (fun, x, y)`. *fun* can be a function handle, an inline function or a string of a function name.

The function to use for each of the plots can be independently defined with *fun1* and *fun2*.

If given, *h* defines the principal axis in which to plot the *x1* and *y1* data. The return value *ax* is a two element vector with the axis handles of the two plots. *h1* and *h2* are handles to the objects generated by the plot commands.

```
x = 0:0.1:2*pi;
y1 = sin (x);
y2 = exp (x - 1);
ax = plotyy (x, y1, x - 1, y2, @plot, @semilogy);
xlabel ("X");
ylabel (ax(1), "Axis 1");
ylabel (ax(2), "Axis 2");
```

The functions `semilogx`, `semilogy`, and `loglog` are similar to the `plot` function, but produce plots in which one or both of the axes use log scales.

`semilogx` (*args*) [Function File]

Produce a two-dimensional plot using a log scale for the *x* axis. See the description of `plot` for a description of the arguments that `semilogx` will accept.

See also: [plot], page 205, [semilogy], page 208, [loglog], page 208.

`semilogy` (*args*) [Function File]

Produce a two-dimensional plot using a log scale for the *y* axis. See the description of `plot` for a description of the arguments that `semilogy` will accept.

See also: [plot], page 205, [semilogx], page 208, [loglog], page 208.

`loglog` (*args*) [Function File]

Produce a two-dimensional plot using log scales for both axes. See the description of `plot` for a description of the arguments that `loglog` will accept.

See also: [plot], page 205, [semilogx], page 208, [semilogy], page 208.

The functions `bar`, `barh`, `stairs`, and `stem` are useful for displaying discrete data. For example,

```
hist (randn (10000, 1), 30);
```

produces the histogram of 10,000 normally distributed random numbers shown in Figure 15.2.

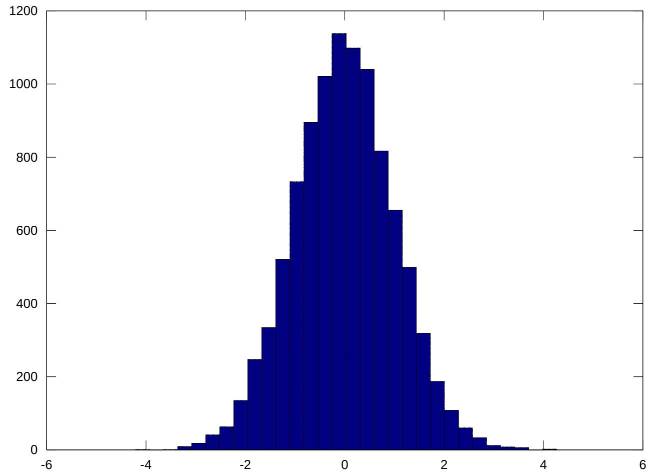

Figure 15.2: Histogram.

`bar` (*x*, *y*) [Function File]
`bar` (*y*) [Function File]
`bar` (*x*, *y*, *w*) [Function File]
`bar` (*x*, *y*, *w*, *style*) [Function File]
h = `bar` (. . ., *prop*, *val*) [Function File]

`bar` (*h*, ...) [Function File]

Produce a bar graph from two vectors of x-y data.

If only one argument is given, it is taken as a vector of y-values and the x coordinates are taken to be the indices of the elements.

The default width of 0.8 for the bars can be changed using *w*.

If *y* is a matrix, then each column of *y* is taken to be a separate bar graph plotted on the same graph. By default the columns are plotted side-by-side. This behavior can be changed by the *style* argument, which can take the values `"grouped"` (the default), or `"stacked"`.

The optional return value *h* provides a handle to the "bar series" object with one handle per column of the variable *y*. This series allows common elements of the group of bar series objects to be changed in a single bar series and the same properties are changed in the other "bar series". For example

```
h = bar (rand (5, 10));
set (h(1), "basevalue", 0.5);
```

changes the position on the base of all of the bar series.

The optional input handle *h* allows an axis handle to be passed. Properties of the patch graphics object can be changed using *prop*, *val* pairs.

See also: [barh], page 209, [plot], page 205.

`barh` (*x*, *y*) [Function File]
`barh` (*y*) [Function File]
`barh` (*x*, *y*, *w*) [Function File]
`barh` (*x*, *y*, *w*, *style*) [Function File]
h = `barh` (..., *prop*, *val*) [Function File]
`barh` (*h*, ...) [Function File]

Produce a horizontal bar graph from two vectors of x-y data.

If only one argument is given, it is taken as a vector of y-values and the x coordinates are taken to be the indices of the elements.

The default width of 0.8 for the bars can be changed using *w*.

If *y* is a matrix, then each column of *y* is taken to be a separate bar graph plotted on the same graph. By default the columns are plotted side-by-side. This behavior can be changed by the *style* argument, which can take the values `"grouped"` (the default), or `"stacked"`.

The optional return value *h* provides a handle to the bar series object. See `bar` for a description of the use of the bar series.

The optional input handle *h* allows an axis handle to be passed. Properties of the patch graphics object can be changed using *prop*, *val* pairs.

See also: [bar], page 208, [plot], page 205.

`hist` (*y*, *x*, *norm*) [Function File]

Produce histogram counts or plots.

With one vector input argument, plot a histogram of the values with 10 bins. The range of the histogram bins is determined by the range of the data. With one matrix input argument, plot a histogram where each bin contains a bar per input column.

Given a second scalar argument, use that as the number of bins.

Given a second vector argument, use that as the centers of the bins, with the width of the bins determined from the adjacent values in the vector.

If third argument is provided, the histogram is normalized such that the sum of the bars is equal to *norm*.

Extreme values are lumped in the first and last bins.

With two output arguments, produce the values *nn* and *xx* such that `bar (xx, nn)` will plot the histogram.

See also: [bar], page 208.

`stairs` (*x*, *y*) [Function File]
`stairs` (..., *style*) [Function File]
`stairs` (..., *prop*, *val*) [Function File]
`stairs` (*h*, ...) [Function File]
h = `stairs` (...) [Function File]

Produce a stairstep plot. The arguments may be vectors or matrices.

If only one argument is given, it is taken as a vector of y-values and the x coordinates are taken to be the indices of the elements.

If two output arguments are specified, the data are generated but not plotted. For example,

```
stairs (x, y);
```

and

```
[xs, ys] = stairs (x, y);
plot (xs, ys);
```

are equivalent.

See also: [plot], page 205, [semilogx], page 208, [semilogy], page 208, [loglog], page 208, [polar], page 218, [mesh], page 227, [contour], page 213, [bar], page 208, [xlabel], page 236, [ylabel], page 236, [title], page 236.

h = `stem` (*x*, *y*, *linespec*) [Function File]
h = `stem` (..., "*filled*") [Function File]

Plot a stem graph from two vectors of x-y data. If only one argument is given, it is taken as the y-values and the x coordinates are taken from the indices of the elements.

If *y* is a matrix, then each column of the matrix is plotted as a separate stem graph. In this case *x* can either be a vector, the same length as the number of rows in *y*, or it can be a matrix of the same size as *y*.

The default color is `"r"` (red). The default line style is `"-"` and the default marker is `"o"`. The line style can be altered by the `linespec` argument in the same manner as the `plot` command. For example

```
x = 1:10;
y = ones (1, length (x))*2.*x;
stem (x, y, "b");
```

plots 10 stems with heights from 2 to 20 in blue;

The return value of `stem` is a vector if "stem series" graphics handles, with one handle per column of the variable *y*. This handle regroups the elements of the stem graph together as the children of the "stem series" handle, allowing them to be altered together. For example

```
x = [0 : 10].';
y = [sin(x), cos(x)]
h = stem (x, y);
set (h(2), "color", "g");
set (h(1), "basevalue", -1)
```

changes the color of the second "stem series" and moves the base line of the first.

See also: [bar], page 208, [barh], page 209, [plot], page 205.

h = `stem3` (*x*, *y*, *z*, *linespec*) [Function File]

Plot a three-dimensional stem graph and return the handles of the line and marker objects used to draw the stems as "stem series" object. The default color is `"r"` (red). The default line style is `"-"` and the default marker is `"o"`.

For example,

```
theta = 0:0.2:6;
stem3 (cos (theta), sin (theta), theta)
```

plots 31 stems with heights from 0 to 6 lying on a circle. Color definitions with rgb-triples are not valid!

See also: [bar], page 208, [barh], page 209, [stem], page 210, [plot], page 205.

`scatter` (*x*, *y*, *s*, *c*) [Function File]
`scatter` (. . ., *'filled'*) [Function File]
`scatter` (. . ., *style*) [Function File]
`scatter` (. . ., *prop*, *val*) [Function File]
`scatter` (*h*, . . .) [Function File]
h = `scatter` (. . .) [Function File]

Plot a scatter plot of the data. A marker is plotted at each point defined by the points in the vectors *x* and *y*. The size of the markers used is determined by the *s*, which can be a scalar, a vector of the same length of *x* and *y*. If *s* is not given or is an empty matrix, then the default value of 8 points is used.

The color of the markers is determined by *c*, which can be a string defining a fixed color, a 3 element vector giving the red, green and blue components of the color, a vector of the same length as *x* that gives a scaled index into the current colormap, or a *n*-by-3 matrix defining the colors of each of the markers individually.

The marker to use can be changed with the *style* argument, that is a string defining a marker in the same manner as the `plot` command. If the argument 'filled' is given then the markers as filled. All additional arguments are passed to the underlying patch command.

The optional return value *h* provides a handle to the patch object

```
x = randn (100, 1);
y = randn (100, 1);
scatter (x, y, [], sqrt(x.^2 + y.^2));
```

See also: [plot], page 205, [patch], page 246, [scatter3], page 212.

`scatter3 (x, y, z, s, c)` [Function File]
`scatter3 (..., 'filled')` [Function File]
`scatter3 (..., style)` [Function File]
`scatter3 (..., prop, val)` [Function File]
`scatter3 (h, ...)` [Function File]
`h = scatter3 (...)` [Function File]

Plot a scatter plot of the data in 3D. A marker is plotted at each point defined by the points in the vectors *x*, *y* and *z*. The size of the markers used is determined by *s*, which can be a scalar or a vector of the same length of *x*, *y* and *z*. If *s* is not given or is an empty matrix, then the default value of 8 points is used.

The color of the markers is determined by *c*, which can be a string defining a fixed color, a 3 element vector giving the red, green and blue components of the color, a vector of the same length as *x* that gives a scaled index into the current colormap, or a *n*-by-3 matrix defining the colors of each of the markers individually.

The marker to use can be changed with the *style* argument, that is a string defining a marker in the same manner as the `plot` command. If the argument 'filled' is given then the markers as filled. All additional arguments are passed to the underlying patch command.

The optional return value *h* provides a handle to the patch object

```
[x, y, z] = peaks (20);
scatter3 (x(:), y(:), z(:), [], z(:));
```

See also: [plot], page 205, [patch], page 246, [scatter], page 211.

`plotmatrix (x, y)` [Function File]
`plotmatrix (x)` [Function File]
`plotmatrix (..., style)` [Function File]
`plotmatrix (h, ...)` [Function File]
`[h, ax, bigax, p, pax] = plotmatrix (...)` [Function File]

Scatter plot of the columns of one matrix against another. Given the arguments *x* and *y*, that have a matching number of rows, `plotmatrix` plots a set of axes corresponding to

```
plot (x (:, i), y (:, j)
```

Given a single argument *x*, then this is equivalent to

```
plotmatrix (x, x)
```

except that the diagonal of the set of axes will be replaced with the histogram `hist (x (:, i))`.

The marker to use can be changed with the *style* argument, that is a string defining a marker in the same manner as the `plot` command. If a leading axes handle *h* is passed to `plotmatrix`, then this axis will be used for the plot.

The optional return value *h* provides handles to the individual graphics objects in the scatter plots, whereas *ax* returns the handles to the scatter plot axis objects. *bigax* is a hidden axis object that surrounds the other axes, such that the commands `xlabel`, `title`, etc., will be associated with this hidden axis. Finally *p* returns the graphics objects associated with the histogram and *pax* the corresponding axes objects.

```
plotmatrix (randn (100, 3), 'g+')
```

`pareto (x)` [Function File]
`pareto (x, y)` [Function File]
`pareto (h, ...)` [Function File]
`h = pareto (...)` [Function File]

Draw a Pareto chart, also called ABC chart. A Pareto chart is a bar graph used to arrange information in such a way that priorities for process improvement can be established. It organizes and displays information to show the relative importance of data. The chart is similar to the histogram or bar chart, except that the bars are arranged in decreasing order from left to right along the abscissa.

The fundamental idea (Pareto principle) behind the use of Pareto diagrams is that the majority of an effect is due to a small subset of the causes, so for quality improvement the first few (as presented on the diagram) contributing causes to a problem usually account for the majority of the result. Thus, targeting these "major causes" for elimination results in the most cost-effective improvement scheme.

The data are passed as *x* and the abscissa as *y*. If *y* is absent, then the abscissa are assumed to be `1 : length (x)`. *y* can be a string array, a cell array of strings or a numerical vector.

An example of the use of `pareto` is

```
Cheese = {"Cheddar", "Swiss", "Camembert", ...
          "Munster", "Stilton", "Blue"};
Sold = [105, 30, 70, 10, 15, 20];
pareto(Sold, Cheese);
```

`rose (th, r)` [Function File]
`rose (h, ...)` [Function File]
`h = rose (...)` [Function File]
`[r, th] = rose (...)` [Function File]

Plot an angular histogram. With one vector argument *th*, plots the histogram with 20 angular bins. If *th* is a matrix, then each column of *th* produces a separate histogram.

If *r* is given and is a scalar, then the histogram is produced with *r* bins. If *r* is a vector, then the center of each bin are defined by the values of *r*.

The optional return value *h* provides a list of handles to the the parts of the vector field (body, arrow and marker).

If two output arguments are requested, then rather than plotting the histogram, the polar vectors necessary to plot the histogram are returned.

```
[r, t] = rose ([2*randn(1e5,1), pi + 2 * randn(1e5,1)]);
polar (r, t);
```

See also: [plot], page 205, [compass], page 220, [polar], page 218, [hist], page 209.

The `contour`, `contourf` and `contourc` functions produce two-dimensional contour plots from three-dimensional data.

`contour (z)` [Function File]
`contour (z, vn)` [Function File]

`contour (x, y, z)` [Function File]
`contour (x, y, z, vn)` [Function File]
`contour (..., style)` [Function File]
`contour (h, ...)` [Function File]
`[c, h] = contour (...)` [Function File]

Plot level curves (contour lines) of the matrix *z*, using the contour matrix *c* computed by `contourc` from the same arguments; see the latter for their interpretation. The set of contour levels, *c*, is only returned if requested. For example:

```
x = 0:2;
y = x;
z = x' * y;
contour (x, y, z, 2:3)
```

The style to use for the plot can be defined with a line style *style* in a similar manner to the line styles used with the `plot` command. Any markers defined by *style* are ignored.

The optional input and output argument *h* allows an axis handle to be passed to `contour` and the handles to the contour objects to be returned.

See also: [contourc], page 214, [patch], page 246, [plot], page 205.

`[c, h] = contourf (x, y, z, lvl)` [Function File]
`[c, h] = contourf (x, y, z, n)` [Function File]
`[c, h] = contourf (x, y, z)` [Function File]
`[c, h] = contourf (z, n)` [Function File]
`[c, h] = contourf (z, lvl)` [Function File]
`[c, h] = contourf (z)` [Function File]
`[c, h] = contourf (ax, ...)` [Function File]
`[c, h] = contourf (..., "property", val)` [Function File]

Compute and plot filled contours of the matrix *z*. Parameters *x*, *y* and *n* or *lvl* are optional.

The return value *c* is a 2xn matrix containing the contour lines as described in the help to the contourc function.

The return value *h* is handle-vector to the patch objects creating the filled contours.

If *x* and *y* are omitted they are taken as the row/column index of *z*. *n* is a scalar denoting the number of lines to compute. Alternatively *lvl* is a vector containing the contour levels. If only one value (e.g., lvl0) is wanted, set *lvl* to [lvl0, lvl0]. If both *n* or *lvl* are omitted a default value of 10 contour level is assumed.

If provided, the filled contours are added to the axes object *ax* instead of the current axis.

The following example plots filled contours of the `peaks` function.

```
[x, y, z] = peaks (50);
contourf (x, y, z, -7:9)
```

See also: [contour], page 213, [contourc], page 214, [patch], page 246.

`[c, lev] = contourc (x, y, z, vn)` [Function File]

Compute isolines (contour lines) of the matrix *z*. Parameters *x*, *y* and *vn* are optional.

The return value *lev* is a vector of the contour levels. The return value *c* is a 2 by *n* matrix containing the contour lines in the following format

```
c = [lev1, x1, x2, ..., levn, x1, x2, ...
     len1, y1, y2, ..., lenn, y1, y2, ...]
```

in which contour line *n* has a level (height) of *levn* and length of *lenn*.

If *x* and *y* are omitted they are taken as the row/column index of *z*. *vn* is either a scalar denoting the number of lines to compute or a vector containing the values of the lines. If only one value is wanted, set `vn = [val, val]`; If *vn* is omitted it defaults to 10.

For example,

```
x = 0:2;
y = x;
z = x' * y;
contourc (x, y, z, 2:3)
     ⇒   2.0000   2.0000   1.0000   3.0000   1.5000   2.0000
     2.0000   1.0000   2.0000   2.0000   2.0000   1.5000
```

See also: [contour], page 213.

`contour3` (*z*) [Function File]
`contour3` (*z*, *vn*) [Function File]
`contour3` (*x*, *y*, *z*) [Function File]
`contour3` (*x*, *y*, *z*, *vn*) [Function File]
`contour3` (. . ., *style*) [Function File]
`contour3` (*h*, . . .) [Function File]
`[c, h] = contour3` (. . .) [Function File]

Plot level curves (contour lines) of the matrix *z*, using the contour matrix *c* computed by `contourc` from the same arguments; see the latter for their interpretation. The contours are plotted at the Z level corresponding to their contour. The set of contour levels, *c*, is only returned if requested. For example:

```
contour3 (peaks (19));
hold on
surface (peaks (19), "facecolor", "none", "EdgeColor", "black")
colormap hot
```

The style to use for the plot can be defined with a line style *style* in a similar manner to the line styles used with the `plot` command. Any markers defined by *style* are ignored.

The optional input and output argument *h* allows an axis handle to be passed to `contour` and the handles to the contour objects to be returned.

See also: [contourc], page 214, [patch], page 246, [plot], page 205.

The `errorbar`, `semilogxerr`, `semilogyerr`, and `loglogerr` functions produce plots with error bar markers. For example,

```
x = 0:0.1:10;
y = sin (x);
yp =  0.1 .* randn (size (x));
ym = -0.1 .* randn (size (x));
errorbar (x, sin (x), ym, yp);
```

produces the figure shown in Figure 15.3.

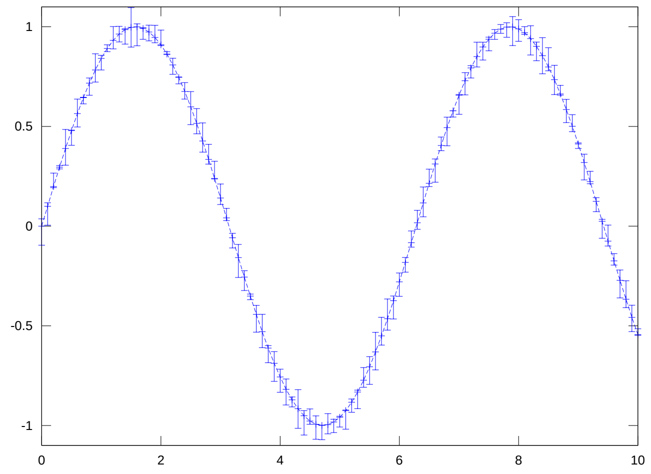

Figure 15.3: Errorbar plot.

errorbar (*args*) [Function File]

This function produces two-dimensional plots with errorbars. Many different combinations of arguments are possible. The simplest form is

```
errorbar (y, ey)
```

where the first argument is taken as the set of *y* coordinates and the second argument *ey* is taken as the errors of the *y* values. *x* coordinates are taken to be the indices of the elements, starting with 1.

If more than two arguments are given, they are interpreted as

```
errorbar (x, y, ..., fmt, ...)
```

where after *x* and *y* there can be up to four error parameters such as *ey*, *ex*, *ly*, *uy*, etc., depending on the plot type. Any number of argument sets may appear, as long as they are separated with a format string *fmt*.

If *y* is a matrix, *x* and error parameters must also be matrices having same dimensions. The columns of *y* are plotted versus the corresponding columns of *x* and errorbars are drawn from the corresponding columns of error parameters.

If *fmt* is missing, yerrorbars (`"~"`) plot style is assumed.

If the *fmt* argument is supplied, it is interpreted as in normal plots. In addition the following plot styles are supported by errorbar:

‘~’ Set yerrorbars plot style (default).

'>' Set xerrorbars plot style.

'~>' Set xyerrorbars plot style.

'#' Set boxes plot style.

'#~' Set boxerrorbars plot style.

'#~>' Set boxxyerrorbars plot style.

Examples:

```
errorbar (x, y, ex, ">")
```

produces an xerrorbar plot of *y* versus *x* with *x* errorbars drawn from *x*-*ex* to *x*+*ex*.

```
errorbar (x, y1, ey, "~",
          x, y2, ly, uy)
```

produces yerrorbar plots with *y1* and *y2* versus *x*. Errorbars for *y1* are drawn from *y1*-*ey* to *y1*+*ey*, errorbars for *y2* from *y2*-*ly* to *y2*+*uy*.

```
errorbar (x, y, lx, ux,
          ly, uy, "~>")
```

produces an xyerrorbar plot of *y* versus *x* in which *x* errorbars are drawn from *x*-*lx* to *x*+*ux* and *y* errorbars from *y*-*ly* to *y*+*uy*.

See also: [semilogxerr], page 217, [semilogyerr], page 217, [loglogerr], page 217.

`semilogxerr` (*args*) [Function File]

Produce two-dimensional plots on a semilogarithm axis with errorbars. Many different combinations of arguments are possible. The most used form is

```
semilogxerr (x, y, ey, fmt)
```

which produces a semi-logarithm plot of *y* versus *x* with errors in the *y*-scale defined by *ey* and the plot format defined by *fmt*. See errorbar for available formats and additional information.

See also: [errorbar], page 216, [loglogerr], page 217, [semilogyerr], page 217.

`semilogyerr` (*args*) [Function File]

Produce two-dimensional plots on a semilogarithm axis with errorbars. Many different combinations of arguments are possible. The most used form is

```
semilogyerr (x, y, ey, fmt)
```

which produces a semi-logarithm plot of *y* versus *x* with errors in the *y*-scale defined by *ey* and the plot format defined by *fmt*. See errorbar for available formats and additional information.

See also: [errorbar], page 216, [loglogerr], page 217, [semilogxerr], page 217.

`loglogerr` (*args*) [Function File]

Produce two-dimensional plots on double logarithm axis with errorbars. Many different combinations of arguments are possible. The most used form is

```
loglogerr (x, y, ey, fmt)
```

which produces a double logarithm plot of *y* versus *x* with errors in the *y*-scale defined by *ey* and the plot format defined by *fmt*. See errorbar for available formats and additional information.

See also: [errorbar], page 216, [semilogxerr], page 217, [semilogyerr], page 217.

Finally, the `polar` function allows you to easily plot data in polar coordinates. However, the display coordinates remain rectangular and linear. For example,

```
polar (0:0.1:10*pi, 0:0.1:10*pi);
```

produces the spiral plot shown in Figure 15.4.

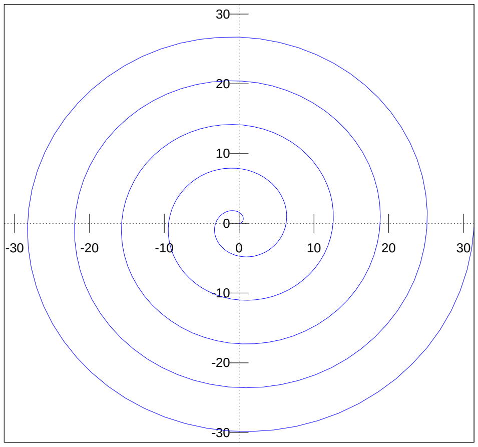

Figure 15.4: Polar plot.

`polar (`*`theta`*`, `*`rho`*`, `*`fmt`*`)` [Function File]

Make a two-dimensional plot given the polar coordinates *theta* and *rho*.

The optional third argument specifies the line type.

See also: [plot], page 205.

`pie (`*y*`)` [Function File]
`pie (`*y*`, `*`explode`*`)` [Function File]
`pie (`. . . `, `*`labels`*`)` [Function File]
`pie (`*h*`, `. . .`);` [Function File]
h `= pie (`. . .`);` [Function File]

Produce a pie chart.

Called with a single vector argument, produces a pie chart of the elements in *x*, with the size of the slice determined by percentage size of the values of *x*.

The variable *explode* is a vector of the same length as *x* that if non zero 'explodes' the slice from the pie chart.

If given *labels* is a cell array of strings of the same length as *x*, giving the labels of each of the slices of the pie chart.

The optional return value *h* provides a handle to the patch object.

See also: [bar], page 208, [stem], page 210.

`quiver (`*u*`, `*v*`)` [Function File]
`quiver (`*x*`, `*y*`, `*u*`, `*v*`)` [Function File]
`quiver (`. . . `, `*s*`)` [Function File]

`quiver (..., style)` [Function File]
`quiver (..., 'filled')` [Function File]
`quiver (h, ...)` [Function File]
`h = quiver (...)` [Function File]

Plot the `(u, v)` components of a vector field in an `(x, y)` meshgrid. If the grid is uniform, you can specify *x* and *y* as vectors.

If *x* and *y* are undefined they are assumed to be `(1:m, 1:n)` where `[m, n] = size(u)`.

The variable *s* is a scalar defining a scaling factor to use for the arrows of the field relative to the mesh spacing. A value of 0 disables all scaling. The default value is 1.

The style to use for the plot can be defined with a line style *style* in a similar manner to the line styles used with the `plot` command. If a marker is specified then markers at the grid points of the vectors are printed rather than arrows. If the argument 'filled' is given then the markers as filled.

The optional return value *h* provides a quiver group that regroups the components of the quiver plot (body, arrow and marker), and allows them to be changed together

```
[x, y] = meshgrid (1:2:20);
h = quiver (x, y, sin (2*pi*x/10), sin (2*pi*y/10));
set (h, "maxheadsize", 0.33);
```

See also: [plot], page 205.

`quiver3 (u, v, w)` [Function File]
`quiver3 (x, y, z, u, v, w)` [Function File]
`quiver3 (..., s)` [Function File]
`quiver3 (..., style)` [Function File]
`quiver3 (..., 'filled')` [Function File]
`quiver3 (h, ...)` [Function File]
`h = quiver3 (...)` [Function File]

Plot the `(u, v, w)` components of a vector field in an `(x, y), z` meshgrid. If the grid is uniform, you can specify *x*, *y* *z* as vectors.

If *x*, *y* and *z* are undefined they are assumed to be `(1:m, 1:n, 1:p)` where `[m, n] = size(u)` and `p = max (size (w))`.

The variable *s* is a scalar defining a scaling factor to use for the arrows of the field relative to the mesh spacing. A value of 0 disables all scaling. The default value is 1.

The style to use for the plot can be defined with a line style *style* in a similar manner to the line styles used with the `plot` command. If a marker is specified then markers at the grid points of the vectors are printed rather than arrows. If the argument 'filled' is given then the markers as filled.

The optional return value *h* provides a quiver group that regroups the components of the quiver plot (body, arrow and marker), and allows them to be changed together

```
[x, y, z] = peaks (25);
surf (x, y, z);
hold on;
[u, v, w] = surfnorm (x, y, z / 10);
h = quiver3 (x, y, z, u, v, w);
set (h, "maxheadsize", 0.33);
```

See also: [plot], page 205.

`compass (u, v)` [Function File]
`compass (z)` [Function File]
`compass (..., style)` [Function File]
`compass (h, ...)` [Function File]
`h = compass (...)` [Function File]

Plot the `(u, v)` components of a vector field emanating from the origin of a polar plot. If a single complex argument *z* is given, then `u = real (z)` and `v = imag (z)`.

The style to use for the plot can be defined with a line style *style* in a similar manner to the line styles used with the `plot` command.

The optional return value *h* provides a list of handles to the the parts of the vector field (body, arrow and marker).

```
a = toeplitz([1;randn(9,1)],[1,randn(1,9)]);
compass (eig (a))
```

See also: [plot], page 205, [polar], page 218, [quiver], page 218, [feather], page 220.

`feather (u, v)` [Function File]
`feather (z)` [Function File]
`feather (..., style)` [Function File]
`feather (h, ...)` [Function File]
`h = feather (...)` [Function File]

Plot the `(u, v)` components of a vector field emanating from equidistant points on the x-axis. If a single complex argument *z* is given, then `u = real (z)` and `v = imag (z)`.

The style to use for the plot can be defined with a line style *style* in a similar manner to the line styles used with the `plot` command.

The optional return value *h* provides a list of handles to the the parts of the vector field (body, arrow and marker).

```
phi = [0 : 15 : 360] * pi / 180;
feather (sin (phi), cos (phi))
```

See also: [plot], page 205, [quiver], page 218, [compass], page 220.

`pcolor (x, y, c)` [Function File]
`pcolor (c)` [Function File]

Density plot for given matrices *x*, and *y* from `meshgrid` and a matrix *c* corresponding to the *x* and *y* coordinates of the mesh's vertices. If *x* and *y* are vectors, then a typical vertex is (*x*(j), *y*(i), *c*(i,j)). Thus, columns of *c* correspond to different *x* values and rows of *c* correspond to different *y* values.

The `colormap` is scaled to the extents of *c*. Limits may be placed on the color axis by the command `caxis`, or by setting the `clim` property of the parent axis.

The face color of each cell of the mesh is determined by interpolating the values of *c* for the cell's vertices. Contrast this with `imagesc` which renders one cell for each element of *c*.

`shading` modifies an attribute determining the manner by which the face color of each cell is interpolated from the values of *c*, and the visibility of the cells' edges. By

default the attribute is "faceted", which renders a single color for each cell's face with the edge visible.

h is the handle to the surface object.

See also: [caxis], page 222, [contour], page 213, [meshgrid], page 229, [imagesc], page 488, [shading], page 231.

`area (x, y)` [Function File]
`area (x, y, lvl)` [Function File]
`area (..., prop, val, ...)` [Function File]
`area (y, ...)` [Function File]
`area (h, ...)` [Function File]
`h = area (...)` [Function File]

Area plot of cumulative sum of the columns of *y*. This shows the contributions of a value to a sum, and is functionally similar to `plot (x, cumsum (y, 2))`, except that the area under the curve is shaded.

If the *x* argument is omitted it is assumed to be given by `1 : rows (y)`. A value *lvl* can be defined that determines where the base level of the shading under the curve should be defined.

Additional arguments to the `area` function are passed to the `patch`. The optional return value *h* provides a handle to area series object representing the patches of the areas.

See also: [plot], page 205, [patch], page 246.

`comet (y)` [Function File]
`comet (x, y)` [Function File]
`comet (x, y, p)` [Function File]
`comet (ax, ...)` [Function File]

Produce a simple comet style animation along the trajectory provided by the input coordinate vectors (*x*, *y*), where *x* will default to the indices of *y*.

The speed of the comet may be controlled by *p*, which represents the time which passes as the animation passes from one point to the next. The default for *p* is 0.1 seconds.

If *ax* is specified the animation is produced in that axis rather than the `gca`.

The axis function may be used to change the axis limits of an existing plot and various other axis properties, such as the aspect ratio and the appearance of tic marks.

`axis (limits)` [Function File]

Set axis limits for plots.

The argument *limits* should be a 2, 4, or 6 element vector. The first and second elements specify the lower and upper limits for the x axis. The third and fourth specify the limits for the y-axis, and the fifth and sixth specify the limits for the z-axis.

Without any arguments, `axis` turns autoscaling on.

With one output argument, `x = axis` returns the current axes

The vector argument specifying limits is optional, and additional string arguments may be used to specify various axis properties. For example,

```
axis ([1, 2, 3, 4], "square");
```

forces a square aspect ratio, and

```
axis ("labely", "tic");
```

turns tic marks on for all axes and tic mark labels on for the y-axis only.

The following options control the aspect ratio of the axes.

`"square"` Force a square aspect ratio.

`"equal"` Force x distance to equal y-distance.

`"normal"` Restore the balance.

The following options control the way axis limits are interpreted.

`"auto"` Set the specified axes to have nice limits around the data or all if no axes are specified.

`"manual"` Fix the current axes limits.

`"tight"` Fix axes to the limits of the data.

The option `"image"` is equivalent to `"tight"` and `"equal"`.

The following options affect the appearance of tic marks.

`"on"` Turn tic marks and labels on for all axes.

`"off"` Turn tic marks off for all axes.

`"tic[xyz]"`
Turn tic marks on for all axes, or turn them on for the specified axes and off for the remainder.

`"label[xyz]"`
Turn tic labels on for all axes, or turn them on for the specified axes and off for the remainder.

`"nolabel"`
Turn tic labels off for all axes.

Note, if there are no tic marks for an axis, there can be no labels.

The following options affect the direction of increasing values on the axes.

`"ij"` Reverse y-axis, so lower values are nearer the top.

`"xy"` Restore y-axis, so higher values are nearer the top.

If an axes handle is passed as the first argument, then operate on this axes rather than the current axes.

Similarly the axis limits of the colormap can be changed with the caxis function.

caxis (*limits*) [Function File]
caxis (*h*, ...) [Function File]

Set color axis limits for plots.

The argument *limits* should be a 2 element vector specifying the lower and upper limits to assign to the first and last value in the colormap. Values outside this range are clamped to the first and last colormap entries.

If *limits* is 'auto', then automatic colormap scaling is applied, whereas if *limits* is 'manual' the colormap scaling is set to manual.

Called without any arguments to current color axis limits are returned.

If an axes handle is passed as the first argument, then operate on this axes rather than the current axes.

The `xlim`, `ylim`, and `zlim` functions may be used to get or set individual axis limits. Each has the same form.

xl = **xlim** () [Function File]
xlim (*xl*) [Function File]
m = **xlim** ('*mode*') [Function File]
xlim (*m*) [Function File]
xlim (*h*, ...) [Function File]

Get or set the limits of the x-axis of the current plot. Called without arguments `xlim` returns the x-axis limits of the current plot. If passed a two element vector *xl*, the limits of the x-axis are set to this value.

The current mode for calculation of the x-axis can be returned with a call `xlim ('mode')`, and can be either 'auto' or 'manual'. The current plotting mode can be set by passing either 'auto' or 'manual' as the argument.

If passed an handle as the first argument, then operate on this handle rather than the current axes handle.

See also: [ylim], page 223, [zlim], page 223, [set], page 245, [get], page 245, [gca], page 244.

15.1.1.1 Two-dimensional Function Plotting

Octave can plot a function from a function handle inline function or string defining the function without the user needing to explicitly create the data to be plotted. The function `fplot` also generates two-dimensional plots with linear axes using a function name and limits for the range of the x-coordinate instead of the x and y data. For example,

```
fplot (@sin, [-10, 10], 201);
```

produces a plot that is equivalent to the one above, but also includes a legend displaying the name of the plotted function.

fplot (*fn*, *limits*) [Function File]
fplot (*fn*, *limits*, *tol*) [Function File]
fplot (*fn*, *limits*, *n*) [Function File]
fplot (..., *fmt*) [Function File]

Plot a function *fn*, within the defined limits. *fn* an be either a string, a function handle or an inline function. The limits of the plot are given by *limits* of the form

[*xlo*, *xhi*] or [*xlo*, *xhi*, *ylo*, *yhi*]. *tol* is the default tolerance to use for the plot, and if *tol* is an integer it is assumed that it defines the number points to use in the plot. The *fmt* argument is passed to the plot command.

```
fplot ("cos", [0, 2*pi])
fplot ("[cos(x), sin(x)]", [0, 2*pi])
```

See also: [plot], page 205.

Other functions that can create two-dimensional plots directly from a function include `ezplot`, `ezcontour`, `ezcontourf` and `ezpolar`.

`ezplot` (*f*) [Function File]
`ezplot` (*fx*, *fy*) [Function File]
`ezplot` (. . ., *dom*) [Function File]
`ezplot` (. . ., *n*) [Function File]
`ezplot` (*h*, . . .) [Function File]
h = `ezplot` (. . .) [Function File]

Plots in two-dimensions the curve defined by *f*. The function *f* may be a string, inline function or function handle and can have either one or two variables. If *f* has one variable, then the function is plotted over the domain `-2*pi < x < 2*pi` with 500 points.

If *f* has two variables then `f(x,y) = 0` is calculated over the meshed domain `-2*pi < x | y < 2*pi` with 60 by 60 in the mesh. For example

```
ezplot (@(x, y) x .^ 2 - y .^ 2 - 1)
```

If two functions are passed as strings, inline functions or function handles, then the parametric function

```
x = fx (t)
y = fy (t)
```

is plotted over the domain `-2*pi < t < 2*pi` with 500 points.

If *dom* is a two element vector, it represents the minimum and maximum value of *x*, *y* and *t*. If it is a four element vector, then the minimum and maximum values of *x* and *t* are determined by the first two elements and the minimum and maximum of *y* by the second pair of elements.

n is a scalar defining the number of points to use in plotting the function.

The optional return value *h* provides a list of handles to the the line objects plotted.

See also: [plot], page 205, [ezplot3], page 232.

`ezcontour` (*f*) [Function File]
`ezcontour` (. . ., *dom*) [Function File]
`ezcontour` (. . ., *n*) [Function File]
`ezcontour` (*h*, . . .) [Function File]
h = `ezcontour` (. . .) [Function File]

Plots the contour lines of a function. *f* is a string, inline function or function handle with two arguments defining the function. By default the plot is over the domain `-2*pi < x < 2*pi` and `-2*pi < y < 2*pi` with 60 points in each dimension.

If *dom* is a two element vector, it represents the minimum and maximum value of both *x* and *y*. If *dom* is a four element vector, then the minimum and maximum value of *x* and *y* are specify separately.

n is a scalar defining the number of points to use in each dimension.

The optional return value *h* provides a list of handles to the the parts of the vector field (body, arrow and marker).

```
f = @(x,y) sqrt(abs(x .* y)) ./ (1 + x.^2 + y.^2);
ezcontour (f, [-3, 3]);
```

See also: [ezplot], page 224, [ezcontourf], page 225, [ezsurfc], page 234, [ezmeshc], page 233.

ezcontourf (*f*) [Function File]
ezcontourf (. . . , *dom*) [Function File]
ezcontourf (. . . , *n*) [Function File]
ezcontourf (*h*, . . .) [Function File]
h = **ezcontourf** (. . .) [Function File]

Plots the filled contour lines of a function. *f* is a string, inline function or function handle with two arguments defining the function. By default the plot is over the domain `-2*pi` < *x* < `2*pi` and `-2*pi` < *y* < `2*pi` with 60 points in each dimension.

If *dom* is a two element vector, it represents the minimum and maximum value of both *x* and *y*. If *dom* is a four element vector, then the minimum and maximum value of *x* and *y* are specify separately.

n is a scalar defining the number of points to use in each dimension.

The optional return value *h* provides a list of handles to the the parts of the vector field (body, arrow and marker).

```
f = @(x,y) sqrt(abs(x .* y)) ./ (1 + x.^2 + y.^2);
ezcontourf (f, [-3, 3]);
```

See also: [ezplot], page 224, [ezcontour], page 224, [ezsurfc], page 234, [ezmeshc], page 233.

ezpolar (*f*) [Function File]
ezpolar (. . . , *dom*) [Function File]
ezpolar (. . . , *n*) [Function File]
ezpolar (*h*, . . .) [Function File]
h = **ezpolar** (. . .) [Function File]

Plots in polar plot defined by a function. The function *f* is either a string, inline function or function handle with one arguments defining the function. By default the plot is over the domain `0` < *x* < `2*pi` with 60 points.

If *dom* is a two element vector, it represents the minimum and maximum value of both *t*. *n* is a scalar defining the number of points to use.

The optional return value *h* provides a list of handles to the the parts of the vector field (body, arrow and marker).

```
ezpolar (@(t) 1 + sin (t));
```

See also: [polar], page 218, [ezplot], page 224, [ezsurf], page 233, [ezmesh], page 232.

15.1.2 Three-Dimensional Plotting

The function `mesh` produces mesh surface plots. For example,

```
tx = ty = linspace (-8, 8, 41)';
[xx, yy] = meshgrid (tx, ty);
r = sqrt (xx .^ 2 + yy .^ 2) + eps;
tz = sin (r) ./ r;
mesh (tx, ty, tz);
```

produces the familiar "sombrero" plot shown in Figure 15.5. Note the use of the function `meshgrid` to create matrices of X and Y coordinates to use for plotting the Z data. The `ndgrid` function is similar to `meshgrid`, but works for N-dimensional matrices.

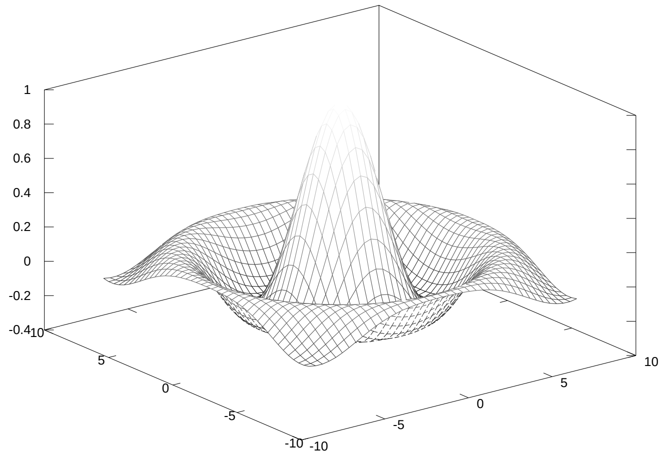

Figure 15.5: Mesh plot.

The `meshc` function is similar to `mesh`, but also produces a plot of contours for the surface.

The `plot3` function displays arbitrary three-dimensional data, without requiring it to form a surface. For example

```
t = 0:0.1:10*pi;
r = linspace (0, 1, numel (t));
z = linspace (0, 1, numel (t));
plot3 (r.*sin(t), r.*cos(t), z);
```

displays the spiral in three dimensions shown in Figure 15.6.

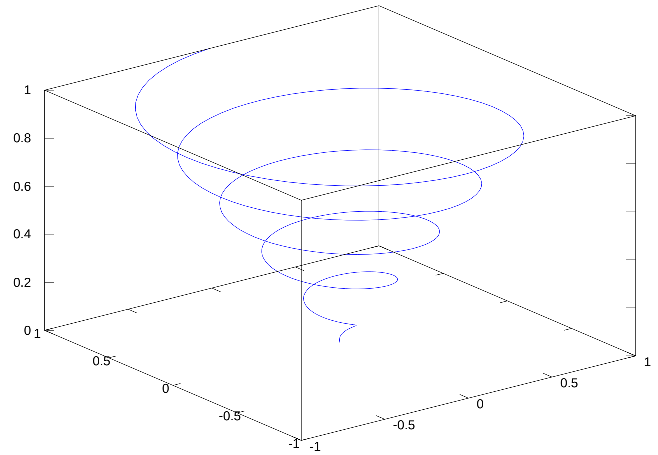

Figure 15.6: Three dimensional spiral.

Finally, the `view` function changes the viewpoint for three-dimensional plots.

`mesh` (*x*, *y*, *z*) [Function File]

Plot a mesh given matrices *x*, and *y* from `meshgrid` and a matrix *z* corresponding to the *x* and *y* coordinates of the mesh. If *x* and *y* are vectors, then a typical vertex is (*x*(j), *y*(i), *z*(i,j)). Thus, columns of *z* correspond to different *x* values and rows of *z* correspond to different *y* values.

See also: [meshgrid], page 229, [contour], page 213.

`meshc` (*x*, *y*, *z*) [Function File]

Plot a mesh and contour given matrices *x*, and *y* from `meshgrid` and a matrix *z* corresponding to the *x* and *y* coordinates of the mesh. If *x* and *y* are vectors, then a typical vertex is (*x*(j), *y*(i), *z*(i,j)). Thus, columns of *z* correspond to different *x* values and rows of *z* correspond to different *y* values.

See also: [meshgrid], page 229, [mesh], page 227, [contour], page 213.

`meshz` (*x*, *y*, *z*) [Function File]

Plot a curtain mesh given matrices *x*, and *y* from `meshgrid` and a matrix *z* corresponding to the *x* and *y* coordinates of the mesh. If *x* and *y* are vectors, then a typical vertex is (*x*(j), *y*(i), *z*(i,j)). Thus, columns of *z* correspond to different *x* values and rows of *z* correspond to different *y* values.

See also: [meshgrid], page 229, [mesh], page 227, [contour], page 213.

`hidden` (*mode*) [Function File]
`hidden` () [Function File]

Manipulation the mesh hidden line removal. Called with no argument the hidden line removal is toggled. The argument *mode* can be either 'on' or 'off' and the set of the hidden line removal is set accordingly.

See also: [mesh], page 227, [meshc], page 227, [surf], page 228.

`surf (x, y, z)` [Function File]

Plot a surface given matrices *x*, and *y* from `meshgrid` and a matrix *z* corresponding to the *x* and *y* coordinates of the mesh. If *x* and *y* are vectors, then a typical vertex is (*x*(j), *y*(i), *z*(i,j)). Thus, columns of *z* correspond to different *x* values and rows of *z* correspond to different *y* values.

See also: [mesh], page 227, [surface], page 246.

`surfc (x, y, z)` [Function File]

Plot a surface and contour given matrices *x*, and *y* from `meshgrid` and a matrix *z* corresponding to the *x* and *y* coordinates of the mesh. If *x* and *y* are vectors, then a typical vertex is (*x*(j), *y*(i), *z*(i,j)). Thus, columns of *z* correspond to different *x* values and rows of *z* correspond to different *y* values.

See also: [meshgrid], page 229, [surf], page 228, [contour], page 213.

`surfl (x, y, z)` [Function File]
`surfl (z)` [Function File]
`surfl (x, y, z, L)` [Function File]
`surfl (x, y, z, L, P)` [Function File]
`surfl (...,"light")` [Function File]

Plot a lighted surface given matrices *x*, and *y* from `meshgrid` and a matrix *z* corresponding to the *x* and *y* coordinates of the mesh. If *x* and *y* are vectors, then a typical vertex is (*x*(j), *y*(i), *z*(i,j)). Thus, columns of *z* correspond to different *x* values and rows of *z* correspond to different *y* values.

The light direction can be specified using *L*. It can be given as 2-element vector [azimuth, elevation] in degrees or as 3-element vector [lx, ly, lz]. The default value is rotated 45 counter-clockwise from the current view.

The material properties of the surface can specified using a 4-element vector *P* = [*AM D SP exp*] which defaults to *p* = [0.55 0.6 0.4 10].

"AM" strength of ambient light
"D" strength of diffuse reflection
"SP" strength of specular reflection
"EXP" specular exponent

The default lighting mode "cdata", changes the cdata property to give the impression of a lighted surface. Please note: the alternative "light" mode, which creates a light object to illuminate the surface is not implemented (yet).

Example:

```
colormap(bone);
surfl(peaks);
shading interp;
```

See also: [surf], page 228, [diffuse], page 229, [specular], page 229, [surface], page 246.

`surfnorm (x, y, z)` [Function File]
`surfnorm (z)` [Function File]
`[nx, ny, nz] = surfnorm (...)` [Function File]

`surfnorm (h, ...)` [Function File]

Find the vectors normal to a meshgridded surface. The meshed gridded surface is defined by *x*, *y*, and *z*. If *x* and *y* are not defined, then it is assumed that they are given by

```
[x, y] = meshgrid (1:size(z, 1),
                   1:size(z, 2));
```

If no return arguments are requested, a surface plot with the normal vectors to the surface is plotted. Otherwise the components of the normal vectors at the mesh gridded points are returned in *nx*, *ny*, and *nz*.

The normal vectors are calculated by taking the cross product of the diagonals of each of the quadrilaterals in the meshgrid to find the normal vectors of the centers of these quadrilaterals. The four nearest normal vectors to the meshgrid points are then averaged to obtain the normal to the surface at the meshgridded points.

An example of the use of `surfnorm` is

```
surfnorm (peaks (25));
```

See also: [surf], page 228, [quiver3], page 219.

`diffuse (sx, sy, sz, l)` [Function File]

Calculate diffuse reflection strength of a surface defined by the normal vector elements *sx*, *sy*, *sz*. The light vector can be specified using parameter *L*. It can be given as 2-element vector [azimuth, elevation] in degrees or as 3-element vector [lx, ly, lz].

See also: [specular], page 229, [surfl], page 228.

`specular (sx, sy, sz, l, v)` [Function File]
`specular (sx, sy, sz, l, v, se)` [Function File]

Calculate specular reflection strength of a surface defined by the normal vector elements *sx*, *sy*, *sz* using Phong's approximation. The light and view vectors can be specified using parameter *L* and *V* respectively. Both can be given as 2-element vectors [azimuth, elevation] in degrees or as 3-element vector [x, y, z]. An optional 6th argument describes the specular exponent (spread) *se*.

See also: [surfl], page 228, [diffuse], page 229.

`[xx, yy, zz] = meshgrid (x, y, z)` [Function File]
`[xx, yy] = meshgrid (x, y)` [Function File]
`[xx, yy] = meshgrid (x)` [Function File]

Given vectors of *x* and *y* and *z* coordinates, and returning 3 arguments, return three-dimensional arrays corresponding to the *x*, *y*, and *z* coordinates of a mesh. When returning only 2 arguments, return matrices corresponding to the *x* and *y* coordinates of a mesh. The rows of *xx* are copies of *x*, and the columns of *yy* are copies of *y*. If *y* is omitted, then it is assumed to be the same as *x*, and *z* is assumed the same as *y*.

See also: [mesh], page 227, [contour], page 213.

`[y1, y2, ..., yn] = ndgrid (x1, x2, ..., xn)` [Function File]
`[y1, y2, ..., yn] = ndgrid (x)` [Function File]

Given n vectors *x1*, ... xn, `ndgrid` returns n arrays of dimension n. The elements of the i-th output argument contains the elements of the vector xi repeated over all

dimensions different from the i-th dimension. Calling ndgrid with only one input argument *x* is equivalent of calling ndgrid with all n input arguments equal to *x*:

[*y1*, *y2*, . . . , *y*n] = ndgrid (*x*, . . . , *x*)

See also: [meshgrid], page 229.

`plot3 (args)` [Function File]

Produce three-dimensional plots. Many different combinations of arguments are possible. The simplest form is

```
plot3 (x, y, z)
```

in which the arguments are taken to be the vertices of the points to be plotted in three dimensions. If all arguments are vectors of the same length, then a single continuous line is drawn. If all arguments are matrices, then each column of the matrices is treated as a separate line. No attempt is made to transpose the arguments to make the number of rows match.

If only two arguments are given, as

```
plot3 (x, c)
```

the real and imaginary parts of the second argument are used as the *y* and *z* coordinates, respectively.

If only one argument is given, as

```
plot3 (c)
```

the real and imaginary parts of the argument are used as the *y* and *z* values, and they are plotted versus their index.

Arguments may also be given in groups of three as

```
plot3 (x1, y1, z1, x2, y2, z2, ...)
```

in which each set of three arguments is treated as a separate line or set of lines in three dimensions.

To plot multiple one- or two-argument groups, separate each group with an empty format string, as

```
plot3 (x1, c1, "", c2, "", ...)
```

An example of the use of `plot3` is

```
z = [0:0.05:5];
plot3 (cos(2*pi*z), sin(2*pi*z), z, ";helix;");
plot3 (z, exp(2i*pi*z), ";complex sinusoid;");
```

See also: [plot], page 205, [xlabel], page 236, [ylabel], page 236, [zlabel], page 236, [title], page 236, [print], page 239.

`view (azimuth, elevation)` [Function File]
`view (dims)` [Function File]
`[azimuth, elevation] = view ()` [Function File]

Set or get the viewpoint for the current axes.

`slice (x, y, z, v, sx, sy, sz)` [Function File]
`slice (x, y, z, v, xi, yi, zi)` [Function File]
`slice (v, sx, sy, sz)` [Function File]
`slice (v, xi, yi, zi)` [Function File]
`h = slice (...)` [Function File]
`h = slice (..., method)` [Function File]

Plot slices of 3D data/scalar fields. Each element of the 3-dimensional array *v* represents a scalar value at a location given by the parameters *x*, *y*, and *z*. The parameters *x*, *x*, and *z* are either 3-dimensional arrays of the same size as the array *v* in the "meshgrid" format or vectors. The parameters *xi*, etc. respect a similar format to *x*, etc., and they represent the points at which the array *vi* is interpolated using interp3. The vectors *sx*, *sy*, and *sz* contain points of orthogonal slices of the respective axes.

If *x*, *y*, *z* are omitted, they are assumed to be `x = 1:size (v, 2)`, `y = 1:size (v, 1)` and `z = 1:size (v, 3)`.

Method is one of:

`"nearest"`
: Return the nearest neighbor.

`"linear"` Linear interpolation from nearest neighbors.

`"cubic"` Cubic interpolation from four nearest neighbors (not implemented yet).

`"spline"` Cubic spline interpolation—smooth first and second derivatives throughout the curve.

The default method is `"linear"`. The optional return value *h* is a vector of handles to the surface graphic objects.

Examples:

```
[x, y, z] = meshgrid (linspace (-8, 8, 32));
v = sin (sqrt (x.^2 + y.^2 + z.^2)) ./ (sqrt (x.^2 + y.^2 + z.^2));
slice (x, y, z, v, [], 0, []);
[xi, yi] = meshgrid (linspace (-7, 7));
zi = xi + yi;
slice (x, y, z, v, xi, yi, zi);
```

See also: [interp3], page 460, [surface], page 246, [pcolor], page 220.

`ribbon (x, y, width)` [Function File]
`ribbon (y)` [Function File]
`h = ribbon (...)` [Function File]

Plot a ribbon plot for the columns of *y* vs. *x*. The optional parameter *width* specifies the width of a single ribbon (default is 0.75). If *x* is omitted, a vector containing the row numbers is assumed (1:rows(Y)). If requested, return a vector *h* of the handles to the surface objects.

See also: [gca], page 244, [colorbar], page 238.

`shading (type)` [Function File]
`shading (ax, ...)` [Function File]

Set the shading of surface or patch graphic objects. Valid arguments for *type* are

"flat" Single colored patches with invisible edges.

"faceted"
Single colored patches with visible edges.

"interp" Color between patch vertices are interpolated and the patch edges are invisible.

If *ax* is given the shading is applied to axis *ax* instead of the current axis.

15.1.2.1 Three-dimensional Function Plotting

`ezplot3 (fx, fy, fz)` [Function File]
`ezplot3 (..., dom)` [Function File]
`ezplot3 (..., n)` [Function File]
`ezplot3 (h, ...)` [Function File]
`h = ezplot3 (...)` [Function File]

Plots in three-dimensions the curve defined parametrically. *fx*, *fy*, and *fz* are strings, inline functions or function handles with one arguments defining the function. By default the plot is over the domain `-2*pi < x < 2*pi` with 60 points.

If *dom* is a two element vector, it represents the minimum and maximum value of *t*. *n* is a scalar defining the number of points to use.

The optional return value *h* provides a list of handles to the the parts of the vector field (body, arrow and marker).

```
fx = @(t) cos (t);
fy = @(t) sin (t);
fz = @(t) t;
ezplot3 (fx, fy, fz, [0, 10*pi], 100);
```

See also: [plot3], page 230, [ezplot], page 224, [ezsurf], page 233, [ezmesh], page 232.

`ezmesh (f)` [Function File]
`ezmesh (fx, fy, fz)` [Function File]
`ezmesh (..., dom)` [Function File]
`ezmesh (..., n)` [Function File]
`ezmesh (..., 'circ')` [Function File]
`ezmesh (h, ...)` [Function File]
`h = ezmesh (...)` [Function File]

Plots the mesh defined by a function. *f* is a string, inline function or function handle with two arguments defining the function. By default the plot is over the domain `-2*pi < x < 2*pi` and `-2*pi < y < 2*pi` with 60 points in each dimension.

If *dom* is a two element vector, it represents the minimum and maximum value of both *x* and *y*. If *dom* is a four element vector, then the minimum and maximum value of *x* and *y* are specify separately.

n is a scalar defining the number of points to use in each dimension.

If three functions are passed, then plot the parametrically defined function `[fx (s, t), fy (s, t), fz (s, t)]`.

If the argument 'circ' is given, then the function is plotted over a disk centered on the middle of the domain *dom*.

The optional return value *h* provides a list of handles to the the parts of the vector field (body, arrow and marker).

```
f = @(x,y) sqrt(abs(x .* y)) ./ (1 + x.^2 + y.^2);
ezmesh (f, [-3, 3]);
```

An example of a parametrically defined function is

```
fx = @(s,t) cos (s) .* cos(t);
fy = @(s,t) sin (s) .* cos(t);
fz = @(s,t) sin(t);
ezmesh (fx, fy, fz, [-pi, pi, -pi/2, pi/2], 20);
```

See also: [ezplot], page 224, [ezsurf], page 233, [ezsurfc], page 234, [ezmeshc], page 233.

`ezmeshc` (*f*) [Function File]
`ezmeshc` (*fx*, *fy*, *fz*) [Function File]
`ezmeshc` (..., *dom*) [Function File]
`ezmeshc` (..., *n*) [Function File]
`ezmeshc` (..., 'circ') [Function File]
`ezmeshc` (*h*, ...) [Function File]
h = `ezmeshc` (...) [Function File]

Plots the mesh and contour lines defined by a function. *f* is a string, inline function or function handle with two arguments defining the function. By default the plot is over the domain `-2*pi < x < 2*pi` and `-2*pi < y < 2*pi` with 60 points in each dimension.

If *dom* is a two element vector, it represents the minimum and maximum value of both *x* and *y*. If *dom* is a four element vector, then the minimum and maximum value of *x* and *y* are specify separately.

n is a scalar defining the number of points to use in each dimension.

If three functions are passed, then plot the parametrically defined function `[fx (s, t), fy (s, t), fz (s, t)]`.

If the argument 'circ' is given, then the function is plotted over a disk centered on the middle of the domain *dom*.

The optional return value *h* provides a list of handles to the the parts of the vector field (body, arrow and marker).

```
f = @(x,y) sqrt(abs(x .* y)) ./ (1 + x.^2 + y.^2);
ezmeshc (f, [-3, 3]);
```

See also: [ezplot], page 224, [ezsurfc], page 234, [ezsurf], page 233, [ezmesh], page 232.

`ezsurf` (*f*) [Function File]
`ezsurf` (*fx*, *fy*, *fz*) [Function File]
`ezsurf` (..., *dom*) [Function File]
`ezsurf` (..., *n*) [Function File]
`ezsurf` (..., 'circ') [Function File]
`ezsurf` (*h*, ...) [Function File]
h = `ezsurf` (...) [Function File]

Plots the surface defined by a function. *f* is a string, inline function or function handle with two arguments defining the function. By default the plot is over the domain `-2*pi < x < 2*pi` and `-2*pi < y < 2*pi` with 60 points in each dimension.

If *dom* is a two element vector, it represents the minimum and maximum value of both *x* and *y*. If *dom* is a four element vector, then the minimum and maximum value of *x* and *y* are specify separately.

n is a scalar defining the number of points to use in each dimension.

If three functions are passed, then plot the parametrically defined function `[fx (s, t), fy (s, t), fz (s, t)]`.

If the argument 'circ' is given, then the function is plotted over a disk centered on the middle of the domain *dom*.

The optional return value *h* provides a list of handles to the the parts of the vector field (body, arrow and marker).

```
f = @(x,y) sqrt(abs(x .* y)) ./ (1 + x.^2 + y.^2);
ezsurf (f, [-3, 3]);
```

An example of a parametrically defined function is

```
fx = @(s,t) cos (s) .* cos(t);
fy = @(s,t) sin (s) .* cos(t);
fz = @(s,t) sin(t);
ezsurf (fx, fy, fz, [-pi, pi, -pi/2, pi/2], 20);
```

See also: [ezplot], page 224, [ezmesh], page 232, [ezsurfc], page 234, [ezmeshc], page 233.

`ezsurfc` (*f*) [Function File]
`ezsurfc` (*fx*, *fy*, *fz*) [Function File]
`ezsurfc` (..., *dom*) [Function File]
`ezsurfc` (..., *n*) [Function File]
`ezsurfc` (..., *'circ'*) [Function File]
`ezsurfc` (*h*, ...) [Function File]
h = `ezsurfc` (...) [Function File]

Plots the surface and contour lines defined by a function. *f* is a string, inline function or function handle with two arguments defining the function. By default the plot is over the domain `-2*pi < x < 2*pi` and `-2*pi < y < 2*pi` with 60 points in each dimension.

If *dom* is a two element vector, it represents the minimum and maximum value of both *x* and *y*. If *dom* is a four element vector, then the minimum and maximum value of *x* and *y* are specify separately.

n is a scalar defining the number of points to use in each dimension.

If three functions are passed, then plot the parametrically defined function `[fx (s, t), fy (s, t), fz (s, t)]`.

If the argument 'circ' is given, then the function is plotted over a disk centered on the middle of the domain *dom*.

The optional return value *h* provides a list of handles to the the parts of the vector field (body, arrow and marker).

```
f = @(x,y) sqrt(abs(x .* y)) ./ (1 + x.^2 + y.^2);
ezsurfc (f, [-3, 3]);
```

See also: [ezplot], page 224, [ezmeshc], page 233, [ezsurf], page 233, [ezmesh], page 232.

15.1.2.2 Three-dimensional Geometric Shapes

`cylinder` [Function File]
`cylinder (r)` [Function File]
`cylinder (r, n)` [Function File]
`[x, y, z] = cylinder (...)` [Function File]
`cylinder (ax, ...)` [Function File]

Generates three matrices in `meshgrid` format, such that `surf (x, y, z)` generates a unit cylinder. The matrices are of size *n*+1-by-*n*+1. *r* is a vector containing the radius along the z-axis. If *n* or *r* are omitted then default values of 20 or [1 1] are assumed.

Called with no return arguments, `cylinder` calls directly `surf (x, y, z)`. If an axes handle *ax* is passed as the first argument, the surface is plotted to this set of axes.

Examples:

```
disp ("plotting a cone")
[x, y, z] = cylinder (10:-1:0,50);
surf (x, y, z);
```

See also: [sphere], page 235.

`[x, y, z] = sphere (n)` [Function File]
`sphere (h, ...)` [Function File]

Generates three matrices in `meshgrid` format, such that `surf (x, y, z)` generates a unit sphere. The matrices of *n*+1-by-*n*+1. If *n* is omitted then a default value of 20 is assumed.

Called with no return arguments, `sphere` call directly `surf (x, y, z)`. If an axes handle is passed as the first argument, the surface is plotted to this set of axes.

See also: [peaks], page 243.

`[x, y, z] = ellipsoid (xc,yc, zc, xr, yr, zr, n)` [Function File]
`ellipsoid (h, ...)` [Function File]

Generate three matrices in `meshgrid` format that define an ellipsoid. Called with no return arguments, `ellipsoid` calls directly `surf (x, y, z)`. If an axes handle is passed as the first argument, the surface is plotted to this set of axes.

See also: [sphere], page 235.

15.1.3 Plot Annotations

You can add titles, axis labels, legends, and arbitrary text to an existing plot. For example,

```
x = -10:0.1:10;
plot (x, sin (x));
title ("sin(x) for x = -10:0.1:10");
xlabel ("x");
ylabel ("sin (x)");
text (pi, 0.7, "arbitrary text");
legend ("sin (x)");
```

The functions `grid` and `box` may also be used to add grid and border lines to the plot. By default, the grid is off and the border lines are on.

`title` (*title*) [Function File]
`title` (*title*, *p1*, *v1*, . . .) [Function File]

Create a title object and return a handle to it.

`legend` (*st1*, *st2*, . . .) [Function File]
`legend` (*st1*, *st2*, . . ., "*location*", *pos*) [Function File]
`legend` (*matstr*) [Function File]
`legend` (*matstr*, "*location*", *pos*) [Function File]
`legend` (*cell*) [Function File]
`legend` (*cell*, "*location*", *pos*) [Function File]
`legend` (*'func'*) [Function File]

Display a legend for the current axes using the specified strings as labels. Legend entries may be specified as individual character string arguments, a character array, or a cell array of character strings. Legend works on line graphs, bar graphs, etc. A plot must exist before legend is called.

The optional parameter *pos* specifies the location of the legend as follows:

north	center top
south	center bottom
east	right center
west	left center
northeast	right top (default)
northwest	left top
southeast	right bottom
southwest	left bottom
outside	can be appended to any location string

Some specific functions are directly available using *func*:

"show" Show legends from the plot

"hide"
"off" Hide legends from the plot

"boxon" Draw a box around legends

"boxoff" Withdraw the box around legends

"left" Text is to the left of the keys

"right" Text is to the right of the keys

h = `text` (*x*, *y*, *label*) [Function File]
h = `text` (*x*, *y*, *z*, *label*) [Function File]
h = `text` (*x*, *y*, *label*, *p1*, *v1*, . . .) [Function File]
h = `text` (*x*, *y*, *z*, *label*, *p1*, *v1*, . . .) [Function File]

Create a text object with text *label* at position *x*, *y*, *z* on the current axes. Property-value pairs following *label* may be used to specify the appearance of the text.

See Section 15.2.2.5 [Text Properties], page 252 for the properties that you can set.

`xlabel` (*string*) [Function File]
`ylabel` (*string*) [Function File]
`zlabel` (*string*) [Function File]
`xlabel` (*h*, *string*) [Function File]

Specify x, y, and z axis labels for the current figure. If *h* is specified then label the axis defined by *h*.

See also: [plot], page 205, [semilogx], page 208, [semilogy], page 208, [loglog], page 208, [polar], page 218, [mesh], page 227, [contour], page 213, [bar], page 208, [stairs], page 210, [title], page 236.

`clabel` (*c*, *h*) [Function File]
`clabel` (*c*, *h*, *v*) [Function File]
`clabel` (*c*, *h*, "*manual*") [Function File]
`clabel` (*c*) [Function File]
`clabel` (*c*, *h*) [Function File]
`clabel` (. . ., *prop*, *val*, . . .) [Function File]
h = `clabel` (. . .) [Function File]

Adds labels to the contours of a contour plot. The contour plot is specified by the contour matrix *c* and optionally the contourgroup object *h* that are returned by `contour`, `contourf` and `contour3`. The contour labels are rotated and placed in the contour itself.

By default, all contours are labelled. However, the contours to label can be specified by the vector *v*. If the "manual" argument is given then the contours to label can be selected with the mouse.

Additional property/value pairs that are valid properties of text objects can be given and are passed to the underlying text objects. Additionally, the property "LabelSpacing" is available allowing the spacing between labels on a contour (in points) to be specified. The default is 144 points, or 2 inches.

The returned value *h* is the set of text object that represent the contour labels. The "userdata" property of the text objects contains the numerical value of the contour label.

An example of the use of `clabel` is

```
[c, h] = contour (peaks(), -4 : 6);
clabel (c, h, -4 : 2 : 6, 'fontsize', 12);
```

See also: [contour], page 213, [contourf], page 214, [contour3], page 215, [meshc], page 227, [surfc], page 228, [text], page 236.

`box` (*arg*) [Function File]
`box` (*h*, . . .) [Function File]

Control the display of a border around the plot. The argument may be either `"on"` or `"off"`. If it is omitted, the current box state is toggled.

See also: [grid], page 237.

`grid` (*arg*) [Function File]
`grid` ("*minor*", *arg2*) [Function File]

grid (*hax*, . . .) [Function File]

Force the display of a grid on the plot. The argument may be either `"on"`, or `"off"`. If it is omitted, the current grid state is toggled.

If *arg* is `"minor"` then the minor grid is toggled. When using a minor grid a second argument *arg2* is allowed, which can be either `"on"` or `"off"` to explicitly set the state of the minor grid.

If the first argument is an axis handle, *hax*, operate on the specified axis object.

See also: [plot], page 205.

colorbar (*s*) [Function File]
colorbar ("*peer*", *h*, . . .) [Function File]

Adds a colorbar to the current axes. Valid values for *s* are

"EastOutside"
: Place the colorbar outside the plot to the right. This is the default.

"East"
: Place the colorbar inside the plot to the right.

"WestOutside"
: Place the colorbar outside the plot to the left.

"West"
: Place the colorbar inside the plot to the left.

"NorthOutside"
: Place the colorbar above the plot.

"North"
: Place the colorbar at the top of the plot.

"SouthOutside"
: Place the colorbar under the plot.

"South"
: Place the colorbar at the bottom of the plot.

"Off", "None"
: Remove any existing colorbar from the plot.

If the argument "peer" is given, then the following argument is treated as the axes handle on which to add the colorbar.

15.1.4 Multiple Plots on One Page

Octave can display more than one plot in a single figure. The simplest way to do this is to use the `subplot` function to divide the plot area into a series of subplot windows that are indexed by an integer. For example,

```
subplot (2, 1, 1)
fplot (@sin, [-10, 10]);
subplot (2, 1, 2)
fplot (@cos, [-10, 10]);
```

creates a figure with two separate axes, one displaying a sine wave and the other a cosine wave. The first call to subplot divides the figure into two plotting areas (two rows and one column) and makes the first plot area active. The grid of plot areas created by `subplot` is numbered in column-major order (top to bottom, left to right).

`subplot` (*rows*, *cols*, *index*) [Function File]
`subplot` (*rcn*) [Function File]

Set up a plot grid with *cols* by *rows* subwindows and plot in location given by *index*.

If only one argument is supplied, then it must be a three digit value specifying the location in digits 1 (rows) and 2 (columns) and the plot index in digit 3.

The plot index runs row-wise. First all the columns in a row are filled and then the next row is filled.

For example, a plot with 2 by 3 grid will have plot indices running as follows:

1	2	3
4	5	6

See also: [plot], page 205.

15.1.5 Multiple Plot Windows

You can open multiple plot windows using the `figure` function. For example

```
figure (1);
fplot (@sin, [-10, 10]);
figure (2);
fplot (@cos, [-10, 10]);
```

creates two figures, with the first displaying a sine wave and the second a cosine wave. Figure numbers must be positive integers.

`figure` (*n*) [Function File]
`figure` (*n*, *property*, *value*, ...) [Function File]

Set the current plot window to plot window *n*. If no arguments are specified, the next available window number is chosen.

Multiple property-value pairs may be specified for the figure, but they must appear in pairs.

15.1.6 Printing Plots

The `print` command allows you to save plots in a variety of formats. For example,

```
print -deps foo.eps
```

writes the current figure to an encapsulated PostScript file called '`foo.eps`'.

`print` () [Function File]
`print` (*options*) [Function File]
`print` (*filename*, *options*) [Function File]
`print` (*h*, *filename*, *options*) [Function File]

Print a graph, or save it to a file

filename defines the file name of the output file. If no filename is specified, the output is sent to the printer.

h specifies the figure handle. If no handle is specified the handle for the current figure is used.

options:

`-P`*`printer`*
: Set the *printer* name to which the graph is sent if no *filename* is specified.

`-G`*`ghostscript_command`*
: Specify the command for calling Ghostscript. For Unix and Windows, the defaults are 'gs' and 'gswin32c', respectively.

`-color`
`-mono`
: Monochrome or color lines.

`-solid`
`-dashed`
: Solid or dashed lines.

`-portrait`
`-landscape`
: Specify the orientation of the plot for printed output.

`-d`*`device`*
: Output device, where *device* is one of:

  `ps`
  `ps2`
  `psc`
  `psc2`
  : Postscript (level 1 and 2, mono and color)

  `eps`
  `eps2`
  `epsc`
  `epsc2`
  : Encapsulated postscript (level 1 and 2, mono and color)

  `tex`
  `epslatex`
  `epslatexstandalone`
  `pstex`
  `pslatex`
  : Generate a LaTeX (or TeX) file for labels, and eps/ps for graphics. The file produced by `epslatexstandalone` can be processed directly by LaTeX. The other formats are intended to be included in a LaTeX (or TeX) document. The `tex` device is the same as the `epslatex` device.

  `ill`
  `aifm`
  : Adobe Illustrator

  `cdr`
  `corel`
  : CorelDraw

  `dxf`
  : AutoCAD

  `emf`
  `meta`
  : Microsoft Enhanced Metafile

`fig` XFig. If this format is selected the additional options `-textspecial` or `-textnormal` can be used to control whether the special flag should be set for the text in the figure (default is `-textnormal`).

`hpgl` HP plotter language

`mf` Metafont

`png` Portable network graphics

`jpg`
`jpeg` JPEG image

`gif` GIF image

`pbm` PBMplus

`svg` Scalable vector graphics

`pdf` Portable document format

If the device is omitted, it is inferred from the file extension, or if there is no filename it is sent to the printer as postscript.

`-d`*`gs_device`*
Additional devices are supported by Ghostscript. Some examples are;

`ljet2p` HP LaserJet IIP

`ljet3` HP LaserJet III

`deskjet` HP DeskJet and DeskJet Plus

`cdj550` HP DeskJet 550C

`paintjet` HP PointJet

`pcx24b` 24-bit color PCX file format

`ppm` Portable Pixel Map file format

For a complete list, type 'system ("gs -h")' to see what formats and devices are available.

When the ghostscript is sent to a printer the size is determined by the figure's "papersize" property. When the ghostscript output is sent to a file the size is determined by the figure's "paperposition" property.

`-r`*`NUM`* Resolution of bitmaps in pixels per inch. For both metafiles and SVG the default is the screen resolution, for other it is 150 dpi. To specify screen resolution, use "-r0".

`-tight` Forces a tight bounding box for eps-files. Since the ghostscript devices are conversion of an eps-file, this option works the those devices as well.

`-S`*`xsize,ysize`*
Plot size in pixels for EMF, GIF, JPEG, PBM, PNG and SVG. If using the command form of the print function, you must quote the *xsize,ysize* option. For example, by writing `"-S640,480"`. The size defaults to that specified by the figure's paperposition property.

`-F`*fontname*
`-F`*fontname*`:`*size*
`-F:`*size* *fontname* set the postscript font (for use with postscript, aifm, corel and fig). By default, 'Helvetica' is set for PS/Aifm, and 'SwitzerlandLight' for Corel. It can also be 'Times-Roman'. *size* is given in points. *fontname* is ignored for the fig device.

The filename and options can be given in any order.

`orient (`*orientation*`)` [Function File]

Set the default print orientation. Valid values for *orientation* include `"landscape"`, `"portrait"`, and `"tall"`.

The `"tall"` option sets the orientation to portait and fills the page with the plot, while leaving a 0.25in border.

If called with no arguments, return the default print orientation.

15.1.7 Interacting with plots

The user can select points on a plot with the `ginput` function or selection the position at which to place text on the plot with the `gtext` function using the mouse.

`[`*x*`,` *y*`,` *buttons*`] = ginput (`*n*`)` [Function File]

Return which mouse buttons were pressed and keys were hit on the current figure. If *n* is defined, then wait for *n* mouse clicks before returning. If *n* is not defined, then `ginput` will loop until the return key is pressed.

b `= waitforbuttonpress ()` [Function File]

Wait for button or mouse press.over a figure window. The value of *b* returns 0 if a mouse button was pressed or 1 is a key was pressed.

See also: [ginput], page 242.

`gtext (`*s*`)` [Function File]
`gtext ({`*s1*`;` *s2*`; ...})` [Function File]
`gtext (...,` *prop*`,` *val*`)` [Function File]

Place text on the current figure using the mouse. The text is defined by the string *s*. If *s* is a cell array, each element of the cell array is written to a separate line. Additional arguments are passed to the underlying text object as properties.

See also: [ginput], page 242, [text], page 236.

15.1.8 Test Plotting Functions

The functions `sombrero` and `peaks` provide a way to check that plotting is working. Typing either `sombrero` or `peaks` at the Octave prompt should display a three-dimensional plot.

`sombrero (`*n*`)` [Function File]

Produce the familiar three-dimensional sombrero plot using *n* grid lines. If *n* is omitted, a value of 41 is assumed.

The function plotted is

```
z = sin (sqrt (x^2 + y^2)) / (sqrt (x^2 + y^2))
```

See also: [surf], page 228, [meshgrid], page 229, [mesh], page 227.

peaks () [Function File]
peaks (*n*) [Function File]
peaks (*x*, *y*) [Function File]
z = peaks (...) [Function File]
[*x*, *y*, *z*] = peaks (...) [Function File]

Generate a function with lots of local maxima and minima. The function has the form

$f(x,y) = 3(1-x)^2 e^{\left(-x^2-(y+1)^2\right)} - 10\left(\frac{x}{5} - x^3 - y^5\right)\right) - \frac{1}{3} e^{\left(-(x+1)^2-y^2\right)}$

Called without a return argument, `peaks` plots the surface of the above function using `mesh`. If *n* is a scalar, the `peaks` returns the values of the above function on a *n*-by-*n* mesh over the range `[-3,3]`. The default value for *n* is 49.

If *n* is a vector, then it represents the *x* and *y* values of the grid on which to calculate the above function. The *x* and *y* values can be specified separately.

See also: [surf], page 228, [mesh], page 227, [meshgrid], page 229.

15.2 Advanced Plotting

15.2.1 Graphics Objects

Plots in Octave are constructed from the following *graphics objects*. Each graphics object has a set of properties that define its appearance and may also contain links to other graphics objects. Graphics objects are only referenced by a numeric index, or *handle*.

root figure The parent of all figure objects. The index for the root figure is defined to be 0.

figure A figure window.

axes A set of axes. This object is a child of a figure object and may be a parent of line, text, image, patch, or surface objects.

line A line in two or three dimensions.

text Text annotations.

image A bitmap image.

patch A filled polygon, currently limited to two dimensions.

surface A three-dimensional surface.

To determine whether a variable is a graphics object index or a figure index, use the functions `ishandle` and `isfigure`.

ishandle (*h*) [Built-in Function]

Return true if *h* is a graphics handle and false otherwise.

ishghandle (*h*) [Function File]

Return true if *h* is a graphics handle and false otherwise.

isfigure (*h*) [Function File]

Return true if *h* is a graphics handle that contains a figure object and false otherwise.

The function gcf returns an index to the current figure object, or creates one if none exists. Similarly, gca returns the current axes object, or creates one (and its parent figure object) if none exists.

gcf () [Function File]

Return the current figure handle. If a figure does not exist, create one and return its handle. The handle may then be used to examine or set properties of the figure. For example,

```
fplot (@sin, [-10, 10]);
fig = gcf ();
set (fig, "visible", "off");
```

plots a sine wave, finds the handle of the current figure, and then makes that figure invisible. Setting the visible property of the figure to "on" will cause it to be displayed again.

See also: [get], page 245, [set], page 245.

gca () [Function File]

Return a handle to the current axis object. If no axis object exists, create one and return its handle. The handle may then be used to examine or set properties of the axes. For example,

```
ax = gca ();
set (ax, "position", [0.5, 0.5, 0.5, 0.5]);
```

creates an empty axes object, then changes its location and size in the figure window.

See also: [get], page 245, [set], page 245.

The get and set functions may be used to examine and set properties for graphics objects. For example,

```
get (0)
    ⇒ ans =
       {
         type = root
         currentfigure = [](0x0)
         children = [](0x0)
         visible = on
...
       }
```

returns a structure containing all the properties of the root figure. As with all functions in Octave, the structure is returned by value, so modifying it will not modify the internal root figure plot object. To do that, you must use the set function. Also, note that in this case, the currentfigure property is empty, which indicates that there is no current figure window.

The get function may also be used to find the value of a single property. For example,

```
get (gca (), "xlim")
    ⇒ [ 0 1 ]
```

returns the range of the x-axis for the current axes object in the current figure.

To set graphics object properties, use the set function. For example,

```
set (gca (), "xlim", [-10, 10]);
```

sets the range of the x-axis for the current axes object in the current figure to '[-10, 10]'. Additionally, calling set with a graphics object index as the only argument returns a structure containing the default values for all the properties for the given object type. For example,

```
set (gca ())
```

returns a structure containing the default property values for axes objects.

`get` (*h*, *p*) [Built-in Function]

Return the named property *p* from the graphics handle *h*. If *p* is omitted, return the complete property list for *h*. If *h* is a vector, return a cell array including the property values or lists respectively.

`set` (*h*, *p*, *v*, ...) [Built-in Function]

Set the named property value or vector *p* to the value *v* for the graphics handle *h*.

parent = `ancestor` (*h*, *type*) [Function File]
parent = `ancestor` (*h*, *type*, *'toplevel'*) [Function File]

Return the first ancestor of handle object *h* whose type matches *type*, where *type* is a character string. If *type* is a cell array of strings, return the first parent whose type matches any of the given type strings.

If the handle object *h* is of type *type*, return *h*.

If `"toplevel"` is given as a 3rd argument, return the highest parent in the object hierarchy that matches the condition, instead of the first (nearest) one.

See also: [get], page 245, [set], page 245.

h = `allchild` (*handles*) [Function File]

Find all children, including hidden children, of a graphics object.

This function is similar to `get (h, "children")`, but also returns includes hidden objects. If *handles* is a scalar, *h* will be a vector. Otherwise, *h* will be a cell matrix of the same size as *handles* and each cell will contain a vector of handles.

See also: [get], page 245, [set], page 245, [findall], page 257, [findobj], page 256.

You can create axes, line, and patch objects directly using the `axes`, `line`, and `patch` functions. These objects become children of the current axes object.

`axes` () [Function File]
`axes` (*property*, *value*, ...) [Function File]
`axes` (*h*) [Function File]

Create an axes object and return a handle to it.

`line` () [Function File]
`line` (*x*, *y*) [Function File]
`line` (*x*, *y*, *z*) [Function File]
`line` (*x*, *y*, *z*, *property*, *value*, ...) [Function File]

Create line object from *x* and *y* and insert in current axes object. Return a handle (or vector of handles) to the line objects created.

Multiple property-value pairs may be specified for the line, but they must appear in pairs.

`patch ()` [Function File]
`patch (`*x*, *y*, *c*`)` [Function File]
`patch (`*x*, *y*, *z*, *c*`)` [Function File]
`patch (`*fv*`)` [Function File]
`patch (`*'Faces'*, *f*, *'Vertices'*, *v*, ...`)` [Function File]
`patch (`..., *prop*, *val*`)` [Function File]
`patch (`*h*, ...`)` [Function File]
h `= patch (`...`)` [Function File]

Create patch object from *x* and *y* with color *c* and insert in the current axes object. Return handle to patch object.

For a uniform colored patch, *c* can be given as an RGB vector, scalar value referring to the current colormap, or string value (for example, "r" or "red").

If passed a structure *fv* contain the fields "vertices", "faces" and optionally "facevertexcdata", create the patch based on these properties.

`fill (`*x*, *y*, *c*`)` [Function File]
`fill (`*x1*, *y1*, *c1*, *x2*, *y2*, *c2*`)` [Function File]
`fill (`..., *prop*, *val*`)` [Function File]
`fill (`*h*, ...`)` [Function File]
h `= fill (`...`)` [Function File]

Create one or more filled patch objects, returning a patch object for each.

`surface (`*x*, *y*, *z*, *c*`)` [Function File]
`surface (`*x*, *y*, *z*`)` [Function File]
`surface (`*z*, *c*`)` [Function File]
`surface (`*z*`)` [Function File]
`surface (`..., *prop*, *val*`)` [Function File]
`surface (`*h*, ...`)` [Function File]
h `= surface (`...`)` [Function File]

Plot a surface graphic object given matrices *x*, and *y* from `meshgrid` and a matrix *z* corresponding to the *x* and *y* coordinates of the surface. If *x* and *y* are vectors, then a typical vertex is (*x*(j), *y*(i), *z*(i,j)). Thus, columns of *z* correspond to different *x* values and rows of *z* correspond to different *y* values. If *x* and *y* are missing, they are constructed from size of the matrix *z*.

Any additional properties passed are assigned to the surface.

See also: [surf], page 228, [mesh], page 227, [patch], page 246, [line], page 245.

By default, Octave refreshes the plot window when a prompt is printed, or when waiting for input. To force an update at other times, call the `drawnow` function.

`drawnow ()` [Built-in Function]
`drawnow ("`*expose*`")` [Built-in Function]
`drawnow (`*term*, *file*, *mono*, *debug_file*`)` [Built-in Function]

Update figure windows and their children. The event queue is flushed and any callbacks generated are executed. With the optional argument `"expose"`, only graphic objects are updated and no other events or callbacks are processed. The third calling form of `drawnow` is for debugging and is undocumented.

Only figures that are modified will be updated. The `refresh` function can also be used to force an update of the current figure, even if it is not modified.

`refresh ()` [Function File]
`refresh (h)` [Function File]

Refresh a figure, forcing it to be redrawn. Called without an argument the current figure is redrawn, otherwise the figure pointed to by *h* is redrawn.

See also: [drawnow], page 246.

Normally, high-level plot functions like `plot` or `mesh` call `newplot` to initialize the state of the current axes so that the next plot is drawn in a blank window with default property settings. To have two plots superimposed over one another, use the `hold` function. For example,

```
hold on;
x = -10:0.1:10;
plot (x, sin (x));
plot (x, cos (x));
hold off;
```

displays sine and cosine waves on the same axes. If the hold state is off, consecutive plotting commands like this will only display the last plot.

`newplot ()` [Function File]

Prepare graphics engine to produce a new plot. This function should be called at the beginning of all high-level plotting functions.

`hold` [Function File]
`hold state` [Function File]
`hold (hax, ...)` [Function File]

Toggle or set the 'hold' state of the plotting engine which determines whether new graphic objects are added to the plot or replace the existing objects.

`hold on` Retain plot data and settings so that subsequent plot commands are displayed on a single graph.

`hold off` Clear plot and restore default graphics settings before each new plot command. (default).

`hold` Toggle the current 'hold' state.

When given the additional argument *hax*, the hold state is modified only for the given axis handle.

To query the current 'hold' state use the `ishold` function.

See also: [ishold], page 247, [cla], page 248, [newplot], page 247, [clf], page 248.

`ishold` [Function File]

Return true if the next line will be added to the current plot, or false if the plot device will be cleared before drawing the next line.

To clear the current figure, call the `clf` function. To clear the current axis, call the `cla` function. To bring the current figure to the top of the window stack, call the `shg` function. To delete a graphics object, call `delete` on its index. To close the figure window, call the `close` function.

`clf` () [Function File]
`clf` ("*reset*") [Function File]
`clf` (*hfig*) [Function File]
`clf` (*hfig*, "*reset*") [Function File]

Clear the current figure window. `clf` operates by deleting child graphics objects with visible handles (`HandleVisibility` = on). If *hfig* is specified operate on it instead of the current figure. If the optional argument `"reset"` is specified, all objects including those with hidden handles are deleted.

See also: [cla], page 248, [close], page 248, [delete], page 248.

`cla` () [Function File]
`cla` ("*reset*") [Function File]
`cla` (*hax*) [Function File]
`cla` (*hax*, "*reset*") [Function File]

Delete the children of the current axes with visible handles. If *hax* is specified and is an axes object handle, operate on it instead of the current axes. If the optional argument `"reset"` is specified, also delete the children with hidden handles.

See also: [clf], page 248.

`shg` [Function File]

Show the graph window. Currently, this is the same as executing `drawnow`.

See also: [drawnow], page 246, [figure], page 239.

`delete` (*file*) [Function File]
`delete` (*handle*) [Function File]

Delete the named file or graphics handle.

Deleting graphics objects is the proper way to remove features from a plot without clearing the entire figure.

See also: [clf], page 248, [cla], page 248.

`close` [Command]
`close` (*n*) [Command]
`close` *all* [Command]
`close` *all hidden* [Command]

Close figure window(s) by calling the function specified by the `"closerequestfcn"` property for each figure. By default, the function `closereq` is used.

See also: [closereq], page 248.

`closereq` () [Function File]

Close the current figure and delete all graphics objects associated with it.

See also: [close], page 248, [delete], page 248.

15.2.2 Graphics Object Properties

15.2.2.1 Root Figure Properties

`currentfigure`
: Index to graphics object for the current figure.

15.2.2.2 Figure Properties

`nextplot` May be one of

`"new"`

`"add"`

`"replace"`
`"replacechildren"`

`closerequestfcn`
: Handle of function to call when a figure is closed.

`currentaxes`
: Index to graphics object of current axes.

`colormap` An N-by-3 matrix containing the color map for the current axes.

`visible` Either `"on"` or `"off"` to toggle display of the figure.

`paperorientation`
: Indicates the orientation for printing. Either `"landscape"` or `"portrait"`.

15.2.2.3 Axes Properties

`position` A vector specifying the position of the plot, excluding titles, axes and legend. The four elements of the vector are the coordinates of the lower left corner and width and height of the plot, in units normalized to the width and height of the plot window. For example, `[0.2, 0.3, 0.4, 0.5]` sets the lower left corner of the axes at $(0.2, 0.3)$ and the width and height to be 0.4 and 0.5 respectively. See also the `outerposition` property.

`title` Index of text object for the axes title.

`box` Either `"on"` or `"off"` to toggle display of the box around the axes.

`key` Either `"on"` or `"off"` to toggle display of the legend. Note that this property is not compatible with MATLAB and may be removed in a future version of Octave.

`keybox` Either `"on"` or `"off"` to toggle display of a box around the legend. Note that this property is not compatible with MATLAB and may be removed in a future version of Octave.

`keypos` An integer from 1 to 4 specifying the position of the legend. 1 indicates upper right corner, 2 indicates upper left, 3 indicates lower left, and 4 indicates lower right. Note that this property is not compatible with MATLAB and may be removed in a future version of Octave.

`dataaspectratio`
: A two-element vector specifying the relative height and width of the data displayed in the axes. Setting `dataaspectratio` to '`1, 2]`' causes the length of one unit as displayed on the y-axis to be the same as the length of 2 units on the x-axis. Setting `dataaspectratio` also forces the `dataaspectratiomode` property to be set to `"manual"`.

`dataaspectratiomode`
: Either `"manual"` or `"auto"`.

`xlim`
`ylim`
`zlim`
`clim`
: Two-element vectors defining the limits for the x, y, and z axes and the Setting one of these properties also forces the corresponding mode property to be set to `"manual"`.

`xlimmode`
`ylimmode`
`zlimmode`
`climmode`
: Either `"manual"` or `"auto"`.

`xlabel`
`ylabel`
`zlabel`
: Indices to text objects for the axes labels.

`xgrid`
`ygrid`
`zgrid`
: Either `"on"` or `"off"` to toggle display of grid lines.

`xminorgrid`
`yminorgrid`
`zminorgrid`
: Either `"on"` or `"off"` to toggle display of minor grid lines.

`xtick`
`ytick`
`ztick`
: Setting one of these properties also forces the corresponding mode property to be set to `"manual"`.

`xtickmode`
`ytickmode`
`ztickmode`
: Either `"manual"` or `"auto"`.

`xticklabel`
`yticklabel`
`zticklabel`
: Setting one of these properties also forces the corresponding mode property to be set to `"manual"`.

`xticklabelmode`
`yticklabelmode`
`zticklabelmode`
Either `"manual"` or `"auto"`.

`xscale`
`yscale`
`zscale` Either `"linear"` or `"log"`.

`xdir`
`ydir`
`zdir` Either `"forward"` or `"reverse"`.

`xaxislocation`
`yaxislocation`
Either `"top"` or `"bottom"` for the x-axis and `"left"` or `"right"` for the y-axis.

`view` A three element vector specifying the view point for three-dimensional plots.

`visible` Either `"on"` or `"off"` to toggle display of the axes.

`nextplot` May be one of

`"new"`

`"add"`

`"replace"`
`"replacechildren"`

`outerposition`
A vector specifying the position of the plot, including titles, axes and legend. The four elements of the vector are the coordinates of the lower left corner and width and height of the plot, in units normalized to the width and height of the plot window. For example, `[0.2, 0.3, 0.4, 0.5]` sets the lower left corner of the axes at $(0.2, 0.3)$ and the width and height to be 0.4 and 0.5 respectively. See also the `position` property.

15.2.2.4 Line Properties

`xdata`
`ydata`
`zdata`
`ldata`
`udata`
`xldata`
`xudata` The data to be plotted. The `ldata` and `udata` elements are for errorbars in the y direction, and the `xldata` and `xudata` elements are for errorbars in the x direction.

`color` The RGB color of the line, or a color name. See Section 15.2.4 [Colors], page 258.

`linestyle`
`linewidth`
See Section 15.2.5 [Line Styles], page 258.

`marker`

`markeredgecolor`
`markerfacecolor`
`markersize`
: See Section 15.2.6 [Marker Styles], page 258.

`keylabel` The text of the legend entry corresponding to this line. Note that this property is not compatible with MATLAB and may be removed in a future version of Octave.

15.2.2.5 Text Properties

`string` The character string contained by the text object.

`units` May be `"normalized"` or `"graph"`.

`position` The coordinates of the text object.

`rotation` The angle of rotation for the displayed text, measured in degrees.

`horizontalalignment`
: May be `"left"`, `"center"`, or `"right"`.

`color` The color of the text. See Section 15.2.4 [Colors], page 258.

`fontname` The font used for the text.

`fontsize` The size of the font, in points to use.

`fontangle`
: Flag whether the font is italic or normal. Valid values are 'normal', 'italic' and 'oblique'.

`fontweight`
: Flag whether the font is bold, etc. Valid values are 'normal', 'bold', 'demi' or 'light'.

`interpreter`
: Determines how the text is rendered. Valid values are 'none', 'tex' or 'latex'.

All text objects, including titles, labels, legends, and text, include the property 'interpreter', this property determines the manner in which special control sequences in the text are rendered. If the interpreter is set to 'none', then no rendering occurs. At this point the 'latex' option is not implemented and so the 'latex' interpreter also does not interpret the text.

The 'tex' option implements a subset of TeX functionality in the rendering of the text. This allows the insertion of special characters such as Greek or mathematical symbols within the text. The special characters are also inserted with a code starting with the back-slash (\) character, as in the table Table 15.1.

In addition, the formatting of the text can be changed within the string with the codes

\bf	Bold font
\it	Italic font
\sl	Oblique Font

\rm	Normal font

These are be used in conjunction with the { and } characters to limit the change in the font to part of the string. For example

```
xlabel ('{\bf H} = a {\bf V}')
```

where the character 'a' will not appear in a bold font. Note that to avoid having Octave interpret the backslash characters in the strings, the strings should be in single quotes.

It is also possible to change the fontname and size within the text

\fontname{*fontname*}	Specify the font to use
\fontsize{*size*}	Specify the size of the font to use

Finally, the superscript and subscripting can be controlled with the '^' and '_' characters. If the '^' or '_' is followed by a { character, then all of the block surrounded by the { } pair is super- or sub-scripted. Without the { } pair, only the character immediately following the '^' or '_' is super- or sub-scripted.

Code	Sym	Code	Sym	Code	Sym
\forall	$\forall$	\exists	$\exists$	\ni	$\ni$
\cong	$\cong$	\Delta	Δ	\Phi	Φ
\Gamma	Γ	\vartheta	ϑ	\Lambda	Λ
\Pi	Π	\Theta	Θ	\Sigma	Σ
\varsigma	ς	\Omega	Ω	\Xi	Ξ
\Psi	Ψ	\perp	$\perp$	\alpha	α
\beta	β	\chi	χ	\delta	δ
\epsilon	ϵ	\phi	ϕ	\gamma	γ
\eta	η	\iota	ι	\varphi	φ
\kappa	κ	\lambda	λ	\mu	μ
\nu	ν	\o	$\leq$	\pi	π
\theta	θ	\rho	ρ	\sigma	σ
\tau	τ	\upsilon	υ	\varpi	ϖ
\omega	ω	\xi	ξ	\psi	ψ
\zeta	ζ	\sim	$\sim$	\Upsilon	Υ
\prime	$\prime$	\leq	$\leq$	\infty	∞
\clubsuit	$\clubsuit$	\diamondsuit	$\diamondsuit$	\heartsuit	$\heartsuit$
\spadesuit	$\spadesuit$	\leftrightarrow	$\leftrightarrow$	\leftarrow	$\leftarrow$
\uparrow	$\uparrow$	\rightarrow	$\rightarrow$	\downarrow	$\downarrow$
\circ	$\circ$	\pm	$\pm$	\geq	$\geq$
\times	$\times$	\propto	$\propto$	\partial	∂
\bullet	$\bullet$	\div	$\div$	\neq	$\neq$
\equiv	$\equiv$	\approx	$\approx$	\ldots	$\ldots$
\mid	$\mid$	\aleph	$\aleph$	\Im	$\Im$
\Re	$\Re$	\wp	$\wp$	\otimes	$\otimes$
\oplus	$\oplus$	\oslash	$\oslash$	\cap	$\cap$
\cup	$\cup$	\supset	$\supset$	\supseteq	$\supseteq$
\subset	$\subset$	\subseteq	$\subseteq$	\in	$\in$
\notin	$\notin$	\angle	$\angle$	\bigtriangledown	$\bigtriangledown$
\langle	$\langle$	\rangle	$\rangle$	\nabla	∇
\prod	$\prod$	\surd	$\surd$	\cdot	$\cdot$
\neg	$\neg$	\wedge	$\wedge$	\vee	$\vee$
\Leftrightarrow	$\Leftrightarrow$	\Leftarrow	$\Leftarrow$	\Uparrow	$\Uparrow$
\Rightarrow	$\Rightarrow$	\Downarrow	$\Downarrow$	\diamond	$\diamond$
\copyright	©	\rfloor	$\rfloor$	\lceil	$\lceil$
\lfloor	$\lfloor$	\rceil	$\rceil$	\int	$\int$

Table 15.1: Available special characters in TeX mode

A complete example showing the capabilities of the extended text is

```
x = 0:0.01:3;
plot(x,erf(x));
hold on;
plot(x,x,"r");
axis([0, 3, 0, 1]);
text(0.65, 0.6175, strcat('\leftarrow x = {2/\surd\pi',
' {\fontsize{16}\int_{\fontsize{8}0}^{\fontsize{8}x}}',
' e^{-t^2} dt} = 0.6175'))
```

The result of which can be seen in Figure 15.7

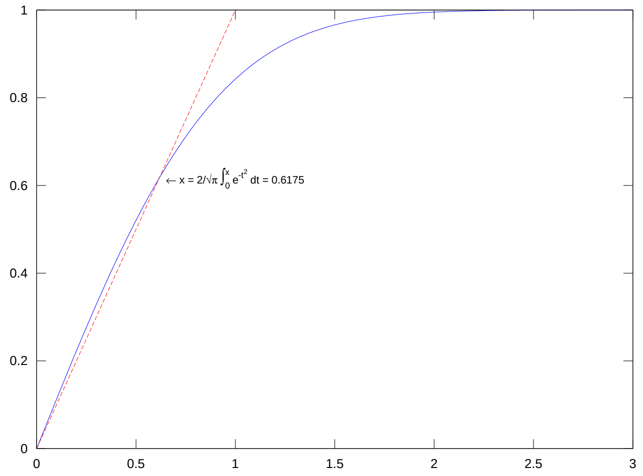

Figure 15.7: Example of inclusion of text with the TeX interpreter

15.2.2.6 Image Properties

`cdata` The data for the image. Each pixel of the image corresponds to an element of `cdata`. The value of an element of `cdata` specifies the row-index into the colormap of the axes object containing the image. The color value found in the color map for the given index determines the color of the pixel.

`xdata`
`ydata` Two-element vectors specifying the range of the x- and y- coordinates for the image.

15.2.2.7 Patch Properties

`cdata`
`xdata`
`ydata`
`zdata` Data defining the patch object.

`facecolor`
The fill color of the patch. See Section 15.2.4 [Colors], page 258.

`facealpha`
: A number in the range [0, 1] indicating the transparency of the patch.

`edgecolor`
: The color of the line defining the patch. See Section 15.2.4 [Colors], page 258.

`linestyle`
`linewidth`
: See Section 15.2.5 [Line Styles], page 258.

`marker`
`markeredgecolor`
`markerfacecolor`
`markersize`
: See Section 15.2.6 [Marker Styles], page 258.

15.2.2.8 Surface Properties

`xdata`
`ydata`
`zdata` The data determining the surface. The `xdata` and `ydata` elements are vectors and `zdata` must be a matrix.

`keylabel` The text of the legend entry corresponding to this surface. Note that this property is not compatible with MATLAB and may be removed in a future version of Octave.

15.2.2.9 Searching Properties

h = **findobj** () [Function File]
h = **findobj** (*prop_name*, *prop_value*) [Function File]
h = **findobj** ('-property', *prop_name*) [Function File]
h = **findobj** ('-regexp', *prop_name*, *pattern*) [Function File]
h = **findobj** ('flat', . . .) [Function File]
h = **findobj** (*h*, . . .) [Function File]
h = **findobj** (*h*, '-depth', *d*, . . .) [Function File]

Find object with specified property values. The simplest form is

```
findobj (prop_name, prop_Value)
```

which returns all of the handles to the objects with the name *prop_name* and the name *prop_Value*. The search can be limited to a particular object or set of objects and their descendants by passing a handle or set of handles *h* as the first argument to `findobj`.

The depth of hierarchy of objects to which to search to can be limited with the '-depth' argument. To limit the number depth of the hierarchy to search to *d* generations of children, and example is

```
findobj (h, '-depth', d, prop_Name, prop_Value)
```

Specifying a depth *d* of 0, limits the search to the set of object passed in *h*. A depth *d* of 0 is equivalent to the '-flat' argument.

A specified logical operator may be applied to the pairs of *prop_Name* and *prop_Value*. The supported logical operators are '-and', '-or', '-xor', '-not'.

The objects may also be matched by comparing a regular expression to the property values, where property values that match `regexp (prop_Value, pattern)` are returned. Finally, objects may be matched by property name only, using the '-property' option.

See also: [get], page 245, [set], page 245.

h = `findall` () [Function File]
h = `findall` (*prop_name*, *prop_value*) [Function File]
h = `findall` (*h*, ...) [Function File]
h = `findall` (*h*, "*-depth*", *d*, ...) [Function File]

Find object with specified property values including hidden handles.

This function performs the same function as `findobj`, but it includes hidden objects in its search. For full documentation, see `findobj`.

See also: [get], page 245, [set], page 245, [findobj], page 256, [allchild], page 245.

15.2.3 Managing Default Properties

Object properties have two classes of default values, *factory defaults* (the initial values) and *user-defined defaults*, which may override the factory defaults.

Although default values may be set for any object, they are set in parent objects and apply to child objects. For example,

```
set (0, "defaultlinecolor", "green");
```

sets the default line color for all objects. The rule for constructing the property name to set a default value is

```
default + object-type + property-name
```

This rule can lead to some strange looking names, for example `defaultlinelinewidth"` specifies the default `linewidth` property for `line` objects.

The example above used the root figure object, 0, so the default property value will apply to all line objects. However, default values are hierarchical, so defaults set in a figure objects override those set in the root figure object. Likewise, defaults set in axes objects override those set in figure or root figure objects. For example,

```
subplot (2, 1, 1);
set (0, "defaultlinecolor", "red");
set (1, "defaultlinecolor", "green");
set (gca (), "defaultlinecolor", "blue");
line (1:10, rand (1, 10));
subplot (2, 1, 2);
line (1:10, rand (1, 10));
figure (2)
line (1:10, rand (1, 10));
```

produces two figures. The line in first subplot window of the first figure is blue because it inherits its color from its parent axes object. The line in the second subplot window of the first figure is green because it inherits its color from its parent figure object. The line in the

second figure window is red because it inherits its color from the global root figure parent object.

To remove a user-defined default setting, set the default property to the value `"remove"`. For example,

```
set (gca (), "defaultlinecolor", "remove");
```

removes the user-defined default line color setting from the current axes object.

Getting the `"default"` property of an object returns a list of user-defined defaults set for the object. For example,

```
get (gca (), "default");
```

returns a list of user-defined default values for the current axes object.

Factory default values are stored in the root figure object. The command

```
get (0, "factory");
```

returns a list of factory defaults.

15.2.4 Colors

Colors may be specified as RGB triplets with values ranging from zero to one, or by name. Recognized color names include `"blue"`, `"black"`, `"cyan"`, `"green"`, `"magenta"`, `"red"`, `"white"`, and `"yellow"`.

15.2.5 Line Styles

Line styles are specified by the following properties:

`linestyle`
: May be one of

  `"-"` Solid lines.

  `"--"` Dashed lines.

  `":"` Points.

  `"-."` A dash-dot line.

`linewidth`
: A number specifying the width of the line. The default is 1. A value of 2 is twice as wide as the default, etc.

15.2.6 Marker Styles

Marker styles are specified by the following properties:

`marker` A character indicating a plot marker to be place at each data point, or `"none"`, meaning no markers should be displayed.

`markeredgecolor`
The color of the edge around the marker, or `"auto"`, meaning that the edge color is the same as the face color. See Section 15.2.4 [Colors], page 258.

`markerfacecolor`
The color of the marker, or `"none"` to indicate that the marker should not be filled. See Section 15.2.4 [Colors], page 258.

`markersize`
A number specifying the size of the marker. The default is 1. A value of 2 is twice as large as the default, etc.

15.2.7 Callbacks

Callback functions can be associated with graphics objects and triggered after certain events occur. The basic structure of all callback function is

```
function mycallback (src, data)
...
endfunction
```

where `src` gives a handle to the source of the callback, and `code` gives some event specific data. This can then be associated with an object either at the objects creation or later with the `set` function. For example

```
plot (x, "DeleteFcn", @(s, e) disp("Window Deleted"))
```

where at the moment that the plot is deleted, the message "Window Deleted" will be displayed.

Additional user arguments can be passed to callback functions, and will be passed after the 2 default arguments. For example

```
plot (x, "DeleteFcn", {@mycallback, "1"})
...
function mycallback (src, data, a1)
  fprintf ("Closing plot %d\n", a1);
endfunction
```

The basic callback functions that are available for all graphics objects are

- CreateFcn This is the callback that is called at the moment of the objects creation. It is not called if the object is altered in any way, and so it only makes sense to define this callback in the function call that defines the object. Callbacks that are added to `CreateFcn` later with the `set` function will never be executed.
- DeleteFcn This is the callback that is called at the moment an object is deleted.
- ButtonDownFcn This is the callback that is called if a mouse button is pressed while the pointer is over this object. Note, that the gnuplot interface does not respect this callback.

The object and figure that the event occurred in that resulted in the callback being called can be found with the `gcbo` and `gcbf` functions.

h = gcbo () [Function File]
[*h*, *fig*] = gcbo () [Function File]

Return a handle to the object whose callback is currently executing. If no callback is executing, this function returns the empty matrix. This handle is obtained from the root object property "CallbackObject".

Additionally return the handle of the figure containing the object whose callback is currently executing. If no callback is executing, the second output is also set to the empty matrix.

See also: [gcf], page 244, [gca], page 244, [gcbf], page 260.

fig = gcbf () [Function File]

Return a handle to the figure containing the object whose callback is currently executing. If no callback is executing, this function returns the empty matrix. The handle returned by this function is the same as the second output argument of gcbo.

See also: [gcf], page 244, [gca], page 244, [gcbo], page 259.

Callbacks can equally be added to properties with the `addlistener` function described below.

15.2.8 Object Groups

A number of Octave high level plot functions return groups of other graphics objects or they return graphics objects that are have their properties linked in such a way that changes to one of the properties results in changes in the others. A graphic object that groups other objects is an `hggroup`

hggroup () [Function File]
hggroup (*h*) [Function File]
hggroup (. . ., *property*, *value*, . . .) [Function File]

Create group object with parent *h*. If no parent is specified, the group is created in the current axes. Return the handle of the group object created.

Multiple property-value pairs may be specified for the group, but they must appear in pairs.

For example a simple use of a `hggroup` might be

```
x = 0:0.1:10;
hg = hggroup ();
plot (x, sin (x), "color", [1, 0, 0], "parent", hg);
hold on
plot (x, cos (x), "color", [0, 1, 0], "parent", hg);
set (hg, "visible", "off");
```

which groups the two plots into a single object and controls their visibility directly. The default properties of an `hggroup` are the same as the set of common properties for the other graphics objects. Additional properties can be added with the `addproperty` function.

addproperty (*name*, *h*, *type*, [*arg*, . . .]) [Built-in Function]

Create a new property named *name* in graphics object *h*. *type* determines the type of the property to create. *args* usually contains the default value of the property, but additional arguments might be given, depending on the type of the property.

The supported property types are:

`string` A string property. *arg* contains the default string value.

`any` An un-typed property. This kind of property can hold any octave value. *args* contains the default value.

`radio` A string property with a limited set of accepted values. The first argument must be a string with all accepted values separated by a vertical bar ('|'). The default value can be marked by enclosing it with a '{' '}' pair. The default value may also be given as an optional second string argument.

`boolean` A boolean property. This property type is equivalent to a radio property with "on|off" as accepted values. *arg* contains the default property value.

`double` A scalar double property. *arg* contains the default value.

`handle` A handle property. This kind of property holds the handle of a graphics object. *arg* contains the default handle value. When no default value is given, the property is initialized to the empty matrix.

`data` A data (matrix) property. *arg* contains the default data value. When no default value is given, the data is initialized to the empty matrix.

`color` A color property. *arg* contains the default color value. When no default color is given, the property is set to black. An optional second string argument may be given to specify an additional set of accepted string values (like a radio property).

type may also be the concatenation of a core object type and a valid property name for that object type. The property created then has the same characteristics as the referenced property (type, possible values, hidden state...). This allows to clone an existing property into the graphics object *h*.

Examples:

```
addproperty ("my_property", gcf, "string", "a string value");
addproperty ("my_radio", gcf, "radio", "val_1|val_2|{val_3}");
addproperty ("my_style", gcf, "linelinestyle", "--");
```

Once a property in added to an `hggroup`, it is not linked to any other property of either the children of the group, or any other graphics object. Add so to control the way in which this newly added property is used, the `addlistener` function is used to define a callback function that is executed when the property is altered.

`addlistener (h, prop, fcn)` [Built-in Function]

Register *fcn* as listener for the property *prop* of the graphics object *h*. Property listeners are executed (in order of registration) when the property is set. The new value is already available when the listeners are executed.

prop must be a string naming a valid property in *h*.

fcn can be a function handle, a string or a cell array whose first element is a function handle. If *fcn* is a function handle, the corresponding function should accept at least 2 arguments, that will be set to the object handle and the empty matrix respectively.

If *fcn* is a string, it must be any valid octave expression. If *fcn* is a cell array, the first element must be a function handle with the same signature as described above. The next elements of the cell array are passed as additional arguments to the function.

Example:

```
function my_listener (h, dummy, p1)
  fprintf ("my_listener called with p1=%s\n", p1);
endfunction

addlistener (gcf, "position", {@my_listener, "my string"})
```

`dellistener` (*h*, *prop*, *fcn*) [Built-in Function]

Remove the registration of *fcn* as a listener for the property *prop* of the graphics object *h*. The function *fcn* must be the same variable (not just the same value), as was passed to the original call to `addlistener`.

If *fcn* is not defined then all listener functions of *prop* are removed.

Example:

```
function my_listener (h, dummy, p1)
  fprintf ("my_listener called with p1=%s\n", p1);
endfunction

c = {@my_listener, "my string"};
addlistener (gcf, "position", c);
dellistener (gcf, "position", c);
```

An example of the use of these two functions might be

```
x = 0:0.1:10;
hg = hggroup ();
h = plot (x, sin (x), "color", [1, 0, 0], "parent", hg);
addproperty ("linestyle", hg, "linelinestyle", get (h, "linestyle"));
addlistener (hg, "linestyle", @update_props);
hold on
plot (x, cos (x), "color", [0, 1, 0], "parent", hg);

function update_props (h, d)
  set (get (h, "children"), "linestyle", get (h, "linestyle"));
endfunction
```

that adds a `linestyle` property to the `hggroup` and propagating any changes its value to the children of the group. The `linkprop` function can be used to simplify the above to be

```
x = 0:0.1:10;
hg = hggroup ();
h1 = plot (x, sin (x), "color", [1, 0, 0], "parent", hg);
addproperty ("linestyle", hg, "linelinestyle", get (h, "linestyle"));
hold on
h2 = plot (x, cos (x), "color", [0, 1, 0], "parent", hg);
hlink = linkprop ([hg, h1, h2], "color");
```

hlink = linkprop (*h*, *prop*) [Function File]

Links graphics object properties, such that a change in one is propagated to the others. The properties to link are given as a string of cell string array by *prop* and the objects containing these properties by the handle array *h*.

An example of the use of linkprops is

```
x = 0:0.1:10;
subplot (1, 2, 1);
h1 = plot (x, sin (x));
subplot (1, 2, 2);
h2 = plot (x, cos (x));
hlink = linkprop ([h1, h2], {"color","linestyle"});
set (h1, "color", "green");
set (h2, "linestyle", "--");
```

These capabilities are used in a number of basic graphics objects. The `hggroup` objects created by the functions of Octave contain one or more graphics object and are used to:

- group together multiple graphics objects,
- create linked properties between different graphics objects, and
- to hide the nominal user data, from the actual data of the objects.

For example the `stem` function creates a stem series where each `hggroup` of the stem series contains two line objects representing the body and head of the stem. The `ydata` property of the `hggroup` of the stem series represents the head of the stem, whereas the body of the stem is between the baseline and this value. For example

```
h = stem (1:4)
get (h, "xdata")
⇒ [  1   2   3   4]'
get (get (h, "children")(1), "xdata")
⇒ [  1   1 NaN   2   2 NaN   3   3 NaN   4   4 NaN]'
```

shows the difference between the `xdata` of the `hggroup` of a stem series object and the underlying line.

The basic properties of such group objects is that they consist of one or more linked `hggroup`, and that changes in certain properties of these groups are propagated to other members of the group. Whereas, certain properties of the members of the group only apply to the current member.

In addition the members of the group can also be linked to other graphics objects through callback functions. For example the baseline of the `bar` or `stem` functions is a line object, whose length and position are automatically adjusted, based on changes to the corresponding hggroup elements.

15.2.8.1 Data sources in object groups

All of the group objects contain data source parameters. There are string parameters that contain an expression that is evaluated to update the relevant data property of the group when the `refreshdata` function is called.

refreshdata () [Function File]
refreshdata (*h*) [Function File]
refreshdata (*h*, *workspace*) [Function File]

Evaluate any '`datasource`' properties of the current figure and update the plot if the corresponding data has changed. If called with one or more arguments *h* is a scalar or array of figure handles to refresh. The optional second argument *workspace* can take the following values.

`"base"` Evaluate the datasource properties in the base workspace. (default).

`"caller"` Evaluate the datasource properties in the workspace of the function that called `refreshdata`.

An example of the use of `refreshdata` is:

```
x = 0:0.1:10;
y = sin (x);
plot (x, y, "ydatasource", "y");
for i = 1 : 100
  pause(0.1)
  y = sin (x + 0.1 * i);
  refreshdata();
endfor
```

15.2.8.2 Area series

Area series objects are created by the `area` function. Each of the `hggroup` elements contains a single patch object. The properties of the area series are

`basevalue`
: The value where the base of the area plot is drawn.

`linewidth`
`linestyle`
: The line width and style of the edge of the patch objects making up the areas. See Section 15.2.5 [Line Styles], page 258.

`edgecolor`
`facecolor`
: The line and fill color of the patch objects making up the areas. See Section 15.2.4 [Colors], page 258.

`xdata`
`ydata`
: The x and y coordinates of the original columns of the data passed to `area` prior to the cumulative summation used in the `area` function.

`xdatasource`
`ydatasource`
: Data source variables.

15.2.8.3 Bar series

Bar series objects are created by the `bar` or `barh` functions. Each `hggroup` element contains a single patch object. The properties of the bar series are

`showbaseline`
`baseline`
`basevalue`
: The property `showbaseline` flags whether the baseline of the bar series is displayed (default is "on"). The handle of the graphics object representing the baseline is given by the `baseline` property and the y-value of the baseline by the `basevalue` property.

  Changes to any of these property are propagated to the other members of the bar series and to the baseline itself. Equally changes in the properties of the base line itself are propagated to the members of the corresponding bar series.

`barwidth`
`barlayout`
`horizontal`
: The property `barwidth` is the width of the bar corresponding to the *width* variable passed to `bar` or *barh*. Whether the bar series is "grouped" or "stacked" is determined by the `barlayout` property and whether the bars are horizontal or vertical by the `horizontal` property.

  Changes to any of these property are propagated to the other members of the bar series.

`linewidth`
`linestyle`
: The line width and style of the edge of the patch objects making up the bars. See Section 15.2.5 [Line Styles], page 258.

`edgecolor`
`facecolor`
: The line and fill color of the patch objects making up the bars. See Section 15.2.4 [Colors], page 258.

`xdata` : The nominal x positions of the bars. Changes in this property and propagated to the other members of the bar series.

`ydata` : The y value of the bars in the `hggroup`.

`xdatasource`
`ydatasource`
: Data source variables.

15.2.8.4 Contour groups

Contour group objects are created by the `contour`, `contourf` and `contour3` functions. The are equally one of the handles returned by the `surfc` and `meshc` functions. The properties of the contour group are

`contourmatrix`
: A read only property that contains the data return by `contourc` used to create the contours of the plot.

`fill` : A radio property that can have the values "on" or "off" that flags whether the contours to plot are to be filled.

`zlevelmode`
`zlevel` The radio property `zlevelmode` can have the values "none", "auto" or "manual". When its value is "none" there is no z component to the plotted contours. When its value is "auto" the z value of the plotted contours is at the same value as the contour itself. If the value is "manual", then the z value at which to plot the contour is determined by the `zlevel` property.

`levellistmode`
`levellist`
`levelstepmode`
`levelstep`
If `levellistmode` is "manual", then the levels at which to plot the contours is determined by `levellist`. If `levellistmode` is set to "auto", then the distance between contours is determined by `levelstep`. If both `levellistmode` and `levelstepmode` are set to "auto", then there are assumed to be 10 equal spaced contours.

`textlistmode`
`textlist`
`textstepmode`
`textstep` If `textlistmode` is "manual", then the labelled contours is determined by `textlist`. If `textlistmode` is set to "auto", then the distance between labelled contours is determined by `textstep`. If both `textlistmode` and `textstepmode` are set to "auto", then there are assumed to be 10 equal spaced labelled contours.

`showtext` Flag whether the contour labels are shown or not.

`labelspacing`
The distance between labels on a single contour in points.

`linewidth`
`linestyle`
`linecolor`
The properties of the contour lines. The properties `linewidth` and `linestyle` are similar to the corresponding properties for lines. The property `linecolor` is a color property (see Section 15.2.4 [Colors], page 258), that can also have the values of "none" or "auto". If `linecolor` is "none", then no contour line is drawn. If `linecolor` is "auto" then the line color is determined by the colormap.

`xdata`
`ydata`
`zdata` The original x, y, and z data of the contour lines.

`xdatasource`
`ydatasource`
`zdatasource`
Data source variables.

15.2.8.5 Error bar series

Error bar series are created by the `errorbar` function. Each `hggroup` element contains two line objects representing the data and the errorbars separately. The properties of the error bar series are

`color` The RGB color or color name of the line objects of the error bars. See Section 15.2.4 [Colors], page 258.

`linewidth`
`linestyle`
The line width and style of the line objects of the error bars. See Section 15.2.5 [Line Styles], page 258.

`marker`
`markeredgecolor`
`markerfacecolor`
`markersize`
The line and fill color of the markers on the error bars. See Section 15.2.4 [Colors], page 258.

`xdata`
`ydata`
`ldata`
`udata`
`xldata`
`xudata` The original x, y, l, u, xl, xu data of the error bars.

`xdatasource`
`ydatasource`
`ldatasource`
`udatasource`
`xldatasource`
`xudatasource`
Data source variables.

15.2.8.6 Line series

Line series objects are created by the `plot` and `plot3` functions and are of the type `line`. The properties of the line series with the ability to add data sources.

`color` The RGB color or color name of the line objects. See Section 15.2.4 [Colors], page 258.

`linewidth`
`linestyle`
The line width and style of the line objects. See Section 15.2.5 [Line Styles], page 258.

`marker`
`markeredgecolor`
`markerfacecolor`
`markersize`
The line and fill color of the markers. See Section 15.2.4 [Colors], page 258.

`xdata`
`ydata`
`zdata` The original x, y and z data.

`xdatasource`
`ydatasource`
`zdatasource`
Data source variables.

15.2.8.7 Quiver group

Quiver series objects are created by the `quiver` or `quiver3` functions. Each `hggroup` element of the series contains three line objects as children representing the body and head of the arrow, together with a marker as the point of original of the arrows. The properties of the quiver series are

`autoscale`
`autoscalefactor`
Flag whether the length of the arrows is scaled or defined directly from the *u*, *v* and *w* data. If the arrow length is flagged as being scaled by the `autoscale` property, then the length of the autoscaled arrow is controlled by the `autoscalefactor`.

`maxheadsize`
This property controls the size of the head of the arrows in the quiver series. The default value is 0.2.

`showarrowhead`
Flag whether the arrow heads are displayed in the quiver plot.

`color` The RGB color or color name of the line objects of the quiver. See Section 15.2.4 [Colors], page 258.

`linewidth`
`linestyle`
The line width and style of the line objects of the quiver. See Section 15.2.5 [Line Styles], page 258.

`marker`
`markerfacecolor`
`markersize`
The line and fill color of the marker objects at the original of the arrows. See Section 15.2.4 [Colors], page 258.

`xdata`
`ydata`
`zdata` The origins of the values of the vector field.

`udata`
`vdata`
`wdata` The values of the vector field to plot.

`xdatasource`
`ydatasource`
`zdatasource`
`udatasource`
`vdatasource`
`wdatasource`
Data source variables.

15.2.8.8 Scatter group

Scatter series objects are created by the `scatter` or `scatter3` functions. A single hggroup element contains as many children as there are points in the scatter plot, with each child representing one of the points. The properties of the stem series are

`linewidth`
The line width of the line objects of the points. See Section 15.2.5 [Line Styles], page 258.

`marker`
`markeredgecolor`
`markerfacecolor`
The line and fill color of the markers of the points. See Section 15.2.4 [Colors], page 258.

`xdata`
`ydata`
`zdata` The original x, y and z data of the stems.

`cdata` The color data for the points of the plot. Each point can have a separate color, or a unique color can be specified.

`sizedata` The size data for the points of the plot. Each point can its own size or a unique size can be specified.

`xdatasource`
`ydatasource`
`zdatasource`
`cdatasource`
`sizedatasource`
Data source variables.

15.2.8.9 Stair group

Stair series objects are created by the `stair` function. Each `hggroup` element of the series contains a single line object as a child representing the stair. The properties of the stair series are

`color` The RGB color or color name of the line objects of the stairs. See Section 15.2.4 [Colors], page 258.

`linewidth`
`linestyle`
The line width and style of the line objects of the stairs. See Section 15.2.5 [Line Styles], page 258.

`marker`
`markeredgecolor`
`markerfacecolor`
`markersize`
: The line and fill color of the markers on the stairs. See Section 15.2.4 [Colors], page 258.

`xdata`
`ydata` The original x and y data of the stairs.

`xdatasource`
`ydatasource`
: Data source variables.

15.2.8.10 Stem Series

Stem series objects are created by the `stem` or `stem3` functions. Each `hggroup` element contains a single line object as a child representing the stems. The properties of the stem series are

`showbaseline`
`baseline`
`basevalue`
: The property `showbaseline` flags whether the baseline of the stem series is displayed (default is `"on"`). The handle of the graphics object representing the baseline is given by the `baseline` property and the y-value (or z-value for `stem3`) of the baseline by the `basevalue` property.

Changes to any of these property are propagated to the other members of the stem series and to the baseline itself. Equally changes in the properties of the base line itself are propagated to the members of the corresponding stem series.

`color` The RGB color or color name of the line objects of the stems. See Section 15.2.4 [Colors], page 258.

`linewidth`
`linestyle`
: The line width and style of the line objects of the stems. See Section 15.2.5 [Line Styles], page 258.

`marker`
`markeredgecolor`
`markerfacecolor`
`markersize`
: The line and fill color of the markers on the stems. See Section 15.2.4 [Colors], page 258.

`xdata`
`ydata`
`zdata` The original x, y and z data of the stems.

`xdatasource`
`ydatasource`
`zdatasource`
Data source variables.

15.2.8.11 Surface group

Surface group objects are created by the `surf` or `mesh` functions, but are equally one of the handles returned by the `surfc` or `meshc` functions. The surface group is of the type `surface`.

The properties of the surface group are

`edgecolor`
`facecolor`
The RGB color or color name of the edges or faces of the surface. See Section 15.2.4 [Colors], page 258.

`linewidth`
`linestyle`
The line width and style of the lines on the surface. See Section 15.2.5 [Line Styles], page 258.

`marker`
`markeredgecolor`
`markerfacecolor`
`markersize`
The line and fill color of the markers on the surface. See Section 15.2.4 [Colors], page 258.

`xdata`
`ydata`
`zdata`

`cdata` The original x, y, z and c data.

`xdatasource`
`ydatasource`
`zdatasource`
`cdatasource`
Data source variables.

15.2.9 Graphics backends

`backend (`*`name`*`)` [Function File]
`backend (`*`hlist`*`, `*`name`*`)` [Function File]

Change the default graphics backend to *name*. If the backend is not already loaded, it is first initialized (initialization is done through the execution of `__init_`*`name`*`__`).

When called with a list of figure handles, *hlist*, the backend is changed only for the listed figures.

See also: [available_backends], page 272.

`available_backends ()` [Built-in Function]

Return a cell array of registered graphics backends.

15.2.9.1 Interaction with gnuplot

16 Matrix Manipulation

There are a number of functions available for checking to see if the elements of a matrix meet some condition, and for rearranging the elements of a matrix. For example, Octave can easily tell you if all the elements of a matrix are finite, or are less than some specified value. Octave can also rotate the elements, extract the upper- or lower-triangular parts, or sort the columns of a matrix.

16.1 Finding Elements and Checking Conditions

The functions `any` and `all` are useful for determining whether any or all of the elements of a matrix satisfy some condition. The `find` function is also useful in determining which elements of a matrix meet a specified condition.

`any` (*x*, *dim*) [Built-in Function]

For a vector argument, return 1 if any element of the vector is nonzero.

For a matrix argument, return a row vector of ones and zeros with each element indicating whether any of the elements of the corresponding column of the matrix are nonzero. For example,

```
any (eye (2, 4))
     ⇒ [ 1, 1, 0, 0 ]
```

If the optional argument *dim* is supplied, work along dimension *dim*. For example,

```
any (eye (2, 4), 2)
     ⇒ [ 1; 1 ]
```

`all` (*x*, *dim*) [Built-in Function]

The function `all` behaves like the function `any`, except that it returns true only if all the elements of a vector, or all the elements along dimension *dim* of a matrix, are nonzero.

Since the comparison operators (see Section 8.4 [Comparison Ops], page 113) return matrices of ones and zeros, it is easy to test a matrix for many things, not just whether the elements are nonzero. For example,

```
all (all (rand (5) < 0.9))
     ⇒ 0
```

tests a random 5 by 5 matrix to see if all of its elements are less than 0.9.

Note that in conditional contexts (like the test clause of `if` and `while` statements) Octave treats the test as if you had typed `all (all (condition))`.

`xor` (*x*, *y*) [Mapping Function]

Return the 'exclusive or' of the entries of *x* and *y*. For boolean expressions *x* and *y*, `xor (x, y)` is true if and only if *x* or *y* is true, but not if both *x* and *y* are true.

`is_duplicate_entry` (*x*) [Function File]

Return non-zero if any entries in *x* are duplicates of one another.

`diff (x, k, dim)` [Function File]

If *x* is a vector of length *n*, `diff (x)` is the vector of first differences $x_2 - x_1, \ldots, x_n - x_{n-1}$.

If *x* is a matrix, `diff (x)` is the matrix of column differences along the first non-singleton dimension.

The second argument is optional. If supplied, `diff (x, k)`, where *k* is a non-negative integer, returns the *k*-th differences. It is possible that *k* is larger than then first non-singleton dimension of the matrix. In this case, `diff` continues to take the differences along the next non-singleton dimension.

The dimension along which to take the difference can be explicitly stated with the optional variable *dim*. In this case the *k*-th order differences are calculated along this dimension. In the case where *k* exceeds `size (x, dim)` then an empty matrix is returned.

`isinf (x)` [Mapping Function]

Return 1 for elements of *x* that are infinite and zero otherwise. For example,

```
isinf ([13, Inf, NA, NaN])
     ⇒ [ 0, 1, 0, 0 ]
```

`isnan (x)` [Mapping Function]

Return 1 for elements of *x* that are NaN values and zero otherwise. NA values are also considered NaN values. For example,

```
isnan ([13, Inf, NA, NaN])
     ⇒ [ 0, 0, 1, 1 ]
```

See also: [isna], page 35.

`finite (x)` [Mapping Function]

Return 1 for elements of *x* that are finite values and zero otherwise. For example,

```
finite ([13, Inf, NA, NaN])
     ⇒ [ 1, 0, 0, 0 ]
```

`find (x)` [Loadable Function]
`find (x, n)` [Loadable Function]
`find (x, n, direction)` [Loadable Function]

Return a vector of indices of nonzero elements of a matrix, as a row if *x* is a row or as a column otherwise. To obtain a single index for each matrix element, Octave pretends that the columns of a matrix form one long vector (like Fortran arrays are stored). For example,

```
find (eye (2))
     ⇒ [ 1; 4 ]
```

If two outputs are requested, `find` returns the row and column indices of nonzero elements of a matrix. For example,

```
[i, j] = find (2 * eye (2))
     ⇒ i = [ 1; 2 ]
     ⇒ j = [ 1; 2 ]
```

If three outputs are requested, `find` also returns a vector containing the nonzero values. For example,

```
[i, j, v] = find (3 * eye (2))
     ⇒ i = [ 1; 2 ]
     ⇒ j = [ 1; 2 ]
     ⇒ v = [ 3; 3 ]
```

If two inputs are given, *n* indicates the maximum number of elements to find from the beginning of the matrix or vector.

If three inputs are given, *direction* should be one of "first" or "last", requesting only the first or last *n* indices, respectively. However, the indices are always returned in ascending order.

Note that this function is particularly useful for sparse matrices, as it extracts the non-zero elements as vectors, which can then be used to create the original matrix. For example,

```
sz = size(a);
[i, j, v] = find (a);
b = sparse(i, j, v, sz(1), sz(2));
```

See also: [sparse], page 348.

`[err, y1, ...] = common_size (x1, ...)` [Function File]

Determine if all input arguments are either scalar or of common size. If so, *err* is zero, and *yi* is a matrix of the common size with all entries equal to *xi* if this is a scalar or *xi* otherwise. If the inputs cannot be brought to a common size, errorcode is 1, and *yi* is *xi*. For example,

```
[errorcode, a, b] = common_size ([1 2; 3 4], 5)
     ⇒ errorcode = 0
     ⇒ a = [ 1, 2; 3, 4 ]
     ⇒ b = [ 5, 5; 5, 5 ]
```

This is useful for implementing functions where arguments can either be scalars or of common size.

16.2 Rearranging Matrices

`fliplr (x)` [Function File]

Return a copy of *x* with the order of the columns reversed. For example,

```
fliplr ([1, 2; 3, 4])
     ⇒  2  1
        4  3
```

Note that `fliplr` only work with 2-D arrays. To flip N-d arrays use `flipdim` instead.

See also: [flipud], page 275, [flipdim], page 276, [rot90], page 276, [rotdim], page 276.

`flipud (x)` [Function File]

Return a copy of *x* with the order of the rows reversed. For example,

```
flipud ([1, 2; 3, 4])
     ⇒  3  4
        1  2
```

Due to the difficulty of defining which axis about which to flip the matrix `flipud` only work with 2-d arrays. To flip N-d arrays use `flipdim` instead.

See also: [fliplr], page 275, [flipdim], page 276, [rot90], page 276, [rotdim], page 276.

`flipdim` (*x*, *dim*) [Function File]

Return a copy of *x* flipped about the dimension *dim*. For example

```
flipdim ([1, 2; 3, 4], 2)
     ⇒  2  1
        4  3
```

See also: [fliplr], page 275, [flipud], page 275, [rot90], page 276, [rotdim], page 276.

`rot90` (*x*, *n*) [Function File]

Return a copy of *x* with the elements rotated counterclockwise in 90-degree increments. The second argument is optional, and specifies how many 90-degree rotations are to be applied (the default value is 1). Negative values of *n* rotate the matrix in a clockwise direction. For example,

```
rot90 ([1, 2; 3, 4], -1)
     ⇒  3  1
        4  2
```

rotates the given matrix clockwise by 90 degrees. The following are all equivalent statements:

```
rot90 ([1, 2; 3, 4], -1)
rot90 ([1, 2; 3, 4], 3)
rot90 ([1, 2; 3, 4], 7)
```

Due to the difficulty of defining an axis about which to rotate the matrix `rot90` only work with 2-D arrays. To rotate N-d arrays use `rotdim` instead.

See also: [rotdim], page 276, [flipud], page 275, [fliplr], page 275, [flipdim], page 276.

`rotdim` (*x*, *n*, *plane*) [Function File]

Return a copy of *x* with the elements rotated counterclockwise in 90-degree increments. The second argument is optional, and specifies how many 90-degree rotations are to be applied (the default value is 1). The third argument is also optional and defines the plane of the rotation. As such *plane* is a two element vector containing two different valid dimensions of the matrix. If *plane* is not given Then the first two non-singleton dimensions are used.

Negative values of *n* rotate the matrix in a clockwise direction. For example,

```
rotdim ([1, 2; 3, 4], -1, [1, 2])
     ⇒  3  1
        4  2
```

rotates the given matrix clockwise by 90 degrees. The following are all equivalent statements:

```
rotdim ([1, 2; 3, 4], -1, [1, 2])
rotdim ([1, 2; 3, 4], 3, [1, 2])
rotdim ([1, 2; 3, 4], 7, [1, 2])
```

See also: [rot90], page 276, [flipud], page 275, [fliplr], page 275, [flipdim], page 276.

cat (*dim*, *array1*, *array2*, ..., *arrayN*) [Built-in Function]

Return the concatenation of N-d array objects, *array1*, *array2*, ..., *arrayN* along dimension *dim*.

```
A = ones (2, 2);
B = zeros (2, 2);
cat (2, A, B)
⇒ ans =

    1 1 0 0
    1 1 0 0
```

Alternatively, we can concatenate *A* and *B* along the second dimension the following way:

```
[A, B].
```

dim can be larger than the dimensions of the N-d array objects and the result will thus have *dim* dimensions as the following example shows:

```
cat (4, ones(2, 2), zeros (2, 2))
⇒ ans =

   ans(:,:,1,1) =

     1 1
     1 1

   ans(:,:,1,2) =
     0 0
     0 0
```

See also: [horzcat], page 277, [vertcat], page 277.

horzcat (*array1*, *array2*, ..., *arrayN*) [Built-in Function]

Return the horizontal concatenation of N-d array objects, *array1*, *array2*, ..., *arrayN* along dimension 2.

See also: [cat], page 277, [vertcat], page 277.

vertcat (*array1*, *array2*, ..., *arrayN*) [Built-in Function]

Return the vertical concatenation of N-d array objects, *array1*, *array2*, ..., *arrayN* along dimension 1.

See also: [cat], page 277, [horzcat], page 277.

permute (*a*, *perm*) [Built-in Function]

Return the generalized transpose for an N-d array object *a*. The permutation vector *perm* must contain the elements `1:ndims(a)` (in any order, but each element must appear just once).

See also: [ipermute], page 277.

ipermute (*a*, *iperm*) [Built-in Function]

The inverse of the `permute` function. The expression

```
ipermute (permute (a, perm), perm)
```

returns the original array *a*.

See also: [permute], page 277.

`reshape` (*a*, *m*, *n*, ...) [Built-in Function]
`reshape` (*a*, *size*) [Built-in Function]

Return a matrix with the given dimensions whose elements are taken from the matrix *a*. The elements of the matrix are accessed in column-major order (like Fortran arrays are stored).

For example,

```
reshape ([1, 2, 3, 4], 2, 2)
     ⇒  1  3
        2  4
```

Note that the total number of elements in the original matrix must match the total number of elements in the new matrix.

A single dimension of the return matrix can be unknown and is flagged by an empty argument.

`resize` (*x*, *m*) [Built-in Function]
`resize` (*x*, *m*, *n*) [Built-in Function]
`resize` (*x*, *m*, *n*, ...) [Built-in Function]

Resize *x* cutting off elements as necessary.

In the result, element with certain indices is equal to the corresponding element of *x* if the indices are within the bounds of *x*; otherwise, the element is set to zero.

In other words, the statement

```
y = resize (x, dv);
```

is equivalent to the following code:

```
y = zeros (dv, class (x));
sz = min (dv, size (x));
for i = 1:length (sz), idx{i} = 1:sz(i); endfor
y(idx{:}) = x(idx{:});
```

but is performed more efficiently.

If only *m* is supplied and it is a scalar, the dimension of the result is *m*-by-*m*. If *m* is a vector, then the dimensions of the result are given by the elements of *m*. If both *m* and *n* are scalars, then the dimensions of the result are *m*-by-*n*.

An object can be resized to more dimensions than it has; in such case the missing dimensions are assumed to be 1. Resizing an object to fewer dimensions is not possible.

See also: [reshape], page 278, [postpad], page 281.

y = `circshift` (*x*, *n*) [Function File]

Circularly shifts the values of the array *x*. *n* must be a vector of integers no longer than the number of dimensions in *x*. The values of *n* can be either positive or negative, which determines the direction in which the values or *x* are shifted. If an element of *n* is zero, then the corresponding dimension of *x* will not be shifted. For example

```
x = [1, 2, 3; 4, 5, 6; 7, 8, 9];
circshift (x, 1)
⇒  7, 8, 9
    1, 2, 3
    4, 5, 6
circshift (x, -2)
⇒  7, 8, 9
    1, 2, 3
    4, 5, 6
circshift (x, [0,1])
⇒  3, 1, 2
    6, 4, 5
    9, 7, 8
```

See also: permute, ipermute, shiftdim.

`y = shiftdim (x, n)` [Function File]
`[y, ns] = shiftdim (x)` [Function File]

Shifts the dimension of *x* by *n*, where *n* must be an integer scalar. When *n* is positive, the dimensions of *x* are shifted to the left, with the leading dimensions circulated to the end. If *n* is negative, then the dimensions of *x* are shifted to the right, with *n* leading singleton dimensions added.

Called with a single argument, `shiftdim`, removes the leading singleton dimensions, returning the number of dimensions removed in the second output argument *ns*.

For example

```
x = ones (1, 2, 3);
size (shiftdim (x, -1))
     ⇒ [1, 1, 2, 3]
size (shiftdim (x, 1))
     ⇒ [2, 3]
[b, ns] = shiftdim (x);
     ⇒ b =  [1, 1, 1; 1, 1, 1]
     ⇒ ns = 1
```

See also: reshape, permute, ipermute, circshift, squeeze.

`shift (x, b)` [Function File]
`shift (x, b, dim)` [Function File]

If *x* is a vector, perform a circular shift of length *b* of the elements of *x*.

If *x* is a matrix, do the same for each column of *x*. If the optional *dim* argument is given, operate along this dimension

`[s, i] = sort (x)` [Loadable Function]
`[s, i] = sort (x, dim)` [Loadable Function]
`[s, i] = sort (x, mode)` [Loadable Function]
`[s, i] = sort (x, dim, mode)` [Loadable Function]

Return a copy of *x* with the elements arranged in increasing order. For matrices, `sort` orders the elements in each column.

For example,

```
sort ([1, 2; 2, 3; 3, 1])
     ⇒  1  1
        2  2
        3  3
```

The `sort` function may also be used to produce a matrix containing the original row indices of the elements in the sorted matrix. For example,

```
[s, i] = sort ([1, 2; 2, 3; 3, 1])
     ⇒ s = 1  1
           2  2
           3  3
     ⇒ i = 1  3
           2  1
           3  2
```

If the optional argument *dim* is given, then the matrix is sorted along the dimension defined by *dim*. The optional argument `mode` defines the order in which the values will be sorted. Valid values of `mode` are 'ascend' or 'descend'.

For equal elements, the indices are such that the equal elements are listed in the order that appeared in the original list.

The `sort` function may also be used to sort strings and cell arrays of strings, in which case the dictionary order of the strings is used.

The algorithm used in `sort` is optimized for the sorting of partially ordered lists.

`sortrows (a, c)` [Function File]

Sort the rows of the matrix *a* according to the order of the columns specified in *c*. If *c* is omitted, a lexicographical sort is used. By default ascending order is used however if elements of *c* are negative then the corresponding column is sorted in descending order.

`issorted (a, rows)` [Built-in Function]

Returns true if the array is sorted, ascending or descending. NaNs are treated as by `sort`. If *rows* is supplied and has the value "rows", checks whether the array is sorted by rows as if output by `sortrows` (with no options).

This function does not yet support sparse matrices.

See also: [sortrows], page 280, [sort], page 279.

Since the `sort` function does not allow sort keys to be specified, it can't be used to order the rows of a matrix according to the values of the elements in various columns[1] in a single call. Using the second output, however, it is possible to sort all rows based on the values in a given column. Here's an example that sorts the rows of a matrix based on the values in the second column.

[1] For example, to first sort based on the values in column 1, and then, for any values that are repeated in column 1, sort based on the values found in column 2, etc.

```
a = [1, 2; 2, 3; 3, 1];
[s, i] = sort (a (:, 2));
a (i, :)
     ⇒ 3  1
       1  2
       2  3
```

tril (*a*, *k*) [Function File]
triu (*a*, *k*) [Function File]

Return a new matrix formed by extracting the lower (`tril`) or upper (`triu`) triangular part of the matrix *a*, and setting all other elements to zero. The second argument is optional, and specifies how many diagonals above or below the main diagonal should also be set to zero.

The default value of *k* is zero, so that `triu` and `tril` normally include the main diagonal as part of the result matrix.

If the value of *k* is negative, additional elements above (for `tril`) or below (for `triu`) the main diagonal are also selected.

The absolute value of *k* must not be greater than the number of sub- or super-diagonals.

For example,

```
tril (ones (3), -1)
     ⇒ 0  0  0
       1  0  0
       1  1  0
```

and

```
tril (ones (3), 1)
     ⇒ 1  1  0
       1  1  1
       1  1  1
```

See also: [triu], page 281, [diag], page 282.

vec (*x*) [Function File]

Return the vector obtained by stacking the columns of the matrix *x* one above the other.

vech (*x*) [Function File]

Return the vector obtained by eliminating all supradiagonal elements of the square matrix *x* and stacking the result one column above the other.

prepad (*x*, *l*, *c*) [Function File]
prepad (*x*, *l*, *c*, *dim*) [Function File]

Prepend (append) the scalar value *c* to the vector *x* until it is of length *l*. If the third argument is not supplied, a value of 0 is used.

If `length (x) > l`, elements from the beginning (end) of *x* are removed until a vector of length *l* is obtained.

If *x* is a matrix, elements are prepended or removed from each row.

If the optional *dim* argument is given, then operate along this dimension.

See also: [postpad], page 281.

`diag (v, k)` [Built-in Function]

Return a diagonal matrix with vector *v* on diagonal *k*. The second argument is optional. If it is positive, the vector is placed on the *k*-th super-diagonal. If it is negative, it is placed on the -*k*-th sub-diagonal. The default value of *k* is 0, and the vector is placed on the main diagonal. For example,

```
diag ([1, 2, 3], 1)
     ⇒  0  1  0  0
         0  0  2  0
         0  0  0  3
         0  0  0  0
```

Given a matrix argument, instead of a vector, `diag` extracts the *k*-th diagonal of the matrix.

`blkdiag (a, b, c, ...)` [Function File]

Build a block diagonal matrix from *a*, *b*, *c*, ... All the arguments must be numeric and are two-dimensional matrices or scalars.

See also: [diag], page 282, [horzcat], page 277, [vertcat], page 277.

16.3 Applying a Function to an Array

`arrayfun (func, a)` [Function File]
`x = arrayfun (func, a)` [Function File]
`x = arrayfun (func, a, b, ...)` [Function File]
`[x, y, ...] = arrayfun (func, a, ...)` [Function File]
`arrayfun (..., "UniformOutput", val)` [Function File]
`arrayfun (..., "ErrorHandler", errfunc)` [Function File]

Execute a function on each element of an array. This is useful for functions that do not accept array arguments. If the function does accept array arguments it is better to call the function directly.

The first input argument *func* can be a string, a function handle, an inline function or an anonymous function. The input argument *a* can be a logic array, a numeric array, a string array, a structure array or a cell array. By a call of the function `arrayfun` all elements of *a* are passed on to the named function *func* individually.

The named function can also take more than two input arguments, with the input arguments given as third input argument *b*, fourth input argument *c*, . . . If given more than one array input argument then all input arguments must have the same sizes, for example

```
arrayfun (@atan2, [1, 0], [0, 1])
⇒ ans = [1.5708   0.0000]
```

If the parameter *val* after a further string input argument "UniformOutput" is set `true` (the default), then the named function *func* must return a single element which then will be concatenated into the return value and is of type matrix. Otherwise, if

that parameter is set to `false`, then the outputs are concatenated in a cell array. For example

```
arrayfun (@(x,y) x:y, "abc", "def", "UniformOutput", false)
⇒ ans =
{
  [1,1] = abcd
  [1,2] = bcde
  [1,3] = cdef
}
```

If more than one output arguments are given then the named function must return the number of return values that also are expected, for example

```
[A, B, C] = arrayfun (@find, [10; 0], "UniformOutput", false)
⇒
A =
{
  [1,1] =  1
  [2,1] = [](0x0)
}
B =
{
  [1,1] =  1
  [2,1] = [](0x0)
}
C =
{
  [1,1] =  10
  [2,1] = [](0x0)
}
```

If the parameter *errfunc* after a further string input argument "ErrorHandler" is another string, a function handle, an inline function or an anonymous function, then *errfunc* defines a function to call in the case that *func* generates an error. The definition of the function must be of the form

```
function [...] = errfunc (s, ...)
```

where there is an additional input argument to *errfunc* relative to *func*, given by *s*. This is a structure with the elements "identifier", "message" and "index", giving respectively the error identifier, the error message and the index of the array elements that caused the error. The size of the output argument of *errfunc* must have the same size as the output argument of *func*, otherwise a real error is thrown. For example

```
function y = ferr (s, x), y = "MyString"; endfunction
arrayfun (@str2num, [1234], \
          "UniformOutput", false, "ErrorHandler", @ferr)
⇒ ans =
{
 [1,1] = MyString
}
```

See also: [cellfun], page 91, [spfun], page 346, [structfun], page 84.

`bsxfun (f, a, b)` [Loadable Function]

Applies a binary function *f* element-wise to two matrix arguments *a* and *b*. The function *f* must be capable of accepting two column vector arguments of equal length, or one column vector argument and a scalar.

The dimensions of *a* and *b* must be equal or singleton. The singleton dimensions of the matrices will be expanded to the same dimensionality as the other matrix.

See also: [arrayfun], page 282, [cellfun], page 91.

16.4 Special Utility Matrices

`eye (x)` [Built-in Function]
`eye (n, m)` [Built-in Function]
`eye (..., class)` [Built-in Function]

Return an identity matrix. If invoked with a single scalar argument, `eye` returns a square matrix with the dimension specified. If you supply two scalar arguments, `eye` takes them to be the number of rows and columns. If given a vector with two elements, `eye` uses the values of the elements as the number of rows and columns, respectively. For example,

```
eye (3)
     ⇒  1  0  0
        0  1  0
        0  0  1
```

The following expressions all produce the same result:

```
eye (2)
≡
eye (2, 2)
≡
eye (size ([1, 2; 3, 4])
```

The optional argument *class*, allows `eye` to return an array of the specified type, like

```
val = zeros (n,m, "uint8")
```

Calling `eye` with no arguments is equivalent to calling it with an argument of 1. This odd definition is for compatibility with MATLAB.

`ones (x)` [Built-in Function]
`ones (n, m)` [Built-in Function]
`ones (n, m, k, ...)` [Built-in Function]
`ones (..., class)` [Built-in Function]

Return a matrix or N-dimensional array whose elements are all 1. The arguments are handled the same as the arguments for `eye`.

If you need to create a matrix whose values are all the same, you should use an expression like

```
val_matrix = val * ones (n, m)
```

The optional argument *class*, allows `ones` to return an array of the specified type, for example

```
val = ones (n,m, "uint8")
```

zeros (*x*) [Built-in Function]
zeros (*n*, *m*) [Built-in Function]
zeros (*n*, *m*, *k*, ...) [Built-in Function]
zeros (..., *class*) [Built-in Function]

Return a matrix or N-dimensional array whose elements are all 0. The arguments are handled the same as the arguments for `eye`.

The optional argument *class*, allows `zeros` to return an array of the specified type, for example

```
val = zeros (n,m, "uint8")
```

repmat (*A*, *m*, *n*) [Function File]
repmat (*A*, [*m n*]) [Function File]
repmat (*A*, [*m n p* ...]) [Function File]

Form a block matrix of size *m* by *n*, with a copy of matrix *A* as each element. If *n* is not specified, form an *m* by *m* block matrix.

rand (*x*) [Loadable Function]
rand (*n*, *m*) [Loadable Function]
rand ("*state*", *x*) [Loadable Function]
rand ("*seed*", *x*) [Loadable Function]

Return a matrix with random elements uniformly distributed on the interval (0, 1). The arguments are handled the same as the arguments for `eye`.

You can query the state of the random number generator using the form

```
v = rand ("state")
```

This returns a column vector *v* of length 625. Later, you can restore the random number generator to the state *v* using the form

```
rand ("state", v)
```

You may also initialize the state vector from an arbitrary vector of length <= 625 for *v*. This new state will be a hash based on the value of *v*, not *v* itself.

By default, the generator is initialized from `/dev/urandom` if it is available, otherwise from cpu time, wall clock time and the current fraction of a second.

To compute the pseudo-random sequence, `rand` uses the Mersenne Twister with a period of $2^{19937} - 1$ (See M. Matsumoto and T. Nishimura, *Mersenne Twister: A 623-dimensionally equidistributed uniform pseudorandom number generator*, ACM Trans. on Modeling and Computer Simulation Vol. 8, No. 1, January pp.3-30 1998, `http://www.math.sci.hiroshima-u.ac.jp/~m-mat/MT/emt.html`). Do **not** use for cryptography without securely hashing several returned values together, otherwise the generator state can be learned after reading 624 consecutive values.

Older versions of Octave used a different random number generator. The new generator is used by default as it is significantly faster than the old generator, and produces random numbers with a significantly longer cycle time. However, in some circumstances it might be desirable to obtain the same random sequences as used by the old generators. To do this the keyword "seed" is used to specify that the old generators should be use, as in

```
rand ("seed", val)
```

which sets the seed of the generator to *val*. The seed of the generator can be queried with

```
s = rand ("seed")
```

However, it should be noted that querying the seed will not cause `rand` to use the old generators, only setting the seed will. To cause `rand` to once again use the new generators, the keyword "state" should be used to reset the state of the `rand`.

See also: [randn], page 286, [rande], page 286, [randg], page 287, [randp], page 286.

randn (*x*) [Loadable Function]
randn (*n*, *m*) [Loadable Function]
randn ("*state*", *x*) [Loadable Function]
randn ("*seed*", *x*) [Loadable Function]

Return a matrix with normally distributed pseudo-random elements having zero mean and variance one. The arguments are handled the same as the arguments for `rand`.

By default, `randn` uses the Marsaglia and Tsang "Ziggurat technique" to transform from a uniform to a normal distribution. (G. Marsaglia and W.W. Tsang, *Ziggurat method for generating random variables*, J. Statistical Software, vol 5, 2000, http://www.jstatsoft.org/v05/i08/)

See also: [rand], page 285, [rande], page 286, [randg], page 287, [randp], page 286.

rande (*x*) [Loadable Function]
rande (*n*, *m*) [Loadable Function]
rande ("*state*", *x*) [Loadable Function]
rande ("*seed*", *x*) [Loadable Function]

Return a matrix with exponentially distributed random elements. The arguments are handled the same as the arguments for `rand`.

By default, `randn` uses the Marsaglia and Tsang "Ziggurat technique" to transform from a uniform to a exponential distribution. (G. Marsaglia and W.W. Tsang, *Ziggurat method for generating random variables*, J. Statistical Software, vol 5, 2000, http://www.jstatsoft.org/v05/i08/)

See also: [rand], page 285, [randn], page 286, [randg], page 287, [randp], page 286.

randp (*l*, *x*) [Loadable Function]
randp (*l*, *n*, *m*) [Loadable Function]
randp ("*state*", *x*) [Loadable Function]
randp ("*seed*", *x*) [Loadable Function]

Return a matrix with Poisson distributed random elements with mean value parameter given by the first argument, *l*. The arguments are handled the same as the arguments for `rand`, except for the argument *l*.

Five different algorithms are used depending on the range of *l* and whether or not *l* is a scalar or a matrix.

For scalar *l* <= 12, use direct method.
Press, et al., 'Numerical Recipes in C', Cambridge University Press, 1992.

For scalar *l* > 12, use rejection method.[1]
Press, et al., 'Numerical Recipes in C', Cambridge University Press, 1992.

For matrix *l* <= 10, use inversion method.[2]
Stadlober E., et al., WinRand source code, available via FTP.

For matrix *l* > 10, use patchwork rejection method.
Stadlober E., et al., WinRand source code, available via FTP, or H. Zechner, 'Efficient sampling from continuous and discrete unimodal distributions', Doctoral Dissertation, 156pp., Technical University Graz, Austria, 1994.

For *l* > 1e8, use normal approximation.
L. Montanet, et al., 'Review of Particle Properties', Physical Review D 50 p1284, 1994

See also: [rand], page 285, [randn], page 286, [rande], page 286, [randg], page 287.

randg (a, *x*) [Loadable Function]
randg (a, *n*, *m*) [Loadable Function]
randg ("*state*", *x*) [Loadable Function]
randg ("*seed*", *x*) [Loadable Function]

Return a matrix with `gamma(a,1)` distributed random elements. The arguments are handled the same as the arguments for `rand`, except for the argument *a*.

This can be used to generate many distributions:

`gamma (a, b)` for `a > -1`, `b > 0`
```
r = b * randg (a)
```

`beta (a, b)` for `a > -1`, `b > -1`
```
r1 = randg (a, 1)
r = r1 / (r1 + randg (b, 1))
```

`Erlang (a, n)`
```
r = a * randg (n)
```

`chisq (df)` for `df > 0`
```
r = 2 * randg (df / 2)
```

`t(df)` for `0 < df < inf` (use randn if df is infinite)
```
r = randn () / sqrt (2 * randg (df / 2) / df)
```

`F (n1, n2)` for `0 < n1`, `0 < n2`
```
## r1 equals 1 if n1 is infinite
r1 = 2 * randg (n1 / 2) / n1
## r2 equals 1 if n2 is infinite
r2 = 2 * randg (n2 / 2) / n2
r = r1 / r2
```

negative `binomial (n, p)` for `n > 0`, `0 < p <= 1`
```
r = randp ((1 - p) / p * randg (n))
```

non-central `chisq (df, L)`, for `df >= 0` and `L > 0`
(use chisq if `L = 0`)

```
r = randp (L / 2)
r(r > 0) = 2 * randg (r(r > 0))
r(df > 0) += 2 * randg (df(df > 0)/2)
```

`Dirichlet (a1, ... ak)`

```
r = (randg (a1), ..., randg (ak))
r = r / sum (r)
```

See also: [rand], page 285, [randn], page 286, [rande], page 286, [randp], page 286.

The generators operate in the new or old style together, it is not possible to mix the two. Initializing any generator with `"state"` or `"seed"` causes the others to switch to the same style for future calls.

The state of each generator is independent and calls to different generators can be interleaved without affecting the final result. For example,

```
rand ("state", [11, 22, 33]);
randn ("state", [44, 55, 66]);
u = rand (100, 1);
n = randn (100, 1);
```

and

```
rand ("state", [11, 22, 33]);
randn ("state", [44, 55, 66]);
u = zeros (100, 1);
n = zeros (100, 1);
for i = 1:100
  u(i) = rand ();
  n(i) = randn ();
end
```

produce equivalent results. When the generators are initialized in the old style with `"seed"` only `rand` and `randn` are independent, because the old `rande`, `randg` and `randp` generators make calls to `rand` and `randn`.

The generators are initialized with random states at start-up, so that the sequences of random numbers are not the same each time you run Octave.[2] If you really do need to reproduce a sequence of numbers exactly, you can set the state or seed to a specific value.

If invoked without arguments, `rand` and `randn` return a single element of a random sequence.

The original `rand` and `randn` functions use Fortran code from RANLIB, a library of fortran routines for random number generation, compiled by Barry W. Brown and James Lovato of the Department of Biomathematics at The University of Texas, M.D. Anderson Cancer Center, Houston, TX 77030.

`randperm (`*n*`)` [Function File]

Return a row vector containing a random permutation of the integers from 1 to *n*.

---

[2] The old versions of `rand` and `randn` obtain their initial seeds from the system clock.

The functions `linspace` and `logspace` make it very easy to create vectors with evenly or logarithmically spaced elements. See Section 4.2 [Ranges], page 43.

`linspace (base, limit, n)` [Built-in Function]

Return a row vector with *n* linearly spaced elements between *base* and *limit*. If the number of elements is greater than one, then the *base* and *limit* are always included in the range. If *base* is greater than *limit*, the elements are stored in decreasing order. If the number of points is not specified, a value of 100 is used.

The `linspace` function always returns a row vector.

For compatibility with MATLAB, return the second argument if fewer than two values are requested.

`logspace (base, limit, n)` [Function File]

Similar to `linspace` except that the values are logarithmically spaced from 10^{base} to 10^{limit}.

If *limit* is equal to π, the points are between 10^{base} and π, *not* 10^{base} and 10^{π}, in order to be compatible with the corresponding MATLAB function.

Also for compatibility, return the second argument if fewer than two values are requested.

See also: [linspace], page 289.

16.5 Famous Matrices

The following functions return famous matrix forms.

`hadamard (n)` [Function File]

Construct a Hadamard matrix *Hn* of size *n*-by-*n*. The size *n* must be of the form `2 ^ k * p` in which *p* is one of 1, 12, 20 or 28. The returned matrix is normalized, meaning `Hn(:,1) == 1` and `H(1,:) == 1`.

Some of the properties of Hadamard matrices are:

- `kron (Hm, Hn)` is a Hadamard matrix of size *m*-by-*n*.
- `Hn * Hn' == n * eye (n)`.
- The rows of *Hn* are orthogonal.
- `det (A) <= abs(det (Hn))` for all *A* with `abs (A (i, j)) <= 1`.
- Multiply any row or column by -1 and still have a Hadamard matrix.

`hankel (c, r)` [Function File]

Return the Hankel matrix constructed given the first column *c*, and (optionally) the last row *r*. If the last element of *c* is not the same as the first element of *r*, the last element of *c* is used. If the second argument is omitted, it is assumed to be a vector of zeros with the same size as *c*.

A Hankel matrix formed from an m-vector *c*, and an n-vector *r*, has the elements

$$H(i,j) = \begin{cases} c_{i+j-1}, & i+j-1 \le m; \\ r_{i+j-m}, & \text{otherwise.} \end{cases}$$

See also: [vander], page 291, [sylvester_matrix], page 291, [hilb], page 290, [invhilb], page 290, [toeplitz], page 291.

`hilb (n)` [Function File]

Return the Hilbert matrix of order *n*. The i, j element of a Hilbert matrix is defined as

$$H(i,j) = \frac{1}{(i+j-1)}$$

See also: [hankel], page 289, [vander], page 291, [sylvester_matrix], page 291, [invhilb], page 290, [toeplitz], page 291.

`invhilb (n)` [Function File]

Return the inverse of a Hilbert matrix of order *n*. This can be computed exactly using

$$\begin{aligned} A_{ij} &= -1^{i+j}(i+j-1)\binom{n+i-1}{n-j}\binom{n+j-1}{n-i}\binom{i+j-2}{i-2}^2 \\ &= \frac{p(i)p(j)}{(i+j-1)} \end{aligned}$$

where

$$p(k) = -1^k \binom{k+n-1}{k-1}\binom{n}{k}$$

The validity of this formula can easily be checked by expanding the binomial coefficients in both formulas as factorials. It can be derived more directly via the theory of Cauchy matrices: see J. W. Demmel, Applied Numerical Linear Algebra, page 92.

Compare this with the numerical calculation of `inverse (hilb (n))`, which suffers from the ill-conditioning of the Hilbert matrix, and the finite precision of your computer's floating point arithmetic.

See also: [hankel], page 289, [vander], page 291, [sylvester_matrix], page 291, [hilb], page 290, [toeplitz], page 291.

`magic (n)` [Function File]

Create an *n*-by-*n* magic square. Note that `magic (2)` is undefined since there is no 2-by-2 magic square.

`pascal (n, t)` [Function File]

Return the Pascal matrix of order *n* if `t = 0`. *t* defaults to 0. Return lower triangular Cholesky factor of the Pascal matrix if `t = 1`. This matrix is its own inverse, that is `pascal (n, 1) ^ 2 == eye (n)`. If `t = -1`, return its absolute value. This is the standard pascal triangle as a lower-triangular matrix. If `t = 2`, return a transposed and permuted version of `pascal (n, 1)`, which is the cube-root of the identity matrix. That is `pascal (n, 2) ^ 3 == eye (n)`.

See also: [hankel], page 289, [vander], page 291, [sylvester_matrix], page 291, [hilb], page 290, [invhilb], page 290, [toeplitz], page 291, [hadamard], page 289, [wilkinson], page 291, [compan], page 446, [rosser], page 290.

`rosser ()` [Function File]

Returns the Rosser matrix. This is a difficult test case used to test eigenvalue algorithms.

See also: [hankel], page 289, [vander], page 291, [sylvester_matrix], page 291, [hilb], page 290, [invhilb], page 290, [toeplitz], page 291, [hadamard], page 289, [wilkinson], page 291, [compan], page 446, [pascal], page 290.

`sylvester_matrix` (*k*) [Function File]

Return the Sylvester matrix of order $n = 2^k$.

See also: [hankel], page 289, [vander], page 291, [hilb], page 290, [invhilb], page 290, [toeplitz], page 291.

`toeplitz` (*c*, *r*) [Function File]

Return the Toeplitz matrix constructed given the first column *c*, and (optionally) the first row *r*. If the first element of *c* is not the same as the first element of *r*, the first element of *c* is used. If the second argument is omitted, the first row is taken to be the same as the first column.

A square Toeplitz matrix has the form:

$$\begin{bmatrix} c_0 & r_1 & r_2 & \cdots & r_n \\ c_1 & c_0 & r_1 & \cdots & r_{n-1} \\ c_2 & c_1 & c_0 & \cdots & r_{n-2} \\ \vdots & \vdots & \vdots & \ddots & \vdots \\ c_n & c_{n-1} & c_{n-2} & \ldots & c_0 \end{bmatrix}$$

See also: [hankel], page 289, [vander], page 291, [sylvester_matrix], page 291, [hilb], page 290, [invhilb], page 290.

`vander` (*c*, *n*) [Function File]

Return the Vandermonde matrix whose next to last column is *c*. If *n* is specified, it determines the number of columns; otherwise, *n* is taken to be equal to the length of *c*.

A Vandermonde matrix has the form:

$$\begin{bmatrix} c_1^{n-1} & \cdots & c_1^2 & c_1 & 1 \\ c_2^{n-1} & \cdots & c_2^2 & c_2 & 1 \\ \vdots & \ddots & \vdots & \vdots & \vdots \\ c_n^{n-1} & \cdots & c_n^2 & c_n & 1 \end{bmatrix}$$

See also: [hankel], page 289, [sylvester_matrix], page 291, [hilb], page 290, [invhilb], page 290, [toeplitz], page 291.

`wilkinson` (*n*) [Function File]

Return the Wilkinson matrix of order *n*.

See also: [hankel], page 289, [vander], page 291, [sylvester_matrix], page 291, [hilb], page 290, [invhilb], page 290, [toeplitz], page 291, [hadamard], page 289, [rosser], page 290, [compan], page 446, [pascal], page 290.

17 Arithmetic

Unless otherwise noted, all of the functions described in this chapter will work for real and complex scalar, vector, or matrix arguments. Functions described as *mapping functions* apply the given operation individually to each element when given a matrix argument. For example,

```
sin ([1, 2; 3, 4])
     ⇒  0.84147   0.90930
        0.14112  -0.75680
```

17.1 Exponents and Logarithms

`exp (x)` [Mapping Function]

Compute e^x for each element of *x*. To compute the matrix exponential, see Chapter 18 [Linear Algebra], page 315.

See also: [log], page 293.

`expm1 (x)` [Mapping Function]

Compute $e^x - 1$ accurately in the neighborhood of zero.

See also: [exp], page 293.

`log (x)` [Mapping Function]

Compute the natural logarithm, $\ln(x)$, for each element of *x*. To compute the matrix logarithm, see Chapter 18 [Linear Algebra], page 315.

See also: [exp], page 293, [log1p], page 293, [log2], page 293, [log10], page 293, [logspace], page 289.

`log1p (x)` [Mapping Function]

Compute $\ln(1 + x)$ accurately in the neighborhood of zero.

See also: [log], page 293, [exp], page 293, [expm1], page 293.

`log10 (x)` [Mapping Function]

Compute the base-10 logarithm of each element of *x*.

See also: [log], page 293, [log2], page 293, [logspace], page 289, [exp], page 293.

`log2 (x)` [Mapping Function]
`[f, e] = log2 (x)` [Mapping Function]

Compute the base-2 logarithm of each element of *x*.

If called with two output arguments, split *x* into binary mantissa and exponent so that $\frac{1}{2} \le |f| < 1$ and *e* is an integer. If $x = 0$, $f = e = 0$.

See also: [pow2], page 294, [log], page 293, [log10], page 293, [exp], page 293.

`nextpow2 (x)` [Function File]

If *x* is a scalar, return the first integer *n* such that $2^n \ge |x|$.

If *x* is a vector, return `nextpow2 (length (x))`.

See also: [pow2], page 294, [log2], page 293.

`nthroot (x, n)` [Function File]

Compute the n-th root of *x*, returning real results for real components of *x*. For example

```
nthroot (-1, 3)
⇒ -1
(-1) ^ (1 / 3)
⇒ 0.50000 - 0.86603i
```

`pow2 (x)` [Mapping Function]

`pow2 (f, e)` [Mapping Function]

With one argument, computes 2^x for each element of *x*.

With two arguments, returns $f \cdot 2^e$.

See also: [log2], page 293, [nextpow2], page 293.

`reallog (x)` [Function File]

Return the real-valued natural logarithm of each element of *x*. Report an error if any element results in a complex return value.

See also: [log], page 293, [realpow], page 294, [realsqrt], page 294.

`realpow (x, y)` [Function File]

Compute the real-valued, element-by-element power operator. This is equivalent to `x .^ y`, except that `realpow` reports an error if any return value is complex.

See also: [reallog], page 294, [realsqrt], page 294.

`realsqrt (x)` [Function File]

Return the real-valued square root of each element of *x*. Report an error if any element results in a complex return value.

See also: [sqrt], page 294, [realpow], page 294, [reallog], page 294.

`sqrt (x)` [Mapping Function]

Compute the square root of each element of *x*. If *x* is negative, a complex result is returned. To compute the matrix square root, see Chapter 18 [Linear Algebra], page 315.

See also: [realsqrt], page 294.

17.2 Complex Arithmetic

In the descriptions of the following functions, z is the complex number $x + iy$, where i is defined as $\sqrt{-1}$.

`abs (z)` [Mapping Function]

Compute the magnitude of *z*, defined as $|z| = \sqrt{x^2 + y^2}$.

For example,

```
abs (3 + 4i)
     ⇒ 5
```

arg (*z*) [Mapping Function]
angle (*z*) [Mapping Function]

Compute the argument of z, defined as, $\theta = atan2(y, x)$, in radians.

For example,

```
arg (3 + 4i)
     ⇒ 0.92730
```

conj (*z*) [Mapping Function]

Return the complex conjugate of z, defined as $\bar{z} = x - iy$.

See also: [real], page 295, [imag], page 295.

cplxpair (*z*) [Function File]
cplxpair (*z*, *tol*) [Function File]
cplxpair (*z*, *tol*, *dim*) [Function File]

Sort the numbers *z* into complex conjugate pairs ordered by increasing real part. Place the negative imaginary complex number first within each pair. Place all the real numbers (those with `abs (imag (z) / z) < tol)`) after the complex pairs.

If *tol* is unspecified the default value is 100*`eps`.

By default the complex pairs are sorted along the first non-singleton dimension of *z*. If *dim* is specified, then the complex pairs are sorted along this dimension.

Signal an error if some complex numbers could not be paired. Signal an error if all complex numbers are not exact conjugates (to within *tol*). Note that there is no defined order for pairs with identical real parts but differing imaginary parts.

```
cplxpair (exp(2i*pi*[0:4]'/5)) == exp(2i*pi*[3; 2; 4; 1; 0]/5)
```

imag (*z*) [Mapping Function]

Return the imaginary part of *z* as a real number.

See also: [real], page 295, [conj], page 295.

real (*z*) [Mapping Function]

Return the real part of *z*.

See also: [imag], page 295, [conj], page 295.

17.3 Trigonometry

Octave provides the following trigonometric functions where angles are specified in radians. To convert from degrees to radians multiply by $\pi/180$ (e.g., `sin (30 * pi/180)` returns the sine of 30 degrees). As an alternative, Octave provides a number of trigonometric functions which work directly on an argument specified in degrees. These functions are named after the base trigonometric function with a '`d`' suffix. For example, `sin` expects an angle in radians while `sind` expects an angle in degrees.

sin (*x*) [Mapping Function]

Compute the sine for each element of *x* in radians.

See also: [asin], page 296, [sind], page 298, [sinh], page 296.

`cos` (*x*) [Mapping Function]
Compute the cosine for each element of *x* in radians.

See also: [acos], page 296, [cosd], page 298, [cosh], page 297.

`tan` (*z*) [Mapping Function]
Compute the tangent for each element of *x* in radians.

See also: [atan], page 296, [tand], page 298, [tanh], page 297.

`sec` (*x*) [Mapping Function]
Compute the secant for each element of *x* in radians.

See also: [asec], page 296, [secd], page 298, [sech], page 297.

`csc` (*x*) [Mapping Function]
Compute the cosecant for each element of *x* in radians.

See also: [acsc], page 296, [cscd], page 298, [csch], page 297.

`cot` (*x*) [Mapping Function]
Compute the cotangent for each element of *x* in radians.

See also: [acot], page 296, [cotd], page 298, [coth], page 297.

`asin` (*x*) [Mapping Function]
Compute the inverse sine in radians for each element of *x*.

See also: [sin], page 295, [asind], page 298.

`acos` (*x*) [Mapping Function]
Compute the inverse cosine in radians for each element of *x*.

See also: [cos], page 296, [acosd], page 298.

`atan` (*x*) [Mapping Function]
Compute the inverse tangent in radians for each element of *x*.

See also: [tan], page 296, [atand], page 298.

`asec` (*x*) [Mapping Function]
Compute the inverse secant in radians for each element of *x*.

See also: [sec], page 296, [asecd], page 299.

`acsc` (*x*) [Mapping Function]
Compute the inverse cosecant in radians for each element of *x*.

See also: [csc], page 296, [acscd], page 299.

`acot` (*x*) [Mapping Function]
Compute the inverse cotangent in radians for each element of *x*.

See also: [cot], page 296, [acotd], page 299.

`sinh` (*x*) [Mapping Function]
Compute the hyperbolic sine for each element of *x*.

See also: [asinh], page 297, [cosh], page 297, [tanh], page 297.

`cosh` (*x*) [Mapping Function]

Compute the hyperbolic cosine for each element of *x*.

See also: [acosh], page 297, [sinh], page 296, [tanh], page 297.

`tanh` (*x*) [Mapping Function]

Compute hyperbolic tangent for each element of *x*.

See also: [atanh], page 297, [sinh], page 296, [cosh], page 297.

`sech` (*x*) [Mapping Function]

Compute the hyperbolic secant of each element of *x*.

See also: [asech], page 297.

`csch` (*x*) [Mapping Function]

Compute the hyperbolic cosecant of each element of *x*.

See also: [acsch], page 297.

`coth` (*x*) [Mapping Function]

Compute the hyperbolic cotangent of each element of *x*.

See also: [acoth], page 297.

`asinh` (*x*) [Mapping Function]

Compute the inverse hyperbolic sine for each element of *x*.

See also: [sinh], page 296.

`acosh` (*x*) [Mapping Function]

Compute the inverse hyperbolic cosine for each element of *x*.

See also: [cosh], page 297.

`atanh` (*x*) [Mapping Function]

Compute the inverse hyperbolic tangent for each element of *x*.

See also: [tanh], page 297.

`asech` (*x*) [Mapping Function]

Compute the inverse hyperbolic secant of each element of *x*.

See also: [sech], page 297.

`acsch` (*x*) [Mapping Function]

Compute the inverse hyperbolic cosecant of each element of *x*.

See also: [csch], page 297.

`acoth` (*x*) [Mapping Function]

Compute the inverse hyperbolic cotangent of each element of *x*.

See also: [coth], page 297.

`atan2` (*y*, *x*) [Mapping Function]

Compute atan (*y* / *x*) for corresponding elements of *y* and *x*. Signal an error if *y* and *x* do not match in size and orientation.

Octave provides the following trigonometric functions where angles are specified in degrees. These functions produce true zeros at the appropriate intervals rather than the small roundoff error that occurs when using radians. For example:

```
cosd (90)
     ⇒ 0
cos (pi/2)
     ⇒ 6.1230e-17
```

`sind` (*x*) [Function File]

Compute the sine for each element of *x* in degrees. Returns zero for elements where *x*/180 is an integer.

See also: [asind], page 298, [sin], page 295.

`cosd` (*x*) [Function File]

Compute the cosine for each element of *x* in degrees. Returns zero for elements where (*x*-90)/180 is an integer.

See also: [acosd], page 298, [cos], page 296.

`tand` (*x*) [Function File]

Compute the tangent for each element of *x* in degrees. Returns zero for elements where *x*/180 is an integer and `Inf` for elements where (*x*-90)/180 is an integer.

See also: [atand], page 298, [tan], page 296.

`secd` (*x*) [Function File]

Compute the secant for each element of *x* in degrees.

See also: [asecd], page 299, [sec], page 296.

`cscd` (*x*) [Function File]

Compute the cosecant for each element of *x* in degrees.

See also: [acscd], page 299, [csc], page 296.

`cotd` (*x*) [Function File]

Compute the cotangent for each element of *x* in degrees.

See also: [acotd], page 299, [cot], page 296.

`asind` (*x*) [Function File]

Compute the inverse sine in degrees for each element of *x*.

See also: [sind], page 298, [asin], page 296.

`acosd` (*x*) [Function File]

Compute the inverse cosine in degrees for each element of *x*.

See also: [cosd], page 298, [acos], page 296.

`atand` (*x*) [Function File]

Compute the inverse tangent in degrees for each element of *x*.

See also: [tand], page 298, [atan], page 296.

asecd (*x*) [Function File]

Compute the inverse secant in degrees for each element of *x*.

See also: [secd], page 298, [asec], page 296.

acscd (*x*) [Function File]

Compute the inverse cosecant in degrees for each element of *x*.

See also: [cscd], page 298, [acsc], page 296.

acotd (*x*) [Function File]

Compute the inverse cotangent in degrees for each element of *x*.

See also: [cotd], page 298, [acot], page 296.

17.4 Sums and Products

sum (*x*) [Built-in Function]
sum (*x*, *dim*) [Built-in Function]
sum (. . . , *'native'*) [Built-in Function]

Sum of elements along dimension *dim*. If *dim* is omitted, it defaults to 1 (column-wise sum).

As a special case, if *x* is a vector and *dim* is omitted, return the sum of the elements.

If the optional argument 'native' is given, then the sum is performed in the same type as the original argument, rather than in the default double type. For example

```
sum ([true, true])
  ⇒ 2
sum ([true, true], 'native')
  ⇒ true
```

See also: [cumsum], page 299, [sumsq], page 300, [prod], page 299.

prod (*x*) [Built-in Function]
prod (*x*, *dim*) [Built-in Function]

Product of elements along dimension *dim*. If *dim* is omitted, it defaults to 1 (column-wise products).

As a special case, if *x* is a vector and *dim* is omitted, return the product of the elements.

See also: [cumprod], page 300, [sum], page 299.

cumsum (*x*) [Built-in Function]
cumsum (*x*, *dim*) [Built-in Function]
cumsum (. . . , *'native'*) [Built-in Function]

Cumulative sum of elements along dimension *dim*. If *dim* is omitted, it defaults to 1 (column-wise cumulative sums).

As a special case, if *x* is a vector and *dim* is omitted, return the cumulative sum of the elements as a vector with the same orientation as *x*.

The "native" argument implies the summation is performed in native type. See `sum` for a complete description and example of the use of "native".

See also: [sum], page 299, [cumprod], page 300.

`cumprod` (*x*) [Built-in Function]
`cumprod` (*x*, *dim*) [Built-in Function]

Cumulative product of elements along dimension *dim*. If *dim* is omitted, it defaults to 1 (column-wise cumulative products).

As a special case, if *x* is a vector and *dim* is omitted, return the cumulative product of the elements as a vector with the same orientation as *x*.

See also: [prod], page 299, [cumsum], page 299.

`sumsq` (*x*) [Built-in Function]
`sumsq` (*x*, *dim*) [Built-in Function]

Sum of squares of elements along dimension *dim*. If *dim* is omitted, it defaults to 1 (column-wise sum of squares).

As a special case, if *x* is a vector and *dim* is omitted, return the sum of squares of the elements.

This function is conceptually equivalent to computing

```
sum (x .* conj (x), dim)
```

but it uses less memory and avoids calling `conj` if *x* is real.

See also: [sum], page 299.

`accumarray` (*subs*, *vals*, *sz*, *func*, *fillval*, *issparse*) [Function File]
`accumarray` (*csubs*, *vals*, ...) [Function File]

Create an array by accumulating the elements of a vector into the positions defined by their subscripts. The subscripts are defined by the rows of the matrix *subs* and the values by *vals*. Each row of *subs* corresponds to one of the values in *vals*.

The size of the matrix will be determined by the subscripts themselves. However, if *sz* is defined it determines the matrix size. The length of *sz* must correspond to the number of columns in *subs*.

The default action of `accumarray` is to sum the elements with the same subscripts. This behavior can be modified by defining the *func* function. This should be a function or function handle that accepts a column vector and returns a scalar. The result of the function should not depend on the order of the subscripts.

The elements of the returned array that have no subscripts associated with them are set to zero. Defining *fillval* to some other value allows these values to be defined.

By default `accumarray` returns a full matrix. If *issparse* is logically true, then a sparse matrix is returned instead.

An example of the use of `accumarray` is:

```
accumarray ([1,1,1;2,1,2;2,3,2;2,1,2;2,3,2], 101:105)
⇒ ans(:,:,1) = [101, 0, 0; 0, 0, 0]
   ans(:,:,2) = [0, 0, 0; 206, 0, 208]
```

17.5 Utility Functions

`ceil (x)` [Mapping Function]

Return the smallest integer not less than *x*. This is equivalent to rounding towards positive infinity. If *x* is complex, return `ceil (real (x)) + ceil (imag (x)) * I`.

```
ceil ([-2.7, 2.7])
   ⇒  -2   3
```

See also: [floor], page 302, [round], page 306, [fix], page 302.

`cross (x, y)` [Function File]
`cross (x, y, dim)` [Function File]

Compute the vector cross product of two 3-dimensional vectors *x* and *y*.

```
cross ([1,1,0], [0,1,1])
     ⇒ [ 1; -1; 1 ]
```

If *x* and *y* are matrices, the cross product is applied along the first dimension with 3 elements. The optional argument *dim* forces the cross product to be calculated along the specified dimension.

See also: [dot], page 316.

`d = del2 (m)` [Function File]
`d = del2 (m, h)` [Function File]
`d = del2 (m, dx, dy, ...)` [Function File]

Calculate the discrete Laplace operator (∇^2). For a 2-dimensional matrix *m* this is defined as

$$d = \frac{1}{4}\left(\frac{d^2}{dx^2}M(x,y) + \frac{d^2}{dy^2}M(x,y)\right)$$

For N-dimensional arrays the sum in parentheses is expanded to include second derivatives over the additional higher dimensions.

The spacing between evaluation points may be defined by *h*, which is a scalar defining the equidistant spacing in all dimensions. Alternatively, the spacing in each dimension may be defined separately by *dx*, *dy*, etc. A scalar spacing argument defines equidistant spacing, whereas a vector argument can be used to specify variable spacing. The length of the spacing vectors must match the respective dimension of *m*. The default spacing value is 1.

At least 3 data points are needed for each dimension. Boundary points are calculated from the linear extrapolation of interior points.

See also: [gradient], page 303, [diff], page 274.

`p = factor (q)` [Function File]
`[p, n] = factor (q)` [Function File]

Return prime factorization of *q*. That is, `prod (p) == q` and every element of *p* is a prime number. If `q == 1`, returns 1.

With two output arguments, return the unique primes *p* and their multiplicities. That is, `prod (p .^ n) == q`.

See also: [gcd], page 302, [lcm], page 303.

`factorial (n)` [Function File]

Return the factorial of *n* where *n* is a positive integer. If *n* is a scalar, this is equivalent to `prod (1:n)`. For vector or matrix arguments, return the factorial of each element in the array. For non-integers see the generalized factorial function `gamma`.

See also: [prod], page 299, [gamma], page 309.

`fix (x)` [Mapping Function]

Truncate fractional portion of *x* and return the integer portion. This is equivalent to rounding towards zero. If *x* is complex, return `fix (real (x)) + fix (imag (x)) * I`.

```
fix ([-2.7, 2.7])
   ⇒ -2   2
```

See also: [ceil], page 301, [floor], page 302, [round], page 306.

`floor (x)` [Mapping Function]

Return the largest integer not greater than *x*. This is equivalent to rounding towards negative infinity. If *x* is complex, return `floor (real (x)) + floor (imag (x)) * I`.

```
floor ([-2.7, 2.7])
     ⇒ -3   2
```

See also: [ceil], page 301, [round], page 306, [fix], page 302.

`fmod (x, y)` [Mapping Function]

Compute the floating point remainder of dividing *x* by *y* using the C library function `fmod`. The result has the same sign as *x*. If *y* is zero, the result is implementation-dependent.

See also: [mod], page 306, [rem], page 306.

`g = gcd (a)` [Loadable Function]
`g = gcd (a1, a2, ...)` [Loadable Function]
`[g, v1, ...] = gcd (a1, a2, ...)` [Loadable Function]

Compute the greatest common divisor of the elements of *a*. If more than one argument is given all arguments must be the same size or scalar. In this case the greatest common divisor is calculated for each element individually. All elements must be integers. For example,

```
gcd ([15, 20])
    ⇒  5
```

and

```
gcd ([15, 9], [20, 18])
    ⇒  5  9
```

Optional return arguments *v1*, etc., contain integer vectors such that,

$g = v_1 a_1 + v_2 a_2 + \cdots$

For backward compatibility with previous versions of this function, when all arguments are scalar, a single return argument *v1* containing all of the values of *v1*, ... is acceptable.

See also: [lcm], page 303, [factor], page 301.

`dx = gradient (m)` [Function File]
`[dx, dy, dz, ...] = gradient (m)` [Function File]
`[...] = gradient (m, s)` [Function File]
`[...] = gradient (m, x, y, z, ...)` [Function File]
`[...] = gradient (f, x0)` [Function File]
`[...] = gradient (f, x0, s)` [Function File]
`[...] = gradient (f, x0, x, y, ...)` [Function File]

Calculate the gradient of sampled data or a function. If *m* is a vector, calculate the one-dimensional gradient of *m*. If *m* is a matrix the gradient is calculated for each dimension.

`[dx, dy] = gradient (m)` calculates the one dimensional gradient for *x* and *y* direction if *m* is a matrix. Additional return arguments can be use for multi-dimensional matrices.

A constant spacing between two points can be provided by the *s* parameter. If *s* is a scalar, it is assumed to be the spacing for all dimensions. Otherwise, separate values of the spacing can be supplied by the *x*, ... arguments. Scalar values specify an equidistant spacing. Vector values for the *x*, ... arguments specify the coordinate for that dimension. The length must match their respective dimension of *m*.

At boundary points a linear extrapolation is applied. Interior points are calculated with the first approximation of the numerical gradient

```
y'(i) = 1/(x(i+1)-x(i-1)) * (y(i-1)-y(i+1)).
```

If the first argument *f* is a function handle, the gradient of the function at the points in *x0* is approximated using central difference. For example, `gradient (@cos, 0)` approximates the gradient of the cosine function in the point $x0 = 0$. As with sampled data, the spacing values between the points from which the gradient is estimated can be set via the *s* or *dx*, *dy*, ... arguments. By default a spacing of 1 is used.

See also: [diff], page 274, [del2], page 301.

`hypot (x, y)` [Built-in Function]

Compute the element-by-element square root of the sum of the squares of *x* and *y*. This is equivalent to `sqrt (x.^2 + y.^2)`, but calculated in a manner that avoids overflows for large values of *x* or *y*.

`lcm (x)` [Mapping Function]
`lcm (x, ...)` [Mapping Function]

Compute the least common multiple of the elements of *x*, or of the list of all arguments. For example,

```
lcm (a1, ..., ak)
```

is the same as

```
lcm ([a1, ..., ak]).
```

All elements must be the same size or scalar.

See also: [factor], page 301, [gcd], page 302.

`list_primes (n)` [Function File]

List the first *n* primes. If *n* is unspecified, the first 25 primes are listed.

The algorithm used is from page 218 of the TEXbook.

See also: [primes], page 306, [isprime], page 53.

`max` (*x*) [Loadable Function]
`max` (*x*, *y*) [Loadable Function]
`max` (*x*, *y*, *dim*) [Loadable Function]
`[w, iw] = max (x)` [Loadable Function]

For a vector argument, return the maximum value. For a matrix argument, return the maximum value from each column, as a row vector, or over the dimension *dim* if defined. For two matrices (or a matrix and scalar), return the pair-wise maximum. Thus,

```
max (max (x))
```

returns the largest element of the matrix *x*, and

```
max (2:5, pi)
    ⇒  3.1416  3.1416  4.0000  5.0000
```

compares each element of the range `2:5` with `pi`, and returns a row vector of the maximum values.

For complex arguments, the magnitude of the elements are used for comparison.

If called with one input and two output arguments, `max` also returns the first index of the maximum value(s). Thus,

```
[x, ix] = max ([1, 3, 5, 2, 5])
    ⇒  x = 5
        ix = 3
```

See also: [min], page 304, [cummax], page 305, [cummin], page 305.

`min` (*x*) [Loadable Function]
`min` (*x*, *y*) [Loadable Function]
`min` (*x*, *y*, *dim*) [Loadable Function]
`[w, iw] = min (x)` [Loadable Function]

For a vector argument, return the minimum value. For a matrix argument, return the minimum value from each column, as a row vector, or over the dimension *dim* if defined. For two matrices (or a matrix and scalar), return the pair-wise minimum. Thus,

```
min (min (x))
```

returns the smallest element of *x*, and

```
min (2:5, pi)
    ⇒  2.0000  3.0000  3.1416  3.1416
```

compares each element of the range `2:5` with `pi`, and returns a row vector of the minimum values.

For complex arguments, the magnitude of the elements are used for comparison.

If called with one input and two output arguments, `min` also returns the first index of the minimum value(s). Thus,

```
[x, ix] = min ([1, 3, 0, 2, 0])
    ⇒  x = 0
       ix = 3
```

See also: [max], page 304, [cummin], page 305, [cummax], page 305.

`cummax` (*x*) [Loadable Function]
`cummax` (*x*, *dim*) [Loadable Function]
[*w*, *iw*] = `cummax` (*x*) [Loadable Function]

Return the cumulative maximum values along dimension *dim*. If *dim* is unspecified it defaults to column-wise operation. For example,

```
cummax ([1 3 2 6 4 5])
    ⇒  1   3   3   6   6   6
```

The call

```
[w, iw] = cummax (x, dim)
```

is equivalent to the following code:

```
w = iw = zeros (size (x));
idxw = idxx = repmat ({':'}, 1, ndims (x));
for i = 1:size (x, dim)
  idxw{dim} = i; idxx{dim} = 1:i;
  [w(idxw{:}), iw(idxw{:})] = max(x(idxx{:}), [], dim);
endfor
```

but computed in a much faster manner.

See also: [cummin], page 305, [max], page 304, [min], page 304.

`cummin` (*x*) [Loadable Function]
`cummin` (*x*, *dim*) [Loadable Function]
[*w*, *iw*] = `cummin` (*x*) [Loadable Function]

Return the cumulative minimum values along dimension *dim*. If *dim* is unspecified it defaults to column-wise operation. For example,

```
cummin ([5 4 6 2 3 1])
    ⇒  5   4   4   2   2   1
```

The call

```
  [w, iw] = cummin (x, dim)
```

is equivalent to the following code:

```
w = iw = zeros (size (x));
idxw = idxx = repmat ({':'}, 1, ndims (x));
for i = 1:size (x, dim)
  idxw{dim} = i; idxx{dim} = 1:i;
  [w(idxw{:}), iw(idxw{:})] = min(x(idxx{:}), [], dim);
endfor
```

but computed in a much faster manner.

See also: [cummax], page 305, [min], page 304, [max], page 304.

`mod (x, y)` [Mapping Function]

Compute the modulo of *x* and *y*. Conceptually this is given by

```
x - y .* floor (x ./ y)
```

and is written such that the correct modulus is returned for integer types. This function handles negative values correctly. That is, `mod (-1, 3)` is 2, not -1, as `rem (-1, 3)` returns. `mod (x, 0)` returns *x*.

An error results if the dimensions of the arguments do not agree, or if either of the arguments is complex.

See also: [rem], page 306, [fmod], page 302.

`primes (n)` [Function File]

Return all primes up to *n*.

The algorithm used is the Sieve of Erastothenes.

Note that if you need a specific number of primes you can use the fact the distance from one prime to the next is, on average, proportional to the logarithm of the prime. Integrating, one finds that there are about k primes less than $k \log(5k)$.

See also: [list_primes], page 303, [isprime], page 53.

`rem (x, y)` [Mapping Function]

Return the remainder of the division `x / y`, computed using the expression

```
x - y .* fix (x ./ y)
```

An error message is printed if the dimensions of the arguments do not agree, or if either of the arguments is complex.

See also: [mod], page 306, [fmod], page 302.

`round (x)` [Mapping Function]

Return the integer nearest to *x*. If *x* is complex, return `round (real (x)) + round (imag (x)) * I`.

```
round ([-2.7, 2.7])
     ⇒ -3   3
```

See also: [ceil], page 301, [floor], page 302, [fix], page 302.

`roundb (x)` [Mapping Function]

Return the integer nearest to *x*. If there are two nearest integers, return the even one (banker's rounding). If *x* is complex, return `roundb (real (x)) + roundb (imag (x)) * I`.

See also: [round], page 306.

`sign (x)` [Mapping Function]

Compute the *signum* function, which is defined as

$$\mathrm{sign}(x) = \begin{cases} 1, & x > 0; \\ 0, & x = 0; \\ -1, & x < 0. \end{cases}$$

For complex arguments, `sign` returns `x ./ abs (x)`.

17.6 Special Functions

`[a, ierr] = airy (k, z, opt)` [Loadable Function]

Compute Airy functions of the first and second kind, and their derivatives.

```
 K   Function   Scale factor (if 'opt' is supplied)
---  --------   ---------------------------------------
 0   Ai (Z)     exp ((2/3) * Z * sqrt (Z))
 1   dAi(Z)/dZ  exp ((2/3) * Z * sqrt (Z))
 2   Bi (Z)     exp (-abs (real ((2/3) * Z *sqrt (Z))))
 3   dBi(Z)/dZ  exp (-abs (real ((2/3) * Z *sqrt (Z))))
```

The function call `airy (z)` is equivalent to `airy (0, z)`.

The result is the same size as *z*.

If requested, *ierr* contains the following status information and is the same size as the result.

0. Normal return.
1. Input error, return `NaN`.
2. Overflow, return `Inf`.
3. Loss of significance by argument reduction results in less than half of machine accuracy.
4. Complete loss of significance by argument reduction, return `NaN`.
5. Error—no computation, algorithm termination condition not met, return `NaN`.

`[j, ierr] = besselj (alpha, x, opt)` [Loadable Function]
`[y, ierr] = bessely (alpha, x, opt)` [Loadable Function]
`[i, ierr] = besseli (alpha, x, opt)` [Loadable Function]
`[k, ierr] = besselk (alpha, x, opt)` [Loadable Function]
`[h, ierr] = besselh (alpha, k, x, opt)` [Loadable Function]

Compute Bessel or Hankel functions of various kinds:

`besselj` Bessel functions of the first kind. If the argument *opt* is supplied, the result is multiplied by `exp(-abs(imag(x)))`.

`bessely` Bessel functions of the second kind. If the argument *opt* is supplied, the result is multiplied by `exp(-abs(imag(x)))`.

`besseli` Modified Bessel functions of the first kind. If the argument *opt* is supplied, the result is multiplied by `exp(-abs(real(x)))`.

`besselk` Modified Bessel functions of the second kind. If the argument *opt* is supplied, the result is multiplied by `exp(x)`.

`besselh` Compute Hankel functions of the first ($k = 1$) or second ($k = 2$) kind. If the argument *opt* is supplied, the result is multiplied by `exp (-I*x)` for $k = 1$ or `exp (I*x)` for $k = 2$.

If *alpha* is a scalar, the result is the same size as *x*. If *x* is a scalar, the result is the same size as *alpha*. If *alpha* is a row vector and *x* is a column vector, the result is a matrix with `length (x)` rows and `length (alpha)` columns. Otherwise, *alpha* and *x* must conform and the result will be the same size.

The value of *alpha* must be real. The value of *x* may be complex.

If requested, *ierr* contains the following status information and is the same size as the result.

0. Normal return.
1. Input error, return `NaN`.
2. Overflow, return `Inf`.
3. Loss of significance by argument reduction results in less than half of machine accuracy.
4. Complete loss of significance by argument reduction, return `NaN`.
5. Error—no computation, algorithm termination condition not met, return `NaN`.

`beta (a, b)` [Mapping Function]

For real inputs, return the Beta function,

$$B(a,b) = \frac{\Gamma(a)\Gamma(b)}{\Gamma(a+b)}.$$

`betainc (x, a, b)` [Mapping Function]

Return the incomplete Beta function,

$$\beta(x,a,b) = B(a,b)^{-1} \int_0^x t^{(a-z)}(1-t)^{(b-1)}dt.$$

If x has more than one component, both *a* and *b* must be scalars. If *x* is a scalar, *a* and *b* must be of compatible dimensions.

`betaln (a, b)` [Mapping Function]

Return the log of the Beta function,

$$B(a,b) = \log \frac{\Gamma(a)\Gamma(b)}{\Gamma(a+b)}.$$

See also: [beta], page 308, [betainc], page 308, [gammaln], page 311.

`bincoeff (n, k)` [Mapping Function]

Return the binomial coefficient of *n* and *k*, defined as

$$\binom{n}{k} = \frac{n(n-1)(n-2)\cdots(n-k+1)}{k!}$$

For example,

```
bincoeff (5, 2)
     ⇒ 10
```

In most cases, the `nchoosek` function is faster for small scalar integer arguments. It also warns about loss of precision for big arguments.

See also: [nchoosek], page 418.

`commutation_matrix (m, n)` [Function File]

Return the commutation matrix $K_{m,n}$ which is the unique $mn \times mn$ matrix such that $K_{m,n} \cdot \text{vec}(A) = \text{vec}(A^T)$ for all $m \times n$ matrices A.

If only one argument m is given, $K_{m,m}$ is returned.

See Magnus and Neudecker (1988), Matrix differential calculus with applications in statistics and econometrics.

`duplication_matrix (n)` [Function File]

Return the duplication matrix D_n which is the unique $n^2 \times n(n+1)/2$ matrix such that $D_n * \text{vech}(A) = \text{vec}(A)$ for all symmetric $n \times n$ matrices A.

See Magnus and Neudecker (1988), Matrix differential calculus with applications in statistics and econometrics.

`erf (z)` [Mapping Function]

Computes the error function,

$$\text{erf}(z) = \frac{2}{\sqrt{\pi}} \int_0^z e^{-t^2} dt$$

See also: [erfc], page 309, [erfinv], page 309.

`erfc (z)` [Mapping Function]

Computes the complementary error function, $1 - \text{erf}(z)$.

See also: [erf], page 309, [erfinv], page 309.

`erfinv (z)` [Mapping Function]

Computes the inverse of the error function.

See also: [erf], page 309, [erfc], page 309.

`gamma (z)` [Mapping Function]

Computes the Gamma function,

$$\Gamma(z) = \int_0^\infty t^{z-1} e^{-t} dt.$$

See also: [gammainc], page 309, [lgamma], page 311.

`gammainc (x, a)` [Mapping Function]

Compute the normalized incomplete gamma function,

$$\gamma(x, a) = \frac{\displaystyle\int_0^x e^{-t} t^{a-1} dt}{\Gamma(a)}$$

with the limiting value of 1 as x approaches infinity. The standard notation is $P(a, x)$, e.g., Abramowitz and Stegun (6.5.1).

If a is scalar, then `gammainc (x, a)` is returned for each element of x and vice versa.

If neither x nor a is scalar, the sizes of x and a must agree, and *gammainc* is applied element-by-element.

See also: [gamma], page 309, [lgamma], page 311.

`l = legendre (n, x)` [Function File]
`l = legendre (n, x, normalization)` [Function File]

Compute the Legendre function of degree *n* and order $m = 0 \ldots N$. The optional argument, *normalization*, may be one of `"unnorm"`, `"sch"`, or `"norm"`. The default is `"unnorm"`. The value of *n* must be a non-negative scalar integer.

If the optional argument *normalization* is missing or is `"unnorm"`, compute the Legendre function of degree *n* and order *m* and return all values for $m = 0 \ldots n$. The return value has one dimension more than *x*.

The Legendre Function of degree *n* and order *m*:

```
 m         m       2  m/2   d^m
P(x) = (-1) * (1-x  )     * ----  P (x)
 n                          dx^m   n
```

with Legendre polynomial of degree *n*:

```
          1     d^n   2    n
P (x) = ------ [----(x - 1)  ]
 n      2^n n!  dx^n
```

`legendre (3, [-1.0, -0.9, -0.8])` returns the matrix:

```
 x  |   -1.0   |   -0.9   |  -0.8
------------------------------------
m=0 | -1.00000 | -0.47250 | -0.08000
m=1 |  0.00000 | -1.99420 | -1.98000
m=2 |  0.00000 | -2.56500 | -4.32000
m=3 |  0.00000 | -1.24229 | -3.24000
```

If the optional argument `normalization` is `"sch"`, compute the Schmidt semi-normalized associated Legendre function. The Schmidt semi-normalized associated Legendre function is related to the unnormalized Legendre functions by the following:

For Legendre functions of degree n and order 0:

```
  0       0
SP (x) = P (x)
  n       n
```

For Legendre functions of degree n and order m:

```
  m       m          m    2(n-m)! 0.5
SP (x) = P (x) * (-1)  * [-------]
  n       n               (n+m)!
```

If the optional argument *normalization* is `"norm"`, compute the fully normalized associated Legendre function. The fully normalized associated Legendre function is related to the unnormalized Legendre functions by the following:

For Legendre functions of degree *n* and order *m*

```
  m       m          m    (n+0.5)(n-m)! 0.5
NP (x) = P (x) * (-1)  * [-------------]
  n       n                   (n+m)!
```

`lgamma (x)` [Mapping Function]
`gammaln (x)` [Mapping Function]

Return the natural logarithm of the gamma function of *x*.

See also: [gamma], page 309, [gammainc], page 309.

17.7 Coordinate Transformations

`[theta, r] = cart2pol (x, y)` [Function File]
`[theta, r, z] = cart2pol (x, y, z)` [Function File]

Transform Cartesian to polar or cylindrical coordinates. *x*, *y* (and *z*) must be the same shape, or scalar. *theta* describes the angle relative to the positive x-axis. *r* is the distance to the z-axis (0, 0, z).

See also: [pol2cart], page 311, [cart2sph], page 311, [sph2cart], page 311.

`[x, y] = pol2cart (theta, r)` [Function File]
`[x, y, z] = pol2cart (theta, r, z)` [Function File]

Transform polar or cylindrical to Cartesian coordinates. *theta*, *r* (and *z*) must be the same shape, or scalar. *theta* describes the angle relative to the positive x-axis. *r* is the distance to the z-axis (0, 0, z).

See also: [cart2pol], page 311, [cart2sph], page 311, [sph2cart], page 311.

`[theta, phi, r] = cart2sph (x, y, z)` [Function File]

Transform Cartesian to spherical coordinates. *x*, *y* and *z* must be the same shape, or scalar. *theta* describes the angle relative to the positive x-axis. *phi* is the angle relative to the xy-plane. *r* is the distance to the origin (0, 0, 0).

See also: [pol2cart], page 311, [cart2pol], page 311, [sph2cart], page 311.

`[x, y, z] = sph2cart (theta, phi, r)` [Function File]

Transform spherical to Cartesian coordinates. *x*, *y* and *z* must be the same shape, or scalar. *theta* describes the angle relative to the positive x-axis. *phi* is the angle relative to the xy-plane. *r* is the distance to the origin (0, 0, 0).

See also: [pol2cart], page 311, [cart2pol], page 311, [cart2sph], page 311.

17.8 Mathematical Constants

`e` [Built-in Function]
`e (n)` [Built-in Function]
`e (n, m)` [Built-in Function]
`e (n, m, k, ...)` [Built-in Function]
`e (..., class)` [Built-in Function]

Return a scalar, matrix, or N-dimensional array whose elements are all equal to the base of natural logarithms. The constant e satisfies the equation $\log(e) = 1$.

When called with no arguments, return a scalar with the value e. When called with a single argument, return a square matrix with the dimension specified. When called with more than one scalar argument the first two arguments are taken as the number of rows and columns and any further arguments specify additional matrix dimensions.

The optional argument *class* specifies the return type and may be either "double" or "single".

`pi` [Built-in Function]
`pi (`*n*`)` [Built-in Function]
`pi (`*n*, *m*`)` [Built-in Function]
`pi (`*n*, *m*, *k*, ...`)` [Built-in Function]
`pi (`..., *class*`)` [Built-in Function]

Return a scalar, matrix, or N-dimensional array whose elements are all equal to the ratio of the circumference of a circle to its diameter(π). Internally, `pi` is computed as '`4.0 * atan (1.0)`'.

When called with no arguments, return a scalar with the value of π. When called with a single argument, return a square matrix with the dimension specified. When called with more than one scalar argument the first two arguments are taken as the number of rows and columns and any further arguments specify additional matrix dimensions. The optional argument *class* specifies the return type and may be either "double" or "single".

`I` [Built-in Function]
`I (`*n*`)` [Built-in Function]
`I (`*n*, *m*`)` [Built-in Function]
`I (`*n*, *m*, *k*, ...`)` [Built-in Function]
`I (`..., *class*`)` [Built-in Function]

Return a scalar, matrix, or N-dimensional array whose elements are all equal to the pure imaginary unit, defined as $\sqrt{-1}$. I, and its equivalents i, J, and j, are functions so any of the names may be reused for other purposes (such as i for a counter variable).

When called with no arguments, return a scalar with the value i. When called with a single argument, return a square matrix with the dimension specified. When called with more than one scalar argument the first two arguments are taken as the number of rows and columns and any further arguments specify additional matrix dimensions. The optional argument *class* specifies the return type and may be either "double" or "single".

`Inf` [Built-in Function]
`Inf (`*n*`)` [Built-in Function]
`Inf (`*n*, *m*`)` [Built-in Function]
`Inf (`*n*, *m*, *k*, ...`)` [Built-in Function]
`Inf (`..., *class*`)` [Built-in Function]

Return a scalar, matrix or N-dimensional array whose elements are all equal to the IEEE representation for positive infinity.

Infinity is produced when results are too large to be represented using the the IEEE floating point format for numbers. Two common examples which produce infinity are division by zero and overflow.

```
[1/0 e^800]
⇒
Inf   Inf
```

When called with no arguments, return a scalar with the value 'Inf'. When called with a single argument, return a square matrix with the dimension specified. When called with more than one scalar argument the first two arguments are taken as the number of rows and columns and any further arguments specify additional matrix dimensions. The optional argument *class* specifies the return type and may be either "double" or "single".

See also: [isinf], page 274.

`NaN` [Built-in Function]
`NaN` (*n*) [Built-in Function]
`NaN` (*n*, *m*) [Built-in Function]
`NaN` (*n*, *m*, *k*, . . .) [Built-in Function]
`NaN` (. . ., *class*) [Built-in Function]

Return a scalar, matrix, or N-dimensional array whose elements are all equal to the IEEE symbol NaN (Not a Number). NaN is the result of operations which do not produce a well defined numerical result. Common operations which produce a NaN are arithmetic with infinity ($\infty - \infty$), zero divided by zero (0/0), and any operation involving another NaN value (5 + NaN).

Note that NaN always compares not equal to NaN (NaN != NaN). This behavior is specified by the IEEE standard for floating point arithmetic. To find NaN values, use the `isnan` function.

When called with no arguments, return a scalar with the value 'NaN'. When called with a single argument, return a square matrix with the dimension specified. When called with more than one scalar argument the first two arguments are taken as the number of rows and columns and any further arguments specify additional matrix dimensions. The optional argument *class* specifies the return type and may be either "double" or "single".

See also: [isnan], page 274.

`eps` [Built-in Function]
`eps` (*x*) [Built-in Function]
`eps` (*n*, *m*) [Built-in Function]
`eps` (*n*, *m*, *k*, . . .) [Built-in Function]
`eps` (. . ., *class*) [Built-in Function]

Return a scalar, matrix or N-dimensional array whose elements are all eps, the machine precision. More precisely, `eps` is the relative spacing between any two adjacent numbers in the machine's floating point system. This number is obviously system dependent. On machines that support IEEE floating point arithmetic, `eps` is approximately 2.2204×10^{-16} for double precision and 1.1921×10^{-7} for single precision.

When called with no arguments, return a scalar with the value `eps(1.0)`. Given a single argument *x*, return the distance between *x* and the next largest value. When called with more than one argument the first two arguments are taken as the number of rows and columns and any further arguments specify additional matrix dimensions. The optional argument *class* specifies the return type and may be either "double" or "single".

realmax [Built-in Function]
realmax (*n*) [Built-in Function]
realmax (*n*, *m*) [Built-in Function]
realmax (*n*, *m*, *k*, . . .) [Built-in Function]
realmax (. . ., *class*) [Built-in Function]

Return a scalar, matrix or N-dimensional array whose elements are all equal to the largest floating point number that is representable. The actual value is system dependent. On machines that support IEEE floating point arithmetic, `realmax` is approximately 1.7977×10^{308} for double precision and 3.4028×10^{38} for single precision.

When called with no arguments, return a scalar with the value `realmax("double")`. When called with a single argument, return a square matrix with the dimension specified. When called with more than one scalar argument the first two arguments are taken as the number of rows and columns and any further arguments specify additional matrix dimensions. The optional argument *class* specifies the return type and may be either "double" or "single".

See also: [realmin], page 314, [intmax], page 45, [bitmax], page 48.

realmin [Built-in Function]
realmin (*n*) [Built-in Function]
realmin (*n*, *m*) [Built-in Function]
realmin (*n*, *m*, *k*, . . .) [Built-in Function]
realmin (. . ., *class*) [Built-in Function]

Return a scalar, matrix or N-dimensional array whose elements are all equal to the smallest normalized floating point number that is representable. The actual value is system dependent. On machines that support IEEE floating point arithmetic, `realmin` is approximately 2.2251×10^{-308} for double precision and 1.1755×10^{-38} for single precision.

When called with no arguments, return a scalar with the value `realmin("double")`. When called with a single argument, return a square matrix with the dimension specified. When called with more than one scalar argument the first two arguments are taken as the number of rows and columns and any further arguments specify additional matrix dimensions. The optional argument *class* specifies the return type and may be either "double" or "single".

See also: [realmax], page 314, [intmin], page 46.

18 Linear Algebra

This chapter documents the linear algebra functions of Octave. Reference material for many of these functions may be found in Golub and Van Loan, *Matrix Computations, 2nd Ed.*, Johns Hopkins, 1989, and in the LAPACK *Users' Guide*, SIAM, 1992.

18.1 Techniques used for Linear Algebra

Octave includes a polymorphic solver, that selects an appropriate matrix factorization depending on the properties of the matrix itself. Generally, the cost of determining the matrix type is small relative to the cost of factorizing the matrix itself, but in any case the matrix type is cached once it is calculated, so that it is not re-determined each time it is used in a linear equation.

The selection tree for how the linear equation is solve or a matrix inverse is form is given by

1. If the matrix is upper or lower triangular sparse a forward or backward substitution using the LAPACK xTRTRS function, and goto 4.
2. If the matrix is square, hermitian with a real positive diagonal, attempt Cholesky factorization using the LAPACK xPOTRF function.
3. If the Cholesky factorization failed or the matrix is not hermitian with a real positive diagonal, and the matrix is square, factorize using the LAPACK xGETRF function.
4. If the matrix is not square, or any of the previous solvers flags a singular or near singular matrix, find a least squares solution using the LAPACK xGELSD function.

The user can force the type of the matrix with the `matrix_type` function. This overcomes the cost of discovering the type of the matrix. However, it should be noted that identifying the type of the matrix incorrectly will lead to unpredictable results, and so `matrix_type` should be used with care.

It should be noted that the test for whether a matrix is a candidate for Cholesky factorization, performed above and by the `matrix_type` function, does not give a certainty that the matrix is Hermitian. However, the attempt to factorize the matrix will quickly flag a non-Hermitian matrix.

18.2 Basic Matrix Functions

`aa = balance (a, opt)` [Loadable Function]
`[dd, aa] = balance (a, opt)` [Loadable Function]
`[d, p, aa] = balance (a, opt)` [Loadable Function]
`[cc, dd, aa, bb] = balance (a, b, opt)` [Loadable Function]

Compute `aa = dd \ a * dd` in which `aa` is a matrix whose row and column norms are roughly equal in magnitude, and `dd` = `p * d`, in which `p` is a permutation matrix and `d` is a diagonal matrix of powers of two. This allows the equilibration to be computed without roundoff. Results of eigenvalue calculation are typically improved by balancing first.

If two output values are requested, `balance` returns the diagonal `d` and the permutation `p` separately as vectors. In this case, `dd = eye(n)(:,p) * diag (d)`, where `n` is the matrix size.

If four output values are requested, compute `aa = cc*a*dd` and `bb = cc*b*dd)`, in which `aa` and `bb` have non-zero elements of approximately the same magnitude and `cc` and `dd` are permuted diagonal matrices as in `dd` for the algebraic eigenvalue problem.

The eigenvalue balancing option `opt` may be one of:

`"noperm"`, `"S"`
: Scale only; do not permute.

`"noscal"`, `"P"`
: Permute only; do not scale.

Algebraic eigenvalue balancing uses standard LAPACK routines.

Generalized eigenvalue problem balancing uses Ward's algorithm (SIAM Journal on Scientific and Statistical Computing, 1981).

`cond (a,p)` [Function File]

Compute the *p*-norm condition number of a matrix. `cond (a)` is defined as `norm (a, p) * norm (inv (a), p)`. By default `p=2` is used which implies a (relatively slow) singular value decomposition. Other possible selections are `p= 1, Inf, inf, 'Inf', 'fro'` which are generally faster.

See also: [condest], page 365, [rcond], page 319, [norm], page 318, [svd], page 326.

`[d, rcond] = det (a)` [Loadable Function]

Compute the determinant of *a* using LAPACK for full and UMFPACK for sparse matrices. Return an estimate of the reciprocal condition number if requested.

`dmult (a, b)` [Function File]

This function has been deprecated. Use the direct syntax `diag(A)*B` which is more readable and now also more efficient.

`dot (x, y, dim)` [Function File]

Computes the dot product of two vectors. If *x* and *y* are matrices, calculate the dot-product along the first non-singleton dimension. If the optional argument *dim* is given, calculate the dot-product along this dimension.

`lambda = eig (a)` [Loadable Function]
`lambda = eig (a, b)` [Loadable Function]
`[v, lambda] = eig (a)` [Loadable Function]
`[v, lambda] = eig (a, b)` [Loadable Function]

The eigenvalues (and eigenvectors) of a matrix are computed in a several step process which begins with a Hessenberg decomposition, followed by a Schur decomposition, from which the eigenvalues are apparent. The eigenvectors, when desired, are computed by further manipulations of the Schur decomposition.

The eigenvalues returned by `eig` are not ordered.

See also: [eigs], page 368.

`g = givens (x, y)` [Loadable Function]
`[c, s] = givens (x, y)` [Loadable Function]

Return a 2×2 orthogonal matrix

$$G = \begin{bmatrix} c & s \\ -s' & c \end{bmatrix}$$

such that

$$G \begin{bmatrix} x \\ y \end{bmatrix} = \begin{bmatrix} * \\ 0 \end{bmatrix}$$

with x and y scalars.

For example,

```
givens (1, 1)
     ⇒   0.70711   0.70711
        -0.70711   0.70711
```

[*g*, *y*] = planerot (*x*) [Function File]

Given a two-element column vector, returns the 2×2 orthogonal matrix G such that `y = g * x` and `y(2) = 0`.

See also: [givens], page 316.

[*x*, *rcond*] = inv (*a*) [Loadable Function]
[*x*, *rcond*] = inverse (*a*) [Loadable Function]

Compute the inverse of the square matrix *a*. Return an estimate of the reciprocal condition number if requested, otherwise warn of an ill-conditioned matrix if the reciprocal condition number is small.

If called with a sparse matrix, then in general *x* will be a full matrix, and so if possible forming the inverse of a sparse matrix should be avoided. It is significantly more accurate and faster to do `y = a \ b`, rather than `y = inv (a) * b`.

type = matrix_type (*a*) [Loadable Function]
a = matrix_type (*a*, *type*) [Loadable Function]
a = matrix_type (*a*, *'upper'*, *perm*) [Loadable Function]
a = matrix_type (*a*, *'lower'*, *perm*) [Loadable Function]
a = matrix_type (*a*, *'banded'*, *nl*, *nu*) [Loadable Function]

Identify the matrix type or mark a matrix as a particular type. This allows rapid for solutions of linear equations involving *a* to be performed. Called with a single argument, `matrix_type` returns the type of the matrix and caches it for future use. Called with more than one argument, `matrix_type` allows the type of the matrix to be defined.

The possible matrix types depend on whether the matrix is full or sparse, and can be one of the following

'unknown' Remove any previously cached matrix type, and mark type as unknown

'full' Mark the matrix as full.

'positive definite'
Probable full positive definite matrix.

'diagonal' Diagonal Matrix. (Sparse matrices only)

'permuted diagonal'
Permuted Diagonal matrix. The permutation does not need to be specifically indicated, as the structure of the matrix explicitly gives this. (Sparse matrices only)

'upper' Upper triangular. If the optional third argument *perm* is given, the matrix is assumed to be a permuted upper triangular with the permutations defined by the vector *perm*.

'lower' Lower triangular. If the optional third argument *perm* is given, the matrix is assumed to be a permuted lower triangular with the permutations defined by the vector *perm*.

'banded'
'banded positive definite'
Banded matrix with the band size of *nl* below the diagonal and *nu* above it. If *nl* and *nu* are 1, then the matrix is tridiagonal and treated with specialized code. In addition the matrix can be marked as probably a positive definite (Sparse matrices only)

'singular' The matrix is assumed to be singular and will be treated with a minimum norm solution

Note that the matrix type will be discovered automatically on the first attempt to solve a linear equation involving *a*. Therefore `matrix_type` is only useful to give Octave hints of the matrix type. Incorrectly defining the matrix type will result in incorrect results from solutions of linear equations, and so it is entirely the responsibility of the user to correctly identify the matrix type.

Also the test for positive definiteness is a low-cost test for a hermitian matrix with a real positive diagonal. This does not guarantee that the matrix is positive definite, but only that it is a probable candidate. When such a matrix is factorized, a Cholesky factorization is first attempted, and if that fails the matrix is then treated with an LU factorization. Once the matrix has been factorized, `matrix_type` will return the correct classification of the matrix.

norm (*a*, *p*, *opt*) [Built-in Function]

Compute the p-norm of the matrix *a*. If the second argument is missing, `p = 2` is assumed.

If *a* is a matrix (or sparse matrix):

p = `1` 1-norm, the largest column sum of the absolute values of *a*.

p = `2` Largest singular value of *a*.

p = `Inf` or `"inf"`
Infinity norm, the largest row sum of the absolute values of *a*.

p = `"fro"`
Frobenius norm of *a*, `sqrt (sum (diag (a' * a)))`.

other *p*, `p > 1`
maximum `norm (A*x, p)` such that `norm (x, p) == 1`

If *a* is a vector or a scalar:

p = `Inf` or `"inf"`
`max (abs (a))`.

p = `-Inf` `min (abs (a))`.

p = "fro"
: Frobenius norm of *a*, `sqrt (sumsq (abs (a)))`.

$p = 0$
: Hamming norm - the number of nonzero elements.

other *p*, `p > 1`
: p-norm of *a*, `(sum (abs (a) .^ p)) ^ (1/p)`.

other *p* `p < 1`
: the p-pseudonorm defined as above.

If `"rows"` is given as *opt*, the norms of all rows of the matrix *a* are returned as a column vector. Similarly, if `"columns"` or `"cols"` is passed column norms are computed.

See also: [cond], page 316, [svd], page 326.

`null (a, tol)` [Function File]

Return an orthonormal basis of the null space of *a*.

The dimension of the null space is taken as the number of singular values of *a* not greater than *tol*. If the argument *tol* is missing, it is computed as

```
max (size (a)) * max (svd (a)) * eps
```

`orth (a, tol)` [Function File]

Return an orthonormal basis of the range space of *a*.

The dimension of the range space is taken as the number of singular values of *a* greater than *tol*. If the argument *tol* is missing, it is computed as

```
max (size (a)) * max (svd (a)) * eps
```

`pinv (x, tol)` [Loadable Function]

Return the pseudoinverse of *x*. Singular values less than *tol* are ignored.

If the second argument is omitted, it is assumed that

```
tol = max (size (x)) * sigma_max (x) * eps,
```

where `sigma_max (x)` is the maximal singular value of *x*.

`rank (a, tol)` [Function File]

Compute the rank of *a*, using the singular value decomposition. The rank is taken to be the number of singular values of *a* that are greater than the specified tolerance *tol*. If the second argument is omitted, it is taken to be

```
tol = max (size (a)) * sigma(1) * eps;
```

where `eps` is machine precision and `sigma(1)` is the largest singular value of *a*.

`c = rcond (a)` [Loadable Function]

Compute the 1-norm estimate of the reciprocal condition as returned by LAPACK. If the matrix is well-conditioned then *c* will be near 1 and if the matrix is poorly conditioned it will be close to zero.

The matrix *a* must not be sparse. If the matrix is sparse then `condest (a)` or `rcond (full (a))` should be used instead.

See also: [inv], page 317.

`trace (a)` [Function File]

Compute the trace of *a*, `sum (diag (a))`.

`[r, k] = rref (a, tol)` [Function File]

Returns the reduced row echelon form of *a*. *tol* defaults to `eps * max (size (a)) * norm (a, inf)`.

Called with two return arguments, *k* returns the vector of "bound variables", which are those columns on which elimination has been performed.

18.3 Matrix Factorizations

`r = chol (a)` [Loadable Function]
`[r, p] = chol (a)` [Loadable Function]
`[r, p, q] = chol (s)` [Loadable Function]
`[r, p, q] = chol (s, 'vector')` [Loadable Function]
`[l, ...] = chol (..., 'lower')` [Loadable Function]

Compute the Cholesky factor, *r*, of the symmetric positive definite matrix *a*, where $R^T R = A$.

Called with one output argument `chol` fails if *a* or *s* is not positive definite. With two or more output arguments *p* flags whether the matrix was positive definite and `chol` does not fail. A zero value indicated that the matrix was positive definite and the *r* gives the factorization, and *p* will have a positive value otherwise.

If called with 3 outputs then a sparsity preserving row/column permutation is applied to *a* prior to the factorization. That is *r* is the factorization of `a(q,q)` such that $R^T R = Q^T A Q$.

The sparsity preserving permutation is generally returned as a matrix. However, given the flag 'vector', *q* will be returned as a vector such that $R^T R = A(Q, Q)$.

Called with either a sparse or full matrix and using the 'lower' flag, `chol` returns the lower triangular factorization such that $LL^T = A$.

In general the lower triangular factorization is significantly faster for sparse matrices.

See also: [cholinv], page 320, [chol2inv], page 320.

`cholinv (a)` [Loadable Function]

Use the Cholesky factorization to compute the inverse of the symmetric positive definite matrix *a*.

See also: [chol], page 320, [chol2inv], page 320.

`chol2inv (u)` [Loadable Function]

Invert a symmetric, positive definite square matrix from its Cholesky decomposition, *u*. Note that *u* should be an upper-triangular matrix with positive diagonal elements. `chol2inv (u)` provides `inv (u'*u)` but it is much faster than using `inv`.

See also: [chol], page 320, [cholinv], page 320.

`[R1, info] = cholupdate (R, u, op)` [Loadable Function]

Update or downdate a Cholesky factorization. Given an upper triangular matrix *R* and a column vector *u*, attempt to determine another upper triangular matrix *R1* such that

- *R1*'**R1* = *R*'**R* + *u***u*' if *op* is `"+"`
- *R1*'**R1* = *R*'**R* - *u***u*' if *op* is `"-"`

If *op* is `"-"`, *info* is set to

- 0 if the downdate was successful,
- 1 if *R*'**R* - *u***u*' is not positive definite,
- 2 if *R* is singular.

If *info* is not present, an error message is printed in cases 1 and 2.

See also: [chol], page 320, [qrupdate], page 324.

`[R1, info] = cholinsert (R, j, u)` [Loadable Function]

Given a Cholesky factorization of a real symmetric or complex hermitian positive definite matrix *A* = *R*'**R*, *R* upper triangular, return the Cholesky factorization of *A1*, where A1(p,p) = A, A1(:,j) = A1(j,:)' = u and p = [1:j-1,j+1:n+1]. u(j) should be positive. On return, *info* is set to

- 0 if the insertion was successful,
- 1 if *A1* is not positive definite,
- 2 if *R* is singular.

If *info* is not present, an error message is printed in cases 1 and 2.

See also: [chol], page 320, [cholupdate], page 320, [choldelete], page 321.

`R1 = choldelete (R, j)` [Loadable Function]

Given a Cholesky factorization of a real symmetric or complex hermitian positive definite matrix *A* = *R*'**R*, *R* upper triangular, return the Cholesky factorization of A(p,p), where p = [1:j-1,j+1:n+1].

See also: [chol], page 320, [cholupdate], page 320, [cholinsert], page 321.

`R1 = cholshift (R, i, j)` [Loadable Function]

Given a Cholesky factorization of a real symmetric or complex hermitian positive definite matrix *A* = *R*'**R*, *R* upper triangular, return the Cholesky factorization of *A*(p,p), where p is the permutation

`p = [1:i-1, shift(i:j, 1), j+1:n]` if *i* < *j*

or

`p = [1:j-1, shift(j:i,-1), i+1:n]` if *j* < *i*.

See also: [chol], page 320, [cholinsert], page 321, [choldelete], page 321.

`h = hess (a)` [Loadable Function]

`[p, h] = hess (a)` [Loadable Function]

Compute the Hessenberg decomposition of the matrix *a*.

The Hessenberg decomposition is usually used as the first step in an eigenvalue computation, but has other applications as well (see Golub, Nash, and Van Loan, IEEE Transactions on Automatic Control, 1979). The Hessenberg decomposition is

$$A = PHP^T$$

where P is a square unitary matrix ($P^HP = I$), and H is upper Hessenberg ($H_{i,j} = 0, \forall i \geq j+1$).

`[l, u, p] = lu (a)` [Loadable Function]
`[l, u, p, q] = lu (s)` [Loadable Function]
`[l, u, p, q, r] = lu (s)` [Loadable Function]
`[...] = lu (s, thres)` [Loadable Function]
`y = lu (...)` [Loadable Function]
`[...] = lu (..., 'vector')` [Loadable Function]

Compute the LU decomposition of *a*. If *a* is full subroutines from LAPACK are used and if *a* is sparse then UMFPACK is used. The result is returned in a permuted form, according to the optional return value *p*. For example, given the matrix `a = [1, 2; 3, 4]`,

```
[l, u, p] = lu (a)
```

returns

```
l =

  1.00000  0.00000
  0.33333  1.00000

u =

  3.00000  4.00000
  0.00000  0.66667

p =

  0  1
  1  0
```

The matrix is not required to be square.

Called with two or three output arguments and a spare input matrix, then *lu* does not attempt to perform sparsity preserving column permutations. Called with a fourth output argument, the sparsity preserving column transformation *Q* is returned, such that `p * a * q = l * u`.

Called with a fifth output argument and a sparse input matrix, then *lu* attempts to use a scaling factor *r* on the input matrix such that `p * (r \ a) * q = l * u`. This typically leads to a sparser and more stable factorization.

An additional input argument *thres*, that defines the pivoting threshold can be given. *thres* can be a scalar, in which case it defines UMFPACK pivoting tolerance for both symmetric and unsymmetric cases. If *thres* is a two element vector, then the first element defines the pivoting tolerance for the unsymmetric UMFPACK pivoting strategy and the second the symmetric strategy. By default, the values defined by `spparms` are used and are by default `[0.1, 0.001]`.

Given the string argument 'vector', *lu* returns the values of *p q* as vector values, such that for full matrix, `a (p,:) = l * u`, and `r(p,:) * a (:, q) = l * u`.

With two output arguments, returns the permuted forms of the upper and lower triangular matrices, such that `a = l * u`. With one output argument *y*, then the matrix returned by the LAPACK routines is returned. If the input matrix is sparse

then the matrix *l* is embedded into *u* to give a return value similar to the full case. For both full and sparse matrices, *lu* looses the permutation information.

`[q, r, p] = qr (a)` [Loadable Function]
`[q, r, p] = qr (a, '0')` [Loadable Function]

Compute the QR factorization of *a*, using standard LAPACK subroutines. For example, given the matrix `a = [1, 2; 3, 4]`,

```
[q, r] = qr (a)
```

returns

```
q =

  -0.31623  -0.94868
  -0.94868   0.31623

r =

  -3.16228  -4.42719
   0.00000  -0.63246
```

The `qr` factorization has applications in the solution of least squares problems

$$\min_x \|Ax - b\|_2$$

for overdetermined systems of equations (i.e., A is a tall, thin matrix). The QR factorization is $QR = A$ where Q is an orthogonal matrix and R is upper triangular.

If given a second argument of '0', `qr` returns an economy-sized QR factorization, omitting zero rows of R and the corresponding columns of Q.

If the matrix *a* is full, the permuted QR factorization `[q, r, p] = qr (a)` forms the QR factorization such that the diagonal entries of `r` are decreasing in magnitude order. For example,given the matrix `a = [1, 2; 3, 4]`,

```
[q, r, p] = qr(a)
```

returns

```
q =

  -0.44721  -0.89443
  -0.89443   0.44721

r =

  -4.47214  -3.13050
   0.00000   0.44721

p =

   0  1
   1  0
```

The permuted `qr` factorization `[q, r, p] = qr (a)` factorization allows the construction of an orthogonal basis of `span (a)`.

If the matrix *a* is sparse, then compute the sparse QR factorization of *a*, using CSparse. As the matrix *Q* is in general a full matrix, this function returns the *Q*-less factorization *r* of *a*, such that `r = chol (a' * a)`.

If the final argument is the scalar `0` and the number of rows is larger than the number of columns, then an economy factorization is returned. That is *r* will have only `size (a,1)` rows.

If an additional matrix *b* is supplied, then `qr` returns *c*, where `c = q' * b`. This allows the least squares approximation of `a \ b` to be calculated as

```
[c,r] = spqr (a,b)
x = r \ c
```

`[Q1, R1] = qrupdate (Q, R, u, v)` [Loadable Function]

Given a QR factorization of a real or complex matrix $A = Q^*R$, Q unitary and R upper trapezoidal, return the QR factorization of $A + u^*v'$, where u and v are column vectors (rank-1 update) or matrices with equal number of columns (rank-k update). Notice that the latter case is done as a sequence of rank-1 updates; thus, for k large enough, it will be both faster and more accurate to recompute the factorization from scratch.

The QR factorization supplied may be either full (Q is square) or economized (R is square).

See also: [qr], page 323, [qrinsert], page 324, [qrdelete], page 324.

`[Q1, R1] = qrinsert (Q, R, j, x, orient)` [Loadable Function]

Given a QR factorization of a real or complex matrix $A = Q^*R$, Q unitary and R upper trapezoidal, return the QR factorization of [A(:,1:j-1) x A(:,j:n)], where *u* is a column vector to be inserted into *A* (if *orient* is `"col"`), or the QR factorization of [A(1:j-1,:);x;A(:,j:n)], where *x* is a row vector to be inserted into *A* (if *orient* is `"row"`).

The default value of *orient* is `"col"`. If *orient* is `"col"`, *u* may be a matrix and *j* an index vector resulting in the QR factorization of a matrix *B* such that B(:,*j*) gives *u* and B(:,*j*) = [] gives *A*. Notice that the latter case is done as a sequence of k insertions; thus, for k large enough, it will be both faster and more accurate to recompute the factorization from scratch.

If *orient* is `"col"`, the QR factorization supplied may be either full (Q is square) or economized (R is square).

If *orient* is `"row"`, full factorization is needed.

See also: [qr], page 323, [qrupdate], page 324, [qrdelete], page 324.

`[Q1, R1] = qrdelete (Q, R, j, orient)` [Loadable Function]

Given a QR factorization of a real or complex matrix $A = Q^*R$, Q unitary and R upper trapezoidal, return the QR factorization of [A(:,1:j-1) A(:,j+1:n)], i.e., *A* with one column deleted (if *orient* is `"col"`), or the QR factorization of [A(1:j-1,:);A(:,j+1:n)], i.e., *A* with one row deleted (if *orient* is "row").

The default value of *orient* is "col".

If *orient* is "col", *j* may be an index vector resulting in the QR factorization of a matrix B such that A(:,j) = [] gives B. Notice that the latter case is done as a sequence of k deletions; thus, for k large enough, it will be both faster and more accurate to recompute the factorization from scratch.

If *orient* is "col", the QR factorization supplied may be either full (Q is square) or economized (R is square).

If *orient* is "row", full factorization is needed.

See also: [qr], page 323, [qrinsert], page 324, [qrupdate], page 324.

`[Q1, R1] = qrshift (Q, R, i, j)` [Loadable Function]

Given a QR factorization of a real or complex matrix $A = Q*R$, Q unitary and R upper trapezoidal, return the QR factorization of A(:,p), where p is the permutation

`p = [1:i-1, shift(i:j, 1), j+1:n]` if $i < j$

or

`p = [1:j-1, shift(j:i,-1), i+1:n]` if $j < i$.

See also: [qr], page 323, [qrinsert], page 324, [qrdelete], page 324.

`lambda = qz (a, b)` [Loadable Function]

Generalized eigenvalue problem $Ax = sBx$, QZ decomposition. There are three ways to call this function:

1. `lambda = qz(A,B)`

   Computes the generalized eigenvalues λ of $(A - sB)$.

2. `[AA, BB, Q, Z, V, W, lambda] = qz (A, B)`

   Computes qz decomposition, generalized eigenvectors, and generalized eigenvalues of $(A - sB)$

   $$AV = BV\text{diag}(\lambda)$$

   $$W^TA = \text{diag}(\lambda)W^TB$$

   $$AA = Q^TAZ, BB = Q^TBZ$$

   with Q and Z orthogonal (unitary)$= I$

3. `[AA,BB,Z{, lambda}] = qz(A,B,opt)`

   As in form [2], but allows ordering of generalized eigenpairs for (e.g.) solution of discrete time algebraic Riccati equations. Form 3 is not available for complex matrices, and does not compute the generalized eigenvectors V, W, nor the orthogonal matrix Q.

   opt for ordering eigenvalues of the GEP pencil. The leading block of the revised pencil contains all eigenvalues that satisfy:

   "N" = unordered (default)

   "S" = small: leading block has all |lambda| <=1

   "B" = big: leading block has all |lambda| >= 1

"-" = negative real part: leading block has all eigenvalues in the open left half-plane

"+" = non-negative real part: leading block has all eigenvalues in the closed right half-plane

Note: qz performs permutation balancing, but not scaling (see balance). Order of output arguments was selected for compatibility with MATLAB

See also: [balance], page 315, [eig], page 316, [schur], page 326.

`[aa, bb, q, z] = qzhess (a, b)` [Function File]

Compute the Hessenberg-triangular decomposition of the matrix pencil `(a, b)`, returning `aa = q * a * z`, `bb = q * b * z`, with *q* and *z* orthogonal. For example,

```
[aa, bb, q, z] = qzhess ([1, 2; 3, 4], [5, 6; 7, 8])
     ⇒ aa = [ -3.02244, -4.41741;  0.92998,  0.69749 ]
     ⇒ bb = [ -8.60233, -9.99730;  0.00000, -0.23250 ]
     ⇒  q = [ -0.58124, -0.81373; -0.81373,  0.58124 ]
     ⇒  z = [ 1, 0; 0, 1 ]
```

The Hessenberg-triangular decomposition is the first step in Moler and Stewart's QZ decomposition algorithm.

Algorithm taken from Golub and Van Loan, *Matrix Computations, 2nd edition*.

`s = schur (a)` [Loadable Function]
`[u, s] = schur (a, opt)` [Loadable Function]

The Schur decomposition is used to compute eigenvalues of a square matrix, and has applications in the solution of algebraic Riccati equations in control (see `are` and `dare`). `schur` always returns $S = U^T AU$ where U is a unitary matrix ($U^T U$ is identity) and S is upper triangular. The eigenvalues of A (and S) are the diagonal elements of S. If the matrix A is real, then the real Schur decomposition is computed, in which the matrix U is orthogonal and S is block upper triangular with blocks of size at most 2×2 along the diagonal. The diagonal elements of S (or the eigenvalues of the 2×2 blocks, when appropriate) are the eigenvalues of A and S.

The eigenvalues are optionally ordered along the diagonal according to the value of `opt`. `opt = "a"` indicates that all eigenvalues with negative real parts should be moved to the leading block of S (used in `are`), `opt = "d"` indicates that all eigenvalues with magnitude less than one should be moved to the leading block of S (used in `dare`), and `opt = "u"`, the default, indicates that no ordering of eigenvalues should occur. The leading k columns of U always span the A-invariant subspace corresponding to the k leading eigenvalues of S.

`angle = subspace (a, B)` [Function File]

Determine the largest principal angle between two subspaces spanned by columns of matrices *a* and *b*.

`s = svd (a)` [Loadable Function]
`[u, s, v] = svd (a)` [Loadable Function]

Compute the singular value decomposition of *a*

$$A = USV^H$$

The function `svd` normally returns the vector of singular values. If asked for three return values, it computes U, S, and V. For example,

```
svd (hilb (3))
```

returns

```
ans =

  1.4083189
  0.1223271
  0.0026873
```

and

```
[u, s, v] = svd (hilb (3))
```

returns

```
u =

  -0.82704   0.54745   0.12766
  -0.45986  -0.52829  -0.71375
  -0.32330  -0.64901   0.68867

s =

  1.40832  0.00000  0.00000
  0.00000  0.12233  0.00000
  0.00000  0.00000  0.00269

v =

  -0.82704   0.54745   0.12766
  -0.45986  -0.52829  -0.71375
  -0.32330  -0.64901   0.68867
```

If given a second argument, `svd` returns an economy-sized decomposition, eliminating the unnecessary rows or columns of *u* or *v*.

`[`*housv*`, `*beta*`, `*zer*`] = housh (`*x*, *j*, *z*`)` [Function File]

Compute Householder reflection vector *housv* to reflect *x* to be the j-th column of identity, i.e.,

```
(I - beta*housv*housv')x =  norm(x)*e(j) if x(1) < 0,
(I - beta*housv*housv')x = -norm(x)*e(j) if x(1) >= 0
```

Inputs

x vector

j index into vector

z threshold for zero (usually should be the number 0)

Outputs (see Golub and Van Loan):

beta If beta = 0, then no reflection need be applied (zer set to 0)

housv householder vector

`[u, h, nu] = krylov (a, v, k, eps1, pflg)` [Function File]

Construct an orthogonal basis *u* of block Krylov subspace

```
[v a*v a^2*v ... a^(k+1)*v]
```

Using Householder reflections to guard against loss of orthogonality.

If *v* is a vector, then *h* contains the Hessenberg matrix such that `a*u == u*h+rk*ek'`, in which `rk = a*u(:,k)-u*h(:,k)`, and `ek'` is the vector `[0, 0, ..., 1]` of length `k`. Otherwise, *h* is meaningless.

If *v* is a vector and *k* is greater than `length(A)-1`, then *h* contains the Hessenberg matrix such that `a*u == u*h`.

The value of *nu* is the dimension of the span of the krylov subspace (based on *eps1*).

If *b* is a vector and *k* is greater than *m-1*, then *h* contains the Hessenberg decomposition of *a*.

The optional parameter *eps1* is the threshold for zero. The default value is 1e-12.

If the optional parameter *pflg* is nonzero, row pivoting is used to improve numerical behavior. The default value is 0.

Reference: Hodel and Misra, "Partial Pivoting in the Computation of Krylov Subspaces", to be submitted to Linear Algebra and its Applications

18.4 Functions of a Matrix

`expm (a)` [Function File]

Return the exponential of a matrix, defined as the infinite Taylor series

$$\exp(A) = I + A + \frac{A^2}{2!} + \frac{A^3}{3!} + \cdots$$

The Taylor series is *not* the way to compute the matrix exponential; see Moler and Van Loan, *Nineteen Dubious Ways to Compute the Exponential of a Matrix*, SIAM Review, 1978. This routine uses Ward's diagonal Padé approximation method with three step preconditioning (SIAM Journal on Numerical Analysis, 1977). Diagonal Padé approximations are rational polynomials of matrices $D_q(a)^{-1}N_q(a)$ whose Taylor series matches the first $2q+1$ terms of the Taylor series above; direct evaluation of the Taylor series (with the same preconditioning steps) may be desirable in lieu of the Padé approximation when $D_q(a)$ is ill-conditioned.

`logm (a)` [Function File]

Compute the matrix logarithm of the square matrix *a*. Note that this is currently implemented in terms of an eigenvalue expansion and needs to be improved to be more robust.

`[result, error_estimate] = sqrtm (a)` [Loadable Function]

Compute the matrix square root of the square matrix *a*.

Ref: Nicholas J. Higham. A new sqrtm for MATLAB. Numerical Analysis Report No. 336, Manchester Centre for Computational Mathematics, Manchester, England, January 1999.

See also: [expm], page 328, [logm], page 328.

`kron (a, b)` [Loadable Function]

Form the kronecker product of two matrices, defined block by block as

```
x = [a(i, j) b]
```

For example,

```
kron (1:4, ones (3, 1))
     ⇒  1  2  3  4
        1  2  3  4
        1  2  3  4
```

`x = syl (a, b, c)` [Loadable Function]

Solve the Sylvester equation

$$AX + XB + C = 0$$

using standard LAPACK subroutines. For example,

```
syl ([1, 2; 3, 4], [5, 6; 7, 8], [9, 10; 11, 12])
    ⇒ [ -0.50000, -0.66667; -0.66667, -0.50000 ]
```

18.5 Specialized Solvers

`bicgstab (A, b)` [Function File]
`bicgstab (A, b, tol, maxit, M1, M2, x0)` [Function File]

This procedure attempts to solve a system of linear equations A*x = b for x. The *A* must be square, symmetric and positive definite real matrix N*N. The *b* must be a one column vector with a length of N. The *tol* specifies the tolerance of the method, the default value is 1e-6. The *maxit* specifies the maximum number of iterations, the default value is min(20,N). The *M1* specifies a preconditioner, can also be a function handler which returns M\X. The *M2* combined with *M1* defines preconditioner as preconditioner=M1*M2. The *x0* is the initial guess, the default value is zeros(N,1).

The value *x* is a computed result of this procedure. The value *flag* can be 0 when we reach tolerance in *maxit* iterations, 1 when we don't reach tolerance in *maxit* iterations and 3 when the procedure stagnates. The value *relres* is a relative residual - norm(b-A*x)/norm(b). The value *iter* is an iteration number in which x was computed. The value *resvec* is a vector of *relres* for each iteration.

`cgs (A, b)` [Function File]
`cgs (A, b, tol, maxit, M1, M2, x0)` [Function File]

This procedure attempts to solve a system of linear equations A*x = b for x. The *A* must be square, symmetric and positive definite real matrix N*N. The *b* must be a one column vector with a length of N. The *tol* specifies the tolerance of the method, default value is 1e-6. The *maxit* specifies the maximum number of iteration, default value is MIN(20,N). The *M1* specifies a preconditioner, can also be a function handler which returns M\X. The *M2* combined with *M1* defines preconditioner as preconditioner=M1*M2. The *x0* is initial guess, default value is zeros(N,1).

19 Nonlinear Equations

Octave can solve sets of nonlinear equations of the form

$$f(x) = 0$$

using the function `fsolve`, which is based on the MINPACK subroutine `hybrd`. This is an iterative technique so a starting point will have to be provided. This also has the consequence that convergence is not guaranteed even if a solution exists.

`fsolve (`*fcn*, *x0*, *options*`)` [Function File]
`[`*x*, *fvec*, *info*, *output*, *fjac*`] =` *fsolve* `(`*fcn*, ...`)` [Function File]

Solve a system of nonlinear equations defined by the function *fcn*. *fcn* should accepts a vector (array) defining the unknown variables, and return a vector of left-hand sides of the equations. Right-hand sides are defined to be zeros. In other words, this function attempts to determine a vector *x* such that `fcn (x)` gives (approximately) all zeros. *x0* determines a starting guess. The shape of *x0* is preserved in all calls to *fcn*, but otherwise it is treated as a column vector. *options* is a structure specifying additional options. Currently, `fsolve` recognizes these options: `"FunValCheck"`, `"OutputFcn"`, `"TolX"`, `"TolFun"`, `"MaxIter"`, `"MaxFunEvals"`, `"Jacobian"`, `"Updating"` and `"ComplexEqn"`.

If `"Jacobian"` is `"on"`, it specifies that *fcn*, called with 2 output arguments, also returns the Jacobian matrix of right-hand sides at the requested point. `"TolX"` specifies the termination tolerance in the unknown variables, while `"TolFun"` is a tolerance for equations. Default is `1e-7` for both `"TolX"` and `"TolFun"`. If `"Updating"` is `"on"`, the function will attempt to use Broyden updates to update the Jacobian, in order to reduce the amount of jacobian calculations. If your user function always calculates the Jacobian (regardless of number of output arguments), this option provides no advantage and should be set to false.

`"ComplexEqn"` is `"on"`, `fsolve` will attempt to solve complex equations in complex variables, assuming that the equations possess a complex derivative (i.e., are holomorphic). If this is not what you want, should unpack the real and imaginary parts of the system to get a real system.

For description of the other options, see `optimset`.

On return, *fval* contains the value of the function *fcn* evaluated at *x*, and *info* may be one of the following values:

1 Converged to a solution point. Relative residual error is less than specified by TolFun.

2 Last relative step size was less that TolX.

3 Last relative decrease in residual was less than TolF.

0 Iteration limit exceeded.

-3 The trust region radius became excessively small.

Note: If you only have a single nonlinear equation of one variable, using `fzero` is usually a much better idea.

See also: [fzero], page 333, [optimset], page 411.

Note about user-supplied jacobians: As an inherent property of the algorithm, jacobian is always requested for a solution vector whose residual vector is already known, and it is the last accepted successful step. Often this will be one of the last two calls, but not always. If the savings by reusing intermediate results from residual calculation in jacobian calculation are significant, the best strategy is to employ OutputFcn: After a vector is evaluated for residuals, if OutputFcn is called with that vector, then the intermediate results should be saved for future jacobian evaluation, and should be kept until a jacobian evaluation is requested or until outputfcn is called with a different vector, in which case they should be dropped in favor of this most recent vector. A short example how this can be achieved follows:

```
function [fvec, fjac] = user_func (x, optimvalues, state)
persistent sav = [], sav0 = [];
if (nargin == 1)
  ## evaluation call
  if (nargout == 1)
    sav0.x = x; # mark saved vector
    ## calculate fvec, save results to sav0.
  elseif (nargout == 2)
    ## calculate fjac using sav.
  endif
else
  ## outputfcn call.
  if (all (x == sav0.x))
    sav = sav0;
  endif
  ## maybe output iteration status, etc.
endif
endfunction

 ....

fsolve (@user_func, x0, optimset ("OutputFcn", @user_func, ...))
```

Here is a complete example. To solve the set of equations

$$-2x^2 + 3xy + 4\sin(y) - 6 = 0$$
$$3x^2 - 2xy^2 + 3\cos(x) + 4 = 0$$

you first need to write a function to compute the value of the given function. For example:

```
function y = f (x)
  y(1) = -2*x(1)^2 + 3*x(1)*x(2)   + 4*sin(x(2)) - 6;
  y(2) =  3*x(1)^2 - 2*x(1)*x(2)^2 + 3*cos(x(1)) + 4;
endfunction
```

Then, call `fsolve` with a specified initial condition to find the roots of the system of equations. For example, given the function `f` defined above,

```
[x, fval, info] = fsolve (@f, [1; 2])
```

results in the solution

```
x =

  0.57983
  2.54621

fval =

  -5.7184e-10
   5.5460e-10

info = 1
```

A value of `info = 1` indicates that the solution has converged.

The function `perror` may be used to print English messages corresponding to the numeric error codes. For example,

```
perror ("fsolve", 1)
     ⊣ solution converged to requested tolerance
```

When no Jacobian is supplied (as in the example above) it is approximated numerically. This requires more function evaluations, and hence is less efficient. In the example above we could compute the Jacobian analytically as

$$\begin{bmatrix} \frac{\partial f_1}{\partial x_1} & \frac{\partial f_1}{\partial x_2} \\ \frac{\partial f_2}{\partial x_1} & \frac{\partial f_2}{\partial x_2} \end{bmatrix} = \begin{bmatrix} 3x_2 - 4x_1 & 4\cos(x_2) + 3x_1 \\ -2x_2^2 - 3\sin(x_1) + 6x_1 & -4x_1x_2 \end{bmatrix}$$

and compute it with the following Octave function

```
function J = jacobian(x)
  J(1,1) =  3*x(2) - 4*x(1);
  J(1,2) =  4*cos(x(2)) + 3*x(1);
  J(2,1) = -2*x(2)^2 - 3*sin(x(1)) + 6*x(1);
  J(2,2) = -4*x(1)*x(2);
endfunction
```

The Jacobian can then be used with the following call to `fsolve`:

```
[x, fval, info] = fsolve ({@f, @jacobian}, [1; 2]);
```

which gives the same solution as before.

[*x*, *fval*, *info*, *output*] = **fzero** (*fun*, *x0*, *options*) [Function File]

Find a zero point of a univariate function. *fun* should be a function handle or name. *x0* specifies a starting point. *options* is a structure specifying additional options. Currently, `fzero` recognizes these options: `"FunValCheck"`, `"OutputFcn"`, `"TolX"`, `"MaxIter"`, `"MaxFunEvals"`. For description of these options, see [optimset], page 411.

On exit, the function returns *x*, the approximate zero point and *fval*, the function value thereof. *info* is an exit flag that can have these values:

- 1 The algorithm converged to a solution.
- 0 Maximum number of iterations or function evaluations has been exhausted.
- -1 The algorithm has been terminated from user output function.
- -2 A general unexpected error.
- -3 A non-real value encountered.
- -4 A NaN value encountered.

See also: [optimset], page 411, [fsolve], page 331.

20 Diagonal and Permutation Matrices

20.1 Creating and Manipulating Diagonal and Permutation Matrices

A diagonal matrix is defined as a matrix that has zero entries outside the main diagonal; that is, $D_{ij} = 0$ if $i \neq j$ Most often, square diagonal matrices are considered; however, the definition can equally be applied to nonsquare matrices, in which case we usually speak of a rectangular diagonal matrix.

A permutation matrix is defined as a square matrix that has a single element equal to unity in each row and each column; all other elements are zero. That is, there exists a permutation (vector) p such that $P_{ij} = 1$ if $j = p_i$ and $P_{ij} = 0$ otherwise.

Octave provides special treatment of real and complex rectangular diagonal matrices, as well as permutation matrices. They are stored as special objects, using efficient storage and algorithms, facilitating writing both readable and efficient matrix algebra expressions in the Octave language.

20.1.1 Creating Diagonal Matrices

The most common and easiest way to create a diagonal matrix is using the built-in function *diag*. The expression `diag (v)`, with *v* a vector, will create a square diagonal matrix with elements on the main diagonal given by the elements of *v*, and size equal to the length of *v*. `diag (v, m, n)` can be used to construct a rectangular diagonal matrix. The result of these expressions will be a special diagonal matrix object, rather than a general matrix object.

Diagonal matrix with unit elements can be created using *eye*. Some other built-in functions can also return diagonal matrices. Examples include *balance* or *inv*.

Example:

```
  diag (1:4)
⇒
Diagonal Matrix

   1   0   0   0
   0   2   0   0
   0   0   3   0
   0   0   0   4

  diag(1:3,5,3)

⇒
Diagonal Matrix

   1   0   0
   0   2   0
   0   0   3
   0   0   0
   0   0   0
```

20.1.2 Creating Permutation Matrices

For creating permutation matrices, Octave does not introduce a new function, but rather overrides an existing syntax: permutation matrices can be conveniently created by indexing an identity matrix by permutation vectors. That is, if q is a permutation vector of length n, the expression

```
P = eye (n) (:, q);
```

will create a permutation matrix - a special matrix object.

```
eye (n) (q, :)
```

will also work (and create a row permutation matrix), as well as

```
eye (n) (q1, q2).
```

For example:

```
  eye (4) ([1,3,2,4],:)
⇒
Permutation Matrix

   1   0   0   0
   0   0   1   0
   0   1   0   0
   0   0   0   1

  eye (4) (:,[1,3,2,4])
⇒
Permutation Matrix

   1   0   0   0
   0   0   1   0
   0   1   0   0
   0   0   0   1
```

Mathematically, an identity matrix is both diagonal and permutation matrix. In Octave, `eye (n)` returns a diagonal matrix, because a matrix can only have one class. You can convert this diagonal matrix to a permutation matrix by indexing it by an identity permutation, as shown below. This is a special property of the identity matrix; indexing other diagonal matrices generally produces a full matrix.

```
  eye (3)
⇒
Diagonal Matrix

   1   0   0
   0   1   0
   0   0   1

  eye(3)(1:3,:)
⇒
Permutation Matrix

   1   0   0
   0   1   0
   0   0   1
```

Some other built-in functions can also return permutation matrices. Examples include *inv* or *lu*.

20.1.3 Explicit and Implicit Conversions

The diagonal and permutation matrices are special objects in their own right. A number of operations and built-in functions are defined for these matrices to use special, more efficient code than would be used for a full matrix in the same place. Examples are given in further sections.

To facilitate smooth mixing with full matrices, backward compatibility, and compatibility with MATLAB, the diagonal and permutation matrices should allow any operation that works on full matrices, and will either treat it specially, or implicitly convert themselves to full matrices.

Instances include matrix indexing, except for extracting a single element or a leading submatrix, indexed assignment, or applying most mapper functions, such as *exp*.

An explicit conversion to a full matrix can be requested using the built-in function *full*. It should also be noted that the diagonal and permutation matrix objects will cache the result of the conversion after it is first requested (explicitly or implicitly), so that subsequent conversions will be very cheap.

20.2 Linear Algebra with Diagonal and Permutation Matrices

As has been already said, diagonal and permutation matrices make it possible to use efficient algorithms while preserving natural linear algebra syntax. This section describes in detail the operations that are treated specially when performed on these special matrix objects.

20.2.1 Expressions Involving Diagonal Matrices

Assume D is a diagonal matrix. If M is a full matrix, then `D*M` will scale the rows of M. That means, if `S = D*M`, then for each pair of indices i,j it holds

$$S_{ij} = D_{ii} M_{ij}$$

Similarly, `M*D` will do a column scaling.

The matrix *D* may also be rectangular, m-by-n where `m != n`. If `m < n`, then the expression `D*M` is equivalent to

```
D(:,1:m) * M(1:m,:),
```

i.e., trailing `n-m` rows of *M* are ignored. If `m > n`, then `D*M` is equivalent to

```
[D(1:n,n) * M; zeros(m-n, columns (M))],
```

i.e., null rows are appended to the result. The situation for right-multiplication `M*D` is analogous.

The expressions `D \ M` and `M / D` perform inverse scaling. They are equivalent to solving a diagonal (or rectangular diagonal) in a least-squares minimum-norm sense. In exact arithmetics, this is equivalent to multiplying by a pseudoinverse. The pseudoinverse of a rectangular diagonal matrix is again a rectangular diagonal matrix with swapped dimensions, where each nonzero diagonal element is replaced by its reciprocal. The matrix division algorithms do, in fact, use division rather than multiplication by reciprocals for better numerical accuracy; otherwise, they honor the above definition. Note that a diagonal matrix is never truncated due to ill-conditioning; otherwise, it would not be much useful for scaling. This is typically consistent with linear algebra needs. A full matrix that only happens to be diagonal (an is thus not a special object) is of course treated normally.

Multiplication and division by diagonal matrices works efficiently also when combined with sparse matrices, i.e., `D*S`, where *D* is a diagonal matrix and *S* is a sparse matrix scales the rows of the sparse matrix and returns a sparse matrix. The expressions `S*D`, `D\S`, `S/D` work analogically.

If *D1* and *D2* are both diagonal matrices, then the expressions

```
D1 + D2
D1 - D2
D1 * D2
D1 / D2
D1 \ D2
```

again produce diagonal matrices, provided that normal dimension matching rules are obeyed. The relations used are same as described above.

Also, a diagonal matrix *D* can be multiplied or divided by a scalar, or raised to a scalar power if it is square, producing diagonal matrix result in all cases.

A diagonal matrix can also be transposed or conjugate-transposed, giving the expected result. Extracting a leading submatrix of a diagonal matrix, i.e., `D(1:m,1:n)`, will produce a diagonal matrix, other indexing expressions will implicitly convert to full matrix.

Adding a diagonal matrix to a full matrix only operates on the diagonal elements. Thus,

```
A = A + eps * eye (n)
```

is an efficient method of augmenting the diagonal of a matrix. Subtraction works analogically.

When involved in expressions with other element-by-element operators, `.*`, `./`, `.\` or `.^`, an implicit conversion to full matrix will take place. This is not always strictly necessary but chosen to facilitate better consistency with MATLAB.

20.2.2 Expressions Involving Permutation Matrices

If P is a permutation matrix and M a matrix, the expression `P*M` will permute the rows of M. Similarly, `M*P` will yield a column permutation. Matrix division `P\M` and `M/P` can be used to do inverse permutation.

The previously described syntax for creating permutation matrices can actually help an user to understand the connection between a permutation matrix and a permuting vector. Namely, the following holds, where `I = eye (n)` is an identity matrix:

```
I(p,:) * M = (I*M) (p,:) = M(p,:)
```

Similarly,

```
M * I(:,p) = (M*I) (:,p) = M(:,p)
```

The expressions `I(p,:)` and `I(:,p)` are permutation matrices.

A permutation matrix can be transposed (or conjugate-transposed, which is the same, because a permutation matrix is never complex), inverting the permutation, or equivalently, turning a row-permutation matrix into a column-permutation one. For permutation matrices, transpose is equivalent to inversion, thus `P\M` is equivalent to `P'*M`. Transpose of a permutation matrix (or inverse) is a constant-time operation, flipping only a flag internally, and thus the choice between the two above equivalent expressions for inverse permuting is completely up to the user's taste.

Multiplication and division by permutation matrices works efficiently also when combined with sparse matrices, i.e., `P*S`, where P is a permutation matrix and S is a sparse matrix permutes the rows of the sparse matrix and returns a sparse matrix. The expressions `S*P`, `P\S`, `S/P` work analogically.

Two permutation matrices can be multiplied or divided (if their sizes match), performing a composition of permutations. Also a permutation matrix can be indexed by a permutation vector (or two vectors), giving again a permutation matrix. Any other operations do not generally yield a permutation matrix and will thus trigger the implicit conversion.

20.3 Functions That Are Aware of These Matrices

This section lists the built-in functions that are aware of diagonal and permutation matrices on input, or can return them as output. Passed to other functions, these matrices will in general trigger an implicit conversion. (Of course, user-defined dynamically linked functions may also work with diagonal or permutation matrices).

20.3.1 Diagonal Matrix Functions

inv and *pinv* can be applied to a diagonal matrix, yielding again a diagonal matrix. *det* will use an efficient straightforward calculation when given a diagonal matrix, as well as *cond*. The following mapper functions can be applied to a diagonal matrix without converting it to a full one: *abs*, *real*, *imag*, *conj*, *sqrt*. A diagonal matrix can also be returned from the *balance* and *svd* functions. The *sparse* function will convert a diagonal matrix efficiently to a sparse matrix.

20.3.2 Permutation Matrix Functions

inv and *pinv* will invert a permutation matrix, preserving its specialness. *det* can be applied to a permutation matrix, efficiently calculating the sign of the permutation (which is equal to the determinant).

A permutation matrix can also be returned from the built-in functions *lu* and *qr*, if a pivoted factorization is requested.

The *sparse* function will convert a permutation matrix efficiently to a sparse matrix. The *find* function will also work efficiently with a permutation matrix, making it possible to conveniently obtain the permutation indices.

20.4 Some Examples of Usage

The following can be used to solve a linear system `A*x = b` using the pivoted LU factorization:

```
[L, U, P] = lu (A); ## now L*U = P*A
x = U \ L \ P*b;
```

This is how you normalize columns of a matrix X to unit norm:

```
s = norm (X, "columns");
X = diag (s) \ X;
```

The following expression is a way to efficiently calculate the sign of a permutation, given by a permutation vector p. It will also work in earlier versions of Octave, but slowly.

```
det (eye (length (p))(p, :))
```

Finally, here's how you solve a linear system `A*x = b` with Tikhonov regularization (ridge regression) using SVD (a skeleton only):

```
m = rows (A); n = columns (A);
[U, S, V] = svd (A);
## determine the regularization factor alpha
## alpha = ...
## transform to orthogonal basis
b = U'*b;
## Use the standard formula, replacing A with S.
## S is diagonal, so the following will be very fast and accurate.
x = (S'*S + alpha^2 * eye (n)) \ (S' * b);
## transform to solution basis
x = V*x;
```

20.5 The Differences in Treatment of Zero Elements

Making diagonal and permutation matrices special matrix objects in their own right and the consequent usage of smarter algorithms for certain operations implies, as a side effect, small differences in treating zeros. The contents of this section applies also to sparse matrices, discussed in the following chapter.

The IEEE standard defines the result of the expressions `0*Inf` and `0*NaN` as `NaN`, as it has been generally agreed that this is the best compromise. Numerical software dealing with structured and sparse matrices (including Octave) however, almost always makes a distinction between a "numerical zero" and an "assumed zero". A "numerical zero" is a zero value occurring in a place where any floating-point value could occur. It is normally stored somewhere in memory as an explicit value. An "assumed zero", on the contrary, is a zero matrix element implied by the matrix structure (diagonal, triangular) or a sparsity

pattern; its value is usually not stored explicitly anywhere, but is implied by the underlying data structure.

The primary distinction is that an assumed zero, when multiplied by any number, or divided by any nonzero number, yields *always* a zero, even when, e.g., multiplied by `Inf` or divided by `NaN`. The reason for this behavior is that the numerical multiplication is not actually performed anywhere by the underlying algorithm; the result is just assumed to be zero. Equivalently, one can say that the part of the computation involving assumed zeros is performed symbolically, not numerically.

This behavior not only facilitates the most straightforward and efficient implementation of algorithms, but also preserves certain useful invariants, like:

- scalar * diagonal matrix is a diagonal matrix
- sparse matrix / scalar preserves the sparsity pattern
- permutation matrix * matrix is equivalent to permuting rows

all of these natural mathematical truths would be invalidated by treating assumed zeros as numerical ones.

Note that certain competing software does not strictly follow this principle and converts assumed zeros to numerical zeros in certain cases, while not doing so in other cases. As of today, there are no intentions to mimic such behavior in Octave.

Examples of effects of assumed zeros vs. numerical zeros:

```
Inf * eye (3)
⇒
   Inf     0     0
     0   Inf     0
     0     0   Inf

Inf * speye (3)
⇒
Compressed Column Sparse (rows = 3, cols = 3, nnz = 3 [33%])

  (1, 1) -> Inf
  (2, 2) -> Inf
  (3, 3) -> Inf

Inf * full (eye (3))
⇒
   Inf   NaN   NaN
   NaN   Inf   NaN
   NaN   NaN   Inf
```

```
diag(1:3) * [NaN; 1; 1]
⇒
   NaN
     2
     3

sparse(1:3,1:3,1:3) * [NaN; 1; 1]
⇒
   NaN
     2
     3
[1,0,0;0,2,0;0,0,3] * [NaN; 1; 1]
⇒
   NaN
   NaN
   NaN
```

21 Sparse Matrices

21.1 The Creation and Manipulation of Sparse Matrices

The size of mathematical problems that can be treated at any particular time is generally limited by the available computing resources. Both, the speed of the computer and its available memory place limitation on the problem size.

There are many classes of mathematical problems which give rise to matrices, where a large number of the elements are zero. In this case it makes sense to have a special matrix type to handle this class of problems where only the non-zero elements of the matrix are stored. Not only does this reduce the amount of memory to store the matrix, but it also means that operations on this type of matrix can take advantage of the a-priori knowledge of the positions of the non-zero elements to accelerate their calculations.

A matrix type that stores only the non-zero elements is generally called sparse. It is the purpose of this document to discuss the basics of the storage and creation of sparse matrices and the fundamental operations on them.

21.1.1 Storage of Sparse Matrices

It is not strictly speaking necessary for the user to understand how sparse matrices are stored. However, such an understanding will help to get an understanding of the size of sparse matrices. Understanding the storage technique is also necessary for those users wishing to create their own oct-files.

There are many different means of storing sparse matrix data. What all of the methods have in common is that they attempt to reduce the complexity and storage given a-priori knowledge of the particular class of problems that will be solved. A good summary of the available techniques for storing sparse matrix is given by Saad[1]. With full matrices, knowledge of the point of an element of the matrix within the matrix is implied by its position in the computers memory. However, this is not the case for sparse matrices, and so the positions of the non-zero elements of the matrix must equally be stored.

An obvious way to do this is by storing the elements of the matrix as triplets, with two elements being their position in the array (rows and column) and the third being the data itself. This is conceptually easy to grasp, but requires more storage than is strictly needed.

The storage technique used within Octave is the compressed column format. In this format the position of each element in a row and the data are stored as previously. However, if we assume that all elements in the same column are stored adjacent in the computers memory, then we only need to store information on the number of non-zero elements in each column, rather than their positions. Thus assuming that the matrix has more non-zero elements than there are columns in the matrix, we win in terms of the amount of memory used.

In fact, the column index contains one more element than the number of columns, with the first element always being zero. The advantage of this is a simplification in the code, in that there is no special case for the first or last columns. A short example, demonstrating this in C is.

---

[1] Youcef Saad "SPARSKIT: A basic toolkit for sparse matrix computation", 1994, `http://www-users.cs.umn.edu/~saad/software/SPARSKIT/paper.ps`

```
for (j = 0; j < nc; j++)
  for (i = cidx (j); i < cidx(j+1); i++)
     printf ("non-zero element (%i,%i) is %d\n",
 ridx(i), j, data(i));
```

A clear understanding might be had by considering an example of how the above applies to an example matrix. Consider the matrix

```
1   2   0  0
0   0   0  3
0   0   0  4
```

The non-zero elements of this matrix are

```
(1, 1)  ⇒ 1
(1, 2)  ⇒ 2
(2, 4)  ⇒ 3
(3, 4)  ⇒ 4
```

This will be stored as three vectors *cidx*, *ridx* and *data*, representing the column indexing, row indexing and data respectively. The contents of these three vectors for the above matrix will be

```
cidx = [0, 1, 2, 2, 4]
ridx = [0, 0, 1, 2]
data = [1, 2, 3, 4]
```

Note that this is the representation of these elements with the first row and column assumed to start at zero, while in Octave itself the row and column indexing starts at one. Thus the number of elements in the *i*-th column is given by `cidx (i + 1) - cidx (i)`.

Although Octave uses a compressed column format, it should be noted that compressed row formats are equally possible. However, in the context of mixed operations between mixed sparse and dense matrices, it makes sense that the elements of the sparse matrices are in the same order as the dense matrices. Octave stores dense matrices in column major ordering, and so sparse matrices are equally stored in this manner.

A further constraint on the sparse matrix storage used by Octave is that all elements in the rows are stored in increasing order of their row index, which makes certain operations faster. However, it imposes the need to sort the elements on the creation of sparse matrices. Having disordered elements is potentially an advantage in that it makes operations such as concatenating two sparse matrices together easier and faster, however it adds complexity and speed problems elsewhere.

21.1.2 Creating Sparse Matrices

There are several means to create sparse matrix.

Returned from a function

There are many functions that directly return sparse matrices. These include *speye*, *sprand*, *diag*, etc.

Constructed from matrices or vectors

The function *sparse* allows a sparse matrix to be constructed from three vectors representing the row, column and data. Alternatively, the function *spconvert*

uses a three column matrix format to allow easy importation of data from elsewhere.

Created and then filled
: The function *sparse* or *spalloc* can be used to create an empty matrix that is then filled by the user

From a user binary program
: The user can directly create the sparse matrix within an oct-file.

There are several basic functions to return specific sparse matrices. For example the sparse identity matrix, is a matrix that is often needed. It therefore has its own function to create it as `speye (n)` or `speye (r, c)`, which creates an *n*-by-*n* or *r*-by-*c* sparse identity matrix.

Another typical sparse matrix that is often needed is a random distribution of random elements. The functions *sprand* and *sprandn* perform this for uniform and normal random distributions of elements. They have exactly the same calling convention, where `sprand (r, c, d)`, creates an *r*-by-*c* sparse matrix with a density of filled elements of *d*.

Other functions of interest that directly create sparse matrices, are *diag* or its generalization *spdiags*, that can take the definition of the diagonals of the matrix and create the sparse matrix that corresponds to this. For example

```
s = diag (sparse(randn(1,n)), -1);
```

creates a sparse (*n*+1)-by-(*n*+1) sparse matrix with a single diagonal defined.

`[b, c] = spdiags (a)` [Function File]
`b = spdiags (a, c)` [Function File]
`b = spdiags (v, c, a)` [Function File]
`b = spdiags (v, c, m, n)` [Function File]

A generalization of the function `diag`. Called with a single input argument, the non-zero diagonals *c* of *A* are extracted. With two arguments the diagonals to extract are given by the vector *c*.

The other two forms of `spdiags` modify the input matrix by replacing the diagonals. They use the columns of *v* to replace the columns represented by the vector *c*. If the sparse matrix *a* is defined then the diagonals of this matrix are replaced. Otherwise a matrix of *m* by *n* is created with the diagonals given by *v*.

Negative values of *c* represent diagonals below the main diagonal, and positive values of *c* diagonals above the main diagonal.

For example

```
spdiags (reshape (1:12, 4, 3), [-1 0 1], 5, 4)
⇒     5 10  0  0
      1  6 11  0
      0  2  7 12
      0  0  3  8
      0  0  0  4
```

`y = speye (m)` [Function File]
`y = speye (m, n)` [Function File]

`y = speye (sz)` [Function File]

Returns a sparse identity matrix. This is significantly more efficient than `sparse (eye (m))` as the full matrix is not constructed.

Called with a single argument a square matrix of size *m* by *m* is created. Otherwise a matrix of *m* by *n* is created. If called with a single vector argument, this argument is taken to be the size of the matrix to create.

`y = spfun (f,x)` [Function File]

Compute `f(x)` for the non-zero values of *x*. This results in a sparse matrix with the same structure as *x*. The function *f* can be passed as a string, a function handle or an inline function.

`spmax (x, y, dim)` [Mapping Function]
`[w, iw] = spmax (x)` [Mapping Function]

This function has been deprecated. Use `max` instead.

`spmin (x, y, dim)` [Mapping Function]
`[w, iw] = spmin (x)` [Mapping Function]

This function has been deprecated. Use `min` instead.

`y = spones (x)` [Function File]

Replace the non-zero entries of *x* with ones. This creates a sparse matrix with the same structure as *x*.

`sprand (m, n, d)` [Function File]
`sprand (s)` [Function File]

Generate a random sparse matrix. The size of the matrix will be *m* by *n*, with a density of values given by *d*. *d* should be between 0 and 1. Values will be uniformly distributed between 0 and 1.

Note: sometimes the actual density may be a bit smaller than *d*. This is unlikely to happen for large really sparse matrices.

If called with a single matrix argument, a random sparse matrix is generated wherever the matrix *S* is non-zero.

See also: [sprandn], page 346.

`sprandn (m, n, d)` [Function File]
`sprandn (s)` [Function File]

Generate a random sparse matrix. The size of the matrix will be *m* by *n*, with a density of values given by *d*. *d* should be between 0 and 1. Values will be normally distributed with mean of zero and variance 1.

Note: sometimes the actual density may be a bit smaller than *d*. This is unlikely to happen for large really sparse matrices.

If called with a single matrix argument, a random sparse matrix is generated wherever the matrix *S* is non-zero.

See also: [sprand], page 346.

sprandsym (*n*, *d*) [Function File]
sprandsym (*s*) [Function File]

Generate a symmetric random sparse matrix. The size of the matrix will be *n* by *n*, with a density of values given by *d*. *d* should be between 0 and 1. Values will be normally distributed with mean of zero and variance 1.

Note: sometimes the actual density may be a bit smaller than *d*. This is unlikely to happen for large really sparse matrices.

If called with a single matrix argument, a random sparse matrix is generated wherever the matrix *S* is non-zero in its lower triangular part.

See also: [sprand], page 346, [sprandn], page 346.

The recommended way for the user to create a sparse matrix, is to create two vectors containing the row and column index of the data and a third vector of the same size containing the data to be stored. For example

```
ri = ci = d = [];
for j = 1:c
  ri = [ri; randperm(r)(1:n)'];
  ci = [ci; j*ones(n,1)];
  d = [d; rand(n,1)];
endfor
s = sparse (ri, ci, d, r, c);
```

creates an *r*-by-*c* sparse matrix with a random distribution of *n* (<*r*) elements per column. The elements of the vectors do not need to be sorted in any particular order as Octave will sort them prior to storing the data. However, pre-sorting the data will make the creation of the sparse matrix faster.

The function *spconvert* takes a three or four column real matrix. The first two columns represent the row and column index respectively and the third and four columns, the real and imaginary parts of the sparse matrix. The matrix can contain zero elements and the elements can be sorted in any order. Adding zero elements is a convenient way to define the size of the sparse matrix. For example

```
s = spconvert ([1 2 3 4; 1 3 4 4; 1 2 3 0]')
⇒ Compressed Column Sparse (rows=4, cols=4, nnz=3)
      (1 , 1) -> 1
      (2 , 3) -> 2
      (3 , 4) -> 3
```

An example of creating and filling a matrix might be

```
k = 5;
nz = r * k;
s = spalloc (r, c, nz)
for j = 1:c
  idx = randperm (r);
  s (:, j) = [zeros(r - k, 1); ...
        rand(k, 1)] (idx);
endfor
```

It should be noted, that due to the way that the Octave assignment functions are written that the assignment will reallocate the memory used by the sparse matrix at each iteration of the above loop. Therefore the *spalloc* function ignores the *nz* argument and does not preassign the memory for the matrix. Therefore, it is vitally important that code using to above structure should be vectorized as much as possible to minimize the number of assignments and reduce the number of memory allocations.

FM = `full` (*SM*) [Loadable Function]

returns a full storage matrix from a sparse, diagonal, permutation matrix or a range.

See also: [sparse], page 348.

s = `spalloc` (*r*, *c*, *nz*) [Function File]

Returns an empty sparse matrix of size *r*-by-*c*. As Octave resizes sparse matrices at the first opportunity, so that no additional space is needed, the argument *nz* is ignored. This function is provided only for compatibility reasons.

It should be noted that this means that code like

```
k = 5;
nz = r * k;
s = spalloc (r, c, nz)
for j = 1:c
  idx = randperm (r);
  s (:, j) = [zeros(r - k, 1); rand(k, 1)] (idx);
endfor
```

will reallocate memory at each step. It is therefore vitally important that code like this is vectorized as much as possible.

See also: [sparse], page 348, [nzmax], page 349.

s = `sparse` (*a*) [Loadable Function]
s = `sparse` (*i*, *j*, *sv*, *m*, *n*, *nzmax*) [Loadable Function]
s = `sparse` (*i*, *j*, *sv*) [Loadable Function]
s = `sparse` (*i*, *j*, *s*, *m*, *n*, "*unique*") [Loadable Function]
s = `sparse` (*m*, *n*) [Loadable Function]

Create a sparse matrix from the full matrix or row, column, value triplets. If *a* is a full matrix, convert it to a sparse matrix representation, removing all zero values in the process.

Given the integer index vectors *i* and *j*, a 1-by-`nnz` vector of real of complex values *sv*, overall dimensions *m* and *n* of the sparse matrix. The argument `nzmax` is ignored but accepted for compatibility with MATLAB. If *m* or *n* are not specified their values are derived from the maximum index in the vectors *i* and *j* as given by *m* = `max (`*i*`)`, *n* = `max (`*j*`)`.

Note: if multiple values are specified with the same *i*, *j* indices, the corresponding values in *s* will be added.

The following are all equivalent:

```
s = sparse (i, j, s, m, n)
s = sparse (i, j, s, m, n, "summation")
s = sparse (i, j, s, m, n, "sum")
```

Given the option "unique". if more than two values are specified for the same *i*, *j* indices, the last specified value will be used.

`sparse(m, n)` is equivalent to `sparse ([], [], [], m, n, 0)`

If any of *sv*, *i* or *j* are scalars, they are expanded to have a common size.

See also: [full], page 348.

`x = spconvert (m)` [Function File]

This function converts for a simple sparse matrix format easily produced by other programs into Octave's internal sparse format. The input *x* is either a 3 or 4 column real matrix, containing the row, column, real and imaginary parts of the elements of the sparse matrix. An element with a zero real and imaginary part can be used to force a particular matrix size.

The above problem of memory reallocation can be avoided in oct-files. However, the construction of a sparse matrix from an oct-file is more complex than can be discussed here, and you are referred to chapter Appendix A [Dynamically Linked Functions], page 557, to have a full description of the techniques involved.

21.1.3 Finding out Information about Sparse Matrices

There are a number of functions that allow information concerning sparse matrices to be obtained. The most basic of these is *issparse* that identifies whether a particular Octave object is in fact a sparse matrix.

Another very basic function is *nnz* that returns the number of non-zero entries there are in a sparse matrix, while the function *nzmax* returns the amount of storage allocated to the sparse matrix. Note that Octave tends to crop unused memory at the first opportunity for sparse objects. There are some cases of user created sparse objects where the value returned by *nzmax* will not be the same as *nnz*, but in general they will give the same result. The function *spstats* returns some basic statistics on the columns of a sparse matrix including the number of elements, the mean and the variance of each column.

`issparse (expr)` [Loadable Function]

Return 1 if the value of the expression *expr* is a sparse matrix.

`scalar = nnz (a)` [Built-in Function]

Returns the number of non zero elements in *a*.

See also: [sparse], page 348.

`nonzeros (s)` [Function File]

Returns a vector of the non-zero values of the sparse matrix *s*.

`scalar = nzmax (SM)` [Built-in Function]

Return the amount of storage allocated to the sparse matrix *SM*. Note that Octave tends to crop unused memory at the first opportunity for sparse objects. There are some cases of user created sparse objects where the value returned by *nzmax* will not be the same as *nnz*, but in general they will give the same result.

See also: [sparse], page 348, [spalloc], page 348.

[*count*, *mean*, *var*] = spstats (*s*) [Function File]
[*count*, *mean*, *var*] = spstats (*s*, *j*) [Function File]

Return the stats for the non-zero elements of the sparse matrix *s*. *count* is the number of non-zeros in each column, *mean* is the mean of the non-zeros in each column, and *var* is the variance of the non-zeros in each column.

Called with two input arguments, if *s* is the data and *j* is the bin number for the data, compute the stats for each bin. In this case, bins can contain data values of zero, whereas with `spstats (s)` the zeros may disappear.

When solving linear equations involving sparse matrices Octave determines the means to solve the equation based on the type of the matrix as discussed in Section 21.2 [Sparse Linear Algebra], page 363. Octave probes the matrix type when the div (/) or ldiv (\) operator is first used with the matrix and then caches the type. However the *matrix_type* function can be used to determine the type of the sparse matrix prior to use of the div or ldiv operators. For example

```
a = tril (sprandn(1024, 1024, 0.02), -1) ...
    + speye(1024);
matrix_type (a);
ans = Lower
```

show that Octave correctly determines the matrix type for lower triangular matrices. *matrix_type* can also be used to force the type of a matrix to be a particular type. For example

```
a = matrix_type (tril (sprandn (1024, ...
   1024, 0.02), -1) + speye(1024), 'Lower');
```

This allows the cost of determining the matrix type to be avoided. However, incorrectly defining the matrix type will result in incorrect results from solutions of linear equations, and so it is entirely the responsibility of the user to correctly identify the matrix type

There are several graphical means of finding out information about sparse matrices. The first is the *spy* command, which displays the structure of the non-zero elements of the matrix. See Figure 21.1, for an example of the use of *spy*. More advanced graphical information can be obtained with the *treeplot*, *etreeplot* and *gplot* commands.

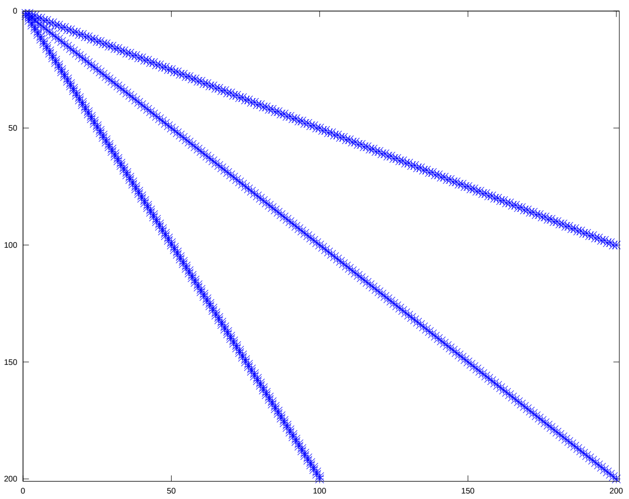

Figure 21.1: Structure of simple sparse matrix.

One use of sparse matrices is in graph theory, where the interconnections between nodes are represented as an adjacency matrix. That is, if the i-th node in a graph is connected to the j-th node. Then the ij-th node (and in the case of undirected graphs the ji-th node) of the sparse adjacency matrix is non-zero. If each node is then associated with a set of coordinates, then the *gplot* command can be used to graphically display the interconnections between nodes.

As a trivial example of the use of *gplot*, consider the example

```
A = sparse([2,6,1,3,2,4,3,5,4,6,1,5],
    [1,1,2,2,3,3,4,4,5,5,6,6],1,6,6);
xy = [0,4,8,6,4,2;5,0,5,7,5,7]';
gplot(A,xy)
```

which creates an adjacency matrix `A` where node 1 is connected to nodes 2 and 6, node 2 with nodes 1 and 3, etc. The coordinates of the nodes are given in the n-by-2 matrix `xy`. See Figure 21.2.

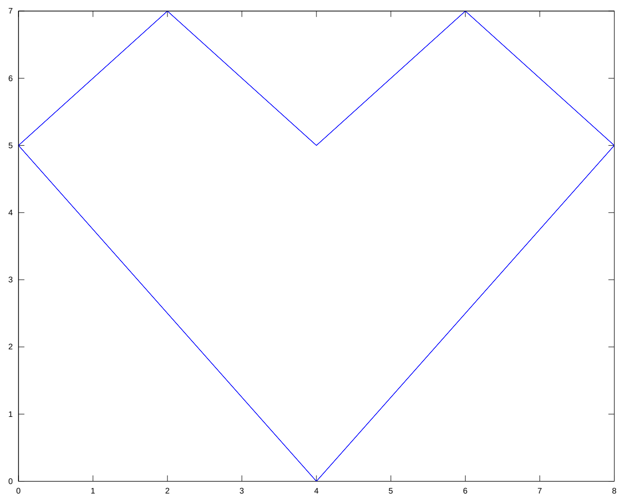

Figure 21.2: Simple use of the *gplot* command.

The dependencies between the nodes of a Cholesky factorization can be calculated in linear time without explicitly needing to calculate the Cholesky factorization by the `etree` command. This command returns the elimination tree of the matrix and can be displayed graphically by the command `treeplot(etree(A))` if `A` is symmetric or `treeplot(etree(A+A'))` otherwise.

`spy` (*x*) [Function File]
`spy` (..., *markersize*) [Function File]
`spy` (..., *line_spec*) [Function File]

Plot the sparsity pattern of the sparse matrix *x*. If the argument *markersize* is given as an scalar value, it is used to determine the point size in the plot. If the string *line_spec* is given it is passed to `plot` and determines the appearance of the plot.

See also: [plot], page 205.

p = `etree` (*s*) [Loadable Function]
p = `etree` (*s*, *typ*) [Loadable Function]
[*p*, *q*] = `etree` (*s*, *typ*) [Loadable Function]

Returns the elimination tree for the matrix *s*. By default *s* is assumed to be symmetric and the symmetric elimination tree is returned. The argument *typ* controls whether a symmetric or column elimination tree is returned. Valid values of *typ* are 'sym' or 'col', for symmetric or column elimination tree respectively

Called with a second argument, *etree* also returns the postorder permutations on the tree.

`etreeplot` (*tree*) [Function File]
`etreeplot` (*tree*, *node_style*, *edge_style*) [Function File]

Plot the elimination tree of the matrix *s* or `s+s'` if *s* in non-symmetric. The optional parameters *line_style* and *edge_style* define the output style.

See also: [treeplot], page 353, [gplot], page 353.

`gplot (a, xy)` [Function File]
`gplot (a, xy, line_style)` [Function File]
`[x, y] = gplot (a, xy)` [Function File]

Plot a graph defined by *A* and *xy* in the graph theory sense. *A* is the adjacency matrix of the array to be plotted and *xy* is an *n*-by-2 matrix containing the coordinates of the nodes of the graph.

The optional parameter *line_style* defines the output style for the plot. Called with no output arguments the graph is plotted directly. Otherwise, return the coordinates of the plot in *x* and *y*.

See also: [treeplot], page 353, [etreeplot], page 352, [spy], page 352.

`treeplot (tree)` [Function File]
`treeplot (tree, line_style, edge_style)` [Function File]

Produces a graph of tree or forest. The first argument is vector of predecessors, optional parameters *line_style* and *edge_style* define the output style. The complexity of the algorithm is O(n) in terms of is time and memory requirements.

See also: [etreeplot], page 352, [gplot], page 353.

`treelayout (Tree)` [Function File]
`treelayout (Tree, permutation)` [Function File]

treelayout lays out a tree or a forest. The first argument *Tree* is a vector of predecessors, optional parameter *permutation* is an optional postorder permutation. The complexity of the algorithm is O(n) in terms of time and memory requirements.

See also: [etreeplot], page 352, [gplot], page 353, [treeplot], page 353.

21.1.4 Basic Operators and Functions on Sparse Matrices

21.1.4.1 Sparse Functions

An important consideration in the use of the sparse functions of Octave is that many of the internal functions of Octave, such as *diag*, cannot accept sparse matrices as an input. The sparse implementation in Octave therefore uses the *dispatch* function to overload the normal Octave functions with equivalent functions that work with sparse matrices. However, at any time the sparse matrix specific version of the function can be used by explicitly calling its function name.

The table below lists all of the sparse functions of Octave. Note that the names of the specific sparse forms of the functions are typically the same as the general versions with a *sp* prefix. In the table below, and the rest of this article the specific sparse versions of the functions are used.

Generate sparse matrices:
: *spalloc*, *spdiags*, *speye*, *sprand*, *sprandn*, *sprandsym*

Sparse matrix conversion:
: *full*, *sparse*, *spconvert*

Manipulate sparse matrices
: *issparse*, *nnz*, *nonzeros*, *nzmax*, *spfun*, *spones*, *spy*

Graph Theory:
etree, *etreeplot*, *gplot*, *treeplot*

Sparse matrix reordering:
amd, *ccolamd*, *colamd*, *colperm*, *csymamd*, *dmperm*, *symamd*, *randperm*, *symrcm*

Linear algebra:
condest, *eigs*, *matrix_type*, *normest*, *sprank*, *spaugment*, *svds*

Iterative techniques:
luinc, *pcg*, *pcr*

Miscellaneous:
spparms, *symbfact*, *spstats*

In addition all of the standard Octave mapper functions (i.e., basic math functions that take a single argument) such as *abs*, etc. can accept sparse matrices. The reader is referred to the documentation supplied with these functions within Octave itself for further details.

21.1.4.2 The Return Types of Operators and Functions

The two basic reasons to use sparse matrices are to reduce the memory usage and to not have to do calculations on zero elements. The two are closely related in that the computation time on a sparse matrix operator or function is roughly linear with the number of non-zero elements.

Therefore, there is a certain density of non-zero elements of a matrix where it no longer makes sense to store it as a sparse matrix, but rather as a full matrix. For this reason operators and functions that have a high probability of returning a full matrix will always return one. For example adding a scalar constant to a sparse matrix will almost always make it a full matrix, and so the example

```
speye(3) + 0
⇒   1  0  0
  0  1  0
  0  0  1
```

returns a full matrix as can be seen.

Additionally, if `sparse_auto_mutate` is true, all sparse functions test the amount of memory occupied by the sparse matrix to see if the amount of storage used is larger than the amount used by the full equivalent. Therefore `speye (2) * 1` will return a full matrix as the memory used is smaller for the full version than the sparse version.

As all of the mixed operators and functions between full and sparse matrices exist, in general this does not cause any problems. However, one area where it does cause a problem is where a sparse matrix is promoted to a full matrix, where subsequent operations would resparsify the matrix. Such cases are rare, but can be artificially created, for example `(fliplr(speye(3)) + speye(3)) - speye(3)` gives a full matrix when it should give a sparse one. In general, where such cases occur, they impose only a small memory penalty.

There is however one known case where this behavior of Octave's sparse matrices will cause a problem. That is in the handling of the *diag* function. Whether *diag* returns a sparse or full matrix depending on the type of its input arguments. So

```
a = diag (sparse([1,2,3]), -1);
```

should return a sparse matrix. To ensure this actually happens, the *sparse* function, and other functions based on it like *speye*, always returns a sparse matrix, even if the memory used will be larger than its full representation.

val = **sparse_auto_mutate** () [Built-in Function]
old_val = **sparse_auto_mutate** (*new_val*) [Built-in Function]

Query or set the internal variable that controls whether Octave will automatically mutate sparse matrices to real matrices to save memory. For example,

```
s = speye(3);
sparse_auto_mutate (false)
s (:, 1) = 1;
typeinfo (s)
⇒ sparse matrix
sparse_auto_mutate (true)
s (1, :) = 1;
typeinfo (s)
⇒ matrix
```

Note that the `sparse_auto_mutate` option is incompatible with MATLAB, and so it is off by default.

21.1.4.3 Mathematical Considerations

The attempt has been made to make sparse matrices behave in exactly the same manner as there full counterparts. However, there are certain differences and especially differences with other products sparse implementations.

Firstly, the "./" and ".^" operators must be used with care. Consider what the examples

```
s = speye (4);
a1 = s .^ 2;
a2 = s .^ s;
a3 = s .^ -2;
a4 = s ./ 2;
a5 = 2 ./ s;
a6 = s ./ s;
```

will give. The first example of *s* raised to the power of 2 causes no problems. However *s* raised element-wise to itself involves a large number of terms `0 .^ 0` which is 1. There `s .^ s` is a full matrix.

Likewise `s .^ -2` involves terms like `0 .^ -2` which is infinity, and so `s .^ -2` is equally a full matrix.

For the "./" operator `s ./ 2` has no problems, but `2 ./ s` involves a large number of infinity terms as well and is equally a full matrix. The case of `s ./ s` involves terms like `0 ./ 0` which is a `NaN` and so this is equally a full matrix with the zero elements of *s* filled with `NaN` values.

The above behavior is consistent with full matrices, but is not consistent with sparse implementations in other products.

A particular problem of sparse matrices comes about due to the fact that as the zeros are not stored, the sign-bit of these zeros is equally not stored. In certain cases the sign-bit of zero is important. For example

```
a = 0 ./ [-1, 1; 1, -1];
b = 1 ./ a
⇒ -Inf            Inf
    Inf          -Inf
c = 1 ./ sparse (a)
⇒  Inf            Inf
    Inf           Inf
```

To correct this behavior would mean that zero elements with a negative sign-bit would need to be stored in the matrix to ensure that their sign-bit was respected. This is not done at this time, for reasons of efficiency, and so the user is warned that calculations where the sign-bit of zero is important must not be done using sparse matrices.

In general any function or operator used on a sparse matrix will result in a sparse matrix with the same or a larger number of non-zero elements than the original matrix. This is particularly true for the important case of sparse matrix factorizations. The usual way to address this is to reorder the matrix, such that its factorization is sparser than the factorization of the original matrix. That is the factorization of `L * U = P * S * Q` has sparser terms `L` and `U` than the equivalent factorization `L * U = S`.

Several functions are available to reorder depending on the type of the matrix to be factorized. If the matrix is symmetric positive-definite, then *symamd* or *csymamd* should be used. Otherwise *amd*, *colamd* or *ccolamd* should be used. For completeness the reordering functions *colperm* and *randperm* are also available.

See Figure 21.3, for an example of the structure of a simple positive definite matrix.

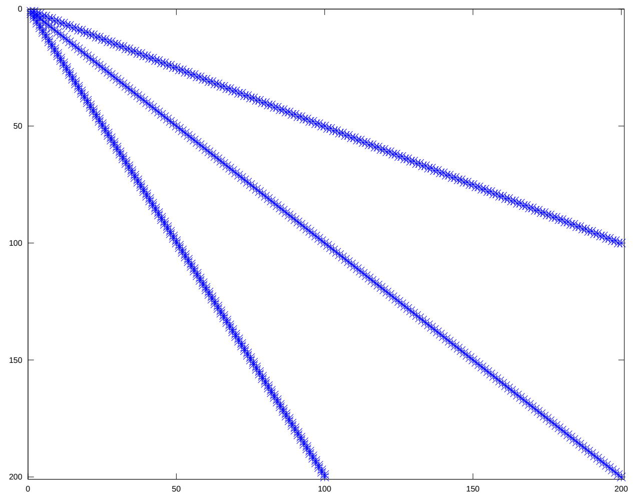

Figure 21.3: Structure of simple sparse matrix.

The standard Cholesky factorization of this matrix can be obtained by the same command that would be used for a full matrix. This can be visualized with the command `r`

`= chol(A); spy(r);`. See Figure 21.4. The original matrix had 598 non-zero terms, while this Cholesky factorization has 10200, with only half of the symmetric matrix being stored. This is a significant level of fill in, and although not an issue for such a small test case, can represents a large overhead in working with other sparse matrices.

The appropriate sparsity preserving permutation of the original matrix is given by *symamd* and the factorization using this reordering can be visualized using the command `q = symamd(A); r = chol(A(q,q)); spy(r)`. This gives 399 non-zero terms which is a significant improvement.

The Cholesky factorization itself can be used to determine the appropriate sparsity preserving reordering of the matrix during the factorization, In that case this might be obtained with three return arguments as `r[r, p, q] = chol(A); spy(r)`.

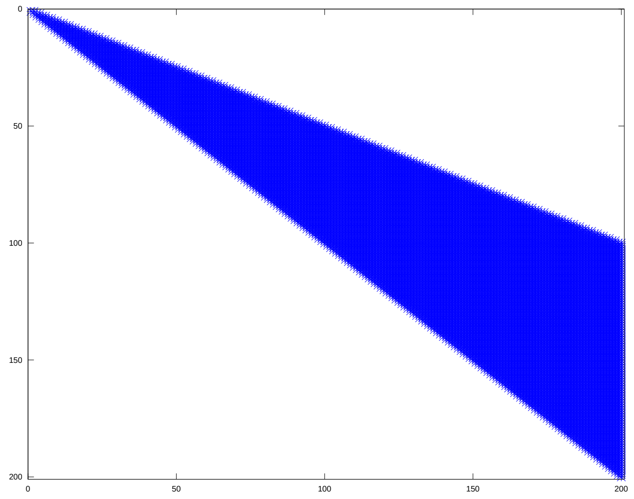

Figure 21.4: Structure of the un-permuted Cholesky factorization of the above matrix.

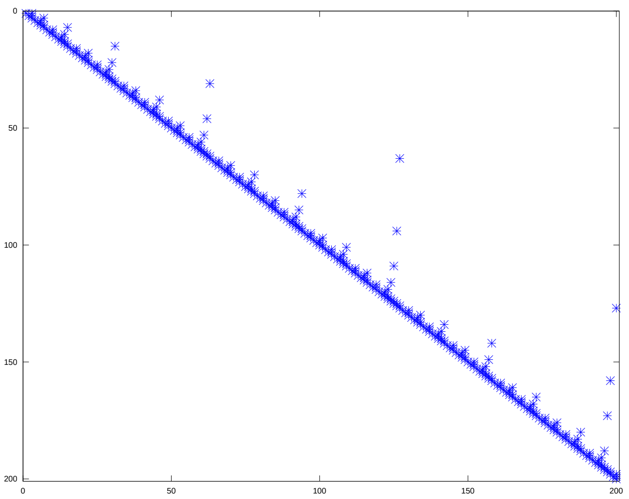

Figure 21.5: Structure of the permuted Cholesky factorization of the above matrix.

In the case of an asymmetric matrix, the appropriate sparsity preserving permutation is *colamd* and the factorization using this reordering can be visualized using the command `q = colamd(A); [l, u, p] = lu(A(:,q)); spy(l+u)`.

Finally, Octave implicitly reorders the matrix when using the div (/) and ldiv (\) operators, and so no the user does not need to explicitly reorder the matrix to maximize performance.

`p = amd (s)` [Loadable Function]
`p = amd (s, opts)` [Loadable Function]

Returns the approximate minimum degree permutation of a matrix. This permutation such that the Cholesky factorization of `s (p, p)` tends to be sparser than the Cholesky factorization of *s* itself. `amd` is typically faster than `symamd` but serves a similar purpose.

The optional parameter *opts* is a structure that controls the behavior of `amd`. The fields of these structure are

opts.dense
: Determines what `amd` considers to be a dense row or column of the input matrix. Rows or columns with more than `max(16, (dense * sqrt (n)` entries, where *n* is the order of the matrix *s*, are ignored by `amd` during the calculation of the permutation The value of dense must be a positive scalar and its default value is 10.0

opts.aggressive
: If this value is a non zero scalar, then `amd` performs aggressive absorption. The default is not to perform aggressive absorption.

The author of the code itself is Timothy A. Davis (davis@cise.ufl.edu), University of Florida (see `http://www.cise.ufl.edu/research/sparse/amd`).

See also: [symamd], page 362, [colamd], page 360.

p = ccolamd (*s*) [Loadable Function]
p = ccolamd (*s*, *knobs*) [Loadable Function]
p = ccolamd (*s*, *knobs*, *cmember*) [Loadable Function]
[*p*, *stats*] = ccolamd (...) [Loadable Function]

Constrained column approximate minimum degree permutation. *p* = ccolamd (*s*) returns the column approximate minimum degree permutation vector for the sparse matrix *s*. For a non-symmetric matrix *s*, *s* (:, *p*) tends to have sparser LU factors than *s*. chol (*s* (:, *p*)' * *s* (:, *p*)) also tends to be sparser than chol (*s*' * *s*). *p* = ccolamd (*s*, 1) optimizes the ordering for lu (*s* (:, *p*)). The ordering is followed by a column elimination tree post-ordering.

knobs is an optional one- to five-element input vector, with a default value of [0 10 10 1 0] if not present or empty. Entries not present are set to their defaults.

knobs(1) if nonzero, the ordering is optimized for lu (S (:, p)). It will be a poor ordering for chol (*s* (:, *p*)' * *s* (:, *p*)). This is the most important knob for ccolamd.

knob(2) if *s* is m-by-n, rows with more than max (16, *knobs* (2) * sqrt (n)) entries are ignored.

knob(3) columns with more than max (16, *knobs* (3) * sqrt (min (*m*, *n*))) entries are ignored and ordered last in the output permutation (subject to the cmember constraints).

knob(4) if nonzero, aggressive absorption is performed.

knob(5) if nonzero, statistics and knobs are printed.

cmember is an optional vector of length n. It defines the constraints on the column ordering. If *cmember* (j) = *c*, then column *j* is in constraint set *c* (*c* must be in the range 1 to *n*). In the output permutation *p*, all columns in set 1 appear first, followed by all columns in set 2, and so on. *cmember* = ones(1,n) if not present or empty. ccolamd (*s*, [], 1 : *n*) returns 1 : *n*

p = ccolamd (*s*) is about the same as *p* = colamd (*s*). *knobs* and its default values differ. colamd always does aggressive absorption, and it finds an ordering suitable for both lu (*s* (:, *p*)) and chol (*S* (:, *p*)' * *s* (:, *p*)); it cannot optimize its ordering for lu (*s* (:, *p*)) to the extent that ccolamd (*s*, 1) can.

stats is an optional 20-element output vector that provides data about the ordering and the validity of the input matrix *s*. Ordering statistics are in *stats* (1 : 3). *stats* (1) and *stats* (2) are the number of dense or empty rows and columns ignored by CCOLAMD and *stats* (3) is the number of garbage collections performed on the internal data structure used by CCOLAMD (roughly of size 2.2 * nnz (*s*) + 4 * *m* + 7 * *n* integers).

stats (4 : 7) provide information if CCOLAMD was able to continue. The matrix is OK if *stats* (4) is zero, or 1 if invalid. *stats* (5) is the rightmost column index that is unsorted or contains duplicate entries, or zero if no such column exists. *stats* (6) is the last seen duplicate or out-of-order row index in the column index given by *stats* (5), or zero if no such row index exists. *stats* (7) is the number of duplicate or out-of-order row indices. *stats* (8 : 20) is always zero in the current version of CCOLAMD (reserved for future use).

The authors of the code itself are S. Larimore, T. Davis (Uni of Florida) and S. Rajamanickam in collaboration with J. Bilbert and E. Ng. Supported by the National Science Foundation (DMS-9504974, DMS-9803599, CCR-0203270), and a grant from Sandia National Lab. See `http://www.cise.ufl.edu/research/sparse` for ccolamd, csymamd, amd, colamd, symamd, and other related orderings.

See also: [colamd], page 360, [csymamd], page 361.

p = colamd (*s*) [Loadable Function]
p = colamd (*s*, *knobs*) [Loadable Function]
[*p*, *stats*] = colamd (*s*) [Loadable Function]
[*p*, *stats*] = colamd (*s*, *knobs*) [Loadable Function]

Column approximate minimum degree permutation. `p = colamd (s)` returns the column approximate minimum degree permutation vector for the sparse matrix *s*. For a non-symmetric matrix *s*, `s (:,p)` tends to have sparser LU factors than *s*. The Cholesky factorization of `s (:,p)' * s (:,p)` also tends to be sparser than that of `s' * s`.

knobs is an optional one- to three-element input vector. If *s* is m-by-n, then rows with more than `max(16,knobs(1)*sqrt(n))` entries are ignored. Columns with more than `max(16,knobs(2)*sqrt(min(m,n)))` entries are removed prior to ordering, and ordered last in the output permutation *p*. Only completely dense rows or columns are removed if `knobs (1)` and `knobs (2)` are < 0, respectively. If `knobs (3)` is nonzero, *stats* and *knobs* are printed. The default is `knobs = [10 10 0]`. Note that *knobs* differs from earlier versions of colamd

stats is an optional 20-element output vector that provides data about the ordering and the validity of the input matrix *s*. Ordering statistics are in `stats (1:3)`. `stats (1)` and `stats (2)` are the number of dense or empty rows and columns ignored by COLAMD and `stats (3)` is the number of garbage collections performed on the internal data structure used by COLAMD (roughly of size `2.2 * nnz(s) + 4 * m + 7 * n` integers).

Octave built-in functions are intended to generate valid sparse matrices, with no duplicate entries, with ascending row indices of the nonzeros in each column, with a non-negative number of entries in each column (!) and so on. If a matrix is invalid, then COLAMD may or may not be able to continue. If there are duplicate entries (a row index appears two or more times in the same column) or if the row indices in a column are out of order, then COLAMD can correct these errors by ignoring the duplicate entries and sorting each column of its internal copy of the matrix *s* (the input matrix *s* is not repaired, however). If a matrix is invalid in other ways then COLAMD cannot continue, an error message is printed, and no output arguments (*p* or *stats*) are returned. COLAMD is thus a simple way to check a sparse matrix to see if it's valid.

`stats (4:7)` provide information if COLAMD was able to continue. The matrix is OK if `stats (4)` is zero, or 1 if invalid. `stats (5)` is the rightmost column index that is unsorted or contains duplicate entries, or zero if no such column exists. `stats (6)` is the last seen duplicate or out-of-order row index in the column index given by `stats (5)`, or zero if no such row index exists. `stats (7)` is the number of duplicate

or out-of-order row indices. *stats* (8:20) is always zero in the current version of COLAMD (reserved for future use).

The ordering is followed by a column elimination tree post-ordering.

The authors of the code itself are Stefan I. Larimore and Timothy A. Davis (davis@cise.ufl.edu), University of Florida. The algorithm was developed in collaboration with John Gilbert, Xerox PARC, and Esmond Ng, Oak Ridge National Laboratory. (see http://www.cise.ufl.edu/research/sparse/colamd)

See also: [colperm], page 361, [symamd], page 362.

p = colperm (*s*) [Function File]

Returns the column permutations such that the columns of *s* (:, *p*) are ordered in terms of increase number of non-zero elements. If *s* is symmetric, then *p* is chosen such that *s* (*p*, *p*) orders the rows and columns with increasing number of non zeros elements.

p = csymamd (*s*) [Loadable Function]
p = csymamd (*s*, *knobs*) [Loadable Function]
p = csymamd (*s*, *knobs*, *cmember*) [Loadable Function]
[*p*, *stats*] = csymamd (...) [Loadable Function]

For a symmetric positive definite matrix *s*, returns the permutation vector *p* such that *s*(*p*,*p*) tends to have a sparser Cholesky factor than *s*. Sometimes csymamd works well for symmetric indefinite matrices too. The matrix *s* is assumed to be symmetric; only the strictly lower triangular part is referenced. *s* must be square. The ordering is followed by an elimination tree post-ordering.

knobs is an optional one- to three-element input vector, with a default value of [10 1 0] if present or empty. Entries not present are set to their defaults.

knobs(1) If *s* is n-by-n, then rows and columns with more than max(16,*knobs*(1)*sqrt(n)) entries are ignored, and ordered last in the output permutation (subject to the cmember constraints).

knobs(2) If nonzero, aggressive absorption is performed.

knobs(3) If nonzero, statistics and knobs are printed.

cmember is an optional vector of length n. It defines the constraints on the ordering. If *cmember*(j) = *s*, then row/column j is in constraint set *c* (*c* must be in the range 1 to n). In the output permutation *p*, rows/columns in set 1 appear first, followed by all rows/columns in set 2, and so on. *cmember* = ones(1,n) if not present or empty. csymamd(*s*,[],1:n) returns 1:n.

p = csymamd(*s*) is about the same as *p* = symamd(*s*). *knobs* and its default values differ.

stats (4:7) provide information if CCOLAMD was able to continue. The matrix is OK if *stats* (4) is zero, or 1 if invalid. *stats* (5) is the rightmost column index that is unsorted or contains duplicate entries, or zero if no such column exists. *stats* (6) is the last seen duplicate or out-of-order row index in the column index given by *stats* (5), or zero if no such row index exists. *stats* (7) is the number of duplicate or out-of-order row indices. *stats* (8:20) is always zero in the current version of CCOLAMD (reserved for future use).

The authors of the code itself are S. Larimore, T. Davis (Uni of Florida) and S. Rajamanickam in collaboration with J. Bilbert and E. Ng. Supported by the National Science Foundation (DMS-9504974, DMS-9803599, CCR-0203270), and a grant from Sandia National Lab. See `http://www.cise.ufl.edu/research/sparse` for ccolamd, csymamd, amd, colamd, symamd, and other related orderings.

See also: [symamd], page 362, [ccolamd], page 359.

p = `dmperm` (*s*) [Loadable Function]
[*p*, *q*, *r*, *s*] = `dmperm` (*s*) [Loadable Function]

Perform a Dulmage-Mendelsohn permutation on the sparse matrix *s*. With a single output argument *dmperm* performs the row permutations *p* such that `s (p,:)` has no zero elements on the diagonal.

Called with two or more output arguments, returns the row and column permutations, such that `s (p, q)` is in block triangular form. The values of *r* and *s* define the boundaries of the blocks. If *s* is square then `r == s`.

The method used is described in: A. Pothen & C.-J. Fan. Computing the block triangular form of a sparse matrix. ACM Trans. Math. Software, 16(4):303-324, 1990.

See also: [colamd], page 360, [ccolamd], page 359.

p = `symamd` (*s*) [Loadable Function]
p = `symamd` (*s*, *knobs*) [Loadable Function]
[*p*, *stats*] = `symamd` (*s*) [Loadable Function]
[*p*, *stats*] = `symamd` (*s*, *knobs*) [Loadable Function]

For a symmetric positive definite matrix *s*, returns the permutation vector p such that `s (p, p)` tends to have a sparser Cholesky factor than *s*. Sometimes SYMAMD works well for symmetric indefinite matrices too. The matrix *s* is assumed to be symmetric; only the strictly lower triangular part is referenced. *s* must be square.

knobs is an optional one- to two-element input vector. If *s* is n-by-n, then rows and columns with more than `max(16,knobs(1)*sqrt(n))` entries are removed prior to ordering, and ordered last in the output permutation *p*. No rows/columns are removed if `knobs(1) < 0`. If `knobs (2)` is nonzero, `stats` and *knobs* are printed. The default is `knobs = [10 0]`. Note that *knobs* differs from earlier versions of symamd.

stats is an optional 20-element output vector that provides data about the ordering and the validity of the input matrix *s*. Ordering statistics are in `stats (1:3)`. `stats (1) = stats (2)` is the number of dense or empty rows and columns ignored by SYMAMD and `stats (3)` is the number of garbage collections performed on the internal data structure used by SYMAMD (roughly of size `8.4 * nnz (tril (s, -1)) + 9 * n` integers).

Octave built-in functions are intended to generate valid sparse matrices, with no duplicate entries, with ascending row indices of the nonzeros in each column, with a non-negative number of entries in each column (!) and so on. If a matrix is invalid, then SYMAMD may or may not be able to continue. If there are duplicate entries (a row index appears two or more times in the same column) or if the row indices in a column are out of order, then SYMAMD can correct these errors by ignoring the duplicate entries and sorting each column of its internal copy of the matrix S (the

input matrix S is not repaired, however). If a matrix is invalid in other ways then SYMAMD cannot continue, an error message is printed, and no output arguments (*p* or *stats*) are returned. SYMAMD is thus a simple way to check a sparse matrix to see if it's valid.

`stats (4:7)` provide information if SYMAMD was able to continue. The matrix is OK if `stats (4)` is zero, or 1 if invalid. `stats (5)` is the rightmost column index that is unsorted or contains duplicate entries, or zero if no such column exists. `stats (6)` is the last seen duplicate or out-of-order row index in the column index given by `stats (5)`, or zero if no such row index exists. `stats (7)` is the number of duplicate or out-of-order row indices. `stats (8:20)` is always zero in the current version of SYMAMD (reserved for future use).

The ordering is followed by a column elimination tree post-ordering.

The authors of the code itself are Stefan I. Larimore and Timothy A. Davis (davis@cise.ufl.edu), University of Florida. The algorithm was developed in collaboration with John Gilbert, Xerox PARC, and Esmond Ng, Oak Ridge National Laboratory. (see `http://www.cise.ufl.edu/research/sparse/colamd`)

See also: [colperm], page 361, [colamd], page 360.

`p = symrcm (S)` [Loadable Function]

Symmetric reverse Cuthill-McKee permutation of *S*. Return a permutation vector *p* such that `S (p, p)` tends to have its diagonal elements closer to the diagonal than *S*. This is a good preordering for LU or Cholesky factorization of matrices that come from 'long, skinny' problems. It works for both symmetric and asymmetric *S*.

The algorithm represents a heuristic approach to the NP-complete bandwidth minimization problem. The implementation is based in the descriptions found in

E. Cuthill, J. McKee: Reducing the Bandwidth of Sparse Symmetric Matrices. Proceedings of the 24th ACM National Conference, 157–172 1969, Brandon Press, New Jersey.

Alan George, Joseph W. H. Liu: Computer Solution of Large Sparse Positive Definite Systems, Prentice Hall Series in Computational Mathematics, ISBN 0-13-165274-5, 1981.

See also: [colperm], page 361, [colamd], page 360, [symamd], page 362.

21.2 Linear Algebra on Sparse Matrices

Octave includes a polymorphic solver for sparse matrices, where the exact solver used to factorize the matrix, depends on the properties of the sparse matrix itself. Generally, the cost of determining the matrix type is small relative to the cost of factorizing the matrix itself, but in any case the matrix type is cached once it is calculated, so that it is not re-determined each time it is used in a linear equation.

The selection tree for how the linear equation is solve is

1. If the matrix is diagonal, solve directly and goto 8
2. If the matrix is a permuted diagonal, solve directly taking into account the permutations. Goto 8

3. If the matrix is square, banded and if the band density is less than that given by `spparms ("bandden")` continue, else goto 4.
   a. If the matrix is tridiagonal and the right-hand side is not sparse continue, else goto 3b.
      1. If the matrix is hermitian, with a positive real diagonal, attempt Cholesky factorization using LAPACK xPTSV.
      2. If the above failed or the matrix is not hermitian with a positive real diagonal use Gaussian elimination with pivoting using LAPACK xGTSV, and goto 8.
   b. If the matrix is hermitian with a positive real diagonal, attempt Cholesky factorization using LAPACK xPBTRF.
   c. if the above failed or the matrix is not hermitian with a positive real diagonal use Gaussian elimination with pivoting using LAPACK xGBTRF, and goto 8.
4. If the matrix is upper or lower triangular perform a sparse forward or backward substitution, and goto 8
5. If the matrix is a upper triangular matrix with column permutations or lower triangular matrix with row permutations, perform a sparse forward or backward substitution, and goto 8
6. If the matrix is square, hermitian with a real positive diagonal, attempt sparse Cholesky factorization using CHOLMOD.
7. If the sparse Cholesky factorization failed or the matrix is not hermitian with a real positive diagonal, and the matrix is square, factorize using UMFPACK.
8. If the matrix is not square, or any of the previous solvers flags a singular or near singular matrix, find a minimum norm solution using CXSPARSE[2].

The band density is defined as the number of non-zero values in the matrix divided by the number of non-zero values in the matrix. The banded matrix solvers can be entirely disabled by using *spparms* to set `bandden` to 1 (i.e., `spparms ("bandden", 1)`).

The QR solver factorizes the problem with a Dulmage-Mendelsohn, to separate the problem into blocks that can be treated as over-determined, multiple well determined blocks, and a final over-determined block. For matrices with blocks of strongly connected nodes this is a big win as LU decomposition can be used for many blocks. It also significantly improves the chance of finding a solution to over-determined problems rather than just returning a vector of *NaN*'s.

All of the solvers above, can calculate an estimate of the condition number. This can be used to detect numerical stability problems in the solution and force a minimum norm solution to be used. However, for narrow banded, triangular or diagonal matrices, the cost of calculating the condition number is significant, and can in fact exceed the cost of factoring the matrix. Therefore the condition number is not calculated in these cases, and Octave relies on simpler techniques to detect singular matrices or the underlying LAPACK code in the case of banded matrices.

The user can force the type of the matrix with the `matrix_type` function. This overcomes the cost of discovering the type of the matrix. However, it should be noted that identifying

[2] The CHOLMOD, UMFPACK and CXSPARSE packages were written by Tim Davis and are available at http://www.cise.ufl.edu/research/sparse/

the type of the matrix incorrectly will lead to unpredictable results, and so `matrix_type` should be used with care.

`[n, c] = normest (a, tol)` [Function File]

Estimate the 2-norm of the matrix *a* using a power series analysis. This is typically used for large matrices, where the cost of calculating the `norm (a)` is prohibitive and an approximation to the 2-norm is acceptable.

tol is the tolerance to which the 2-norm is calculated. By default *tol* is 1e-6. *c* returns the number of iterations needed for `normest` to converge.

`[est, v, w, iter] = onenormest (a, t)` [Function File]
`[est, v, w, iter] = onenormest (apply, apply_t, n, t)` [Function File]

Apply Higham and Tisseur's randomized block 1-norm estimator to matrix *a* using *t* test vectors. If *t* exceeds 5, then only 5 test vectors are used.

If the matrix is not explicit, e.g., when estimating the norm of `inv (A)` given an LU factorization, `onenormest` applies *A* and its conjugate transpose through a pair of functions *apply* and *apply_t*, respectively, to a dense matrix of size *n* by *t*. The implicit version requires an explicit dimension *n*.

Returns the norm estimate *est*, two vectors *v* and *w* related by norm `(w, 1) = est * norm (v, 1)`, and the number of iterations *iter*. The number of iterations is limited to 10 and is at least 2.

References:

- Nicholas J. Higham and Franoise Tisseur, "A Block Algorithm for Matrix 1-Norm Estimation, with an Application to 1-Norm Pseudospectra." SIMAX vol 21, no 4, pp 1185-1201. http://dx.doi.org/10.1137/S0895479899356080
- Nicholas J. Higham and Franoise Tisseur, "A Block Algorithm for Matrix 1-Norm Estimation, with an Application to 1-Norm Pseudospectra." http://citeseer.ist.psu.edu/223007.html

See also: [condest], page 365, [norm], page 318, [cond], page 316.

`[est, v] = condest (a, t)` [Function File]
`[est, v] = condest (a, solve, solve_t, t)` [Function File]
`[est, v] = condest (apply, apply_t, solve, solve_t, n, t)` [Function File]

Estimate the 1-norm condition number of a matrix *A* using *t* test vectors using a randomized 1-norm estimator. If *t* exceeds 5, then only 5 test vectors are used.

If the matrix is not explicit, e.g., when estimating the condition number of *a* given an LU factorization, `condest` uses the following functions:

apply `A*x` for a matrix `x` of size *n* by *t*.

apply_t `A'*x` for a matrix `x` of size *n* by *t*.

solve `A \ b` for a matrix `b` of size *n* by *t*.

solve_t `A' \ b` for a matrix `b` of size *n* by *t*.

The implicit version requires an explicit dimension *n*.

`condest` uses a randomized algorithm to approximate the 1-norms.

condest returns the 1-norm condition estimate *est* and a vector *v* satisfying norm (A*v, 1) == norm (A, 1) * norm (*v*, 1) / *est*. When *est* is large, *v* is an approximate null vector.

References:

- Nicholas J. Higham and Franoise Tisseur, "A Block Algorithm for Matrix 1-Norm Estimation, with an Application to 1-Norm Pseudospectra." SIMAX vol 21, no 4, pp 1185-1201. http://dx.doi.org/10.1137/S0895479899356080
- Nicholas J. Higham and Franoise Tisseur, "A Block Algorithm for Matrix 1-Norm Estimation, with an Application to 1-Norm Pseudospectra." http://citeseer.ist.psu.edu/223007.html

See also: [cond], page 316, [norm], page 318, [onenormest], page 365.

spparms () [Loadable Function]
vals = spparms () [Loadable Function]
[*keys*, *vals*] = spparms () [Loadable Function]
val = spparms (*key*) [Loadable Function]
spparms (*vals*) [Loadable Function]
spparms (*'defaults'*) [Loadable Function]
spparms (*'tight'*) [Loadable Function]
spparms (*key*, *val*) [Loadable Function]

Sets or displays the parameters used by the sparse solvers and factorization functions. The first four calls above get information about the current settings, while the others change the current settings. The parameters are stored as pairs of keys and values, where the values are all floats and the keys are one of the following strings:

spumoni Printing level of debugging information of the solvers (default 0)

ths_rel Included for compatibility. Not used. (default 1)

ths_abs Included for compatibility. Not used. (default 1)

exact_d Included for compatibility. Not used. (default 0)

supernd Included for compatibility. Not used. (default 3)

rreduce Included for compatibility. Not used. (default 3)

wh_frac Included for compatibility. Not used. (default 0.5)

autommd Flag whether the LU/QR and the '\' and '/' operators will automatically use the sparsity preserving mmd functions (default 1)

autoamd Flag whether the LU and the '\' and '/' operators will automatically use the sparsity preserving amd functions (default 1)

piv_tol The pivot tolerance of the UMFPACK solvers (default 0.1)

sym_tol The pivot tolerance of the UMFPACK symmetric solvers (default 0.001)

bandden The density of non-zero elements in a banded matrix before it is treated by the LAPACK banded solvers (default 0.5)

umfpack Flag whether the UMFPACK or mmd solvers are used for the LU, '\' and '/' operations (default 1)

The value of individual keys can be set with `spparms (key, val)`. The default values can be restored with the special keyword 'defaults'. The special keyword 'tight' can be used to set the mmd solvers to attempt for a sparser solution at the potential cost of longer running time.

p = `sprank (`*s*`)` [Loadable Function]

Calculates the structural rank of a sparse matrix *s*. Note that only the structure of the matrix is used in this calculation based on a Dulmage-Mendelsohn permutation to block triangular form. As such the numerical rank of the matrix *s* is bounded by `sprank (s) >= rank (s)`. Ignoring floating point errors `sprank (s) == rank (s)`.

See also: [dmperm], page 362.

`[`*count*`,` *h*`,` *parent*`,` *post*`,` *r*`] = symbfact (`*s*, *typ*, *mode*`)` [Loadable Function]

Performs a symbolic factorization analysis on the sparse matrix *s*. Where

s — *s* is a complex or real sparse matrix.

typ — Is the type of the factorization and can be one of

- `sym` — Factorize *s*. This is the default.
- `col` — Factorize `s' * s`.
- `row` — Factorize `s * s'`.
- `lo` — Factorize `s'`

mode — The default is to return the Cholesky factorization for *r*, and if *mode* is 'L', the conjugate transpose of the Cholesky factorization is returned. The conjugate transpose version is faster and uses less memory, but returns the same values for *count*, *h*, *parent* and *post* outputs.

The output variables are

count — The row counts of the Cholesky factorization as determined by *typ*.

h — The height of the elimination tree.

parent — The elimination tree itself.

post — A sparse boolean matrix whose structure is that of the Cholesky factorization as determined by *typ*.

For non square matrices, the user can also utilize the `spaugment` function to find a least squares solution to a linear equation.

s = `spaugment (`*a*, *c*`)` [Function File]

Creates the augmented matrix of *a*. This is given by

```
[c * eye(m, m),a; a', zeros(n,
n)]
```

This is related to the least squares solution of `a \\ b`, by

```
s * [ r / c; x] = [b, zeros(n,
columns(b)]
```

where *r* is the residual error

```
r = b - a * x
```

As the matrix *s* is symmetric indefinite it can be factorized with `lu`, and the minimum norm solution can therefore be found without the need for a `qr` factorization. As the residual error will be `zeros (m, m)` for under determined problems, and example can be

```
m = 11; n = 10; mn = max(m ,n);
a = spdiags ([ones(mn,1), 10*ones(mn,1), -ones(mn,1)],
             [-1, 0, 1], m, n);
x0 = a \ ones (m,1);
s = spaugment (a);
[L, U, P, Q] = lu (s);
x1 = Q * (U \ (L \ (P  * [ones(m,1); zeros(n,1)])));
x1 = x1(end - n + 1 : end);
```

To find the solution of an overdetermined problem needs an estimate of the residual error *r* and so it is more complex to formulate a minimum norm solution using the `spaugment` function.

In general the left division operator is more stable and faster than using the `spaugment` function.

Finally, the function `eigs` can be used to calculate a limited number of eigenvalues and eigenvectors based on a selection criteria and likewise for `svds` which calculates a limited number of singular values and vectors.

d = *eigs* (a) [Loadable Function]
d = *eigs* (a, *k*) [Loadable Function]
d = *eigs* (a, *k*, *sigma*) [Loadable Function]
d = *eigs* (a, *k*, *sigma*,*opts*) [Loadable Function]
d = *eigs* (a, *b*) [Loadable Function]
d = *eigs* (a, *b*, *k*) [Loadable Function]
d = *eigs* (a, *b*, *k*, *sigma*) [Loadable Function]
d = *eigs* (a, *b*, *k*, *sigma*, *opts*) [Loadable Function]
d = *eigs* (af, *n*) [Loadable Function]
d = *eigs* (af, *n*, *b*) [Loadable Function]
d = *eigs* (af, *n*, *k*) [Loadable Function]
d = *eigs* (af, *n*, *b*, *k*) [Loadable Function]
d = *eigs* (af, *n*, *k*, *sigma*) [Loadable Function]
d = *eigs* (af, *n*, *b*, *k*, *sigma*) [Loadable Function]
d = *eigs* (af, *n*, *k*, *sigma*, *opts*) [Loadable Function]
d = *eigs* (af, *n*, *b*, *k*, *sigma*, *opts*) [Loadable Function]
[*v*, *d*] = *eigs* (a, ...) [Loadable Function]
[*v*, *d*] = *eigs* (af, *n*, ...) [Loadable Function]
[*v*, *d*, *flag*] = *eigs* (a, ...) [Loadable Function]
[*v*, *d*, *flag*] = *eigs* (af, *n*, ...) [Loadable Function]

Calculate a limited number of eigenvalues and eigenvectors of *a*, based on a selection criteria. The number eigenvalues and eigenvectors to calculate is given by *k* whose default value is 6.

By default `eigs` solve the equation $A\nu = \lambda\nu$, where λ is a scalar representing one of the eigenvalues, and ν is the corresponding eigenvector. If given the positive definite matrix B then `eigs` solves the general eigenvalue equation $A\nu = \lambda B\nu$.

The argument *sigma* determines which eigenvalues are returned. *sigma* can be either a scalar or a string. When *sigma* is a scalar, the *k* eigenvalues closest to *sigma* are returned. If *sigma* is a string, it must have one of the values

'lm' Largest magnitude (default).

'sm' Smallest magnitude.

'la' Largest Algebraic (valid only for real symmetric problems).

'sa' Smallest Algebraic (valid only for real symmetric problems).

'be' Both ends, with one more from the high-end if *k* is odd (valid only for real symmetric problems).

'lr' Largest real part (valid only for complex or unsymmetric problems).

'sr' Smallest real part (valid only for complex or unsymmetric problems).

'li' Largest imaginary part (valid only for complex or unsymmetric problems).

'si' Smallest imaginary part (valid only for complex or unsymmetric problems).

If *opts* is given, it is a structure defining some of the options that `eigs` should use. The fields of the structure *opts* are

`issym` If *af* is given, then flags whether the function *af* defines a symmetric problem. It is ignored if *a* is given. The default is false.

`isreal` If *af* is given, then flags whether the function *af* defines a real problem. It is ignored if *a* is given. The default is true.

`tol` Defines the required convergence tolerance, given as `tol * norm (A)`. The default is `eps`.

`maxit` The maximum number of iterations. The default is 300.

`p` The number of Lanzcos basis vectors to use. More vectors will result in faster convergence, but a larger amount of memory. The optimal value of 'p' is problem dependent and should be in the range *k* to *n*. The default value is `2 * k`.

`v0` The starting vector for the computation. The default is to have ARPACK randomly generate a starting vector.

`disp` The level of diagnostic printout. If `disp` is 0 then there is no printout. The default value is 1.

`cholB` Flag if `chol (b)` is passed rather than *b*. The default is false.

`permB` The permutation vector of the Cholesky factorization of *b* if `cholB` is true. That is `chol ( b (permB, permB))`. The default is `1:n`.

It is also possible to represent *a* by a function denoted *af*. *af* must be followed by a scalar argument *n* defining the length of the vector argument accepted by *af*. *af* can be passed either as an inline function, function handle or as a string. In the case where *af* is passed as a string, the name of the string defines the function to use.

af is a function of the form `function y = af (x), y = ...; endfunction`, where the required return value of *af* is determined by the value of *sigma*, and are

`A * x` If *sigma* is not given or is a string other than 'sm'.

`A \ x` If *sigma* is 'sm'.

`(A - sigma * I) \ x`
for standard eigenvalue problem, where `I` is the identity matrix of the same size as `A`. If *sigma* is zero, this reduces the `A \ x`.

`(A - sigma * B) \ x`
for the general eigenvalue problem.

The return arguments of `eigs` depends on the number of return arguments. With a single return argument, a vector *d* of length *k* is returned, represent the *k* eigenvalues that have been found. With two return arguments, *v* is a *n*-by-*k* matrix whose columns are the *k* eigenvectors corresponding to the returned eigenvalues. The eigenvalues themselves are then returned in *d* in the form of a *n*-by-*k* matrix, where the elements on the diagonal are the eigenvalues.

Given a third return argument *flag*, `eigs` also returns the status of the convergence. If *flag* is 0, then all eigenvalues have converged, otherwise not.

This function is based on the ARPACK package, written by R Lehoucq, K Maschhoff, D Sorensen and C Yang. For more information see http://www.caam.rice.edu/software/ARPACK/.

See also: [eig], page 316, [svds], page 370.

`s = svds (a)` [Function File]
`s = svds (a, k)` [Function File]
`s = svds (a, k, sigma)` [Function File]
`s = svds (a, k, sigma, opts)` [Function File]
`[u, s, v, flag] = svds (...)` [Function File]

Find a few singular values of the matrix *a*. The singular values are calculated using

```
[m, n] = size(a)
s = eigs([sparse(m, m), a; ...
                    a', sparse(n, n)])
```

The eigenvalues returned by `eigs` correspond to the singular values of *a*. The number of singular values to calculate is given by *k*, whose default value is 6.

The argument *sigma* can be used to specify which singular values to find. *sigma* can be either the string 'L', the default, in which case the largest singular values of *a* are found. Otherwise *sigma* should be a real scalar, in which case the singular values closest to *sigma* are found. Note that for relatively small values of *sigma*, there is the chance that the requested number of singular values are not returned. In that case *sigma* should be increased.

If *opts* is given, then it is a structure that defines options that `svds` will pass to *eigs*. The possible fields of this structure are therefore determined by `eigs`. By default three fields of this structure are set by `svds`.

`tol` The required convergence tolerance for the singular values. `eigs` is passed *tol* divided by `sqrt(2)`. The default value is 1e-10.

`maxit` The maximum number of iterations. The default is 300.

`disp` The level of diagnostic printout. If `disp` is 0 then there is no printout. The default value is 0.

If more than one output argument is given, then `svds` also calculates the left and right singular vectors of *a*. *flag* is used to signal the convergence of `svds`. If `svds` converges to the desired tolerance, then *flag* given by

```
norm (a * v - u * s, 1) <= ...
        tol * norm (a, 1)
```

will be zero.

See also: [eigs], page 368.

21.3 Iterative Techniques applied to sparse matrices

The left division `\` and right division `/` operators, discussed in the previous section, use direct solvers to resolve a linear equation of the form `x = A \ b` or `x = b / A`. Octave equally includes a number of functions to solve sparse linear equations using iterative techniques.

x = pcg (*a*, *b*, *tol*, *maxit*, *m1*, *m2*, *x0*, . . .) [Function File]
[*x*, *flag*, *relres*, *iter*, *resvec*, *eigest*] = pcg (. . .) [Function File]

Solves the linear system of equations `a * x = b` by means of the Preconditioned Conjugate Gradient iterative method. The input arguments are

- *a* can be either a square (preferably sparse) matrix or a function handle, inline function or string containing the name of a function which computes `a * x`. In principle *a* should be symmetric and positive definite; if `pcg` finds *a* to not be positive definite, you will get a warning message and the *flag* output parameter will be set.
- *b* is the right hand side vector.
- *tol* is the required relative tolerance for the residual error, `b - a * x`. The iteration stops if `norm (b - a * x) <= tol * norm (b - a * x0)`. If *tol* is empty or is omitted, the function sets `tol = 1e-6` by default.
- *maxit* is the maximum allowable number of iterations; if `[]` is supplied for `maxit`, or `pcg` has less arguments, a default value equal to 20 is used.
- $m = m1 * m2$ is the (left) preconditioning matrix, so that the iteration is (theoretically) equivalent to solving by `pcg` `P * x = m \ b`, with `P = m \ a`. Note that a proper choice of the preconditioner may dramatically improve the overall performance of the method. Instead of matrices *m1* and *m2*, the user may pass two functions which return the results of applying the inverse of *m1* and *m2* to a vector (usually this is the preferred way of using the preconditioner). If `[]`

is supplied for *m1*, or *m1* is omitted, no preconditioning is applied. If *m2* is omitted, *m* = *m1* will be used as preconditioner.

- *x0* is the initial guess. If *x0* is empty or omitted, the function sets *x0* to a zero vector by default.

The arguments which follow *x0* are treated as parameters, and passed in a proper way to any of the functions (*a* or *m*) which are passed to `pcg`. See the examples below for further details. The output arguments are

- *x* is the computed approximation to the solution of `a * x = b`.
- *flag* reports on the convergence. `flag = 0` means the solution converged and the tolerance criterion given by *tol* is satisfied. `flag = 1` means that the *maxit* limit for the iteration count was reached. `flag = 3` reports that the (preconditioned) matrix was found not positive definite.
- *relres* is the ratio of the final residual to its initial value, measured in the Euclidean norm.
- *iter* is the actual number of iterations performed.
- *resvec* describes the convergence history of the method. `resvec (i,1)` is the Euclidean norm of the residual, and `resvec (i,2)` is the preconditioned residual norm, after the (*i*-1)-th iteration, `i = 1, 2, ..., iter+1`. The preconditioned residual norm is defined as `norm (r) ^ 2 = r' * (m \ r)` where `r = b - a * x`, see also the description of *m*. If *eigest* is not required, only `resvec (:,1)` is returned.
- *eigest* returns the estimate for the smallest `eigest (1)` and largest `eigest (2)` eigenvalues of the preconditioned matrix `P = m \ a`. In particular, if no preconditioning is used, the estimates for the extreme eigenvalues of *a* are returned. `eigest (1)` is an overestimate and `eigest (2)` is an underestimate, so that `eigest (2) / eigest (1)` is a lower bound for `cond (P, 2)`, which nevertheless in the limit should theoretically be equal to the actual value of the condition number. The method which computes *eigest* works only for symmetric positive definite *a* and *m*, and the user is responsible for verifying this assumption.

Let us consider a trivial problem with a diagonal matrix (we exploit the sparsity of A)

```
n = 10;
a = diag (sparse (1:n));
b = rand (n, 1);
     [l, u, p, q] = luinc (a, 1.e-3);
```

EXAMPLE 1: Simplest use of `pcg`

```
x = pcg(A,b)
```

EXAMPLE 2: `pcg` with a function which computes `a * x`

```
function y = apply_a (x)
  y = [1:N]'.*x;
endfunction

x = pcg ("apply_a", b)
```

EXAMPLE 3: `pcg` with a preconditioner: *l* * *u*

```
x = pcg (a, b, 1.e-6, 500, l*u);
```

Example 4: pcg with a preconditioner: *l* * *u*. Faster than Example 3 since lower and upper triangular matrices are easier to invert

```
x = pcg (a, b, 1.e-6, 500, l, u);
```

Example 5: Preconditioned iteration, with full diagnostics. The preconditioner (quite strange, because even the original matrix *a* is trivial) is defined as a function

```
function y = apply_m (x)
  k = floor (length (x) - 2);
  y = x;
  y(1:k) = x(1:k)./[1:k]';
endfunction

[x, flag, relres, iter, resvec, eigest] = ...
                   pcg (a, b, [], [], "apply_m");
semilogy (1:iter+1, resvec);
```

Example 6: Finally, a preconditioner which depends on a parameter *k*.

```
function y = apply_M (x, varargin)
K = varargin{1};
y = x;
y(1:K) = x(1:K)./[1:K]';
endfunction

[x, flag, relres, iter, resvec, eigest] = ...
     pcg (A, b, [], [], "apply_m", [], [], 3)
```

References

[1] C.T.Kelley, 'Iterative methods for linear and nonlinear equations', SIAM, 1995 (the base PCG algorithm)

[2] Y.Saad, 'Iterative methods for sparse linear systems', PWS 1996 (condition number estimate from PCG) Revised version of this book is available online at http://www-users.cs.umn.edu/~saad/books.html

See also: [sparse], page 348, [pcr], page 373.

x = pcr (*a*, *b*, *tol*, *maxit*, *m*, *x0*, . . .) [Function File]
[*x*, *flag*, *relres*, *iter*, *resvec*] = pcr (. . .) [Function File]

Solves the linear system of equations `a * x = b` by means of the Preconditioned Conjugate Residuals iterative method. The input arguments are

- *a* can be either a square (preferably sparse) matrix or a function handle, inline function or string containing the name of a function which computes `a * x`. In principle *a* should be symmetric and non-singular; if `pcr` finds *a* to be numerically singular, you will get a warning message and the *flag* output parameter will be set.
- *b* is the right hand side vector.
- *tol* is the required relative tolerance for the residual error, `b - a * x`. The iteration stops if `norm (b - a * x) <= tol * norm (b - a * x0)`. If *tol* is empty or is omitted, the function sets `tol = 1e-6` by default.

- *maxit* is the maximum allowable number of iterations; if `[]` is supplied for `maxit`, or `pcr` has less arguments, a default value equal to 20 is used.
- *m* is the (left) preconditioning matrix, so that the iteration is (theoretically) equivalent to solving by `pcr` `P * x = m \ b`, with `P = m \ a`. Note that a proper choice of the preconditioner may dramatically improve the overall performance of the method. Instead of matrix *m*, the user may pass a function which returns the results of applying the inverse of *m* to a vector (usually this is the preferred way of using the preconditioner). If `[]` is supplied for *m*, or *m* is omitted, no preconditioning is applied.
- *x0* is the initial guess. If *x0* is empty or omitted, the function sets *x0* to a zero vector by default.

The arguments which follow *x0* are treated as parameters, and passed in a proper way to any of the functions (*a* or *m*) which are passed to `pcr`. See the examples below for further details. The output arguments are

- *x* is the computed approximation to the solution of `a * x = b`.
- *flag* reports on the convergence. `flag = 0` means the solution converged and the tolerance criterion given by *tol* is satisfied. `flag = 1` means that the *maxit* limit for the iteration count was reached. `flag = 3` reports t `pcr` breakdown, see [1] for details.
- *relres* is the ratio of the final residual to its initial value, measured in the Euclidean norm.
- *iter* is the actual number of iterations performed.
- *resvec* describes the convergence history of the method, so that `resvec (i)` contains the Euclidean norms of the residual after the (*i*-1)-th iteration, `i = 1,2, ..., iter+1`.

Let us consider a trivial problem with a diagonal matrix (we exploit the sparsity of A)

```
n = 10;
a = sparse (diag (1:n));
b = rand (N, 1);
```

Example 1: Simplest use of `pcr`

```
x = pcr(A, b)
```

Example 2: `pcr` with a function which computes `a * x`.

```
function y = apply_a (x)
  y = [1:10]'.*x;
endfunction

x = pcr ("apply_a", b)
```

Example 3: Preconditioned iteration, with full diagnostics. The preconditioner (quite strange, because even the original matrix *a* is trivial) is defined as a function

```
function y = apply_m (x)
  k = floor (length(x)-2);
  y = x;
  y(1:k) = x(1:k)./[1:k]';
endfunction

[x, flag, relres, iter, resvec] = ...
                   pcr (a, b, [], [], "apply_m")
semilogy([1:iter+1], resvec);
```

EXAMPLE 4: Finally, a preconditioner which depends on a parameter *k*.

```
function y = apply_m (x, varargin)
  k = varargin{1};
  y = x; y(1:k) = x(1:k)./[1:k]';
endfunction

[x, flag, relres, iter, resvec] = ...
                   pcr (a, b, [], [], "apply_m"', [], 3)
```

REFERENCES

[1] W. Hackbusch, "Iterative Solution of Large Sparse Systems of Equations", section 9.5.4; Springer, 1994

See also: [sparse], page 348, [pcg], page 371.

The speed with which an iterative solver converges to a solution can be accelerated with the use of a pre-conditioning matrix *M*. In this case the linear equation `M^-1 * x = M^-1 * A \ b` is solved instead. Typical pre-conditioning matrices are partial factorizations of the original matrix.

`[l, u, p, q] = luinc (a, '0')` [Loadable Function]
`[l, u, p, q] = luinc (a, droptol)` [Loadable Function]
`[l, u, p, q] = luinc (a, opts)` [Loadable Function]

Produce the incomplete LU factorization of the sparse matrix *a*. Two types of incomplete factorization are possible, and the type is determined by the second argument to *luinc*.

Called with a second argument of '0', the zero-level incomplete LU factorization is produced. This creates a factorization of *a* where the position of the non-zero arguments correspond to the same positions as in the matrix *a*.

Alternatively, the fill-in of the incomplete LU factorization can be controlled through the variable *droptol* or the structure *opts*. The UMFPACK multifrontal factorization code by Tim A. Davis is used for the incomplete LU factorization, (availability `http://www.cise.ufl.edu/research/sparse/umfpack/`)

droptol determines the values below which the values in the LU factorization are dropped and replaced by zero. It must be a positive scalar, and any values in the factorization whose absolute value are less than this value are dropped, expect if leaving them increase the sparsity of the matrix. Setting *droptol* to zero results in a complete LU factorization which is the default.

opts is a structure containing one or more of the fields

`droptol` The drop tolerance as above. If *opts* only contains `droptol` then this is equivalent to using the variable *droptol*.

`milu` A logical variable flagging whether to use the modified incomplete LU factorization. In the case that `milu` is true, the dropped values are subtracted from the diagonal of the matrix U of the factorization. The default is `false`.

`udiag` A logical variable that flags whether zero elements on the diagonal of U should be replaced with *droptol* to attempt to avoid singular factors. The default is `false`.

`thresh` Defines the pivot threshold in the interval [0,1]. Values outside that range are ignored.

All other fields in *opts* are ignored. The outputs from *luinc* are the same as for *lu*.

Given the string argument 'vector', *luinc* returns the values of *p q* as vector values.

See also: [sparse], page 348, [lu], page 322.

21.4 Real Life Example of the use of Sparse Matrices

A common application for sparse matrices is in the solution of Finite Element Models. Finite element models allow numerical solution of partial differential equations that do not have closed form solutions, typically because of the complex shape of the domain.

In order to motivate this application, we consider the boundary value Laplace equation. This system can model scalar potential fields, such as heat or electrical potential. Given a medium Ω with boundary $\partial\Omega$. At all points on the $\partial\Omega$ the boundary conditions are known, and we wish to calculate the potential in Ω . Boundary conditions may specify the potential (Dirichlet boundary condition), its normal derivative across the boundary (Neumann boundary condition), or a weighted sum of the potential and its derivative (Cauchy boundary condition).

In a thermal model, we want to calculate the temperature in Ω and know the boundary temperature (Dirichlet condition) or heat flux (from which we can calculate the Neumann condition by dividing by the thermal conductivity at the boundary). Similarly, in an electrical model, we want to calculate the voltage in Ω and know the boundary voltage (Dirichlet) or current (Neumann condition after diving by the electrical conductivity). In an electrical model, it is common for much of the boundary to be electrically isolated; this is a Neumann boundary condition with the current equal to zero.

The simplest finite element models will divide Ω into simplexes (triangles in 2D, pyramids in 3D). We take as an 3D example a cylindrical liquid filled tank with a small non-conductive ball from the EIDORS project[3]. This is model is designed to reflect an application of electrical impedance tomography, where current patterns are applied to such a tank in order to image the internal conductivity distribution. In order to describe the FEM geometry, we have a matrix of vertices `nodes` and simplices `elems`.

[3] EIDORS - Electrical Impedance Tomography and Diffuse optical Tomography Reconstruction Software `http://eidors3d.sourceforge.net`

The following example creates a simple rectangular 2D electrically conductive medium with 10 V and 20 V imposed on opposite sides (Dirichlet boundary conditions). All other edges are electrically isolated.

```
node_y= [1;1.2;1.5;1.8;2]*ones(1,11);
node_x= ones(5,1)*[1,1.05,1.1,1.2, ...
          1.3,1.5,1.7,1.8,1.9,1.95,2];
nodes= [node_x(:), node_y(:)];

[h,w]= size(node_x);
elems= [];
for idx= 1:w-1
  widx= (idx-1)*h;
  elems= [elems; ...
    widx+[(1:h-1);(2:h);h+(1:h-1)]'; ...
    widx+[(2:h);h+(2:h);h+(1:h-1)]' ];
endfor

E= size(elems,1); # No. of simplices
N= size(nodes,1); # No. of vertices
D= size(elems,2); # dimensions+1
```

This creates a N-by-2 matrix `nodes` and a E-by-3 matrix `elems` with values, which define finite element triangles:

```
nodes(1:7,:)'
  1.00 1.00 1.00 1.00 1.00 1.05 1.05 ...
  1.00 1.20 1.50 1.80 2.00 1.00 1.20 ...

elems(1:7,:)'
  1    2    3    4    2    3    4 ...
  2    3    4    5    7    8    9 ...
  6    7    8    9    6    7    8 ...
```

Using a first order FEM, we approximate the electrical conductivity distribution in Ω as constant on each simplex (represented by the vector `conductivity`). Based on the finite element geometry, we first calculate a system (or stiffness) matrix for each simplex (represented as 3-by-3 elements on the diagonal of the element-wise system matrix `SE`. Based on `SE` and a N-by-DE connectivity matrix `C`, representing the connections between simplices and vertices, the global connectivity matrix `S` is calculated.

```
# Element conductivity
conductivity= [1*ones(1,16), ...
       2*ones(1,48), 1*ones(1,16)];

# Connectivity matrix
C = sparse ((1:D*E), reshape (elems', ...
       D*E, 1), 1, D*E, N);

# Calculate system matrix
Siidx = floor ([0:D*E-1]'/D) * D * ...
```

```
        ones(1,D) + ones(D*E,1)*(1:D) ;
Sjidx = [1:D*E]'*ones(1,D);
Sdata = zeros(D*E,D);
dfact = factorial(D-1);
for j=1:E
   a = inv([ones(D,1), ...
        nodes(elems(j,:), :)]);
   const = conductivity(j) * 2 / ...
        dfact / abs(det(a));
   Sdata(D*(j-1)+(1:D),:) = const * ...
        a(2:D,:)' * a(2:D,:);
endfor
# Element-wise system matrix
SE= sparse(Siidx,Sjidx,Sdata);
# Global system matrix
S= C'* SE *C;
```

The system matrix acts like the conductivity S in Ohm's law $SV = I$. Based on the Dirichlet and Neumann boundary conditions, we are able to solve for the voltages at each vertex `V`.

```
# Dirichlet boundary conditions
D_nodes=[1:5, 51:55];
D_value=[10*ones(1,5), 20*ones(1,5)];

V= zeros(N,1);
V(D_nodes) = D_value;
idx = 1:N; # vertices without Dirichlet
           # boundary condns
idx(D_nodes) = [];

# Neumann boundary conditions.  Note that
# N_value must be normalized by the
# boundary length and element conductivity
N_nodes=[];
N_value=[];

Q = zeros(N,1);
Q(N_nodes) = N_value;

V(idx) = S(idx,idx) \ ( Q(idx) - ...
          S(idx,D_nodes) * V(D_nodes));
```

Finally, in order to display the solution, we show each solved voltage value in the z-axis for each simplex vertex. See Figure 21.6.

```
elemx = elems(:,[1,2,3,1])';
xelems = reshape (nodes(elemx, 1), 4, E);
yelems = reshape (nodes(elemx, 2), 4, E);
velems = reshape (V(elemx), 4, E);
plot3 (xelems,yelems,velems,'k');
print ('grid.eps');
```

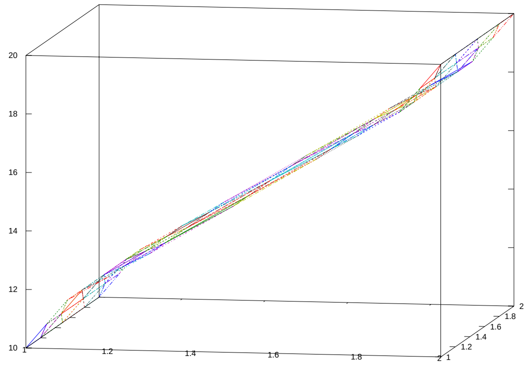

Figure 21.6: Example finite element model the showing triangular elements. The height of each vertex corresponds to the solution value.

22 Numerical Integration

Octave comes with several built-in functions for computing the integral of a function numerically. These functions all solve 1-dimensional integration problems.

22.1 Functions of One Variable

Octave supports three different algorithms for computing the integral

$$\int_a^b f(x)dx$$

of a function f over the interval from a to b. These are

`quad` Numerical integration based on Gaussian quadrature.

`quadl` Numerical integration using an adaptive Lobatto rule.

`quadgk` Numerical integration using an adaptive Gauss-Konrod rule.

`quadv` Numerical integration using an adaptive vectorized Simpson's rule.

`trapz` Numerical integration using the trapezoidal method.

Besides these functions Octave also allows you to perform cumulative numerical integration using the trapezoidal method through the `cumtrapz` function.

`[`*v*`,` *ier*`,` *nfun*`,` *err*`] = quad (`*f*, *a*, *b*, *tol*, *sing*`)` [Loadable Function]

Integrate a nonlinear function of one variable using Quadpack. The first argument is the name of the function, the function handle or the inline function to call to compute the value of the integrand. It must have the form

```
y = f (x)
```

where y and x are scalars.

The second and third arguments are limits of integration. Either or both may be infinite.

The optional argument *tol* is a vector that specifies the desired accuracy of the result. The first element of the vector is the desired absolute tolerance, and the second element is the desired relative tolerance. To choose a relative test only, set the absolute tolerance to zero. To choose an absolute test only, set the relative tolerance to zero.

The optional argument *sing* is a vector of values at which the integrand is known to be singular.

The result of the integration is returned in *v* and *ier* contains an integer error code (0 indicates a successful integration). The value of *nfun* indicates how many function evaluations were required, and *err* contains an estimate of the error in the solution.

You can use the function `quad_options` to set optional parameters for `quad`.

It should be noted that since `quad` is written in Fortran it cannot be called recursively.

`quad_options (opt, val)` [Loadable Function]

When called with two arguments, this function allows you set options parameters for the function `quad`. Given one argument, `quad_options` returns the value of the corresponding option. If no arguments are supplied, the names of all the available options and their current values are displayed.

Options include

`"absolute tolerance"`
: Absolute tolerance; may be zero for pure relative error test.

`"relative tolerance"`
: Nonnegative relative tolerance. If the absolute tolerance is zero, the relative tolerance must be greater than or equal to `max (50*eps, 0.5e-28)`.

`"single precision absolute tolerance"`
: Absolute tolerance for single precision; may be zero for pure relative error test.

`"single precision relative tolerance"`
: Nonnegative relative tolerance for single precision. If the absolute tolerance is zero, the relative tolerance must be greater than or equal to `max (50*eps, 0.5e-28)`.

Here is an example of using `quad` to integrate the function

$$f(x) = x \sin(1/x)\sqrt{|1-x|}$$

from $x = 0$ to $x = 3$.

This is a fairly difficult integration (plot the function over the range of integration to see why).

The first step is to define the function:

```
function y = f (x)
  y = x .* sin (1 ./ x) .* sqrt (abs (1 - x));
endfunction
```

Note the use of the 'dot' forms of the operators. This is not necessary for the call to `quad`, but it makes it much easier to generate a set of points for plotting (because it makes it possible to call the function with a vector argument to produce a vector result).

Then we simply call quad:

```
[v, ier, nfun, err] = quad ("f", 0, 3)
     ⇒ 1.9819
     ⇒ 1
     ⇒ 5061
     ⇒ 1.1522e-07
```

Although `quad` returns a nonzero value for *ier*, the result is reasonably accurate (to see why, examine what happens to the result if you move the lower bound to 0.1, then 0.01, then 0.001, etc.).

q = quadl (*f*, *a*, *b*) [Function File]
q = quadl (*f*, *a*, *b*, *tol*) [Function File]
q = quadl (*f*, *a*, *b*, *tol*, *trace*) [Function File]
q = quadl (*f*, *a*, *b*, *tol*, *trace*, *p1*, *p2*, . . .) [Function File]

Numerically evaluate integral using adaptive Lobatto rule. `quadl (f, a, b)` approximates the integral of `f(x)` to machine precision. *f* is either a function handle, inline function or string containing the name of the function to evaluate. The function *f* must return a vector of output values if given a vector of input values.

If defined, *tol* defines the relative tolerance to which to which to integrate `f(x)`. While if *trace* is defined, displays the left end point of the current interval, the interval length, and the partial integral.

Additional arguments *p1*, etc., are passed directly to *f*. To use default values for *tol* and *trace*, one may pass empty matrices.

Reference: W. Gander and W. Gautschi, 'Adaptive Quadrature - Revisited', BIT Vol. 40, No. 1, March 2000, pp. 84–101. http://www.inf.ethz.ch/personal/gander/

quadgk (*f*, *a*, *b*, *abstol*, *trace*) [Function File]
quadgk (*f*, *a*, *b*, *prop*, *val*, . . .) [Function File]
[*q*, *err*] = quadgk (. . .) [Function File]

Numerically evaluate integral using adaptive Gauss-Konrod quadrature. The formulation is based on a proposal by L.F. Shampine, "*Vectorized adaptive quadrature in* MATLAB", *Journal of Computational and Applied Mathematics, pp131-140, Vol 211, Issue 2, Feb 2008* where all function evaluations at an iteration are calculated with a single call to *f*. Therefore the function *f* must be of the form `f (x)` and accept vector values of *x* and return a vector of the same length representing the function evaluations at the given values of *x*. The function *f* can be defined in terms of a function handle, inline function or string.

The bounds of the quadrature `[a, b]` can be finite or infinite and contain weak end singularities. Variable transformation will be used to treat infinite intervals and weaken the singularities. For example

```
quadgk(@(x) 1 ./ (sqrt (x) .* (x + 1)), 0, Inf)
```

Note that the formulation of the integrand uses the element-by-element operator `./` and all user functions to `quadgk` should do the same.

The absolute tolerance can be passed as a fourth argument in a manner compatible with `quadv`. Equally the user can request that information on the convergence can be printed is the fifth argument is logically true.

Alternatively, certain properties of `quadgk` can be passed as pairs `prop, val`. Valid properties are

`AbsTol` Defines the absolute error tolerance for the quadrature. The default absolute tolerance is 1e-10.

`RelTol` Defines the relative error tolerance for the quadrature. The default relative tolerance is 1e-5.

`MaxIntervalCount`
`quadgk` initially subdivides the interval on which to perform the quadrature into 10 intervals. Sub-intervals that have an unacceptable error are

sub-divided and re-evaluated. If the number of sub-intervals exceeds at any point 650 sub-intervals then a poor convergence is signaled and the current estimate of the integral is returned. The property 'MaxIntervalCount' can be used to alter the number of sub-intervals that can exist before exiting.

`WayPoints`
: If there exists discontinuities in the first derivative of the function to integrate, then these can be flagged with the `"WayPoints"` property. This forces the ends of a sub-interval to fall on the breakpoints of the function and can result in significantly improved estimation of the error in the integral, faster computation or both. For example,

```
quadgk (@(x) abs (1 - x .^ 2), 0, 2, 'Waypoints', 1)
```

signals the breakpoint in the integrand at `x = 1`.

`Trace`
: If logically true, then `quadgk` prints information on the convergence of the quadrature at each iteration.

If any of *a*, *b* or *waypoints* is complex, then the quadrature is treated as a contour integral along a piecewise continuous path defined by the above. In this case the integral is assumed to have no edge singularities. For example

```
quadgk (@(z) log (z), 1+1i, 1+1i, "WayPoints",
        [1-1i, -1,-1i, -1+1i])
```

integrates `log (z)` along the square defined by `[1+1i, 1-1i, -1-1i, -1+1i]`

If two output arguments are requested, then *err* returns the approximate bounds on the error in the integral `abs (q - i)`, where *i* is the exact value of the integral.

See also: [triplequad], page 386, [dblquad], page 386, [quad], page 381, [quadl], page 382, [quadv], page 384, [trapz], page 385.

q = quadv (*f*, *a*, *b*) [Function File]
q = quadl (*f*, *a*, *b*, *tol*) [Function File]
q = quadl (*f*, *a*, *b*, *tol*, *trace*) [Function File]
q = quadl (*f*, *a*, *b*, *tol*, *trace*, *p1*, *p2*, . . .) [Function File]
[*q*, *fcnt*] = quadl (. . .) [Function File]

Numerically evaluate integral using adaptive Simpson's rule. `quadv (f, a, b)` approximates the integral of `f(x)` to the default absolute tolerance of `1e-6`. *f* is either a function handle, inline function or string containing the name of the function to evaluate. The function *f* must accept a string, and can return a vector representing the approximation to *n* different sub-functions.

If defined, *tol* defines the absolute tolerance to which to which to integrate each sub-interval of `f(x)`. While if *trace* is defined, displays the left end point of the current interval, the interval length, and the partial integral.

Additional arguments *p1*, etc., are passed directly to *f*. To use default values for *tol* and *trace*, one may pass empty matrices.

See also: [triplequad], page 386, [dblquad], page 386, [quad], page 381, [quadl], page 382, [quadgk], page 383, [trapz], page 385.

`z = trapz (y)` [Function File]
`z = trapz (x, y)` [Function File]
`z = trapz (..., dim)` [Function File]

Numerical integration using trapezoidal method. `trapz (y)` computes the integral of the *y* along the first non-singleton dimension. If the argument *x* is omitted a equally spaced vector is assumed. `trapz (x, y)` evaluates the integral with respect to *x*.

See also: [cumtrapz], page 385.

`z = cumtrapz (y)` [Function File]
`z = cumtrapz (x, y)` [Function File]
`z = cumtrapz (..., dim)` [Function File]

Cumulative numerical integration using trapezoidal method. `cumtrapz (y)` computes the cumulative integral of the *y* along the first non-singleton dimension. If the argument *x* is omitted a equally spaced vector is assumed. `cumtrapz (x, y)` evaluates the cumulative integral with respect to *x*.

See also: [trapz], page 385, [cumsum], page 299.

22.2 Orthogonal Collocation

`[r, amat, bmat, q] = colloc (n, "left", "right")` [Loadable Function]

Compute derivative and integral weight matrices for orthogonal collocation using the subroutines given in J. Villadsen and M. L. Michelsen, *Solution of Differential Equation Models by Polynomial Approximation*.

Here is an example of using `colloc` to generate weight matrices for solving the second order differential equation $u' - \alpha u'' = 0$ with the boundary conditions $u(0) = 0$ and $u(1) = 1$.

First, we can generate the weight matrices for n points (including the endpoints of the interval), and incorporate the boundary conditions in the right hand side (for a specific value of α).

```
n = 7;
alpha = 0.1;
[r, a, b] = colloc (n-2, "left", "right");
at = a(2:n-1,2:n-1);
bt = b(2:n-1,2:n-1);
rhs = alpha * b(2:n-1,n) - a(2:n-1,n);
```

Then the solution at the roots r is

```
u = [ 0; (at - alpha * bt) \ rhs; 1]
     ⇒ [ 0.00; 0.004; 0.01 0.00; 0.12; 0.62; 1.00 ]
```

22.3 Functions of Multiple Variables

Octave does not have built-in functions for computing the integral of functions of multiple variables directly. It is however possible to compute the integral of a function of multiple variables using the functions for one-dimensional integrals.

To illustrate how the integration can be performed, we will integrate the function

$$f(x, y) = \sin(\pi xy)\sqrt{xy}$$

for x and y between 0 and 1.

The first approach creates a function that integrates f with respect to x, and then integrates that function with respect to y. Since `quad` is written in Fortran it cannot be called recursively. This means that `quad` cannot integrate a function that calls `quad`, and hence cannot be used to perform the double integration. It is however possible with `quadl`, which is what the following code does.

```
function I = g(y)
  I = ones(1, length(y));
  for i = 1:length(y)
    f = @(x) sin(pi.*x.*y(i)).*sqrt(x.*y(i));
    I(i) = quadl(f, 0, 1);
  endfor
endfunction

I = quadl("g", 0, 1)
      ⇒ 0.30022
```

The above process can be simplified with the `dblquad` and `triplequad` functions for integrals over two and three variables. For example

```
I =  dblquad (@(x, y) sin(pi.*x.*y).*sqrt(x.*y), 0, 1, 0, 1)
      ⇒ 0.30022
```

`dblquad` (*f*, *xa*, *xb*, *ya*, *yb*, *tol*, *quadf*, . . .) [Function File]

Numerically evaluate a double integral. The function over with to integrate is defined by `f`, and the interval for the integration is defined by `[xa, xb, ya, yb]`. The function *f* must accept a vector *x* and a scalar *y*, and return a vector of the same length as *x*.

If defined, *tol* defines the absolute tolerance to which to which to integrate each sub-integral.

Additional arguments, are passed directly to *f*. To use the default value for *tol* one may pass an empty matrix.

See also: [triplequad], page 386, [quad], page 381, [quadv], page 384, [quadl], page 382, [quadgk], page 383, [trapz], page 385.

`triplequad` (*f*, *xa*, *xb*, *ya*, *yb*, *za*, *zb*, *tol*, *quadf*, . . .) [Function File]

Numerically evaluate a triple integral. The function over which to integrate is defined by `f`, and the interval for the integration is defined by `[xa, xb, ya, yb, za, zb]`. The function *f* must accept a vector *x* and a scalar *y*, and return a vector of the same length as *x*.

If defined, *tol* defines the absolute tolerance to which to which to integrate each sub-integral.

Additional arguments, are passed directly to *f*. To use the default value for *tol* one may pass an empty matrix.

See also: [dblquad], page 386, [quad], page 381, [quadv], page 384, [quadl], page 382, [quadgk], page 383, [trapz], page 385.

The above mentioned approach works but is fairly slow, and that problem increases exponentially with the dimensionality the problem. Another possible solution is to use Orthogonal Collocation as described in the previous section. The integral of a function $f(x, y)$ for x and y between 0 and 1 can be approximated using n points by

$$\int_0^1 \int_0^1 f(x,y) dx dy \approx \sum_{i=1}^{n} \sum_{j=1}^{n} q_i q_j f(r_i, r_j),$$

where q and r is as returned by `colloc(n)`. The generalization to more than two variables is straight forward. The following code computes the studied integral using $n = 7$ points.

```
f = @(x,y) sin(pi*x*y').*sqrt(x*y');
n = 7;
[t, A, B, q] = colloc(n);
I = q'*f(t,t)*q;
     ⇒ 0.30022
```

It should be noted that the number of points determines the quality of the approximation. If the integration needs to be performed between a and b instead of 0 and 1, a change of variables is needed.

23 Differential Equations

Octave has built-in functions for solving ordinary differential equations, and differential-algebraic equations. All solvers are based on reliable ODE routines written in Fortran.

23.1 Ordinary Differential Equations

The function `lsode` can be used to solve ODEs of the form

$$\frac{dx}{dt} = f(x, t)$$

using Hindmarsh's ODE solver LSODE.

`[x, istate, msg] = lsode (fcn, x_0, t, t_crit)` [Loadable Function]

Solve the set of differential equations

$$\frac{dx}{dt} = f(x, t)$$

with

$$x(t_0) = x_0$$

The solution is returned in the matrix *x*, with each row corresponding to an element of the vector *t*. The first element of *t* should be t_0 and should correspond to the initial state of the system *x_0*, so that the first row of the output is *x_0*.

The first argument, *fcn*, is a string, inline, or function handle that names the function f to call to compute the vector of right hand sides for the set of equations. The function must have the form

```
xdot = f (x, t)
```

in which *xdot* and *x* are vectors and *t* is a scalar.

If *fcn* is a two-element string array or a two-element cell array of strings, inline functions, or function handles, the first element names the function f described above, and the second element names a function to compute the Jacobian of f. The Jacobian function must have the form

```
jac = j (x, t)
```

in which *jac* is the matrix of partial derivatives

$$J = \frac{\partial f_i}{\partial x_j} = \begin{bmatrix} \frac{\partial f_1}{\partial x_1} & \frac{\partial f_1}{\partial x_2} & \cdots & \frac{\partial f_1}{\partial x_N} \\ \frac{\partial f_2}{\partial x_1} & \frac{\partial f_2}{\partial x_2} & \cdots & \frac{\partial f_2}{\partial x_N} \\ \vdots & \vdots & \ddots & \vdots \\ \frac{\partial f_3}{\partial x_1} & \frac{\partial f_3}{\partial x_2} & \cdots & \frac{\partial f_3}{\partial x_N} \end{bmatrix}$$

The second and third arguments specify the initial state of the system, x_0, and the initial value of the independent variable t_0.

The fourth argument is optional, and may be used to specify a set of times that the ODE solver should not integrate past. It is useful for avoiding difficulties with singularities and points where there is a discontinuity in the derivative.

After a successful computation, the value of *istate* will be 2 (consistent with the Fortran version of LSODE).

If the computation is not successful, *istate* will be something other than 2 and *msg* will contain additional information.

You can use the function `lsode_options` to set optional parameters for `lsode`.

See also: [daspk], page 391, [dassl], page 395, [dasrt], page 397.

`lsode_options (`*opt*`, `*val*`)` [Loadable Function]

When called with two arguments, this function allows you set options parameters for the function `lsode`. Given one argument, `lsode_options` returns the value of the corresponding option. If no arguments are supplied, the names of all the available options and their current values are displayed.

Options include

`"absolute tolerance"`
: Absolute tolerance. May be either vector or scalar. If a vector, it must match the dimension of the state vector.

`"relative tolerance"`
: Relative tolerance parameter. Unlike the absolute tolerance, this parameter may only be a scalar.

  The local error test applied at each integration step is

```
abs (local error in x(i)) <= ...
    rtol * abs (y(i)) + atol(i)
```

`"integration method"`
: A string specifying the method of integration to use to solve the ODE system. Valid values are

  "adams"
  "non-stiff"
  : No Jacobian used (even if it is available).

  "bdf"
  "stiff"
  : Use stiff backward differentiation formula (BDF) method. If a function to compute the Jacobian is not supplied, `lsode` will compute a finite difference approximation of the Jacobian matrix.

`"initial step size"`
: The step size to be attempted on the first step (default is determined automatically).

`"maximum order"`
: Restrict the maximum order of the solution method. If using the Adams method, this option must be between 1 and 12. Otherwise, it must be between 1 and 5, inclusive.

`"maximum step size"`
: Setting the maximum stepsize will avoid passing over very large regions (default is not specified).

"minimum step size"
The minimum absolute step size allowed (default is 0).

"step limit"
Maximum number of steps allowed (default is 100000).

Here is an example of solving a set of three differential equations using `lsode`. Given the function

```
function xdot = f (x, t)

  xdot = zeros (3,1);

  xdot(1) = 77.27 * (x(2) - x(1)*x(2) + x(1) \
            - 8.375e-06*x(1)^2);
  xdot(2) = (x(3) - x(1)*x(2) - x(2)) / 77.27;
  xdot(3) = 0.161*(x(1) - x(3));

endfunction
```

and the initial condition `x0 = [ 4; 1.1; 4 ]`, the set of equations can be integrated using the command

```
t = linspace (0, 500, 1000);

y = lsode ("f", x0, t);
```

If you try this, you will see that the value of the result changes dramatically between $t = 0$ and 5, and again around $t = 305$. A more efficient set of output points might be

```
t = [0, logspace (-1, log10(303), 150), \
        logspace (log10(304), log10(500), 150)];
```

See Alan C. Hindmarsh, *ODEPACK, A Systematized Collection of ODE Solvers*, in Scientific Computing, R. S. Stepleman, editor, (1983) for more information about the inner workings of `lsode`.

23.2 Differential-Algebraic Equations

The function `daspk` can be used to solve DAEs of the form

$$0 = f(\dot{x}, x, t), \qquad x(t = 0) = x_0, \dot{x}(t = 0) = \dot{x}_0$$

where $\dot{x} = \frac{dx}{dt}$ is the derivative of x. The equation is solved using Petzold's DAE solver Daspk.

[*x*, *xdot*, *istate*, *msg*] = daspk (*fcn*, *x_0*, *xdot_0*, *t*, *t_crit*) [Loadable Function]

Solve the set of differential-algebraic equations

$$0 = f(x, \dot{x}, t)$$

with

$$x(t_0) = x_0, \dot{x}(t_0) = \dot{x}_0$$

The solution is returned in the matrices *x* and *xdot*, with each row in the result matrices corresponding to one of the elements in the vector *t*. The first element of *t* should be t_0 and correspond to the initial state of the system *x_0* and its derivative *xdot_0*, so that the first row of the output *x* is *x_0* and the first row of the output *xdot* is *xdot_0*.

The first argument, *fcn*, is a string, inline, or function handle that names the function *f* to call to compute the vector of residuals for the set of equations. It must have the form

```
res = f (x, xdot, t)
```

in which *x*, *xdot*, and *res* are vectors, and *t* is a scalar.

If *fcn* is a two-element string array or a two-element cell array of strings, inline functions, or function handles, the first element names the function *f* described above, and the second element names a function to compute the modified Jacobian

$$J = \frac{\partial f}{\partial x} + c \frac{\partial f}{\partial \dot{x}}$$

The modified Jacobian function must have the form

```
jac = j (x, xdot, t, c)
```

The second and third arguments to `daspk` specify the initial condition of the states and their derivatives, and the fourth argument specifies a vector of output times at which the solution is desired, including the time corresponding to the initial condition.

The set of initial states and derivatives are not strictly required to be consistent. If they are not consistent, you must use the `daspk_options` function to provide additional information so that `daspk` can compute a consistent starting point.

The fifth argument is optional, and may be used to specify a set of times that the DAE solver should not integrate past. It is useful for avoiding difficulties with singularities and points where there is a discontinuity in the derivative.

After a successful computation, the value of *istate* will be greater than zero (consistent with the Fortran version of DASPK).

If the computation is not successful, the value of *istate* will be less than zero and *msg* will contain additional information.

You can use the function `daspk_options` to set optional parameters for `daspk`.

See also: [dassl], page 395.

`daspk_options (`*opt*, *val*`)` [Loadable Function]

When called with two arguments, this function allows you set options parameters for the function `daspk`. Given one argument, `daspk_options` returns the value of the corresponding option. If no arguments are supplied, the names of all the available options and their current values are displayed.

Options include

`"absolute tolerance"`
: Absolute tolerance. May be either vector or scalar. If a vector, it must match the dimension of the state vector, and the relative tolerance must also be a vector of the same length.

`"relative tolerance"`
: Relative tolerance. May be either vector or scalar. If a vector, it must match the dimension of the state vector, and the absolute tolerance must also be a vector of the same length.

: The local error test applied at each integration step is

```
abs (local error in x(i))
     <= rtol(i) * abs (Y(i)) + atol(i)
```

`"compute consistent initial condition"`
: Denoting the differential variables in the state vector by '`Y_d`' and the algebraic variables by '`Y_a`', `ddaspk` can solve one of two initialization problems:

1. Given Y_d, calculate Y_a and Y'_d
2. Given Y', calculate Y.

: In either case, initial values for the given components are input, and initial guesses for the unknown components must also be provided as input. Set this option to 1 to solve the first problem, or 2 to solve the second (the default is 0, so you must provide a set of initial conditions that are consistent).

: If this option is set to a nonzero value, you must also set the `"algebraic variables"` option to declare which variables in the problem are algebraic.

`"use initial condition heuristics"`
: Set to a nonzero value to use the initial condition heuristics options described below.

`"initial condition heuristics"`
: A vector of the following parameters that can be used to control the initial condition calculation.

`MXNIT`
: Maximum number of Newton iterations (default is 5).

`MXNJ`
: Maximum number of Jacobian evaluations (default is 6).

`MXNH`
: Maximum number of values of the artificial stepsize parameter to be tried if the `"compute consistent initial condition"` option has been set to 1 (default is 5).

: Note that the maximum total number of Newton iterations allowed is `MXNIT*MXNJ*MXNH` if the `"compute consistent initial condition"` option has been set to 1 and `MXNIT*MXNJ` if it is set to 2.

`LSOFF`
: Set to a nonzero value to disable the linesearch algorithm (default is 0).

`STPTOL` Minimum scaled step in linesearch algorithm (default is eps^(2/3)).

`EPINIT` Swing factor in the Newton iteration convergence test. The test is applied to the residual vector, premultiplied by the approximate Jacobian. For convergence, the weighted RMS norm of this vector (scaled by the error weights) must be less than `EPINIT*EPCON`, where `EPCON` = 0.33 is the analogous test constant used in the time steps. The default is `EPINIT` = 0.01.

`"print initial condition info"`
Set this option to a nonzero value to display detailed information about the initial condition calculation (default is 0).

`"exclude algebraic variables from error test"`
Set to a nonzero value to exclude algebraic variables from the error test. You must also set the `"algebraic variables"` option to declare which variables in the problem are algebraic (default is 0).

`"algebraic variables"`
A vector of the same length as the state vector. A nonzero element indicates that the corresponding element of the state vector is an algebraic variable (i.e., its derivative does not appear explicitly in the equation set.

This option is required by the `compute consistent initial condition"` and `"exclude algebraic variables from error test"` options.

`"enforce inequality constraints"`
Set to one of the following values to enforce the inequality constraints specified by the `"inequality constraint types"` option (default is 0).

1. To have constraint checking only in the initial condition calculation.
2. To enforce constraint checking during the integration.
3. To enforce both options 1 and 2.

`"inequality constraint types"`
A vector of the same length as the state specifying the type of inequality constraint. Each element of the vector corresponds to an element of the state and should be assigned one of the following codes

-2 Less than zero.

-1 Less than or equal to zero.

0 Not constrained.

1 Greater than or equal to zero.

2 Greater than zero.

This option only has an effect if the `"enforce inequality constraints"` option is nonzero.

`"initial step size"`
: Differential-algebraic problems may occasionally suffer from severe scaling difficulties on the first step. If you know a great deal about the scaling of your problem, you can help to alleviate this problem by specifying an initial stepsize (default is computed automatically).

`"maximum order"`
: Restrict the maximum order of the solution method. This option must be between 1 and 5, inclusive (default is 5).

`"maximum step size"`
: Setting the maximum stepsize will avoid passing over very large regions (default is not specified).

Octave also includes DASSL, an earlier version of *Daspk*, and *dasrt*, which can be used to solve DAEs with constraints (stopping conditions).

`[x, xdot, istate, msg] = dassl (fcn, x_0, xdot_0, t, t_crit)` [Loadable Function]

Solve the set of differential-algebraic equations

$$0 = f(x, \dot{x}, t)$$

with

$$x(t_0) = x_0, \dot{x}(t_0) = \dot{x}_0$$

The solution is returned in the matrices *x* and *xdot*, with each row in the result matrices corresponding to one of the elements in the vector *t*. The first element of *t* should be t_0 and correspond to the initial state of the system *x_0* and its derivative *xdot_0*, so that the first row of the output *x* is *x_0* and the first row of the output *xdot* is *xdot_0*.

The first argument, *fcn*, is a string, inline, or function handle that names the function *f* to call to compute the vector of residuals for the set of equations. It must have the form

```
res = f (x, xdot, t)
```

in which *x*, *xdot*, and *res* are vectors, and *t* is a scalar.

If *fcn* is a two-element string array or a two-element cell array of strings, inline functions, or function handles, the first element names the function *f* described above, and the second element names a function to compute the modified Jacobian

$$J = \frac{\partial f}{\partial x} + c\frac{\partial f}{\partial \dot{x}}$$

The modified Jacobian function must have the form

```
jac = j (x, xdot, t, c)
```

The second and third arguments to `dassl` specify the initial condition of the states and their derivatives, and the fourth argument specifies a vector of output times at which the solution is desired, including the time corresponding to the initial condition.

The set of initial states and derivatives are not strictly required to be consistent. In practice, however, Dassl is not very good at determining a consistent set for you, so it is best if you ensure that the initial values result in the function evaluating to zero.

The fifth argument is optional, and may be used to specify a set of times that the DAE solver should not integrate past. It is useful for avoiding difficulties with singularities and points where there is a discontinuity in the derivative.

After a successful computation, the value of *istate* will be greater than zero (consistent with the Fortran version of Dassl).

If the computation is not successful, the value of *istate* will be less than zero and *msg* will contain additional information.

You can use the function `dassl_options` to set optional parameters for `dassl`.

See also: [daspk], page 391, [dasrt], page 397, [lsode], page 389.

`dassl_options (`*opt*, *val*`)` [Loadable Function]

When called with two arguments, this function allows you set options parameters for the function `dassl`. Given one argument, `dassl_options` returns the value of the corresponding option. If no arguments are supplied, the names of all the available options and their current values are displayed.

Options include

`"absolute tolerance"`
: Absolute tolerance. May be either vector or scalar. If a vector, it must match the dimension of the state vector, and the relative tolerance must also be a vector of the same length.

`"relative tolerance"`
: Relative tolerance. May be either vector or scalar. If a vector, it must match the dimension of the state vector, and the absolute tolerance must also be a vector of the same length.

  The local error test applied at each integration step is

```
abs (local error in x(i))
     <= rtol(i) * abs (Y(i)) + atol(i)
```

`"compute consistent initial condition"`
: If nonzero, `dassl` will attempt to compute a consistent set of initial conditions. This is generally not reliable, so it is best to provide a consistent set and leave this option set to zero.

`"enforce nonnegativity constraints"`
: If you know that the solutions to your equations will always be nonnegative, it may help to set this parameter to a nonzero value. However, it is probably best to try leaving this option set to zero first, and only setting it to a nonzero value if that doesn't work very well.

`"initial step size"`
: Differential-algebraic problems may occasionally suffer from severe scaling difficulties on the first step. If you know a great deal about the scaling of your problem, you can help to alleviate this problem by specifying an initial stepsize.

`"maximum order"`
: Restrict the maximum order of the solution method. This option must be between 1 and 5, inclusive.

`"maximum step size"`
: Setting the maximum stepsize will avoid passing over very large regions (default is not specified).

`"step limit"`
: Maximum number of integration steps to attempt on a single call to the underlying Fortran code.

`[x, xdot, t_out, istat, msg] = dasrt (fcn [, g], x_0, xdot_0, t [, t_crit])` [Loadable Function]

Solve the set of differential-algebraic equations

$$0 = f(x, \dot{x}, t)$$

with

$$x(t_0) = x_0, \dot{x}(t_0) = \dot{x}_0$$

with functional stopping criteria (root solving).

The solution is returned in the matrices *x* and *xdot*, with each row in the result matrices corresponding to one of the elements in the vector *t_out*. The first element of *t* should be t_0 and correspond to the initial state of the system *x_0* and its derivative *xdot_0*, so that the first row of the output *x* is *x_0* and the first row of the output *xdot* is *xdot_0*.

The vector *t* provides an upper limit on the length of the integration. If the stopping condition is met, the vector *t_out* will be shorter than *t*, and the final element of *t_out* will be the point at which the stopping condition was met, and may not correspond to any element of the vector *t*.

The first argument, *fcn*, is a string, inline, or function handle that names the function *f* to call to compute the vector of residuals for the set of equations. It must have the form

```
res = f (x, xdot, t)
```

in which *x*, *xdot*, and *res* are vectors, and *t* is a scalar.

If *fcn* is a two-element string array or a two-element cell array of strings, inline functions, or function handles, the first element names the function *f* described above, and the second element names a function to compute the modified Jacobian

$$J = \frac{\partial f}{\partial x} + c\frac{\partial f}{\partial \dot{x}}$$

The modified Jacobian function must have the form

```
jac = j (x, xdot, t, c)
```

The optional second argument names a function that defines the constraint functions whose roots are desired during the integration. This function must have the form

```
g_out = g (x, t)
```

and return a vector of the constraint function values. If the value of any of the constraint functions changes sign, DASRT will attempt to stop the integration at the point of the sign change.

If the name of the constraint function is omitted, `dasrt` solves the same problem as `daspk` or `dassl`.

Note that because of numerical errors in the constraint functions due to roundoff and integration error, DASRT may return false roots, or return the same root at two or more nearly equal values of T. If such false roots are suspected, the user should consider smaller error tolerances or higher precision in the evaluation of the constraint functions.

If a root of some constraint function defines the end of the problem, the input to DASRT should nevertheless allow integration to a point slightly past that root, so that DASRT can locate the root by interpolation.

The third and fourth arguments to `dasrt` specify the initial condition of the states and their derivatives, and the fourth argument specifies a vector of output times at which the solution is desired, including the time corresponding to the initial condition.

The set of initial states and derivatives are not strictly required to be consistent. In practice, however, DASSL is not very good at determining a consistent set for you, so it is best if you ensure that the initial values result in the function evaluating to zero.

The sixth argument is optional, and may be used to specify a set of times that the DAE solver should not integrate past. It is useful for avoiding difficulties with singularities and points where there is a discontinuity in the derivative.

After a successful computation, the value of *istate* will be greater than zero (consistent with the Fortran version of DASSL).

If the computation is not successful, the value of *istate* will be less than zero and *msg* will contain additional information.

You can use the function `dasrt_options` to set optional parameters for `dasrt`.

See also: [daspk], page 391, [dasrt], page 397, [lsode], page 389.

`dasrt_options (`*opt*`, `*val*`)` [Loadable Function]

When called with two arguments, this function allows you set options parameters for the function `dasrt`. Given one argument, `dasrt_options` returns the value of the corresponding option. If no arguments are supplied, the names of all the available options and their current values are displayed.

Options include

`"absolute tolerance"`
: Absolute tolerance. May be either vector or scalar. If a vector, it must match the dimension of the state vector, and the relative tolerance must also be a vector of the same length.

`"relative tolerance"`
: Relative tolerance. May be either vector or scalar. If a vector, it must match the dimension of the state vector, and the absolute tolerance must also be a vector of the same length.

The local error test applied at each integration step is

```
abs (local error in x(i)) <= ...
    rtol(i) * abs (Y(i)) + atol(i)
```

`"initial step size"`
: Differential-algebraic problems may occasionally suffer from severe scaling difficulties on the first step. If you know a great deal about the scaling of your problem, you can help to alleviate this problem by specifying an initial stepsize.

`"maximum order"`
: Restrict the maximum order of the solution method. This option must be between 1 and 5, inclusive.

`"maximum step size"`
: Setting the maximum stepsize will avoid passing over very large regions.

`"step limit"`
: Maximum number of integration steps to attempt on a single call to the underlying Fortran code.

See K. E. Brenan, et al., *Numerical Solution of Initial-Value Problems in Differential-Algebraic Equations*, North-Holland (1989) for more information about the implementation of Dassl.

24 Optimization

Octave comes with support for solving various kinds of optimization problems. Specifically Octave can solve problems in Linear Programming, Quadratic Programming, Nonlinear Programming, and Linear Least Squares Minimization.

24.1 Linear Programming

Octave can solve Linear Programming problems using the `glpk` function. That is, Octave can solve

$$\min_x c^T x$$

subject to the linear constraints $Ax = b$ where $x \geq 0$.

The `glpk` function also supports variations of this problem.

`[xopt, fmin, status, extra] = glpk (c, a, b, lb, ub, ctype, vartype, sense, param)` [Function File]

Solve a linear program using the GNU GLPK library. Given three arguments, `glpk` solves the following standard LP:

$$\min_x C^T x$$

subject to

$$Ax = b \qquad x \geq 0$$

but may also solve problems of the form

$$[\min_x \mid \max_x] C^T x$$

subject to

$$Ax[= \mid \leq \mid \geq]b \qquad LB \leq x \leq UB$$

Input arguments:

c A column array containing the objective function coefficients.

a A matrix containing the constraints coefficients.

b A column array containing the right-hand side value for each constraint in the constraint matrix.

lb An array containing the lower bound on each of the variables. If *lb* is not supplied, the default lower bound for the variables is zero.

ub An array containing the upper bound on each of the variables. If *ub* is not supplied, the default upper bound is assumed to be infinite.

ctype An array of characters containing the sense of each constraint in the constraint matrix. Each element of the array may be one of the following values

"F" A free (unbounded) constraint (the constraint is ignored).

"U" An inequality constraint with an upper bound (`A(i,:)*x <= b(i)`).

"S" An equality constraint (`A(i,:)*x = b(i)`).

"L" An inequality with a lower bound (`A(i,:)*x >= b(i)`).

"D" An inequality constraint with both upper and lower bounds (`A(i,:)*x >= -b(i)` *and* (`A(i,:)*x <= b(i)`).

vartype A column array containing the types of the variables.

"C" A continuous variable.

"I" An integer variable.

sense If *sense* is 1, the problem is a minimization. If *sense* is -1, the problem is a maximization. The default value is 1.

param A structure containing the following parameters used to define the behavior of solver. Missing elements in the structure take on default values, so you only need to set the elements that you wish to change from the default.

Integer parameters:

`msglev (LPX_K_MSGLEV, default: 1)`
Level of messages output by solver routines:

0 No output.

1 Error messages only.

2 Normal output .

3 Full output (includes informational messages).

`scale (LPX_K_SCALE, default: 1)`
Scaling option:

0 No scaling.

1 Equilibration scaling.

2 Geometric mean scaling, then equilibration scaling.

`dual (LPX_K_DUAL, default: 0)`
Dual simplex option:

0 Do not use the dual simplex.

1 If initial basic solution is dual feasible, use the dual simplex.

`price (LPX_K_PRICE, default: 1)`
: Pricing option (for both primal and dual simplex):

  0 Textbook pricing.

  1 Steepest edge pricing.

`round (LPX_K_ROUND, default: 0)`
: Solution rounding option:

  0 Report all primal and dual values "as is".

  1 Replace tiny primal and dual values by exact zero.

`itlim (LPX_K_ITLIM, default: -1)`
: Simplex iterations limit. If this value is positive, it is decreased by one each time when one simplex iteration has been performed, and reaching zero value signals the solver to stop the search. Negative value means no iterations limit.

`itcnt (LPX_K_OUTFRQ, default: 200)`
: Output frequency, in iterations. This parameter specifies how frequently the solver sends information about the solution to the standard output.

`branch (LPX_K_BRANCH, default: 2)`
: Branching heuristic option (for MIP only):

  0 Branch on the first variable.

  1 Branch on the last variable.

  2 Branch using a heuristic by Driebeck and Tomlin.

`btrack (LPX_K_BTRACK, default: 2)`
: Backtracking heuristic option (for MIP only):

  0 Depth first search.

  1 Breadth first search.

  2 Backtrack using the best projection heuristic.

`presol (LPX_K_PRESOL, default: 1)`
: If this flag is set, the routine lpx_simplex solves the problem using the built-in LP presolver. Otherwise the LP presolver is not used.

`lpsolver (default: 1)`
: Select which solver to use. If the problem is a MIP problem this flag will be ignored.

  1 Revised simplex method.

  2 Interior point method.

`save (default: 0)`
: If this parameter is nonzero, save a copy of the problem in CPLEX LP format to the file '`"outpb.lp"`'. There is currently no way to change the name of the output file.

Real parameters:

`relax (LPX_K_RELAX, default: 0.07)`
: Relaxation parameter used in the ratio test. If it is zero, the textbook ratio test is used. If it is non-zero (should be positive), Harris' two-pass ratio test is used. In the latter case on the first pass of the ratio test basic variables (in the case of primal simplex) or reduced costs of non-basic variables (in the case of dual simplex) are allowed to slightly violate their bounds, but not more than `relax*tolbnd` or `relax*toldj (thus, relax is a percentage of tolbnd or toldj`.

`tolbnd (LPX_K_TOLBND, default: 10e-7)`
: Relative tolerance used to check if the current basic solution is primal feasible. It is not recommended that you change this parameter unless you have a detailed understanding of its purpose.

`toldj (LPX_K_TOLDJ, default: 10e-7)`
: Absolute tolerance used to check if the current basic solution is dual feasible. It is not recommended that you change this parameter unless you have a detailed understanding of its purpose.

`tolpiv (LPX_K_TOLPIV, default: 10e-9)`
: Relative tolerance used to choose eligible pivotal elements of the simplex table. It is not recommended that you change this parameter unless you have a detailed understanding of its purpose.

`objll (LPX_K_OBJLL, default: -DBL_MAX)`
: Lower limit of the objective function. If on the phase II the objective function reaches this limit and continues decreasing, the solver stops the search. This parameter is used in the dual simplex method only.

`objul (LPX_K_OBJUL, default: +DBL_MAX)`
: Upper limit of the objective function. If on the phase II the objective function reaches this limit and continues increasing, the solver stops the search. This parameter is used in the dual simplex only.

`tmlim (LPX_K_TMLIM, default: -1.0)`
: Searching time limit, in seconds. If this value is positive, it is decreased each time when one simplex iteration has been performed by the amount of time spent for the iteration, and

reaching zero value signals the solver to stop the search. Negative value means no time limit.

`outdly (LPX_K_OUTDLY, default: 0.0)`
: Output delay, in seconds. This parameter specifies how long the solver should delay sending information about the solution to the standard output. Non-positive value means no delay.

`tolint (LPX_K_TOLINT, default: 10e-5)`
: Relative tolerance used to check if the current basic solution is integer feasible. It is not recommended that you change this parameter unless you have a detailed understanding of its purpose.

`tolobj (LPX_K_TOLOBJ, default: 10e-7)`
: Relative tolerance used to check if the value of the objective function is not better than in the best known integer feasible solution. It is not recommended that you change this parameter unless you have a detailed understanding of its purpose.

Output values:

xopt The optimizer (the value of the decision variables at the optimum).

fopt The optimum value of the objective function.

status Status of the optimization.

Simplex Method:

180 (`LPX_OPT`)
: Solution is optimal.

181 (`LPX_FEAS`)
: Solution is feasible.

182 (`LPX_INFEAS`)
: Solution is infeasible.

183 (`LPX_NOFEAS`)
: Problem has no feasible solution.

184 (`LPX_UNBND`)
: Problem has no unbounded solution.

185 (`LPX_UNDEF`)
: Solution status is undefined.

Interior Point Method:

150 (`LPX_T_UNDEF`)
: The interior point method is undefined.

151 (`LPX_T_OPT`)
: The interior point method is optimal.

Mixed Integer Method:

170 (`LPX_I_UNDEF`)
: The status is undefined.

171 (`LPX_I_OPT`)
: The solution is integer optimal.

172 (`LPX_I_FEAS`)
: Solution integer feasible but its optimality has not been proven

173 (`LPX_I_NOFEAS`)
: No integer feasible solution.

If an error occurs, *status* will contain one of the following codes:

204 (`LPX_E_FAULT`)
: Unable to start the search.

205 (`LPX_E_OBJLL`)
: Objective function lower limit reached.

206 (`LPX_E_OBJUL`)
: Objective function upper limit reached.

207 (`LPX_E_ITLIM`)
: Iterations limit exhausted.

208 (`LPX_E_TMLIM`)
: Time limit exhausted.

209 (`LPX_E_NOFEAS`)
: No feasible solution.

210 (`LPX_E_INSTAB`)
: Numerical instability.

211 (`LPX_E_SING`)
: Problems with basis matrix.

212 (`LPX_E_NOCONV`)
: No convergence (interior).

213 (`LPX_E_NOPFS`)
: No primal feasible solution (LP presolver).

214 (`LPX_E_NODFS`)
: No dual feasible solution (LP presolver).

extra A data structure containing the following fields:

`lambda` Dual variables.

`redcosts` Reduced Costs.

`time` Time (in seconds) used for solving LP/MIP problem.

`mem` Memory (in bytes) used for solving LP/MIP problem (this is not available if the version of GLPK is 4.15 or later).

Example:

```
c = [10, 6, 4]';
a = [ 1, 1, 1;
     10, 4, 5;
      2, 2, 6];
b = [100, 600, 300]';
lb = [0, 0, 0]';
ub = [];
ctype = "UUU";
vartype = "CCC";
s = -1;

param.msglev = 1;
param.itlim = 100;

[xmin, fmin, status, extra] = ...
   glpk (c, a, b, lb, ub, ctype, vartype, s, param);
```

24.2 Quadratic Programming

Octave can also solve Quadratic Programming problems, this is

$$\min_x \frac{1}{2} x^T H x + x^T q$$

subject to

$$Ax = b \qquad lb \leq x \leq ub \qquad A_{lb} \leq A_{in} \leq A_{ub}$$

`[x, obj, info, lambda] = qp (x0, H, q, A, b, lb, ub, A_lb, A_in, A_ub)` [Function File]

Solve the quadratic program

$$\min_x \frac{1}{2} x^T H x + x^T q$$

subject to

$$Ax = b \qquad lb \leq x \leq ub \qquad A_{lb} \leq A_{in} \leq A_{ub}$$

using a null-space active-set method.

Any bound (*A*, *b*, *lb*, *ub*, *A_lb*, *A_ub*) may be set to the empty matrix (`[]`) if not present. If the initial guess is feasible the algorithm is faster.

The value *info* is a structure with the following fields:

`solveiter`
The number of iterations required to find the solution.

`info` An integer indicating the status of the solution, as follows:

0	The problem is feasible and convex. Global solution found.
1	The problem is not convex. Local solution found.
2	The problem is not convex and unbounded.
3	Maximum number of iterations reached.
6	The problem is infeasible.

24.3 Nonlinear Programming

Octave can also perform general nonlinear minimization using a successive quadratic programming solver.

`[x, obj, info, iter, nf, lambda] = sqp (x, phi, g, h, lb, ub, maxiter, tolerance)` [Function File]

Solve the nonlinear program

$$\min_x \phi(x)$$

subject to

$$g(x) = 0 \qquad h(x) \geq 0 \qquad lb \leq x \leq ub$$

using a successive quadratic programming method.

The first argument is the initial guess for the vector *x*.

The second argument is a function handle pointing to the objective function. The objective function must be of the form

```
y = phi (x)
```

in which *x* is a vector and *y* is a scalar.

The second argument may also be a 2- or 3-element cell array of function handles. The first element should point to the objective function, the second should point to a function that computes the gradient of the objective function, and the third should point to a function to compute the hessian of the objective function. If the gradient function is not supplied, the gradient is computed by finite differences. If the hessian function is not supplied, a BFGS update formula is used to approximate the hessian.

If supplied, the gradient function must be of the form

```
g = gradient (x)
```

in which *x* is a vector and *g* is a vector.

If supplied, the hessian function must be of the form

```
h = hessian (x)
```

in which *x* is a vector and *h* is a matrix.

The third and fourth arguments are function handles pointing to functions that compute the equality constraints and the inequality constraints, respectively.

If your problem does not have equality (or inequality) constraints, you may pass an empty matrix for *cef* (or *cif*).

If supplied, the equality and inequality constraint functions must be of the form

```
r = f (x)
```

in which *x* is a vector and *r* is a vector.

The third and fourth arguments may also be 2-element cell arrays of function handles. The first element should point to the constraint function and the second should point to a function that computes the gradient of the constraint function:

$$\left(\frac{\partial f(x)}{\partial x_1}, \frac{\partial f(x)}{\partial x_2}, \ldots, \frac{\partial f(x)}{\partial x_N}\right)^T$$

The fifth and sixth arguments are vectors containing lower and upper bounds on *x*. These must be consistent with equality and inequality constraints *g* and *h*. If the bounds are not specified, or are empty, they are set to *-realmax* and *realmax* by default.

The seventh argument is max. number of iterations. If not specified, the default value is 100.

The eighth argument is tolerance for stopping criteria. If not specified, the default value is *eps*.

Here is an example of calling `sqp`:

```
function r = g (x)
  r = [ sumsq(x)-10;
        x(2)*x(3)-5*x(4)*x(5);
        x(1)^3+x(2)^3+1 ];
endfunction

function obj = phi (x)
  obj = exp(prod(x)) - 0.5*(x(1)^3+x(2)^3+1)^2;
endfunction

x0 = [-1.8; 1.7; 1.9; -0.8; -0.8];

[x, obj, info, iter, nf, lambda] = sqp (x0, @phi, @g, [])

x =

  -1.71714
   1.59571
   1.82725
  -0.76364
  -0.76364

obj = 0.053950
info = 101
iter = 8
nf = 10
lambda =
```

```
-0.0401627
 0.0379578
-0.0052227
```

The value returned in *info* may be one of the following:

101 The algorithm terminated because the norm of the last step was less than `tol * norm (x))` (the value of tol is currently fixed at `sqrt (eps)`—edit '`sqp.m`' to modify this value.

102 The BFGS update failed.

103 The maximum number of iterations was reached (the maximum number of allowed iterations is currently fixed at 100—edit '`sqp.m`' to increase this value).

See also: [qp], page 407.

24.4 Linear Least Squares

Octave also supports linear least squares minimization. That is, Octave can find the parameter b such that the model $y = xb$ fits data (x, y) as well as possible, assuming zero-mean Gaussian noise. If the noise is assumed to be isotropic the problem can be solved using the '\' or '/' operators, or the `ols` function. In the general case where the noise is assumed to be anisotropic the `gls` is needed.

`[beta, sigma, r] = ols (y, x)` [Function File]

Ordinary least squares estimation for the multivariate model $y = xb + e$ with $\bar{e} = 0$, and $\text{cov}(\text{vec}(e)) = \text{kron}\ (s, I)$ where y is a $t \times p$ matrix, x is a $t \times k$ matrix, b is a $k \times p$ matrix, and e is a $t \times p$ matrix.

Each row of y and x is an observation and each column a variable.

The return values *beta*, *sigma*, and *r* are defined as follows.

beta The OLS estimator for b, `beta = pinv (x) * y`, where `pinv (x)` denotes the pseudoinverse of x.

sigma The OLS estimator for the matrix s,

```
sigma = (y-x*beta)'
  * (y-x*beta)
  / (t-rank(x))
```

r The matrix of OLS residuals, `r = y - x * beta`.

`[beta, v, r] = gls (y, x, o)` [Function File]

Generalized least squares estimation for the multivariate model $y = xb + e$ with $\bar{e} = 0$ and $\text{cov}(\text{vec}(e)) = (s^2)o$, where y is a $t \times p$ matrix, x is a $t \times k$ matrix, b is a $k \times p$ matrix, e is a $t \times p$ matrix, and o is a $tp \times tp$ matrix.

Each row of y and x is an observation and each column a variable. The return values *beta*, *v*, and *r* are defined as follows.

beta The GLS estimator for b.

v The GLS estimator for s^2.

r The matrix of GLS residuals, $r = y - xbeta$.

`x = lsqnonneg (c, d)` [Function File]
`x = lsqnonneg (c, d, x0)` [Function File]
`[x, resnorm] = lsqnonneg (...)` [Function File]
`[x, resnorm, residual] = lsqnonneg (...)` [Function File]
`[x, resnorm, residual, exitflag] = lsqnonneg (...)` [Function File]
`[x, resnorm, residual, exitflag, output] = lsqnonneg (...)` [Function File]
`[x, resnorm, residual, exitflag, output, lambda] = lsqnonneg (...)` [Function File]

Minimize `norm (c*x-d)` subject to `x >= 0`. *c* and *d* must be real. *x0* is an optional initial guess for *x*.

Outputs:

- resnorm

  The squared 2-norm of the residual: norm(*c**x-*d*)^2
- residual

  The residual: *d*-*c**x
- exitflag

  An indicator of convergence. 0 indicates that the iteration count was exceeded, and therefore convergence was not reached; >0 indicates that the algorithm converged. (The algorithm is stable and will converge given enough iterations.)
- output

  A structure with two fields:
  - "algorithm": The algorithm used ("nnls")
  - "iterations": The number of iterations taken.
- lambda

  Not implemented.

See also: [optimset], page 411.

`optimset ()` [Function File]
`optimset (par, val, ...)` [Function File]
`optimset (old, par, val, ...)` [Function File]
`optimset (old, new)` [Function File]

Create options struct for optimization functions.

`optimget (options, parname)` [Function File]
`optimget (options, parname, default)` [Function File]

Return a specific option from a structure created by `optimset`. If *parname* is not a field of the *options* structure, return *default* if supplied, otherwise return an empty matrix.

25 Statistics

Octave has support for various statistical methods. This includes basic descriptive statistics, statistical tests, random number generation, and much more.

The functions that analyze data all assume that multidimensional data is arranged in a matrix where each row is an observation, and each column is a variable. So, the matrix defined by

```
a = [ 0.9, 0.7;
      0.1, 0.1;
      0.5, 0.4 ];
```

contains three observations from a two-dimensional distribution. While this is the default data arrangement, most functions support different arrangements.

It should be noted that the statistics functions don't test for data containing NaN, NA, or Inf. Such values need to be handled explicitly.

25.1 Descriptive Statistics

Octave can compute various statistics such as the moments of a data set.

`mean` (*x*, *dim*, *opt*) [Function File]

If *x* is a vector, compute the mean of the elements of *x*

$$\text{mean}(x) = \bar{x} = \frac{1}{N}\sum_{i=1}^{N} x_i$$

If *x* is a matrix, compute the mean for each column and return them in a row vector.

With the optional argument *opt*, the kind of mean computed can be selected. The following options are recognized:

`"a"` Compute the (ordinary) arithmetic mean. This is the default.

`"g"` Compute the geometric mean.

`"h"` Compute the harmonic mean.

If the optional argument *dim* is supplied, work along dimension *dim*.

Both *dim* and *opt* are optional. If both are supplied, either may appear first.

`median` (*x*, *dim*) [Function File]

If *x* is a vector, compute the median value of the elements of *x*. If the elements of *x* are sorted, the median is defined as

$$\text{median}(x) = \begin{cases} x(\lceil N/2 \rceil), & N \text{ odd;} \\ (x(N/2) + x(N/2+1))/2, & N \text{ even.} \end{cases}$$

If *x* is a matrix, compute the median value for each column and return them in a row vector. If the optional *dim* argument is given, operate along this dimension.

See also: [std], page 415, [mean], page 413.

q = `quantile` (*x*, *p*) [Function File]
q = `quantile` (*x*, *p*, *dim*) [Function File]
q = `quantile` (*x*, *p*, *dim*, *method*) [Function File]

For a sample, *x*, calculate the quantiles, *q*, corresponding to the cumulative probability values in *p*. All non-numeric values (NaNs) of *x* are ignored.

If *x* is a matrix, compute the quantiles for each column and return them in a matrix, such that the i-th row of *q* contains the *p*(i)th quantiles of each column of *x*.

The optional argument *dim* determines the dimension along which the percentiles are calculated. If *dim* is omitted, and *x* is a vector or matrix, it defaults to 1 (column wise quantiles). In the instance that *x* is a N-d array, *dim* defaults to the first dimension whose size greater than unity.

The methods available to calculate sample quantiles are the nine methods used by R (http://www.r-project.org/). The default value is METHOD = 5.

Discontinuous sample quantile methods 1, 2, and 3

1. Method 1: Inverse of empirical distribution function.
2. Method 2: Similar to method 1 but with averaging at discontinuities.
3. Method 3: SAS definition: nearest even order statistic.

Continuous sample quantile methods 4 through 9, where p(k) is the linear interpolation function respecting each methods' representative cdf.

4. Method 4: p(k) = k / n. That is, linear interpolation of the empirical cdf.
5. Method 5: p(k) = (k - 0.5) / n. That is a piecewise linear function where the knots are the values midway through the steps of the empirical cdf.
6. Method 6: p(k) = k / (n + 1).
7. Method 7: p(k) = (k - 1) / (n - 1).
8. Method 8: p(k) = (k - 1/3) / (n + 1/3). The resulting quantile estimates are approximately median-unbiased regardless of the distribution of *x*.
9. Method 9: p(k) = (k - 3/8) / (n + 1/4). The resulting quantile estimates are approximately unbiased for the expected order statistics if *x* is normally distributed.

Hyndman and Fan (1996) recommend method 8. Maxima, S, and R (versions prior to 2.0.0) use 7 as their default. Minitab and SPSS use method 6. MATLAB uses method 5.

References:

- Becker, R. A., Chambers, J. M. and Wilks, A. R. (1988) The New S Language. Wadsworth & Brooks/Cole.
- Hyndman, R. J. and Fan, Y. (1996) Sample quantiles in statistical packages, American Statistician, 50, 361–365.
- R: A Language and Environment for Statistical Computing; `http://cran.r-project.org/doc/manuals/fullrefman.pdf`.

y = `prctile` (*x*, *p*) [Function File]
q = `prctile` (*x*, *p*, *dim*) [Function File]

For a sample *x*, compute the quantiles, *y*, corresponding to the cumulative probability values, P, in percent. All non-numeric values (NaNs) of X are ignored.

If *x* is a matrix, compute the percentiles for each column and return them in a matrix, such that the i-th row of *y* contains the p(i)th percentiles of each column of *x*.

The optional argument *dim* determines the dimension along which the percentiles are calculated. If *dim* is omitted, and *x* is a vector or matrix, it defaults to 1 (column wise quantiles). In the instance that *x* is a N-d array, *dim* defaults to the first dimension whose size greater than unity.

`meansq (x)` [Function File]
`meansq (x, dim)` [Function File]

For vector arguments, return the mean square of the values. For matrix arguments, return a row vector containing the mean square of each column. With the optional *dim* argument, returns the mean squared of the values along this dimension.

`std (x)` [Function File]
`std (x, opt)` [Function File]
`std (x, opt, dim)` [Function File]

If *x* is a vector, compute the standard deviation of the elements of *x*.

$$\operatorname{std}(x) = \sigma(x) = \sqrt{\frac{\sum_{i=1}^{N}(x_i - \bar{x})^2}{N-1}}$$

where $\bar{x}$ is the mean value of x. If *x* is a matrix, compute the standard deviation for each column and return them in a row vector.

The argument *opt* determines the type of normalization to use. Valid values are

0: normalizes with $N-1$, provides the square root of best unbiased estimator of the variance [default]

1: normalizes with N, this provides the square root of the second moment around the mean

The third argument *dim* determines the dimension along which the standard deviation is calculated.

See also: [mean], page 413, [median], page 413.

`var (x)` [Function File]

For vector arguments, return the (real) variance of the values. For matrix arguments, return a row vector containing the variance for each column.

The argument *opt* determines the type of normalization to use. Valid values are

0: Normalizes with $N-1$, provides the best unbiased estimator of the variance [default].

1: Normalizes with N, this provides the second moment around the mean.

The third argument *dim* determines the dimension along which the variance is calculated.

`[m, f, c] = mode (x, dim)` [Function File]

Count the most frequently appearing value. `mode` counts the frequency along the first non-singleton dimension and if two or more values have the same frequency returns the smallest of the two in *m*. The dimension along which to count can be specified by the *dim* parameter.

The variable *f* counts the frequency of each of the most frequently occurring elements. The cell array *c* contains all of the elements with the maximum frequency .

`cov (x, y)` [Function File]

Compute covariance.

If each row of *x* and *y* is an observation and each column is a variable, the (*i*, *j*)-th entry of `cov (x, y)` is the covariance between the *i*-th variable in *x* and the *j*-th variable in *y*.

$$\sigma_{ij} = \frac{1}{N-1}\sum_{i=1}^{N}(x_i - \bar{x})(y_i - \bar{y})$$

where $\bar{x}$ and $\bar{y}$ are the mean values of x and y. If called with one argument, compute `cov (x, x)`.

`cor (x, y)` [Function File]

Compute correlation.

The (*i*, *j*)-th entry of `cor (x, y)` is the correlation between the *i*-th variable in *x* and the *j*-th variable in *y*.

$$\text{corrcoef}(x, y) = \frac{\text{cov}(x, y)}{\text{std}(x)\text{std}(y)}$$

For matrices, each row is an observation and each column a variable; vectors are always observations and may be row or column vectors.

`cor (x)` is equivalent to `cor (x, x)`.

Note that the `corrcoef` function does the same as `cor`.

`corrcoef (x, y)` [Function File]

Compute correlation.

If each row of *x* and *y* is an observation and each column is a variable, the (*i*, *j*)-th entry of `corrcoef (x, y)` is the correlation between the *i*-th variable in *x* and the *j*-th variable in *y*.

$$\text{corrcoef}(x, y) = \frac{\text{cov}(x, y)}{\text{std}(x)\text{std}(y)}$$

If called with one argument, compute `corrcoef (x, x)`.

`kurtosis (x, dim)` [Function File]

If *x* is a vector of length N, return the kurtosis

$$\text{kurtosis}(x) = \frac{1}{N\sigma(x)^4}\sum_{i=1}^{N}(x_i - \bar{x})^4 - 3$$

where $\bar{x}$ is the mean value of x.

of *x*. If *x* is a matrix, return the kurtosis over the first non-singleton dimension. The optional argument *dim* can be given to force the kurtosis to be given over that dimension.

`skewness (x, dim)` [Function File]

If *x* is a vector of length n, return the skewness

$$\text{skewness}(x) = \frac{1}{N\sigma(x)^3}\sum_{i=1}^{N}(x_i - \bar{x})^3$$

where $\bar{x}$ is the mean value of x.

of *x*. If *x* is a matrix, return the skewness along the first non-singleton dimension of the matrix. If the optional *dim* argument is given, operate along this dimension.

`statistics (x)` [Function File]

If *x* is a matrix, return a matrix with the minimum, first quartile, median, third quartile, maximum, mean, standard deviation, skewness and kurtosis of the columns of *x* as its columns.

If *x* is a vector, calculate the statistics along the non-singleton dimension.

`moment (x, p, opt, dim)` [Function File]

If *x* is a vector, compute the *p*-th moment of *x*.

If *x* is a matrix, return the row vector containing the *p*-th moment of each column.

With the optional string opt, the kind of moment to be computed can be specified. If opt contains `"c"` or `"a"`, central and/or absolute moments are returned. For example,

```
moment (x, 3, "ac")
```

computes the third central absolute moment of *x*.

If the optional argument *dim* is supplied, work along dimension *dim*.

25.2 Basic Statistical Functions

Octave also supports various helpful statistical functions.

`mahalanobis (x, y)` [Function File]

Return the Mahalanobis' D-square distance between the multivariate samples *x* and *y*, which must have the same number of components (columns), but may have a different number of observations (rows).

`center (x)` [Function File]
`center (x, dim)` [Function File]

If *x* is a vector, subtract its mean. If *x* is a matrix, do the above for each column. If the optional argument *dim* is given, perform the above operation along this dimension

`studentize (x, dim)` [Function File]

If *x* is a vector, subtract its mean and divide by its standard deviation.

If *x* is a matrix, do the above along the first non-singleton dimension. If the optional argument *dim* is given then operate along this dimension.

c = `nchoosek` (*n*, *k*) [Function File]

Compute the binomial coefficient or all combinations of *n*. If *n* is a scalar then, calculate the binomial coefficient of *n* and *k*, defined as

$$\binom{n}{k} = \frac{n(n-1)(n-2)\cdots(n-k+1)}{k!} = \frac{n!}{k!(n-k)!}$$

If *n* is a vector generate all combinations of the elements of *n*, taken *k* at a time, one row per combination. The resulting *c* has size `[nchoosek (length (n), k), k]`.

`nchoosek` works only for non-negative integer arguments; use `bincoeff` for non-integer scalar arguments and for using vector arguments to compute many coefficients at once.

See also: [bincoeff], page 308.

n = `histc` (*y*, *edges*) [Function File]
n = `histc` (*y*, *edges*, *dim*) [Function File]
[*n*, *idx*] = `histc` (...) [Function File]

Produce histogram counts.

When *y* is a vector, the function counts the number of elements of *y* that fall in the histogram bins defined by *edges*. This must be a vector of monotonically non-decreasing values that define the edges of the histogram bins. So, `n (k)` contains the number of elements in *y* for which `edges (k) <= y < edges (k+1)`. The final element of *n* contains the number of elements of *y* that was equal to the last element of *edges*.

When *y* is a *N*-dimensional array, the same operation as above is repeated along dimension *dim*. If this argument is given, the operation is performed along the first non-singleton dimension.

If a second output argument is requested an index matrix is also returned. The *idx* matrix has same size as *y*. Each element of *idx* contains the index of the histogram bin in which the corresponding element of *y* was counted.

See also: [hist], page 209.

`perms` (*v*) [Function File]

Generate all permutations of *v*, one row per permutation. The result has size `factorial (n) * n`, where *n* is the length of *v*.

As an example, `perms([1, 2, 3])` returns the matrix

```
1  2  3
2  1  3
1  3  2
2  3  1
3  1  2
3  2  1
```

`values` (*x*) [Function File]

Return the different values in a column vector, arranged in ascending order.

As an example, `values([1, 2, 3, 1])` returns the vector `[1, 2, 3]`.

`[t, l_x] = table (x)` [Function File]
`[t, l_x, l_y] = table (x, y)` [Function File]

Create a contingency table *t* from data vectors. The *l* vectors are the corresponding levels.

Currently, only 1- and 2-dimensional tables are supported.

`spearman (x, y)` [Function File]

Compute Spearman's rank correlation coefficient *rho* for each of the variables specified by the input arguments.

For matrices, each row is an observation and each column a variable; vectors are always observations and may be row or column vectors.

`spearman (x)` is equivalent to `spearman (x, x)`.

For two data vectors *x* and *y*, Spearman's *rho* is the correlation of the ranks of *x* and *y*.

If *x* and *y* are drawn from independent distributions, *rho* has zero mean and variance `1 / (n - 1)`, and is asymptotically normally distributed.

`run_count (x, n)` [Function File]

Count the upward runs along the first non-singleton dimension of *x* of length 1, 2, . . ., *n*-1 and greater than or equal to *n*. If the optional argument *dim* is given operate along this dimension

`ranks (x, dim)` [Function File]

Return the ranks of *x* along the first non-singleton dimension adjust for ties. If the optional argument *dim* is given, operate along this dimension.

`range (x)` [Function File]
`range (x, dim)` [Function File]

If *x* is a vector, return the range, i.e., the difference between the maximum and the minimum, of the input data.

If *x* is a matrix, do the above for each column of *x*.

If the optional argument *dim* is supplied, work along dimension *dim*.

`probit (p)` [Function File]

For each component of *p*, return the probit (the quantile of the standard normal distribution) of *p*.

`logit (p)` [Function File]

For each component of *p*, return the logit of *p* defined as

$$\mathrm{logit}(p) = \log\left(\frac{p}{1-p}\right)$$

`cloglog (x)` [Function File]

Return the complementary log-log function of *x*, defined as

$$\mathrm{cloglog}(x) = -\log(-\log(x))$$

`kendall (x, y)` [Function File]

Compute Kendall's *tau* for each of the variables specified by the input arguments.

For matrices, each row is an observation and each column a variable; vectors are always observations and may be row or column vectors.

`kendall (x)` is equivalent to `kendall (x, x)`.

For two data vectors *x*, *y* of common length *n*, Kendall's *tau* is the correlation of the signs of all rank differences of *x* and *y*; i.e., if both *x* and *y* have distinct entries, then

$$\tau = \frac{1}{n(n-1)} \sum_{i,j} \mathrm{sign}(q_i - q_j)\mathrm{sign}(r_i - r_j)$$

in which the q_i and r_i are the ranks of *x* and *y*, respectively.

If *x* and *y* are drawn from independent distributions, Kendall's *tau* is asymptotically normal with mean 0 and variance $\frac{2(2n+5)}{9n(n-1)}$.

`iqr (x, dim)` [Function File]

If *x* is a vector, return the interquartile range, i.e., the difference between the upper and lower quartile, of the input data.

If *x* is a matrix, do the above for first non-singleton dimension of *x*. If the option *dim* argument is given, then operate along this dimension.

`cut (x, breaks)` [Function File]

Create categorical data out of numerical or continuous data by cutting into intervals.

If *breaks* is a scalar, the data is cut into that many equal-width intervals. If *breaks* is a vector of break points, the category has `length (breaks) - 1` groups.

The returned value is a vector of the same size as *x* telling which group each point in *x* belongs to. Groups are labelled from 1 to the number of groups; points outside the range of *breaks* are labelled by `NaN`.

25.3 Statistical Plots

Octave can create Quantile Plots (QQ-Plots), and Probability Plots (PP-Plots). These are simple graphical tests for determining if a data set comes from a certain distribution.

Note that Octave can also show histograms of data using the `hist` function as described in Section 15.1.1 [Two-Dimensional Plots], page 205.

`[q, s] = qqplot (x, dist, params)` [Function File]

Perform a QQ-plot (quantile plot).

If F is the CDF of the distribution *dist* with parameters *params* and G its inverse, and *x* a sample vector of length *n*, the QQ-plot graphs ordinate $s(i)$ = i-th largest element of x versus abscissa $q(i\mathrm{f})$ = G((i - 0.5)/n).

If the sample comes from F except for a transformation of location and scale, the pairs will approximately follow a straight line.

The default for *dist* is the standard normal distribution. The optional argument *params* contains a list of parameters of *dist*. For example, for a quantile plot of the uniform distribution on [2,4] and *x*, use

```
qqplot (x, "uniform", 2, 4)
```

dist can be any string for which a function *dist_inv* that calculates the inverse CDF of distribution *dist* exists.

If no output arguments are given, the data are plotted directly.

`[p, y] = ppplot (x, dist, params)` [Function File]

Perform a PP-plot (probability plot).

If F is the CDF of the distribution *dist* with parameters *params* and *x* a sample vector of length *n*, the PP-plot graphs ordinate $y(i)$ = F (*i*-th largest element of *x*) versus abscissa $p(i) = (i$ - $0.5)/n$. If the sample comes from F, the pairs will approximately follow a straight line.

The default for *dist* is the standard normal distribution. The optional argument *params* contains a list of parameters of *dist*. For example, for a probability plot of the uniform distribution on [2,4] and *x*, use

```
ppplot (x, "uniform", 2, 4)
```

dist can be any string for which a function *dist_cdf* that calculates the CDF of distribution *dist* exists.

If no output arguments are given, the data are plotted directly.

25.4 Tests

Octave can perform several different statistical tests. The following table summarizes the available tests.

**Hypothesis**	**Test Functions**
Equal mean values	anova, hotelling_test2, t_test_2, welch_test, wilcoxon_test, z_test_2
Equal medians	kruskal_wallis_test, sign_test
Equal variances	bartlett_test, manova, var_test
Equal distributions	chisquare_test_homogeneity, kolmogorov_smirnov_test_2, u_test
Equal marginal frequencies	mcnemar_test
Equal success probabilities	prop_test_2
Independent observations	chisquare_test_independence, run_test
Uncorrelated observations	cor_test
Given mean value	hotelling_test, t_test, z_test
Observations from distribution	kolmogorov_smirnov_test
Regression	f_test_regression, t_test_regression

The tests return a p-value that describes the outcome of the test. Assuming that the test hypothesis is true, the p-value is the probability of obtaining a worse result than the observed one. So large p-values corresponds to a successful test. Usually a test hypothesis is accepted if the p-value exceeds 0.05.

`[pval, f, df_b, df_w] = anova (y, g)` [Function File]

Perform a one-way analysis of variance (ANOVA). The goal is to test whether the population means of data taken from *k* different groups are all equal.

Data may be given in a single vector *y* with groups specified by a corresponding vector of group labels *g* (e.g., numbers from 1 to *k*). This is the general form which does not impose any restriction on the number of data in each group or the group labels.

If *y* is a matrix and *g* is omitted, each column of *y* is treated as a group. This form is only appropriate for balanced ANOVA in which the numbers of samples from each group are all equal.

Under the null of constant means, the statistic *f* follows an F distribution with *df_b* and *df_w* degrees of freedom.

The p-value (1 minus the CDF of this distribution at *f*) is returned in *pval*.

If no output argument is given, the standard one-way ANOVA table is printed.

`[pval, chisq, df] = bartlett_test (x1, ...)` [Function File]

Perform a Bartlett test for the homogeneity of variances in the data vectors *x1*, *x2*, . . ., *xk*, where $k > 1$.

Under the null of equal variances, the test statistic *chisq* approximately follows a chi-square distribution with *df* degrees of freedom.

The p-value (1 minus the CDF of this distribution at *chisq*) is returned in *pval*.

If no output argument is given, the p-value is displayed.

`[pval, chisq, df] = chisquare_test_homogeneity (x, y, c)` [Function File]

Given two samples *x* and *y*, perform a chisquare test for homogeneity of the null hypothesis that *x* and *y* come from the same distribution, based on the partition induced by the (strictly increasing) entries of *c*.

For large samples, the test statistic *chisq* approximately follows a chisquare distribution with *df* = `length (c)` degrees of freedom.

The p-value (1 minus the CDF of this distribution at *chisq*) is returned in *pval*.

If no output argument is given, the p-value is displayed.

`[pval, chisq, df] = chisquare_test_independence (x)` [Function File]

Perform a chi-square test for independence based on the contingency table *x*. Under the null hypothesis of independence, *chisq* approximately has a chi-square distribution with *df* degrees of freedom.

The p-value (1 minus the CDF of this distribution at chisq) of the test is returned in *pval*.

If no output argument is given, the p-value is displayed.

`cor_test (x, y, alt, method)` [Function File]

Test whether two samples *x* and *y* come from uncorrelated populations.

The optional argument string *alt* describes the alternative hypothesis, and can be `"!="` or `"<>"` (non-zero), `">"` (greater than 0), or `"<"` (less than 0). The default is the two-sided case.

The optional argument string *method* specifies on which correlation coefficient the test should be based. If *method* is `"pearson"` (default), the (usual) Pearson's product moment correlation coefficient is used. In this case, the data should come from a bivariate normal distribution. Otherwise, the other two methods offer nonparametric alternatives. If *method* is `"kendall"`, then Kendall's rank correlation tau is used. If *method* is `"spearman"`, then Spearman's rank correlation rho is used. Only the first character is necessary.

The output is a structure with the following elements:

pval The p-value of the test.

stat The value of the test statistic.

dist The distribution of the test statistic.

params The parameters of the null distribution of the test statistic.

alternative
The alternative hypothesis.

method The method used for testing.

If no output argument is given, the p-value is displayed.

`[pval, f, df_num, df_den] = f_test_regression (y, x, rr, r)` [Function File]

Perform an F test for the null hypothesis rr * b = r in a classical normal regression model y = X * b + e.

Under the null, the test statistic *f* follows an F distribution with *df_num* and *df_den* degrees of freedom.

The p-value (1 minus the CDF of this distribution at *f*) is returned in *pval*.

If not given explicitly, $r = 0$.

If no output argument is given, the p-value is displayed.

`[pval, tsq] = hotelling_test (x, m)` [Function File]

For a sample *x* from a multivariate normal distribution with unknown mean and covariance matrix, test the null hypothesis that `mean (x) == m`.

Hotelling's T^2 is returned in *tsq*. Under the null, $(n-p)T^2/(p(n-1))$ has an F distribution with p and $n-p$ degrees of freedom, where n and p are the numbers of samples and variables, respectively.

The p-value of the test is returned in *pval*.

If no output argument is given, the p-value of the test is displayed.

`[pval, tsq] = hotelling_test_2 (x, y)` [Function File]

For two samples *x* from multivariate normal distributions with the same number of variables (columns), unknown means and unknown equal covariance matrices, test the null hypothesis `mean (x) == mean (y)`.

Hotelling's two-sample T^2 is returned in *tsq*. Under the null,

$$\frac{n_x + n_y - p - 1)T^2}{p(n_x + n_y - 2)}$$

has an F distribution with p and $n_x + n_y - p - 1$ degrees of freedom, where n_x and n_y are the sample sizes and p is the number of variables.

The p-value of the test is returned in *pval*.

If no output argument is given, the p-value of the test is displayed.

`[pval, ks] = kolmogorov_smirnov_test (x, dist, params, alt)` [Function File]

Perform a Kolmogorov-Smirnov test of the null hypothesis that the sample *x* comes from the (continuous) distribution dist. I.e., if F and G are the CDFs corresponding to the sample and dist, respectively, then the null is that F == G.

The optional argument *params* contains a list of parameters of *dist*. For example, to test whether a sample *x* comes from a uniform distribution on [2,4], use

```
kolmogorov_smirnov_test(x, "uniform", 2, 4)
```

dist can be any string for which a function *dist_cdf* that calculates the CDF of distribution *dist* exists.

With the optional argument string *alt*, the alternative of interest can be selected. If *alt* is `"!="` or `"<>"`, the null is tested against the two-sided alternative F != G. In this case, the test statistic *ks* follows a two-sided Kolmogorov-Smirnov distribution. If *alt* is `">"`, the one-sided alternative F `>` G is considered. Similarly for `"<"`, the one-sided alternative F `>` G is considered. In this case, the test statistic *ks* has a one-sided Kolmogorov-Smirnov distribution. The default is the two-sided case.

The p-value of the test is returned in *pval*.

If no output argument is given, the p-value is displayed.

`[pval, ks, d] = kolmogorov_smirnov_test_2 (x, y, alt)` [Function File]

Perform a 2-sample Kolmogorov-Smirnov test of the null hypothesis that the samples *x* and *y* come from the same (continuous) distribution. I.e., if F and G are the CDFs corresponding to the *x* and *y* samples, respectively, then the null is that F == G.

With the optional argument string *alt*, the alternative of interest can be selected. If *alt* is `"!="` or `"<>"`, the null is tested against the two-sided alternative F != G. In this case, the test statistic *ks* follows a two-sided Kolmogorov-Smirnov distribution. If *alt* is `">"`, the one-sided alternative F `>` G is considered. Similarly for `"<"`, the one-sided alternative F `<` G is considered. In this case, the test statistic *ks* has a one-sided Kolmogorov-Smirnov distribution. The default is the two-sided case.

The p-value of the test is returned in *pval*.

The third returned value, *d*, is the test statistic, the maximum vertical distance between the two cumulative distribution functions.

If no output argument is given, the p-value is displayed.

`[pval, k, df] = kruskal_wallis_test (x1, ...)` [Function File]

Perform a Kruskal-Wallis one-factor "analysis of variance".

Suppose a variable is observed for $k > 1$ different groups, and let $x1, \ldots, xk$ be the corresponding data vectors.

Under the null hypothesis that the ranks in the pooled sample are not affected by the group memberships, the test statistic k is approximately chi-square with $df = k - 1$ degrees of freedom.

If the data contains ties (some value appears more than once) k is divided by

1 - *sum_ties* / (*n*^3 - *n*)

where *sum_ties* is the sum of *t*^2 - *t* over each group of ties where *t* is the number of ties in the group and *n* is the total number of values in the input data. For more info on this adjustment see "Use of Ranks in One-Criterion Variance Analysis" in Journal of the American Statistical Association, Vol. 47, No. 260 (Dec 1952) by William H. Kruskal and W. Allen Wallis.

The p-value (1 minus the CDF of this distribution at *k*) is returned in *pval*.

If no output argument is given, the p-value is displayed.

`manova (y, g)` [Function File]

Perform a one-way multivariate analysis of variance (MANOVA). The goal is to test whether the p-dimensional population means of data taken from *k* different groups are all equal. All data are assumed drawn independently from p-dimensional normal distributions with the same covariance matrix.

The data matrix is given by *y*. As usual, rows are observations and columns are variables. The vector *g* specifies the corresponding group labels (e.g., numbers from 1 to *k*).

The LR test statistic (Wilks' Lambda) and approximate p-values are computed and displayed.

`[pval, chisq, df] = mcnemar_test (x)` [Function File]

For a square contingency table *x* of data cross-classified on the row and column variables, McNemar's test can be used for testing the null hypothesis of symmetry of the classification probabilities.

Under the null, *chisq* is approximately distributed as chisquare with *df* degrees of freedom.

The p-value (1 minus the CDF of this distribution at *chisq*) is returned in *pval*.

If no output argument is given, the p-value of the test is displayed.

`[pval, z] = prop_test_2 (x1, n1, x2, n2, alt)` [Function File]

If *x1* and *n1* are the counts of successes and trials in one sample, and *x2* and *n2* those in a second one, test the null hypothesis that the success probabilities *p1* and *p2* are the same. Under the null, the test statistic *z* approximately follows a standard normal distribution.

With the optional argument string *alt*, the alternative of interest can be selected. If *alt* is `"!="` or `"<>"`, the null is tested against the two-sided alternative *p1* != *p2*. If *alt* is `">"`, the one-sided alternative $p1 > p2$ is used. Similarly for `"<"`, the one-sided alternative $p1 < p2$ is used. The default is the two-sided case.

The p-value of the test is returned in *pval*.

If no output argument is given, the p-value of the test is displayed.

[*pval*, *chisq*] = run_test (*x*) [Function File]

Perform a chi-square test with 6 degrees of freedom based on the upward runs in the columns of *x*. Can be used to test whether *x* contains independent data.

The p-value of the test is returned in *pval*.

If no output argument is given, the p-value is displayed.

[*pval*, *b*, *n*] = sign_test (*x*, *y*, *alt*) [Function File]

For two matched-pair samples *x* and *y*, perform a sign test of the null hypothesis PROB (*x* > *y*) == PROB (*x* < *y*) == 1/2. Under the null, the test statistic *b* roughly follows a binomial distribution with parameters `n = sum (x != y)` and *p* = 1/2.

With the optional argument `alt`, the alternative of interest can be selected. If *alt* is `"!="` or `"<>"`, the null hypothesis is tested against the two-sided alternative PROB (*x* < *y*) != 1/2. If *alt* is `">"`, the one-sided alternative PROB (*x* > *y*) > 1/2 ("x is stochastically greater than y") is considered. Similarly for `"<"`, the one-sided alternative PROB (*x* > *y*) < 1/2 ("x is stochastically less than y") is considered. The default is the two-sided case.

The p-value of the test is returned in *pval*.

If no output argument is given, the p-value of the test is displayed.

[*pval*, *t*, *df*] = t_test (*x*, *m*, *alt*) [Function File]

For a sample *x* from a normal distribution with unknown mean and variance, perform a t-test of the null hypothesis `mean (x) == m`. Under the null, the test statistic *t* follows a Student distribution with `df = length (x) - 1` degrees of freedom.

With the optional argument string *alt*, the alternative of interest can be selected. If *alt* is `"!="` or `"<>"`, the null is tested against the two-sided alternative `mean (x) != m`. If *alt* is `">"`, the one-sided alternative `mean (x) > m` is considered. Similarly for `"<"`, the one-sided alternative `mean (x) < m` is considered. The default is the two-sided case.

The p-value of the test is returned in *pval*.

If no output argument is given, the p-value of the test is displayed.

[*pval*, *t*, *df*] = t_test_2 (*x*, *y*, *alt*) [Function File]

For two samples x and y from normal distributions with unknown means and unknown equal variances, perform a two-sample t-test of the null hypothesis of equal means. Under the null, the test statistic *t* follows a Student distribution with *df* degrees of freedom.

With the optional argument string *alt*, the alternative of interest can be selected. If *alt* is `"!="` or `"<>"`, the null is tested against the two-sided alternative `mean (x) != mean (y)`. If *alt* is `">"`, the one-sided alternative `mean (x) > mean (y)` is used. Similarly for `"<"`, the one-sided alternative `mean (x) < mean (y)` is used. The default is the two-sided case.

The p-value of the test is returned in *pval*.

If no output argument is given, the p-value of the test is displayed.

[*pval*, *t*, *df*] = t_test_regression (*y*, *x*, *rr*, *r*, *alt*) [Function File]

Perform an t test for the null hypothesis *rr* * *b* = *r* in a classical normal regression model *y* = *x* * *b* + *e*. Under the null, the test statistic *t* follows a *t* distribution with *df* degrees of freedom.

If *r* is omitted, a value of 0 is assumed.

With the optional argument string *alt*, the alternative of interest can be selected. If *alt* is "!=" or "<>", the null is tested against the two-sided alternative *rr* * *b* != *r*. If *alt* is ">", the one-sided alternative *rr* * *b* > *r* is used. Similarly for "<", the one-sided alternative *rr* * *b* < *r* is used. The default is the two-sided case.

The p-value of the test is returned in *pval*.

If no output argument is given, the p-value of the test is displayed.

[*pval*, *z*] = u_test (*x*, *y*, *alt*) [Function File]

For two samples *x* and *y*, perform a Mann-Whitney U-test of the null hypothesis PROB (*x* > *y*) == 1/2 == PROB (*x* < *y*). Under the null, the test statistic *z* approximately follows a standard normal distribution. Note that this test is equivalent to the Wilcoxon rank-sum test.

With the optional argument string *alt*, the alternative of interest can be selected. If *alt* is "!=" or "<>", the null is tested against the two-sided alternative PROB (*x* > *y*) != 1/2. If *alt* is ">", the one-sided alternative PROB (*x* > *y*) > 1/2 is considered. Similarly for "<", the one-sided alternative PROB (*x* > *y*) < 1/2 is considered. The default is the two-sided case.

The p-value of the test is returned in *pval*.

If no output argument is given, the p-value of the test is displayed.

[*pval*, *f*, *df_num*, *df_den*] = var_test (*x*, *y*, *alt*) [Function File]

For two samples *x* and *y* from normal distributions with unknown means and unknown variances, perform an F-test of the null hypothesis of equal variances. Under the null, the test statistic *f* follows an F-distribution with *df_num* and *df_den* degrees of freedom.

With the optional argument string *alt*, the alternative of interest can be selected. If *alt* is "!=" or "<>", the null is tested against the two-sided alternative var (*x*) != var (*y*). If *alt* is ">", the one-sided alternative var (*x*) > var (*y*) is used. Similarly for "<", the one-sided alternative var (*x*) > var (*y*) is used. The default is the two-sided case.

The p-value of the test is returned in *pval*.

If no output argument is given, the p-value of the test is displayed.

[*pval*, *t*, *df*] = welch_test (*x*, *y*, *alt*) [Function File]

For two samples *x* and *y* from normal distributions with unknown means and unknown and not necessarily equal variances, perform a Welch test of the null hypothesis of equal means. Under the null, the test statistic *t* approximately follows a Student distribution with *df* degrees of freedom.

With the optional argument string *alt*, the alternative of interest can be selected. If *alt* is "!=" or "<>", the null is tested against the two-sided alternative mean (*x*) !=

m. If *alt* is ">", the one-sided alternative mean(x) > *m* is considered. Similarly for "<", the one-sided alternative mean(x) < *m* is considered. The default is the two-sided case.

The p-value of the test is returned in *pval*.

If no output argument is given, the p-value of the test is displayed.

[*pval*, *z*] = wilcoxon_test (*x*, *y*, *alt*) [Function File]

For two matched-pair sample vectors *x* and *y*, perform a Wilcoxon signed-rank test of the null hypothesis PROB (*x* > *y*) == 1/2. Under the null, the test statistic *z* approximately follows a standard normal distribution when *n* > 25.

Warning: This function assumes a normal distribution for *z* and thus is invalid for *n* <= 25.

With the optional argument string *alt*, the alternative of interest can be selected. If *alt* is "!=" or "<>", the null is tested against the two-sided alternative PROB (*x* > *y*) != 1/2. If alt is ">", the one-sided alternative PROB (*x* > *y*) > 1/2 is considered. Similarly for "<", the one-sided alternative PROB (*x* > *y*) < 1/2 is considered. The default is the two-sided case.

The p-value of the test is returned in *pval*.

If no output argument is given, the p-value of the test is displayed.

[*pval*, *z*] = z_test (*x*, *m*, *v*, *alt*) [Function File]

Perform a Z-test of the null hypothesis mean (*x*) == *m* for a sample *x* from a normal distribution with unknown mean and known variance *v*. Under the null, the test statistic *z* follows a standard normal distribution.

With the optional argument string *alt*, the alternative of interest can be selected. If *alt* is "!=" or "<>", the null is tested against the two-sided alternative mean (*x*) != *m*. If *alt* is ">", the one-sided alternative mean (*x*) > *m* is considered. Similarly for "<", the one-sided alternative mean (*x*) < *m* is considered. The default is the two-sided case.

The p-value of the test is returned in *pval*.

If no output argument is given, the p-value of the test is displayed along with some information.

[*pval*, *z*] = z_test_2 (*x*, *y*, *v_x*, *v_y*, *alt*) [Function File]

For two samples *x* and *y* from normal distributions with unknown means and known variances *v_x* and *v_y*, perform a Z-test of the hypothesis of equal means. Under the null, the test statistic *z* follows a standard normal distribution.

With the optional argument string *alt*, the alternative of interest can be selected. If *alt* is "!=" or "<>", the null is tested against the two-sided alternative mean (*x*) != mean (*y*). If alt is ">", the one-sided alternative mean (*x*) > mean (*y*) is used. Similarly for "<", the one-sided alternative mean (*x*) < mean (*y*) is used. The default is the two-sided case.

The p-value of the test is returned in *pval*.

If no output argument is given, the p-value of the test is displayed along with some information.

25.5 Models

[*theta*, *beta*, *dev*, *dl*, *d2l*, *p*] = logistic_regression (*y*, *x*, *print*, *theta*, *beta*) [Function File]

Perform ordinal logistic regression.

Suppose *y* takes values in *k* ordered categories, and let gamma_i (*x*) be the cumulative probability that *y* falls in one of the first *i* categories given the covariate *x*. Then

```
[theta, beta] = logistic_regression (y, x)
```

fits the model

```
logit (gamma_i (x)) = theta_i - beta' * x,   i = 1 ... k-1
```

The number of ordinal categories, *k*, is taken to be the number of distinct values of round (*y*). If *k* equals 2, *y* is binary and the model is ordinary logistic regression. The matrix *x* is assumed to have full column rank.

Given *y* only, theta = logistic_regression (y) fits the model with baseline logit odds only.

The full form is

```
[theta, beta, dev, dl, d2l, gamma]
   = logistic_regression (y, x, print, theta, beta)
```

in which all output arguments and all input arguments except *y* are optional.

Setting *print* to 1 requests summary information about the fitted model to be displayed. Setting *print* to 2 requests information about convergence at each iteration. Other values request no information to be displayed. The input arguments *theta* and *beta* give initial estimates for *theta* and *beta*.

The returned value *dev* holds minus twice the log-likelihood.

The returned values *dl* and *d2l* are the vector of first and the matrix of second derivatives of the log-likelihood with respect to *theta* and *beta*.

p holds estimates for the conditional distribution of *y* given *x*.

25.6 Distributions

Octave has functions for computing the Probability Density Function (PDF), the Cumulative Distribution function (CDF), and the quantile (the inverse of the CDF) of a large number of distributions.

The following table summarizes the supported distributions (in alphabetical order).

Distribution	PDF	CDF	Quantile
Beta	betapdf	betacdf	betainv
Binomial	binopdf	binocdf	binoinv
Cauchy	cauchy_pdf	cauchy_cdf	cauchy_inv
Chi-Square	chi2pdf	chi2cdf	chi2inv
Univariate Discrete	discrete_pdf	discrete_cdf	discrete_inv
Empirical	empirical_pdf	empirical_cdf	empirical_inv
Exponential	exppdf	expcdf	expinv
F	fpdf	fcdf	finv
Gamma	gampdf	gamcdf	gaminv
Geometric	geopdf	geocdf	geoinv
Hypergeometric	hygepdf	hygecdf	hygeinv
Kolmogorov Smirnov	*Not Available*	kolmogorov_ smirnov_cdf	*Not Available*
Laplace	laplace_pdf	laplace_cdf	laplace_inv
Logistic	logistic_pdf	logistic_cdf	logistic_inv
Log-Normal	lognpdf	logncdf	logninv
Pascal	nbinpdf	nbincdf	nbininv
Univariate Normal	normpdf	normcdf	norminv
Poisson	poisspdf	poisscdf	poissinv
t (Student)	tpdf	tcdf	tinv
Univariate Discrete	unidpdf	unidcdf	unidinv
Uniform	unifpdf	unifcdf	unifinv
Weibull	wblpdf	wblcdf	wblinv

`betacdf` (*x*, *a*, *b*) [Function File]

For each element of *x*, returns the CDF at *x* of the beta distribution with parameters *a* and *b*, i.e., PROB (beta (*a*, *b*) `<=` *x*).

`betainv` (*x*, *a*, *b*) [Function File]

For each component of *x*, compute the quantile (the inverse of the CDF) at *x* of the Beta distribution with parameters *a* and *b*.

`betapdf` (*x*, *a*, *b*) [Function File]

For each element of *x*, returns the PDF at *x* of the beta distribution with parameters *a* and *b*.

`binocdf` (*x*, *n*, *p*) [Function File]

For each element of *x*, compute the CDF at *x* of the binomial distribution with parameters *n* and *p*.

`binoinv` (*x*, *n*, *p*) [Function File]

For each element of *x*, compute the quantile at *x* of the binomial distribution with parameters *n* and *p*.

`binopdf` (*x*, *n*, *p*) [Function File]

For each element of *x*, compute the probability density function (PDF) at *x* of the binomial distribution with parameters *n* and *p*.

`cauchy_cdf` (*x*, *lambda*, *sigma*) [Function File]
For each element of *x*, compute the cumulative distribution function (CDF) at *x* of the Cauchy distribution with location parameter *lambda* and scale parameter *sigma*. Default values are *lambda* = 0, *sigma* = 1.

`cauchy_inv` (*x*, *lambda*, *sigma*) [Function File]
For each element of *x*, compute the quantile (the inverse of the CDF) at *x* of the Cauchy distribution with location parameter *lambda* and scale parameter *sigma*. Default values are *lambda* = 0, *sigma* = 1.

`cauchy_pdf` (*x*, *lambda*, *sigma*) [Function File]
For each element of *x*, compute the probability density function (PDF) at *x* of the Cauchy distribution with location parameter *lambda* and scale parameter *sigma* > 0. Default values are *lambda* = 0, *sigma* = 1.

`chi2cdf` (*x*, *n*) [Function File]
For each element of *x*, compute the cumulative distribution function (CDF) at *x* of the chisquare distribution with *n* degrees of freedom.

`chi2inv` (*x*, *n*) [Function File]
For each element of *x*, compute the quantile (the inverse of the CDF) at *x* of the chisquare distribution with *n* degrees of freedom.

`chisquare_pdf` (*x*, *n*) [Function File]
For each element of *x*, compute the probability density function (PDF) at *x* of the chisquare distribution with *n* degrees of freedom.

`discrete_cdf` (*x*, *v*, *p*) [Function File]
For each element of *x*, compute the cumulative distribution function (CDF) at *x* of a univariate discrete distribution which assumes the values in *v* with probabilities *p*.

`discrete_inv` (*x*, *v*, *p*) [Function File]
For each component of *x*, compute the quantile (the inverse of the CDF) at *x* of the univariate distribution which assumes the values in *v* with probabilities *p*.

`discrete_pdf` (*x*, *v*, *p*) [Function File]
For each element of *x*, compute the probability density function (PDF) at *x* of a univariate discrete distribution which assumes the values in *v* with probabilities *p*.

`empirical_cdf` (*x*, *data*) [Function File]
For each element of *x*, compute the cumulative distribution function (CDF) at *x* of the empirical distribution obtained from the univariate sample *data*.

`empirical_inv` (*x*, *data*) [Function File]
For each element of *x*, compute the quantile (the inverse of the CDF) at *x* of the empirical distribution obtained from the univariate sample *data*.

`empirical_pdf` (*x*, *data*) [Function File]
For each element of *x*, compute the probability density function (PDF) at *x* of the empirical distribution obtained from the univariate sample *data*.

`expcdf (x, lambda)` [Function File]

For each element of *x*, compute the cumulative distribution function (CDF) at *x* of the exponential distribution with mean *lambda*.

The arguments can be of common size or scalar.

`expinv (x, lambda)` [Function File]

For each element of *x*, compute the quantile (the inverse of the CDF) at *x* of the exponential distribution with mean *lambda*.

`exppdf (x, lambda)` [Function File]

For each element of *x*, compute the probability density function (PDF) of the exponential distribution with mean *lambda*.

`fcdf (x, m, n)` [Function File]

For each element of *x*, compute the CDF at *x* of the F distribution with *m* and *n* degrees of freedom, i.e., PROB (F (*m*, *n*) <= *x*).

`finv (x, m, n)` [Function File]

For each component of *x*, compute the quantile (the inverse of the CDF) at *x* of the F distribution with parameters *m* and *n*.

`fpdf (x, m, n)` [Function File]

For each element of *x*, compute the probability density function (PDF) at *x* of the F distribution with *m* and *n* degrees of freedom.

`gamcdf (x, a, b)` [Function File]

For each element of *x*, compute the cumulative distribution function (CDF) at *x* of the Gamma distribution with parameters *a* and *b*.

See also: [gamma], page 309, [gammaln], page 311, [gammainc], page 309, [gampdf], page 432, [gaminv], page 432, [gamrnd], page 438.

`gaminv (x, a, b)` [Function File]

For each component of *x*, compute the quantile (the inverse of the CDF) at *x* of the Gamma distribution with parameters *a* and *b*.

See also: [gamma], page 309, [gammaln], page 311, [gammainc], page 309, [gampdf], page 432, [gamcdf], page 432, [gamrnd], page 438.

`gampdf (x, a, b)` [Function File]

For each element of *x*, return the probability density function (PDF) at *x* of the Gamma distribution with parameters *a* and *b*.

See also: [gamma], page 309, [gammaln], page 311, [gammainc], page 309, [gamcdf], page 432, [gaminv], page 432, [gamrnd], page 438.

`geocdf (x, p)` [Function File]

For each element of *x*, compute the CDF at *x* of the geometric distribution with parameter *p*.

`geoinv (x, p)` [Function File]

For each element of *x*, compute the quantile at *x* of the geometric distribution with parameter *p*.

`geopdf (x, p)` [Function File]

For each element of *x*, compute the probability density function (PDF) at *x* of the geometric distribution with parameter *p*.

`hygecdf (x, t, m, n)` [Function File]

Compute the cumulative distribution function (CDF) at *x* of the hypergeometric distribution with parameters *t*, *m*, and *n*. This is the probability of obtaining not more than *x* marked items when randomly drawing a sample of size *n* without replacement from a population of total size *t* containing *m* marked items.

The parameters *t*, *m*, and *n* must positive integers with *m* and *n* not greater than *t*.

`hygeinv (x, t, m, n)` [Function File]

For each element of *x*, compute the quantile at *x* of the hypergeometric distribution with parameters *t*, *m*, and *n*.

The parameters *t*, *m*, and *n* must positive integers with *m* and *n* not greater than *t*.

`hygepdf (x, t, m, n)` [Function File]

Compute the probability density function (PDF) at *x* of the hypergeometric distribution with parameters *t*, *m*, and *n*. This is the probability of obtaining *x* marked items when randomly drawing a sample of size *n* without replacement from a population of total size *t* containing *m* marked items.

The arguments must be of common size or scalar.

`kolmogorov_smirnov_cdf (x, tol)` [Function File]

Return the CDF at *x* of the Kolmogorov-Smirnov distribution,

$$Q(x) = \sum_{k=-\infty}^{\infty} (-1)^k \exp(-2k^2x^2)$$

for *x* > 0.

The optional parameter *tol* specifies the precision up to which the series should be evaluated; the default is *tol* = `eps`.

`laplace_cdf (x)` [Function File]

For each element of *x*, compute the cumulative distribution function (CDF) at *x* of the Laplace distribution.

`laplace_inv (x)` [Function File]

For each element of *x*, compute the quantile (the inverse of the CDF) at *x* of the Laplace distribution.

`laplace_pdf (x)` [Function File]

For each element of *x*, compute the probability density function (PDF) at *x* of the Laplace distribution.

`logistic_cdf (x)` [Function File]

For each component of *x*, compute the CDF at *x* of the logistic distribution.

`logistic_inv (x)` [Function File]

For each component of *x*, compute the quantile (the inverse of the CDF) at *x* of the logistic distribution.

`logistic_pdf (x)` [Function File]

For each component of *x*, compute the PDF at *x* of the logistic distribution.

`logncdf (x, mu, sigma)` [Function File]

For each element of *x*, compute the cumulative distribution function (CDF) at *x* of the lognormal distribution with parameters *mu* and *sigma*. If a random variable follows this distribution, its logarithm is normally distributed with mean *mu* and standard deviation *sigma*.

Default values are *mu* = 1, *sigma* = 1.

`logninv (x, mu, sigma)` [Function File]

For each element of *x*, compute the quantile (the inverse of the CDF) at *x* of the lognormal distribution with parameters *mu* and *sigma*. If a random variable follows this distribution, its logarithm is normally distributed with mean `log (mu)` and variance *sigma*.

Default values are *mu* = 1, *sigma* = 1.

`lognpdf (x, mu, sigma)` [Function File]

For each element of *x*, compute the probability density function (PDF) at *x* of the lognormal distribution with parameters *mu* and *sigma*. If a random variable follows this distribution, its logarithm is normally distributed with mean *mu* and standard deviation *sigma*.

Default values are *mu* = 1, *sigma* = 1.

`nbincdf (x, n, p)` [Function File]

For each element of *x*, compute the CDF at x of the Pascal (negative binomial) distribution with parameters *n* and *p*.

The number of failures in a Bernoulli experiment with success probability *p* before the *n*-th success follows this distribution.

`nbininv (x, n, p)` [Function File]

For each element of *x*, compute the quantile at *x* of the Pascal (negative binomial) distribution with parameters *n* and *p*.

The number of failures in a Bernoulli experiment with success probability *p* before the *n*-th success follows this distribution.

`nbinpdf (x, n, p)` [Function File]

For each element of *x*, compute the probability density function (PDF) at *x* of the Pascal (negative binomial) distribution with parameters *n* and *p*.

The number of failures in a Bernoulli experiment with success probability *p* before the *n*-th success follows this distribution.

`normcdf (x, m, s)` [Function File]

For each element of *x*, compute the cumulative distribution function (CDF) at *x* of the normal distribution with mean *m* and standard deviation *s*.

Default values are $m = 0$, $s = 1$.

`norminv` (*x*, *m*, *s*) [Function File]
: For each element of *x*, compute the quantile (the inverse of the CDF) at *x* of the normal distribution with mean *m* and standard deviation *s*.

  Default values are $m = 0$, $s = 1$.

`normpdf` (*x*, *m*, *s*) [Function File]
: For each element of *x*, compute the probability density function (PDF) at *x* of the normal distribution with mean *m* and standard deviation *s*.

  Default values are $m = 0$, $s = 1$.

`poisscdf` (*x*, *lambda*) [Function File]
: For each element of *x*, compute the cumulative distribution function (CDF) at *x* of the Poisson distribution with parameter lambda.

`poissinv` (*x*, *lambda*) [Function File]
: For each component of *x*, compute the quantile (the inverse of the CDF) at *x* of the Poisson distribution with parameter *lambda*.

`poisspdf` (*x*, *lambda*) [Function File]
: For each element of *x*, compute the probability density function (PDF) at *x* of the poisson distribution with parameter *lambda*.

`tcdf` (*x*, *n*) [Function File]
: For each element of *x*, compute the cumulative distribution function (CDF) at *x* of the t (Student) distribution with *n* degrees of freedom, i.e., PROB (t(*n*) <= *x*).

`tinv` (*x*, *n*) [Function File]
: For each probability value *x*, compute the inverse of the cumulative distribution function (CDF) of the t (Student) distribution with degrees of freedom *n*. This function is analogous to looking in a table for the t-value of a single-tailed distribution.

`tpdf` (*x*, *n*) [Function File]
: For each element of *x*, compute the probability density function (PDF) at *x* of the *t* (Student) distribution with *n* degrees of freedom.

`unidcdf` (*x*, *v*) [Function File]
: For each element of *x*, compute the cumulative distribution function (CDF) at *x* of a univariate discrete distribution which assumes the values in *v* with equal probability.

`unidinv` (*x*, *v*) [Function File]
: For each component of *x*, compute the quantile (the inverse of the CDF) at *x* of the univariate discrete distribution which assumes the values in *v* with equal probability

`unidpdf` (*x*, *v*) [Function File]
: For each element of *x*, compute the probability density function (PDF) at *x* of a univariate discrete distribution which assumes the values in *v* with equal probability.

`unifcdf` (*x*, *a*, *b*) [Function File]
: Return the CDF at *x* of the uniform distribution on [*a*, *b*], i.e., PROB (uniform (*a*, *b*) <= x).

  Default values are $a = 0$, $b = 1$.

`unifinv (x, a, b)` [Function File]

For each element of *x*, compute the quantile (the inverse of the CDF) at *x* of the uniform distribution on [*a*, *b*].

Default values are $a = 0$, $b = 1$.

`unifpdf (x, a, b)` [Function File]

For each element of *x*, compute the PDF at *x* of the uniform distribution on [*a*, *b*].

Default values are $a = 0$, $b = 1$.

`wblcdf (x, scale, shape)` [Function File]

Compute the cumulative distribution function (CDF) at *x* of the Weibull distribution with shape parameter *scale* and scale parameter *shape*, which is

$$1 - \exp(-(x/shape)^{scale})$$

for $x \geq 0$.

`wblinv (x, scale, shape)` [Function File]

Compute the quantile (the inverse of the CDF) at *x* of the Weibull distribution with shape parameter *scale* and scale parameter *shape*.

`wblpdf (x, scale, shape)` [Function File]

Compute the probability density function (PDF) at *x* of the Weibull distribution with shape parameter *scale* and scale parameter *shape* which is given by

$$scale \cdot shape^{-scale} x^{scale-1} \exp(-(x/shape)^{scale})$$

for $x > 0$.

25.7 Random Number Generation

Octave can generate random numbers from a large number of distributions. The random number generators are based on the random number generators described in Section 16.4 [Special Utility Matrices], page 284.

The following table summarizes the available random number generators (in alphabetical order).

**Distribution**	**Function**
Beta Distribution	betarnd
Binomial Distribution	binornd
Cauchy Distribution	cauchy_rnd
Chi-Square Distribution	chi2rnd
Univariate Discrete Distribution	discrete_rnd
Empirical Distribution	empirical_rnd
Exponential Distribution	exprnd
F Distribution	frnd
Gamma Distribution	gamrnd
Geometric Distribution	geornd
Hypergeometric Distribution	hygernd
Laplace Distribution	laplace_rnd
Logistic Distribution	logistic_rnd
Log-Normal Distribution	lognrnd
Pascal Distribution	nbinrnd
Univariate Normal Distribution	normrnd
Poisson Distribution	poissrnd
t (Student) Distribution	trnd
Univariate Discrete Distribution	unidrnd
Uniform Distribution	unifrnd
Weibull Distribution	wblrnd
Wiener Process	wienrnd

`betarnd (a, b, r, c)` [Function File]
`betarnd (a, b, sz)` [Function File]

Return an *r* by *c* or `size (sz)` matrix of random samples from the Beta distribution with parameters *a* and *b*. Both *a* and *b* must be scalar or of size *r* by *c*.

If *r* and *c* are omitted, the size of the result matrix is the common size of *a* and *b*.

`binornd (n, p, r, c)` [Function File]
`binornd (n, p, sz)` [Function File]

Return an *r* by *c* or a `size (sz)` matrix of random samples from the binomial distribution with parameters *n* and *p*. Both *n* and *p* must be scalar or of size *r* by *c*.

If *r* and *c* are omitted, the size of the result matrix is the common size of *n* and *p*.

`cauchy_rnd (lambda, sigma, r, c)` [Function File]
`cauchy_rnd (lambda, sigma, sz)` [Function File]

Return an *r* by *c* or a `size (sz)` matrix of random samples from the Cauchy distribution with parameters *lambda* and *sigma* which must both be scalar or of size *r* by *c*.

If *r* and *c* are omitted, the size of the result matrix is the common size of *lambda* and *sigma*.

`chi2rnd` (*n*, *r*, *c*) [Function File]
`chi2rnd` (*n*, *sz*) [Function File]

Return an *r* by *c* or a `size (sz)` matrix of random samples from the chisquare distribution with *n* degrees of freedom. *n* must be a scalar or of size *r* by *c*.

If *r* and *c* are omitted, the size of the result matrix is the size of *n*.

`discrete_rnd` (*n*, *v*, *p*) [Function File]
`discrete_rnd` (*v*, *p*, *r*, *c*) [Function File]
`discrete_rnd` (*v*, *p*, *sz*) [Function File]

Generate a row vector containing a random sample of size *n* from the univariate distribution which assumes the values in *v* with probabilities *p*. *n* must be a scalar.

If *r* and *c* are given create a matrix with *r* rows and *c* columns. Or if *sz* is a vector, create a matrix of size *sz*.

`empirical_rnd` (*n*, *data*) [Function File]
`empirical_rnd` (*data*, *r*, *c*) [Function File]
`empirical_rnd` (*data*, *sz*) [Function File]

Generate a bootstrap sample of size *n* from the empirical distribution obtained from the univariate sample *data*.

If *r* and *c* are given create a matrix with *r* rows and *c* columns. Or if *sz* is a vector, create a matrix of size *sz*.

`exprnd` (*lambda*, *r*, *c*) [Function File]
`exprnd` (*lambda*, *sz*) [Function File]

Return an *r* by *c* matrix of random samples from the exponential distribution with mean *lambda*, which must be a scalar or of size *r* by *c*. Or if *sz* is a vector, create a matrix of size *sz*.

If *r* and *c* are omitted, the size of the result matrix is the size of *lambda*.

`frnd` (*m*, *n*, *r*, *c*) [Function File]
`frnd` (*m*, *n*, *sz*) [Function File]

Return an *r* by *c* matrix of random samples from the F distribution with *m* and *n* degrees of freedom. Both *m* and *n* must be scalar or of size *r* by *c*. If *sz* is a vector the random samples are in a matrix of size *sz*.

If *r* and *c* are omitted, the size of the result matrix is the common size of *m* and *n*.

`gamrnd` (*a*, *b*, *r*, *c*) [Function File]
`gamrnd` (*a*, *b*, *sz*) [Function File]

Return an *r* by *c* or a `size (sz)` matrix of random samples from the Gamma distribution with parameters *a* and *b*. Both *a* and *b* must be scalar or of size *r* by *c*.

If *r* and *c* are omitted, the size of the result matrix is the common size of *a* and *b*.

See also: [gamma], page 309, [gammaln], page 311, [gammainc], page 309, [gampdf], page 432, [gamcdf], page 432, [gaminv], page 432.

georndd (*p*, *r*, *c*) [Function File]
geornd (*p*, *sz*) [Function File]

Return an *r* by *c* matrix of random samples from the geometric distribution with parameter *p*, which must be a scalar or of size *r* by *c*.

If *r* and *c* are given create a matrix with *r* rows and *c* columns. Or if *sz* is a vector, create a matrix of size *sz*.

hygernd (*t*, *m*, *n*, *r*, *c*) [Function File]
hygernd (*t*, *m*, *n*, *sz*) [Function File]
hygernd (*t*, *m*, *n*) [Function File]

Return an *r* by *c* matrix of random samples from the hypergeometric distribution with parameters *t*, *m*, and *n*.

The parameters *t*, *m*, and *n* must positive integers with *m* and *n* not greater than *t*.

The parameter *sz* must be scalar or a vector of matrix dimensions. If *sz* is scalar, then a *sz* by *sz* matrix of random samples is generated.

laplace_rnd (*r*, *c*) [Function File]
laplace_rnd (*sz*)*;* [Function File]

Return an *r* by *c* matrix of random numbers from the Laplace distribution. Or if *sz* is a vector, create a matrix of *sz*.

logistic_rnd (*r*, *c*) [Function File]
logistic_rnd (*sz*) [Function File]

Return an *r* by *c* matrix of random numbers from the logistic distribution. Or if *sz* is a vector, create a matrix of *sz*.

lognrnd (*mu*, *sigma*, *r*, *c*) [Function File]
lognrnd (*mu*, *sigma*, *sz*) [Function File]

Return an *r* by *c* matrix of random samples from the lognormal distribution with parameters *mu* and *sigma*. Both *mu* and *sigma* must be scalar or of size *r* by *c*. Or if *sz* is a vector, create a matrix of size *sz*.

If *r* and *c* are omitted, the size of the result matrix is the common size of *mu* and *sigma*.

nbinrnd (*n*, *p*, *r*, *c*) [Function File]
nbinrnd (*n*, *p*, *sz*) [Function File]

Return an *r* by *c* matrix of random samples from the Pascal (negative binomial) distribution with parameters *n* and *p*. Both *n* and *p* must be scalar or of size *r* by *c*.

If *r* and *c* are omitted, the size of the result matrix is the common size of *n* and *p*. Or if *sz* is a vector, create a matrix of size *sz*.

normrnd (*m*, *s*, *r*, *c*) [Function File]
normrnd (*m*, *s*, *sz*) [Function File]

Return an *r* by *c* or `size (sz)` matrix of random samples from the normal distribution with parameters mean *m* and standard deviation *s*. Both *m* and *s* must be scalar or of size *r* by *c*.

If *r* and *c* are omitted, the size of the result matrix is the common size of *m* and *s*.

`poissrnd (lambda, r, c)` [Function File]

Return an *r* by *c* matrix of random samples from the Poisson distribution with parameter *lambda*, which must be a scalar or of size *r* by *c*.

If *r* and *c* are omitted, the size of the result matrix is the size of *lambda*.

`trnd (n, r, c)` [Function File]
`trnd (n, sz)` [Function File]

Return an *r* by *c* matrix of random samples from the t (Student) distribution with *n* degrees of freedom. *n* must be a scalar or of size *r* by *c*. Or if *sz* is a vector create a matrix of size *sz*.

If *r* and *c* are omitted, the size of the result matrix is the size of *n*.

`unidrnd (mx);` [Function File]
`unidrnd (mx, v);` [Function File]
`unidrnd (mx, m, n, ...);` [Function File]

Return random values from discrete uniform distribution, with maximum value(s) given by the integer *mx*, which may be a scalar or multidimensional array.

If *mx* is a scalar, the size of the result is specified by the vector *v*, or by the optional arguments *m*, *n*, ... Otherwise, the size of the result is the same as the size of *mx*.

`unifrnd (a, b, r, c)` [Function File]
`unifrnd (a, b, sz)` [Function File]

Return an *r* by *c* or a `size (sz)` matrix of random samples from the uniform distribution on [*a*, *b*]. Both *a* and *b* must be scalar or of size *r* by *c*.

If *r* and *c* are omitted, the size of the result matrix is the common size of *a* and *b*.

`wblrnd (scale, shape, r, c)` [Function File]
`wblrnd (scale, shape, sz)` [Function File]

Return an *r* by *c* matrix of random samples from the Weibull distribution with parameters *scale* and *shape* which must be scalar or of size *r* by *c*. Or if *sz* is a vector return a matrix of size *sz*.

If *r* and *c* are omitted, the size of the result matrix is the common size of *alpha* and *sigma*.

`wienrnd (t, d, n)` [Function File]

Return a simulated realization of the *d*-dimensional Wiener Process on the interval [0, *t*]. If *d* is omitted, $d = 1$ is used. The first column of the return matrix contains time, the remaining columns contain the Wiener process.

The optional parameter *n* gives the number of summands used for simulating the process over an interval of length 1. If *n* is omitted, $n = 1000$ is used.

26 Sets

Octave has a limited number of functions for managing sets of data, where a set is defined as a collection of unique elements. In Octave a set is represented as a vector of numbers.

`unique (x)` [Function File]
`unique (x, "rows")` [Function File]
`unique (..., "first")` [Function File]
`unique (..., "last")` [Function File]
`[y, i, j] = unique (...)` [Function File]

Return the unique elements of *x*, sorted in ascending order. If *x* is a row vector, return a row vector, but if *x* is a column vector or a matrix return a column vector.

If the optional argument `"rows"` is supplied, return the unique rows of *x*, sorted in ascending order.

If requested, return index vectors *i* and *j* such that `x(i)==y` and `y(j)==x`.

Additionally, one of `"first"` or `"last"` may be given as an argument. If `"last"` is specified, return the highest possible indices in *i*, otherwise, if `"first"` is specified, return the lowest. The default is `"last"`.

See also: [union], page 442, [intersect], page 442, [setdiff], page 442, [setxor], page 443, [ismember], page 441.

26.1 Set Operations

Octave supports the basic set operations. That is, Octave can compute the union, intersection, complement, and difference of two sets. Octave also supports the *Exclusive Or* set operation, and membership determination. The functions for set operations all work in pretty much the same way. As an example, assume that `x` and `y` contains two sets, then

```
union(x, y)
```

computes the union of the two sets.

`[tf = ismember (A, S)` [Function File]
`[tf, S_idx] = ismember (A, S)` [Function File]
`[tf, S_idx] = ismember (A, S, "rows")` [Function File]

Return a matrix *tf* with the same shape as *A* which has a 1 if `A(i,j)` is in *S* and 0 if it is not. If a second output argument is requested, the index into *S* of each of the matching elements is also returned.

```
a = [3, 10, 1];
s = [0:9];
[tf, s_idx] = ismember (a, s);
     ⇒ tf = [1, 0, 1]
     ⇒ s_idx = [4, 0, 2]
```

The inputs, *A* and *S*, may also be cell arrays.

```
a = {'abc'};
s = {'abc', 'def'};
[tf, s_idx] = ismember (a, s);
     ⇒ tf = [1, 0]
     ⇒ s_idx = [1, 0]
```

With the optional third argument "rows", and matrices *A* and *S* with the same number of columns, compare rows in *A* with the rows in *S*.

```
a = [1:3; 5:7; 4:6];
s = [0:2; 1:3; 2:4; 3:5; 4:6];
[tf, s_idx] = ismember(a, s, 'rows');
     ⇒ tf = logical ([1; 0; 1])
     ⇒ s_idx = [2; 0; 5];
```

See also: [unique], page 441, [union], page 442, [intersect], page 442, [setxor], page 443, [setdiff], page 442.

`union` (*a*, *b*) [Function File]
`union` (*a*, *b*, "*rows*") [Function File]

Return the set of elements that are in either of the sets *a* and *b*. For example,

```
union ([1, 2, 4], [2, 3, 5])
     ⇒ [1, 2, 3, 4, 5]
```

If the optional third input argument is the string "rows" each row of the matrices *a* and *b* will be considered an element of sets. For example,

```
union([1, 2; 2, 3], [1, 2; 3, 4], "rows")
     ⇒  1   2
    2   3
    3   4
```

`[c, ia, ib] = union` (*a*, *b*) [Function File]

Return index vectors *ia* and *ib* such that `a == c(ia)` and `b == c(ib)`.

See also: [intersect], page 442, [complement], page 442, [unique], page 441.

`intersect` (*a*, *b*) [Function File]
`[c, ia, ib] = intersect` (*a*, *b*) [Function File]

Return the elements in both *a* and *b*, sorted in ascending order. If *a* and *b* are both column vectors return a column vector, otherwise return a row vector.

Return index vectors *ia* and *ib* such that `a(ia)==c` and `b(ib)==c`.

See also: [unique], page 441, [union], page 442, [setxor], page 443, [setdiff], page 442, [ismember], page 441.

`complement` (*x*, *y*) [Function File]

Return the elements of set *y* that are not in set *x*. For example,

```
complement ([ 1, 2, 3 ], [ 2, 3, 5 ])
     ⇒ 5
```

See also: [union], page 442, [intersect], page 442, [unique], page 441.

`setdiff` (*a*, *b*) [Function File]
`setdiff` (*a*, *b*, "*rows*") [Function File]
`[c, i] = setdiff` (*a*, *b*) [Function File]

Return the elements in *a* that are not in *b*, sorted in ascending order. If *a* and *b* are both column vectors return a column vector, otherwise return a row vector.

Given the optional third argument '"rows"', return the rows in *a* that are not in *b*, sorted in ascending order by rows.

If requested, return *i* such that `c = a(i)`.

See also: [unique], page 441, [union], page 442, [intersect], page 442, [setxor], page 443, [ismember], page 441.

`setxor` (*a*, *b*) [Function File]
`setxor` (*a*, *b*, *'rows'*) [Function File]

Return the elements exclusive to *a* or *b*, sorted in ascending order. If *a* and *b* are both column vectors return a column vector, otherwise return a row vector.

`[`*c*`, `*ia*`, `*ib*`] = setxor` (*a*, *b*) [Function File]

Return index vectors *ia* and *ib* such that `a == c(ia)` and `b == c(ib)`.

See also: [unique], page 441, [union], page 442, [intersect], page 442, [setdiff], page 442, [ismember], page 441.

27 Polynomial Manipulations

In Octave, a polynomial is represented by its coefficients (arranged in descending order). For example, a vector c of length $N+1$ corresponds to the following polynomial of order N

$$p(x) = c_1 x^N + \ldots + c_N x + c_{N+1}.$$

27.1 Evaluating Polynomials

The value of a polynomial represented by the vector c can be evaluated at the point x very easily, as the following example shows:

```
N = length(c)-1;
val = dot( x.^(N:-1:0), c );
```

While the above example shows how easy it is to compute the value of a polynomial, it isn't the most stable algorithm. With larger polynomials you should use more elegant algorithms, such as Horner's Method, which is exactly what the Octave function `polyval` does.

In the case where x is a square matrix, the polynomial given by c is still well-defined. As when x is a scalar the obvious implementation is easily expressed in Octave, but also in this case more elegant algorithms perform better. The `polyval` function provides such an algorithm.

y = `polyval` (*p*, *x*) [Function File]
y = `polyval` (*p*, *x*, [], *mu*) [Function File]
Evaluate the polynomial at of the specified values for *x*. When *mu* is
present evaluate the polynomial for (*x*-*mu*(1))/*mu*(2). If *x* is a vector or matrix, the polynomial is evaluated for each of the elements of *x*. [*y*, *dy*] = `polyval` (*p*, *x*, *s*)

[*y*, *dy*] = `polyval` (*p*, *x*, *s*, *mu*) [Function File]

In addition to evaluating the polynomial, the second output represents the prediction interval, *y* +/- *dy*, which contains at least 50% of the future predictions. To calculate the prediction interval, the structured variable *s*, originating form 'polyfit', must be present.

See also: [polyfit], page 450, [polyvalm], page 445, [poly], page 452, [roots], page 446, [conv], page 447, [deconv], page 447, [residue], page 448, [filter], page 478, [polyderiv], page 449, [polyinteg], page 450.

`polyvalm` (*c*, *x*) [Function File]

Evaluate a polynomial in the matrix sense.

`polyvalm (c, x)` will evaluate the polynomial in the matrix sense, i.e., matrix multiplication is used instead of element by element multiplication as is used in polyval.

The argument *x* must be a square matrix.

See also: [polyval], page 445, [poly], page 452, [roots], page 446, [conv], page 447, [deconv], page 447, [residue], page 448, [filter], page 478, [polyderiv], page 449, [polyinteg], page 450.

27.2 Finding Roots

Octave can find the roots of a given polynomial. This is done by computing the companion matrix of the polynomial (see the `compan` function for a definition), and then finding its eigenvalues.

`roots (v)` [Function File]

For a vector *v* with *N* components, return the roots of the polynomial

$$v_1 z^{N-1} + \cdots + v_{N-1} z + v_N.$$

As an example, the following code finds the roots of the quadratic polynomial

$$p(x) = x^2 - 5.$$

```
c = [1, 0, -5];
roots(c)
⇒  2.2361
⇒ -2.2361
```

Note that the true result is $\pm\sqrt{5}$ which is roughly ± 2.2361.

See also: [compan], page 446.

`compan (c)` [Function File]

Compute the companion matrix corresponding to polynomial coefficient vector *c*.

The companion matrix is

$$A = \begin{bmatrix} -c_2/c_1 & -c_3/c_1 & \cdots & -c_N/c_1 & -c_{N+1}/c_1 \\ 1 & 0 & \cdots & 0 & 0 \\ 0 & 1 & \cdots & 0 & 0 \\ \vdots & \vdots & \ddots & \vdots & \vdots \\ 0 & 0 & \cdots & 1 & 0 \end{bmatrix}.$$

The eigenvalues of the companion matrix are equal to the roots of the polynomial.

See also: [poly], page 452, [roots], page 446, [residue], page 448, [conv], page 447, [deconv], page 447, [polyval], page 445, [polyderiv], page 449, [polyinteg], page 450.

`[multp, indx] = mpoles (p)` [Function File]
`[multp, indx] = mpoles (p, tol)` [Function File]
`[multp, indx] = mpoles (p, tol, reorder)` [Function File]

Identify unique poles in *p* and associates their multiplicity, ordering them from largest to smallest.

If the relative difference of the poles is less than *tol*, then they are considered to be multiples. The default value for *tol* is 0.001.

If the optional parameter *reorder* is zero, poles are not sorted.

The value *multp* is a vector specifying the multiplicity of the poles. *multp*(:) refers to multiplicity of *p*(*indx*(:)).

For example,

```
p = [2 3 1 1 2];
[m, n] = mpoles(p);
  ⇒ m = [1; 1; 2; 1; 2]
  ⇒ n = [2; 5; 1; 4; 3]
  ⇒ p(n) = [3, 2, 2, 1, 1]
```

See also: [poly], page 452, [roots], page 446, [conv], page 447, [deconv], page 447, [polyval], page 445, [polyderiv], page 449, [polyinteg], page 450, [residue], page 448.

27.3 Products of Polynomials

`conv (a, b)` [Function File]

Convolve two vectors.

`y = conv (a, b)` returns a vector of length equal to `length (a) + length (b) - 1`. If *a* and *b* are polynomial coefficient vectors, `conv` returns the coefficients of the product polynomial.

See also: [deconv], page 447, [poly], page 452, [roots], page 446, [residue], page 448, [polyval], page 445, [polyderiv], page 449, [polyinteg], page 450.

`c = convn (a, b, shape)` [Function File]

N-dimensional convolution of matrices *a* and *b*.

The size of the output is determined by the *shape* argument. This can be any of the following character strings:

"full" The full convolution result is returned. The size out of the output is `size (a) + size (b)-1`. This is the default behavior.

"same" The central part of the convolution result is returned. The size out of the output is the same as *a*.

"valid" The valid part of the convolution is returned. The size of the result is `max (size (a) - size (b)+1, 0)`.

See also: [conv], page 447, [conv2], page 447.

`deconv (y, a)` [Function File]

Deconvolve two vectors.

`[b, r] = deconv (y, a)` solves for *b* and *r* such that `y = conv (a, b) + r`.

If *y* and *a* are polynomial coefficient vectors, *b* will contain the coefficients of the polynomial quotient and *r* will be a remainder polynomial of lowest order.

See also: [conv], page 447, [poly], page 452, [roots], page 446, [residue], page 448, [polyval], page 445, [polyderiv], page 449, [polyinteg], page 450.

`y = conv2 (a, b, shape)` [Loadable Function]
`y = conv2 (v1, v2, M, shape)` [Loadable Function]

Returns 2D convolution of *a* and *b* where the size of *c* is given by

shape= 'full'
returns full 2-D convolution

shape= 'same'
: same size as a. 'central' part of convolution

shape= 'valid'
: only parts which do not include zero-padded edges

By default *shape* is 'full'. When the third argument is a matrix returns the convolution of the matrix *M* by the vector *v1* in the column direction and by vector *v2* in the row direction

`q = polygcd (b, a, tol)` [Function File]

Find greatest common divisor of two polynomials. This is equivalent to the polynomial found by multiplying together all the common roots. Together with deconv, you can reduce a ratio of two polynomials. Tolerance defaults to

```
sqrt(eps).
```

Note that this is an unstable algorithm, so don't try it on large polynomials.

Example

```
polygcd (poly(1:8), poly(3:12)) - poly(3:8)
⇒ [ 0, 0, 0, 0, 0, 0, 0 ]
deconv (poly(1:8), polygcd (poly(1:8), poly(3:12))) ...
  - poly(1:2)
⇒ [ 0, 0, 0 ]
```

See also: [poly], page 452, [polyinteg], page 450, [polyderiv], page 449, [polyreduce], page 452, [roots], page 446, [conv], page 447, [deconv], page 447, [residue], page 448, [filter], page 478, [polyval], page 445, [polyvalm], page 445.

`[r, p, k, e] = residue (b, a)` [Function File]

Compute the partial fraction expansion for the quotient of the polynomials, *b* and *a*.

$$\frac{B(s)}{A(s)} = \sum_{m=1}^{M} \frac{r_m}{(s-p_m)_m^e} + \sum_{i=1}^{N} k_i s^{N-i}.$$

where *M* is the number of poles (the length of the *r*, *p*, and *e*), the *k* vector is a polynomial of order $N-1$ representing the direct contribution, and the *e* vector specifies the multiplicity of the m-th residue's pole.

For example,

```
b = [1, 1, 1];
a = [1, -5, 8, -4];
[r, p, k, e] = residue (b, a);
     ⇒ r = [-2; 7; 3]
     ⇒ p = [2; 2; 1]
     ⇒ k = [](0x0)
     ⇒ e = [1; 2; 1]
```

which represents the following partial fraction expansion

$$\frac{s^2+s+1}{s^3-5s^2+8s-4} = \frac{-2}{s-2} + \frac{7}{(s-2)^2} + \frac{3}{s-1}$$

`[b, a] = residue (r, p, k)` [Function File]
`[b, a] = residue (r, p, k, e)` [Function File]

Compute the reconstituted quotient of polynomials, $b(s)/a(s)$, from the partial fraction expansion; represented by the residues, poles, and a direct polynomial specified by r, p and k, and the pole multiplicity e.

If the multiplicity, e, is not explicitly specified the multiplicity is determined by the script mpoles.m.

For example,

```
r = [-2; 7; 3];
p = [2; 2; 1];
k = [1, 0];
[b, a] = residue (r, p, k);
     ⇒ b = [1, -5, 9, -3, 1]
     ⇒ a = [1, -5, 8, -4]

where mpoles.m is used to determine e = [1; 2; 1]
```

Alternatively the multiplicity may be defined explicitly, for example,

```
r = [7; 3; -2];
p = [2; 1; 2];
k = [1, 0];
e = [2; 1; 1];
[b, a] = residue (r, p, k, e);
     ⇒ b = [1, -5, 9, -3, 1]
     ⇒ a = [1, -5, 8, -4]
```

which represents the following partial fraction expansion

$$\frac{-2}{s-2} + \frac{7}{(s-2)^2} + \frac{3}{s-1} + s = \frac{s^4 - 5s^3 + 9s^2 - 3s + 1}{s^3 - 5s^2 + 8s - 4}$$

See also: [poly], page 452, [roots], page 446, [conv], page 447, [deconv], page 447, [mpoles], page 446, [polyval], page 445, [polyderiv], page 449, [polyinteg], page 450.

27.4 Derivatives and Integrals

Octave comes with functions for computing the derivative and the integral of a polynomial. The functions `polyderiv` and `polyint` both return new polynomials describing the result. As an example we'll compute the definite integral of $p(x) = x^2 + 1$ from 0 to 3.

```
c = [1, 0, 1];
integral = polyint(c);
area = polyval(integral, 3) - polyval(integral, 0)
⇒ 12
```

`polyderiv (c)` [Function File]
`[q] = polyderiv (b, a)` [Function File]

`[q, r] = polyderiv (b, a)` [Function File]

Return the coefficients of the derivative of the polynomial whose coefficients are given by vector *c*. If a pair of polynomials is given *b* and *a*, the derivative of the product is returned in *q*, or the quotient numerator in *q* and the quotient denominator in *r*.

See also: [poly], page 452, [polyinteg], page 450, [polyreduce], page 452, [roots], page 446, [conv], page 447, [deconv], page 447, [residue], page 448, [filter], page 478, [polygcd], page 448, [polyval], page 445, [polyvalm], page 445.

`polyder (c)` [Function File]
`[q] = polyder (b, a)` [Function File]
`[q, r] = polyder (b, a)` [Function File]

See polyderiv.

`polyinteg (c)` [Function File]

Return the coefficients of the integral of the polynomial whose coefficients are represented by the vector *c*.

The constant of integration is set to zero.

See also: [polyint], page 450, [poly], page 452, [polyderiv], page 449, [polyreduce], page 452, [roots], page 446, [conv], page 447, [deconv], page 447, [residue], page 448, [filter], page 478, [polyval], page 445, [polyvalm], page 445.

`polyint (c, k)` [Function File]

Return the coefficients of the integral of the polynomial whose coefficients are represented by the vector *c*. The variable *k* is the constant of integration, which by default is set to zero.

See also: [poly], page 452, [polyderiv], page 449, [polyreduce], page 452, [roots], page 446, [conv], page 447, [deconv], page 447, [residue], page 448, [filter], page 478, [polyval], page 445, [polyvalm], page 445.

27.5 Polynomial Interpolation

Octave comes with good support for various kinds of interpolation, most of which are described in Chapter 28 [Interpolation], page 455. One simple alternative to the functions described in the aforementioned chapter, is to fit a single polynomial to some given data points. To avoid a highly fluctuating polynomial, one most often wants to fit a low-order polynomial to data. This usually means that it is necessary to fit the polynomial in a least-squares sense, which is what the `polyfit` function does.

`[p, s, mu] = polyfit (x, y, n)` [Function File]

Return the coefficients of a polynomial $p(x)$ of degree *n* that minimizes the least-squares-error of the fit.

The polynomial coefficients are returned in a row vector.

The second output is a structure containing the following fields:

‘`R`’ Triangular factor R from the QR decomposition.

‘`X`’ The Vandermonde matrix used to compute the polynomial coefficients.

‘`df`’ The degrees of freedom.

'normr' The norm of the residuals.

'yf' The values of the polynomial for each value of *x*.

The second output may be used by `polyval` to calculate the statistical error limits of the predicted values.

When the third output, *mu*, is present the coefficients, *p*, are associated with a polynomial in *xhat* = (*x*-*mu*(1))/*mu*(2). Where *mu*(1) = mean (*x*), and *mu*(2) = std (*x*). This linear transformation of *x* improves the numerical stability of the fit.

See also: [polyval], page 445, [residue], page 448.

In situations where a single polynomial isn't good enough, a solution is to use several polynomials pieced together. The function `mkpp` creates a piece-wise polynomial, `ppval` evaluates the function created by `mkpp`, and `unmkpp` returns detailed information about the function.

The following example shows how to combine two linear functions and a quadratic into one function. Each of these functions is expressed on adjoined intervals.

```
x = [-2, -1, 1, 2];
p = [ 0,  1, 0;
      1, -2, 1;
      0, -1, 1 ];
pp = mkpp(x, p);
xi = linspace(-2, 2, 50);
yi = ppval(pp, xi);
plot(xi, yi);
```

yi = `ppval` (*pp*, *xi*) [Function File]

Evaluate piece-wise polynomial *pp* at the points *xi*. If `pp.d` is a scalar greater than 1, or an array, then the returned value *yi* will be an array that is `d1, d1, ..., dk, length (xi)]`.

See also: [mkpp], page 451, [unmkpp], page 451, [spline], page 458.

pp = `mkpp` (*x*, *p*) [Function File]
pp = `mkpp` (*x*, *p*, *d*) [Function File]

Construct a piece-wise polynomial structure from sample points *x* and coefficients *p*. The i-th row of *p*, `p (i,:)`, contains the coefficients for the polynomial over the *i*-th interval, ordered from highest to lowest. There must be one row for each interval in *x*, so `rows (p) == length (x) - 1`.

You can concatenate multiple polynomials of the same order over the same set of intervals using `p = [ p1; p2; ...; pd ]`. In this case, `rows (p) == d * (length (x) - 1)`.

d specifies the shape of the matrix *p* for all except the last dimension. If *d* is not specified it will be computed as `round (rows (p) / (length (x) - 1))` instead.

See also: [unmkpp], page 451, [ppval], page 451, [spline], page 458.

[*x*, *p*, *n*, *k*, *d*] = `unmkpp` (*pp*) [Function File]

Extract the components of a piece-wise polynomial structure *pp*. These are as follows:

x Sample points.

p Polynomial coefficients for points in sample interval. `p (i, :)` contains the coefficients for the polynomial over interval *i* ordered from highest to lowest. If `d > 1`, `p (r, i, :)` contains the coefficients for the r-th polynomial defined on interval *i*. However, this is stored as a 2-D array such that `c = reshape (p (:, j), n, d)` gives `c (i, r)` is the j-th coefficient of the r-th polynomial over the i-th interval.

n Number of polynomial pieces.

k Order of the polynomial plus 1.

d Number of polynomials defined for each interval.

See also: [mkpp], page 451, [ppval], page 451, [spline], page 458.

27.6 Miscellaneous Functions

`poly (a)` [Function File]

If *a* is a square *N*-by-*N* matrix, `poly (a)` is the row vector of the coefficients of `det (z * eye (N) - a)`, the characteristic polynomial of *a*. As an example we can use this to find the eigenvalues of *a* as the roots of `poly (a)`.

```
roots(poly(eye(3)))
⇒ 1.00000 + 0.00000i
⇒ 1.00000 - 0.00000i
⇒ 1.00000 + 0.00000i
```

In real-life examples you should, however, use the `eig` function for computing eigenvalues.

If *x* is a vector, `poly (x)` is a vector of coefficients of the polynomial whose roots are the elements of *x*. That is, of *c* is a polynomial, then the elements of `d = roots (poly (c))` are contained in *c*. The vectors *c* and *d* are, however, not equal due to sorting and numerical errors.

See also: [eig], page 316, [roots], page 446.

`polyout (c, x)` [Function File]

Write formatted polynomial

$$c(x) = c_1 x^n + \ldots + c_n x + c_{n+1}$$

and return it as a string or write it to the screen (if *nargout* is zero). *x* defaults to the string `"s"`.

See also: [polyval], page 445, [polyvalm], page 445, [poly], page 452, [roots], page 446, [conv], page 447, [deconv], page 447, [residue], page 448, [filter], page 478, [polyderiv], page 449, [polyinteg], page 450.

`polyreduce (c)` [Function File]

Reduces a polynomial coefficient vector to a minimum number of terms by stripping off any leading zeros.

See also: [poly], page 452, [roots], page 446, [conv], page 447, [deconv], page 447, [residue], page 448, [filter], page 478, [polyval], page 445, [polyvalm], page 445, [polyderiv], page 449, [polyinteg], page 450.

28 Interpolation

28.1 One-dimensional Interpolation

Octave supports several methods for one-dimensional interpolation, most of which are described in this section. Section 27.5 [Polynomial Interpolation], page 450 and Section 29.4 [Interpolation on Scattered Data], page 472 describe further methods.

yi = `interp1` (*x*, *y*, *xi*) [Function File]
yi = `interp1` (. . ., *method*) [Function File]
yi = `interp1` (. . ., *extrap*) [Function File]
pp = `interp1` (. . ., *'pp'*) [Function File]

One-dimensional interpolation. Interpolate *y*, defined at the points *x*, at the points *xi*. The sample points *x* must be strictly monotonic. If *y* is an array, treat the columns of *y* separately.

Method is one of:

'nearest' Return the nearest neighbor.

'linear' Linear interpolation from nearest neighbors

'pchip' Piece-wise cubic hermite interpolating polynomial

'cubic' Cubic interpolation from four nearest neighbors

'spline' Cubic spline interpolation–smooth first and second derivatives throughout the curve

Appending '*' to the start of the above method forces `interp1` to assume that *x* is uniformly spaced, and only `x (1)` and `x (2)` are referenced. This is usually faster, and is never slower. The default method is 'linear'.

If *extrap* is the string 'extrap', then extrapolate values beyond the endpoints. If *extrap* is a number, replace values beyond the endpoints with that number. If *extrap* is missing, assume NA.

If the string argument 'pp' is specified, then *xi* should not be supplied and `interp1` returns the piece-wise polynomial that can later be used with `ppval` to evaluate the interpolation. There is an equivalence, such that `ppval (interp1 (x, y, method, 'pp'), xi) == interp1 (x, y, xi, method, 'extrap')`.

An example of the use of `interp1` is

```
xf = [0:0.05:10];
yf = sin (2*pi*xf/5);
xp = [0:10];
yp = sin (2*pi*xp/5);
lin = interp1 (xp, yp, xf);
spl = interp1 (xp, yp, xf, "spline");
cub = interp1 (xp, yp, xf, "cubic");
near = interp1 (xp, yp, xf, "nearest");
plot (xf, yf, "r", xf, lin, "g", xf, spl, "b",
      xf, cub, "c", xf, near, "m", xp, yp, "r*");
legend ("original", "linear", "spline", "cubic", "nearest")
```

See also: [interpft], page 457.

There are some important differences between the various interpolation methods. The 'spline' method enforces that both the first and second derivatives of the interpolated values have a continuous derivative, whereas the other methods do not. This means that the results of the 'spline' method are generally smoother. If the function to be interpolated is in fact smooth, then 'spline' will give excellent results. However, if the function to be evaluated is in some manner discontinuous, then 'pchip' interpolation might give better results.

This can be demonstrated by the code

```
t = -2:2;
dt = 1;
ti =-2:0.025:2;
dti = 0.025;
y = sign(t);
ys = interp1(t,y,ti,'spline');
yp = interp1(t,y,ti,'pchip');
ddys = diff(diff(ys)./dti)./dti;
ddyp = diff(diff(yp)./dti)./dti;
figure(1);
plot (ti, ys,'r-', ti, yp,'g-');
legend('spline','pchip',4);
figure(2);
plot (ti, ddys,'r+', ti, ddyp,'g*');
legend('spline','pchip');
```

The result of which can be seen in Figure 28.1 and Figure 28.2.

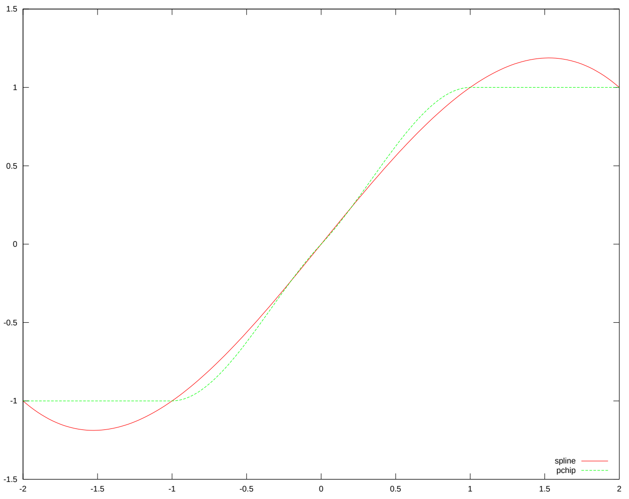

Figure 28.1: Comparison of 'pchip' and 'spline' interpolation methods for a step function

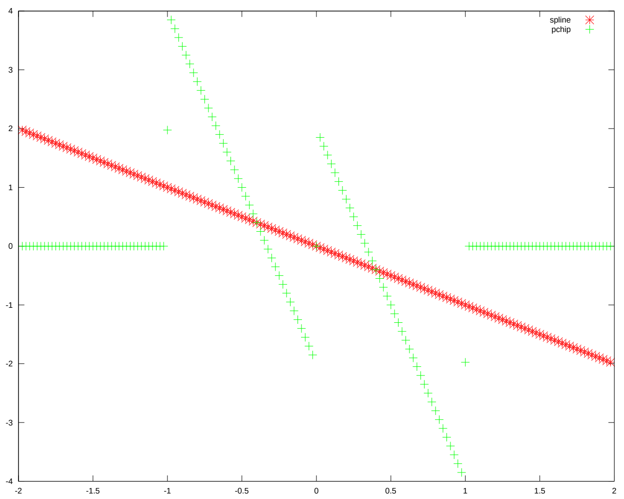

Figure 28.2: Comparison of the second derivative of the 'pchip' and 'spline' interpolation methods for a step function

A simplified version of `interp1` that performs only linear interpolation is available in `interp1q`. This argument is slightly faster than `interp1` as to performs little error checking.

yi = **interp1q** (*x*, *y*, *xi*) [Function File]

One-dimensional linear interpolation without error checking. Interpolates *y*, defined at the points *x*, at the points *xi*. The sample points *x* must be a strictly monotonically increasing column vector. If *y* is an array, treat the columns of *y* separately. If *y* is a vector, it must be a column vector of the same length as *x*.

Values of *xi* beyond the endpoints of the interpolation result in NA being returned.

Note that the error checking is only a significant portion of the execution time of this `interp1` if the size of the input arguments is relatively small. Therefore, the benefit of using `interp1q` is relatively small.

See also: [interp1], page 455.

Fourier interpolation, is a resampling technique where a signal is converted to the frequency domain, padded with zeros and then reconverted to the time domain.

interpft (*x*, *n*) [Function File]
interpft (*x*, *n*, *dim*) [Function File]

Fourier interpolation. If *x* is a vector, then *x* is resampled with *n* points. The data in *x* is assumed to be equispaced. If *x* is an array, then operate along each column of the array separately. If *dim* is specified, then interpolate along the dimension *dim*.

`interpft` assumes that the interpolated function is periodic, and so assumptions are made about the end points of the interpolation.

See also: [interp1], page 455.

There are two significant limitations on Fourier interpolation. Firstly, the function signal is assumed to be periodic, and so non-periodic signals will be poorly represented at the edges. Secondly, both the signal and its interpolation are required to be sampled at equispaced points. An example of the use of `interpft` is

```
t = 0 : 0.3 : pi; dt = t(2)-t(1);
n = length (t); k = 100;
ti = t(1) + [0 : k-1]*dt*n/k;
y = sin (4*t + 0.3) .* cos (3*t - 0.1);
yp = sin (4*ti + 0.3) .* cos (3*ti - 0.1);
plot (ti, yp, 'g', ti, interp1(t, y, ti, 'spline'), 'b', ...
      ti, interpft (y, k), 'c', t, y, 'r+');
legend ('sin(4t+0.3)cos(3t-0.1','spline','interpft','data');
```

which demonstrates the poor behavior of Fourier interpolation for non-periodic functions, as can be seen in Figure 28.3.

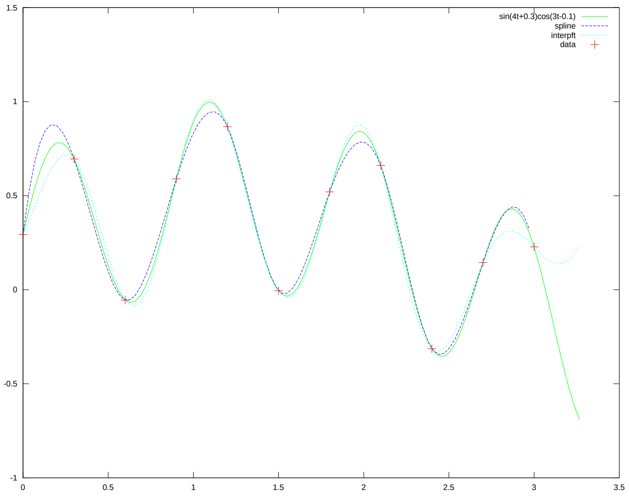

Figure 28.3: Comparison of `interp1` and `interpft` for non-periodic data

In additional the support function `spline` and `lookup` that underlie the `interp1` function can be called directly.

pp = `spline` (*x*, *y*) [Function File]
yi = `spline` (*x*, *y*, *xi*) [Function File]

Return the cubic spline interpolant of *y* at points *x*. If called with two arguments, `spline` returns the piece-wise polynomial *pp* that may later be used with `ppval` to evaluate the polynomial at specific points. If called with a third input argument, `spline` evaluates the spline at the points *xi*. There is an equivalence between `ppval (spline (x, y), xi)` and `spline (x, y, xi)`.

The variable *x* must be a vector of length *n*, and *y* can be either a vector or array. In the case where *y* is a vector, it can have a length of either *n* or `n + 2`. If the length of *y* is *n*, then the 'not-a-knot' end condition is used. If the length of *y* is `n + 2`, then

the first and last values of the vector *y* are the values of the first derivative of the cubic spline at the end-points.

If *y* is an array, then the size of *y* must have the form

$$[s_1, s_2, \cdots, s_k, n]$$

or

$$[s_1, s_2, \cdots, s_k, n+2].$$

The array is then reshaped internally to a matrix where the leading dimension is given by

$$s_1 s_2 \cdots s_k$$

and each row of this matrix is then treated separately. Note that this is exactly the opposite treatment than `interp1` and is done for compatibility.

See also: [ppval], page 451, [mkpp], page 451, [unmkpp], page 451.

The `lookup` function is used by other interpolation functions to identify the points of the original data that are closest to the current point of interest.

idx = `lookup` (*table*, *y*, *opt*) [Loadable Function]

Lookup values in a sorted table. Usually used as a prelude to interpolation.

If table is strictly increasing and `idx = lookup (table, y)`, then `table(idx(i)) <= y(i) < table(idx(i+1))` for all `y(i)` within the table. If `y(i) < table (1)` then `idx(i)` is 0. If `y(i) >= table(end)` then `idx(i)` is `table(n)`.

If the table is strictly decreasing, then the tests are reversed. There are no guarantees for tables which are non-monotonic or are not strictly monotonic.

The algorithm used by lookup is standard binary search, with optimizations to speed up the case of partially ordered arrays (dense downsampling). In particular, looking up a single entry is of logarithmic complexity (unless a conversion occurs due to non-numeric or unequal types).

table and *y* can also be cell arrays of strings (or *y* can be a single string). In this case, string lookup is performed using lexicographical comparison.

If *opts* is specified, it shall be a string with letters indicating additional options. For numeric lookup, 'l' in *opts* indicates that the leftmost subinterval shall be extended to infinity (i.e., all indices at least 1), and 'r' indicates that the rightmost subinterval shall be extended to infinity (i.e., all indices at most n-1).

For string lookup, 'i' indicates case-insensitive comparison.

28.2 Multi-dimensional Interpolation

There are three multi-dimensional interpolation functions in Octave, with similar capabilities. Methods using Delaunay tessellation are described in Section 29.4 [Interpolation on Scattered Data], page 472.

zi = interp2 (*x*, *y*, *z*, *xi*, *yi*) [Function File]
zi = interp2 (*Z*, *xi*, *yi*) [Function File]
zi = interp2 (*Z*, *n*) [Function File]
zi = interp2 (. . ., *method*) [Function File]
zi = interp2 (. . ., *method*, *extrapval*) [Function File]

Two-dimensional interpolation. *x*, *y* and *z* describe a surface function. If *x* and *y* are vectors their length must correspondent to the size of *z*. *x* and *y* must be monotonic. If they are matrices they must have the `meshgrid` format.

`interp2 (x, y, Z, xi, yi, ...)`
: Returns a matrix corresponding to the points described by the matrices *xi*, *yi*.

  If the last argument is a string, the interpolation method can be specified. The method can be 'linear', 'nearest' or 'cubic'. If it is omitted 'linear' interpolation is assumed.

`interp2 (z, xi, yi)`
: Assumes `x = 1:rows (z)` and `y = 1:columns (z)`

`interp2 (z, n)`
: Interleaves the matrix *z* n-times. If *n* is omitted a value of `n = 1` is assumed.

The variable *method* defines the method to use for the interpolation. It can take one of the following values

'nearest' Return the nearest neighbor.

'linear' Linear interpolation from nearest neighbors.

'pchip' Piece-wise cubic hermite interpolating polynomial (not implemented yet).

'cubic' Cubic interpolation from four nearest neighbors.

'spline' Cubic spline interpolation–smooth first and second derivatives throughout the curve.

If a scalar value *extrapval* is defined as the final value, then values outside the mesh as set to this value. Note that in this case *method* must be defined as well. If *extrapval* is not defined then NA is assumed.

See also: [interp1], page 455.

vi = interp3 (*x*, *y*,*z*, *v*, *xi*, *yi*, *zi*) [Function File]
vi = interp3 (*v*, *xi*, *yi*, *zi*) [Function File]
vi = interp3 (*v*, *m*) [Function File]
vi = interp3 (*v*) [Function File]
vi = interp3 (. . ., *method*) [Function File]
vi = interp3 (. . ., *method*, *extrapval*) [Function File]

Perform 3-dimensional interpolation. Each element of the 3-dimensional array *v* represents a value at a location given by the parameters *x*, *y*, and *z*. The parameters *x*, *x*, and *z* are either 3-dimensional arrays of the same size as the array *v* in the 'meshgrid' format or vectors. The parameters *xi*, etc. respect a similar format to *x*, etc., and they represent the points at which the array *vi* is interpolated.

If *x*, *y*, *z* are omitted, they are assumed to be `x = 1 : size (v, 2)`, `y = 1 : size (v, 1)` and `z = 1 : size (v, 3)`. If *m* is specified, then the interpolation adds a point half way between each of the interpolation points. This process is performed *m* times. If only *v* is specified, then *m* is assumed to be `1`.

Method is one of:

'nearest' Return the nearest neighbor.

'linear' Linear interpolation from nearest neighbors.

'cubic' Cubic interpolation from four nearest neighbors (not implemented yet).

'spline' Cubic spline interpolation–smooth first and second derivatives throughout the curve.

The default method is 'linear'.

If *extrap* is the string 'extrap', then extrapolate values beyond the endpoints. If *extrap* is a number, replace values beyond the endpoints with that number. If *extrap* is missing, assume NA.

See also: [interp1], page 455, [interp2], page 459, [spline], page 458, [meshgrid], page 229.

`vi = interpn (x1, x2, ..., v, y1, y2, ...)` [Function File]
`vi = interpn (v, y1, y2, ...)` [Function File]
`vi = interpn (v, m)` [Function File]
`vi = interpn (v)` [Function File]
`vi = interpn (..., method)` [Function File]
`vi = interpn (..., method, extrapval)` [Function File]

Perform *n*-dimensional interpolation, where *n* is at least two. Each element of the *n*-dimensional array *v* represents a value at a location given by the parameters *x1*, *x2*, ..., *xn*. The parameters *x1*, *x2*, ..., *xn* are either *n*-dimensional arrays of the same size as the array *v* in the 'ndgrid' format or vectors. The parameters *y1*, etc. respect a similar format to *x1*, etc., and they represent the points at which the array *vi* is interpolated.

If *x1*, ..., *xn* are omitted, they are assumed to be `x1 = 1 : size (v, 1)`, etc. If *m* is specified, then the interpolation adds a point half way between each of the interpolation points. This process is performed *m* times. If only *v* is specified, then *m* is assumed to be `1`.

Method is one of:

'nearest' Return the nearest neighbor.

'linear' Linear interpolation from nearest neighbors.

'cubic' Cubic interpolation from four nearest neighbors (not implemented yet).

'spline' Cubic spline interpolation–smooth first and second derivatives throughout the curve.

The default method is 'linear'.

If *extrapval* is the scalar value, use it to replace the values beyond the endpoints with that number. If *extrapval* is missing, assume NA.

See also: [interp1], page 455, [interp2], page 459, [spline], page 458, [ndgrid], page 229.

A significant difference between `interpn` and the other two multidimensional interpolation functions is the fashion in which the dimensions are treated. For `interp2` and `interp3`, the 'y' axis is considered to be the columns of the matrix, whereas the 'x' axis corresponds to the rows of the array. As Octave indexes arrays in column major order, the first dimension of any array is the columns, and so `interpn` effectively reverses the 'x' and 'y' dimensions. Consider the example

```
x = y = z = -1:1;
f = @(x,y,z) x.^2 - y - z.^2;
[xx, yy, zz] = meshgrid (x, y, z);
v = f (xx,yy,zz);
xi = yi = zi = -1:0.1:1;
[xxi, yyi, zzi] = meshgrid (xi, yi, zi);
vi = interp3(x, y, z, v, xxi, yyi, zzi, 'spline');
[xxi, yyi, zzi] = ndgrid (xi, yi, zi);
vi2 = interpn(x, y, z, v, xxi, yyi, zzi, 'spline');
mesh (zi, yi, squeeze (vi2(1,:,:)));
```

where `vi` and `vi2` are identical. The reversal of the dimensions is treated in the `meshgrid` and `ndgrid` functions respectively. The result of this code can be seen in Figure 28.4.

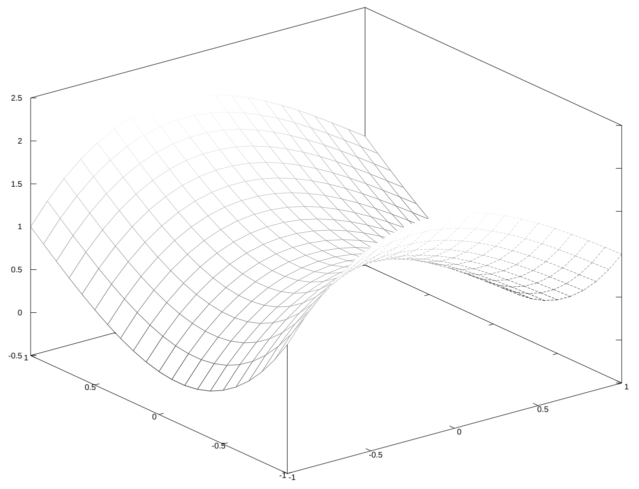

Figure 28.4: Demonstration of the use of `interpn`

In additional the support function `bicubic` that underlies the cubic interpolation of `interp2` function can be called directly.

zi = `bicubic` (*x*, *y*, *z*, *xi*, *yi*, *extrapval*) [Function File]

Return a matrix *zi* corresponding to the bicubic interpolations at *xi* and *yi* of the data supplied as *x*, *y* and *z*. Points outside the grid are set to *extrapval*.

See `http://wiki.woodpecker.org.cn/moin/Octave/Bicubic` for further information.

See also: [interp2], page 459.

29 Geometry

Much of the geometry code in Octave is based on the Qhull library[1]. Some of the documentation for Qhull, particularly for the options that can be passed to `delaunay`, `voronoi` and `convhull`, etc., is relevant to Octave users.

29.1 Delaunay Triangulation

The Delaunay triangulation is constructed from a set of circum-circles. These circum-circles are chosen so that there are at least three of the points in the set to triangulation on the circumference of the circum-circle. None of the points in the set of points falls within any of the circum-circles.

In general there are only three points on the circumference of any circum-circle. However, in some cases, and in particular for the case of a regular grid, 4 or more points can be on a single circum-circle. In this case the Delaunay triangulation is not unique.

tri = delaunay (*x*, *y*) [Function File]
tri = delaunay (*x*, *y*, *opt*) [Function File]

The return matrix of size [n, 3] contains a set triangles which are described by the indices to the data point x and y vector. The triangulation satisfies the Delaunay circum-circle criterion. No other data point is in the circum-circle of the defining triangle.

A third optional argument, which must be a string, contains extra options passed to the underlying qhull command. See the documentation for the Qhull library for details.

```
x = rand (1, 10);
y = rand (size (x));
T = delaunay (x, y);
X = [x(T(:,1)); x(T(:,2)); x(T(:,3)); x(T(:,1))];
Y = [y(T(:,1)); y(T(:,2)); y(T(:,3)); y(T(:,1))];
axis ([0,1,0,1]);
plot (X, Y, "b", x, y, "r*");
```

See also: [voronoi], page 468, [delaunay3], page 463, [delaunayn], page 464.

The 3- and N-dimensional extension of the Delaunay triangulation are given by `delaunay3` and `delaunayn` respectively. `delaunay3` returns a set of tetrahedra that satisfy the Delaunay circum-circle criteria. Similarly, `delaunayn` returns the N-dimensional simplex satisfying the Delaunay circum-circle criteria. The N-dimensional extension of a triangulation is called a tessellation.

T = delaunay3 (*x*, *y*, *z*) [Function File]
T = delaunay3 (*x*, *y*, *z*, *opt*) [Function File]

A matrix of size [n, 4] is returned. Each row contains a set of tetrahedron which are described by the indices to the data point vectors (x,y,z).

---

[1] Barber, C.B., Dobkin, D.P., and Huhdanpaa, H.T., "The Quickhull algorithm for convex hulls," ACM Trans. on Mathematical Software, 22(4):469–483, Dec 1996, `http://www.qhull.org`

A fourth optional argument, which must be a string or cell array of strings, contains extra options passed to the underlying qhull command. See the documentation for the Qhull library for details.

See also: [delaunay], page 463, [delaunayn], page 464.

T = **delaunayn** (*P*) [Function File]
T = **delaunayn** (*P*, *opt*) [Function File]

Form the Delaunay triangulation for a set of points. The Delaunay triangulation is a tessellation of the convex hull of the points such that no n-sphere defined by the n-triangles contains any other points from the set. The input matrix *P* of size `[n, dim]` contains *n* points in a space of dimension dim. The return matrix *T* has the size `[m, dim+1]`. It contains for each row a set of indices to the points, which describes a simplex of dimension dim. For example, a 2d simplex is a triangle and 3d simplex is a tetrahedron.

Extra options for the underlying Qhull command can be specified by the second argument. This argument is a cell array of strings. The default options depend on the dimension of the input:

- 2D and 3D: *opt* = `{"Qt", "Qbb", "Qc"}`
- 4D and higher: *opt* = `{"Qt", "Qbb", "Qc", "Qz"}`

If *opt* is [], then the default arguments are used. If *opt* is `{""}`, then none of the default arguments are used by Qhull. See the Qhull documentation for the available options.

All options can also be specified as single string, for example `"Qt Qbb Qc Qz"`.

An example of a Delaunay triangulation of a set of points is

```
rand ("state", 2);
x = rand (10, 1);
y = rand (10, 1);
T = delaunay (x, y);
X = [ x(T(:,1)); x(T(:,2)); x(T(:,3)); x(T(:,1)) ];
Y = [ y(T(:,1)); y(T(:,2)); y(T(:,3)); y(T(:,1)) ];
axis ([0, 1, 0, 1]);
plot(X, Y, "b", x, y, "r*");
```

The result of which can be seen in Figure 29.1.

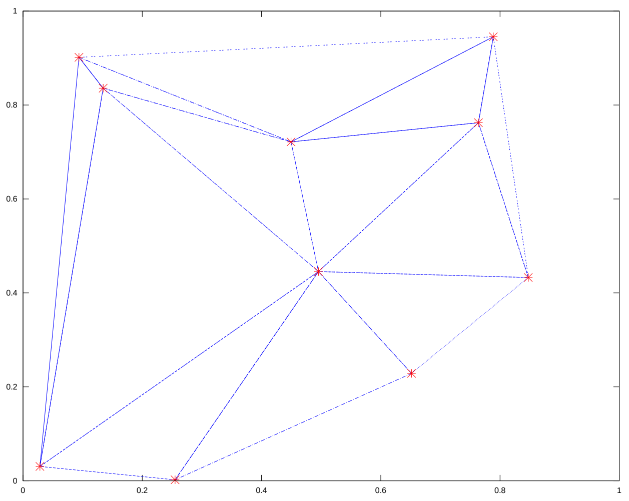

Figure 29.1: Delaunay triangulation of a random set of points

29.1.1 Plotting the Triangulation

Octave has the functions `triplot` and `trimesh` to plot the Delaunay triangulation of a 2-dimensional set of points.

`triplot (`*tri*, *x*, *y*`)` [Function File]
`triplot (`*tri*, *x*, *y*, *linespec*`)` [Function File]
h `= triplot (...)` [Function File]

Plot a triangular mesh in 2D. The variable *tri* is the triangular meshing of the points `(`*x*`, `*y*`)` which is returned from `delaunay`. If given, the *linespec* determines the properties to use for the lines. The output argument *h* is the graphic handle to the plot.

See also: [plot], page 205, [trimesh], page 465, [delaunay], page 463.

`trimesh (`*tri*, *x*, *y*, *z*`)` [Function File]
h `= trimesh (...)` [Function File]

Plot a triangular mesh in 3D. The variable *tri* is the triangular meshing of the points `(`*x*`, `*y*`)` which is returned from `delaunay`. The variable *z* is value at the point `(`*x*`, `*y*`)`. The output argument *h* is the graphic handle to the plot.

See also: [triplot], page 465, [delaunay3], page 463.

The difference between `triplot` and `trimesh` is that the former only plots the 2-dimensional triangulation itself, whereas the second plots the value of some function `f (`*x*`, `*y*`)`. An example of the use of the `triplot` function is

```
rand ("state", 2)
x = rand (20, 1);
y = rand (20, 1);
tri = delaunay (x, y);
triplot (tri, x, y);
```

that plot the Delaunay triangulation of a set of random points in 2-dimensions. The output of the above can be seen in Figure 29.2.

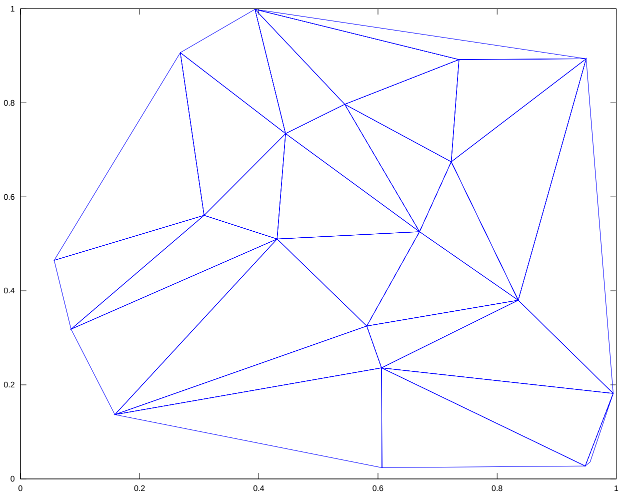

Figure 29.2: Delaunay triangulation of a random set of points

29.1.2 Identifying points in Triangulation

It is often necessary to identify whether a particular point in the N-dimensional space is within the Delaunay tessellation of a set of points in this N-dimensional space, and if so which N-simplex contains the point and which point in the tessellation is closest to the desired point. The functions `tsearch` and `dsearch` perform this function in a triangulation, and `tsearchn` and `dsearchn` in an N-dimensional tessellation.

To identify whether a particular point represented by a vector p falls within one of the simplices of an N-simplex, we can write the Cartesian coordinates of the point in a parametric form with respect to the N-simplex. This parametric form is called the Barycentric Coordinates of the point. If the points defining the N-simplex are given by `N + 1` vectors $t(i,:)$, then the Barycentric coordinates defining the point p are given by

```
p = sum (beta(1:N+1) * t(1:N+1),:)
```

where there are `N + 1` values `beta(i)` that together as a vector represent the Barycentric coordinates of the point p. To ensure a unique solution for the values of `beta(i)` an additional criteria of

```
sum (beta(1:N+1)) == 1
```

is imposed, and we can therefore write the above as

```
p - t(end, :) = beta(1:end-1) * (t(1:end-1, :)
      - ones(N, 1) * t(end, :)
```

Solving for *beta* we can then write

```
beta(1:end-1) = (p - t(end, :)) / (t(1:end-1, :)
      - ones(N, 1) * t(end, :))
beta(end) = sum(beta(1:end-1))
```

which gives the formula for the conversion of the Cartesian coordinates of the point *p* to the Barycentric coordinates *beta*. An important property of the Barycentric coordinates is that for all points in the N-simplex

```
0 <= beta(i) <= 1
```

Therefore, the test in `tsearch` and `tsearchn` essentially only needs to express each point in terms of the Barycentric coordinates of each of the simplices of the N-simplex and test the values of *beta*. This is exactly the implementation used in `tsearchn`. `tsearch` is optimized for 2-dimensions and the Barycentric coordinates are not explicitly formed.

idx = **tsearch** (*x*, *y*, *t*, *xi*, *yi*) [Loadable Function]

Searches for the enclosing Delaunay convex hull. For `t = delaunay (x, y)`, finds the index in *t* containing the points `(xi, yi)`. For points outside the convex hull, *idx* is NaN.

See also: [delaunay], page 463, [delaunayn], page 464.

[*idx*, *p*] = **tsearchn** (*x*, *t*, *xi*) [Function File]

Searches for the enclosing Delaunay convex hull. For `t = delaunayn (x)`, finds the index in *t* containing the points *xi*. For points outside the convex hull, *idx* is NaN. If requested `tsearchn` also returns the Barycentric coordinates *p* of the enclosing triangles.

See also: [delaunay], page 463, [delaunayn], page 464.

An example of the use of `tsearch` can be seen with the simple triangulation

```
x = [-1; -1; 1; 1];
y = [-1; 1; -1; 1];
tri = [1, 2, 3; 2, 3, 1];
```

consisting of two triangles defined by *tri*. We can then identify which triangle a point falls in like

```
tsearch (x, y, tri, -0.5, -0.5)
⇒ 1
tsearch (x, y, tri, 0.5, 0.5)
⇒ 2
```

and we can confirm that a point doesn't lie within one of the triangles like

```
tsearch (x, y, tri, 2, 2)
⇒ NaN
```

The `dsearch` and `dsearchn` find the closest point in a tessellation to the desired point. The desired point does not necessarily have to be in the tessellation, and even if it the returned point of the tessellation does not have to be one of the vertexes of the N-simplex within which the desired point is found.

idx = **dsearch** (*x*, *y*, *tri*, *xi*, *yi*) [Function File]

idx = **dsearch** (*x*, *y*, *tri*, *xi*, *yi*, *s*) [Function File]

Returns the index *idx* or the closest point in `x, y` to the elements `[xi(:), yi(:)]`. The variable *s* is accepted but ignored for compatibility.

See also: [dsearchn], page 468, [tsearch], page 467.

idx = dsearchn (*x*, *tri*, *xi*) [Function File]
idx = dsearchn (*x*, *tri*, *xi*, *outval*) [Function File]
idx = dsearchn (*x*, *xi*) [Function File]
[*idx*, *d*] = dsearchn (...) [Function File]

Returns the index *idx* or the closest point in *x* to the elements *xi*. If *outval* is supplied, then the values of *xi* that are not contained within one of the simplicies *tri* are set to *outval*. Generally, *tri* is returned from `delaunayn (x)`.

See also: [dsearch], page 467, [tsearch], page 467.

An example of the use of `dsearch`, using the above values of *x*, *y* and *tri* is

```
dsearch (x, y, tri, -2, -2)
⇒ 1
```

If you wish the points that are outside the tessellation to be flagged, then `dsearchn` can be used as

```
dsearchn ([x, y], tri, [-2, -2], NaN)
⇒ NaN
dsearchn ([x, y], tri, [-0.5, -0.5], NaN)
⇒ 1
```

where the point outside the tessellation are then flagged with `NaN`.

29.2 Voronoi Diagrams

A Voronoi diagram or Voronoi tessellation of a set of points *s* in an N-dimensional space, is the tessellation of the N-dimensional space such that all points in `v(p)`, a partitions of the tessellation where *p* is a member of *s*, are closer to *p* than any other point in *s*. The Voronoi diagram is related to the Delaunay triangulation of a set of points, in that the vertexes of the Voronoi tessellation are the centers of the circum-circles of the simplicies of the Delaunay tessellation.

voronoi (*x*, *y*) [Function File]
voronoi (*x*, *y*, "*plotstyle*") [Function File]
voronoi (*x*, *y*, "*plotstyle*", *options*) [Function File]
[*vx*, *vy*] = voronoi (...) [Function File]

plots voronoi diagram of points `(x, y)`. The voronoi facets with points at infinity are not drawn. [*vx*, *vy*] = voronoi(...) returns the vertices instead of plotting the diagram. plot (*vx*, *vy*) shows the voronoi diagram.

A fourth optional argument, which must be a string, contains extra options passed to the underlying qhull command. See the documentation for the Qhull library for details.

```
x = rand (10, 1);
y = rand (size (x));
h = convhull (x, y);
[vx, vy] = voronoi (x, y);
plot (vx, vy, "-b", x, y, "o", x(h), y(h), "-g")
legend ("", "points", "hull");
```

See also: [voronoin], page 469, [delaunay], page 463, [convhull], page 471.

`[C, F] = voronoin (pts)` [Function File]
`[C, F] = voronoin (pts, options)` [Function File]

computes n- dimensional voronoi facets. The input matrix *pts* of size [n, dim] contains n points of dimension dim. *C* contains the points of the voronoi facets. The list *F* contains for each facet the indices of the voronoi points.

A second optional argument, which must be a string, contains extra options passed to the underlying qhull command. See the documentation for the Qhull library for details.

See also: [voronoin], page 469, [delaunay], page 463, [convhull], page 471.

An example of the use of `voronoi` is

```
rand("state",9);
x = rand(10,1);
y = rand(10,1);
tri = delaunay (x, y);
[vx, vy] = voronoi (x, y, tri);
triplot (tri, x, y, "b");
hold on;
plot (vx, vy, "r");
```

The result of which can be seen in Figure 29.3. Note that the circum-circle of one of the triangles has been added to this figure, to make the relationship between the Delaunay tessellation and the Voronoi diagram clearer.

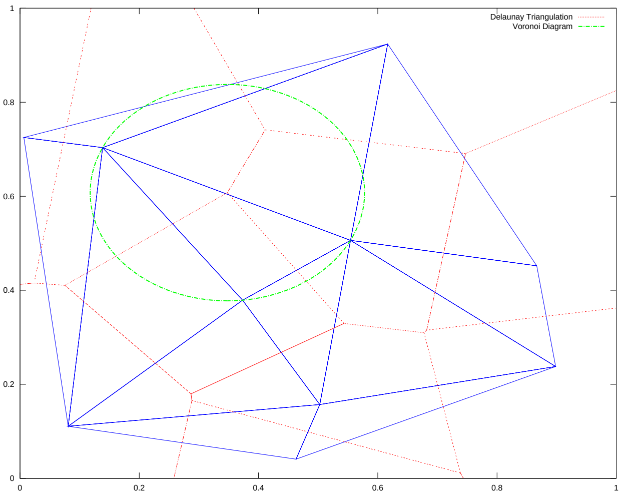

Figure 29.3: Delaunay triangulation and Voronoi diagram of a random set of points

Additional information about the size of the facets of a Voronoi diagram, and which points of a set of points is in a polygon can be had with the `polyarea` and `inpolygon` functions respectively.

`polyarea (x, y)` [Function File]
`polyarea (x, y, dim)` [Function File]

Determines area of a polygon by triangle method. The variables *x* and *y* define the vertex pairs, and must therefore have the same shape. They can be either vectors or arrays. If they are arrays then the columns of *x* and *y* are treated separately and an area returned for each.

If the optional *dim* argument is given, then `polyarea` works along this dimension of the arrays *x* and *y*.

An example of the use of `polyarea` might be

```
rand ("state", 2);
x = rand (10, 1);
y = rand (10, 1);
[c, f] = voronoin ([x, y]);
af = zeros (size(f));
for i = 1 : length (f)
  af(i) = polyarea (c (f {i, :}, 1), c (f {i, :}, 2));
endfor
```

Facets of the Voronoi diagram with a vertex at infinity have infinity area. A simplified version of `polyarea` for rectangles is available with `rectint`

`area = rectint (a, b)` [Function File]

Compute the area of intersection of rectangles in *a* and rectangles in *b*. Rectangles are defined as [x y width height] where x and y are the minimum values of the two orthogonal dimensions.

If *a* or *b* are matrices, then the output, *area*, is a matrix where the i-th row corresponds to the i-th row of a and the j-th column corresponds to the j-th row of b.

See also: [polyarea], page 469.

`[in, on] = inpolygon (x, y, xv, xy)` [Function File]

For a polygon defined by `(xv, yv)` points, determine if the points `(x, y)` are inside or outside the polygon. The variables *x*, *y*, must have the same dimension. The optional output *on* gives the points that are on the polygon.

An example of the use of `inpolygon` might be

```
randn ("state", 2);
x = randn (100, 1);
y = randn (100, 1);
vx = cos (pi * [-1 : 0.1: 1]);
vy = sin (pi * [-1 : 0.1 : 1]);
in = inpolygon (x, y, vx, vy);
plot(vx, vy, x(in), y(in), "r+", x(!in), y(!in), "bo");
axis ([-2, 2, -2, 2]);
```

The result of which can be seen in Figure 29.4.

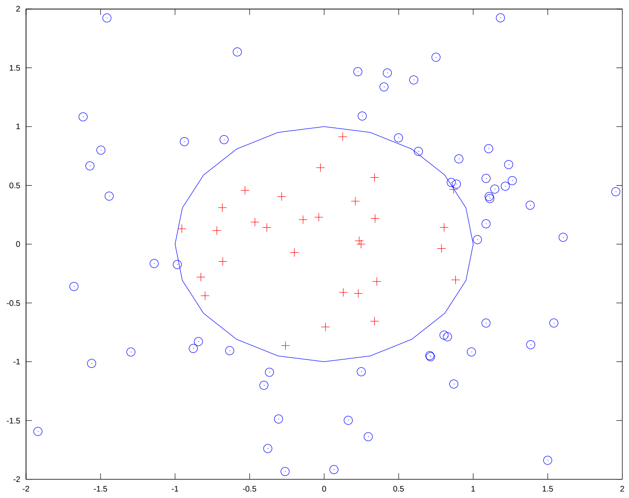

Figure 29.4: Demonstration of the `inpolygon` function to determine the points inside a polygon

29.3 Convex Hull

The convex hull of a set of points is the minimum convex envelope containing all of the points. Octave has the functions `convhull` and `convhulln` to calculate the convex hull of 2-dimensional and N-dimensional sets of points.

H = `convhull` (*x*, *y*) [Function File]
H = `convhull` (*x*, *y*, *opt*) [Function File]

Returns the index vector to the points of the enclosing convex hull. The data points are defined by the x and y vectors.

A third optional argument, which must be a string, contains extra options passed to the underlying qhull command. See the documentation for the Qhull library for details.

See also: [delaunay], page 463, [convhulln], page 471.

h = `convhulln` (*p*) [Loadable Function]
h = `convhulln` (*p*, *opt*) [Loadable Function]
[*h*, *v*] = `convhulln` (. . .) [Loadable Function]

Return an index vector to the points of the enclosing convex hull. The input matrix of size [n, dim] contains n points of dimension dim.

If a second optional argument is given, it must be a string or cell array of strings containing options for the underlying qhull command. (See the Qhull documentation for the available options.) The default options are `"s Qci Tcv"`. If the second output *V* is requested the volume of the convex hull is calculated.

See also: [convhull], page 471, [delaunayn], page 464.

An example of the use of convhull is

```
x = -3:0.05:3;
y = abs (sin (x));
k = convhull (x, y);
plot (x(k), y(k), "r-", x, y, "b+");
axis ([-3.05, 3.05, -0.05, 1.05]);
```

The output of the above can be seen in Figure 29.5.

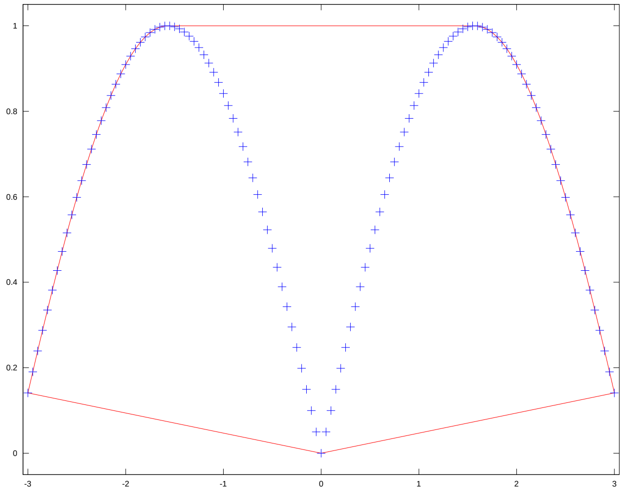

Figure 29.5: The convex hull of a simple set of points

29.4 Interpolation on Scattered Data

An important use of the Delaunay tessellation is that it can be used to interpolate from scattered data to an arbitrary set of points. To do this the N-simplex of the known set of points is calculated with delaunay, delaunay3 or delaunayn. Then the simplicies in to which the desired points are found are identified. Finally the vertices of the simplicies are used to interpolate to the desired points. The functions that perform this interpolation are griddata, griddata3 and griddatan.

zi = griddata (*x*, *y*, *z*, *xi*, *yi*, *method*) [Function File]
[*xi*, *yi*, *zi*] = griddata (*x*, *y*, *z*, *xi*, *yi*, *method*) [Function File]

Generate a regular mesh from irregular data using interpolation. The function is defined by *z* = f (*x*, *y*). The interpolation points are all (*xi*, *yi*). If *xi*, *yi* are vectors then they are made into a 2D mesh.

The interpolation method can be "nearest", "cubic" or "linear". If method is omitted it defaults to "linear".

See also: [delaunay], page 463.

vi = griddata3 (*x*, *y*, *z*, *v* *xi*, *yi*, *zi*, *method*, *options*) [Function File]

Generate a regular mesh from irregular data using interpolation. The function is defined by `y = f (x,y,z)`. The interpolation points are all *xi*.

The interpolation method can be `"nearest"` or `"linear"`. If method is omitted it defaults to `"linear"`.

See also: [griddata], page 472, [delaunayn], page 464.

yi = griddatan (*x*, *y*, *xi*, *method*, *options*) [Function File]

Generate a regular mesh from irregular data using interpolation. The function is defined by `y = f (x)`. The interpolation points are all *xi*.

The interpolation method can be `"nearest"` or `"linear"`. If method is omitted it defaults to `"linear"`.

See also: [griddata], page 472, [delaunayn], page 464.

An example of the use of the `griddata` function is

```
rand("state",1);
x=2*rand(1000,1)-1;
y=2*rand(size(x))-1;
z=sin(2*(x.^2+y.^2));
[xx,yy]=meshgrid(linspace(-1,1,32));
griddata(x,y,z,xx,yy);
```

that interpolates from a random scattering of points, to a uniform grid. The output of the above can be seen in Figure 29.6.

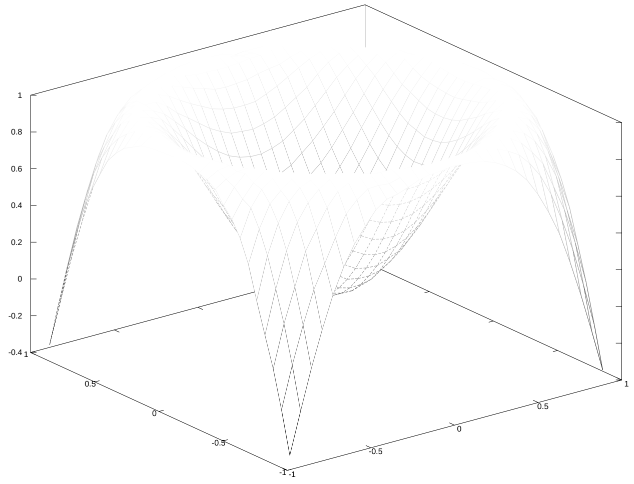

Figure 29.6: Interpolation from a scattered data to a regular grid

30 Signal Processing

This chapter describes the signal processing and fast Fourier transform functions available in Octave. Fast Fourier transforms are computed with the FFTW or FFTPACK libraries depending on how Octave is built.

`detrend (x, p)` [Function File]

If *x* is a vector, `detrend (x, p)` removes the best fit of a polynomial of order *p* from the data *x*.

If *x* is a matrix, `detrend (x, p)` does the same for each column in *x*.

The second argument is optional. If it is not specified, a value of 1 is assumed. This corresponds to removing a linear trend.

`fft (a, n, dim)` [Loadable Function]

Compute the FFT of *a* using subroutines from FFTW. The FFT is calculated along the first non-singleton dimension of the array. Thus if *a* is a matrix, `fft (a)` computes the FFT for each column of *a*.

If called with two arguments, *n* is expected to be an integer specifying the number of elements of *a* to use, or an empty matrix to specify that its value should be ignored. If *n* is larger than the dimension along which the FFT is calculated, then *a* is resized and padded with zeros. Otherwise, if *n* is smaller than the dimension along which the FFT is calculated, then *a* is truncated.

If called with three arguments, *dim* is an integer specifying the dimension of the matrix along which the FFT is performed

See also: [ifft], page 476, [fft2], page 477, [fftn], page 477, [fftw], page 475.

Octave uses the FFTW libraries to perform FFT computations. When Octave starts up and initializes the FFTW libraries, they read a system wide file (on a Unix system, it is typically '`/etc/fftw/wisdom`') that contains information useful to speed up FFT computations. This information is called the *wisdom*. The system-wide file allows wisdom to be shared between all applications using the FFTW libraries.

Use the `fftw` function to generate and save wisdom. Using the utilities provided together with the FFTW libraries (`fftw-wisdom` on Unix systems), you can even add wisdom generated by Octave to the system-wide wisdom file.

`method = fftw ('planner')` [Loadable Function]
`fftw ('planner', method)` [Loadable Function]
`wisdom = fftw ('dwisdom')` [Loadable Function]
`wisdom = fftw ('dwisdom', wisdom)` [Loadable Function]

Manage FFTW wisdom data. Wisdom data can be used to significantly accelerate the calculation of the FFTs but implies an initial cost in its calculation. When the FFTW libraries are initialized, they read a system wide wisdom file (typically in '`/etc/fftw/wisdom`'), allowing wisdom to be shared between applications other than Octave. Alternatively, the `fftw` function can be used to import wisdom. For example

```
wisdom = fftw ('dwisdom')
```

will save the existing wisdom used by Octave to the string *wisdom*. This string can then be saved to a file and restored using the `save` and `load` commands respectively. This existing wisdom can be reimported as follows

```
fftw ('dwisdom', wisdom)
```

If *wisdom* is an empty matrix, then the wisdom used is cleared.

During the calculation of Fourier transforms further wisdom is generated. The fashion in which this wisdom is generated is equally controlled by the `fftw` function. There are five different manners in which the wisdom can be treated, these being

'estimate' This specifies that no run-time measurement of the optimal means of calculating a particular is performed, and a simple heuristic is used to pick a (probably sub-optimal) plan. The advantage of this method is that there is little or no overhead in the generation of the plan, which is appropriate for a Fourier transform that will be calculated once.

'measure' In this case a range of algorithms to perform the transform is considered and the best is selected based on their execution time.

'patient' This is like 'measure', but a wider range of algorithms is considered.

'exhaustive'
This is like 'measure', but all possible algorithms that may be used to treat the transform are considered.

'hybrid' As run-time measurement of the algorithm can be expensive, this is a compromise where 'measure' is used for transforms up to the size of 8192 and beyond that the 'estimate' method is used.

The default method is 'estimate', and the method currently being used can be probed with

```
method = fftw ('planner')
```

and the method used can be set using

```
fftw ('planner', method)
```

Note that calculated wisdom will be lost when restarting Octave. However, the wisdom data can be reloaded if it is saved to a file as described above. Saved wisdom files should not be used on different platforms since they will not be efficient and the point of calculating the wisdom is lost.

See also: [fft], page 475, [ifft], page 476, [fft2], page 477, [ifft2], page 477, [fftn], page 477, [ifftn], page 477.

`ifft` (*a*, *n*, *dim*) [Loadable Function]

Compute the inverse FFT of *a* using subroutines from FFTW. The inverse FFT is calculated along the first non-singleton dimension of the array. Thus if *a* is a matrix, `fft (a)` computes the inverse FFT for each column of *a*.

If called with two arguments, *n* is expected to be an integer specifying the number of elements of *a* to use, or an empty matrix to specify that its value should be ignored. If *n* is larger than the dimension along which the inverse FFT is calculated, then *a* is resized and padded with zeros. Otherwise, if*n* is smaller than the dimension along which the inverse FFT is calculated, then *a* is truncated.

If called with three arguments, *dim* is an integer specifying the dimension of the matrix along which the inverse FFT is performed

See also: [fft], page 475, [ifft2], page 477, [ifftn], page 477, [fftw], page 475.

`fft2 (a, n, m)` [Loadable Function]

Compute the two-dimensional FFT of *a* using subroutines from FFTW. The optional arguments *n* and *m* may be used specify the number of rows and columns of *a* to use. If either of these is larger than the size of *a*, *a* is resized and padded with zeros.

If *a* is a multi-dimensional matrix, each two-dimensional sub-matrix of *a* is treated separately

See also: ifft2, fft, fftn, fftw.

`fft2 (a, n, m)` [Loadable Function]

Compute the inverse two-dimensional FFT of *a* using subroutines from FFTW. The optional arguments *n* and *m* may be used specify the number of rows and columns of *a* to use. If either of these is larger than the size of *a*, *a* is resized and padded with zeros.

If *a* is a multi-dimensional matrix, each two-dimensional sub-matrix of *a* is treated separately

See also: fft2, ifft, ifftn, fftw.

`fftn (a, size)` [Loadable Function]

Compute the N-dimensional FFT of *a* using subroutines from FFTW. The optional vector argument *size* may be used specify the dimensions of the array to be used. If an element of *size* is smaller than the corresponding dimension, then the dimension is truncated prior to performing the FFT. Otherwise if an element of *size* is larger than the corresponding dimension *a* is resized and padded with zeros.

See also: ifftn, fft, fft2, fftw.

`ifftn (a, size)` [Loadable Function]

Compute the inverse N-dimensional FFT of *a* using subroutines from FFTW. The optional vector argument *size* may be used specify the dimensions of the array to be used. If an element of *size* is smaller than the corresponding dimension, then the dimension is truncated prior to performing the inverse FFT. Otherwise if an element of *size* is larger than the corresponding dimension *a* is resized and padded with zeros.

See also: fftn, ifft, ifft2, fftw.

`fftconv (a, b, n)` [Function File]

Return the convolution of the vectors *a* and *b*, as a vector with length equal to the `length (a) + length (b) - 1`. If *a* and *b* are the coefficient vectors of two polynomials, the returned value is the coefficient vector of the product polynomial.

The computation uses the FFT by calling the function `fftfilt`. If the optional argument *n* is specified, an N-point FFT is used.

`fftfilt (b, x, n)` [Function File]

With two arguments, `fftfilt` filters *x* with the FIR filter *b* using the FFT.

Given the optional third argument, *n*, `fftfilt` uses the overlap-add method to filter *x* with *b* using an N-point FFT.

If *x* is a matrix, filter each column of the matrix.

`y = filter (b, a, x)` [Loadable Function]
`[y, sf] = filter (b, a, x, si)` [Loadable Function]
`[y, sf] = filter (b, a, x, [], dim)` [Loadable Function]
`[y, sf] = filter (b, a, x, si, dim)` [Loadable Function]

Return the solution to the following linear, time-invariant difference equation:

$$\sum_{k=0}^{N} a_{k+1} y_{n-k} = \sum_{k=0}^{M} b_{k+1} x_{n-k}, \qquad 1 \le n \le P$$

where $a \in \Re^{N-1}$, $b \in \Re^{M-1}$, and $x \in \Re^{P}$. over the first non-singleton dimension of *x* or over *dim* if supplied. An equivalent form of this equation is:

$$y_n = -\sum_{k=1}^{N} c_{k+1} y_{n-k} + \sum_{k=0}^{M} d_{k+1} x_{n-k}, \qquad 1 \le n \le P$$

where $c = a/a_1$ and $d = b/a_1$.

If the fourth argument *si* is provided, it is taken as the initial state of the system and the final state is returned as *sf*. The state vector is a column vector whose length is equal to the length of the longest coefficient vector minus one. If *si* is not supplied, the initial state vector is set to all zeros.

In terms of the z-transform, y is the result of passing the discrete- time signal x through a system characterized by the following rational system function:

$$H(z) = \frac{\sum_{k=0}^{M} d_{k+1} z^{-k}}{1 + \sum_{k+1}^{N} c_{k+1} z^{-k}}$$

`y = filter2 (b, x)` [Function File]
`y = filter2 (b, x, shape)` [Function File]

Apply the 2-D FIR filter *b* to *x*. If the argument *shape* is specified, return an array of the desired shape. Possible values are:

'full' pad *x* with zeros on all sides before filtering.

'same' unpadded *x* (default)

'valid' trim *x* after filtering so edge effects are no included.

Note this is just a variation on convolution, with the parameters reversed and *b* rotated 180 degrees.

See also: [conv2], page 447.

`[h, w] = freqz (b, a, n, "whole")` [Function File]

Return the complex frequency response *h* of the rational IIR filter whose numerator and denominator coefficients are *b* and *a*, respectively. The response is evaluated at *n* angular frequencies between 0 and 2π.

The output value *w* is a vector of the frequencies.

If the fourth argument is omitted, the response is evaluated at frequencies between 0 and π.

If *n* is omitted, a value of 512 is assumed.

If *a* is omitted, the denominator is assumed to be 1 (this corresponds to a simple FIR filter).

For fastest computation, *n* should factor into a small number of small primes.

`h = freqz (b, a, w)` [Function File]

Evaluate the response at the specific frequencies in the vector *w*. The values for *w* are measured in radians.

`[...] = freqz (..., Fs)` [Function File]

Return frequencies in Hz instead of radians assuming a sampling rate *Fs*. If you are evaluating the response at specific frequencies *w*, those frequencies should be requested in Hz rather than radians.

`freqz (...)` [Function File]

Plot the pass band, stop band and phase response of *h* rather than returning them.

`freqz_plot (w, h)` [Function File]

Plot the pass band, stop band and phase response of *h*.

`sinc (x)` [Function File]

Return $\sin(\pi x)/(\pi x)$.

`b = unwrap (a, tol, dim)` [Function File]

Unwrap radian phases by adding multiples of 2*pi as appropriate to remove jumps greater than *tol*. *tol* defaults to pi.

Unwrap will unwrap along the first non-singleton dimension of *a*, unless the optional argument *dim* is given, in which case the data will be unwrapped along this dimension

`[a, b] = arch_fit (y, x, p, iter, gamma, a0, b0)` [Function File]

Fit an ARCH regression model to the time series *y* using the scoring algorithm in Engle's original ARCH paper. The model is

```
y(t) = b(1) * x(t,1) + ... + b(k) * x(t,k) + e(t),
h(t) = a(1) + a(2) * e(t-1)^2 + ... + a(p+1) * e(t-p)^2
```

in which $e(t)$ is $N(0, h(t))$, given a time-series vector *y* up to time $t-1$ and a matrix of (ordinary) regressors *x* up to *t*. The order of the regression of the residual variance is specified by *p*.

If invoked as `arch_fit (y, k, p)` with a positive integer *k*, fit an ARCH(*k*, *p*) process, i.e., do the above with the *t*-th row of *x* given by

```
[1, y(t-1), ..., y(t-k)]
```

Optionally, one can specify the number of iterations *iter*, the updating factor *gamma*, and initial values *a*0 and *b*0 for the scoring algorithm.

`arch_rnd` (*a*, *b*, *t*) [Function File]

Simulate an ARCH sequence of length *t* with AR coefficients *b* and CH coefficients *a*. I.e., the result $y(t)$ follows the model

```
y(t) = b(1) + b(2) * y(t-1) + ... + b(lb) * y(t-lb+1) + e(t),
```

where $e(t)$, given y up to time $t-1$, is $N(0, h(t))$, with

```
h(t) = a(1) + a(2) * e(t-1)^2 + ... + a(la) * e(t-la+1)^2
```

`[pval, lm] = arch_test` (*y*, *x*, *p*) [Function File]

For a linear regression model

```
y = x * b + e
```

perform a Lagrange Multiplier (LM) test of the null hypothesis of no conditional heteroscedascity against the alternative of CH(*p*).

I.e., the model is

```
y(t) = b(1) * x(t,1) + ... + b(k) * x(t,k) + e(t),
```

given y up to $t-1$ and *x* up to *t*, *e*(t) is $N(0, h(t))$ with

```
h(t) = v + a(1) * e(t-1)^2 + ... + a(p) * e(t-p)^2,
```

and the null is $a(1) == \ldots == a(p) == 0$.

If the second argument is a scalar integer, *k*, perform the same test in a linear autoregression model of order *k*, i.e., with

```
[1, y(t-1), ..., y(t-k)]
```

as the *t*-th row of *x*.

Under the null, LM approximately has a chisquare distribution with *p* degrees of freedom and *pval* is the *p*-value (1 minus the CDF of this distribution at LM) of the test.

If no output argument is given, the *p*-value is displayed.

`arma_rnd` (*a*, *b*, *v*, *t*, *n*) [Function File]

Return a simulation of the ARMA model

```
x(n) = a(1) * x(n-1) + ... + a(k) * x(n-k)
     + e(n) + b(1) * e(n-1) + ... + b(l) * e(n-l)
```

in which *k* is the length of vector *a*, *l* is the length of vector *b* and *e* is Gaussian white noise with variance *v*. The function returns a vector of length *t*.

The optional parameter *n* gives the number of dummy $x(i)$ used for initialization, i.e., a sequence of length *t*+*n* is generated and *x*(*n*+1:*t*+*n*) is returned. If *n* is omitted, *n* = 100 is used.

`autocor` (*x*, *h*) [Function File]

Return the autocorrelations from lag 0 to *h* of vector *x*. If *h* is omitted, all autocorrelations are computed. If *x* is a matrix, the autocorrelations of each column are computed.

`autocov` (*x*, *h*) [Function File]

Return the autocovariances from lag 0 to *h* of vector *x*. If *h* is omitted, all autocovariances are computed. If *x* is a matrix, the autocovariances of each column are computed.

`autoreg_matrix (y, k)` [Function File]

Given a time series (vector) *y*, return a matrix with ones in the first column and the first *k* lagged values of *y* in the other columns. I.e., for *t* > *k*, `[1, y(t-1), ..., y(t-k)]` is the t-th row of the result. The resulting matrix may be used as a regressor matrix in autoregressions.

`bartlett (m)` [Function File]

Return the filter coefficients of a Bartlett (triangular) window of length *m*.

For a definition of the Bartlett window, see e.g., A. V. Oppenheim & R. W. Schafer, *Discrete-Time Signal Processing*.

`blackman (m)` [Function File]

Return the filter coefficients of a Blackman window of length *m*.

For a definition of the Blackman window, see e.g., A. V. Oppenheim & R. W. Schafer, *Discrete-Time Signal Processing*.

`[d, dd] = diffpara (x, a, b)` [Function File]

Return the estimator *d* for the differencing parameter of an integrated time series.

The frequencies from $[2 * pi * a/t, 2 * pi * b/T]$ are used for the estimation. If *b* is omitted, the interval $[2 * pi/T, 2 * pi * a/T]$ is used. If both *b* and *a* are omitted then $a = 0.5 * sqrt(T)$ and $b = 1.5 * sqrt(T)$ is used, where *T* is the sample size. If *x* is a matrix, the differencing parameter of each column is estimated.

The estimators for all frequencies in the intervals described above is returned in *dd*. The value of *d* is simply the mean of *dd*.

Reference: Brockwell, Peter J. & Davis, Richard A. Time Series: Theory and Methods Springer 1987.

`durbinlevinson (c, oldphi, oldv)` [Function File]

Perform one step of the Durbin-Levinson algorithm.

The vector *c* specifies the autocovariances `[gamma_0, ..., gamma_t]` from lag 0 to *t*, *oldphi* specifies the coefficients based on *c*(*t*-1) and *oldv* specifies the corresponding error.

If *oldphi* and *oldv* are omitted, all steps from 1 to *t* of the algorithm are performed.

`fftshift (v)` [Function File]
`fftshift (v, dim)` [Function File]

Perform a shift of the vector *v*, for use with the `fft` and `ifft` functions, in order the move the frequency 0 to the center of the vector or matrix.

If *v* is a vector of *N* elements corresponding to *N* time samples spaced of *Dt* each, then `fftshift (fft (v))` corresponds to frequencies

```
f = ((1:N) - ceil(N/2)) / N / Dt
```

If *v* is a matrix, the same holds for rows and columns. If *v* is an array, then the same holds along each dimension.

The optional *dim* argument can be used to limit the dimension along which the permutation occurs.

ifftshift (*v*) [Function File]
ifftshift (*v*, *dim*) [Function File]

Undo the action of the `fftshift` function. For even length *v*, `fftshift` is its own inverse, but odd lengths differ slightly.

fractdiff (*x*, *d*) [Function File]

Compute the fractional differences $(1 - L)^d x$ where L denotes the lag-operator and d is greater than -1.

hamming (*m*) [Function File]

Return the filter coefficients of a Hamming window of length *m*.

For a definition of the Hamming window, see e.g., A. V. Oppenheim & R. W. Schafer, *Discrete-Time Signal Processing*.

hanning (*m*) [Function File]

Return the filter coefficients of a Hanning window of length *m*.

For a definition of this window type, see e.g., A. V. Oppenheim & R. W. Schafer, *Discrete-Time Signal Processing*.

hurst (*x*) [Function File]

Estimate the Hurst parameter of sample *x* via the rescaled range statistic. If *x* is a matrix, the parameter is estimated for every single column.

pp = **pchip** (*x*, *y*) [Function File]
yi = **pchip** (*x*, *y*, *xi*) [Function File]

Piecewise Cubic Hermite interpolating polynomial. Called with two arguments, the piece-wise polynomial *pp* is returned, that may later be used with `ppval` to evaluate the polynomial at specific points.

The variable *x* must be a strictly monotonic vector (either increasing or decreasing). While *y* can be either a vector or array. In the case where *y* is a vector, it must have a length of *n*. If *y* is an array, then the size of *y* must have the form

$$[s_1, s_2, \cdots, s_k, n]$$

The array is then reshaped internally to a matrix where the leading dimension is given by

$$s_1 s_2 \cdots s_k$$

and each row in this matrix is then treated separately. Note that this is exactly the opposite treatment than `interp1` and is done for compatibility.

Called with a third input argument, `pchip` evaluates the piece-wise polynomial at the points *xi*. There is an equivalence between `ppval (pchip (x, y), xi)` and `pchip (x, y, xi)`.

See also: [spline], page 458, [ppval], page 451, [mkpp], page 451, [unmkpp], page 451.

periodogram (*x*) [Function File]

For a data matrix *x* from a sample of size *n*, return the periodogram.

`rectangle_lw (n, b)` [Function File]

Rectangular lag window. Subfunction used for spectral density estimation.

`rectangle_sw (n, b)` [Function File]

Rectangular spectral window. Subfunction used for spectral density estimation.

`sinetone (freq, rate, sec, ampl)` [Function File]

Return a sinetone of frequency *freq* with length of *sec* seconds at sampling rate *rate* and with amplitude *ampl*. The arguments *freq* and *ampl* may be vectors of common size.

Defaults are *rate* = 8000, *sec* = 1 and *ampl* = 64.

`sinewave (m, n, d)` [Function File]

Return an *m*-element vector with *i*-th element given by `sin (2 * pi * (i+d-1) / n)`.

The default value for *d* is 0 and the default value for *n* is *m*.

`spectral_adf (c, win, b)` [Function File]

Return the spectral density estimator given a vector of autocovariances *c*, window name *win*, and bandwidth, *b*.

The window name, e.g., `"triangle"` or `"rectangle"` is used to search for a function called `win_sw`.

If *win* is omitted, the triangle window is used. If *b* is omitted, `1 / sqrt (length (x))` is used.

`spectral_xdf (x, win, b)` [Function File]

Return the spectral density estimator given a data vector *x*, window name *win*, and bandwidth, *b*.

The window name, e.g., `"triangle"` or `"rectangle"` is used to search for a function called `win_sw`.

If *win* is omitted, the triangle window is used. If *b* is omitted, `1 / sqrt (length (x))` is used.

`spencer (x)` [Function File]

Return Spencer's 15 point moving average of every single column of *x*.

`[y, c] = stft (x, win_size, inc, num_coef, w_type)` [Function File]

Compute the short-time Fourier transform of the vector *x* with *num_coef* coefficients by applying a window of *win_size* data points and an increment of *inc* points.

Before computing the Fourier transform, one of the following windows is applied:

hanning w_type = 1

hamming w_type = 2

rectangle w_type = 3

The window names can be passed as strings or by the *w_type* number.

If not all arguments are specified, the following defaults are used: *win_size* = 80, *inc* = 24, *num_coef* = 64, and *w_type* = 1.

y = `stft (`*x*`, ...)` returns the absolute values of the Fourier coefficients according to the *num_coef* positive frequencies.

`[`*y*`, `*c*`] = stft (x, ...)` returns the entire STFT-matrix *y* and a 3-element vector *c* containing the window size, increment, and window type, which is needed by the synthesis function.

`synthesis` (*y*, *c*) [Function File]

Compute a signal from its short-time Fourier transform *y* and a 3-element vector *c* specifying window size, increment, and window type.

The values *y* and *c* can be derived by

```
[y, c] = stft (x , ...)
```

`triangle_lw` (*n*, *b*) [Function File]

Triangular lag window. Subfunction used for spectral density estimation.

`triangle_sw` (*n*, *b*) [Function File]

Triangular spectral window. Subfunction used for spectral density estimation.

`[a, v] = yulewalker` (*c*) [Function File]

Fit an AR (p)-model with Yule-Walker estimates given a vector *c* of autocovariances `[gamma_0, ..., gamma_p]`.

Returns the AR coefficients, *a*, and the variance of white noise, *v*.

31 Image Processing

Since an image basically is a matrix Octave is a very powerful environment for processing and analyzing images. To illustrate how easy it is to do image processing in Octave, the following example will load an image, smooth it by a 5-by-5 averaging filter, and compute the gradient of the smoothed image.

```
I = imread ("myimage.jpg");
S = conv2 (I, ones (5, 5) / 25, "same");
[Dx, Dy] = gradient (S);
```

In this example `S` contains the smoothed image, and `Dx` and `Dy` contains the partial spatial derivatives of the image.

31.1 Loading and Saving Images

The first step in most image processing tasks is to load an image into Octave. This is done using the `imread` function, which uses the `GraphicsMagick` library for reading. This means a vast number of image formats is supported. The `imwrite` function is the corresponding function for writing images to the disk.

In summary, most image processing code will follow the structure of this code

```
I = imread ("my_input_image.img");
J = process_my_image (I);
imwrite ("my_output_image.img", J);
```

`[`*`img`*`, `*`map`*`, `*`alpha`*`] = imread (`*`filename`*`)` [Function File]

Read images from various file formats.

The size and numeric class of the output depends on the format of the image. A color image is returned as an MxNx3 matrix. Grey-level and black-and-white images are of size MxN. The color depth of the image determines the numeric class of the output: "uint8" or "uint16" for grey and color, and "logical" for black and white.

See also: [imwrite], page 485, [imfinfo], page 486.

`imwrite (`*`img`*`, `*`filename`*`, `*`fmt`*`, `*`p1`*`, `*`v1`*`, ...)` [Function File]
`imwrite (`*`img`*`, `*`map`*`, `*`filename`*`, `*`fmt`*`, `*`p1`*`, `*`v1`*`, ...)` [Function File]

Write images in various file formats.

If *fmt* is missing, the file extension (if any) of *filename* is used to determine the format.

The parameter-value pairs (*p1*, *v1*, . . .) are optional. Currently the following options are supported for `JPEG` images

‘`Quality`’ Sets the quality of the compression. The corresponding value should be an integer between 0 and 100, with larger values meaning higher visual quality and less compression.

See also: [imread], page 485, [imfinfo], page 486.

`val`` = IMAGE_PATH ()` [Built-in Function]
`old_val`` = IMAGE_PATH (`*`new_val`*`)` [Built-in Function]

Query or set the internal variable that specifies a colon separated list of directories in which to search for image files.

It is possible to get information about an image file on disk, without actually reading it into Octave. This is done using the `imfinfo` function which provides read access to many of the parameters stored in the header of the image file.

info = imfinfo (*filename*) [Function File]
info = imfinfo (*url*) [Function File]

Read image information from a file.

`imfinfo` returns a structure containing information about the image stored in the file *filename*. The output structure contains the following fields.

'`Filename`'
: The full name of the image file.

'`FileSize`'
: Number of bytes of the image on disk

'`FileModDate`'
: Date of last modification to the file.

'`Height`'
: Image height in pixels.

'`Width`'
: Image Width in pixels.

'`BitDepth`'
: Number of bits per channel per pixel.

'`Format`'
: Image format (e.g., `"jpeg"`).

'`LongFormat`'
: Long form image format description.

'`XResolution`'
: X resolution of the image.

'`YResolution`'
: Y resolution of the image.

'`TotalColors`'
: Number of unique colors in the image.

'`TileName`'
: Tile name.

'`AnimationDelay`'
: Time in 1/100ths of a second (0 to 65535) which must expire before displaying the next image in an animated sequence.

'`AnimationIterations`'
: Number of iterations to loop an animation (e.g., Netscape loop extension) for.

'`ByteOrder`'
: Endian option for formats that support it. Is either `"little-endian"`, `"big-endian"`, or `"undefined"`.

'Gamma' Gamma level of the image. The same color image displayed on two different workstations may look different due to differences in the display monitor.

'Matte' `true` if the image has transparency.

'ModulusDepth'
Image modulus depth (minimum number of bits required to support red/green/blue components without loss of accuracy).

'Quality' JPEG/MIFF/PNG compression level.

'QuantizeColors'
Preferred number of colors in the image.

'ResolutionUnits'
Units of image resolution. Is either `"pixels per inch"`, `"pixels per centimeter"`, or `"undefined"`.

'ColorType'
Image type. Is either `"grayscale"`, `"indexed"`, `"truecolor"`, or `"undefined"`.

'View' FlashPix viewing parameters.

See also: [imread], page 485, [imwrite], page 485.

31.2 Displaying Images

A natural part of image processing is visualization of an image. The most basic function for this is the `imshow` function that shows the image given in the first input argument. This function uses an external program to show the image. If gnuplot 4.2 or later is available it will be used to display the image, otherwise the `display`, `xv`, or `xloadimage` program is used. The actual program can be selected with the `image_viewer` function.

`imshow (`*im*`)` [Function File]
`imshow (`*im*`, `*limits*`)` [Function File]
`imshow (`*im*`, `*map*`)` [Function File]
`imshow (`*rgb*`, ...)` [Function File]
`imshow (`*filename*`)` [Function File]
`imshow (..., `*string_param1*`, `*value1*`, ...)` [Function File]

Display the image *im*, where *im* can be a 2-dimensional (gray-scale image) or a 3-dimensional (RGB image) matrix.

If *limits* is a 2-element vector `[`*low*`, `*high*`]`, the image is shown using a display range between *low* and *high*. If an empty matrix is passed for *limits*, the display range is computed as the range between the minimal and the maximal value in the image.

If *map* is a valid color map, the image will be shown as an indexed image using the supplied color map.

If a file name is given instead of an image, the file will be read and shown.

If given, the parameter *string_param1* has value *value1*. *string_param1* can be any of the following:

'"displayrange"'
value1 is the display range as described above.

See also: [image], page 488, [imagesc], page 488, [colormap], page 490, [gray2ind], page 489, [rgb2ind], page 489.

`image (img)` [Function File]
`image (x, y, img)` [Function File]

Display a matrix as a color image. The elements of *x* are indices into the current colormap, and the colormap will be scaled so that the extremes of *x* are mapped to the extremes of the colormap.

It first tries to use `gnuplot`, then `display` from `ImageMagick`, then `xv`, and then `xloadimage`. The actual program used can be changed using the `image_viewer` function.

The axis values corresponding to the matrix elements are specified in *x* and *y*. If you're not using gnuplot 4.2 or later, these variables are ignored.

See also: [imshow], page 487, [imagesc], page 488, [colormap], page 490, [image_viewer], page 488.

`imagesc (a)` [Function File]
`imagesc (x, y, a)` [Function File]
`imagesc (..., limits)` [Function File]
`imagesc (h, ...)` [Function File]
`h = imagesc (...)` [Function File]

Display a scaled version of the matrix *a* as a color image. The colormap is scaled so that the entries of the matrix occupy the entire colormap. If *limits* = [*lo*, *hi*] are given, then that range is set to the 'clim' of the current axes.

The axis values corresponding to the matrix elements are specified in *x* and *y*, either as pairs giving the minimum and maximum values for the respective axes, or as values for each row and column of the matrix *a*.

See also: [image], page 488, [imshow], page 487, [caxis], page 222.

`[fcn, default_zoom] = image_viewer (fcn, default_zoom)` [Function File]

Change the program or function used for viewing images and return the previous values.

When the `image` or `imshow` function is called it will launch an external program to display the image. The default behavior is to use gnuplot if the installed version supports image viewing, and otherwise try the programs `display`, `xv`, and `xloadimage`. Using this function it is possible to change that behavior.

When called with one input argument images will be displayed by saving the image to a file and the system command *command* will be called to view the image. The *command* must be a string containing `%s` and possibly `%f`. The `%s` will be replaced by the filename of the image, and the `%f` will (if present) be replaced by the zoom factor given to the `image` function. For example,

```
image_viewer ("eog %s");
```

changes the image viewer to the `eog` program.

With two input arguments, images will be displayed by calling the function *function_handle*. For example,

```
image_viewer (data, @my_image_viewer);
```

sets the image viewer function to `my_image_viewer`. The image viewer function is called with

```
my_image_viewer (x, y, im, zoom, data)
```

where *x* and *y* are the axis of the image, *im* is the image variable, and *data* is extra user-supplied data to be passed to the viewer function.

With three input arguments it is possible to change the zooming. Some programs (like `xloadimage`) require the zoom factor to be between 0 and 100, and not 0 and 1 like Octave assumes. This is solved by setting the third argument to 100.

See also: [image], page 488, [imshow], page 487.

31.3 Representing Images

In general Octave supports four different kinds of images, gray-scale images, RGB images, binary images, and indexed images. A gray-scale image is represented with an M-by-N matrix in which each element corresponds to the intensity of a pixel. An RGB image is represented with an M-by-N-by-3 array where each 3-vector corresponds to the red, green, and blue intensities of each pixel.

The actual meaning of the value of a pixel in a gray-scale or RGB image depends on the class of the matrix. If the matrix is of class `double` pixel intensities are between 0 and 1, if it is of class `uint8` intensities are between 0 and 255, and if it is of class `uint16` intensities are between 0 and 65535.

A binary image is an M-by-N matrix of class `logical`. A pixel in a binary image is black if it is `false` and white if it is `true`.

An indexed image consists of an M-by-N matrix of integers and a C-by-3 color map. Each integer corresponds to an index in the color map, and each row in the color map corresponds to an RGB color. The color map must be of class `double` with values between 0 and 1.

`[`*img*`, `*map*`] = gray2ind (`*I*, *n*`)` [Function File]

Convert a gray scale intensity image to an Octave indexed image. The indexed image will consist of *n* different intensity values. If not given *n* will default to 64.

`ind2gray (`*x*, *map*`)` [Function File]

Convert an Octave indexed image to a gray scale intensity image. If *map* is omitted, the current colormap is used to determine the intensities.

See also: [gray2ind], page 489, [rgb2ntsc], page 494, [image], page 488, [colormap], page 490.

`[`*x*`, `*map*`] = rgb2ind (`*rgb*`)` [Function File]
`[`*x*`, `*map*`] = rgb2ind (`*r*, *g*, *b*`)` [Function File]

Convert an RGB image to an Octave indexed image.

See also: [ind2rgb], page 490, [rgb2ntsc], page 494.

rgb = `ind2rgb` (*x*, *map*) [Function File]
[*r*, *g*, *b*] = `ind2rgb` (*x*, *map*) [Function File]

Convert an indexed image to red, green, and blue color components. If the colormap doesn't contain enough colors, pad it with the last color in the map. If *map* is omitted, the current colormap is used for the conversion.

See also: [rgb2ind], page 489, [image], page 488, [imshow], page 487, [ind2gray], page 489, [gray2ind], page 489.

`colormap` (*map*) [Function File]
`colormap` ("*default*") [Function File]

Set the current colormap.

`colormap (map)` sets the current colormap to *map*. The color map should be an *n* row by 3 column matrix. The columns contain red, green, and blue intensities respectively. All entries should be between 0 and 1 inclusive. The new colormap is returned.

`colormap ("default")` restores the default colormap (the `jet` map with 64 entries). The default colormap is returned.

With no arguments, `colormap` returns the current color map.

See also: [jet], page 491.

map_out = `brighten` (*map*, *beta*) [Function File]
map_out = `brighten` (*h*, *beta*) [Function File]
map_out = `brighten` (*beta*) [Function File]

Darkens or brightens the given colormap. If the *map* argument is omitted, the function is applied to the current colormap. The first argument can also be a valid graphics handle *h*, in which case `brighten` is applied to the colormap associated with this handle.

Should the resulting colormap *map_out* not be assigned, it will be written to the current colormap.

The argument *beta* should be a scalar between -1 and 1, where a negative value darkens and a positive value brightens the colormap.

See also: [colormap], page 490.

`autumn` (*n*) [Function File]

Create color colormap. This colormap is red through orange to yellow. The argument *n* should be a scalar. If it is omitted, the length of the current colormap or 64 is assumed.

See also: [colormap], page 490.

`bone` (*n*) [Function File]

Create color colormap. This colormap is a gray colormap with a light blue tone. The argument *n* should be a scalar. If it is omitted, the length of the current colormap or 64 is assumed.

See also: [colormap], page 490.

`cool (n)` [Function File]

Create color colormap. The colormap is cyan to magenta. The argument *n* should be a scalar. If it is omitted, the length of the current colormap or 64 is assumed.

See also: [colormap], page 490.

`copper (n)` [Function File]

Create color colormap. This colormap is black to a light copper tone. The argument *n* should be a scalar. If it is omitted, the length of the current colormap or 64 is assumed.

See also: [colormap], page 490.

`flag (n)` [Function File]

Create color colormap. This colormap cycles through red, white, blue and black. The argument *n* should be a scalar. If it is omitted, the length of the current colormap or 64 is assumed.

See also: [colormap], page 490.

`gray (n)` [Function File]

Return a gray colormap with *n* entries corresponding to values from 0 to *n*-1. The argument *n* should be a scalar. If it is omitted, the length of the current colormap or 64 is assumed.

`hot (n)` [Function File]

Create color colormap. This colormap is black through dark red, red, orange, yellow to white. The argument *n* should be a scalar. If it is omitted, the length of the current colormap or 64 is assumed.

See also: [colormap], page 490.

`hsv (n)` [Function File]

Create color colormap. This colormap is red through yellow, green, cyan, blue, magenta to red. It is obtained by linearly varying the hue through all possible values while keeping constant maximum saturation and value and is equivalent to `hsv2rgb ([linspace(0,1,N)', ones(N,2)])`.

The argument *n* should be a scalar. If it is omitted, the length of the current colormap or 64 is assumed.

See also: [colormap], page 490.

`jet (n)` [Function File]

Create color colormap. This colormap is dark blue through blue, cyan, green, yellow, red to dark red. The argument *n* should be a scalar. If it is omitted, the length of the current colormap or 64 is assumed.

See also: [colormap], page 490.

`ocean (n)` [Function File]

Create color colormap. The argument *n* should be a scalar. If it is omitted, the length of the current colormap or 64 is assumed.

`pink (n)` [Function File]

Create color colormap. This colormap gives a sepia tone on black and white images. The argument *n* should be a scalar. If it is omitted, the length of the current colormap or 64 is assumed.

See also: [colormap], page 490.

`prism (n)` [Function File]

Create color colormap. This colormap cycles trough red, orange, yellow, green, blue and violet. The argument *n* should be a scalar. If it is omitted, the length of the current colormap or 64 is assumed.

See also: [colormap], page 490.

`rainbow (n)` [Function File]

Create color colormap. This colormap is red through orange, yellow, green, blue to violet. The argument *n* should be a scalar. If it is omitted, the length of the current colormap or 64 is assumed.

See also: [colormap], page 490.

`spring (n)` [Function File]

Create color colormap. This colormap is magenta to yellow. The argument *n* should be a scalar. If it is omitted, the length of the current colormap or 64 is assumed.

See also: [colormap], page 490.

`summer (n)` [Function File]

Create color colormap. This colormap is green to yellow. The argument *n* should be a scalar. If it is omitted, the length of the current colormap or 64 is assumed.

See also: [colormap], page 490.

`white (n)` [Function File]

Create color colormap. This colormap is completely white. The argument *n* should be a scalar. If it is omitted, the length of the current colormap or 64 is assumed.

See also: [colormap], page 490.

`winter (n)` [Function File]

Create color colormap. This colormap is blue to green. The argument *n* should be a scalar. If it is omitted, the length of the current colormap or 64 is assumed.

See also: [colormap], page 490.

`contrast (x, n)` [Function File]

Return a gray colormap that maximizes the contrast in an image. The returned colormap will have *n* rows. If *n* is not defined then the size of the current colormap is used instead.

See also: [colormap], page 490.

An additional colormap is `gmap40`. This code map contains only colors with integer values of the red, green and blue components. This is a workaround for a limitation of gnuplot 4.0, that does not allow the color of line or patch objects to be set, and so `gmap40` is useful for gnuplot 4.0 users, and in particular in conjunction with the *bar*, *barh* or *contour* functions.

`gmap40 (`*n*`)` [Function File]

Create a color colormap. The colormap is red, green, blue, yellow, magenta and cyan. These are the colors that are allowed with patch objects using gnuplot 4.0, and so this colormap function is specially designed for users of gnuplot 4.0. The argument *n* should be a scalar. If it is omitted, a length of 6 is assumed. Larger values of *n* result in a repetition of the above colors

See also: [colormap], page 490.

You may use the `spinmap` function to cycle through the colors in the current colormap, displaying the changes for the current figure.

`spinmap (`*t*, *inc*`)` [Function File]

Cycle the colormap for *t* seconds with an increment of *inc*. Both parameters are optional. The default cycle time is 5 seconds and the default increment is 2.

A higher value of *inc* causes a faster cycle through the colormap.

See also: [gca], page 244, [colorbar], page 238.

31.4 Plotting on top of Images

If gnuplot is being used to display images it is possible to plot on top of images. Since an image is a matrix it is indexed by row and column values. The plotting system is, however, based on the traditional (x, y) system. To minimize the difference between the two systems Octave places the origin of the coordinate system in the point corresponding to the pixel at $(1, 1)$. So, to plot points given by row and column values on top of an image, one should simply call `plot` with the column values as the first argument and the row values as the second. As an example the following code generates an image with random intensities between 0 and 1, and shows the image with red circles over pixels with an intensity above 0.99.

```
I = rand (100, 100);
[row, col] = find (I > 0.99);
hold ("on");
imshow (I);
plot (col, row, "ro");
hold ("off");
```

31.5 Color Conversion

Octave supports conversion from the RGB color system to NTSC and HSV and vice versa.

hsv_map `= rgb2hsv (`*rgb_map*`)` [Function File]

Transform a colormap or image from the rgb space to the hsv space.

A color n the RGB space consists of the red, green and blue intensities.

In the HSV space each color is represented by their hue, saturation and value (brightness). Value gives the amount of light in the color. Hue describes the dominant wavelength. Saturation is the amount of Hue mixed into the color.

See also: [hsv2rgb], page 494.

rgb_map = `hsv2rgb` (*hsv_map*) [Function File]

Transform a colormap or image from the hsv space to the rgb space.

See also: [rgb2hsv], page 493.

`rgb2ntsc` (*rgb*) [Function File]

Transform a colormap or image from RGB to NTSC.

See also: [ntsc2rgb], page 494.

`ntsc2rgb` (*yiq*) [Function File]

Transform a colormap or image from NTSC to RGB.

See also: [rgb2ntsc], page 494.

32 Audio Processing

Octave provides a few functions for dealing with audio data. An audio 'sample' is a single output value from an A/D converter, i.e., a small integer number (usually 8 or 16 bits), and audio data is just a series of such samples. It can be characterized by three parameters: the sampling rate (measured in samples per second or Hz, e.g., 8000 or 44100), the number of bits per sample (e.g., 8 or 16), and the number of channels (1 for mono, 2 for stereo, etc.).

There are many different formats for representing such data. Currently, only the two most popular, *linear encoding* and *mu-law encoding*, are supported by Octave. There is an excellent FAQ on audio formats by Guido van Rossum <guido@cwi.nl> which can be found at any FAQ ftp site, in particular in the directory '`/pub/usenet/news.answers/audio-fmts`' of the archive site `rtfm.mit.edu`.

Octave simply treats audio data as vectors of samples (non-mono data are not supported yet). It is assumed that audio files using linear encoding have one of the extensions '`lin`' or '`raw`', and that files holding data in mu-law encoding end in '`au`', '`mu`', or '`snd`'.

`lin2mu` (*x*, *n*) [Function File]

Converts audio data from linear to mu-law. Mu-law values use 8-bit unsigned integers. Linear values use *n*-bit signed integers or floating point values in the range -1<=*x*<=1 if *n* is 0. If *n* is not specified it defaults to 0, 8 or 16 depending on the range values in *x*.

See also: [mu2lin], page 495, [loadaudio], page 495, [saveaudio], page 495, [playaudio], page 496, [setaudio], page 496, [record], page 496.

`mu2lin` (*x*, *bps*) [Function File]

Converts audio data from linear to mu-law. Mu-law values are 8-bit unsigned integers. Linear values use *n*-bit signed integers or floating point values in the range -1<=y<=1 if *n* is 0. If *n* is not specified it defaults to 8.

See also: [lin2mu], page 495, [loadaudio], page 495, [saveaudio], page 495, [playaudio], page 496, [setaudio], page 496, [record], page 496.

`loadaudio` (*name*, *ext*, *bps*) [Function File]

Loads audio data from the file '*name.ext*' into the vector *x*.

The extension *ext* determines how the data in the audio file is interpreted; the extensions '`lin`' (default) and '`raw`' correspond to linear, the extensions '`au`', '`mu`', or '`snd`' to mu-law encoding.

The argument *bps* can be either 8 (default) or 16, and specifies the number of bits per sample used in the audio file.

See also: [lin2mu], page 495, [mu2lin], page 495, [saveaudio], page 495, [playaudio], page 496, [setaudio], page 496, [record], page 496.

`saveaudio` (*name*, *x*, *ext*, *bps*) [Function File]

Saves a vector *x* of audio data to the file '*name.ext*'. The optional parameters *ext* and *bps* determine the encoding and the number of bits per sample used in the audio file (see `loadaudio`); defaults are '`lin`' and 8, respectively.

See also: [lin2mu], page 495, [mu2lin], page 495, [loadaudio], page 495, [playaudio], page 496, [setaudio], page 496, [record], page 496.

The following functions for audio I/O require special A/D hardware and operating system support. It is assumed that audio data in linear encoding can be played and recorded by reading from and writing to '`/dev/dsp`', and that similarly '`/dev/audio`' is used for mu-law encoding. These file names are system-dependent. Improvements so that these functions will work without modification on a wide variety of hardware are welcome.

`playaudio` (*name*, *ext*) [Function File]
`playaudio` (*x*) [Function File]

Plays the audio file '*name.ext*' or the audio data stored in the vector *x*.

See also: [lin2mu], page 495, [mu2lin], page 495, [loadaudio], page 495, [saveaudio], page 495, [setaudio], page 496, [record], page 496.

`record` (*sec*, *sampling_rate*) [Function File]

Records *sec* seconds of audio input into the vector *x*. The default value for *sampling_rate* is 8000 samples per second, or 8kHz. The program waits until the user types RET and then immediately starts to record.

See also: [lin2mu], page 495, [mu2lin], page 495, [loadaudio], page 495, [saveaudio], page 495, [playaudio], page 496, [setaudio], page 496.

`setaudio` ([*w_type* [, *value*]]) [Function File]

Execute the shell command '`mixer [w_type [, value]]`'

y `= wavread` (*filename*) [Function File]

Load the RIFF/WAVE sound file *filename*, and return the samples in vector *y*. If the file contains multichannel data, then *y* is a matrix with the channels represented as columns.

`[`*y*`,` *Fs*`,` *bits*`] = wavread` (*filename*) [Function File]

Additionally return the sample rate (*fs*) in Hz and the number of bits per sample (*bits*).

`[...] = wavread` (*filename*, *n*) [Function File]

Read only the first *n* samples from each channel.

`[...] = wavread` (*filename*,[*n1 n2*]) [Function File]

Read only samples *n1* through *n2* from each channel.

`[`*samples*`,` *channels*`] = wavread` (*filename*, "*size*") [Function File]

Return the number of samples (*n*) and channels (*ch*) instead of the audio data.

See also: [wavwrite], page 496.

`wavwrite` (*y*, *filename*) [Function File]
`wavwrite` (*y*, *fs*, *filename*) [Function File]
`wavwrite` (*y*, *fs*, *bits*, *filename*) [Function File]

Write *y* to the canonical RIFF/WAVE sound file *filename* with sample rate *fs* and bits per sample *bits*. The default sample rate is 8000 Hz with 16-bits per sample. Each column of the data represents a separate channel.

See also: [wavread], page 496.

33 Object Oriented Programming

Octave includes the capability to include user classes, including the features of operator and function overloading. Equally a user class can be used to encapsulate certain properties of the class so that they cannot be altered accidentally and can be set up to address the issue of class precedence in mixed class operations.

This chapter discussions the means of constructing a user class with the example of a polynomial class, how to query and set the properties of this class, together with the means to overload operators and functions.

33.1 Creating a Class

We use in the following text a polynomial class to demonstrate the use of object oriented programming within Octave. This class was chosen as it is simple, and so doesn't distract unnecessarily from the discussion of the programming features of Octave. However, even still a small understand of the polynomial class itself is necessary to fully grasp the techniques described.

The polynomial class is used to represent polynomials of the form

$$a_0 \;+\; a_1\;x \;+\; a_2\;x^2 \;+\; \ldots\; a_n\;x^n$$

where a_0, a_1, etc. are elements of $\Re$. Thus the polynomial can be represented by a vector

```
a = [a0, a1, a2, ..., an];
```

We therefore now have sufficient information about the requirements of the class constructor for our polynomial class to write it. All object oriented classes in Octave, must be contained with a directory taking the name of the class, prepended with the `@` symbol. For example, with our polynomial class, we would place the methods defining the class in the `@`polynomial directory.

The constructor of the class, must have the name of the class itself and so in our example the constructor with have the name '`@polynomial/polynomial.m`'. Also ideally when the constructor is called with no arguments to should return a value object. So for example our polynomial might look like

```
## -*- texinfo -*-
## @deftypefn {Function File} {} polynomial ()
## @deftypefnx {Function File} {} polynomial (@var{a})
## Creates a polynomial object representing the polynomial
##
## @example
## a0 + a1 * x + a2 * x^2 + @dots{} + an * x^n
## @end example
##
## from a vector of coefficients [a0 a1 a2 ...  an].
## @end deftypefn

function p = polynomial (a)
  if (nargin == 0)
    p.poly = [0];
    p = class (p, "polynomial");
  elseif (nargin == 1)
    if (strcmp (class (a), "polynomial"))
      p = a;
    elseif (isvector (a) && isreal (a))
      p.poly = a(:).';
      p = class (p, "polynomial");
    else
      error ("polynomial:  expecting real vector");
    endif
  else
    print_usage ();
  endif
endfunction
```

Note that the return value of the constructor must be the output of the `class` function called with the first argument being a structure and the second argument being the class name. An example of the call to this constructor function is then

```
p = polynomial ([1, 0, 1]);
```

Note that methods of a class can be documented. The help for the constructor itself can be obtained with the constructor name, that is for the polynomial constructor `help polynomial` will return the help string. Also the help can be obtained by restricting the search for the help to a particular class, for example `help @polynomial/polynomial`. This second method is the only means of getting help for the overloaded methods and functions of the class.

The same is true for other Octave functions that take a function name as an argument. For example `type @polynomial/display` will print the code of the display method of the polynomial class to the screen, and `dbstop @polynomial/display` will set a breakpoint at the first executable line of the display method of the polynomial class.

To check where a variable is a user class, the `isobject` and `isa` functions can be used. for example

```
p = polynomial ([1, 0, 1]);
isobject (p)
⇒ 1
isa (p, "polynomial")
⇒ 1
```

`isobject` (*x*) [Built-in Function]

Return true if *x* is a class object.

The available methods of a class can be displayed with the `methods` function.

`methods` (*x*) [Built-in Function]
`methods` ("*classname*") [Built-in Function]

Return a cell array containing the names of the methods for the object *x* or the named class.

To inquire whether a particular method is available to a user class, the `ismethod` function can be used.

`ismethod` (*x*, *method*) [Built-in Function]

Return true if *x* is a class object and the string *method* is a method of this class.

For example

```
p = polynomial ([1, 0, 1]);
ismethod (p, "roots")
⇒ 1
```

33.2 Manipulating Classes

There are a number of basic classes methods that can be defined to allow the contents of the classes to be queried and set. The most basic of these is the `display` method. The `display` method is used by Octave when displaying a class on the screen, due to an expression that is not terminated with a semicolon. If this method is not defined, then Octave will printed nothing when displaying the contents of a class.

`display (a)` [Function File]

Display the contents of an object. If *a* is an object of the class "myclass", then `display` is called in a case like

```
myclass (...)
```

where Octave is required to display the contents of a variable of the type "myclass".

See also: [class], page 33, [subsref], page 502, [subsasgn], page 504.

An example of a display method for the polynomial class might be

```
function display (p)
  a = p.poly;
  first = true;
  fprintf("%s =", inputname(1));
  for i = 1 :  length (a);
    if (a(i) != 0)
      if (first)
        first = false;
      elseif (a(i) > 0)
        fprintf (" +");
      endif
      if (a(i) < 0)
        fprintf (" -");
      endif
      if (i == 1)
        fprintf (" %g", abs (a(i)));
      elseif (abs(a(i)) != 1)
        fprintf (" %g *", abs (a(i)));
      endif
      if (i > 1)
        fprintf (" X");
      endif
      if (i > 2)
        fprintf (" ^ %d", i - 1);
      endif
    endif
  endfor
  if (first)
    fprintf(" 0");
  endif
  fprintf("\n");
endfunction
```

Note that in the display method, it makes sense to start the method with the line `fprintf("%s =", inputname(1))` to be consistent with the rest of Octave and print the variable name to be displayed when displaying the class.

To be consistent with the Octave graphic handle classes, a class should also define the `get` and `set` methods. The `get` method should accept one or two arguments, and given one argument of the appropriate class it should return a structure with all of the properties of the class. For example

```
function s = get (p, f)
  if (nargin == 1)
    s.poly = p.poly;
  elseif (nargin == 2)
    if (ischar (f))
      switch (f)
        case "poly"
          s = p.poly;
        otherwise
          error ("get:  invalid property %s", f);
      endswitch
    else
      error ("get:  expecting the property to be a string");
    endif
  else
    print_usage ();
  endif
endfunction
```

Similarly, the `set` method should taken as its first argument an object to modify, and then take property/value pairs to be modified.

```
function s = set (p, varargin)
  s = p;
  if (length (varargin) < 2 || rem (length (varargin), 2) != 0)
    error ("set:  expecting property/value pairs");
  endif
  while (length (varargin) > 1)
    prop = varargin{1};
    val = varargin{2};
    varargin(1:2) = [];
    if (ischar (prop) && strcmp (prop, "poly"))
      if (isvector (val) && isreal (val))
        s.poly = val(:).';
      else
        error ("set:  expecting the value to be a real vector");
      endif
    else
      error ("set:  invalid property of polynomial class");
    endif
  endwhile
endfunction
```

Note that as Octave does not implement pass by reference, than the modified object is the return value of the `set` method and it must be called like

```
p = set (p, "a", [1, 0, 0, 0, 1]);
```

Also the `set` method makes use of the `subsasgn` method of the class, and this method must be defined. The `subsasgn` method is discussed in the next section.

Finally, user classes can be considered as a special type of a structure, and so they can be saved to a file in the same manner as a structure. For example

```
p = polynomial ([1, 0, 1]);
save userclass.mat p
clear p
load userclass.mat
```

All of the file formats supported by `save` and `load` are supported. In certain circumstances, a user class might either contain a field that it makes no sense to save or a field that needs to be initialized before it is saved. This can be done with the `saveobj` method of the class

b = saveobj (*a*) [Function File]

Method of a class to manipulate an object prior to saving it to a file. The function `saveobj` is called when the object *a* is saved using the `save` function. An example of the use of `saveobj` might be to remove fields of the object that don't make sense to be saved or it might be used to ensure that certain fields of the object are initialized before the object is saved. For example

```
function b = saveobj (a)
  b = a;
  if (isempty (b.field))
     b.field = initfield(b);
  endif
endfunction
```

See also: [loadobj], page 502, [class], page 33.

`saveobj` is called just prior to saving the class to a file. Likely, the `loadobj` method is called just after a class is loaded from a file, and can be used to ensure that any removed fields are reinserted into the user object.

b = loadobj (*a*) [Function File]

Method of a class to manipulate an object after loading it from a file. The function `loadobj` is called when the object *a* is loaded using the `load` function. An example of the use of `saveobj` might be to add fields to an object that don't make sense to be saved. For example

```
function b = loadobj (a)
  b = a;
  b.addmissingfield = addfield (b);
endfunction
```

See also: [saveobj], page 502, [class], page 33.

33.3 Indexing Objects

Objects can be indexed with parentheses, either like `a (`*idx*`)` or like `a {`*idx*`}`, or even like `a (`*idx*`).`*field*. However, it is up to the user to decide what this indexing actually means. In the case of our polynomial class *p* (*n*) might mean either the coefficient of the *n*-th power of the polynomial, or it might be the evaluation of the polynomial at *n*. The meaning of this subscripted referencing is determined by the `subsref` method.

`subsref` (*val*, *idx*) [Built-in Function]

Perform the subscripted element selection operation according to the subscript specified by *idx*.

The subscript *idx* is expected to be a structure array with fields '`type`' and '`subs`'. Valid values for '`type`' are '`"()"`', '`"{}"`', and '`"."`'. The '`subs`' field may be either '`":"`' or a cell array of index values.

The following example shows how to extract the two first columns of a matrix

```
val = magic(3)
     ⇒ val = [ 8   1   6
               3   5   7
               4   9   2 ]
idx.type = "()";
idx.subs = {":", 1:2};
subsref(val, idx)
     ⇒ [ 8   1
         3   5
         4   9 ]
```

Note that this is the same as writing `val(:,1:2)`.

See also: [subsasgn], page 504, [substruct], page 84.

For example we might decide that indexing with `"()"` evaluates the polynomial and indexing with `"{}"` returns the n-th coefficient (of n-th power). In this case the `subsref` method of our polynomial class might look like

```
function b = subsref (a, s)
  if (isempty (s))
    error ("polynomial:  missing index");
  endif
  switch (s(1).type)
    case "()"
      ind = s(1).subs;
      if (numel (ind) != 1)
        error ("polynomial:  need exactly one index");
      else
        b = polyval (fliplr (a.poly), ind{1});
      endif
    case "{}"
      ind = s(1).subs;
      if (numel (ind) != 1)
        error ("polynomial:  need exactly one index");
      else
        if (isnumeric (ind{1}))
          b = a.poly(ind{1}+1);
        else
          b = a.poly(ind{1});
        endif
      endif
    case "."
      fld = s.subs;
      if (strcmp (fld, "poly"))
        b = a.poly;
      else
        error ("@polynomial/subsref:  invalid property \"%s\"", fld);
      endif
    otherwise
      error ("invalid subscript type");
  endswitch
  if (numel (s) > 1)
    b = subsref (b, s(2:end));
  endif
endfunction
```

The equivalent functionality for subscripted assignments uses the `subsasgn` method.

`subsasgn` (*val*, *idx*, *rhs*) [Built-in Function]

Perform the subscripted assignment operation according to the subscript specified by *idx*.

The subscript *idx* is expected to be a structure array with fields '`type`' and '`subs`'. Valid values for '`type`' are '`"()"`', '`"{}"`', and '`"."`'. The '`subs`' field may be either '`":"`' or a cell array of index values.

The following example shows how to set the two first columns of a 3-by-3 matrix to zero.

```
val = magic(3);
idx.type = "()";
idx.subs = {":", 1:2};
subsasgn (val, idx, 0)
     ⇒ [ 0   0   6
         0   0   7
         0   0   2 ]
```

Note that this is the same as writing `val(:,1:2) = 0`.

See also: [subsref], page 502, [substruct], page 84.

Note that the `subsref` and `subsasgn` methods always receive the whole index chain, while they usually handle only the first element. It is the responsibility of these methods to handle the rest of the chain (if needed), usually by forwarding it again to `subsref` or `subsasgn`.

If you wish to use the `end` keyword in subscripted expressions of an object, then the user needs to define the `end` method for the class.

For example the `end` method for our polynomial class might look like

```
function r = end (obj, index_pos, num_indices)

  if (num_indices != 1)
    error ("polynomial object may only have one index")
  endif

  r = length (obj.poly) - 1;

endfunction
```

which is a fairly generic `end` method that has a behavior similar to the `end` keyword for Octave Array classes. It can then be used for example like

```
p = polynomial([1,2,3,4]);
p(end-1)
⇒ 3
```

Objects can also be used as the index in a subscripted expression themselves and this is controlled with the `subsindex` function.

idx = `subsindex (a)` [Function File]

Convert an object to an index vector. When *a* is a class object defined with a class constructor, then `subsindex` is the overloading method that allows the conversion of this class object to a valid indexing vector. It is important to note that `subsindex` must return a zero-based real integer vector of the class "double". For example, if the class constructor

```
function b = myclass (a)
 b = myclass (struct ("a", a), "myclass");
endfunction
```

then the `subsindex` function

```
function idx = subsindex (a)
 idx = double (a.a) - 1.0;
endfunction
```

can then be used as follows

```
a = myclass (1:4);
b = 1:10;
b(a)
⇒ 1  2  3  4
```

See also: [class], page 33, [subsref], page 502, [subsasgn], page 504.

Finally, objects can equally be used like ranges, using the `colon` method

r = `colon` (*a*, *b*) [Function File]
r = `colon` (*a*, *b*, *c*) [Function File]

Method of a class to construct a range with the `:` operator. For example.

```
a = myclass (...)
b = myclass (...)
c = a : b
```

See also: [class], page 33, [subsref], page 502, [subsasgn], page 504.

33.4 Overloading Objects

33.4.1 Function Overloading

Any Octave function can be overloaded, and allows a object specific version of this function to be called as needed. A pertinent example for our polynomial class might be to overload the `polyval` function like

```
function [y, dy] = polyval (p, varargin)
  if (nargout == 2)
    [y, dy] = polyval (fliplr(p.poly), varargin{:});
  else
    y = polyval (fliplr(p.poly), varargin{:});
  endif
endfunction
```

This function just hands off the work to the normal Octave `polyval` function. Another interesting example for an overloaded function for our polynomial class is the `plot` function.

```
function h = plot(p, varargin)
  n = 128;
  rmax = max (abs (roots (p.poly)));
  x = [0 :  (n - 1)] / (n - 1) * 2.2 * rmax - 1.1 * rmax;
  if (nargout > 0)
    h = plot(x, p(x), varargin{:});
  else
    plot(x, p(x), varargin{:});
  endif
endfunction
```

which allows polynomials to be plotted in the domain near the region of the roots of the polynomial.

Functions that are of particular interest to be overloaded are the class conversion functions such as `double`. Overloading these functions allows the `cast` function to work with the user class and can aid in the use of methods of other classes with the user class. An example `double` function for our polynomial class might look like.

```
function b = double (a)
  b = a.poly;
endfunction
```

33.4.2 Operator Overloading

Operation	Method	Description
$a + b$	plus (a, b)	Binary addition operator
$a - b$	minus (a, b)	Binary subtraction operator
$+a$	uplus (a)	Unary addition operator
$-a$	uminus (a)	Unary subtraction operator
$a. * b$	times (a, b)	Element-wise multiplication operator
$a * b$	mtimes (a, b)	Matrix multiplication operator
$a./b$	rdivide (a, b)	Element-wise right division operator
a/b	mrdivide (a, b)	Matrix right division operator
$a.\backslash b$	ldivide (a, b)	Element-wise left division operator
$a\backslash b$	mldivide (a, b)	Matrix left division operator
$a.\hat{}b$	power (a, b)	Element-wise power operator
$a\hat{}b$	mpower (a, b)	Matrix power operator
$a < b$	lt (a, b)	Less than operator
$a <= b$	le (a, b)	Less than or equal to operator
$a > b$	gt (a, b)	Greater than operator
$a >= b$	ge (a, b)	Greater than or equal to operator
$a == b$	eq (a, b)	Equal to operator
$a! = b$	ne (a, b)	Not equal to operator
$a\&b$	and (a, b)	Logical and operator
$a\|b$	or (a, b)	Logical or operator
$!b$	not (a)	Logical not operator
a'	ctranspose (a)	Complex conjugate transpose operator
$a.'$	transpose (a)	Transpose operator
$a : b$	colon (a, b)	Two element range operator
$a : b : c$	colon (a, b, c)	Three element range operator
$[a, b]$	horzcat (a, b)	Horizontal concatenation operator
$[a; b]$	vertcat (a, b)	Vertical concatenation operator
$a(s_1, \ldots, s_n)$	subsref (a, s)	Subscripted reference
$a(s_1, \ldots, s_n) = b$	subsasgn (a, s, b)	Subscripted assignment
$b(a)$	subsindex (a)	Convert to zero-based index
display	display (a)	Commandline display function

Table 33.1: Available overloaded operators and their corresponding class method

An example `mtimes` method for our polynomial class might look like

```
function y = mtimes (a, b)
  y = polynomial (conv (double(a),double(b)));
endfunction
```

33.4.3 Precedence of Objects

Many functions and operators take two or more arguments and so the case can easily arise that these functions are called with objects of different classes. It is therefore necessary to

determine the precedence of which method of which class to call when there are mixed objects given to a function or operator. To do this the `superiorto` and `inferiorto` functions can be used

`superiorto` (*class_name*, . . .) [Built-in Function]

> When called from a class constructor, mark the object currently constructed as having a higher precedence than *class_name*. More that one such class can be specified in a single call. This function may only be called from a class constructor.

`inferiorto` (*class_name*, . . .) [Built-in Function]

> When called from a class constructor, mark the object currently constructed as having a lower precedence than *class_name*. More that one such class can be specified in a single call. This function may only be called from a class constructor.

For example with our polynomial class consider the case

```
2 * polynomial ([1, 0, 1]);
```

That mixes an object of the class "double" with an object of the class "polynomial". In this case we like to ensure that the return type of the above is of the type "polynomial" and so we use the `superiorto` function in the class constructor. In particular our polynomial class constructor would be modified to be

```
## -*- texinfo -*-
## @deftypefn {Function File} {} polynomial ()
## @deftypefnx {Function File} {} polynomial (@var{a})
## Creates a polynomial object representing the polynomial
##
## @example
## a0 + a1 * x + a2 * x^2 + @dots{} + an * x^n
## @end example
##
## from a vector of coefficients [a0 a1 a2 ...  an].
## @end deftypefn

function p = polynomial (a)
  if (nargin == 0)
    p.poly = [0];
    p = class (p, "polynomial");
  elseif (nargin == 1)
    if (strcmp (class (a), "polynomial"))
      p = a;
    elseif (isvector (a) && isreal (a))
      p.poly = a(:).';
      p = class (p, "polynomial");
    else
      error ("polynomial:  expecting real vector");
    endif
  else
    print_usage ();
  endif
  superiorto ("double");
endfunction
```

Note that user classes always have higher precedence than built-in Octave types. So in fact marking our polynomial class higher than the "double" class is in fact not necessary.

33.5 Inheritance and Aggregation

Using classes to build new classes is supported by octave through the use of both inheritance and aggregation.

Class inheritance is provided by octave using the `class` function in the class constructor. As in the case of the polynomial class, the octave programmer will create a struct that contains the data fields required by the class, and then call the class function to indicate that an object is to be created from the struct. Creating a child of an existing object is done by creating an object of the parent class and providing that object as the third argument of the class function.

This is easily demonstrated by example. Suppose the programmer needs an FIR filter, i.e. a filter with a numerator polynomial but a unity denominator polynomial. In traditional octave programming, this would be performed as follows.

```
octave:1> x = [some data vector];
octave:2> n = [some coefficient vector];
octave:3> y = filter (n, 1, x);
```

The equivalent class could be implemented in a class directory `@FIRfilter` that is on the octave path. The constructor is a file FIRfilter.m in the class directory.

```
## -*- texinfo -*-
## @deftypefn {Function File} {} FIRfilter ()
## @deftypefnx {Function File} {} FIRfilter (@var{p})
## Creates an FIR filter with polynomial @var{p} as
## coefficient vector.
##
## @end deftypefn

function f = FIRfilter (p)

  f.polynomial = [];
  if (nargin == 0)
    p = @polynomial ([1]);
  elseif (nargin == 1)
    if (!isa (p, "polynomial"))
      error ("FIRfilter:  expecting polynomial as input argument");
    endif
  else
    print_usage ();
  endif
  f = class (f, "FIRfilter", p);
endfunction
```

As before, the leading comments provide command-line documentation for the class constructor. This constructor is very similar to the polynomial class constructor, except that we pass a polynomial object as the third argument to the class function, telling octave that the FIRfilter class will be derived from the polynomial class. Our FIR filter does not have any data fields, but we must provide a struct to the `class` function. The `class` function will add an element named polynomial to the object struct, so we simply add a dummy element named polynomial as the first line of the constructor. This dummy element will be overwritten by the class function.

Note further that all our examples provide for the case in which no arguments are supplied. This is important since octave will call the constructor with no arguments when loading ojects from save files to determine the inheritance structure.

A class may be a child of more than one class (see the documentation for the `class` function), and inheritance may be nested. There is no limitation to the number of parents or the level of nesting other than memory or other physical issues.

As before, we need a `display` method. A simple example might be

```
function display (f)

  display(f.polynomial);

endfunction
```

Note that we have used the polynomial field of the struct to display the filter coefficients.

Once we have the class constructor and display method, we may create an object by calling the class constructor. We may also check the class type and examine the underlying structure.

```
octave:1> f=FIRfilter(polynomial([1 1 1]/3))
f.polynomial = 0.333333 + 0.333333 * X + 0.333333 * X ^ 2
octave:2> class(f)
ans = FIRfilter
octave:3> isa(f,"FIRfilter")
ans =  1
octave:4> isa(f,"polynomial")
ans =  1
octave:5> struct(f)
ans =
{
polynomial = 0.333333 + 0.333333 * X + 0.333333 * X ^ 2
}
```

We only need to define a method to actually process data with our filter and our class is usable. It is also useful to provide a means of changing the data stored in the class. Since the fields in the underlying struct are private by default, we could provide a mechanism to access the fields. The `subsref` method may be used for both.

```
function out = subsref (f, x)
  switch x.type
    case "()"
      n = f.polynomial;
      out = filter(n.poly, 1, x.subs{1});
    case "."
      fld = x.subs;
      if (strcmp (fld, "polynomial"))
        out = f.polynomial;
      else
        error ("@FIRfilter/subsref:  invalid property \"%s\"", fld);
      endif
    otherwise
      error ("@FIRfilter/subsref:  invalid subscript type for FIR filter");
  endswitch
endfunction
```

The "()" case allows us to filter data using the polynomial provided to the constructor.

```
octave:2> f=FIRfilter(polynomial([1 1 1]/3));
```

```
octave:3> x=ones(5,1);
octave:4> y=f(x)
y =

   0.33333
   0.66667
   1.00000
   1.00000
   1.00000
```

The "." case allows us to view the contents of the polynomial field.

```
octave:1> f=FIRfilter(polynomial([1 1 1]/3));
octave:2> f.polynomial
ans = 0.333333 + 0.333333 * X + 0.333333 * X ^ 2
```

In order to change the contents of the object, we need to define a `subsasgn` method. For example, we may make the polynomial field publicly writeable.

```
function out = subsasgn (f, index, val)
  switch (index.type)
    case "."
      fld = index.subs;
      if (strcmp (fld, "polynomial"))
        out = f;
        out.polynomial = val;
      else
        error ("@FIRfilter/subsref:  invalid property \"%s\"", fld);
      endif
    otherwise
      error ("FIRfilter/subsagn:  Invalid index type")
  endswitch
endfunction
```

So that

```
octave:6> f=FIRfilter();
octave:7> f.polynomial = polynomial([1 2 3]);
f.polynomial = 1 + 2 * X + 3 * X ^ 2
```

Defining the FIRfilter class as a child of the polynomial class implies that and FIRfilter object may be used any place that a polynomial may be used. This is not a normal use of a filter, so that aggregation may be a more sensible design approach. In this case, the polynomial is simply a field in the class structure. A class constructor for this case might be

```
## -*- texinfo -*-
## @deftypefn {Function File} {} FIRfilter ()
## @deftypefnx {Function File} {} FIRfilter (@var{p})
## Creates an FIR filter with polynomial @var{p} as
## coefficient vector.
##
## @end deftypefn

function f = FIRfilter (p)

  if (nargin == 0)
    f.polynomial = @polynomial ([1]);
  elseif (nargin == 1)
    if (isa (p, "polynomial"))
      f.polynomial = p;
    else
      error ("FIRfilter:  expecting polynomial as input argument");
    endif
  else
    print_usage ();
  endif
  f = class (f, "FIRfilter");
endfunction
```

For our example, the remaining class methods remain unchanged.

34 System Utilities

This chapter describes the functions that are available to allow you to get information about what is happening outside of Octave, while it is still running, and use this information in your program. For example, you can get information about environment variables, the current time, and even start other programs from the Octave prompt.

34.1 Timing Utilities

Octave's core set of functions for manipulating time values are patterned after the corresponding functions from the standard C library. Several of these functions use a data structure for time that includes the following elements:

`usec` Microseconds after the second (0-999999).

`sec` Seconds after the minute (0-61). This number can be 61 to account for leap seconds.

`min` Minutes after the hour (0-59).

`hour` Hours since midnight (0-23).

`mday` Day of the month (1-31).

`mon` Months since January (0-11).

`year` Years since 1900.

`wday` Days since Sunday (0-6).

`yday` Days since January 1 (0-365).

`isdst` Daylight Savings Time flag.

`zone` Time zone.

In the descriptions of the following functions, this structure is referred to as a *tm_struct*.

`time ()` [Loadable Function]

Return the current time as the number of seconds since the epoch. The epoch is referenced to 00:00:00 CUT (Coordinated Universal Time) 1 Jan 1970. For example, on Monday February 17, 1997 at 07:15:06 CUT, the value returned by `time` was 856163706.

See also: [strftime], page 517, [strptime], page 519, [localtime], page 516, [gmtime], page 516, [mktime], page 517, [now], page 515, [date], page 519, [clock], page 519, [datenum], page 521, [datestr], page 521, [datevec], page 523, [calendar], page 523, [weekday], page 523.

`t = now ()` [Function File]

Returns the current local time as the number of days since Jan 1, 0000. By this reckoning, Jan 1, 1970 is day number 719529.

The integral part, `floor (now)` corresponds to 00:00:00 today.

The fractional part, `rem (now, 1)` corresponds to the current time on Jan 1, 0000.

The returned value is also called a "serial date number" (see `datenum`).

See also: [clock], page 519, [date], page 519, [datenum], page 521.

`ctime (t)` [Function File]

Convert a value returned from `time` (or any other non-negative integer), to the local time and return a string of the same form as `asctime`. The function `ctime (time)` is equivalent to `asctime (localtime (time))`. For example,

```
ctime (time ())
     ⇒ "Mon Feb 17 01:15:06 1997\n"
```

`gmtime (t)` [Loadable Function]

Given a value returned from time (or any non-negative integer), return a time structure corresponding to CUT. For example,

```
gmtime (time ())
     ⇒ {
           usec = 0
           year = 97
           mon = 1
           mday = 17
           sec = 6
           zone = CST
           min = 15
           wday = 1
           hour = 7
           isdst = 0
           yday = 47
         }
```

See also: [strftime], page 517, [strptime], page 519, [localtime], page 516, [mktime], page 517, [time], page 515, [now], page 515, [date], page 519, [clock], page 519, [datenum], page 521, [datestr], page 521, [datevec], page 523, [calendar], page 523, [weekday], page 523.

`localtime (t)` [Loadable Function]

Given a value returned from time (or any non-negative integer), return a time structure corresponding to the local time zone.

```
localtime (time ())
     ⇒ {
           usec = 0
           year = 97
           mon = 1
           mday = 17
           sec = 6
           zone = CST
           min = 15
           wday = 1
           hour = 1
           isdst = 0
           yday = 47
         }
```

See also: [strftime], page 517, [strptime], page 519, [gmtime], page 516, [mktime], page 517, [time], page 515, [now], page 515, [date], page 519, [clock], page 519, [datenum], page 521, [datestr], page 521, [datevec], page 523, [calendar], page 523, [weekday], page 523.

`mktime (`*`tm_struct`*`)` [Loadable Function]

Convert a time structure corresponding to the local time to the number of seconds since the epoch. For example,

```
mktime (localtime (time ()))
     ⇒ 856163706
```

See also: [strftime], page 517, [strptime], page 519, [localtime], page 516, [gmtime], page 516, [time], page 515, [now], page 515, [date], page 519, [clock], page 519, [datenum], page 521, [datestr], page 521, [datevec], page 523, [calendar], page 523, [weekday], page 523.

`asctime (`*`tm_struct`*`)` [Function File]

Convert a time structure to a string using the following five-field format: Thu Mar 28 08:40:14 1996. For example,

```
asctime (localtime (time ()))
     ⇒ "Mon Feb 17 01:15:06 1997\n"
```

This is equivalent to `ctime (time ())`.

`strftime (`*`fmt`*`, `*`tm_struct`*`)` [Loadable Function]

Format the time structure *tm_struct* in a flexible way using the format string *fmt* that contains '`%`' substitutions similar to those in `printf`. Except where noted, substituted fields have a fixed size; numeric fields are padded if necessary. Padding is with zeros by default; for fields that display a single number, padding can be changed or inhibited by following the '`%`' with one of the modifiers described below. Unknown field specifiers are copied as normal characters. All other characters are copied to the output without change. For example,

```
strftime ("%r (%Z) %A %e %B %Y", localtime (time ()))
     ⇒ "01:15:06 AM (CST) Monday 17 February 1997"
```

Octave's `strftime` function supports a superset of the ANSI C field specifiers.

Literal character fields:

`%` % character.

`n` Newline character.

`t` Tab character.

Numeric modifiers (a nonstandard extension):

`- (dash)` Do not pad the field.

`_ (underscore)`
Pad the field with spaces.

Time fields:

`%H` Hour (00-23).

%I Hour (01-12).

%k Hour (0-23).

%l Hour (1-12).

%M Minute (00-59).

%p Locale's AM or PM.

%r Time, 12-hour (hh:mm:ss [AP]M).

%R Time, 24-hour (hh:mm).

%s Time in seconds since 00:00:00, Jan 1, 1970 (a nonstandard extension).

%S Second (00-61).

%T Time, 24-hour (hh:mm:ss).

%X Locale's time representation (%H:%M:%S).

%Z Time zone (EDT), or nothing if no time zone is determinable.

Date fields:

%a Locale's abbreviated weekday name (Sun-Sat).

%A Locale's full weekday name, variable length (Sunday-Saturday).

%b Locale's abbreviated month name (Jan-Dec).

%B Locale's full month name, variable length (January-December).

%c Locale's date and time (Sat Nov 04 12:02:33 EST 1989).

%C Century (00-99).

%d Day of month (01-31).

%e Day of month (1-31).

%D Date (mm/dd/yy).

%h Same as %b.

%j Day of year (001-366).

%m Month (01-12).

%U Week number of year with Sunday as first day of week (00-53).

%w Day of week (0-6).

%W Week number of year with Monday as first day of week (00-53).

%x Locale's date representation (mm/dd/yy).

%y Last two digits of year (00-99).

%Y Year (1970-).

See also: [strptime], page 519, [localtime], page 516, [gmtime], page 516, [mktime], page 517, [time], page 515, [now], page 515, [date], page 519, [clock], page 519, [datenum], page 521, [datestr], page 521, [datevec], page 523, [calendar], page 523, [weekday], page 523.

`[tm_struct, nchars] = strptime (str, fmt)` [Loadable Function]

Convert the string *str* to the time structure *tm_struct* under the control of the format string *fmt*.

If *fmt* fails to match, *nchars* is 0; otherwise it is set to the position of last matched character plus 1. Always check for this unless you're absolutely sure the date string will be parsed correctly.

See also: [strftime], page 517, [localtime], page 516, [gmtime], page 516, [mktime], page 517, [time], page 515, [now], page 515, [date], page 519, [clock], page 519, [datenum], page 521, [datestr], page 521, [datevec], page 523, [calendar], page 523, [weekday], page 523.

Most of the remaining functions described in this section are not patterned after the standard C library. Some are available for compatibility with MATLAB and others are provided because they are useful.

`clock ()` [Function File]

Return a vector containing the current year, month (1-12), day (1-31), hour (0-23), minute (0-59) and second (0-61). For example,

```
clock ()
     ⇒ [ 1993, 8, 20, 4, 56, 1 ]
```

The function clock is more accurate on systems that have the `gettimeofday` function.

`date ()` [Function File]

Return the date as a character string in the form DD-MMM-YY. For example,

```
date ()
     ⇒ "20-Aug-93"
```

`etime (t1, t2)` [Function File]

Return the difference (in seconds) between two time values returned from `clock`. For example:

```
t0 = clock ();
 many computations later...
elapsed_time = etime (clock (), t0);
```

will set the variable `elapsed_time` to the number of seconds since the variable `t0` was set.

See also: [tic], page 520, [toc], page 520, [clock], page 519, [cputime], page 519.

`[total, user, system] = cputime ();` [Built-in Function]

Return the CPU time used by your Octave session. The first output is the total time spent executing your process and is equal to the sum of second and third outputs, which are the number of CPU seconds spent executing in user mode and the number of CPU seconds spent executing in system mode, respectively. If your system does not have a way to report CPU time usage, `cputime` returns 0 for each of its output values. Note that because Octave used some CPU time to start, it is reasonable to check to see if `cputime` works by checking to see if the total CPU time used is nonzero.

`is_leap_year (year)` [Function File]

Return 1 if the given year is a leap year and 0 otherwise. If no arguments are provided, `is_leap_year` will use the current year. For example,

```
is_leap_year (2000)
     ⇒ 1
```

`tic ()` [Built-in Function]
`toc ()` [Built-in Function]

Set or check a wall-clock timer. Calling `tic` without an output argument sets the timer. Subsequent calls to `toc` return the number of seconds since the timer was set. For example,

```
tic ();
# many computations later...
elapsed_time = toc ();
```

will set the variable `elapsed_time` to the number of seconds since the most recent call to the function `tic`.

If called with one output argument then this function returns a scalar of type `uint64` and the wall-clock timer is not started.

```
t = tic; sleep (5); (double (tic ()) - double (t)) * 1e-6
     ⇒ 5
```

Nested timing with `tic` and `toc` is not supported. Therefore `toc` will always return the elapsed time from the most recent call to `tic`.

If you are more interested in the CPU time that your process used, you should use the `cputime` function instead. The `tic` and `toc` functions report the actual wall clock time that elapsed between the calls. This may include time spent processing other jobs or doing nothing at all. For example,

```
tic (); sleep (5); toc ()
     ⇒ 5
t = cputime (); sleep (5); cputime () - t
     ⇒ 0
```

(This example also illustrates that the CPU timer may have a fairly coarse resolution.)

`pause (seconds)` [Built-in Function]

Suspend the execution of the program. If invoked without any arguments, Octave waits until you type a character. With a numeric argument, it pauses for the given number of seconds. For example, the following statement prints a message and then waits 5 seconds before clearing the screen.

```
fprintf (stderr, "wait please...\n");
pause (5);
clc;
```

`sleep (seconds)` [Built-in Function]

Suspend the execution of the program for the given number of seconds.

`usleep (microseconds)` [Built-in Function]

Suspend the execution of the program for the given number of microseconds. On systems where it is not possible to sleep for periods of time less than one second, `usleep` will pause the execution for `round (microseconds / 1e6)` seconds.

`datenum (year, month, day)` [Function File]
`datenum (year, month, day, hour)` [Function File]
`datenum (year, month, day, hour, minute)` [Function File]
`datenum (year, month, day, hour, minute, second)` [Function File]
`datenum ("date")` [Function File]
`datenum ("date", p)` [Function File]

Returns the specified local time as a day number, with Jan 1, 0000 being day 1. By this reckoning, Jan 1, 1970 is day number 719529. The fractional portion, *p*, corresponds to the portion of the specified day.

Notes:

- Years can be negative and/or fractional.
- Months below 1 are considered to be January.
- Days of the month start at 1.
- Days beyond the end of the month go into subsequent months.
- Days before the beginning of the month go to the previous month.
- Days can be fractional.

Warning: this function does not attempt to handle Julian calendars so dates before Octave 15, 1582 are wrong by as much as eleven days. Also be aware that only Roman Catholic countries adopted the calendar in 1582. It took until 1924 for it to be adopted everywhere. See the Wikipedia entry on the Gregorian calendar for more details.

Warning: leap seconds are ignored. A table of leap seconds is available on the Wikipedia entry for leap seconds.

See also: [date], page 519, [clock], page 519, [now], page 515, [datestr], page 521, [datevec], page 523, [calendar], page 523, [weekday], page 523.

`str = datestr (date, [f, [p]])` [Function File]

Format the given date/time according to the format `f` and return the result in *str*. *date* is a serial date number (see `datenum`) or a date vector (see `datevec`). The value of *date* may also be a string or cell array of strings.

f can be an integer which corresponds to one of the codes in the table below, or a date format string.

p is the year at the start of the century in which two-digit years are to be interpreted in. If not specified, it defaults to the current year minus 50.

For example, the date 730736.65149 (2000-09-07 15:38:09.0934) would be formatted as follows:

Code	Format	Example
0	dd-mmm-yyyy HH:MM:SS	07-Sep-2000 15:38:09

1	dd-mmm-yyyy	07-Sep-2000
2	mm/dd/yy	09/07/00
3	mmm	Sep
4	m	S
5	mm	09
6	mm/dd	09/07
7	dd	07
8	ddd	Thu
9	d	T
10	yyyy	2000
11	yy	00
12	mmmyy	Sep00
13	HH:MM:SS	15:38:09
14	HH:MM:SS PM	03:38:09 PM
15	HH:MM	15:38
16	HH:MM PM	03:38 PM
17	QQ-YY	Q3-00
18	QQ	Q3
19	dd/mm	13/03
20	dd/mm/yy	13/03/95
21	mmm.dd.yyyy HH:MM:SS	Mar.03.1962 13:53:06
22	mmm.dd.yyyy	Mar.03.1962
23	mm/dd/yyyy	03/13/1962
24	dd/mm/yyyy	12/03/1962
25	yy/mm/dd	95/03/13
26	yyyy/mm/dd	1995/03/13
27	QQ-YYYY	Q4-2132
28	mmmyyyy	Mar2047
29	yyyymmdd	20470313
30	yyyymmddTHHMMSS	20470313T132603
31	yyyy-mm-dd HH:MM:SS	1047-03-13 13:26:03

If *f* is a format string, the following symbols are recognized:

**Symbol**	**Meaning**	**Example**
yyyy	Full year	2005
yy	Two-digit year	2005
mmmm	Full month name	December
mmm	Abbreviated month name	Dec
mm	Numeric month number (padded with zeros)	01, 08, 12
m	First letter of month name (capitalized)	D
dddd	Full weekday name	Sunday
ddd	Abbreviated weekday name	Sun
dd	Numeric day of month (padded with zeros)	11
d	First letter of weekday name (capitalized)	S
HH	Hour of day, padded with zeros if PM is set	09:00
	and not padded with zeros otherwise	9:00 AM

MM	Minute of hour (padded with zeros)	10:05
SS	Second of minute (padded with zeros)	10:05:03
PM	Use 12-hour time format	11:30 PM

If *f* is not specified or is `-1`, then use 0, 1 or 16, depending on whether the date portion or the time portion of *date* is empty.

If *p* is nor specified, it defaults to the current year minus 50.

If a matrix or cell array of dates is given, a vector of date strings is returned.

See also: [datenum], page 521, [datevec], page 523, [date], page 519, [clock], page 519, [now], page 515, [datetick], page 524.

v = `datevec` (*date*) [Function File]
v = `datevec` (*date*, *f*) [Function File]
v = `datevec` (*date*, *p*) [Function File]
v = `datevec` (*date*, *f*, *p*) [Function File]
[*y*, *m*, *d*, *h*, *mi*, *s*] = `datevec` (. . .) [Function File]

Convert a serial date number (see `datenum`) or date string (see `datestr`) into a date vector.

A date vector is a row vector with six members, representing the year, month, day, hour, minute, and seconds respectively.

f is the format string used to interpret date strings (see `datestr`).

p is the year at the start of the century in which two-digit years are to be interpreted in. If not specified, it defaults to the current year minus 50.

See also: [datenum], page 521, [datestr], page 521, [date], page 519, [clock], page 519, [now], page 515.

d = `addtodate` (*d*, *q*, *f*) [Function File]

Add *q* amount of time (with units *f*) to the datenum, *d*.

f must be one of "year", "month", "day", "hour", "minute", or "second".

See also: [datenum], page 521, [datevec], page 523.

`calendar` (. . .) [Function File]
c = `calendar` () [Function File]
c = `calendar` (*d*) [Function File]
c = `calendar` (*y*, *m*) [Function File]

If called with no arguments, return the current monthly calendar in a 6x7 matrix.

If *d* is specified, return the calendar for the month containing the day *d*, which must be a serial date number or a date string.

If *y* and *m* are specified, return the calendar for year *y* and month *m*.

If no output arguments are specified, print the calendar on the screen instead of returning a matrix.

See also: [datenum], page 521.

[*n*, *s*] = `weekday` (*d*, [*form*]) [Function File]

Return the day of week as a number in *n* and a string in *s*, for example `[1, "Sun"]`, `[2, "Mon"]`, . . ., or `[7, "Sat"]`.

d is a serial date number or a date string.

If the string *form* is given and is `"long"`, *s* will contain the full name of the weekday; otherwise (or if *form* is `"short"`), *s* will contain the abbreviated name of the weekday.

See also: [datenum], page 521, [datevec], page 523, [eomday], page 524.

`e = eomday (y, m)` [Function File]

Return the last day of the month *m* for the year *y*.

See also: [datenum], page 521, [datevec], page 523, [weekday], page 523.

`datetick (form)` [Function File]
`datetick (axis, form)` [Function File]
`datetick (..., "keeplimits")` [Function File]
`datetick (..., "keepticks")` [Function File]
`datetick (...ax, ...)` [Function File]

Adds date formatted tick labels to an axis. The axis the apply the ticks to is determined by *axis* that can take the values `"x"`, `"y"` or `"z"`. The default value is `"x"`. The formatting of the labels is determined by the variable *form*, that can either be a string in the format needed by `dateform`, or a positive integer that can be accepted by `datestr`.

See also: [datenum], page 521, [datestr], page 521.

34.2 Filesystem Utilities

Octave includes the following functions for renaming and deleting files, creating, deleting, and reading directories, and for getting information about the status of files.

`[err, msg] = rename (old, new)` [Built-in Function]

Change the name of file *old* to *new*.

If successful, *err* is 0 and *msg* is an empty string. Otherwise, *err* is nonzero and *msg* contains a system-dependent error message.

See also: [ls], page 540, [dir], page 540.

`[err, msg] = link (old, new)` [Built-in Function]

Create a new link (also known as a hard link) to an existing file.

If successful, *err* is 0 and *msg* is an empty string. Otherwise, *err* is nonzero and *msg* contains a system-dependent error message.

See also: [symlink], page 524.

`[err, msg] = symlink (old, new)` [Built-in Function]

Create a symbolic link *new* which contains the string *old*.

If successful, *err* is 0 and *msg* is an empty string. Otherwise, *err* is nonzero and *msg* contains a system-dependent error message.

See also: [link], page 524, [readlink], page 525.

`[result, err, msg] = readlink (symlink)` [Built-in Function]

Read the value of the symbolic link *symlink*.

If successful, *result* contains the contents of the symbolic link *symlink*, *err* is 0 and *msg* is an empty string. Otherwise, *err* is nonzero and *msg* contains a system-dependent error message.

See also: [link], page 524, [symlink], page 524.

`[err, msg] = unlink (file)` [Built-in Function]

Delete the file named *file*.

If successful, *err* is 0 and *msg* is an empty string. Otherwise, *err* is nonzero and *msg* contains a system-dependent error message.

`[files, err, msg] = readdir (dir)` [Built-in Function]

Return names of the files in the directory *dir* as a cell array of strings. If an error occurs, return an empty cell array in *files*.

If successful, *err* is 0 and *msg* is an empty string. Otherwise, *err* is nonzero and *msg* contains a system-dependent error message.

See also: [dir], page 540, [glob], page 528.

`[status, msg, msgid] = mkdir (dir)` [Built-in Function]
`[status, msg, msgid] = mkdir (parent, dir)` [Built-in Function]

Create a directory named *dir* in the directory *parent*.

If successful, *status* is 1, with *msg* and *msgid* empty character strings. Otherwise, *status* is 0, *msg* contains a system-dependent error message, and *msgid* contains a unique message identifier.

See also: [rmdir], page 525.

`[status, msg, msgid] = rmdir (dir)` [Built-in Function]
`[status, msg, msgid] = rmdir (dir, "s")` [Built-in Function]

Remove the directory named *dir*.

If successful, *status* is 1, with *msg* and *msgid* empty character strings. Otherwise, *status* is 0, *msg* contains a system-dependent error message, and *msgid* contains a unique message identifier.

If the optional second parameter is supplied with value `"s"`, recursively remove all subdirectories as well.

See also: [mkdir], page 525, [confirm_recursive_rmdir], page 525.

`val = confirm_recursive_rmdir ()` [Built-in Function]
`old_val = confirm_recursive_rmdir (new_val)` [Built-in Function]

Query or set the internal variable that controls whether Octave will ask for confirmation before recursively removing a directory tree.

`[err, msg] = mkfifo (name, mode)` [Built-in Function]

Create a *fifo* special file named *name* with file mode *mode*

If successful, *err* is 0 and *msg* is an empty string. Otherwise, *err* is nonzero and *msg* contains a system-dependent error message.

`umask (mask)` [Built-in Function]

Set the permission mask for file creation. The parameter *mask* is an integer, interpreted as an octal number. If successful, returns the previous value of the mask (as an integer to be interpreted as an octal number); otherwise an error message is printed.

`[info, err, msg] = stat (file)` [Built-in Function]
`[info, err, msg] = lstat (file)` [Built-in Function]

Return a structure *s* containing the following information about *file*.

`dev` ID of device containing a directory entry for this file.

`ino` File number of the file.

`mode` File mode, as an integer. Use the functions `S_ISREG`, `S_ISDIR`, `S_ISCHR`, `S_ISBLK`, `S_ISFIFO`, `S_ISLNK`, or `S_ISSOCK` to extract information from this value.

`modestr` File mode, as a string of ten letters or dashes as would be returned by *ls -l*.

`nlink` Number of links.

`uid` User ID of file's owner.

`gid` Group ID of file's group.

`rdev` ID of device for block or character special files.

`size` Size in bytes.

`atime` Time of last access in the same form as time values returned from `time`. See Section 34.1 [Timing Utilities], page 515.

`mtime` Time of last modification in the same form as time values returned from `time`. See Section 34.1 [Timing Utilities], page 515.

`ctime` Time of last file status change in the same form as time values returned from `time`. See Section 34.1 [Timing Utilities], page 515.

`blksize` Size of blocks in the file.

`blocks` Number of blocks allocated for file.

If the call is successful *err* is 0 and *msg* is an empty string. If the file does not exist, or some other error occurs, *s* is an empty matrix, *err* is −1, and *msg* contains the corresponding system error message.

If *file* is a symbolic link, `stat` will return information about the actual file that is referenced by the link. Use `lstat` if you want information about the symbolic link itself.

For example,

```
[s, err, msg] = stat ("/vmlinuz")
      ⇒ s =
        {
          atime = 855399756
          rdev = 0
```

```
          ctime = 847219094
          uid = 0
          size = 389218
          blksize = 4096
          mtime = 847219094
          gid = 6
          nlink = 1
          blocks = 768
          mode = -rw-r--r--
          modestr = -rw-r--r--
          ino = 9316
          dev = 2049
        }
     ⇒ err = 0
     ⇒ msg =
```

`[`*`info`*`, `*`err`*`, `*`msg`*`] = fstat (`*`fid`*`)` [Built-in Function]

Return information about the open file *fid*. See `stat` for a description of the contents of *info*.

`[`*`status`*`, `*`msg`*`, `*`msgid`*`] = fileattrib (`*`file`*`)` [Function File]

Return information about *file*.

If successful, *status* is 1, with *result* containing a structure with the following fields:

`Name` Full name of *file*.

`archive` True if *file* is an archive (Windows).

`system` True if *file* is a system file (Windows).

`hidden` True if *file* is a hidden file (Windows).

`directory`
: True if *file* is a directory.

`UserRead`
`GroupRead`
`OtherRead`
: True if the user (group; other users) has read permission for *file*.

`UserWrite`
`GroupWrite`
`OtherWrite`
: True if the user (group; other users) has write permission for *file*.

`UserExecute`
`GroupExecute`
`OtherExecute`
: True if the user (group; other users) has execute permission for *file*.

If an attribute does not apply (i.e., archive on a Unix system) then the field is set to NaN.

With no input arguments, return information about the current directory.

If *file* contains globbing characters, return information about all the matching files.

See also: [glob], page 528.

`isdir (f)` [Function File]

Return true if *f* is a directory.

`glob (pattern)` [Built-in Function]

Given an array of strings (as a char array or a cell array) in *pattern*, return a cell array of file names that match any of them, or an empty cell array if no patterns match. Tilde expansion is performed on each of the patterns before looking for matching file names. For example,

```
glob ("/vm*")
     ⇒ "/vmlinuz"
```

See also: [dir], page 540, [ls], page 540, [stat], page 526, [readdir], page 525.

`fnmatch (pattern, string)` [Built-in Function]

Return 1 or zero for each element of *string* that matches any of the elements of the string array *pattern*, using the rules of filename pattern matching. For example,

```
fnmatch ("a*b", {"ab"; "axyzb"; "xyzab"})
     ⇒ [ 1; 1; 0 ]
```

`file_in_path (path, file)` [Built-in Function]
`file_in_path (path, file, "all")` [Built-in Function]

Return the absolute name of *file* if it can be found in *path*. The value of *path* should be a colon-separated list of directories in the format described for `path`. If no file is found, return an empty matrix. For example,

```
file_in_path (EXEC_PATH, "sh")
     ⇒ "/bin/sh"
```

If the second argument is a cell array of strings, search each directory of the path for element of the cell array and return the first that matches.

If the third optional argument `"all"` is supplied, return a cell array containing the list of all files that have the same name in the path. If no files are found, return an empty cell array.

See also: [file_in_loadpath], page 149.

`tilde_expand (string)` [Built-in Function]

Performs tilde expansion on *string*. If *string* begins with a tilde character, (‘~’), all of the characters preceding the first slash (or all characters, if there is no slash) are treated as a possible user name, and the tilde and the following characters up to the slash are replaced by the home directory of the named user. If the tilde is followed immediately by a slash, the tilde is replaced by the home directory of the user running Octave. For example,

```
tilde_expand ("~joeuser/bin")
     ⇒ "/home/joeuser/bin"
tilde_expand ("~/bin")
     ⇒ "/home/jwe/bin"
```

[*cname*, *status*, *msg*] canonicalize_file_name (*name*) [Built-in Function]

Return the canonical name of file *name*.

[*status*, *msg*, *msgid*] = movefile (*f1*, *f2*) [Function File]

Move the file *f1* to the new name *f2*. The name *f1* may contain globbing patterns. If *f1* expands to multiple file names, *f2* must be a directory.

If successful, *status* is 1, with *msg* and *msgid* empty\n\ character strings. Otherwise, *status* is 0, *msg* contains a\n\ system-dependent error message, and *msgid* contains a unique\n\ message identifier.\n\

See also: [glob], page 528.

[*status*, *msg*, *msgid*] = copyfile (*f1*, *f2*, *force*) [Function File]

Copy the file *f1* to the new name *f2*. The name *f1* may contain globbing patterns. If *f1* expands to multiple file names, *f2* must be a directory. If *force* is given and equals the string `"f"` the copy operation will be forced.

If successful, *status* is 1, with *msg* and *msgid* empty\n\ character strings. Otherwise, *status* is 0, *msg* contains a\n\ system-dependent error message, and *msgid* contains a unique\n\ message identifier.\n\

See also: [glob], page 528, [movefile], page 529.

[*dir*, *name*, *ext*, *ver*] = fileparts (*filename*) [Function File]

Return the directory, name, extension, and version components of *filename*.

See also: [fullfile], page 529.

filesep () [Built-in Function]
filesep (*'all'*) [Built-in Function]

Return the system-dependent character used to separate directory names.

If 'all' is given, the function return all valid file separators in the form of a string. The list of file separators is system-dependent. It is / (forward slash) under UNIX or Mac OS X, / and \ (forward and backward slashes) under Windows.

See also: [pathsep], page 149, [dir], page 540, [ls], page 540.

filemarker () [Built-in Function]

Returns or sets the character used to separate filename from the the subfunction names contained within the file. This can be used in a generic manner to interact with subfunctions. For example

```
help (["myfunc", filemarker, "mysubfunc"])
```

returns the help string associated with the sub-function `mysubfunc` of the function `myfunc`. Another use of `filemarker` is when debugging it allows easier placement of breakpoints within sub-functions. For example

```
dbstop (["myfunc", filemarker, "mysubfunc"])
```

will set a breakpoint at the first line of the subfunction `mysubfunc`.

***filename* = fullfile (*dir1*, *dir2*, ..., *file*)** [Function File]

Return a complete filename constructed from the given components.

See also: [fileparts], page 529.

dir = tempdir () [Function File]

Return the name of the system's directory for temporary files.

filename = tempname () [Function File]

This function is an alias for `tmpnam`.

P_tmpdir () [Built-in Function]

Return the default name of the directory for temporary files on this system. The name of this directory is system dependent.

is_absolute_filename (*file*) [Built-in Function]

Return true if *file* is an absolute filename.

is_rooted_relative_filename (*file*) [Built-in Function]

Return true if *file* is a rooted-relative filename.

make_absolute_filename (*file*) [Built-in Function]

Return the full name of *file*, relative to the current directory.

34.3 File Archiving Utilities

bunzip2 (*bzfile*, *dir*) [Function File]

Unpack the bzip2 archive *bzfile* to the directory *dir*. If *dir* is not specified, it defaults to the current directory.

See also: [unpack], page 531, [bzip2], page 531, [tar], page 530, [untar], page 531, [gzip], page 530, [gunzip], page 530, [zip], page 531, [unzip], page 531.

entries = gzip (*files*) [Function File]
entries = gzip (*files*, *outdir*) [Function File]

Compress the list of files and/or directories specified in *files*. Each file is compressed separately and a new file with a '.gz' extension is created. The original files are not touched. Existing compressed files are silently overwritten. If *outdir* is defined the compressed versions of the files are placed in this directory.

See also: [gunzip], page 530, [bzip2], page 531, [zip], page 531, [tar], page 530.

gunzip (*gzfile*, *dir*) [Function File]

Unpack the gzip archive *gzfile* to the directory *dir*. If *dir* is not specified, it defaults to the current directory. If the *gzfile* is a directory, all files in the directory will be recursively gunzipped.

See also: [unpack], page 531, [bunzip2], page 530, [tar], page 530, [untar], page 531, [gzip], page 530, [gunzip], page 530, [zip], page 531, [unzip], page 531.

entries = tar (*tarfile*, *files*, *root*) [Function File]

Pack *files files* into the TAR archive *tarfile*. The list of files must be a string or a cell array of strings.

The optional argument *root* changes the relative path of *files* from the current directory.

If an output argument is requested the entries in the archive are returned in a cell array.

See also: [untar], page 531, [gzip], page 530, [gunzip], page 530, [zip], page 531, [unzip], page 531.

`untar (`*`tarfile`*`, `*`dir`*`)` [Function File]

Unpack the TAR archive *tarfile* to the directory *dir*. If *dir* is not specified, it defaults to the current directory.

See also: [unpack], page 531, [bunzip2], page 530, [tar], page 530, [gzip], page 530, [gunzip], page 530, [zip], page 531, [unzip], page 531.

`entries`` = zip (`*`zipfile`*`, `*`files`*`)` [Function File]

`entries`` = zip (`*`zipfile`*`, `*`files`*`, `*`rootdir`*`)` [Function File]

Compress the list of files and/or directories specified in *files* into the archive *zipfiles* in the same directory. If *rootdir* is defined the *files* is located relative to *rootdir* rather than the current directory

See also: [unzip], page 531, [tar], page 530.

`unzip (`*`zipfile`*`, `*`dir`*`)` [Function File]

Unpack the ZIP archive *zipfile* to the directory *dir*. If *dir* is not specified, it defaults to the current directory.

See also: [unpack], page 531, [bunzip2], page 530, [tar], page 530, [untar], page 531, [gzip], page 530, [gunzip], page 530, [zip], page 531.

`pack ()` [Function File]

This function is provided for compatibility with MATLAB, but it doesn't actually do anything.

`files`` = unpack (`*`file`*`, `*`dir`*`)` [Function File]

`files`` = unpack (`*`file`*`, `*`dir`*`, `*`filetype`*`)` [Function File]

Unpack the archive *file* based on its extension to the directory *dir*. If *file* is a cellstr, then all files will be handled individually. If *dir* is not specified, it defaults to the current directory. It returns a list of *files* unpacked. If a directory is in the file list, then the *filetype* to unpack must also be specified.

The *files* includes the entire path to the output files.

See also: [bunzip2], page 530, [tar], page 530, [untar], page 531, [gzip], page 530, [gunzip], page 530, [zip], page 531, [unzip], page 531.

`entries`` = bzip2 (`*`files`*`)` [Function File]

`entries`` = bzip2 (`*`files`*`, `*`outdir`*`)` [Function File]

Compress the list of files specified in *files*. Each file is compressed separately and a new file with a '.bz2' extension is created. The original files are not touched. Existing compressed files are silently overwritten.If *outdir* is defined the compressed versions of the files are placed in this directory.

See also: [bunzip2], page 530, [gzip], page 530, [zip], page 531, [tar], page 530.

34.4 Networking Utilities

s = urlread (*url*) [Loadable Function]
[*s*, *success*] = urlread (*url*) [Loadable Function]
[*s*, *success*, *message*] = urlread (*url*) [Loadable Function]
[...] = urlread (*url*, *method*, *param*) [Loadable Function]

Download a remote file specified by its *URL* and return its content in string *s*. For example,

```
s = urlread ("ftp://ftp.octave.org/pub/octave/README");
```

The variable *success* is 1 if the download was successful, otherwise it is 0 in which case *message* contains an error message. If no output argument is specified and if an error occurs, then the error is signaled through Octave's error handling mechanism.

This function uses libcurl. Curl supports, among others, the HTTP, FTP and FILE protocols. Username and password may be specified in the URL. For example,

```
s = urlread ("http://user:password@example.com/file.txt");
```

GET and POST requests can be specified by *method* and *param*. The parameter *method* is either 'get' or 'post' and *param* is a cell array of parameter and value pairs. For example,

```
s = urlread ("http://www.google.com/search", "get",
             {"query", "octave"});
```

See also: [urlwrite], page 532.

urlwrite (*URL*, *localfile*) [Loadable Function]
f = urlwrite (*url*, *localfile*) [Loadable Function]
[*f*, *success*] = urlwrite (*url*, *localfile*) [Loadable Function]
[*f*, *success*, *message*] = urlwrite (*url*, *localfile*) [Loadable Function]

Download a remote file specified by its *URL* and save it as *localfile*. For example,

```
urlwrite ("ftp://ftp.octave.org/pub/octave/README",
          "README.txt");
```

The full path of the downloaded file is returned in *f*. The variable *success* is 1 if the download was successful, otherwise it is 0 in which case *message* contains an error message. If no output argument is specified and if an error occurs, then the error is signaled through Octave's error handling mechanism.

This function uses libcurl. Curl supports, among others, the HTTP, FTP and FILE protocols. Username and password may be specified in the URL, for example:

```
urlwrite ("http://username:password@example.com/file.txt",
          "file.txt");
```

GET and POST requests can be specified by *method* and *param*. The parameter *method* is either 'get' or 'post' and *param* is a cell array of parameter and value pairs. For example:

```
urlwrite ("http://www.google.com/search", "search.html",
          "get", {"query", "octave"});
```

See also: [urlread], page 532.

34.5 Controlling Subprocesses

Octave includes some high-level commands like `system` and `popen` for starting subprocesses. If you want to run another program to perform some task and then look at its output, you will probably want to use these functions.

Octave also provides several very low-level Unix-like functions which can also be used for starting subprocesses, but you should probably only use them if you can't find any way to do what you need with the higher-level functions.

`system (`*string*, *return_output*, *type*`)` [Built-in Function]

Execute a shell command specified by *string*. The second argument is optional. If *type* is `"async"`, the process is started in the background and the process id of the child process is returned immediately. Otherwise, the process is started, and Octave waits until it exits. If the *type* argument is omitted, a value of `"sync"` is assumed.

If two input arguments are given (the actual value of *return_output* is irrelevant) and the subprocess is started synchronously, or if *system* is called with one input argument and one or more output arguments, the output from the command is returned. Otherwise, if the subprocess is executed synchronously, its output is sent to the standard output. To send the output of a command executed with *system* through the pager, use a command like

```
disp (system (cmd, 1));
```

or

```
printf ("%s\n", system (cmd, 1));
```

The `system` function can return two values. The first is the exit status of the command and the second is any output from the command that was written to the standard output stream. For example,

```
[status, output] = system ("echo foo; exit 2");
```

will set the variable `output` to the string '`foo`', and the variable `status` to the integer '2'.

`[`*status*`, `*text*`] unix (`*command*`)` [Function File]
`[`*status*`, `*text*`] unix (`*command*, `"`*-echo*`")` [Function File]

Execute a system command if running under a Unix-like operating system, otherwise do nothing. Return the exit status of the program in *status* and any output sent to the standard output in *text*. If the optional second argument `"-echo"` is given, then also send the output from the command to the standard output.

See also: [isunix], page 543, [ispc], page 543, [system], page 533.

`[`*status*`, `*text*`] = dos (`*command*`)` [Function File]
`[`*status*`, `*text*`] = dos (`*command*, `"`*-echo*`")` [Function File]

Execute a system command if running under a Windows-like operating system, otherwise do nothing. Return the exit status of the program in *status* and any output sent to the standard output in *text*. If the optional second argument `"-echo"` is given, then also send the output from the command to the standard output.

See also: [unix], page 533, [isunix], page 543, [ispc], page 543, [system], page 533.

`[output, status] = perl (scriptfile)` [Function File]
`[output, status] = perl (scriptfile, argument1, argument2, ...)` [Function File]

Invoke perl script *scriptfile* with possibly a list of command line arguments. Returns output in *output* and status in *status*.

See also: [system], page 533.

`fid = popen (command, mode)` [Built-in Function]

Start a process and create a pipe. The name of the command to run is given by *command*. The file identifier corresponding to the input or output stream of the process is returned in *fid*. The argument *mode* may be

`"r"` The pipe will be connected to the standard output of the process, and open for reading.

`"w"` The pipe will be connected to the standard input of the process, and open for writing.

For example,

```
fid = popen ("ls -ltr / | tail -3", "r");
while (ischar (s = fgets (fid)))
  fputs (stdout, s);
endwhile
     ⊣ drwxr-xr-x  33 root  root  3072 Feb 15 13:28 etc
     ⊣ drwxr-xr-x   3 root  root  1024 Feb 15 13:28 lib
     ⊣ drwxrwxrwt  15 root  root  2048 Feb 17 14:53 tmp
```

`pclose (fid)` [Built-in Function]

Close a file identifier that was opened by `popen`. You may also use `fclose` for the same purpose.

`[in, out, pid] = popen2 (command, args)` [Built-in Function]

Start a subprocess with two-way communication. The name of the process is given by *command*, and *args* is an array of strings containing options for the command. The file identifiers for the input and output streams of the subprocess are returned in *in* and *out*. If execution of the command is successful, *pid* contains the process ID of the subprocess. Otherwise, *pid* is -1.

For example,

```
[in, out, pid] = popen2 ("sort", "-r");
fputs (in, "these\nare\nsome\nstrings\n");
fclose (in);
EAGAIN = errno ("EAGAIN");
done = false;
do
  s = fgets (out);
  if (ischar (s))
    fputs (stdout, s);
  elseif (errno () == EAGAIN)
```

```
          sleep (0.1);
          fclear (out);
        else
          done = true;
        endif
      until (done)
      fclose (out);
      waitpid (pid);
           ⊣ these
           ⊣ strings
           ⊣ some
           ⊣ are
```

Note that `popen2`, unlike `popen`, will not "reap" the child process. If you don't use `waitpid` to check the child's exit status, it will linger until Octave exits.

val = EXEC_PATH () [Built-in Function]
old_val = EXEC_PATH (*new_val*) [Built-in Function]

Query or set the internal variable that specifies a colon separated list of directories to search when executing external programs. Its initial value is taken from the environment variable `OCTAVE_EXEC_PATH` (if it exists) or `PATH`, but that value can be overridden by the command line argument `--exec-path PATH`. At startup, an additional set of directories (including the shell PATH) is appended to the path specified in the environment or on the command line. If you use the `EXEC_PATH` function to modify the path, you should take care to preserve these additional directories.

In most cases, the following functions simply decode their arguments and make the corresponding Unix system calls. For a complete example of how they can be used, look at the definition of the function `popen2`.

[*pid*, *msg*] = fork () [Built-in Function]

Create a copy of the current process.

Fork can return one of the following values:

> 0 — You are in the parent process. The value returned from `fork` is the process id of the child process. You should probably arrange to wait for any child processes to exit.

0 — You are in the child process. You can call `exec` to start another process. If that fails, you should probably call `exit`.

< 0 — The call to `fork` failed for some reason. You must take evasive action. A system dependent error message will be waiting in *msg*.

[*err*, *msg*] = exec (*file*, *args*) [Built-in Function]

Replace current process with a new process. Calling `exec` without first calling `fork` will terminate your current Octave process and replace it with the program named by *file*. For example,

```
exec ("ls" "-l")
```

will run `ls` and return you to your shell prompt.

If successful, `exec` does not return. If `exec` does return, *err* will be nonzero, and *msg* will contain a system-dependent error message.

`[read_fd, write_fd, err, msg] = pipe ()` [Built-in Function]

Create a pipe and return the reading and writing ends of the pipe into *read_fd* and *write_fd* respectively.

If successful, *err* is 0 and *msg* is an empty string. Otherwise, *err* is nonzero and *msg* contains a system-dependent error message.

`[fid, msg] = dup2 (old, new)` [Built-in Function]

Duplicate a file descriptor.

If successful, *fid* is greater than zero and contains the new file ID. Otherwise, *fid* is negative and *msg* contains a system-dependent error message.

`[pid, status, msg] = waitpid (pid, options)` [Built-in Function]

Wait for process *pid* to terminate. The *pid* argument can be:

−1 Wait for any child process.

0 Wait for any child process whose process group ID is equal to that of the Octave interpreter process.

> 0 Wait for termination of the child process with ID *pid*.

The *options* argument can be a bitwise OR of zero or more of the following constants:

`0` Wait until signal is received or a child process exits (this is the default if the *options* argument is missing).

`WNOHANG` Do not hang if status is not immediately available.

`WUNTRACED`
Report the status of any child processes that are stopped, and whose status has not yet been reported since they stopped.

`WCONTINUE`
Return if a stopped child has been resumed by delivery of `SIGCONT`. This value may not be meaningful on all systems.

If the returned value of *pid* is greater than 0, it is the process ID of the child process that exited. If an error occurs, *pid* will be less than zero and *msg* will contain a system-dependent error message. The value of *status* contains additional system-dependent information about the subprocess that exited.

See also: [WCONTINUE], page 536, [WCOREDUMP], page 537, [WEXITSTATUS], page 537, [WIFCONTINUED], page 537, [WIFSIGNALED], page 537, [WIFSTOPPED], page 537, [WNOHANG], page 537, [WSTOPSIG], page 538, [WTERMSIG], page 538, [WUNTRACED], page 538.

`WCONINTUE ()` [Built-in Function]

Return the numerical value of the option argument that may be passed to `waitpid` to indicate that it should also return if a stopped child has been resumed by delivery of a `SIGCONT` signal.

See also: [waitpid], page 536, [WNOHANG], page 537, [WUNTRACED], page 538.

`WCOREDUMP` (*status*) [Built-in Function]

Given *status* from a call to `waitpid`, return true if the child produced a core dump. This function should only be employed if `WIFSIGNALED` returned true. The macro used to implement this function is not specified in POSIX.1-2001 and is not available on some Unix implementations (e.g., AIX, SunOS).

See also: [waitpid], page 536, [WIFEXITED], page 537, [WEXITSTATUS], page 537, [WIFSIGNALED], page 537, [WTERMSIG], page 538, [WIFSTOPPED], page 537, [WSTOPSIG], page 538, [WIFCONTINUED], page 537.

`WEXITSTATUS` (*status*) [Built-in Function]

Given *status* from a call to `waitpid`, return the exit status of the child. This function should only be employed if `WIFEXITED` returned true.

See also: [waitpid], page 536, [WIFEXITED], page 537, [WIFSIGNALED], page 537, [WTERMSIG], page 538, [WCOREDUMP], page 537, [WIFSTOPPED], page 537, [WSTOPSIG], page 538, [WIFCONTINUED], page 537.

`WIFCONTINUED` (*status*) [Built-in Function]

Given *status* from a call to `waitpid`, return true if the child process was resumed by delivery of `SIGCONT`.

See also: [waitpid], page 536, [WIFEXITED], page 537, [WEXITSTATUS], page 537, [WIFSIGNALED], page 537, [WTERMSIG], page 538, [WCOREDUMP], page 537, [WIFSTOPPED], page 537, [WSTOPSIG], page 538.

`WIFSIGNALED` (*status*) [Built-in Function]

Given *status* from a call to `waitpid`, return true if the child process was terminated by a signal.

See also: [waitpid], page 536, [WIFEXITED], page 537, [WEXITSTATUS], page 537, [WTERMSIG], page 538, [WCOREDUMP], page 537, [WIFSTOPPED], page 537, [WSTOPSIG], page 538, [WIFCONTINUED], page 537.

`WIFSTOPPED` (*status*) [Built-in Function]

Given *status* from a call to `waitpid`, return true if the child process was stopped by delivery of a signal; this is only possible if the call was done using `WUNTRACED` or when the child is being traced (see ptrace(2)).

See also: [waitpid], page 536, [WIFEXITED], page 537, [WEXITSTATUS], page 537, [WIFSIGNALED], page 537, [WTERMSIG], page 538, [WCOREDUMP], page 537, [WSTOPSIG], page 538, [WIFCONTINUED], page 537.

`WIFEXITED` (*status*) [Built-in Function]

Given *status* from a call to `waitpid`, return true if the child terminated normally.

See also: [waitpid], page 536, [WEXITSTATUS], page 537, [WIFSIGNALED], page 537, [WTERMSIG], page 538, [WCOREDUMP], page 537, [WIFSTOPPED], page 537, [WSTOPSIG], page 538, [WIFCONTINUED], page 537.

`WNOHANG` () [Built-in Function]

Return the numerical value of the option argument that may be passed to `waitpid` to indicate that it should return its status immediately instead of waiting for a process to exit.

See also: [waitpid], page 536, [WUNTRACED], page 538, [WCONTINUE], page 536.

`WSTOPSIG (`*`status`*`)` [Built-in Function]

Given *status* from a call to `waitpid`, return the number of the signal which caused the child to stop. This function should only be employed if `WIFSTOPPED` returned true.

See also: [waitpid], page 536, [WIFEXITED], page 537, [WEXITSTATUS], page 537, [WIFSIGNALED], page 537, [WTERMSIG], page 538, [WCOREDUMP], page 537, [WIFSTOPPED], page 537, [WIFCONTINUED], page 537.

`WTERMSIG (`*`status`*`)` [Built-in Function]

Given *status* from a call to `waitpid`, return the number of the signal that caused the child process to terminate. This function should only be employed if `WIFSIGNALED` returned true.

See also: [waitpid], page 536, [WIFEXITED], page 537, [WEXITSTATUS], page 537, [WIFSIGNALED], page 537, [WCOREDUMP], page 537, [WIFSTOPPED], page 537, [WSTOPSIG], page 538, [WIFCONTINUED], page 537.

`WUNTRACED ()` [Built-in Function]

Return the numerical value of the option argument that may be passed to `waitpid` to indicate that it should also return if the child process has stopped but is not traced via the `ptrace` system call

See also: [waitpid], page 536, [WNOHANG], page 537, [WCONTINUE], page 536.

`[`*`err`*`, `*`msg`*`] = fcntl (`*`fid`*`, `*`request`*`, `*`arg`*`)` [Built-in Function]

Change the properties of the open file *fid*. The following values may be passed as *request*:

`F_DUPFD` Return a duplicate file descriptor.

`F_GETFD` Return the file descriptor flags for *fid*.

`F_SETFD` Set the file descriptor flags for *fid*.

`F_GETFL` Return the file status flags for *fid*. The following codes may be returned (some of the flags may be undefined on some systems).

- `O_RDONLY` Open for reading only.
- `O_WRONLY` Open for writing only.
- `O_RDWR` Open for reading and writing.
- `O_APPEND` Append on each write.
- `O_CREAT` Create the file if it does not exist.
- `O_NONBLOCK` Nonblocking mode.
- `O_SYNC` Wait for writes to complete.
- `O_ASYNC` Asynchronous I/O.

`F_SETFL` Set the file status flags for *fid* to the value specified by *arg*. The only flags that can be changed are `O_APPEND` and `O_NONBLOCK`.

If successful, *err* is 0 and *msg* is an empty string. Otherwise, *err* is nonzero and *msg* contains a system-dependent error message.

`[err, msg] = kill (pid, sig)` [Built-in Function]

Send signal *sig* to process *pid*.

If *pid* is positive, then signal *sig* is sent to *pid*.

If *pid* is 0, then signal *sig* is sent to every process in the process group of the current process.

If *pid* is -1, then signal *sig* is sent to every process except process 1.

If *pid* is less than -1, then signal *sig* is sent to every process in the process group *-pid*.

If *sig* is 0, then no signal is sent, but error checking is still performed.

Return 0 if successful, otherwise return -1.

`SIG ()` [Built-in Function]

Return a structure containing Unix signal names and their defined values.

34.6 Process, Group, and User IDs

`pgid = getpgrp ()` [Built-in Function]

Return the process group id of the current process.

`pid = getpid ()` [Built-in Function]

Return the process id of the current process.

`pid = getppid ()` [Built-in Function]

Return the process id of the parent process.

`euid = geteuid ()` [Built-in Function]

Return the effective user id of the current process.

`uid = getuid ()` [Built-in Function]

Return the real user id of the current process.

`egid = getegid ()` [Built-in Function]

Return the effective group id of the current process.

`gid = getgid ()` [Built-in Function]

Return the real group id of the current process.

34.7 Environment Variables

`getenv (var)` [Built-in Function]

Return the value of the environment variable *var*. For example,

```
getenv ("PATH")
```

returns a string containing the value of your path.

`putenv (var, value)` [Built-in Function]
`setenv (var, value)` [Built-in Function]

Set the value of the environment variable *var* to *value*.

34.8 Current Working Directory

`cd dir` [Command]
`chdir dir` [Command]

Change the current working directory to *dir*. If *dir* is omitted, the current directory is changed to the user's home directory. For example,

```
cd ~/octave
```

Changes the current working directory to '`~/octave`'. If the directory does not exist, an error message is printed and the working directory is not changed.

See also: [mkdir], page 525, [rmdir], page 525, [dir], page 540.

`ls options` [Command]

List directory contents. For example,

```
ls -l
     -| total 12
     -| -rw-r--r--   1 jwe  users  4488 Aug 19 04:02 foo.m
     -| -rw-r--r--   1 jwe  users  1315 Aug 17 23:14 bar.m
```

The `dir` and `ls` commands are implemented by calling your system's directory listing command, so the available options may vary from system to system.

See also: [dir], page 540, [stat], page 526, [readdir], page 525, [glob], page 528, [filesep], page 529, [ls_command], page 540.

`[old_cmd = ls_command (cmd)` [Function File]

Set or return the shell command used by Octave's `ls` command. The value of *cmd* must be a character string. With no arguments, simply return the previous value.

See also: [ls], page 540.

`dir (directory)` [Function File]
`[list] = dir (directory)` [Function File]

Display file listing for directory *directory*. If a return value is requested, return a structure array with the fields

```
name
bytes
date
isdir
statinfo
```

in which `statinfo` is the structure returned from `stat`.

If *directory* is not a directory, return information about the named *filename*. *directory* may be a list of directories specified either by name or with wildcard characters (like * and ?) which will be expanded with glob.

Note that for symbolic links, `dir` returns information about the file that a symbolic link points to instead of the link itself. However, if the link points to a nonexistent file, `dir` returns information about the link.

See also: [ls], page 540, [stat], page 526, [lstat], page 526, [readdir], page 525, [glob], page 528, [filesep], page 529.

`pwd` () [Built-in Function]

Return the current working directory.

See also: [dir], page 540, [ls], page 540.

34.9 Password Database Functions

Octave's password database functions return information in a structure with the following fields.

`name` The user name.

`passwd` The encrypted password, if available.

`uid` The numeric user id.

`gid` The numeric group id.

`gecos` The GECOS field.

`dir` The home directory.

`shell` The initial shell.

In the descriptions of the following functions, this data structure is referred to as a *pw_struct*.

pw_struct = `getpwent` () [Loadable Function]

Return a structure containing an entry from the password database, opening it if necessary. Once the end of the data has been reached, `getpwent` returns 0.

pw_struct = `getpwuid` (*uid*). [Loadable Function]

Return a structure containing the first entry from the password database with the user ID *uid*. If the user ID does not exist in the database, `getpwuid` returns 0.

pw_struct = `getpwnam` (*name*) [Loadable Function]

Return a structure containing the first entry from the password database with the user name *name*. If the user name does not exist in the database, `getpwname` returns 0.

`setpwent` () [Loadable Function]

Return the internal pointer to the beginning of the password database.

`endpwent` () [Loadable Function]

Close the password database.

34.10 Group Database Functions

Octave's group database functions return information in a structure with the following fields.

`name` The user name.

`passwd` The encrypted password, if available.

`gid` The numeric group id.

`mem` The members of the group.

In the descriptions of the following functions, this data structure is referred to as a *grp_struct*.

grp_struct = **getgrent** () [Loadable Function]
: Return an entry from the group database, opening it if necessary. Once the end of the data has been reached, `getgrent` returns 0.

grp_struct = **getgrgid** (*gid*). [Loadable Function]
: Return the first entry from the group database with the group ID *gid*. If the group ID does not exist in the database, `getgrgid` returns 0.

grp_struct = **getgrnam** (*name*) [Loadable Function]
: Return the first entry from the group database with the group name *name*. If the group name does not exist in the database, `getgrnam` returns 0.

setgrent () [Loadable Function]
: Return the internal pointer to the beginning of the group database.

endgrent () [Loadable Function]
: Close the group database.

34.11 System Information

[*c*, *maxsize*, *endian*] = **computer** () [Function File]
: Print or return a string of the form *cpu-vendor-os* that identifies the kind of computer Octave is running on. If invoked with an output argument, the value is returned instead of printed. For example,

```
computer ()
     ⊣ i586-pc-linux-gnu

x = computer ()
     ⇒ x = "i586-pc-linux-gnu"
```

If two output arguments are requested, also return the maximum number of elements for an array.

If three output arguments are requested, also return the byte order of the current system as a character (`"B"` for big-endian or `"L"` for little-endian).

`[uts, err, msg] = uname ()` [Built-in Function]

Return system information in the structure. For example,

```
uname ()
     ⇒ {
           sysname = x86_64
           nodename = segfault
           release = 2.6.15-1-amd64-k8-smp
           version = Linux
           machine = #2 SMP Thu Feb 23 04:57:49 UTC 2006
         }
```

If successful, *err* is 0 and *msg* is an empty string. Otherwise, *err* is nonzero and *msg* contains a system-dependent error message.

`ispc ()` [Function File]

Return 1 if Octave is running on a Windows system and 0 otherwise.

See also: [ismac], page 543, [isunix], page 543.

`isunix ()` [Function File]

Return 1 if Octave is running on a Unix-like system and 0 otherwise.

See also: [ismac], page 543, [ispc], page 543.

`ismac ()` [Function File]

Return 1 if Octave is running on a Mac OS X system and 0 otherwise.

See also: [ispc], page 543, [isunix], page 543.

`isieee ()` [Built-in Function]

Return 1 if your computer claims to conform to the IEEE standard for floating point calculations.

`OCTAVE_HOME ()` [Built-in Function]

Return the name of the top-level Octave installation directory.

`OCTAVE_VERSION ()` [Built-in Function]

Return the version number of Octave, as a string.

`license` [Function File]

Display the license of Octave.

`license ("inuse")` [Function File]

Display a list of packages currently being used.

`retval = license ("inuse")` [Function File]

Return a structure containing the fields `feature` and `user`.

`retval = license ("test", feature)` [Function File]

Return 1 if a license exists for the product identified by the string *feature* and 0 otherwise. The argument *feature* is case insensitive and only the first 27 characters are checked.

`license ("test", feature, toggle)` [Function File]

Enable or disable license testing for *feature*, depending on *toggle*, which may be one of:

‘"enable"’
: Future tests for the specified license of *feature* are conducted as usual.

‘"disable"’
: Future tests for the specified license of *feature* return 0.

retval = license ("*checkout*", *feature*) [Function File]

Check out a license for *feature*, returning 1 on success and 0 on failure.

This function is provided for compatibility with MATLAB.

See also: [ver], page 544, [version], page 544.

version () [Function File]

Return Octave's version number as a string. This is also the value of the built-in variable `OCTAVE_VERSION`.

ver () [Function File]

Display a header containing the current Octave version number, license string and operating system, followed by the installed package names, versions, and installation directories. v = ver ()

Return a vector of structures, respecting Octave and each installed package. The structure includes the following fields.

`Name` Package name.

`Version` Version of the package.

`Revision` Revision of the package.

`Date` Date respecting the version/revision.

v = ver ("Octave") [Function File]

Return version information for Octave only.. v = ver (*pkg*)

Return version information for the specified package *pkg*.

See also: [license], page 543, [version], page 544.

octave_config_info (*option*) [Built-in Function]

Return a structure containing configuration and installation information for Octave.

if *option* is a string, return the configuration information for the specified option.

getrusage () [Loadable Function]

Return a structure containing a number of statistics about the current Octave process. Not all fields are available on all systems. If it is not possible to get CPU time statistics, the CPU time slots are set to zero. Other missing data are replaced by NaN. Here is a list of all the possible fields that can be present in the structure returned by `getrusage`:

`idrss` Unshared data size.

`inblock` Number of block input operations.

`isrss` Unshared stack size.

`ixrss` Shared memory size.

`majflt` Number of major page faults.

`maxrss` Maximum data size.

`minflt` Number of minor page faults.

`msgrcv` Number of messages received.

`msgsnd` Number of messages sent.

`nivcsw` Number of involuntary context switches.

`nsignals` Number of signals received.

`nswap` Number of swaps.

`nvcsw` Number of voluntary context switches.

`oublock` Number of block output operations.

`stime` A structure containing the system CPU time used. The structure has the elements `sec` (seconds) `usec` (microseconds).

`utime` A structure containing the user CPU time used. The structure has the elements `sec` (seconds) `usec` (microseconds).

34.12 Hashing Functions

It is often necessary to find if two strings or files are identical. This might be done by comparing them character by character and looking for differences. However, this can be slow, and so comparing a hash of the string or file can be a rapid way of finding if the files differ.

Another use of the hashing function is to check for file integrity. The user can check the hash of the file against a known value and find if the file they have is the same as the one that the original hash was produced with.

Octave supplies the `md5sum` function to perform MD5 hashes on strings and files. An example of the use of `md5sum` function might be

```
if exist (file, "file")
  hash = md5sum (file);
else
  # Treat the variable "file" as a string
  hash = md5sum (file, true);
endif
```

`md5sum (`*`file`*`)` [Loadable Function]
`md5sum (`*`str`*, *`opt`*`)` [Loadable Function]

Calculates the MD5 sum of the file *file*. If the second parameter *opt* exists and is true, then calculate the MD5 sum of the string *str*.

35 Packages

Since Octave is Free Software users are encouraged to share their programs amongst each other. To aid this sharing Octave supports the installation of extra packages. The 'Octave-Forge' project is a community-maintained set of packages that can be downloaded and installed in Octave. At the time of writing the 'Octave-Forge' project can be found on-line at http://octave.sourceforge.net, but since the Internet is an ever-changing place this may not be true at the time of reading. Therefore it is recommended to see the Octave website for an updated reference.

35.1 Installing and Removing Packages

Assuming a package is available in the file 'image-1.0.0.tar.gz' it can be installed from the Octave prompt with the command

```
pkg install image-1.0.0.tar.gz
```

If the package is installed successfully nothing will be printed on the prompt, but if an error occurred during installation it will be reported. It is possible to install several packages at once by writing several package files after the `pkg install` command. If a different version of the package is already installed it will be removed prior to installing the new package. This makes it easy to upgrade and downgrade the version of a package, but makes it impossible to have several versions of the same package installed at once.

To see which packages are installed type

```
pkg list
 ⊣ Package Name  | Version | Installation directory
 ⊣ --------------+---------+-----------------------
 ⊣        image *|   1.0.0 | /home/jwe/octave/image-1.0.0
```

In this case only version 1.0.0 of the `image` package is installed. The '*' character next to the package name shows that the image package is loaded and ready for use.

It is possible to remove a package from the system using the `pkg uninstall` command like this

```
pkg uninstall image
```

If the package is removed successfully nothing will be printed in the prompt, but if an error occurred it will be reported. It should be noted that the package file used for installation is not needed for removal, and that only the package name as reported by `pkg list` should be used when removing a package. It is possible to remove several packages at once by writing several package names after the `pkg uninstall` command.

To minimize the amount of code duplication between packages it is possible that one package depends on another one. If a package depends on another, it will check if that package is installed during installation. If it is not, an error will be reported and the package will not be installed. This behavior can be disabled by passing the `-nodeps` flag to the `pkg install` command

```
pkg install -nodeps my_package_with_dependencies.tar.gz
```

Since the installed package expects its dependencies to be installed it may not function correctly. Because of this it is not recommended to disable dependency checking.

pkg *command pkg_name* [Command]
pkg *command option pkg_name* [Command]

This command interacts with the package manager. Different actions will be taken depending on the value of *command*.

'`install`' Install named packages. For example,

```
pkg install image-1.0.0.tar.gz
```

installs the package found in the file '`image-1.0.0.tar.gz`'.

The *option* variable can contain options that affect the manner in which a package is installed. These options can be one or more of

`-nodeps` The package manager will disable the dependency checking. That way it is possible to install a package even if it depends on another package that's not installed on the system. **Use this option with care.**

`-noauto` The package manager will not automatically load the installed package when starting Octave, even if the package requests that it is.

`-auto` The package manager will automatically load the installed package when starting Octave, even if the package requests that it isn't.

`-local` A local installation is forced, even if the user has system privileges.

`-global` A global installation is forced, even if the user doesn't normally have system privileges

`-verbose` The package manager will print the output of all of the commands that are performed.

'`uninstall`'

Uninstall named packages. For example,

```
pkg uninstall image
```

removes the `image` package from the system. If another installed package depends on the `image` package an error will be issued. The package can be uninstalled anyway by using the `-nodeps` option.

'`load`' Add named packages to the path. After loading a package it is possible to use the functions provided by the package. For example,

```
pkg load image
```

adds the `image` package to the path. It is possible to load all installed packages at once with the command

```
pkg load all
```

'`unload`' Removes named packages from the path. After unloading a package it is no longer possible to use the functions provided by the package. This command behaves like the `load` command.

'list' Show a list of the currently installed packages. By requesting one or two output argument it is possible to get a list of the currently installed packages. For example,

```
installed_packages = pkg list;
```

returns a cell array containing a structure for each installed package. The command

```
[user_packages, system_packages] = pkg list
```

splits the list of installed packages into those who are installed by the current user, and those installed by the system administrator.

'describe'

Show a short description of the named installed packages, with the option '-verbose' also list functions provided by the package, e.g.:

```
pkg describe -verbose all
```

will describe all installed packages and the functions they provide. If one output is requested a cell of structure containing the description and list of functions of each package is returned as output rather than printed on screen:

```
desc = pkg ("describe", "secs1d", "image")
```

If any of the requested packages is not installed, pkg returns an error, unless a second output is requested:

```
[ desc, flag] = pkg ("describe", "secs1d", "image")
```

flag will take one of the values "Not installed", "Loaded" or "Not loaded" for each of the named packages.

'prefix' Set the installation prefix directory. For example,

```
pkg prefix ~/my_octave_packages
```

sets the installation prefix to '~/my_octave_packages'. Packages will be installed in this directory.

It is possible to get the current installation prefix by requesting an output argument. For example,

```
p = pkg prefix
```

The location in which to install the architecture dependent files can be independent specified with an addition argument. For example

```
pkg prefix ~/my_octave_packages ~/my_arch_dep_pkgs
```

'local_list'

Set the file in which to look for information on the locally installed packages. Locally installed packages are those that are typically available only to the current user. For example

```
pkg local_list ~/.octave_packages
```

It is possible to get the current value of local_list with the following

```
pkg local_list
```

'global_list'
: Set the file in which to look for, for information on the globally installed packages. Globally installed packages are those that are typically available to all users. For example

```
pkg global_list /usr/share/octave/octave_packages
```

It is possible to get the current value of global_list with the following

```
pkg global_list
```

'rebuild'
: Rebuilds the package database from the installed directories. This can be used in cases where for some reason the package database is corrupted. It can also take the `-auto` and `-noauto` options to allow the autoloading state of a package to be changed. For example

```
pkg rebuild -noauto image
```

will remove the autoloading status of the image package.

'build'
: Builds a binary form of a package or packages. The binary file produced will itself be an Octave package that can be installed normally with `pkg`. The form of the command to build a binary package is

```
pkg build builddir image-1.0.0.tar.gz ...
```

where `builddir` is the name of a directory where the temporary installation will be produced and the binary packages will be found. The options `-verbose` and `-nodeps` are respected, while the other options are ignored.

35.2 Using Packages

By default installed packages are available from the Octave prompt, but it is possible to control this using the `pkg load` and `pkg unload` commands. The functions from a package can be removed from the Octave path by typing

```
pkg unload package_name
```

where `package_name` is the name of the package to be removed from the path.

In much the same way a package can be added to the Octave path by typing

```
pkg load package_name
```

35.3 Administrating Packages

On UNIX-like systems it is possible to make both per-user and system-wide installations of a package. If the user performing the installation is `root` the packages will be installed in a system-wide directory that defaults to '`OCTAVE_HOME/share/octave/packages/`'. If the user is not `root` the default installation directory is '`~/octave/`'. Packages will be installed in a subdirectory of the installation directory that will be named after the package. It is possible to change the installation directory by using the `pkg prefix` command

```
pkg prefix new_installation_directory
```

The current installation directory can be retrieved by typing

```
current_installation_directory = pkg prefix
```

To function properly the package manager needs to keep some information about the installed packages. For per-user packages this information is by default stored

in the file '~/.octave_packages' and for system-wide installations it is stored in 'OCTAVE_HOME/share/octave/octave_packages'. The path to the per-user file can be changed with the pkg local_list command

```
pkg local_list /path/to/new_file
```

For system-wide installations this can be changed in the same way using the pkg global_list command. If these commands are called without a new path, the current path will be returned.

35.4 Creating Packages

Internally a package is simply a gzipped tar file that contains a top level directory of any given name. This directory will in the following be referred to as package and may contain the following files

package/DESCRIPTION
: This is a required file containing information about the package. See Section 35.4.1 [The DESCRIPTION File], page 552, for details on this file.

package/COPYING
: This is a required file containing the license of the package. No restrictions is made on the license in general. If however the package contains dynamically linked functions the license must be compatible with the GNU General Public License.

package/INDEX
: This is an optional file describing the functions provided by the package. If this file is not given then one with be created automatically from the functions in the package and the Categories keyword in the DESCRIPTION file. See Section 35.4.2 [The INDEX file], page 554, for details on this file.

package/PKG_ADD
: An optional file that includes commands that are run when the package is added to the users path. Note that PKG_ADD directives in the source code of the package will also be added to this file by the Octave package manager. Note that symbolic links are to be avoided in packages, as symbolic links do not exist on some file systems, and so a typical use for this file is the replacement of the symbolic link

```
ln -s foo.oct bar.oct
```

with an autoload directive like

```
autoload ('bar', which ('foo'));
```

See Section 35.4.3 [PKG_ADD and PKG_DEL directives], page 555, for details on PKG_ADD directives.

package/PKG_DEL
: An optional file that includes commands that are run when the package is removed from the users path. Note that PKG_DEL directives in the source code of the package will also be added to this file by the Octave package manager. See Section 35.4.3 [PKG_ADD and PKG_DEL directives], page 555, for details on PKG_DEL directives.

`package/pre_install.m`
: This is an optional script that is run prior to the installation of a package.

`package/post_install.m`
: This is an optional script that is run after the installation of a package.

`package/on_uninstall.m`
: This is an optional script that is run prior to the removal of a package.

Besides the above mentioned files, a package can also contain on or more of the following directories

`package/inst`
: An optional directory containing any files that are directly installed by the package. Typically this will include any `m`-files.

`package/src`
: An optional directory containing code that must be built prior to the packages installation. The Octave package manager will execute '`./configure`' in this directory if this script exists, and will then call `make` if a file '`Makefile`' exists in this directory. `make install` will however not be called. If a file called `FILES` exists all files listed there will be copied to the `inst` directory, so they also will be installed. If the `FILES` file doesn't exist, '`src/*.m`' and '`src/*.oct`' will be copied to the `inst` directory.

`package/doc`
: An optional directory containing documentation for the package. The files in this directory will be directly installed in a sub-directory of the installed package for future reference.

`package/bin`
: An optional directory containing files that will be added to the Octave `EXEC_PATH` when the package is loaded. This might contain external scripts, etc., called by functions within the package.

35.4.1 The DESCRIPTION File

The `DESCRIPTION` file contains various information about the package, such as its name, author, and version. This file has a very simple format

- Lines starting with '`#`' are comments.
- Lines starting with a blank character are continuations from the previous line.
- Everything else is of the form `NameOfOption: ValueOfOption`.

The following is a simple example of a `DESCRIPTION` file

```
Name: The name of my package
Version: 1.0.0
Date: 2007-18-04
Author: The name (and possibly email) of the package author.
Maintainer: The name (and possibly email) of the current
 package maintainer.
Title: The title of the package
Description: A short description of the package.  If this
 description gets too long for one line it can continue
 on the next by adding a space to the beginning of the
 following lines.
License: GPL version 3 or later
```

The package manager currently recognizes the following keywords

`Name` Name of the package.

`Version` Version of the package.

`Date` Date of last update.

`Author` Original author of the package.

`Maintainer`
Maintainer of the package.

`Title` A one line description of the package.

`Description`
A one paragraph description of the package.

`Categories`
Optional keyword describing the package (if no `INDEX` file is given this is mandatory).

`Problems` Optional list of known problems.

`Url` Optional list of homepages related to the package.

`Autoload` Optional field that sets the default loading behavior for the package. If set to `yes`, `true` or `on`, then Octave will automatically load the package when starting. Otherwise the package must be manually loaded with the pkg load command. This default behavior can be overridden when the package is installed.

`Depends` A list of other Octave packages that this package depends on. This can include dependencies on particular versions, with a format

```
Depends: package (>= 1.0.0)
```

Possible operators are `<`, `<=`, `==`, `>=` or `>`. If the part of the dependency in `()` is missing, any version of the package is acceptable. Multiple dependencies can be defined either as a comma separated list or on separate `Depends` lines.

`License` An optional short description of the used license (e.g., GPL version 3 or newer). This is optional since the file `COPYING` is mandatory.

`SystemRequirements`
: These are the external install dependencies of the package and are not checked by the package manager. This is here as a hint to the distribution packager. They follow the same conventions as the `Depends` keyword.

`BuildRequires`
: These are the external build dependencies of the package and are not checked by the package manager. This is here as a hint to the distribution packager. They follow the same conventions as the `Depends` keyword. Note that in general, packaging systems such as `rpm` or `deb` and autoprobe the install dependencies from the build dependencies, and therefore the often a `BuildRequires` dependency removes the need for a `SystemRequirements` dependency.

The developer is free to add additional arguments to the `DESCRIPTION` file for their own purposes. One further detail to aid the packager is that the `SystemRequirements` and `BuildRequires` keywords can have a distribution dependent section, and the automatic build process will use these. An example of the format of this is

```
BuildRequires: libtermcap-devel [Mandriva] libtermcap2-devel
```

where the first package name will be used as a default and if the RPMs are built on a Mandriva distribution, then the second package name will be used instead.

35.4.2 The INDEX file

The optional `INDEX` file provides a categorical view of the functions in the package. This file has a very simple format

- Lines beginning with '`#`' are comments.
- The first non-comment line should look like this

  ```
  toolbox >> Toolbox name
  ```
- Lines beginning with an alphabetical character indicates a new category of functions.
- Lines starting with a white space character indicate that the function names on the line belong to the last mentioned category.

The format can be summarized with the following example

```
# A comment
toolbox >> Toolbox name
Category Name 1
 function1 function2 function3
 function4
Category Name 2
 function2 function5
```

If you wish to refer to a function that users might expect to find in your package but is not there, providing a work around or pointing out that the function is available elsewhere, you can use:

```
fn = workaround description
```

This workaround description will not appear when listing functions in the package with `pkg describe` but they will be published in the html documentation online. Workaround descriptions can use any html markup, but keep in mind that it will be enclosed in a bold-italic environment. For the special case of:

```
fn = use <code>alternate expression</code>
```

the bold-italic is automatically suppressed. You will need to use <code> even in references:

```
fn = use <a href="someothersite.html"><code>fn</code></a>
```

Sometimes functions are only partially compatible, in which case you can list the non-compatible cases separately. To refer to another function in the package, use <f>fn</f>. For example,

```
eig (a, b) = use <f>qz</f>
```

Since sites may have many missing functions, you can define a macro rather than typing the same link over and again.

```
$id = expansion
```

defines the macro id. You can use $id anywhere in the description and it will be expanded. For example,

```
$TSA = see <a href="link_to_spctools">SPC Tools</a>
arcov = $TSA <code>armcv</code>
```

id is any string of letters, numbers and _.

35.4.3 PKG_ADD and PKG_DEL directives

If the package contains files called PKG_ADD or PKG_DEL the commands in these files will be executed when the package is added or removed from the users path. In some situations such files are a bit cumbersome to maintain, so the package manager supports automatic creation of such files. If a source file in the package contains a PKG_ADD or PKG_DEL directive they will be added to either the PKG_ADD or PKG_DEL files.

In m-files a PKG_ADD directive looks like this

```
## PKG_ADD: some_octave_command
```

Such lines should be added before the function keyword. In C++ files a PKG_ADD directive looks like this

```
// PKG_ADD: some_octave_command
```

In both cases some_octave_command should be replaced by the command that should be placed in the PKG_ADD file. PKG_DEL directives work in the same way, except the PKG_ADD keyword is replaced with PKG_DEL and the commands get added to the PKG_DEL file.

Appendix A Dynamically Linked Functions

Octave has the possibility of including compiled code as dynamically linked extensions and then using these extensions as if they were part of Octave itself. Octave can call C++ code through its native oct-file interface or C code through its mex interface. It can also indirectly call functions written in any other language through a simple wrapper. The reasons to write code in a compiled language might be either to link to an existing piece of code and allow it to be used within Octave, or to allow improved performance for key pieces of code.

Before going further, you should first determine if you really need to use dynamically linked functions at all. Before proceeding with writing any dynamically linked function to improve performance you should address ask yourself

- Can I get the same functionality using the Octave scripting language only?
- Is it thoroughly optimized Octave code? Vectorization of Octave code, doesn't just make it concise, it generally significantly improves its performance. Above all, if loops must be used, make sure that the allocation of space for variables takes place outside the loops using an assignment to a matrix of the right size, or zeros.
- Does it make as much use as possible of existing built-in library routines? These are highly optimized and many do not carry the overhead of being interpreted.
- Does writing a dynamically linked function represent useful investment of your time, relative to staying in Octave?

Also, as oct- and mex-files are dynamically linked to Octave, they introduce the possibility of Octave crashing due to errors in the user code. For example a segmentation violation in the user's code will cause Octave to abort.

A.1 Oct-Files

A.1.1 Getting Started with Oct-Files

The basic command to build oct-files is `mkoctfile` and it can be call from within octave or from the command line.

`mkoctfile` [*-options*] *file* . . . [Function File]

The `mkoctfile` function compiles source code written in C, C++, or Fortran. Depending on the options used with `mkoctfile`, the compiled code can be called within Octave or can be used as a stand-alone application.

`mkoctfile` can be called from the shell prompt or from the Octave prompt.

`mkoctfile` accepts the following options, all of which are optional except for the file name of the code you wish to compile:

'`-I DIR`' Add the include directory DIR to compile commands.

'`-D DEF`' Add the definition DEF to the compiler call.

'`-l LIB`' Add the library LIB to the link command.

'`-L DIR`' Add the library directory DIR to the link command.

'-M'
'--depend'
: Generate dependency files (.d) for C and C++ source files.

'-c' Compile but do not link.

'-g' Enable debugging options for compilers.

'-o FILE'
'--output FILE'
: Output file name. Default extension is .oct (or .mex if –mex is specified) unless linking a stand-alone executable.

'-p VAR'
'--print VAR'
: Print the configuration variable VAR. Recognized variables are:

```
ALL_CFLAGS                  FFTW_LIBS
ALL_CXXFLAGS                FLIBS
ALL_FFLAGS                  FPICFLAG
ALL_LDFLAGS                 INCFLAGS
BLAS_LIBS                   LDFLAGS
CC                          LD_CXX
CFLAGS                      LD_STATIC_FLAG
CPICFLAG                    LFLAGS
CPPFLAGS                    LIBCRUFT
CXX                         LIBOCTAVE
CXXFLAGS                    LIBOCTINTERP
CXXPICFLAG                  LIBREADLINE
DEPEND_EXTRA_SED_PATTERN    LIBS
DEPEND_FLAGS                OCTAVE_LIBS
DL_LD                       RDYNAMIC_FLAG
DL_LDFLAGS                  RLD_FLAG
F2C                         SED
F2CFLAGS                    XTRA_CFLAGS
F77                         XTRA_CXXFLAGS
FFLAGS
```

'--link-stand-alone'
: Link a stand-alone executable file.

'--mex' Assume we are creating a MEX file. Set the default output extension to ".mex".

'-s'
'--strip' Strip the output file.

'-v'
'--verbose'
: Echo commands as they are executed.

'file' The file to compile or link. Recognized file types are

.c	C source
.cc	C++ source
.C	C++ source
.cpp	C++ source
.f	Fortran source
.F	Fortran source
.o	object file

Consider the short example

```
#include <octave/oct.h>

DEFUN_DLD (helloworld, args, nargout,
  "Hello World Help String")
{
  int nargin = args.length ();
  octave_stdout << "Hello World has " << nargin
        << " input arguments and "
        << nargout << " output arguments.\n";
  return octave_value_list ();
}
```

This example although short introduces the basics of writing a C++ function that can be dynamically linked to Octave. The easiest way to make available most of the definitions that might be necessary for an oct-file in Octave is to use the #include <octave/oct.h> header.

The macro that defines the entry point into the dynamically loaded function is DEFUN_DLD. This macro takes four arguments, these being

1. The function name as it will be seen in Octave,
2. The list of arguments to the function of type octave_value_list,
3. The number of output arguments, which can and often is omitted if not used, and
4. The string that will be seen as the help text of the function.

The return type of functions defined with DEFUN_DLD is always octave_value_list.

There are a couple of important considerations in the choice of function name. Firstly, it must be a valid Octave function name and so must be a sequence of letters, digits and underscores, not starting with a digit. Secondly, as Octave uses the function name to define the filename it attempts to find the function in, the function name in the DEFUN_DLD macro must match the filename of the oct-file. Therefore, the above function should be in a file 'helloworld.cc', and it should be compiled to an oct-file using the command

```
mkoctfile helloworld.cc
```

This will create a file called 'helloworld.oct', that is the compiled version of the function. It should be noted that it is perfectly acceptable to have more than one DEFUN_DLD function in a source file. However, there must either be a symbolic link to the oct-file for each of the functions defined in the source code with the DEFUN_DLD macro or the autoload (Section 11.7 [Function Files], page 145) function should be used.

The rest of this function then shows how to find the number of input arguments, how to print through the octave pager, and return from the function. After compiling this function as above, an example of its use is

```
helloworld (1, 2, 3)
 ⊣ Hello World has 3 input arguments and 0 output arguments.
```

A.1.2 Matrices and Arrays in Oct-Files

Octave supports a number of different array and matrix classes, the majority of which are based on the Array class. The exception is the sparse matrix types discussed separately below. There are three basic matrix types

`Matrix` A double precision matrix class defined in dMatrix.h,

`ComplexMatrix`
: A complex matrix class defined in CMatrix.h, and

`BoolMatrix`
: A boolean matrix class defined in boolMatrix.h.

These are the basic two-dimensional matrix types of octave. In additional there are a number of multi-dimensional array types, these being

`NDArray` A double precision array class defined in '`dNDArray.h`'

`ComplexNDarray`
: A complex array class defined in '`CNDArray.h`'

`boolNDArray`
: A boolean array class defined in '`boolNDArray.h`'

`int8NDArray`
`int16NDArray`
`int32NDArray`
`int64NDArray`
: 8, 16, 32 and 64-bit signed array classes defined in '`int8NDArray.h`', '`int16NDArray.h`', etc.

`uint8NDArray`
`uint16NDArray`
`uint32NDArray`
`uint64NDArray`
: 8, 16, 32 and 64-bit unsigned array classes defined in '`uint8NDArray.h`', '`uint16NDArray.h`', etc.

There are several basic means of constructing matrices of multi-dimensional arrays. Considering the `Matrix` type as an example

- We can create an empty matrix or array with the empty constructor. For example

  ```
  Matrix a;
  ```

  This can be used on all matrix and array types
- Define the dimensions of the matrix or array with a dim_vector. For example

```
dim_vector dv (2);
dv(0) = 2; dv(1) = 2;
Matrix a (dv);
```

This can be used on all matrix and array types

- Define the number of rows and columns in the matrix. For example

```
Matrix a (2, 2)
```

However, this constructor can only be used with the matrix types.

These types all share a number of basic methods and operators, a selection of which include

`T& operator () (`*octave_idx_type*`)` [Method]
`T& elem (`*octave_idx_type*`)` [Method]

The `()` operator or `elem` method allow the values of the matrix or array to be read or set. These can take a single argument, which is of type `octave_idx_type`, that is the index into the matrix or array. Additionally, the matrix type allows two argument versions of the `()` operator and elem method, giving the row and column index of the value to obtain or set.

Note that these functions do significant error checking and so in some circumstances the user might prefer to access the data of the array or matrix directly through the fortran_vec method discussed below.

`octave_idx_type nelem (`*void*`)` *const* [Method]

The total number of elements in the matrix or array.

`size_t byte_size (`*void*`)` *const* [Method]

The number of bytes used to store the matrix or array.

`dim_vector dims (`*void*`)` *const* [Method]

The dimensions of the matrix or array in value of type dim_vector.

`void resize (`*const dim_vector&*`)` [Method]

A method taking either an argument of type `dim_vector`, or in the case of a matrix two arguments of type `octave_idx_type` defining the number of rows and columns in the matrix.

`T* fortran_vec (`*void*`)` [Method]

This method returns a pointer to the underlying data of the matrix or a array so that it can be manipulated directly, either within Octave or by an external library.

Operators such an `+`, `-`, or `*` can be used on the majority of the above types. In addition there are a number of methods that are of interest only for matrices such as `transpose`, `hermitian`, `solve`, etc.

The typical way to extract a matrix or array from the input arguments of `DEFUN_DLD` function is as follows

```
#include <octave/oct.h>

DEFUN_DLD (addtwomatrices, args, , "Add A to B")
{
  int nargin = args.length ();
  if (nargin != 2)
    print_usage ();
  else
    {
      NDArray A = args(0).array_value ();
      NDArray B = args(1).array_value ();
      if (!  error_state)
        return octave_value (A + B);
    }
  return octave_value_list ();
}
```

To avoid segmentation faults causing Octave to abort, this function explicitly checks that there are sufficient arguments available before accessing these arguments. It then obtains two multi-dimensional arrays of type `NDArray` and adds these together. Note that the array_value method is called without using the `is_matrix_type` type, and instead the error_state is checked before returning `A + B`. The reason to prefer this is that the arguments might be a type that is not an `NDArray`, but it would make sense to convert it to one. The `array_value` method allows this conversion to be performed transparently if possible, and sets `error_state` if it is not.

`A + B`, operating on two `NDArray`'s returns an `NDArray`, which is cast to an `octave_value` on the return from the function. An example of the use of this demonstration function is

```
addtwomatrices (ones (2, 2), ones (2, 2))
      ⇒  2  2
         2  2
```

A list of the basic `Matrix` and `Array` types, the methods to extract these from an `octave_value` and the associated header is listed below.

RowVector	row_vector_value	‘dRowVector.h’
ComplexRowVector	complex_row_vector_value	‘CRowVector.h’
ColumnVector	column_vector_value	‘dColVector.h’
ComplexColumnVector	complex_column_vector_value	‘CColVector.h’
Matrix	matrix_value	‘dMatrix.h’
ComplexMatrix	complex_matrix_value	‘CMatrix.h’
boolMatrix	bool_matrix_value	‘boolMatrix.h’
charMatrix	char_matrix_value	‘chMatrix.h’
NDArray	array_value	‘dNDArray.h’
ComplexNDArray	complex_array_value	‘CNDArray.h’
boolNDArray	bool_array_value	‘boolNDArray.h’
charNDArray	char_array_value	‘charNDArray.h’
int8NDArray	int8_array_value	‘int8NDArray.h’
int16NDArray	int16_array_value	‘int16NDArray.h’
int32NDArray	int32_array_value	‘int32NDArray.h’

int64NDArray	int64_array_value	'int64NDArray.h'
uint8NDArray	uint8_array_value	'uint8NDArray.h'
uint16NDArray	uint16_array_value	'uint16NDArray.h'
uint32NDArray	uint32_array_value	'uint32NDArray.h'
uint64NDArray	uint64_array_value	'uint64NDArray.h'

A.1.3 Character Strings in Oct-Files

In Octave a character string is just a special `Array` class. Consider the example

```
#include <octave/oct.h>

DEFUN_DLD (stringdemo, args, , "String Demo")
{
  int nargin = args.length();
  octave_value_list retval;

  if (nargin != 1)
    print_usage ();
  else
    {
      charMatrix ch = args(0).char_matrix_value ();

      if (!  error_state)
        {
          if (args(0).is_sq_string ())
            retval(1) = octave_value (ch, true);
          else
            retval(1) = octave_value (ch, true, '\'');

          octave_idx_type nr = ch.rows();
          for (octave_idx_type i = 0; i < nr / 2; i++)
            {
              std::string tmp = ch.row_as_string (i);
              ch.insert (ch.row_as_string(nr-i-1).c_str(),
                         i, 0);
              ch.insert (tmp.c_str(), nr-i-1, 0);
            }
          retval(0) = octave_value (ch, true);
        }
    }
  return retval;
}
```

An example of the use of this function is

```
s0 = ["First String"; "Second String"];
[s1,s2] = stringdemo (s0)
⇒ s1 = Second String
       First String

⇒ s2 = First String
       Second String

typeinfo (s2)
⇒ sq_string
typeinfo (s1)
⇒ string
```

One additional complication of strings in Octave is the difference between single quoted and double quoted strings. To find out if an `octave_value` contains a single or double quoted string an example is

```
    if (args(0).is_sq_string ())
      octave_stdout <<
        "First argument is a singularly quoted string\n";
    else if (args(0).is_dq_string ())
      octave_stdout <<
        "First argument is a doubly quoted string\n";
```

Note however, that both types of strings are represented by the `charNDArray` type, and so when assigning to an `octave_value`, the type of string should be specified. For example

```
octave_value_list retval;
charNDArray c;
...
// Create single quoted string
retval(1) = octave_value (ch, true, '\'');

// Create a double quoted string
retval(0) = octave_value (ch, true);
```

A.1.4 Cell Arrays in Oct-Files

Octave's cell type is equally accessible within oct-files. A cell array is just an array of `octave_values`, and so each element of the cell array can then be treated just like any other `octave_value`. A simple example is

```
#include <octave/oct.h>
#include <octave/Cell.h>

DEFUN_DLD (celldemo, args, , "Cell Demo")
{
  octave_value_list retval;
  int nargin = args.length ();

  if (nargin != 1)
    print_usage ();
  else
    {
      Cell c = args (0).cell_value ();
      if (!  error_state)
        for (octave_idx_type i = 0; i < c.nelem (); i++)
          retval(i) = c.elem (i);
    }

  return retval;
}
```

Note that cell arrays are used less often in standard oct-files and so the '`Cell.h`' header file must be explicitly included. The rest of this example extracts the `octave_values` one by one from the cell array and returns be as individual return arguments. For example consider

```
[b1, b2, b3] = celldemo ({1, [1, 2], "test"})
⇒
b1 =  1
b2 =

   1   2

b3 = test
```

A.1.5 Structures in Oct-Files

A structure in Octave is map between a number of fields represented and their values. The Standard Template Library `map` class is used, with the pair consisting of a `std::string` and an octave `Cell` variable.

A simple example demonstrating the use of structures within oct-files is

```
#include <octave/oct.h>
#include <octave/ov-struct.h>

DEFUN_DLD (structdemo, args, , "Struct demo.")
{
  int nargin = args.length ();
  octave_value retval;
```

```
    if (nargin != 2)
      print_usage ();
    else
      {
        Octave_map arg0 = args(0).map_value ();
        std::string arg1 = args(1).string_value ();

        if (! error_state && arg0.contains (arg1))
          {
            // The following two lines might be written as
            //    octave_value tmp;
            //    for (Octave_map::iterator p0 =
            //        arg0.begin();
            //        p0 != arg0.end(); p0++ )
            //      if (arg0.key (p0) == arg1)
            //        {
            //          tmp = arg0.contents (p0) (0);
            //          break;
            //        }
            // though using seek is more concise.
            Octave_map::const_iterator p1 = arg0.seek (arg1);
            octave_value tmp =  arg0.contents(p1)(0);
            Octave_map st;
            st.assign ("selected", tmp);
            retval = octave_value (st);
          }
      }
    return retval;
  }
```

An example of its use is

```
x.a = 1; x.b = "test"; x.c = [1, 2];
structdemo (x, "b")
⇒ selected = test
```

The commented code above demonstrates how to iterate over all of the fields of the structure, where as the following code demonstrates finding a particular field in a more concise manner.

As can be seen the `contents` method of the `Octave_map` class returns a `Cell` which allows structure arrays to be represented. Therefore, to obtain the underlying `octave_value` we write

```
octave_value tmp = arg0.contents (p1) (0);
```

where the trailing (0) is the () operator on the `Cell` object. We can equally iterate of the elements of the Cell array to address the elements of the structure array.

A.1.6 Sparse Matrices in Oct-Files

There are three classes of sparse objects that are of interest to the user.

`SparseMatrix`
: A double precision sparse matrix class

`SparseComplexMatrix`
: A complex sparse matrix class

`SparseBoolMatrix`
: A boolean sparse matrix class

All of these classes inherit from the `Sparse<T>` template class, and so all have similar capabilities and usage. The `Sparse<T>` class was based on Octave `Array<T>` class, and so users familiar with Octave's `Array` classes will be comfortable with the use of the sparse classes.

The sparse classes will not be entirely described in this section, due to their similarity with the existing `Array` classes. However, there are a few differences due the different nature of sparse objects, and these will be described. Firstly, although it is fundamentally possible to have N-dimensional sparse objects, the Octave sparse classes do not allow them at this time. So all operations of the sparse classes must be 2-dimensional. This means that in fact `SparseMatrix` is similar to Octave's `Matrix` class rather than its `NDArray` class.

A.1.6.1 The Differences between the Array and Sparse Classes

The number of elements in a sparse matrix is considered to be the number of non-zero elements rather than the product of the dimensions. Therefore

```
SparseMatrix sm;
...
int nel = sm.nelem ();
```

returns the number of non-zero elements. If the user really requires the number of elements in the matrix, including the non-zero elements, they should use `numel` rather than `nelem`. Note that for very large matrices, where the product of the two dimensions is larger than the representation of an unsigned int, then `numel` can overflow. An example is `speye(1e6)` which will create a matrix with a million rows and columns, but only a million non-zero elements. Therefore the number of rows by the number of columns in this case is more than two hundred times the maximum value that can be represented by an unsigned int. The use of `numel` should therefore be avoided useless it is known it won't overflow.

Extreme care must be take with the elem method and the "()" operator, which perform basically the same function. The reason is that if a sparse object is non-const, then Octave will assume that a request for a zero element in a sparse matrix is in fact a request to create this element so it can be filled. Therefore a piece of code like

```
SparseMatrix sm;
...
for (int j = 0; j < nc; j++)
  for (int i = 0; i < nr; i++)
    std::cerr << " (" << i << "," << j << "): " << sm(i,j)
              << std::endl;
```

is a great way of turning the sparse matrix into a dense one, and a very slow way at that since it reallocates the sparse object at each zero element in the matrix.

An easy way of preventing the above from happening is to create a temporary constant version of the sparse matrix. Note that only the container for the sparse matrix will be copied, while the actual representation of the data will be shared between the two versions of the sparse matrix. So this is not a costly operation. For example, the above would become

```
SparseMatrix sm;
...
const SparseMatrix tmp (sm);
for (int j = 0; j < nc; j++)
  for (int i = 0; i < nr; i++)
    std::cerr << " (" << i << "," << j << "): " << tmp(i,j)
              << std::endl;
```

Finally, as the sparse types aren't just represented as a contiguous block of memory, the `fortran_vec` method of the `Array<T>` is not available. It is however replaced by three separate methods `ridx`, `cidx` and `data`, that access the raw compressed column format that the Octave sparse matrices are stored in. Additionally, these methods can be used in a manner similar to `elem`, to allow the matrix to be accessed or filled. However, in that case it is up to the user to respect the sparse matrix compressed column format discussed previous.

A.1.6.2 Creating Sparse Matrices in Oct-Files

You have several alternatives for creating a sparse matrix. You can first create the data as three vectors representing the row and column indexes and the data, and from those create the matrix. Or alternatively, you can create a sparse matrix with the appropriate amount of space and then fill in the values. Both techniques have their advantages and disadvantages.

Here is an example of how to create a small sparse matrix with the first technique

```
int nz = 4, nr = 3, nc = 4;

ColumnVector ridx (nz);
ColumnVector cidx (nz);
ColumnVector data (nz);

ridx(0) = 0; ridx(1) = 0; ridx(2) = 1; ridx(3) = 2;
cidx(0) = 0; cidx(1) = 1; cidx(2) = 3; cidx(3) = 3;
data(0) = 1; data(1) = 2; data(2) = 3; data(3) = 4;

SparseMatrix sm (data, ridx, cidx, nr, nc);
```

which creates the matrix given in section Section 21.1.1 [Storage of Sparse Matrices], page 343. Note that the compressed matrix format is not used at the time of the creation of the matrix itself, however it is used internally.

As previously mentioned, the values of the sparse matrix are stored in increasing column-major ordering. Although the data passed by the user does not need to respect this requirement, the pre-sorting the data significantly speeds up the creation of the sparse matrix.

The disadvantage of this technique of creating a sparse matrix is that there is a brief time where two copies of the data exists. Therefore for extremely memory constrained problems this might not be the right technique to create the sparse matrix.

The alternative is to first create the sparse matrix with the desired number of non-zero elements and then later fill those elements in. The easiest way to do this is

```
int nz = 4, nr = 3, nc = 4;
SparseMatrix sm (nr, nc, nz);
sm(0,0) = 1; sm(0,1) = 2; sm(1,3) = 3; sm(2,3) = 4;
```

That creates the same matrix as previously. Again, although it is not strictly necessary, it is significantly faster if the sparse matrix is created in this manner that the elements are added in column-major ordering. The reason for this is that if the elements are inserted at the end of the current list of known elements then no element in the matrix needs to be moved to allow the new element to be inserted. Only the column indexes need to be updated.

There are a few further points to note about this technique of creating a sparse matrix. Firstly, it is possible to create a sparse matrix with fewer elements than are actually inserted in the matrix. Therefore

```
int nz = 4, nr = 3, nc = 4;
SparseMatrix sm (nr, nc, 0);
sm(0,0) = 1; sm(0,1) = 2; sm(1,3) = 3; sm(2,3) = 4;
```

is perfectly valid. However it is a very bad idea. The reason is that as each new element is added to the sparse matrix the space allocated to it is increased by reallocating the memory. This is an expensive operation, that will significantly slow this means of creating a sparse matrix. Furthermore, it is possible to create a sparse matrix with too much storage, so having *nz* above equaling 6 is also valid. The disadvantage is that the matrix occupies more memory than strictly needed.

It is not always easy to know the number of non-zero elements prior to filling a matrix. For this reason the additional storage for the sparse matrix can be removed after its creation with the *maybe_compress* function. Furthermore, the maybe_compress can deallocate the unused storage, but it can equally remove zero elements from the matrix. The removal of zero elements from the matrix is controlled by setting the argument of the *maybe_compress* function to be '`true`'. However, the cost of removing the zeros is high because it implies resorting the elements. Therefore, if possible it is better is the user doesn't add the zeros in the first place. An example of the use of *maybe_compress* is

```
int nz = 6, nr = 3, nc = 4;

SparseMatrix sm1 (nr, nc, nz);
sm1(0,0) = 1; sm1(0,1) = 2; sm1(1,3) = 3; sm1(2,3) = 4;
sm1.maybe_compress ();  // No zero elements were added

SparseMatrix sm2 (nr, nc, nz);
sm2(0,0) = 1; sm2(0,1) = 2; sm(0,2) = 0; sm(1,2) = 0;
sm1(1,3) = 3; sm1(2,3) = 4;
sm2.maybe_compress (true);  // Zero elements were added
```

The use of the *maybe_compress* function should be avoided if possible, as it will slow the creation of the matrices.

A third means of creating a sparse matrix is to work directly with the data in compressed row format. An example of this technique might be

```
octave_value arg;
...
int nz = 6, nr = 3, nc = 4;   // Assume we know the max no nz
SparseMatrix sm (nr, nc, nz);
Matrix m = arg.matrix_value ();

int ii = 0;
sm.cidx (0) = 0;
for (int j = 1; j < nc; j++)
  {
    for (int i = 0; i < nr; i++)
      {
        double tmp = foo (m(i,j));
        if (tmp != 0.)
          {
            sm.data(ii) = tmp;
            sm.ridx(ii) = i;
            ii++;
          }
      }
    sm.cidx(j+1) = ii;
 }
sm.maybe_compress ();  // If don't know a-priori
                       // the final no of nz.
```

which is probably the most efficient means of creating the sparse matrix.

Finally, it might sometimes arise that the amount of storage initially created is insufficient to completely store the sparse matrix. Therefore, the method `change_capacity` exists to reallocate the sparse memory. The above example would then be modified as

```
octave_value arg;
...
int nz = 6, nr = 3, nc = 4;   // Assume we know the max no nz
SparseMatrix sm (nr, nc, nz);
Matrix m = arg.matrix_value ();

int ii = 0;
sm.cidx (0) = 0;
for (int j = 1; j < nc; j++)
  {
    for (int i = 0; i < nr; i++)
      {
        double tmp = foo (m(i,j));
        if (tmp != 0.)
```

```
              {
                if (ii == nz)
                  {
                    nz += 2;   // Add 2 more elements
                    sm.change_capacity (nz);
                  }
                sm.data(ii) = tmp;
                sm.ridx(ii) = i;
                ii++;
              }
          }
        sm.cidx(j+1) = ii;
     }
    sm.maybe_mutate ();  // If don't know a-priori
                         // the final no of nz.
```

Note that both increasing and decreasing the number of non-zero elements in a sparse matrix is expensive, as it involves memory reallocation. Also as parts of the matrix, though not its entirety, exist as the old and new copy at the same time, additional memory is needed. Therefore if possible this should be avoided.

A.1.6.3 Using Sparse Matrices in Oct-Files

Most of the same operators and functions on sparse matrices that are available from the Octave are equally available with oct-files. The basic means of extracting a sparse matrix from an `octave_value` and returning them as an `octave_value`, can be seen in the following example

```
octave_value_list retval;

SparseMatrix sm = args(0).sparse_matrix_value ();
SparseComplexMatrix scm =
    args(1).sparse_complex_matrix_value ();
SparseBoolMatrix sbm = args(2).sparse_bool_matrix_value ();
...
retval(2) = sbm;
retval(1) = scm;
retval(0) = sm;
```

The conversion to an octave-value is handled by the sparse `octave_value` constructors, and so no special care is needed.

A.1.7 Accessing Global Variables in Oct-Files

Global variables allow variables in the global scope to be accessed. Global variables can easily be accessed with oct-files using the support functions `get_global_value` and `set_global_value`. `get_global_value` takes two arguments, the first is a string representing the variable name to obtain. The second argument is a boolean argument specifying what to do in the case that no global variable of the desired name is found. An example of the use of these two functions is

```
#include <octave/oct.h>
```

```
DEFUN_DLD (globaldemo, args, , "Global demo.")
{
  int nargin = args.length ();
  octave_value retval;

  if (nargin != 1)
    print_usage ();
  else
    {
      std::string s = args(0).string_value ();
      if (!  error_state)
        {
          octave_value tmp = get_global_value (s, true);
          if (tmp.is_defined ())
            retval = tmp;
          else
            retval = "Global variable not found";

          set_global_value ("a", 42.0);
        }
    }
  return retval;
}
```

An example of its use is

```
global a b
b = 10;
globaldemo ("b")
⇒ 10
globaldemo ("c")
⇒ "Global variable not found"
num2str (a)
⇒ 42
```

A.1.8 Calling Octave Functions from Oct-Files

There is often a need to be able to call another octave function from within an oct-file, and there are many examples of such within octave itself. For example the `quad` function is an oct-file that calculates the definite integral by quadrature over a user supplied function.

There are also many ways in which a function might be passed. It might be passed as one of

1. Function Handle
2. Anonymous Function Handle
3. Inline Function
4. String

The example below demonstrates an example that accepts all four means of passing a function to an oct-file.

```
#include <octave/oct.h>
#include <octave/parse.h>

DEFUN_DLD (funcdemo, args, nargout, "Function Demo")
{
  int nargin = args.length();
  octave_value_list retval;

  if (nargin < 2)
    print_usage ();
  else
    {
      octave_value_list newargs;
      for (octave_idx_type i = nargin - 1; i > 0; i--)
        newargs (i - 1) = args(i);
      if (args(0).is_function_handle ()
          || args(0).is_inline_function ())
        {
          octave_function *fcn = args(0).function_value ();
          if (! error_state)
            retval = feval (fcn, newargs, nargout);
        }
      else if (args(0).is_string ())
        {
          std::string fcn = args (0).string_value ();
          if (! error_state)
            retval = feval (fcn, newargs, nargout);
        }
      else
        error ("funcdemo: expected string,",
               " inline or function handle");
    }
  return retval;
}
```

The first argument to this demonstration is the user supplied function and the following arguments are all passed to the user function.

```
funcdemo (@sin,1)
⇒ 0.84147
funcdemo (@(x) sin(x), 1)
⇒ 0.84147
funcdemo (inline ("sin(x)"), 1)
⇒ 0.84147
funcdemo ("sin",1)
⇒ 0.84147
funcdemo (@atan2, 1, 1)
⇒ 0.78540
```

When the user function is passed as a string, the treatment of the function is different. In some cases it is necessary to always have the user supplied function as an `octave_function` object. In that case the string argument can be used to create a temporary function like

```
std::octave fcn_name = unique_symbol_name ("__fcn__");
std::string fname = "function y = ";
fname.append (fcn_name);
fname.append ("(x) y = ");
fcn = extract_function (args(0), "funcdemo", fcn_name,
                        fname, "; endfunction");
...
if (fcn_name.length ())
  clear_function (fcn_name);
```

There are two important things to know in this case. The number of input arguments to the user function is fixed, and in the above is a single argument, and secondly to avoid leaving the temporary function in the Octave symbol table it should be cleared after use.

A.1.9 Calling External Code from Oct-Files

Linking external C code to Octave is relatively simple, as the C functions can easily be called directly from C++. One possible issue is the declarations of the external C functions might need to be explicitly defined as C functions to the compiler. If the declarations of the external C functions are in the header `foo.h`, then the manner in which to ensure that the C++ compiler treats these declarations as C code is

```
#ifdef __cplusplus
extern "C"
{
#endif
#include "foo.h"
#ifdef __cplusplus
}  /* end extern "C" */
#endif
```

Calling Fortran code however can pose some difficulties. This is due to differences in the manner in compilers treat the linking of Fortran code with C or C++ code. Octave supplies a number of macros that allow consistent behavior across a number of compilers.

The underlying Fortran code should use the `XSTOPX` function to replace the Fortran `STOP` function. `XSTOPX` uses the Octave exception handler to treat failing cases in the fortran code

explicitly. Note that Octave supplies its own replacement BLAS XERBLA function, which uses XSTOPX.

If the underlying code calls XSTOPX, then the F77_XFCN macro should be used to call the underlying fortran function. The Fortran exception state can then be checked with the global variable f77_exception_encountered. If XSTOPX will not be called, then the F77_FCN macro should be used instead to call the Fortran code.

There is no harm in using F77_XFCN in all cases, except that for Fortran code that is short running and executes a large number of times, there is potentially an overhead in doing so. However, if F77_FCN is used with code that calls XSTOP, Octave can generate a segmentation fault.

An example of the inclusion of a Fortran function in an oct-file is given in the following example, where the C++ wrapper is

```
#include <octave/oct.h>
#include <octave/f77-fcn.h>

extern "C"
{
  F77_RET_T
  F77_FUNC (fortsub, FORTSUB)
        (const int&, double*, F77_CHAR_ARG_DECL
         F77_CHAR_ARG_LEN_DECL);
}

DEFUN_DLD (fortdemo , args , , "Fortran Demo.")
{
  octave_value_list retval;
  int nargin = args.length();
  if (nargin != 1)
    print_usage ();
  else
    {
      NDArray a = args(0).array_value ();
      if (!  error_state)
        {
          double *av = a.fortran_vec ();
          octave_idx_type na = a.nelem ();
          OCTAVE_LOCAL_BUFFER (char, ctmp, 128);

          F77_XFCN (fortsub, FORTSUB, (na, av, ctmp
                    F77_CHAR_ARG_LEN (128)));

          retval(1) = std::string (ctmp);
          retval(0) = a;
        }
    }
  return retval;
```

```
}
```

and the fortran function is

```
      subroutine fortsub (n, a, s)
      implicit none
      character*(*) s
      real*8 a(*)
      integer*4 i, n, ioerr
      do i = 1, n
        if (a(i) .eq.  0d0) then
          call xstopx ('fortsub:  divide by zero')
        else
          a(i) = 1d0 / a(i)
        endif
      enddo
      write (unit = s, fmt = '(a,i3,a,a)', iostat = ioerr)
     $       'There are ', n,
     $       ' values in the input vector', char(0)
      if (ioerr .ne.  0) then
        call xstopx ('fortsub:  error writing string')
      endif
      return
      end
```

This example demonstrates most of the features needed to link to an external Fortran function, including passing arrays and strings, as well as exception handling. An example of the behavior of this function is

```
[b, s] = fortdemo (1:3)
⇒
  b = 1.00000   0.50000   0.33333
  s = There are   3 values in the input vector
[b, s] = fortdemo(0:3)
error: fortsub:divide by zero
error: exception encountered in Fortran subroutine fortsub_
error: fortdemo: error in fortran
```

A.1.10 Allocating Local Memory in Oct-Files

Allocating memory within an oct-file might seem easy as the C++ new/delete operators can be used. However, in that case care must be taken to avoid memory leaks. The preferred manner in which to allocate memory for use locally is to use the `OCTAVE_LOCAL_BUFFER` macro. An example of its use is

```
OCTAVE_LOCAL_BUFFER (double, tmp, len)
```

that returns a pointer `tmp` of type `double *` of length `len`.

A.1.11 Input Parameter Checking in Oct-Files

As oct-files are compiled functions they have the possibility of causing Octave to abort abnormally. It is therefore important that each and every function has the minimum of parameter checking needed to ensure that Octave behaves well.

The minimum requirement, as previously discussed, is to check the number of input arguments before using them to avoid referencing a non existent argument. However, it some case this might not be sufficient as the underlying code imposes further constraints. For example an external function call might be undefined if the input arguments are not integers, or if one of the arguments is zero. Therefore, oct-files often need additional input parameter checking.

There are several functions within Octave that might be useful for the purposes of parameter checking. These include the methods of the octave_value class like `is_real_matrix`, etc., but equally include more specialized functions. Some of the more common ones are demonstrated in the following example

```
#include <octave/oct.h>

DEFUN_DLD (paramdemo, args, nargout,
           "Parameter Check Demo.")
{
  int nargin = args.length ();
  octave_value retval;

  if (nargin != 1)
    print_usage();
  else if (nargout != 0)
    error ("paramdemo:  function has no output arguments");
  else
    {
      NDArray m = args(0).array_value();
      double min_val = -10.0;
      double max_val = 10.0;
      octave_stdout << "Properties of input array:\n";
      if (m.any_element_is_negative ())
        octave_stdout << "  includes negative values\n";
      if (m.any_element_is_inf_or_nan())
        octave_stdout << "  includes Inf or NaN values\n";
      if (m.any_element_not_one_or_zero())
        octave_stdout <<
          "  includes other values than 1 and 0\n";
      if (m.all_elements_are_int_or_inf_or_nan())
        octave_stdout <<
          "  includes only int, Inf or NaN values\n";
      if (m.all_integers (min_val, max_val))
        octave_stdout <<
          "  includes only integers in [-10,10]\n";
    }
```

```
    return retval;
  }
```

and an example of its use is

```
paramdemo ([1, 2, NaN, Inf])
⇒ Properties of input array:
      includes Inf or NaN values
      includes other values than 1 and 0
      includes only int, Inf or NaN values
```

A.1.12 Exception and Error Handling in Oct-Files

Another important feature of Octave is its ability to react to the user typing *Control-C* even during calculations. This ability is based on the C++ exception handler, where memory allocated by the C++ new/delete methods are automatically released when the exception is treated. When writing an oct-file, to allow Octave to treat the user typing *Control-C*, the `OCTAVE_QUIT` macro is supplied. For example

```
for (octave_idx_type i = 0; i < a.nelem (); i++)
  {
    OCTAVE_QUIT;
    b.elem(i) = 2. * a.elem(i);
  }
```

The presence of the `OCTAVE_QUIT` macro in the inner loop allows Octave to treat the user request with the *Control-C*. Without this macro, the user must either wait for the function to return before the interrupt is processed, or press *Control-C* three times to force Octave to exit.

The `OCTAVE_QUIT` macro does impose a very small speed penalty, and so for loops that are known to be small it might not make sense to include `OCTAVE_QUIT`.

When creating an oct-file that uses an external libraries, the function might spend a significant portion of its time in the external library. It is not generally possible to use the `OCTAVE_QUIT` macro in this case. The alternative in this case is

```
BEGIN_INTERRUPT_IMMEDIATELY_IN_FOREIGN_CODE;
...  some code that calls a "foreign" function ...
END_INTERRUPT_IMMEDIATELY_IN_FOREIGN_CODE;
```

The disadvantage of this is that if the foreign code allocates any memory internally, then this memory might be lost during an interrupt, without being deallocated. Therefore, ideally Octave itself should allocate any memory that is needed by the foreign code, with either the fortran_vec method or the `OCTAVE_LOCAL_BUFFER` macro.

The Octave unwind_protect mechanism (Section 10.8 [The `unwind_protect` Statement], page 134) can also be used in oct-files. In conjunction with the exception handling of Octave, it is important to enforce that certain code is run to allow variables, etc. to be restored even if an exception occurs. An example of the use of this mechanism is

```
#include <octave/oct.h>
#include <octave/unwind-prot.h>

void
```

```
err_hand (const char *fmt, ...)
{
  // Do nothing!!
}

DEFUN_DLD (unwinddemo, args, nargout, "Unwind Demo")
{
  int nargin = args.length();
  octave_value retval;
  if (nargin < 2)
    print_usage ();
  else
    {
      NDArray a = args(0).array_value ();
      NDArray b = args(1).array_value ();

      if (!  error_state)
        {
          unwind_protect::begin_frame ("Funwinddemo");
          unwind_protect_ptr
            (current_liboctave_warning_handler);
          set_liboctave_warning_handler(err_hand);
          retval = octave_value (quotient (a, b));
          unwind_protect::run_frame ("Funwinddemo");
        }
    }
  return retval;
}
```

As can be seen in the example

```
unwinddemo (1, 0)
⇒ Inf
1 / 0
⇒ warning: division by zero
   Inf
```

The division by zero (and in fact all warnings) is disabled in the `unwinddemo` function.

A.1.13 Documentation and Test of Oct-Files

The documentation of an oct-file is the fourth string parameter of the `DEFUN_DLD` macro. This string can be formatted in the same manner as the help strings for user functions (Section C.5 [Documentation Tips], page 610), however there are some issue that are particular to the formatting of help strings within oct-files.

The major issue is that the help string will typically be longer than a single line of text, and so the formatting of long help strings need to be taken into account. There are several manners in which to treat this issue, but the most common is illustrated in the following example

```
DEFUN_DLD (do_what_i_want, args, nargout,
  "-*- texinfo -*-\n\
@deftypefn {Function File} {} do_what_i_say (@var{n})\n\
A function that does what the user actually wants rather\n\
than what they requested.\n\
@end deftypefn")
{
...
}
```

where, as can be seen, end line of text within the help string is terminated by `\n\` which is an embedded new-line in the string together with a C++ string continuation character. Note that the final `\` must be the last character on the line.

Octave also includes the ability to embed the test and demonstration code for a function within the code itself (Appendix B [Test and Demo Functions], page 597). This can be used from within oct-files (or in fact any file) with certain provisos. Firstly, the test and demo functions of Octave look for a `%!` as the first characters on a new-line to identify test and demonstration code. This is equally a requirement for oct-files. Furthermore the test and demonstration code must be included in a comment block of the compiled code to avoid it being interpreted by the compiler. Finally, the Octave test and demonstration code must have access to the source code of the oct-file and not just the compiled code as the tests are stripped from the compiled code. An example in an oct-file might be

```
/*

%!error (sin())
%!error (sin(1,1))
%!assert (sin([1,2]),[sin(1),sin(2)])

*/
```

A.2 Mex-Files

Octave includes an interface to allow legacy mex-files to be compiled and used with Octave. This interface can also be used to share code between Octave and non Octave users. However, as mex-files expose the internal API of an alternative product to Octave, and the internal structure of Octave is different to this product, a mex-file can never have the same performance in Octave as the equivalent oct-file. In particular to support the manner in which mex-files access the variables passed to mex functions, there are a significant number of additional copies of memory when calling or returning from a mex function. For this reason, new code should be written using the oct-file interface discussed above if possible.

A.2.1 Getting Started with Mex-Files

The basic command to build a mex-file is either `mkoctfile --mex` or `mex`. The first can either be used from within Octave or from the command line. However, to avoid issues with the installation of other products, the use of the command `mex` is limited to within Octave.

`mex` [*options*] *file* . . . [Function File]

Compile source code written in C, C++, or Fortran, to a MEX file. This is equivalent to `mkoctfile --mex [options] file`.

See also: [mkoctfile], page 557.

`mexext` () [Function File]

Return the filename extension used for MEX files.

One important difference between the use of mex with other products and with Octave is that the header file "matrix.h" is implicitly included through the inclusion of "mex.h". This is to avoid a conflict with the Octave file "Matrix.h" with operating systems and compilers that don't distinguish between filenames in upper and lower case

Consider the short example

```
#include "mex.h"

void
mexFunction (int nlhs, mxArray *plhs[], int nrhs,
             const mxArray *prhs[])
{
  mxArray *v = mxCreateDoubleMatrix (1, 1, mxREAL);
  double *data = mxGetPr (v);
  *data = 1.23456789;
  plhs[0] = v;
}
```

This simple example demonstrates the basics of writing a mex-file. The entry point into the mex-file is defined by `mexFunction`. Note that the function name is not explicitly included in the `mexFunction` and so there can only be a single `mexFunction` entry point per-file. Also the name of the function is determined by the name of the mex-file itself. Therefore if the above function is in the file '`firstmexdemo.c`', it can be compiled with

```
mkoctfile --mex firstmexdemo.c
```

which creates a file '`firstmexdemo.mex`'. The function can then be run from Octave as

```
firstmexdemo()
⇒ 1.2346
```

It should be noted that the mex-file contains no help string for the functions it contains. To document mex-files, there should exist an m-file in the same directory as the mex-file itself. Taking the above as an example, we would therefore have a file '`firstmexdemo.m`' that might contain the text

```
%FIRSTMEXDEMO Simple test of the functionality of a mex-file.
```

In this case, the function that will be executed within Octave will be given by the mex-file, while the help string will come from the m-file. This can also be useful to allow a sample implementation of the mex-file within the Octave language itself for testing purposes.

Although we cannot have multiple entry points into a single mex-file, we can use the `mexFunctionName` function to determine what name the mex-file was called with. This can be used to alter the behavior of the mex-file based on the function name. For example if

```
#include "mex.h"

void
mexFunction (int nlhs, mxArray *plhs[], int nrhs,
             const mxArray *prhs[])
{
  const char *nm;
  nm = mexFunctionName ();
  mexPrintf ("You called function:  %s\n", nm);
  if (strcmp (nm, "myfunc") == 0)
    mexPrintf ("This is the principal function\n", nm);
  return;
}
```

is in file 'myfunc.c', and it is compiled with

```
mkoctfile --mex myfunc.c
ln -s myfunc.mex myfunc2.mex
```

Then as can be seen by

```
myfunc()
⇒ You called function: myfunc
    This is the principal function
myfunc2()
⇒ You called function: myfunc2
```

the behavior of the mex-file can be altered depending on the functions name.

Allow the user should only include `mex.h` in their code, Octave declares additional functions, typedefs, etc., available to the user to write mex-files in the headers `mexproto.h` and `mxarray.h`.

A.2.2 Working with Matrices and Arrays in Mex-Files

The basic mex type of all variables is `mxArray`. All variables, such as matrices, cell arrays or structures are all stored in this basic type, and this type serves basically the same purpose as the octave_value class in oct-files. That is it acts as a container for the more specialized types.

The `mxArray` structure contains at a minimum, the variable it represents name, its dimensions, its type and whether the variable is real or complex. It can however contain a number of additional fields depending on the type of the `mxArray`. There are a number of functions to create `mxArray` structures, including `mxCreateCellArray`, `mxCreateSparse` and the generic `mxCreateNumericArray`.

The basic functions to access the data contained in an array is `mxGetPr`. As the mex interface assumes that the real and imaginary parts of a complex array are stored separately, there is an equivalent function `mxGetPi` that get the imaginary part. Both of these functions are for use only with double precision matrices. There also exists the generic function `mxGetData` and `mxGetImagData` that perform the same operation on all matrix types. For example

```
mxArray *m;
mwSize *dims;
UINT32_T *pr;

dims = (mwSize *) mxMalloc (2 * sizeof(mwSize));
dims[0] = 2;
dims[1] = 2;
m = mxCreateNumericArray (2, dims, mxUINT32_CLASS, mxREAL);
pr =  = (UINT32_T *) mxGetData (m);
```

There are also the functions `mxSetPr`, etc., that perform the inverse, and set the data of an Array to use the block of memory pointed to by the argument of `mxSetPr`.

Note the type `mwSize` used above, and `mwIndex` are defined as the native precision of the indexing in Octave on the platform on which the mex-file is built. This allows both 32- and 64-bit platforms to support mex-files. `mwSize` is used to define array dimension and maximum number or elements, while `mwIndex` is used to define indexing into arrays.

An example that demonstration how to work with arbitrary real or complex double precision arrays is given by the file '`mypow2.c`' as given below.

```
#include "mex.h"

void
mexFunction (int nlhs, mxArray* plhs[], int nrhs,
             const mxArray* prhs[])
{
  mwIndex i;
  mwSize n;
  double *vri, *vro;

  if (nrhs != 1 || !  mxIsNumeric (prhs[0]))
    mexErrMsgTxt ("expects matrix");

  n = mxGetNumberOfElements (prhs[0]);
  plhs[0] = (mxArray *) mxCreateNumericArray
    (mxGetNumberOfDimensions (prhs[0]),
     mxGetDimensions (prhs[0]), mxGetClassID (prhs[0]),
     mxIsComplex (prhs[0]));
  vri = mxGetPr (prhs[0]);
  vro = mxGetPr (plhs[0]);

  if (mxIsComplex (prhs[0]))
    {
      double *vii, *vio;
      vii = mxGetPi (prhs[0]);
      vio = mxGetPi (plhs[0]);

      for (i = 0; i < n; i++)
        {
```

```
          vro [i] = vri [i] * vri [i] - vii [i] * vii [i];
          vio [i] = 2 * vri [i] * vii [i];
        }
    }
  else
    {
      for (i = 0; i < n; i++)
        vro [i] = vri [i] * vri [i];
    }
}
```

with an example of its use

```
b = randn(4,1) + 1i * randn(4,1);
all(b.^2 == mypow2(b))
⇒ 1
```

The example above uses the functions `mxGetDimensions`, `mxGetNumberOfElements`, and `mxGetNumberOfDimensions` to work with the dimensions of multi-dimensional arrays. The functions `mxGetM`, and `mxGetN` are also available to find the number of rows and columns in a matrix.

A.2.3 Character Strings in Mex-Files

As mex-files do not make the distinction between single and double quoted strings within Octave, there is perhaps less complexity in the use of strings and character matrices in mex-files. An example of their use, that parallels the demo in '`stringdemo.cc`', is given in the file '`mystring.c`', as seen below.

```
#include <string.h>
#include "mex.h"

void
mexFunction (int nlhs, mxArray *plhs[], int nrhs,
             const mxArray *prhs[])
{
  mwIndex i, j;
  mwSize m, n;
  mxChar *pi, *po;

  if (nrhs != 1 || !  mxIsChar (prhs[0]) ||
      mxGetNumberOfDimensions (prhs[0]) > 2)
    mexErrMsgTxt ("expecting char matrix");

  m = mxGetM (prhs[0]);
  n = mxGetN (prhs[0]);
  pi = mxGetChars (prhs[0]);
  plhs[0] = mxCreateNumericMatrix (m, n, mxCHAR_CLASS,
                                   mxREAL);
  po = mxGetChars (plhs[0]);
```

```
  for (j = 0; j < n; j++)
    for (i = 0; i < m; i++)
      po [j*m + m - 1 - i] = pi [j*m + i];
}
```

An example of its expected output is

```
mystring(["First String"; "Second String"])
⇒ s1 = Second String
        First String
```

Other functions in the mex interface for handling character strings are `mxCreateString`, `mxArrayToString`, and `mxCreateCharMatrixFromStrings`. In a mex-file, a character string is considered to be a vector rather than a matrix. This is perhaps an arbitrary distinction as the data in the mxArray for the matrix is consecutive in any case.

A.2.4 Cell Arrays with Mex-Files

We can perform exactly the same operations in Cell arrays in mex-files as we can in oct-files. An example that reduplicates the functional of the '`celldemo.cc`' oct-file in a mex-file is given by '`mycell.c`' as below

```
#include "mex.h"

void
mexFunction (int nlhs, mxArray* plhs[], int nrhs,
             const mxArray* prhs[])
{
  mwSize n;
  mwIndex i;

  if (nrhs != 1 || !  mxIsCell (prhs[0]))
    mexErrMsgTxt ("expects cell");

  n = mxGetNumberOfElements (prhs[0]);
  n = (n > nlhs ?  nlhs :  n);

  for (i = 0; i < n; i++)
    plhs[i] = mxDuplicateArray (mxGetCell (prhs[0], i));
}
```

which as can be seen below has exactly the same behavior as the oct-file version.

```
[b1, b2, b3] = mycell ({1, [1, 2], "test"})
⇒
b1 =  1
b2 =

   1   2

b3 = test
```

Note in the example the use of the `mxDuplicateArray` function. This is needed as the `mxArray` pointer returned by `mxGetCell` might be deallocated. The inverse function to `mxGetCell` is `mcSetCell` and is defined as

```
void mxSetCell (mxArray *ptr, int idx, mxArray *val);
```

Finally, to create a cell array or matrix, the appropriate functions are

```
mxArray *mxCreateCellArray (int ndims, const int *dims);
mxArray *mxCreateCellMatrix (int m, int n);
```

A.2.5 Structures with Mex-Files

The basic function to create a structure in a mex-file is `mxCreateStructMatrix`, which creates a structure array with a two dimensional matrix, or `mxCreateStructArray`.

```
mxArray *mxCreateStructArray (int ndims, int *dims,
                              int num_keys,
                              const char **keys);
mxArray *mxCreateStructMatrix (int rows, int cols,
                               int num_keys,
                               const char **keys);
```

Accessing the fields of the structure can then be performed with the `mxGetField` and `mxSetField` or alternatively with the `mxGetFieldByNumber` and `mxSetFieldByNumber` functions.

```
mxArray *mxGetField (const mxArray *ptr, mwIndex index,
                     const char *key);
mxArray *mxGetFieldByNumber (const mxArray *ptr,
                             mwIndex index, int key_num);
void mxSetField (mxArray *ptr, mwIndex index,
                 const char *key, mxArray *val);
void mxSetFieldByNumber (mxArray *ptr, mwIndex index,
                         int key_num, mxArray *val);
```

A difference between the oct-file interface to structures and the mex-file version is that the functions to operate on structures in mex-files directly include an `index` over the elements of the arrays of elements per `field`. Whereas the oct-file structure includes a Cell Array per field of the structure.

An example that demonstrates the use of structures in mex-file can be found in the file '`mystruct.c`', as seen below

```
#include "mex.h"

void
mexFunction (int nlhs, mxArray* plhs[], int nrhs,
             const mxArray* prhs[])
{
  int i;
  mwIndex j;
  mxArray *v;
  const char *keys[] = { "this", "that" };
```

```
    if (nrhs != 1 || !  mxIsStruct (prhs[0]))
      mexErrMsgTxt ("expects struct");

    for (i = 0; i < mxGetNumberOfFields (prhs[0]); i++)
      for (j = 0; j < mxGetNumberOfElements (prhs[0]); j++)
        {
          mexPrintf ("field %s(%d) = ",
                     mxGetFieldNameByNumber (prhs[0], i), j);
          v = mxGetFieldByNumber (prhs[0], j, i);
          mexCallMATLAB (0, 0, 1, &v, "disp");
        }

    v = mxCreateStructMatrix (2, 2, 2, keys);

    mxSetFieldByNumber (v, 0, 0, mxCreateString ("this1"));
    mxSetFieldByNumber (v, 0, 1, mxCreateString ("that1"));
    mxSetFieldByNumber (v, 1, 0, mxCreateString ("this2"));
    mxSetFieldByNumber (v, 1, 1, mxCreateString ("that2"));
    mxSetFieldByNumber (v, 2, 0, mxCreateString ("this3"));
    mxSetFieldByNumber (v, 2, 1, mxCreateString ("that3"));
    mxSetFieldByNumber (v, 3, 0, mxCreateString ("this4"));
    mxSetFieldByNumber (v, 3, 1, mxCreateString ("that4"));

    if (nlhs)
      plhs[0] = v;
  }
```

An example of the behavior of this function within Octave is then

```
a(1).f1 = "f11"; a(1).f2 = "f12";
a(2).f1 = "f21"; a(2).f2 = "f22";
b = mystruct(a)
⇒ field f1(0) = f11
    field f1(1) = f21
    field f2(0) = f12
    field f2(1) = f22
    b =
    {
      this =

      (,
        [1] = this1
        [2] = this2
        [3] = this3
        [4] = this4
      ,)

      that =
```

```
    (,
      [1] = that1
      [2] = that2
      [3] = that3
      [4] = that4
    ,)

}
```

A.2.6 Sparse Matrices with Mex-Files

The Octave format for sparse matrices is identical to the mex format in that it is a compressed column sparse format. Also in both, sparse matrices are required to be two-dimensional. The only difference is that the real and imaginary parts of the matrix are stored separately.

The mex-file interface, as well as using `mxGetM`, `mxGetN`, `mxSetM`, `mxSetN`, `mxGetPr`, `mxGetPi`, `mxSetPr` and `mxSetPi`, the mex-file interface supplies the functions

```
mwIndex *mxGetIr (const mxArray *ptr);
mwIndex *mxGetJc (const mxArray *ptr);
mwSize mxGetNzmax (const mxArray *ptr);

void mxSetIr (mxArray *ptr, mwIndex *ir);
void mxSetJc (mxArray *ptr, mwIndex *jc);
void mxSetNzmax (mxArray *ptr, mwSize nzmax);
```

`mxGetNzmax` gets the maximum number of elements that can be stored in the sparse matrix. This is not necessarily the number of non-zero elements in the sparse matrix. `mxGetJc` returns an array with one additional value than the number of columns in the sparse matrix. The difference between consecutive values of the array returned by `mxGetJc` define the number of non-zero elements in each column of the sparse matrix. Therefore

```
mwSize nz, n;
mwIndex *Jc;
mxArray *m;
...
n = mxGetN (m);
Jc = mxGetJc (m);
nz = Jc[n];
```

returns the actual number of non-zero elements stored in the matrix in `nz`. As the arrays returned by `mxGetPr` and `mxGetPi` only contain the non-zero values of the matrix, we also need a pointer to the rows of the non-zero elements, and this is given by `mxGetIr`. A complete example of the use of sparse matrices in mex-files is given by the file '`mysparse.c`' as seen below

```
#include "mex.h"

void
mexFunction (int nlhs, mxArray *plhs[], int nrhs,
```

```
             const mxArray *prhs[])
{
  mwSize n, m, nz;
  mxArray *v;
  mwIndex i;
  double *pr, *pi;
  double *pr2, *pi2;
  mwIndex *ir, *jc;
  mwIndex *ir2, *jc2;

  if (nrhs != 1 || !  mxIsSparse (prhs[0]))
    mexErrMsgTxt ("expects sparse matrix");

  m = mxGetM (prhs [0]);
  n = mxGetN (prhs [0]);
  nz = mxGetNzmax (prhs [0]);

  if (mxIsComplex (prhs[0]))
    {
      mexPrintf ("Matrix is %d-by-%d complex",
                 " sparse matrix", m, n);
      mexPrintf (" with %d elements\n", nz);

      pr = mxGetPr (prhs[0]);
      pi = mxGetPi (prhs[0]);
      ir = mxGetIr (prhs[0]);
      jc = mxGetJc (prhs[0]);

      i = n;
      while (jc[i] == jc[i-1] && i != 0) i--;
      mexPrintf ("last non-zero element (%d, %d) =",
                 ir[nz-1]+ 1, i);
      mexPrintf (" (%g, %g)\n", pr[nz-1], pi[nz-1]);

      v = mxCreateSparse (m, n, nz, mxCOMPLEX);
      pr2 = mxGetPr (v);
      pi2 = mxGetPi (v);
      ir2 = mxGetIr (v);
      jc2 = mxGetJc (v);

      for (i = 0; i < nz; i++)
        {
          pr2[i] = 2 * pr[i];
          pi2[i] = 2 * pi[i];
          ir2[i] = ir[i];
        }
      for (i = 0; i < n + 1; i++)
```

```
        jc2[i] = jc[i];

      if (nlhs > 0)
        plhs[0] = v;
    }
  else if (mxIsLogical (prhs[0]))
    {
      bool *pbr, *pbr2;
      mexPrintf ("Matrix is %d-by-%d logical",
                 " sparse matrix", m, n);
      mexPrintf (" with %d elements\n", nz);

      pbr = mxGetLogicals (prhs[0]);
      ir = mxGetIr (prhs[0]);
      jc = mxGetJc (prhs[0]);

      i = n;
      while (jc[i] == jc[i-1] && i != 0) i--;
      mexPrintf ("last non-zero element (%d, %d) = %d\n",
                 ir[nz-1]+ 1, i, pbr[nz-1]);

      v = mxCreateSparseLogicalMatrix (m, n, nz);
      pbr2 = mxGetLogicals (v);
      ir2 = mxGetIr (v);
      jc2 = mxGetJc (v);

      for (i = 0; i < nz; i++)
        {
          pbr2[i] = pbr[i];
          ir2[i] = ir[i];
        }
      for (i = 0; i < n + 1; i++)
        jc2[i] = jc[i];

      if (nlhs > 0)
        plhs[0] = v;
    }
  else
    {
      mexPrintf ("Matrix is %d-by-%d real",
                 " sparse matrix", m, n);
      mexPrintf (" with %d elements\n", nz);

      pr = mxGetPr (prhs[0]);
      ir = mxGetIr (prhs[0]);
      jc = mxGetJc (prhs[0]);
```

```
      i = n;
      while (jc[i] == jc[i-1] && i != 0) i--;
      mexPrintf ("last non-zero element (%d, %d) = %g\n",
                 ir[nz-1]+ 1, i, pr[nz-1]);

      v = mxCreateSparse (m, n, nz, mxREAL);
      pr2 = mxGetPr (v);
      ir2 = mxGetIr (v);
      jc2 = mxGetJc (v);

      for (i = 0; i < nz; i++)
        {
          pr2[i] = 2 * pr[i];
          ir2[i] = ir[i];
        }
      for (i = 0; i < n + 1; i++)
        jc2[i] = jc[i];

      if (nlhs > 0)
        plhs[0] = v;
    }
}
```

A.2.7 Calling Other Functions in Mex-Files

It is also possible call other Octave functions from within a mex-file using `mexCallMATLAB`. An example of the use of `mexCallMATLAB` can be see in the example below

```
#include "mex.h"

void
mexFunction (int nlhs, mxArray* plhs[], int nrhs,
             const mxArray* prhs[])
{
  char *str;

  mexPrintf ("Hello, World!\n");

  mexPrintf ("I have %d inputs and %d outputs\n", nrhs,
             nlhs);

  if (nrhs < 1 || !  mxIsString (prhs[0]))
    mexErrMsgTxt ("function name expected");

  str = mxArrayToString (prhs[0]);

  mexPrintf ("I'm going to call the function %s\n", str);
```

```
  mexCallMATLAB (nlhs, plhs, nrhs-1, prhs+1, str);

  mxFree (str);
}
```

If this code is in the file 'myfeval.c', and is compiled to 'myfeval.mex', then an example of its use is

```
myfeval("sin", 1)
a = myfeval("sin", 1)
⇒ Hello, World!
    I have 2 inputs and 1 outputs
    I'm going to call the interpreter function sin
    a =  0.84147
```

Note that it is not possible to use function handles or inline functions within a mex-file.

A.3 Standalone Programs

The libraries Octave itself uses, can be utilized in standalone applications. These applications then have access, for example, to the array and matrix classes as well as to all the Octave algorithms. The following C++ program, uses class Matrix from liboctave.a or liboctave.so.

```
#include <iostream>
#include <octave/oct.h>

int
main (void)
{
  std::cout << "Hello Octave world!\n";
  int n = 2;
  Matrix a_matrix = Matrix (n, n);
  for (octave_idx_type i = 0; i < n; i++)
    {
      for (octave_idx_type j = 0; j < n; j++)
        {
          a_matrix (i, j) = (i + 1) * 10 + (j + 1);
        }
    }
  std::cout << a_matrix;
  return 0;
}
```

mkoctfile can then be used to build a standalone application with a command like

```
$ mkoctfile --link-stand-alone standalone.cc -o standalone
$ ./standalone
Hello Octave world!
  11 12
  21 22
$
```

Note that the application `hello` will be dynamically linked against the octave libraries and any octave support libraries. The above allows the Octave math libraries to be used by an application. It does not however allow the script files, oct-files or builtin functions of Octave to be used by the application. To do that the Octave interpreter needs to be initialized first. An example of how to do this can then be seen in the code

```
#include <iostream>
#include <octave/oct.h>
#include <octave/octave.h>
#include <octave/parse.h>

int
main (void)
{
  string_vector argv (2);
  argv(0) = "embedded";
  argv(1) = "-q";

  octave_main (2, argv.c_str_vec(), 1);

  octave_idx_type n = 2;
  Matrix a_matrix = Matrix (1, 2);

  std::cout << "GCD of [";
  for (octave_idx_type i = 0; i < n; i++)
    {
      a_matrix (i) = 5 * (i + 1);
      if (i != 0)
        std::cout << ", " << 5 * (i + 2);
      else
        std::cout << 5 * (i + 2);
    }
  std::cout << "] is ";

  octave_value_list in = octave_value (a_matrix);
  octave_value_list out = feval ("gcd", in, 1);

  if (!error_state && out.length () > 0)
    {
      a_matrix = out(0).matrix_value ();
      if (a_matrix.numel () == 1)
        std::cout << a_matrix(0) << "\n";
      else
        std::cout << "invalid\n";
    }
  else
    std::cout << "invalid\n";

  return 0;
}
```

which is compiled and run as before as a standalone application with

```
$ mkoctfile --link-stand-alone embedded.cc -o embedded
$ ./embedded
GCD of [10, 15] is 5
$
```

Appendix B Test and Demo Functions

Octave includes a number of functions to allow the integration of testing and demonstration code in the source code of the functions themselves.

B.1 Test Functions

`test` *name* [Function File]
`test` *name* *quiet*|*normal*|*verbose* [Function File]
`test` (*'name'*, *'quiet|normal|verbose'*, *fid*) [Function File]
`test` ([], *'explain'*, *fid*) [Function File]
success = `test` (...) [Function File]
[*n*, *max*] = `test` (...) [Function File]
[*code*, *idx*] = `test` (*'name'*,*'grabdemo'*) [Function File]

Perform tests from the first file in the loadpath matching *name*. `test` can be called as a command or as a function. Called with a single argument *name*, the tests are run interactively and stop after the first error is encountered.

With a second argument the tests which are performed and the amount of output is selected.

'quiet' Don't report all the tests as they happen, just the errors.

'normal' Report all tests as they happen, but don't do tests which require user interaction.

'verbose' Do tests which require user interaction.

The argument *fid* can be used to allow batch processing. Errors can be written to the already open file defined by *fid*, and hopefully when Octave crashes this file will tell you what was happening when it did. You can use `stdout` if you want to see the results as they happen. You can also give a file name rather than an *fid*, in which case the contents of the file will be replaced with the log from the current test.

Called with a single output argument *success*, `test` returns true if all of the tests were successful. Called with two output arguments *n* and *max*, the number of successful tests and the total number of tests in the file *name* are returned.

If the second argument is the string 'grabdemo', the contents of the demo blocks are extracted but not executed. Code for all code blocks is concatenated and returned as *code* with *idx* being a vector of positions of the ends of the demo blocks.

If the second argument is 'explain', then *name* is ignored and an explanation of the line markers used is written to the file *fid*.

See also: [error], page 161, [assert], page 600, [fail], page 601, [demo], page 601, [example], page 602.

`test` scans the named script file looking for lines which start with `%!`. The prefix is stripped off and the rest of the line is processed through the Octave interpreter. If the code generates an error, then the test is said to fail.

Since `eval()` will stop at the first error it encounters, you must divide your tests up into blocks, with anything in a separate block evaluated separately. Blocks are introduced by the keyword `test` immediately following the `%!`. For example,

```
%!test error ("this test fails!");
%!test "test doesn't fail. it doesn't generate an error";
```

When a test fails, you will see something like:

```
  ***** test error ('this test fails!')
!!!!! test failed
this test fails!
```

Generally, to test if something works, you want to assert that it produces a correct value. A real test might look something like

```
%!test
%! a = [1, 2, 3; 4, 5, 6]; B = [1; 2];
%! expect = [ a ; 2*a ];
%! get = kron (b, a);
%! if (any(size(expect) != size(get)))
%!   error ("wrong size: expected %d,%d but got %d,%d",
%!          size(expect), size(get));
%! elseif (any(any(expect!=get)))
%!   error ("didn't get what was expected.");
%! endif
```

To make the process easier, use the `assert` function. For example, with `assert` the previous test is reduced to:

```
%!test
%! a = [1, 2, 3; 4, 5, 6]; b = [1; 2];
%! assert (kron (b, a), [ a; 2*a ]);
```

`assert` can accept a tolerance so that you can compare results absolutely or relatively. For example, the following all succeed:

```
%!test assert (1+eps, 1, 2*eps)          # absolute error
%!test assert (100+100*eps, 100, -2*eps) # relative error
```

You can also do the comparison yourself, but still have assert generate the error:

```
%!test assert (isempty([]))
%!test assert ([ 1,2; 3,4 ] > 0)
```

Because `assert` is so frequently used alone in a test block, there is a shorthand form:

```
%!assert (...)
```

which is equivalent to:

```
%!test assert (...)
```

Sometimes during development there is a test that should work but is known to fail. You still want to leave the test in because when the final code is ready the test should pass, but you may not be able to fix it immediately. To avoid unnecessary bug reports for these known failures, mark the block with `xtest` rather than `test`:

```
%!xtest assert (1==0)
%!xtest fail ('success=1','error'))
```

Another use of `xtest` is for statistical tests which should pass most of the time but are known to fail occasionally.

Each block is evaluated in its own function environment, which means that variables defined in one block are not automatically shared with other blocks. If you do want to share variables, then you must declare them as `shared` before you use them. For example, the following declares the variable *a*, gives it an initial value (default is empty), then uses it in several subsequent tests.

```
%!shared a
%! a = [1, 2, 3; 4, 5, 6];
%!assert (kron ([1; 2], a), [ a; 2*a ]);
%!assert (kron ([1, 2], a), [ a, 2*a ]);
%!assert (kron ([1,2; 3,4], a), [ a,2*a; 3*a,4*a ]);
```

You can share several variables at the same time:

```
%!shared a, b
```

You can also share test functions:

```
%!function a = fn(b)
%!  a = 2*b;
%!assert (a(2),4);
```

Note that all previous variables and values are lost when a new shared block is declared.

Error and warning blocks are like test blocks, but they only succeed if the code generates an error. You can check the text of the error is correct using an optional regular expression `<pattern>`. For example:

```
%!error <passes!> error('this test passes!');
```

If the code doesn't generate an error, the test fails. For example,

```
%!error "this is an error because it succeeds.";
```

produces

```
***** error "this is an error because it succeeds.";
!!!!! test failed: no error
```

It is important to automate the tests as much as possible, however some tests require user interaction. These can be isolated into demo blocks, which if you are in batch mode, are only run when called with `demo` or `verbose`. The code is displayed before it is executed. For example,

```
%!demo
%! t=[0:0.01:2*pi]; x=sin(t);
%! plot(t,x);
%! you should now see a sine wave in your figure window
```

produces

```
> t=[0:0.01:2*pi]; x=sin(t);
> plot(t,x);
> you should now see a sine wave in your figure window
Press <enter> to continue:
```

Note that demo blocks cannot use any shared variables. This is so that they can be executed by themselves, ignoring all other tests.

If you want to temporarily disable a test block, put `#` in place of the block type. This creates a comment block which is echoed in the log file, but is not executed. For example:

```
%!#demo
%! t=[0:0.01:2*pi]; x=sin(t);
%! plot(t,x);
%! you should now see a sine wave in your figure window
```

Block type summary:

`%!test` check that entire block is correct

`%!error` check for correct error message

`%!warning`
check for correct warning message

`%!demo` demo only executes in interactive mode

`%!#` comment: ignore everything within the block

`%!shared x,y,z`
declares variables for use in multiple tests

`%!function`
defines a function value for a shared variable

`%!assert (x, y, tol)`
shorthand for %!test assert (x, y, tol)

You can also create test scripts for builtins and your own C++ functions. Just put a file of the function name on your path without any extension and it will be picked up by the test procedure. You can even embed tests directly in your C++ code:

```
#if 0
%!test disp('this is a test')
#endif
```

or

```
/*
%!test disp('this is a test')
*/
```

but then the code will have to be on the load path and the user will have to remember to type test('name.cc'). Conversely, you can separate the tests from normal Octave script files by putting them in plain files with no extension rather than in script files.

`assert` (*cond*) [Function File]
`assert` (*cond*, *errmsg*, ...) [Function File]
`assert` (*cond*, *msg_id*, *errmsg*, ...) [Function File]
`assert` (*observed*,*expected*) [Function File]
`assert` (*observed*,*expected*,*tol*) [Function File]

Produces an error if the condition is not met. `assert` can be called in three different ways.

`assert (`*cond*`)`
`assert (`*cond*`, `*errmsg*`, ...)`
`assert (`*cond*`, `*msg_id*`, `*errmsg*`, ...)`
Called with a single argument *cond*, `assert` produces an error if *cond* is zero. If called with a single argument a generic error message. With

more than one argument, the additional arguments are passed to the `error` function.

`assert (`*observed*`, `*expected*`)`
: Produce an error if observed is not the same as expected. Note that observed and expected can be strings, scalars, vectors, matrices, lists or structures.

`assert(`*observed*`, `*expected*`, `*tol*`)`
: Accept a tolerance when comparing numbers. If *tol* is positive use it as an absolute tolerance, will produce an error if `abs(`*observed* `-` *expected*`) > abs(`*tol*`)`. If *tol* is negative use it as a relative tolerance, will produce an error if `abs(`*observed* `-` *expected*`) > abs(`*tol* `*` *expected*`)`. If *expected* is zero *tol* will always be used as an absolute tolerance.

See also: [test], page 597.

`fail (`*code*,*pattern*`)` [Function File]
`fail (`*code*,'*warning*',*pattern*`)` [Function File]

Return true if *code* fails with an error message matching *pattern*, otherwise produce an error. Note that *code* is a string and if *code* runs successfully, the error produced is:

```
expected error but got none
```

If the code fails with a different error, the message produced is:

```
expected <pattern>
but got <text of actual error>
```

The angle brackets are not part of the output.

Called with three arguments, the behavior is similar to `fail(`*code*`, `*pattern*`)`, but produces an error if no warning is given during code execution or if the code fails.

B.2 Demonstration Functions

`demo ('`*name*`',`*n*`)` [Function File]

Runs any examples associated with the function '*name*'. Examples are stored in the script file, or in a file with the same name but no extension somewhere on your path. To keep them separate from the usual script code, all lines are prefixed by `%!`. Each example is introduced by the keyword 'demo' flush left to the prefix, with no intervening spaces. The remainder of the example can contain arbitrary Octave code. For example:

```
%!demo
%! t=0:0.01:2*pi; x = sin(t);
%! plot(t,x)
%! %-------------------------------------------------
%! % the figure window shows one cycle of a sine wave
```

Note that the code is displayed before it is executed, so a simple comment at the end suffices. It is generally not necessary to use disp or printf within the demo.

Demos are run in a function environment with no access to external variables. This means that all demos in your function must use separate initialization code. Alternatively, you can combine your demos into one huge demo, with the code:

```
%! input("Press <enter> to continue: ","s");
```

between the sections, but this is discouraged. Other techniques include using multiple plots by saying figure between each, or using subplot to put multiple plots in the same window.

Also, since demo evaluates inside a function context, you cannot define new functions inside a demo. Instead you will have to use `eval(example('function',n))` to see them. Because eval only evaluates one line, or one statement if the statement crosses multiple lines, you must wrap your demo in "if 1 <demo stuff> endif" with the 'if' on the same line as 'demo'. For example,

```
%!demo if 1
%!  function y=f(x)
%!    y=x;
%!  endfunction
%!  f(3)
%! endif
```

See also: [test], page 597, [example], page 602.

`rundemos (`*`directory`*`)` [Function File]

`example (`*`'name'`*`,`*`n`*`)` [Function File]
`[`*`x`*`, `*`idx`*`] = example (`*`'name'`*`,`*`n`*`)` [Function File]

Display the code for example *n* associated with the function '*name*', but do not run it. If *n* is not given, all examples are displayed.

Called with output arguments, the examples are returned in the form of a string *x*, with *idx* indicating the ending position of the various examples.

See `demo` for a complete explanation.

See also: [demo], page 601, [test], page 597.

`speed (`*`f`*`, `*`init`*`, `*`max_n`*`, `*`f2`*`, `*`tol`*`)` [Function File]
`[`*`order`*`, `*`n`*`, `*`T_f`*`, `*`T_f2`*`] = speed (...)` [Function File]

Determine the execution time of an expression for various *n*. The *n* are log-spaced from 1 to *max_n*. For each *n*, an initialization expression is computed to create whatever data are needed for the test. If a second expression is given, the execution times of the two expressions will be compared. Called without output arguments the results are presented graphically.

f The expression to evaluate.

max_n The maximum test length to run. Default value is 100. Alternatively, use `[min_n,max_n]` or for complete control, `[n1,n2,...,nk]`.

init Initialization expression for function argument values. Use *k* for the test number and *n* for the size of the test. This should compute values for all variables listed in args. Note that init will be evaluated first for $k = 0$, so things which are constant throughout the test can be computed then. The default value is `x = randn (`*`n`*`, 1);`.

f2 An alternative expression to evaluate, so the speed of the two can be compared. Default is [].

tol If *tol* is Inf, then no comparison will be made between the results of expression *f* and expression *f2*. Otherwise, expression *f* should produce a value *v* and expression *f2* should produce a value *v2*, and these shall be compared using assert(*v*,*v2*,*tol*). If *tol* is positive, the tolerance is assumed to be absolute. If *tol* is negative, the tolerance is assumed to be relative. The default is eps.

order The time complexity of the expression O(a n^p). This is a structure with fields a and p.

n The values *n* for which the expression was calculated and the execution time was greater than zero.

T_f The nonzero execution times recorded for the expression *f* in seconds.

T_f2 The nonzero execution times recorded for the expression *f2* in seconds. If it is needed, the mean time ratio is just mean(T_f./T_f2).

The slope of the execution time graph shows the approximate power of the asymptotic running time O(n^p). This power is plotted for the region over which it is approximated (the latter half of the graph). The estimated power is not very accurate, but should be sufficient to determine the general order of your algorithm. It should indicate if for example your implementation is unexpectedly O(n^2) rather than O(n) because it extends a vector each time through the loop rather than preallocating one which is big enough. For example, in the current version of Octave, the following is not the expected O(n):

```
speed ("for i = 1:n, y{i} = x(i); end", "", [1000,10000])
```

but it is if you preallocate the cell array y:

```
speed ("for i = 1:n, y{i} = x(i); end", ...
       "x = rand (n, 1); y = cell (size (x));", [1000, 10000])
```

An attempt is made to approximate the cost of the individual operations, but it is wildly inaccurate. You can improve the stability somewhat by doing more work for each n. For example:

```
speed ("airy(x)", "x = rand (n, 10)", [10000, 100000])
```

When comparing a new and original expression, the line on the speedup ratio graph should be larger than 1 if the new expression is faster. Better algorithms have a shallow slope. Generally, vectorizing an algorithm will not change the slope of the execution time graph, but it will shift it relative to the original. For example:

```
speed ("v = sum (x)", "", [10000, 100000], ...
       "v = 0; for i = 1:length (x), v += x(i); end")
```

A more complex example, if you had an original version of xcorr using for loops and another version using an FFT, you could compare the run speed for various lags as follows, or for a fixed lag with varying vector lengths as follows:

```
speed ("v = xcorr (x, n)", "x = rand (128, 1);", 100,
       "v2 = xcorr_orig (x, n)", -100*eps)
speed ("v = xcorr (x, 15)", "x = rand (20+n, 1);", 100,
       "v2 = xcorr_orig (x, n)", -100*eps)
```

Assuming one of the two versions is in *xcorr_orig*, this would compare their speed and their output values. Note that the FFT version is not exact, so we specify an acceptable tolerance on the comparison `100*eps`, and the errors should be computed relatively, as `abs((`*x* `-` *y*`)./`*y*`)` rather than absolutely as `abs(`*x* `-` *y*`)`.

Type `example('speed')` to see some real examples. Note for obscure reasons, you can't run examples 1 and 2 directly using `demo('speed')`. Instead use, `eval(example('speed',1))` and `eval(example('speed',2))`.

Appendix C Tips and Standards

This chapter describes no additional features of Octave. Instead it gives advice on making effective use of the features described in the previous chapters.

C.1 Writing Clean Octave Programs

Here are some tips for avoiding common errors in writing Octave code intended for widespread use:

- Since all global variables share the same name space, and all functions share another name space, you should choose a short word to distinguish your program from other Octave programs. Then take care to begin the names of all global variables, constants, and functions with the chosen prefix. This helps avoid name conflicts.

  If you write a function that you think ought to be added to Octave under a certain name, such as `fiddle_matrix`, don't call it by that name in your program. Call it `mylib_fiddle_matrix` in your program, and send mail to `maintainers@octave.org` suggesting that it be added to Octave. If and when it is, the name can be changed easily enough.

  If one prefix is insufficient, your package may use two or three alternative common prefixes, so long as they make sense.

  Separate the prefix from the rest of the symbol name with an underscore '`_`'. This will be consistent with Octave itself and with most Octave programs.
- When you encounter an error condition, call the function `error` (or `usage`). The `error` and `usage` functions do not return. See Section 2.5 [Errors], page 27.
- Please put a copyright notice on the file if you give copies to anyone. Use the same lines that appear at the top of the function files distributed with Octave. If you have not signed papers to assign the copyright to anyone else, then place your name in the copyright notice.

C.2 Tips for Making Code Run Faster.

Here are some ways of improving the execution speed of Octave programs.

- Vectorize loops. For instance, rather than

  ```
  for i = 1:n-1
    a(i) = b(i+1) - b(i);
  endfor
  ```

  write

  ```
  a = b(2:n) - b(1:n-1);
  ```

  This is especially important for loops with "cheap" bodies. Often it suffices to vectorize just the innermost loop to get acceptable performance. A general rule of thumb is that the "order" of the vectorized body should be greater or equal to the "order" of the enclosing loop.
- Use built-in and library functions if possible. Built-in and compiled functions are very fast. Even with a m-file library function, chances are good that it is already optimized, or will be optimized more in a future release.

- Avoid computing costly intermediate results multiple times. Octave currently does not eliminate common subexpressions.
- Be aware of lazy copies (copy-on-write). When a copy of an object is created, the data is not immediately copied, but rather shared. The actual copying is postponed until the copied data needs to be modified. For example:

  ```
  a = zeros (1000); # create a 1000x1000 matrix
  b = a; # no copying done here
  b(1) = 1; # copying done here
  ```

  Lazy copying applies to whole Octave objects such as matrices, cells, struct, and also individual cell or struct elements (not array elements).

  Additionally, index expressions also use lazy copying when Octave can determine that the indexed portion is contiguous in memory. For example:

  ```
  a = zeros (1000); # create a 1000x1000 matrix
  b = a(:,10:100); # no copying done here
  b = a(10:100,:); # copying done here
  ```

  This applies to arrays (matrices), cell arrays, and structs indexed using (). Index expressions generating cs-lists can also benefit of shallow copying in some cases. In particular, when *a* is a struct array, expressions like `{a.x}`, `{a(:,2).x}` will use lazy copying, so that data can be shared between a struct array and a cell array.

  Most indexing expressions do not live longer than their 'parent' objects. In rare cases, however, a lazily copied slice outlasts its parent, in which case it becomes orphaned, still occupying unnecessarily more memory than needed. To provide a remedy working in most real cases, Octave checks for orphaned lazy slices at certain situations, when a value is stored into a "permanent" location, such as a named variable or cell or struct element, and possibly economizes them. For example

  ```
  a = zeros (1000); # create a 1000x1000 matrix
  b = a(:,10:100); # lazy slice
  a = []; # the original a array is still allocated
  c{1} = b; # b is reallocated at this point
  ```

- Avoid deep recursion. Function calls to m-file functions carry a relatively significant overhead, so rewriting a recursion as a loop often helps. Also, note that the maximum level of recursion is limited.
- Avoid resizing matrices unnecessarily. When building a single result matrix from a series of calculations, set the size of the result matrix first, then insert values into it. Write

  ```
  result = zeros (big_n, big_m)
  for i = over:and_over
    r1 = ...
    r2 = ...
    result (r1, r2) = new_value ();
  endfor
  ```

  instead of

```
result = [];
for i = ever:and_ever
  result = [ result, new_value() ];
endfor
```

Sometimes the number of items can't be computed in advance, and stack-like operations are needed. When elements are being repeatedly inserted at/removed from the end of an array, Octave detects it as stack usage and attempts to use a smarter memory management strategy preallocating the array in bigger chunks. Likewise works for cell and struct arrays.

```
a = [];
while (condition)
  ...
  a(end+1) = value; # "push" operation
  ...
  a(end) = []; # "pop" operation
  ...
endwhile
```

- Use `cellfun` intelligently. The `cellfun` function is a useful tool for avoiding loops. See Section 6.2.5 [Processing Data in Cell Arrays], page 91. `cellfun` is often use with anonymous function handles; however, calling an anonymous function involves an overhead quite comparable to the overhead of an m-file function. Passing a handle to a built-in function is faster, because the interpreter is not involved in the internal loop. For example:

```
a = {...}
v = cellfun (@(x) det(x), a); # compute determinants
v = cellfun (@det, a); # faster
```

- Avoid calling `eval` or `feval` excessively, because they require Octave to parse input or look up the name of a function in the symbol table.

  If you are using `eval` as an exception handling mechanism and not because you need to execute some arbitrary text, use the `try` statement instead. See Section 10.9 [The try Statement], page 134.

- If you are calling lots of functions but none of them will need to change during your run, set the variable `ignore_function_time_stamp` to `"all"` so that Octave doesn't waste a lot of time checking to see if you have updated your function files.

C.3 Tips on Writing Comments

Here are the conventions to follow when writing comments.

'`#`' Comments that start with a single sharp-sign, '`#`', should all be aligned to the same column on the right of the source code. Such comments usually explain how the code on the same line does its job. In the Emacs mode for Octave, the *M-;* (`indent-for-comment`) command automatically inserts such a '`#`' in the right place, or aligns such a comment if it is already present.

'`##`' Comments that start with a double sharp-sign, '`##`', should be aligned to the same level of indentation as the code. Such comments usually describe the purpose of the following lines or the state of the program at that point.

The indentation commands of the Octave mode in Emacs, such as *M-;* (`indent-for-comment`) and *TAB* (`octave-indent-line`) automatically indent comments according to these conventions, depending on the number of semicolons. See Section "Manipulating Comments" in *The GNU Emacs Manual*.

C.4 Conventional Headers for Octave Functions

Octave has conventions for using special comments in function files to give information such as who wrote them. This section explains these conventions.

The top of the file should contain a copyright notice, followed by a block of comments that can be used as the help text for the function. Here is an example:

```
## Copyright (C) 1996, 1997, 2007 John W. Eaton
##
## This file is part of Octave.
##
## Octave is free software; you can redistribute it and/or
## modify it under the terms of the GNU General Public
## License as published by the Free Software Foundation;
## either version 3 of the License, or (at your option) any
## later version.
##
## Octave is distributed in the hope that it will be useful,
## but WITHOUT ANY WARRANTY; without even the implied
## warranty of MERCHANTABILITY or FITNESS FOR A PARTICULAR
## PURPOSE.  See the GNU General Public License for more
## details.
##
## You should have received a copy of the GNU General Public
## License along with Octave; see the file COPYING.  If not,
## see <http://www.gnu.org/licenses/>.

## usage: [IN, OUT, PID] = popen2 (COMMAND, ARGS)
##
## Start a subprocess with two-way communication.  COMMAND
## specifies the name of the command to start.  ARGS is an
## array of strings containing options for COMMAND.  IN and
## OUT are the file ids of the input and streams for the
## subprocess, and PID is the process id of the subprocess,
## or -1 if COMMAND could not be executed.
##
## Example:
##
##  [in, out, pid] = popen2 ("sort", "-nr");
```

```
## fputs (in, "these\nare\nsome\nstrings\n");
## fclose (in);
## while (ischar (s = fgets (out)))
##   fputs (stdout, s);
## endwhile
## fclose (out);
```

Octave uses the first block of comments in a function file that do not appear to be a copyright notice as the help text for the file. For Octave to recognize the first comment block as a copyright notice, it must start with the word 'Copyright' after stripping the leading comment characters.

After the copyright notice and help text come several *header comment* lines, each beginning with '`##` *header-name*`:`'. For example,

```
## Author: jwe
## Keywords: subprocesses input-output
## Maintainer: jwe
```

Here is a table of the conventional possibilities for *header-name*:

'`Author`' This line states the name and net address of at least the principal author of the library.

```
## Author: John W. Eaton <jwe@octave.org>
```

'`Maintainer`'
: This line should contain a single name/address as in the Author line, or an address only, or the string '`jwe`'. If there is no maintainer line, the person(s) in the Author field are presumed to be the maintainers. The example above is mildly bogus because the maintainer line is redundant.

  The idea behind the '`Author`' and '`Maintainer`' lines is to make possible a function to "send mail to the maintainer" without having to mine the name out by hand.

  Be sure to surround the network address with '`<...>`' if you include the person's full name as well as the network address.

'`Created`' This optional line gives the original creation date of the file. For historical interest only.

'`Version`' If you wish to record version numbers for the individual Octave program, put them in this line.

'`Adapted-By`'
: In this header line, place the name of the person who adapted the library for installation (to make it fit the style conventions, for example).

'`Keywords`'
: This line lists keywords. Eventually, it will be used by an apropos command to allow people will find your package when they're looking for things by topic area. To separate the keywords, you can use spaces, commas, or both.

Just about every Octave function ought to have the '`Author`' and '`Keywords`' header comment lines. Use the others if they are appropriate. You can also put in header lines with other header names—they have no standard meanings, so they can't do any harm.

C.5 Tips for Documentation Strings

As noted above, documentation is typically in a commented header block on an Octave function following the copyright statement. The help string shown above is an unformatted string and will be displayed as is by Octave. Here are some tips for the writing of documentation strings.

- Every command, function, or variable intended for users to know about should have a documentation string.
- An internal variable or subroutine of an Octave program might as well have a documentation string.
- The first line of the documentation string should consist of one or two complete sentences that stand on their own as a summary.

  The documentation string can have additional lines that expand on the details of how to use the function or variable. The additional lines should also be made up of complete sentences.
- For consistency, phrase the verb in the first sentence of a documentation string as an infinitive with "to" omitted. For instance, use "Return the frob of A and B." in preference to "Returns the frob of A and B." Usually it looks good to do likewise for the rest of the first paragraph. Subsequent paragraphs usually look better if they have proper subjects.
- Write documentation strings in the active voice, not the passive, and in the present tense, not the future. For instance, use "Return a list containing A and B." instead of "A list containing A and B will be returned."
- Avoid using the word "cause" (or its equivalents) unnecessarily. Instead of, "Cause Octave to display text in boldface," write just "Display text in boldface."
- Do not start or end a documentation string with whitespace.
- Format the documentation string so that it fits in an Emacs window on an 80-column screen. It is a good idea for most lines to be no wider than 60 characters.

  However, rather than simply filling the entire documentation string, you can make it much more readable by choosing line breaks with care. Use blank lines between topics if the documentation string is long.
- **Do not** indent subsequent lines of a documentation string so that the text is lined up in the source code with the text of the first line. This looks nice in the source code, but looks bizarre when users view the documentation. Remember that the indentation before the starting double-quote is not part of the string!
- The documentation string for a variable that is a yes-or-no flag should start with words such as "Nonzero means...", to make it clear that all nonzero values are equivalent and indicate explicitly what zero and nonzero mean.
- When a function's documentation string mentions the value of an argument of the function, use the argument name in capital letters as if it were a name for that value. Thus, the documentation string of the operator `/` refers to its second argument as '`DIVISOR`', because the actual argument name is `divisor`.

  Also use all caps for meta-syntactic variables, such as when you show the decomposition of a list or vector into subunits, some of which may vary.

Octave also allows extensive formatting of the help string of functions using Texinfo. The effect on the online documentation is relatively small, but makes the help string of functions conform to the help of Octave's own functions. However, the effect on the appearance of printed or online documentation will be greatly improved.

The fundamental building block of Texinfo documentation strings is the Texinfo-macro `@deftypefn`, which takes three arguments: The class the function is in, its output arguments, and the function's signature. Typical classes for functions include `Function File` for standard Octave functions, and `Loadable Function` for dynamically linked functions. A skeletal Texinfo documentation string therefore looks like this

```
-*- texinfo -*-
@deftypefn{Function File} {@var{ret} =} fn (...)
@cindex index term
Help text in Texinfo format.  Code samples should be marked
like @code{sample of code} and variables should be marked
as @var{variable}.
@seealso{fn2}
@end deftypefn
```

This help string must be commented in user functions, or in the help string of the `DEFUN_DLD` macro for dynamically loadable functions. The important aspects of the documentation string are

-*- texinfo -*-
: This string signals Octave that the following text is in Texinfo format, and should be the first part of any help string in Texinfo format.

@deftypefn{class} . . . @end deftypefn
: The entire help string should be enclosed within the block defined by deftypefn.

@cindex index term
: This generates an index entry, and can be useful when the function is included as part of a larger piece of documentation. It is ignored within Octave's help viewer. Only one index term may appear per line but multiple @cindex lines are valid if the function should be filed under different terms.

@var{variable}
: All variables should be marked with this macro. The markup of variables is then changed appropriately for display.

@code{sample of code}
: All samples of code should be marked with this macro for the same reasons as the @var macro.

@seealso{function2}
: This is a comma separated list of function names that allows cross referencing from one function documentation string to another.

Texinfo format has been designed to generate output for online viewing with text terminals as well as generating high-quality printed output. To these ends, Texinfo has commands which control the diversion of parts of the document into a particular output processor. Three formats are of importance: info, html and TeX. These are selected with

```
@ifinfo
Text area for info only
@end ifinfo

@ifhtml
Text area for HTML only
@end ifhtml

@tex
Text area for TeX only
@end tex
```

Note that often TeX output can be used in html documents and so often the `@ifhtml` blocks are unnecessary. If no specific output processor is chosen, by default, the text goes into all output processors. It is usual to have the above blocks in pairs to allow the same information to be conveyed in all output formats, but with a different markup. Currently, most Octave documentation only makes a distinction between TeX and all other formats. Therefore, the following construct is seen repeatedly.

```
@tex
text for TeX only
@end tex
@ifnottex
text for info, HTML, plaintext
@end ifnottex
```

Another important feature of Texinfo that is often used in Octave help strings is the `@example` environment. An example of its use is

```
@example
@group
@code{2 * 2}
@result{} 4
@end group
@end example
```

which produces

```
2 * 2
⇒ 4
```

The `@group` block prevents the example from being split across a page boundary, while the `@result{}` macro produces a right arrow signifying the result of a command. If your example is larger than 20 lines it is better NOT to use grouping so that a reasonable page boundary can be calculated.

In many cases a function has multiple ways in which it can be called, and the `@deftypefnx` macro can be used to give alternatives. For example

```
-*- texinfo -*-
@deftypefn{Function File} {@var{a} =} fn (@var{x}, ...)
@deftypefnx{Function File} {@var{a} =} fn (@var{y}, ...)
Help text in Texinfo format.
@end deftypefn
```

Many complete examples of Texinfo documentation can be taken from the help strings for the Octave functions themselves. A relatively complete example of which is the `nchoosek` function. The Texinfo documentation string for `nchoosek` is

```
-*- texinfo -*-
@deftypefn {Function File} {} nchoosek (@var{n}, @var{k})

Compute the binomial coefficient or all combinations of
@var{n}.  If @var{n} is a scalar then, calculate the
binomial coefficient of @var{n} and @var{k}, defined as

@tex
$$
 {n \choose k} = {n (n-1) (n-2) \cdots (n-k+1) \over k!}
$$
@end tex
@ifnottex

@example
@group
 /   \
 | n |    n (n-1) (n-2) ... (n-k+1)
 |   |  = -------------------------
 | k |               k!
 \   /
@end group
@end example
@end ifnottex

If @var{n} is a vector, this generates all combinations
of the elements of @var{n}, taken @var{k} at a time,
one row per combination.  The resulting @var{c} has size
@code{[nchoosek (length (@var{n}),@var{k}), @var{k}]}.

@code{nchoosek} works only for non-negative integer arguments; use
@code{bincoeff} for non-integer scalar arguments and for using vector
arguments to compute many coefficients at once.

@seealso{bincoeff}
@end deftypefn
```

which demonstrates most of the concepts discussed above. This documentation string renders as

```
 -- Function File: C = nchoosek (N, K)
     Compute the binomial coefficient or all combinations
     of N.  If N is a scalar then, calculate the binomial
     coefficient of N and K, defined as

           /   \
           | n |    n (n-1) (n-2) ... (n-k+1)       n!
           |   |  = ------------------------- =  ---------
           | k |               k!                k! (n-k)!
           \   /

     If N is a vector generate all combinations of the
     elements of N, taken K at a time, one row per
     combination.  The resulting C has size `[nchoosek
     (length (N), K), K]'.

     `nchoosek' works only for non-negative integer
     arguments; use `bincoeff' for non-integer scalar
     arguments and for using vector arguments to compute
     many coefficients at once.

     See also: bincoeff.
```

using info, whereas in a printed documentation using TEX it will appear as

c = `nchoosek` (*n*, *k*) [Function File]

Compute the binomial coefficient or all combinations of *n*. If *n* is a scalar then, calculate the binomial coefficient of *n* and *k*, defined as

$$\binom{n}{k} = \frac{n(n-1)(n-2)\cdots(n-k+1)}{k!}$$

If *n* is a vector generate all combinations of the elements of *n*, taken *k* at a time, one row per combination. The resulting *c* has size `[nchoosek (length (n), k), k]`.

`nchoosek` works only for non-negative integer arguments; use `bincoeff` for non-integer scalar arguments and for using vector arguments to compute many coefficients at once.

See also: bincoeff.

Appendix D Contributing Guidelines

This chapter is dedicated to those who wish to contribute code to Octave.

D.1 How to Contribute

The mailing list for Octave development discussion and sending contributions is maintainers@octave.org. This concerns the development of Octave core, i.e., code that goes to Octave directly. You may consider developing and publishing a package instead; a great place for this is the allied Octave-Forge project (http://octave.sf.net). Note that the Octave project is inherently more conservative and follows narrower rules.

The preferable form of contribution is creating a Mercurial changeset and sending it via e-mail to the octave-maintainers mailing list. Mercurial is the source code management system currently used to develop Octave. Other forms of contributions (e.g., simple diff patches) are also acceptable, but they slow down the review process. If you want to make more contributions, you should really get familiar with Mercurial. A good place to start is http://www.selenic.com/mercurial/wiki/index.cgi/Tutorial. There you will also find help how to install Mercurial.

A simple contribution sequence could look like this:

```
hg clone http://www.octave.org/hg/octave
                             # make a local copy of the octave
                             # source repository
cd octave
# change some sources...
hg commit -m "make Octave the coolest software ever"
                             # commit the changeset into your
                             # local repository
hg export -o ../cool.diff tip
                             # export the changeset to a diff
                             # file
# send ../cool.diff via email
```

You may want to get familiar with Mercurial queues to manage your changesets. Here is a slightly less simple example using Mercurial queues, where you work on two unrelated changesets in parallel and update one of the changesets after discussion in the maintainers mailing list:

```
hg qnew nasty_bug            # create a new patch
# change sources...
hg qref                      # save the changes into the patch
# change even more...
hg qref -m "solution to nasty bug!"
                             # save again with commit message
hg export -o ../nasty.diff tip
                             # export the patch
# send ../nasty.diff via email
hg qpop                      # undo the application of the patch
                             # and remove the changes from the
```

```
                              # source tree
hg qnew doc_improvements     # create an unrelated patch
# change doc sources...
hg qref -m "could not find myfav.m in the doc"
                              # save the changes into the patch
hg export -o ../doc.diff tip
                              # export the second patch
# send ../doc.diff tip via email
hg qpop
# discussion in the maintainers mailing list ...
hg qpush nasty_bug           # apply the patch again
# change sources yet again ...
hg qref
hg export -o ../nasty2.diff tip
# send ../nasty2.diff via email
```

D.2 General Guidelines

All Octave's sources are distributed under the General Public License (GPL). Currently, Octave uses GPL version 3. For details about this license, see http://www.gnu.org/licenses/gpl.html. Therefore, whenever you create a new source file, it should have the following comment header (use appropriate year, name and comment marks):

```
## Copyright (C) 1996, 1997, 2007 John W. Eaton <jwe@octave.org>
##
## This file is part of Octave.
##
## Octave is free software; you can redistribute it and/or
## modify it under the terms of the GNU General Public
## License as published by the Free Software Foundation;
## either version 3 of the License, or (at your option) any
## later version.
##
## Octave is distributed in the hope that it will be useful,
## but WITHOUT ANY WARRANTY; without even the implied
## warranty of MERCHANTABILITY or FITNESS FOR A PARTICULAR
## PURPOSE.  See the GNU General Public License for more
## details.
##
## You should have received a copy of the GNU General Public
## License along with Octave; see the file COPYING.  If not,
## see <http://www.gnu.org/licenses/>.
```

Always include ChangeLog entries in changesets. After making your source changes, record and briefly describe the changes in the nearest ChangeLog file upwards in the directory tree. Use the previous entries as a template. Your entry should contain your name and email, and the path to the modified source file relative to the parent directory of the

ChangeLog file. If there are more functions in the file, you should also include the name of the modified function (in parentheses after file path). Example:

```
2008-04-02  David Bateman  <dbateman@free.fr>

        * graphics.cc (void gnuplot_backend::close_figure (const
        octave_value&) const): Allow for an input and output stream.
```

The ChangeLog entries should describe what is changed, not why. Any explanation of why a change is needed should appear as comments in the code, particularly if there is something that might not be obvious to someone reading it later.

The preferred comment mark for places that may need further attention is FIXME.

D.3 Octave Sources (m-files)

Don't use tabs. Tabs cause trouble. If you are used to them, set up your editor so that it converts tabs to spaces. Indent the bodies of the statement blocks. Recommended indent is 2 spaces. When calling functions, put spaces after commas and before the calling parentheses, like this:

```
x = max (sin (y+3), 2);
```

An exception are matrix and vector constructors:

```
[sin(x), cos(x)]
```

Here, putting spaces after `sin`, `cos` would result in a parse error. In indexing expression, do not put a space after the identifier (this differentiates indexing and function calls nicely). The space after comma is not necessary if index expressions are simple, i.e., you may write

```
A(:,i,j)
```

but

```
A([1:i-1;i+1:n], XI(:,2:n-1))
```

Use lowercase names if possible. Uppercase is acceptable for variable names consisting of 1-2 letters. Do not use mixed case names. Function names must be lowercase. Function names are global, so choose them wisely.

Always use a specific end-of-block statement (like `endif`, `endswitch`) rather than generic `end`. Enclose the `if`, `while`, `until` and `switch` conditions in parentheses, like in C:

```
if (isvector (a))
  s = sum(a);
endif
```

Do not do this, however, with `for`:

```
for i = 1:n
  b(i) = sum (a(:,i));
endfor
```

D.4 C++ Sources

Don't use tabs. Tabs cause trouble. If you are used to them, set up your editor so that it converts tabs to spaces. Format function headers like this:

```
static bool
matches_patterns (const string_vector& patterns, int pat_idx,
  int num_pat, const std::string& name)
```

The function name should start in column 1, and multi-line argument lists should be aligned on the first char after the open parenthesis. You should put a space after the left open parenthesis and after commas, for both function definitions and function calls.

Recommended indent is 2 spaces. When indenting, indent the statement after control structures (like `if`, `while`, etc.). If there is a compound statement, indent *both* the curly braces and the body of the statement (so that the body gets indented by *two* indents). Example:

```
if (have_args)
  {
    idx.push_back (first_args);
    have_args = false;
  }
else
  idx.push_back (make_value_list (*p_args, *p_arg_nm, &tmp));
```

If you have nested `if` statements, use extra braces for extra clarification.

Split long expressions in such a way that a continuation line starts with an operator rather than identifier. If the split occurs inside braces, continuation should be aligned with the first char after the innermost braces enclosing the split. Example:

```
SVD::type type = ((nargout == 0 || nargout == 1)
                  ? SVD::sigma_only
                  : (nargin == 2) ? SVD::economy : SVD::std);
```

Consider putting extra braces around a multiline expression to make it more readable, even if they are not necessary. Also, do not hesitate to put extra braces anywhere if it improves clarity.

Try declaring variables just before they're needed. Use local variables of blocks - it helps optimization. Don't write multi-line variable declaration with a single type specification and multiple variables. If the variables don't fit on single line, repeat the type specification. Example:

```
octave_value retval;

octave_idx_type nr = b.rows ();
octave_idx_type nc = b.cols ();

double d1, d2;
```

Use lowercase names if possible. Uppercase is acceptable for variable names consisting of 1-2 letters. Do not use mixed case names.

Try to use Octave's types and classes if possible. Otherwise, try to use C++ standard library. Use of STL containers and algorithms is encouraged. Use templates wisely to reduce code duplication. Avoid comma expressions, labels and gotos, and explicit typecasts. If you need to typecast, use the modern C++ casting operators. In functions, try to reduce the number of `return` statements - use nested `if` statements if possible.

D.5 Other Sources

Apart from C++ and Octave language (m-files), Octave's sources include files written in C, Fortran, M4, perl, unix shell, AWK, texinfo and TeX. There are not many rules to follow when using these other languages; some of them are summarized below. In any case, the golden rule is: if you modify a source file, try to follow any conventions you can detect in the file or other similar files.

For C you should obviously follow all C++ rules that can apply.

If you happen to modify a Fortran file, you should stay within Fortran 77 with common extensions like `END DO`. Currently, we want all sources to be compilable with the f2c and g77 compilers, without special flags if possible. This usually means that non-legacy compilers also accept the sources.

The M4 macro language is mainly used for autoconf configuration files. You should follow normal M4 rules when contributing to these files. Some M4 files come from external source, namely the Autoconf archive `http://autoconf-archive.cryp.to`.

If you give a code example in the documentation written in texinfo with the `@example` environment, you should be aware that the text within such an environment will not be wrapped. It is recommended that you keep the lines short enough to fit on pages in the generated pdf or ps documents. Here is a ruler (in an `@example` environment) for finding the appropriate line width:

```
         1         2         3         4         5         6
123456789012345678901234567890123456789012345678901234567890
```

Appendix E Known Causes of Trouble

This section describes known problems that affect users of Octave. Most of these are not Octave bugs per se—if they were, we would fix them. But the result for a user may be like the result of a bug.

Some of these problems are due to bugs in other software, some are missing features that are too much work to add, and some are places where people's opinions differ as to what is best.

E.1 Actual Bugs We Haven't Fixed Yet

- Output that comes directly from Fortran functions is not sent through the pager and may appear out of sequence with other output that is sent through the pager. One way to avoid this is to force pending output to be flushed before calling a function that will produce output from within Fortran functions. To do this, use the command

  ```
  fflush (stdout)
  ```

  Another possible workaround is to use the command

  ```
  page_screen_output (false);
  ```

  to turn the pager off.

A list of ideas for future enhancements is distributed with Octave. See the file '`PROJECTS`' in the top level directory in the source distribution.

E.2 Reporting Bugs

Your bug reports play an essential role in making Octave reliable.

When you encounter a problem, the first thing to do is to see if it is already known. See Appendix E [Trouble], page 621. If it isn't known, then you should report the problem.

Reporting a bug may help you by bringing a solution to your problem, or it may not. In any case, the principal function of a bug report is to help the entire community by making the next version of Octave work better. Bug reports are your contribution to the maintenance of Octave.

In order for a bug report to serve its purpose, you must include the information that makes it possible to fix the bug.

If you have Octave working at all, the easiest way to prepare a complete bug report is to use the Octave function `bug_report`. When you execute this function, Octave will prompt you for a subject and then invoke the editor on a file that already contains all the configuration information. When you exit the editor, Octave will mail the bug report for you.

`bug_report ()` [Function File]

Have Octave create a bug report template file, invoke your favorite editor, and submit the report to the bug-octave mailing list when you are finished editing.

E.3 Have You Found a Bug?

If you are not sure whether you have found a bug, here are some guidelines:

- If Octave gets a fatal signal, for any input whatever, that is a bug. Reliable interpreters never crash.
- If Octave produces incorrect results, for any input whatever, that is a bug.
- Some output may appear to be incorrect when it is in fact due to a program whose behavior is undefined, which happened by chance to give the desired results on another system. For example, the range operator may produce different results because of differences in the way floating point arithmetic is handled on various systems.
- If Octave produces an error message for valid input, that is a bug.
- If Octave does not produce an error message for invalid input, that is a bug. However, you should note that your idea of "invalid input" might be my idea of "an extension" or "support for traditional practice".
- If you are an experienced user of programs like Octave, your suggestions for improvement are welcome in any case.

E.4 Where to Report Bugs

If you have Octave working at all, the easiest way to prepare a complete bug report is to use the Octave function `bug_report`. When you execute this function, Octave will prompt you for a subject and then invoke the editor on a file that already contains all the configuration information. When you exit the editor, Octave will mail the bug report for you.

If for some reason you cannot use Octave's `bug_report` function, send bug reports for Octave to `bug@octave.org`.

Do not send bug reports to '`help-octave`'. Most users of Octave do not want to receive bug reports. Those that do have asked to be on the mailing list.

E.5 How to Report Bugs

Send bug reports for Octave to one of the addresses listed in Section E.4 [Bug Lists], page 622.

The fundamental principle of reporting bugs usefully is this: **report all the facts**. If you are not sure whether to state a fact or leave it out, state it!

Often people omit facts because they think they know what causes the problem and they conclude that some details don't matter. Thus, you might assume that the name of the variable you use in an example does not matter. Well, probably it doesn't, but one cannot be sure. Perhaps the bug is a stray memory reference which happens to fetch from the location where that name is stored in memory; perhaps, if the name were different, the contents of that location would fool the interpreter into doing the right thing despite the bug. Play it safe and give a specific, complete example.

Keep in mind that the purpose of a bug report is to enable someone to fix the bug if it is not known. Always write your bug reports on the assumption that the bug is not known.

Sometimes people give a few sketchy facts and ask, "Does this ring a bell?" This cannot help us fix a bug. It is better to send a complete bug report to begin with.

Try to make your bug report self-contained. If we have to ask you for more information, it is best if you include all the previous information in your response, as well as the information that was missing.

To enable someone to investigate the bug, you should include all these things:

- The version of Octave. You can get this by noting the version number that is printed when Octave starts, or running it with the '`-v`' option.
- A complete input file that will reproduce the bug.

  A single statement may not be enough of an example—the bug might depend on other details that are missing from the single statement where the error finally occurs.
- The command arguments you gave Octave to execute that example and observe the bug. To guarantee you won't omit something important, list all the options.

  If we were to try to guess the arguments, we would probably guess wrong and then we would not encounter the bug.
- The type of machine you are using, and the operating system name and version number.
- The command-line arguments you gave to the `configure` command when you installed the interpreter.
- A complete list of any modifications you have made to the interpreter source.

  Be precise about these changes—show a context diff for them.
- Details of any other deviations from the standard procedure for installing Octave.
- A description of what behavior you observe that you believe is incorrect. For example, "The interpreter gets a fatal signal," or, "The output produced at line 208 is incorrect."

  Of course, if the bug is that the interpreter gets a fatal signal, then one can't miss it. But if the bug is incorrect output, we might not notice unless it is glaringly wrong.

  Even if the problem you experience is a fatal signal, you should still say so explicitly. Suppose something strange is going on, such as, your copy of the interpreter is out of synch, or you have encountered a bug in the C library on your system. Your copy might crash and the copy here would not. If you said to expect a crash, then when the interpreter here fails to crash, we would know that the bug was not happening. If you don't say to expect a crash, then we would not know whether the bug was happening. We would not be able to draw any conclusion from our observations.

  Often the observed symptom is incorrect output when your program is run. Unfortunately, this is not enough information unless the program is short and simple. It is very helpful if you can include an explanation of the expected output, and why the actual output is incorrect.
- If you wish to suggest changes to the Octave source, send them as context diffs. If you even discuss something in the Octave source, refer to it by context, not by line number, because the line numbers in the development sources probably won't match those in your sources.

Here are some things that are not necessary:

- A description of the envelope of the bug.

  Often people who encounter a bug spend a lot of time investigating which changes to the input file will make the bug go away and which changes will not affect it. Such

information is usually not necessary to enable us to fix bugs in Octave, but if you can find a simpler example to report *instead* of the original one, that is a convenience. Errors in the output will be easier to spot, running under the debugger will take less time, etc. Most Octave bugs involve just one function, so the most straightforward way to simplify an example is to delete all the function definitions except the one in which the bug occurs.

However, simplification is not vital; if you don't want to do this, report the bug anyway and send the entire test case you used.

- A patch for the bug. Patches can be helpful, but if you find a bug, you should report it, even if you cannot send a fix for the problem.

E.6 Sending Patches for Octave

If you would like to write bug fixes or improvements for Octave, that is very helpful. When you send your changes, please follow these guidelines to avoid causing extra work for us in studying the patches.

If you don't follow these guidelines, your information might still be useful, but using it will take extra work. Maintaining Octave is a lot of work in the best of circumstances, and we can't keep up unless you do your best to help.

- Send an explanation with your changes of what problem they fix or what improvement they bring about. For a bug fix, just include a copy of the bug report, and explain why the change fixes the bug.
- Always include a proper bug report for the problem you think you have fixed. We need to convince ourselves that the change is right before installing it. Even if it is right, we might have trouble judging it if we don't have a way to reproduce the problem.
- Include all the comments that are appropriate to help people reading the source in the future understand why this change was needed.
- Don't mix together changes made for different reasons. Send them *individually*.

  If you make two changes for separate reasons, then we might not want to install them both. We might want to install just one.
- Use '`diff -c`' to make your diffs. Diffs without context are hard for us to install reliably. More than that, they make it hard for us to study the diffs to decide whether we want to install them. Unidiff format is better than contextless diffs, but not as easy to read as '`-c`' format.

  If you have GNU diff, use '`diff -cp`', which shows the name of the function that each change occurs in.
- Write the change log entries for your changes.

  Read the '`ChangeLog`' file to see what sorts of information to put in, and to learn the style that we use. The purpose of the change log is to show people where to find what was changed. So you need to be specific about what functions you changed; in large functions, it's often helpful to indicate where within the function the change was made.

  On the other hand, once you have shown people where to find the change, you need not explain its purpose. Thus, if you add a new function, all you need to say about it is that it is new. If you feel that the purpose needs explaining, it probably does—but the explanation will be much more useful if you put it in comments in the code.

If you would like your name to appear in the header line for who made the change, send us the header line.

E.7 How To Get Help with Octave

The mailing list help@octave.org exists for the discussion of matters related to using and installing Octave. If would like to join the discussion, please send a short note to help-request@octave.org.

Please do not send requests to be added or removed from the mailing list, or other administrative trivia to the list itself.

If you think you have found a bug in the installation procedure, however, you should send a complete bug report for the problem to bug@octave.org. See Section E.5 [Bug Reporting], page 622, for information that will help you to submit a useful report.

Appendix F Installing Octave

Here is the procedure for installing Octave from scratch on a Unix system.

- Run the shell script '`configure`'. This will determine the features your system has (or doesn't have) and create a file named '`Makefile`' from each of the files named '`Makefile.in`'.

  Here is a summary of the configure options that are most frequently used when building Octave:

  `--prefix=`*`prefix`*
  : Install Octave in subdirectories below *prefix*. The default value of *prefix* is '`/usr/local`'.

  `--srcdir=`*`dir`*
  : Look for Octave sources in the directory *dir*.

  `--enable-bounds-check`
  : Enable bounds checking for indexing operators in the internal array classes. This option is primarily used for debugging Octave. Building Octave with this option has a negative impact on performance and is not recommended for general use.

  `--enable-64`
  : This is an **experimental** option to enable Octave to use 64-bit integers for array dimensions and indexing on 64-bit platforms. You probably don't want to use this option unless you know what you are doing.

    If you use `--enable-64`, you must ensure that your Fortran compiler generates code with 8 byte signed `INTEGER` values, and that your BLAS and LAPACK libraries are compiled to use 8 byte signed integers for array dimensions and indexing.

  `--enable-shared`
  : Create shared libraries (this is the default). If you are planning to use the dynamic loading features, you will probably want to use this option. It will make your '`.oct`' files much smaller and on some systems it may be necessary to build shared libraries in order to use dynamically linked functions.

    You may also want to build a shared version of `libstdc++`, if your system doesn't already have one.

  `--enable-dl`
  : Use `dlopen` and friends to make Octave capable of dynamically linking externally compiled functions (this is the default if `--enable-shared` is specified). This option only works on systems that actually have these functions. If you plan on using this feature, you should probably also use `--enable-shared` to reduce the size of your '`.oct`' files.

  `--without-blas`
  : Compile and use the generic BLAS and LAPACK versions included with Octave. By default, configure first looks for BLAS and LAPACK matrix libraries

on your system, including optimized BLAS implementations such as the free ATLAS 3.0, as well as vendor-tuned libraries. (The use of an optimized BLAS will generally result in several-times faster matrix operations.) Only use this option if your system has BLAS/LAPACK libraries that cause problems for some reason. You can also use `--with-blas=lib` to specify a particular BLAS library that configure doesn't check for automatically.

`--without-ccolamd`
: Don't use CCOLAMD, disable some sparse matrix functionality.

`--without-colamd`
: Don't use COLAMD, disable some sparse matrix functionality.

`--without-curl`
: Don't use the cURL, disable the `urlread` and `urlwrite` functions.

`--without-cxsparse`
: Don't use CXSPARSE, disable some sparse matrix functionality.

`--without-umfpack`
: Don't use UMFPACK, disable some sparse matrix functionality.

`--without-fftw`
: Use the included FFTPACK library instead of the FFTW library.

`--without-glpk`
: Don't use the GLPK library for linear programming.

`--without-hdf5`
: Don't use the HDF5 library for reading and writing HDF5 files.

`--without-zlib`
: Don't use the zlib library, disable data file compression and support for recent MAT file formats.

`--without-lapack`
: Compile and use the generic BLAS and LAPACK versions included with Octave. By default, configure first looks for BLAS and LAPACK matrix libraries on your system, including optimized BLAS implementations such as the free ATLAS 3.0, as well as vendor-tuned libraries. (The use of an optimized BLAS will generally result in several-times faster matrix operations.) Only use this option if your system has BLAS/LAPACK libraries that cause problems for some reason. You can also use `--with-blas=lib` to specify a particular BLAS library that configure doesn't check for automatically.

`--without-framework-carbon`
: Don't use framework Carbon headers, libraries and specific source code for compilation even if the configure test succeeds (the default value is `--with-framework-carbon`). This is a platform specific configure option for Mac systems.

`--without-framework-opengl`
: Don't use framework OpenGL headers, libraries and specific source code for compilation even if the configure test succeeds. If this option is given then

OpenGL headers and libraries in standard system locations are tested (the default value is `--with-framework-opengl`). This is a platform specific configure option for Mac systems.

`--help` Print a summary of the options recognized by the configure script.

See the file '`INSTALL`' for more general information about the command line options used by configure. That file also contains instructions for compiling in a directory other than where the source is located.

- Run make.

  You will need a recent version of GNU Make. Modifying Octave's makefiles to work with other make programs is probably not worth your time. We recommend you get and compile GNU Make instead.

  For plotting, you will need to have gnuplot installed on your system. Gnuplot is a command-driven interactive function plotting program. Gnuplot is copyrighted, but freely distributable. The 'gnu' in gnuplot is a coincidence—it is not related to the GNU project or the FSF in any but the most peripheral sense.

  To compile Octave, you will need a recent version of GNU Make. You will also need a recent version of `g++` or other ANSI C++ compiler. You will also need a Fortran 77 compiler or `f2c`. If you use `f2c`, you will need a script like `fort77` that works like a normal Fortran compiler by combining `f2c` with your C compiler in a single script.

  If you plan to modify the parser you will also need GNU `bison` and `flex`. If you modify the documentation, you will need GNU Texinfo, along with the patch for the `makeinfo` program that is distributed with Octave.

  GNU Make, `gcc`, and `libstdc++`, `gnuplot`, `bison`, `flex`, and Texinfo are all available from many anonymous ftp archives. The primary site is `ftp.gnu.org`, but it is often very busy. A list of sites that mirror the software on `ftp.gnu.org` is available by anonymous ftp from `ftp://ftp.gnu.org/pub/gnu/GNUinfo/FTP`.

  You will need about 1 gigabyte of disk storage to work with when building Octave from source (considerably less if you don't compile with debugging symbols). To do that, use the command

  ```
  make CFLAGS=-O CXXFLAGS=-O LDFLAGS=
  ```

  instead of just '`make`'.

- If you encounter errors while compiling Octave, first check the list of known problems below to see if there is a workaround or solution for your problem. If not, see Appendix E [Trouble], page 621, for information about how to report bugs.

- Once you have successfully compiled Octave, run '`make install`'.

  This will install a copy of Octave, its libraries, and its documentation in the destination directory. As distributed, Octave is installed in the following directories. In the table below, *prefix* defaults to '`/usr/local`', *version* stands for the current version number of the interpreter, and *arch* is the type of computer on which Octave is installed (for example, '`i586-unknown-gnu`').

  '*prefix*`/bin`'

  Octave and other binaries that people will want to run directly.

'*prefix*/lib'
: Libraries like libcruft.a and liboctave.a.

'*prefix*/share'
: Architecture-independent data files.

'*prefix*/include/octave'
: Include files distributed with Octave.

'*prefix*/man/man1'
: Unix-style man pages describing Octave.

'*prefix*/info'
: Info files describing Octave.

'*prefix*/share/octave/*version*/m'
: Function files distributed with Octave. This includes the Octave version, so that multiple versions of Octave may be installed at the same time.

'*prefix*/lib/octave/*version*/exec/*arch*'
: Executables to be run by Octave rather than the user.

'*prefix*/lib/octave/*version*/oct/*arch*'
: Object files that will be dynamically loaded.

'*prefix*/share/octave/*version*/imagelib'
: Image files that are distributed with Octave.

F.1 Installation Problems

This section contains a list of problems (and some apparent problems that don't really mean anything is wrong) that may show up during installation of Octave.

- On some SCO systems, `info` fails to compile if `HAVE_TERMIOS_H` is defined in '`config.h`'. Simply removing the definition from '`info/config.h`' should allow it to compile.
- If `configure` finds `dlopen`, `dlsym`, `dlclose`, and `dlerror`, but not the header file '`dlfcn.h`', you need to find the source for the header file and install it in the directory '`usr/include`'. This is reportedly a problem with Slackware 3.1. For Linux/GNU systems, the source for '`dlfcn.h`' is in the `ldso` package.
- Building '`.oct`' files doesn't work.

  You should probably have a shared version of `libstdc++`. A patch is needed to build shared versions of version 2.7.2 of `libstdc++` on the HP-PA architecture. You can find the patch at ftp://ftp.cygnus.com/pub/g++/libg++-2.7.2-hppa-gcc-fix.
- On some alpha systems there may be a problem with the `libdxml` library, resulting in floating point errors and/or segmentation faults in the linear algebra routines called by Octave. If you encounter such problems, then you should modify the configure script so that `SPECIAL_MATH_LIB` is not set to `-ldxml`.
- On FreeBSD systems Octave may hang while initializing some internal constants. The fix appears to be to use

  ```
  options       GPL_MATH_EMULATE
  ```

  rather than

```
options      MATH_EMULATE
```

in the kernel configuration files (typically found in the directory '/sys/i386/conf'. After making this change, you'll need to rebuild the kernel, install it, and reboot.

- If you encounter errors like

  ```
  passing 'void (*)()' as argument 2 of
    'octave_set_signal_handler(int, void (*)(int))'
  ```

  or

  ```
  warning: ANSI C++ prohibits conversion from '(int)'
           to '(...)'
  ```

  while compiling 'sighandlers.cc', you may need to edit some files in the gcc include subdirectory to add proper prototypes for functions there. For example, Ultrix 4.2 needs proper declarations for the signal function and the SIG_IGN macro in the file 'signal.h'.

  On some systems the SIG_IGN macro is defined to be something like this:

  ```
  #define  SIG_IGN  (void (*)())1
  ```

  when it should really be something like:

  ```
  #define  SIG_IGN  (void (*)(int))1
  ```

  to match the prototype declaration for the signal function. This change should also be made for the SIG_DFL and SIG_ERR symbols. It may be necessary to change the definitions in 'sys/signal.h' as well.

  The gcc fixincludes and fixproto scripts should probably fix these problems when gcc installs its modified set of header files, but I don't think that's been done yet.

  You should not change the files in '/usr/include'. You can find the gcc include directory tree by running the command

  ```
  gcc -print-libgcc-file-name
  ```

  The directory of gcc include files normally begins in the same directory that contains the file 'libgcc.a'.

- Some of the Fortran subroutines may fail to compile with older versions of the Sun Fortran compiler. If you get errors like

  ```
  zgemm.f:
  zgemm:
  warning: unexpected parent of complex expression subtree
  zgemm.f, line 245: warning: unexpected parent of complex
    expression subtree
  warning: unexpected parent of complex expression subtree
  zgemm.f, line 304: warning: unexpected parent of complex
    expression subtree
  warning: unexpected parent of complex expression subtree
  zgemm.f, line 327: warning: unexpected parent of complex
    expression subtree
  pcc_binval: missing IR_CONV in complex op
  make[2]: *** [zgemm.o] Error 1
  ```

  when compiling the Fortran subroutines in the 'libcruft' subdirectory, you should either upgrade your compiler or try compiling with optimization turned off.

- On NeXT systems, if you get errors like this:

```
/usr/tmp/cc007458.s:unknown:Undefined local
      symbol LBB7656
/usr/tmp/cc007458.s:unknown:Undefined local
      symbol LBE7656
```

  when compiling 'Array.cc' and 'Matrix.cc', try recompiling these files without -g.
- Some people have reported that calls to shell_cmd and the pager do not work on SunOS systems. This is apparently due to having G_HAVE_SYS_WAIT defined to be 0 instead of 1 when compiling libg++.
- On NeXT systems, linking to 'libsys_s.a' may fail to resolve the following functions

```
_tcgetattr
_tcsetattr
_tcflow
```

  which are part of 'libposix.a'. Unfortunately, linking Octave with -posix results in the following undefined symbols.

```
.destructors_used
.constructors_used
_objc_msgSend
_NXGetDefaultValue
_NXRegisterDefaults
.objc_class_name_NXStringTable
.objc_class_name_NXBundle
```

  One kluge around this problem is to extract 'termios.o' from 'libposix.a', put it in Octave's 'src' directory, and add it to the list of files to link together in the makefile. Suggestions for better ways to solve this problem are welcome!
- If Octave crashes immediately with a floating point exception, it is likely that it is failing to initialize the IEEE floating point values for infinity and NaN.

  If your system actually does support IEEE arithmetic, you should be able to fix this problem by modifying the function octave_ieee_init in the file 'lo-ieee.cc' to correctly initialize Octave's internal infinity and NaN variables.

  If your system does not support IEEE arithmetic but Octave's configure script incorrectly determined that it does, you can work around the problem by editing the file 'config.h' to not define HAVE_ISINF, HAVE_FINITE, and HAVE_ISNAN.

  In any case, please report this as a bug since it might be possible to modify Octave's configuration script to automatically determine the proper thing to do.
- If Octave is unable to find a header file because it is installed in a location that is not normally searched by the compiler, you can add the directory to the include search path by specifying (for example) CPPFLAGS=-I/some/nonstandard/directory as an argument to configure. Other variables that can be specified this way are CFLAGS, CXXFLAGS, FFLAGS, and LDFLAGS. Passing them as options to the configure script also records them in the 'config.status' file. By default, CPPFLAGS and LDFLAGS are empty, CFLAGS and CXXFLAGS are set to "-g -O" and FFLAGS is set to "-O".

Appendix G Emacs Octave Support

The development of Octave code can greatly be facilitated using Emacs with Octave mode, a major mode for editing Octave files which can e.g. automatically indent the code, do some of the typing (with Abbrev mode) and show keywords, comments, strings, etc. in different faces (with Font-lock mode on devices that support it).

It is also possible to run Octave from within Emacs, either by directly entering commands at the prompt in a buffer in Inferior Octave mode, or by interacting with Octave from within a file with Octave code. This is useful in particular for debugging Octave code.

Finally, you can convince Octave to use the Emacs info reader for `help -i`.

All functionality is provided by the Emacs Lisp package EOS (for "Emacs Octave Support"). This chapter describes how to set up and use this package.

Please contact <Kurt.Hornik@wu-wien.ac.at> if you have any questions or suggestions on using EOS.

G.1 Installing EOS

The Emacs package EOS consists of the three files 'octave-mod.el', 'octave-inf.el', and 'octave-hlp.el'. These files, or better yet their byte-compiled versions, should be somewhere in your Emacs load-path.

If you have GNU Emacs with a version number at least as high as 19.35, you are all set up, because EOS is respectively will be part of GNU Emacs as of version 19.35.

Otherwise, copy the three files from the 'emacs' subdirectory of the Octave distribution to a place where Emacs can find them (this depends on how your Emacs was installed). Byte-compile them for speed if you want.

G.2 Using Octave Mode

If you are lucky, your sysadmins have already arranged everything so that Emacs automatically goes into Octave mode whenever you visit an Octave code file as characterized by its extension '.m'. If not, proceed as follows.

1. To begin using Octave mode for all '.m' files you visit, add the following lines to a file loaded by Emacs at startup time, typically your '~/.emacs' file:

```
(autoload 'octave-mode "octave-mod" nil t)
(setq auto-mode-alist
      (cons '("\\.m$" . octave-mode) auto-mode-alist))
```

2. Finally, to turn on the abbrevs, auto-fill and font-lock features automatically, also add the following lines to one of the Emacs startup files:

```
(add-hook 'octave-mode-hook
          (lambda ()
            (abbrev-mode 1)
            (auto-fill-mode 1)
            (if (eq window-system 'x)
                (font-lock-mode 1))))
```

See the Emacs manual for more information about how to customize Font-lock mode.

In Octave mode, the following special Emacs commands can be used in addition to the standard Emacs commands.

C-h m Describe the features of Octave mode.

LFD Reindent the current Octave line, insert a newline and indent the new line (`octave-reindent-then-newline-and-indent`). An abbrev before point is expanded if `abbrev-mode` is non-`nil`.

TAB Indents current Octave line based on its contents and on previous lines (`indent-according-to-mode`).

; Insert an "electric" semicolon (`octave-electric-semi`). If `octave-auto-indent` is non-`nil`, reindent the current line. If `octave-auto-newline` is non-`nil`, automagically insert a newline and indent the new line.

`* Start entering an abbreviation (`octave-abbrev-start`). If Abbrev mode is turned on, typing *`C-h or *`?* lists all abbrevs. Any other key combination is executed normally. Note that all Octave abbrevs start with a grave accent.

M-LFD Break line at point and insert continuation marker and alignment (`octave-split-line`).

M-TAB Perform completion on Octave symbol preceding point, comparing that symbol against Octave's reserved words and built-in variables (`octave-complete-symbol`).

M-C-a Move backward to the beginning of a function (`octave-beginning-of-defun`). With prefix argument *N*, do it that many times if *N* is positive; otherwise, move forward to the *N*-th following beginning of a function.

M-C-e Move forward to the end of a function (`octave-end-of-defun`). With prefix argument *N*, do it that many times if *N* is positive; otherwise, move back to the *N*-th preceding end of a function.

M-C-h Puts point at beginning and mark at the end of the current Octave function, i.e., the one containing point or following point (`octave-mark-defun`).

M-C-q Properly indents the Octave function which contains point (`octave-indent-defun`).

M-; If there is no comment already on this line, create a code-level comment (started by two comment characters) if the line is empty, or an in-line comment (started by one comment character) otherwise (`octave-indent-for-comment`). Point is left after the start of the comment which is properly aligned.

C-c ; Puts the comment character '`#`' (more precisely, the string value of `octave-comment-start`) at the beginning of every line in the region (`octave-comment-region`). With just *C-u* prefix argument, uncomment each line in the region. A numeric prefix argument *N* means use *N* comment characters.

C-c : Uncomments every line in the region (`octave-uncomment-region`).

C-c C-p Move one line of Octave code backward, skipping empty and comment lines (`octave-previous-code-line`). With numeric prefix argument *N*, move that many code lines backward (forward if *N* is negative).

C-c C-n Move one line of Octave code forward, skipping empty and comment lines (`octave-next-code-line`). With numeric prefix argument *N*, move that many code lines forward (backward if *N* is negative).

C-c C-a Move to the 'real' beginning of the current line (`octave-beginning-of-line`). If point is in an empty or comment line, simply go to its beginning; otherwise, move backwards to the beginning of the first code line which is not inside a continuation statement, i.e., which does not follow a code line ending in '`...`' or '\', or is inside an open parenthesis list.

C-c C-e Move to the 'real' end of the current line (`octave-end-of-line`). If point is in a code line, move forward to the end of the first Octave code line which does not end in '`...`' or '\' or is inside an open parenthesis list. Otherwise, simply go to the end of the current line.

C-c M-C-n Move forward across one balanced begin-end block of Octave code (`octave-forward-block`). With numeric prefix argument *N*, move forward across *n* such blocks (backward if *N* is negative).

C-c M-C-p Move back across one balanced begin-end block of Octave code (`octave-backward-block`). With numeric prefix argument *N*, move backward across *N* such blocks (forward if *N* is negative).

C-c M-C-d Move forward down one begin-end block level of Octave code (`octave-down-block`). With numeric prefix argument, do it that many times; a negative argument means move backward, but still go down one level.

C-c M-C-u Move backward out of one begin-end block level of Octave code (`octave-backward-up-block`). With numeric prefix argument, do it that many times; a negative argument means move forward, but still to a less deep spot.

C-c M-C-h Put point at the beginning of this block, mark at the end (`octave-mark-block`). The block marked is the one that contains point or follows point.

C-c] Close the current block on a separate line (`octave-close-block`). An error is signaled if no block to close is found.

C-c f Insert a function skeleton, prompting for the function's name, arguments and return values which have to be entered without parentheses (`octave-insert-defun`).

C-c C-h Search the function, operator and variable indices of all info files with documentation for Octave for entries (`octave-help`). If used interactively, the entry is prompted for with completion. If multiple matches are found, one can cycle through them using the standard '`,`' (`Info-index-next`) command of the Info reader.

The variable `octave-help-files` is a list of files to search through and defaults to `'("octave")`. If there is also an Octave Local Guide with corresponding info file, say, '`octave-LG`', you can have `octave-help` search both files by

```
(setq octave-help-files '("octave" "octave-LG"))
```

in one of your Emacs startup files.

A common problem is that the RET key does *not* indent the line to where the new text should go after inserting the newline. This is because the standard Emacs convention is that RET (aka `C-m`) just adds a newline, whereas LFD (aka `C-j`) adds a newline and indents it. This is particularly inconvenient for users with keyboards which do not have a special LFD key at all; in such cases, it is typically more convenient to use RET as the LFD key (rather than typing `C-j`).

You can make RET do this by adding

```
(define-key octave-mode-map "\C-m"
  'octave-reindent-then-newline-and-indent)
```

to one of your Emacs startup files. Another, more generally applicable solution is

```
(defun RET-behaves-as-LFD ()
  (let ((x (key-binding "\C-j")))
    (local-set-key "\C-m" x)))
(add-hook 'octave-mode-hook 'RET-behaves-as-LFD)
```

(this works for all modes by adding to the startup hooks, without having to know the particular binding of RET in that mode!). Similar considerations apply for using M-RET as M-LFD. As Barry A. Warsaw <bwarsaw@cnri.reston.va.us> says in the documentation for his `cc-mode`, “This is a very common question. `:-)` If you want this to be the default behavior, don’t lobby me, lobby RMS!”

The following variables can be used to customize Octave mode.

`octave-auto-indent`
: Non-`nil` means auto-indent the current line after a semicolon or space. Default is `nil`.

`octave-auto-newline`
: Non-`nil` means auto-insert a newline and indent after semicolons are typed. The default value is `nil`.

`octave-blink-matching-block`
: Non-`nil` means show matching begin of block when inserting a space, newline or ‘`;`’ after an else or end keyword. Default is `t`. This is an extremely useful feature for automatically verifying that the keywords match—if they don’t, an error message is displayed.

`octave-block-offset`
: Extra indentation applied to statements in block structures. Default is 2.

`octave-continuation-offset`
: Extra indentation applied to Octave continuation lines. Default is 4.

`octave-continuation-string`
: String used for Octave continuation lines. Normally ‘\’.

`octave-mode-startup-message`
: If `t` (default), a startup message is displayed when Octave mode is called.

If Font Lock mode is enabled, Octave mode will display

- strings in `font-lock-string-face`
- comments in `font-lock-comment-face`

- the Octave reserved words (such as all block keywords) and the text functions (such as '`cd`' or '`who`') which are also reserved using `font-lock-keyword-face`
- the built-in operators ('`&&`', '`==`', ...) using `font-lock-reference-face`
- and the function names in function declarations in `font-lock-function-name-face`.

There is also rudimentary support for Imenu (currently, function names can be indexed).

You can generate TAGS files for Emacs from Octave '`.m`' files using the shell script `octave-tags` that is installed alongside your copy of Octave.

Customization of Octave mode can be performed by modification of the variable `octave-mode-hook`. If the value of this variable is non-`nil`, turning on Octave mode calls its value.

If you discover a problem with Octave mode, you can conveniently send a bug report using *C-c C-b* (`octave-submit-bug-report`). This automatically sets up a mail buffer with version information already added. You just need to add a description of the problem, including a reproducible test case and send the message.

G.3 Running Octave From Within Emacs

The package '`octave`' provides commands for running an inferior Octave process in a special Emacs buffer. Use

```
M-x run-octave
```

to directly start an inferior Octave process. If Emacs does not know about this command, add the line

```
(autoload 'run-octave "octave-inf" nil t)
```

to your '`.emacs`' file.

This will start Octave in a special buffer the name of which is specified by the variable `inferior-octave-buffer` and defaults to `"*Inferior Octave*"`. From within this buffer, you can interact with the inferior Octave process 'as usual', i.e., by entering Octave commands at the prompt. The buffer is in Inferior Octave mode, which is derived from the standard Comint mode, a major mode for interacting with an inferior interpreter. See the documentation for `comint-mode` for more details, and use *C-h b* to find out about available special keybindings.

You can also communicate with an inferior Octave process from within files with Octave code (i.e., buffers in Octave mode), using the following commands.

C-c i l Send the current line to the inferior Octave process (`octave-send-line`). With positive prefix argument *N*, send that many lines. If `octave-send-line-auto-forward` is non-`nil`, go to the next unsent code line.

C-c i b Send the current block to the inferior Octave process (`octave-send-block`).

C-c i f Send the current function to the inferior Octave process (`octave-send-defun`).

C-c i r Send the region to the inferior Octave process (`octave-send-region`).

C-c i s Make sure that 'inferior-octave-buffer' is displayed (`octave-show-process-buffer`).

C-c i h Delete all windows that display the inferior Octave buffer (`octave-hide-process-buffer`).

C-c i k Kill the inferior Octave process and its buffer (`octave-kill-process`).

The effect of the commands which send code to the Octave process can be customized by the following variables.

`octave-send-echo-input`
: Non-`nil` means echo input sent to the inferior Octave process. Default is `t`.

`octave-send-show-buffer`
: Non-`nil` means display the buffer running the Octave process after sending a command (but without selecting it). Default is `t`.

If you send code and there is no inferior Octave process yet, it will be started automatically.

The startup of the inferior Octave process is highly customizable. The variable `inferior-octave-startup-args` can be used for specifying command lines arguments to be passed to Octave on startup as a list of strings. For example, to suppress the startup message and use 'traditional' mode, set this to `'("-q" "--traditional")`. You can also specify a startup file of Octave commands to be loaded on startup; note that these commands will not produce any visible output in the process buffer. Which file to use is controlled by the variable `inferior-octave-startup-file`. If this is `nil`, the file '`~/.emacs-octave`' is used if it exists.

And finally, `inferior-octave-mode-hook` is run after starting the process and putting its buffer into Inferior Octave mode. Hence, if you like the up and down arrow keys to behave in the interaction buffer as in the shell, and you want this buffer to use nice colors, add

```
(add-hook 'inferior-octave-mode-hook
          (lambda ()
            (turn-on-font-lock)
            (define-key inferior-octave-mode-map [up]
              'comint-previous-input)
            (define-key inferior-octave-mode-map [down]
              'comint-next-input)))
```

to your '`.emacs`' file. You could also swap the roles of *C*-`a` (`beginning-of-line`) and `C-c C-a` (`comint-bol`) using this hook.

> **Note** that if you set your Octave prompts to something different from the defaults, make sure that `inferior-octave-prompt` matches them. Otherwise, *nothing* will work, because Emacs will not know when Octave is waiting for input, or done sending output.

G.4 Using the Emacs Info Reader for Octave

You may also use the Emacs Info reader with Octave's `doc` function. For this, the package '`gnuserv`' needs to be installed.

If '`gnuserv`' is installed, add the lines

```
(autoload 'octave-help "octave-hlp" nil t)
(require 'gnuserv)
(gnuserv-start)
```

to your '.emacs' file.

You can use either 'plain' Emacs Info or the function octave-help as your Octave info reader (for 'help -i'). In the former case, use info_program ("info-emacs-info"). The latter is perhaps more attractive because it allows to look up keys in the indices of *several* info files related to Octave (provided that the Emacs variable octave-help-files is set correctly). In this case, use info_program ("info-emacs-octave-help").

If you use Octave from within Emacs, it is best to add these settings to your '~/.emacs-octave' startup file (or the file pointed to by the Emacs variable inferior-octave-startup-file).

Appendix H GNU GENERAL PUBLIC LICENSE

Version 3, 29 June 2007

Copyright © 2007 Free Software Foundation, Inc. http://fsf.org/

Everyone is permitted to copy and distribute verbatim copies of this license document, but changing it is not allowed.

Preamble

The GNU General Public License is a free, copyleft license for software and other kinds of works.

The licenses for most software and other practical works are designed to take away your freedom to share and change the works. By contrast, the GNU General Public License is intended to guarantee your freedom to share and change all versions of a program—to make sure it remains free software for all its users. We, the Free Software Foundation, use the GNU General Public License for most of our software; it applies also to any other work released this way by its authors. You can apply it to your programs, too.

When we speak of free software, we are referring to freedom, not price. Our General Public Licenses are designed to make sure that you have the freedom to distribute copies of free software (and charge for them if you wish), that you receive source code or can get it if you want it, that you can change the software or use pieces of it in new free programs, and that you know you can do these things.

To protect your rights, we need to prevent others from denying you these rights or asking you to surrender the rights. Therefore, you have certain responsibilities if you distribute copies of the software, or if you modify it: responsibilities to respect the freedom of others.

For example, if you distribute copies of such a program, whether gratis or for a fee, you must pass on to the recipients the same freedoms that you received. You must make sure that they, too, receive or can get the source code. And you must show them these terms so they know their rights.

Developers that use the GNU GPL protect your rights with two steps: (1) assert copyright on the software, and (2) offer you this License giving you legal permission to copy, distribute and/or modify it.

For the developers' and authors' protection, the GPL clearly explains that there is no warranty for this free software. For both users' and authors' sake, the GPL requires that modified versions be marked as changed, so that their problems will not be attributed erroneously to authors of previous versions.

Some devices are designed to deny users access to install or run modified versions of the software inside them, although the manufacturer can do so. This is fundamentally incompatible with the aim of protecting users' freedom to change the software. The systematic pattern of such abuse occurs in the area of products for individuals to use, which is precisely where it is most unacceptable. Therefore, we have designed this version of the GPL to prohibit the practice for those products. If such problems arise substantially in other domains, we stand ready to extend this provision to those domains in future versions of the GPL, as needed to protect the freedom of users.

Finally, every program is threatened constantly by software patents. States should not allow patents to restrict development and use of software on general-purpose computers, but in those that do, we wish to avoid the special danger that patents applied to a free program could make it effectively proprietary. To prevent this, the GPL assures that patents cannot be used to render the program non-free.

The precise terms and conditions for copying, distribution and modification follow.

TERMS AND CONDITIONS

0. Definitions.

   "This License" refers to version 3 of the GNU General Public License.

   "Copyright" also means copyright-like laws that apply to other kinds of works, such as semiconductor masks.

   "The Program" refers to any copyrightable work licensed under this License. Each licensee is addressed as "you". "Licensees" and "recipients" may be individuals or organizations.

   To "modify" a work means to copy from or adapt all or part of the work in a fashion requiring copyright permission, other than the making of an exact copy. The resulting work is called a "modified version" of the earlier work or a work "based on" the earlier work.

   A "covered work" means either the unmodified Program or a work based on the Program.

   To "propagate" a work means to do anything with it that, without permission, would make you directly or secondarily liable for infringement under applicable copyright law, except executing it on a computer or modifying a private copy. Propagation includes copying, distribution (with or without modification), making available to the public, and in some countries other activities as well.

   To "convey" a work means any kind of propagation that enables other parties to make or receive copies. Mere interaction with a user through a computer network, with no transfer of a copy, is not conveying.

   An interactive user interface displays "Appropriate Legal Notices" to the extent that it includes a convenient and prominently visible feature that (1) displays an appropriate copyright notice, and (2) tells the user that there is no warranty for the work (except to the extent that warranties are provided), that licensees may convey the work under this License, and how to view a copy of this License. If the interface presents a list of user commands or options, such as a menu, a prominent item in the list meets this criterion.

1. Source Code.

   The "source code" for a work means the preferred form of the work for making modifications to it. "Object code" means any non-source form of a work.

   A "Standard Interface" means an interface that either is an official standard defined by a recognized standards body, or, in the case of interfaces specified for a particular programming language, one that is widely used among developers working in that language.

The “System Libraries” of an executable work include anything, other than the work as a whole, that (a) is included in the normal form of packaging a Major Component, but which is not part of that Major Component, and (b) serves only to enable use of the work with that Major Component, or to implement a Standard Interface for which an implementation is available to the public in source code form. A “Major Component”, in this context, means a major essential component (kernel, window system, and so on) of the specific operating system (if any) on which the executable work runs, or a compiler used to produce the work, or an object code interpreter used to run it.

The “Corresponding Source” for a work in object code form means all the source code needed to generate, install, and (for an executable work) run the object code and to modify the work, including scripts to control those activities. However, it does not include the work’s System Libraries, or general-purpose tools or generally available free programs which are used unmodified in performing those activities but which are not part of the work. For example, Corresponding Source includes interface definition files associated with source files for the work, and the source code for shared libraries and dynamically linked subprograms that the work is specifically designed to require, such as by intimate data communication or control flow between those subprograms and other parts of the work.

The Corresponding Source need not include anything that users can regenerate automatically from other parts of the Corresponding Source.

The Corresponding Source for a work in source code form is that same work.

2. Basic Permissions.

   All rights granted under this License are granted for the term of copyright on the Program, and are irrevocable provided the stated conditions are met. This License explicitly affirms your unlimited permission to run the unmodified Program. The output from running a covered work is covered by this License only if the output, given its content, constitutes a covered work. This License acknowledges your rights of fair use or other equivalent, as provided by copyright law.

   You may make, run and propagate covered works that you do not convey, without conditions so long as your license otherwise remains in force. You may convey covered works to others for the sole purpose of having them make modifications exclusively for you, or provide you with facilities for running those works, provided that you comply with the terms of this License in conveying all material for which you do not control copyright. Those thus making or running the covered works for you must do so exclusively on your behalf, under your direction and control, on terms that prohibit them from making any copies of your copyrighted material outside their relationship with you.

   Conveying under any other circumstances is permitted solely under the conditions stated below. Sublicensing is not allowed; section 10 makes it unnecessary.

3. Protecting Users’ Legal Rights From Anti-Circumvention Law.

   No covered work shall be deemed part of an effective technological measure under any applicable law fulfilling obligations under article 11 of the WIPO copyright treaty adopted on 20 December 1996, or similar laws prohibiting or restricting circumvention of such measures.

When you convey a covered work, you waive any legal power to forbid circumvention of technological measures to the extent such circumvention is effected by exercising rights under this License with respect to the covered work, and you disclaim any intention to limit operation or modification of the work as a means of enforcing, against the work's users, your or third parties' legal rights to forbid circumvention of technological measures.

4. Conveying Verbatim Copies.

   You may convey verbatim copies of the Program's source code as you receive it, in any medium, provided that you conspicuously and appropriately publish on each copy an appropriate copyright notice; keep intact all notices stating that this License and any non-permissive terms added in accord with section 7 apply to the code; keep intact all notices of the absence of any warranty; and give all recipients a copy of this License along with the Program.

   You may charge any price or no price for each copy that you convey, and you may offer support or warranty protection for a fee.

5. Conveying Modified Source Versions.

   You may convey a work based on the Program, or the modifications to produce it from the Program, in the form of source code under the terms of section 4, provided that you also meet all of these conditions:

   a. The work must carry prominent notices stating that you modified it, and giving a relevant date.
   b. The work must carry prominent notices stating that it is released under this License and any conditions added under section 7. This requirement modifies the requirement in section 4 to "keep intact all notices".
   c. You must license the entire work, as a whole, under this License to anyone who comes into possession of a copy. This License will therefore apply, along with any applicable section 7 additional terms, to the whole of the work, and all its parts, regardless of how they are packaged. This License gives no permission to license the work in any other way, but it does not invalidate such permission if you have separately received it.
   d. If the work has interactive user interfaces, each must display Appropriate Legal Notices; however, if the Program has interactive interfaces that do not display Appropriate Legal Notices, your work need not make them do so.

   A compilation of a covered work with other separate and independent works, which are not by their nature extensions of the covered work, and which are not combined with it such as to form a larger program, in or on a volume of a storage or distribution medium, is called an "aggregate" if the compilation and its resulting copyright are not used to limit the access or legal rights of the compilation's users beyond what the individual works permit. Inclusion of a covered work in an aggregate does not cause this License to apply to the other parts of the aggregate.

6. Conveying Non-Source Forms.

   You may convey a covered work in object code form under the terms of sections 4 and 5, provided that you also convey the machine-readable Corresponding Source under the terms of this License, in one of these ways:

a. Convey the object code in, or embodied in, a physical product (including a physical distribution medium), accompanied by the Corresponding Source fixed on a durable physical medium customarily used for software interchange.
b. Convey the object code in, or embodied in, a physical product (including a physical distribution medium), accompanied by a written offer, valid for at least three years and valid for as long as you offer spare parts or customer support for that product model, to give anyone who possesses the object code either (1) a copy of the Corresponding Source for all the software in the product that is covered by this License, on a durable physical medium customarily used for software interchange, for a price no more than your reasonable cost of physically performing this conveying of source, or (2) access to copy the Corresponding Source from a network server at no charge.
c. Convey individual copies of the object code with a copy of the written offer to provide the Corresponding Source. This alternative is allowed only occasionally and noncommercially, and only if you received the object code with such an offer, in accord with subsection 6b.
d. Convey the object code by offering access from a designated place (gratis or for a charge), and offer equivalent access to the Corresponding Source in the same way through the same place at no further charge. You need not require recipients to copy the Corresponding Source along with the object code. If the place to copy the object code is a network server, the Corresponding Source may be on a different server (operated by you or a third party) that supports equivalent copying facilities, provided you maintain clear directions next to the object code saying where to find the Corresponding Source. Regardless of what server hosts the Corresponding Source, you remain obligated to ensure that it is available for as long as needed to satisfy these requirements.
e. Convey the object code using peer-to-peer transmission, provided you inform other peers where the object code and Corresponding Source of the work are being offered to the general public at no charge under subsection 6d.

A separable portion of the object code, whose source code is excluded from the Corresponding Source as a System Library, need not be included in conveying the object code work.

A “User Product” is either (1) a “consumer product”, which means any tangible personal property which is normally used for personal, family, or household purposes, or (2) anything designed or sold for incorporation into a dwelling. In determining whether a product is a consumer product, doubtful cases shall be resolved in favor of coverage. For a particular product received by a particular user, “normally used” refers to a typical or common use of that class of product, regardless of the status of the particular user or of the way in which the particular user actually uses, or expects or is expected to use, the product. A product is a consumer product regardless of whether the product has substantial commercial, industrial or non-consumer uses, unless such uses represent the only significant mode of use of the product.

“Installation Information” for a User Product means any methods, procedures, authorization keys, or other information required to install and execute modified versions of a covered work in that User Product from a modified version of its Corresponding Source.

The information must suffice to ensure that the continued functioning of the modified object code is in no case prevented or interfered with solely because modification has been made.

If you convey an object code work under this section in, or with, or specifically for use in, a User Product, and the conveying occurs as part of a transaction in which the right of possession and use of the User Product is transferred to the recipient in perpetuity or for a fixed term (regardless of how the transaction is characterized), the Corresponding Source conveyed under this section must be accompanied by the Installation Information. But this requirement does not apply if neither you nor any third party retains the ability to install modified object code on the User Product (for example, the work has been installed in ROM).

The requirement to provide Installation Information does not include a requirement to continue to provide support service, warranty, or updates for a work that has been modified or installed by the recipient, or for the User Product in which it has been modified or installed. Access to a network may be denied when the modification itself materially and adversely affects the operation of the network or violates the rules and protocols for communication across the network.

Corresponding Source conveyed, and Installation Information provided, in accord with this section must be in a format that is publicly documented (and with an implementation available to the public in source code form), and must require no special password or key for unpacking, reading or copying.

7. Additional Terms.

“Additional permissions” are terms that supplement the terms of this License by making exceptions from one or more of its conditions. Additional permissions that are applicable to the entire Program shall be treated as though they were included in this License, to the extent that they are valid under applicable law. If additional permissions apply only to part of the Program, that part may be used separately under those permissions, but the entire Program remains governed by this License without regard to the additional permissions.

When you convey a copy of a covered work, you may at your option remove any additional permissions from that copy, or from any part of it. (Additional permissions may be written to require their own removal in certain cases when you modify the work.) You may place additional permissions on material, added by you to a covered work, for which you have or can give appropriate copyright permission.

Notwithstanding any other provision of this License, for material you add to a covered work, you may (if authorized by the copyright holders of that material) supplement the terms of this License with terms:

a. Disclaiming warranty or limiting liability differently from the terms of sections 15 and 16 of this License; or
b. Requiring preservation of specified reasonable legal notices or author attributions in that material or in the Appropriate Legal Notices displayed by works containing it; or
c. Prohibiting misrepresentation of the origin of that material, or requiring that modified versions of such material be marked in reasonable ways as different from the original version; or

d. Limiting the use for publicity purposes of names of licensors or authors of the material; or
e. Declining to grant rights under trademark law for use of some trade names, trademarks, or service marks; or
f. Requiring indemnification of licensors and authors of that material by anyone who conveys the material (or modified versions of it) with contractual assumptions of liability to the recipient, for any liability that these contractual assumptions directly impose on those licensors and authors.

All other non-permissive additional terms are considered "further restrictions" within the meaning of section 10. If the Program as you received it, or any part of it, contains a notice stating that it is governed by this License along with a term that is a further restriction, you may remove that term. If a license document contains a further restriction but permits relicensing or conveying under this License, you may add to a covered work material governed by the terms of that license document, provided that the further restriction does not survive such relicensing or conveying.

If you add terms to a covered work in accord with this section, you must place, in the relevant source files, a statement of the additional terms that apply to those files, or a notice indicating where to find the applicable terms.

Additional terms, permissive or non-permissive, may be stated in the form of a separately written license, or stated as exceptions; the above requirements apply either way.

8. Termination.

You may not propagate or modify a covered work except as expressly provided under this License. Any attempt otherwise to propagate or modify it is void, and will automatically terminate your rights under this License (including any patent licenses granted under the third paragraph of section 11).

However, if you cease all violation of this License, then your license from a particular copyright holder is reinstated (a) provisionally, unless and until the copyright holder explicitly and finally terminates your license, and (b) permanently, if the copyright holder fails to notify you of the violation by some reasonable means prior to 60 days after the cessation.

Moreover, your license from a particular copyright holder is reinstated permanently if the copyright holder notifies you of the violation by some reasonable means, this is the first time you have received notice of violation of this License (for any work) from that copyright holder, and you cure the violation prior to 30 days after your receipt of the notice.

Termination of your rights under this section does not terminate the licenses of parties who have received copies or rights from you under this License. If your rights have been terminated and not permanently reinstated, you do not qualify to receive new licenses for the same material under section 10.

9. Acceptance Not Required for Having Copies.

You are not required to accept this License in order to receive or run a copy of the Program. Ancillary propagation of a covered work occurring solely as a consequence of using peer-to-peer transmission to receive a copy likewise does not require acceptance.

However, nothing other than this License grants you permission to propagate or modify any covered work. These actions infringe copyright if you do not accept this License. Therefore, by modifying or propagating a covered work, you indicate your acceptance of this License to do so.

10. Automatic Licensing of Downstream Recipients.

    Each time you convey a covered work, the recipient automatically receives a license from the original licensors, to run, modify and propagate that work, subject to this License. You are not responsible for enforcing compliance by third parties with this License.

    An "entity transaction" is a transaction transferring control of an organization, or substantially all assets of one, or subdividing an organization, or merging organizations. If propagation of a covered work results from an entity transaction, each party to that transaction who receives a copy of the work also receives whatever licenses to the work the party's predecessor in interest had or could give under the previous paragraph, plus a right to possession of the Corresponding Source of the work from the predecessor in interest, if the predecessor has it or can get it with reasonable efforts.

    You may not impose any further restrictions on the exercise of the rights granted or affirmed under this License. For example, you may not impose a license fee, royalty, or other charge for exercise of rights granted under this License, and you may not initiate litigation (including a cross-claim or counterclaim in a lawsuit) alleging that any patent claim is infringed by making, using, selling, offering for sale, or importing the Program or any portion of it.

11. Patents.

    A "contributor" is a copyright holder who authorizes use under this License of the Program or a work on which the Program is based. The work thus licensed is called the contributor's "contributor version".

    A contributor's "essential patent claims" are all patent claims owned or controlled by the contributor, whether already acquired or hereafter acquired, that would be infringed by some manner, permitted by this License, of making, using, or selling its contributor version, but do not include claims that would be infringed only as a consequence of further modification of the contributor version. For purposes of this definition, "control" includes the right to grant patent sublicenses in a manner consistent with the requirements of this License.

    Each contributor grants you a non-exclusive, worldwide, royalty-free patent license under the contributor's essential patent claims, to make, use, sell, offer for sale, import and otherwise run, modify and propagate the contents of its contributor version.

    In the following three paragraphs, a "patent license" is any express agreement or commitment, however denominated, not to enforce a patent (such as an express permission to practice a patent or covenant not to sue for patent infringement). To "grant" such a patent license to a party means to make such an agreement or commitment not to enforce a patent against the party.

    If you convey a covered work, knowingly relying on a patent license, and the Corresponding Source of the work is not available for anyone to copy, free of charge and under the terms of this License, through a publicly available network server or other readily accessible means, then you must either (1) cause the Corresponding Source to be so

available, or (2) arrange to deprive yourself of the benefit of the patent license for this particular work, or (3) arrange, in a manner consistent with the requirements of this License, to extend the patent license to downstream recipients. “Knowingly relying” means you have actual knowledge that, but for the patent license, your conveying the covered work in a country, or your recipient’s use of the covered work in a country, would infringe one or more identifiable patents in that country that you have reason to believe are valid.

If, pursuant to or in connection with a single transaction or arrangement, you convey, or propagate by procuring conveyance of, a covered work, and grant a patent license to some of the parties receiving the covered work authorizing them to use, propagate, modify or convey a specific copy of the covered work, then the patent license you grant is automatically extended to all recipients of the covered work and works based on it.

A patent license is “discriminatory” if it does not include within the scope of its coverage, prohibits the exercise of, or is conditioned on the non-exercise of one or more of the rights that are specifically granted under this License. You may not convey a covered work if you are a party to an arrangement with a third party that is in the business of distributing software, under which you make payment to the third party based on the extent of your activity of conveying the work, and under which the third party grants, to any of the parties who would receive the covered work from you, a discriminatory patent license (a) in connection with copies of the covered work conveyed by you (or copies made from those copies), or (b) primarily for and in connection with specific products or compilations that contain the covered work, unless you entered into that arrangement, or that patent license was granted, prior to 28 March 2007.

Nothing in this License shall be construed as excluding or limiting any implied license or other defenses to infringement that may otherwise be available to you under applicable patent law.

12. No Surrender of Others’ Freedom.

    If conditions are imposed on you (whether by court order, agreement or otherwise) that contradict the conditions of this License, they do not excuse you from the conditions of this License. If you cannot convey a covered work so as to satisfy simultaneously your obligations under this License and any other pertinent obligations, then as a consequence you may not convey it at all. For example, if you agree to terms that obligate you to collect a royalty for further conveying from those to whom you convey the Program, the only way you could satisfy both those terms and this License would be to refrain entirely from conveying the Program.

13. Use with the GNU Affero General Public License.

    Notwithstanding any other provision of this License, you have permission to link or combine any covered work with a work licensed under version 3 of the GNU Affero General Public License into a single combined work, and to convey the resulting work. The terms of this License will continue to apply to the part which is the covered work, but the special requirements of the GNU Affero General Public License, section 13, concerning interaction through a network will apply to the combination as such.

14. Revised Versions of this License.

The Free Software Foundation may publish revised and/or new versions of the GNU General Public License from time to time. Such new versions will be similar in spirit to the present version, but may differ in detail to address new problems or concerns.

Each version is given a distinguishing version number. If the Program specifies that a certain numbered version of the GNU General Public License "or any later version" applies to it, you have the option of following the terms and conditions either of that numbered version or of any later version published by the Free Software Foundation. If the Program does not specify a version number of the GNU General Public License, you may choose any version ever published by the Free Software Foundation.

If the Program specifies that a proxy can decide which future versions of the GNU General Public License can be used, that proxy's public statement of acceptance of a version permanently authorizes you to choose that version for the Program.

Later license versions may give you additional or different permissions. However, no additional obligations are imposed on any author or copyright holder as a result of your choosing to follow a later version.

15. Disclaimer of Warranty.

    THERE IS NO WARRANTY FOR THE PROGRAM, TO THE EXTENT PERMITTED BY APPLICABLE LAW. EXCEPT WHEN OTHERWISE STATED IN WRITING THE COPYRIGHT HOLDERS AND/OR OTHER PARTIES PROVIDE THE PROGRAM "AS IS" WITHOUT WARRANTY OF ANY KIND, EITHER EXPRESSED OR IMPLIED, INCLUDING, BUT NOT LIMITED TO, THE IMPLIED WARRANTIES OF MERCHANTABILITY AND FITNESS FOR A PARTICULAR PURPOSE. THE ENTIRE RISK AS TO THE QUALITY AND PERFORMANCE OF THE PROGRAM IS WITH YOU. SHOULD THE PROGRAM PROVE DEFECTIVE, YOU ASSUME THE COST OF ALL NECESSARY SERVICING, REPAIR OR CORRECTION.

16. Limitation of Liability.

    IN NO EVENT UNLESS REQUIRED BY APPLICABLE LAW OR AGREED TO IN WRITING WILL ANY COPYRIGHT HOLDER, OR ANY OTHER PARTY WHO MODIFIES AND/OR CONVEYS THE PROGRAM AS PERMITTED ABOVE, BE LIABLE TO YOU FOR DAMAGES, INCLUDING ANY GENERAL, SPECIAL, INCIDENTAL OR CONSEQUENTIAL DAMAGES ARISING OUT OF THE USE OR INABILITY TO USE THE PROGRAM (INCLUDING BUT NOT LIMITED TO LOSS OF DATA OR DATA BEING RENDERED INACCURATE OR LOSSES SUSTAINED BY YOU OR THIRD PARTIES OR A FAILURE OF THE PROGRAM TO OPERATE WITH ANY OTHER PROGRAMS), EVEN IF SUCH HOLDER OR OTHER PARTY HAS BEEN ADVISED OF THE POSSIBILITY OF SUCH DAMAGES.

17. Interpretation of Sections 15 and 16.

    If the disclaimer of warranty and limitation of liability provided above cannot be given local legal effect according to their terms, reviewing courts shall apply local law that most closely approximates an absolute waiver of all civil liability in connection with the Program, unless a warranty or assumption of liability accompanies a copy of the Program in return for a fee.

END OF TERMS AND CONDITIONS

How to Apply These Terms to Your New Programs

If you develop a new program, and you want it to be of the greatest possible use to the public, the best way to achieve this is to make it free software which everyone can redistribute and change under these terms.

To do so, attach the following notices to the program. It is safest to attach them to the start of each source file to most effectively state the exclusion of warranty; and each file should have at least the "copyright" line and a pointer to where the full notice is found.

```
one line to give the program's name and a brief idea of what it does.
Copyright (C) year name of author

This program is free software: you can redistribute it and/or modify
it under the terms of the GNU General Public License as published by
the Free Software Foundation, either version 3 of the License, or (at
your option) any later version.

This program is distributed in the hope that it will be useful, but
WITHOUT ANY WARRANTY; without even the implied warranty of
MERCHANTABILITY or FITNESS FOR A PARTICULAR PURPOSE.  See the GNU
General Public License for more details.

You should have received a copy of the GNU General Public License
along with this program.  If not, see http://www.gnu.org/licenses/.
```

Also add information on how to contact you by electronic and paper mail.

If the program does terminal interaction, make it output a short notice like this when it starts in an interactive mode:

```
program Copyright (C) year name of author
This program comes with ABSOLUTELY NO WARRANTY; for details type 'show w'.
This is free software, and you are welcome to redistribute it
under certain conditions; type 'show c' for details.
```

The hypothetical commands '`show w`' and '`show c`' should show the appropriate parts of the General Public License. Of course, your program's commands might be different; for a GUI interface, you would use an "about box".

You should also get your employer (if you work as a programmer) or school, if any, to sign a "copyright disclaimer" for the program, if necessary. For more information on this, and how to apply and follow the GNU GPL, see `http://www.gnu.org/licenses/`.

The GNU General Public License does not permit incorporating your program into proprietary programs. If your program is a subroutine library, you may consider it more useful to permit linking proprietary applications with the library. If this is what you want to do, use the GNU Lesser General Public License instead of this License. But first, please read `http://www.gnu.org/philosophy/why-not-lgpl.html`.

Concept Index

#

'#' 29
'#!' 28
'#{' 30

%

'%' 29
'%{' 30

-

--braindead 15
--debug 13
--doc-cache-file *filename* 13
--echo-commands 13
--exec-path *path* 13
--help 13
--image-path *path* 13
--info-file *filename* 14
--info-program *program* 14
--interactive 14
--line-editing 14
--no-history 14
--no-init-file 14
--no-init-path 14
--no-line-editing 14
--no-site-file 14
--norc 14
--path *path* 14
--persist 14
--quiet 14
--silent 14
--traditional 15
--verbose 15
--version 15
-? 13
-d 13
-f 14
-h 13
-H 14
-i 14
-p *path* 14
-q 14
-v 15
-V 15
-x 13

.

... continuation marker 135
.octaverc 16

\

\ continuation marker 135

~

'~/.inputrc' 24
~/.octaverc 16

A

acknowledgements 1
addition 111
and operator 113
anonymous functions 155
ans 97
answers, incorrect 622, 623
area series 264
arguments in function call 109
arithmetic operators 111
assignment expressions 115
assignment operators 115
axes graphics object 243
axes properties 249

B

backends, graphics 271
bar series 264
batch processing 28
block comments 30
body of a loop 129
boolean expressions 113
boolean operators 113
break statement 132
bug criteria 622
bug report mailing lists 622
bugs 621
bugs, investigating 623
bugs, known 621
bugs, reporting 622
built-in data types 33
built-in function 10

C

callbacks 259
case statement 127
catch 134
cell arrays 35, 85
character strings 35, 55
Cholesky factorization 320
clearing the screen 20
coding standards 605, 615
colors, graphics 258

comma separated lists . . . 93
command and output logs . . . 26
command completion . . . 22
command descriptions . . . 11
command echoing . . . 26
command history . . . 22
command options . . . 13
command-line editing . . . 19
comments . . . 29
comparison expressions . . . 113
complex-conjugate transpose . . . 111
containers . . . 77
continuation lines . . . 135
`continue` statement . . . 133
contour series . . . 265
contributing to Octave . . . 4
contributors . . . 1
conversion specifications (`printf`) . . . 191
conversion specifications (`scanf`) . . . 196
copyright . . . 641
core dump . . . 622
cs-lists . . . 93
customizing `readline` . . . 24
customizing the prompt . . . 25

D

DAE . . . 389
data sources in object groups . . . 263
data structures . . . 35, 77
data types . . . 33
data types, built-in . . . 33
data types, user-defined . . . 35
decrement operator . . . 117
default arguments . . . 144
default graphics properties . . . 257
defining functions . . . 137
description format . . . 10
diary of commands and output . . . 26
Differential Equations . . . 389
diffs, submitting . . . 624
distribution of Octave . . . 4
division . . . 111
`do-until` statement . . . 130
documentation fonts . . . 9
documentation notation . . . 9
documenting functions . . . 30
documenting Octave programs . . . 29
documenting user scripts . . . 30
Dulmage-Mendelsohn decomposition . . . 362
dynamic-linking . . . 557

E

echoing executing commands . . . 26
editing the command line . . . 19
element-by-element evaluation . . . 113
`else` statement . . . 125
`elseif` statement . . . 125
Emacs TAGS files . . . 637
`end` statement . . . 125
`end_try_catch` . . . 134
`end_unwind_protect` . . . 134
`endfor` statement . . . 130
`endfunction` statement . . . 137
`endif` statement . . . 125
`endswitch` statement . . . 127
`endwhile` statement . . . 129
equality operator . . . 113
equality, tests for . . . 113
equations, nonlinear . . . 331
erroneous messages . . . 622
erroneous results . . . 622, 623
error bar series . . . 267
error message notation . . . 10
error messages . . . 27
error messages, incorrect . . . 622
escape sequence notation . . . 55
evaluation notation . . . 9
executable scripts . . . 28
execution speed . . . 605
exiting octave . . . 5, 17
exponentiation . . . 111
expression, range . . . 43
expressions . . . 107
expressions, assignment . . . 115
expressions, boolean . . . 113
expressions, comparison . . . 113
expressions, logical . . . 113

F

factorial function . . . 111
fatal signal . . . 622
figure graphics object . . . 243
figure properties . . . 249
flag character (`printf`) . . . 192
flag character (`scanf`) . . . 197
flying high and fast . . . 97
`for` statement . . . 130
Fordyce, A. P. . . . 121
Frobenius norm . . . 318
function descriptions . . . 10
function file . . . 10, 145
`function` statement . . . 137
functions, user-defined . . . 137
funding Octave development . . . 4

G

general p-norm . . . 318
getting a good job . . . 97
`global` statement . . . 99
global variables . . . 99
gnuplot interaction . . . 272
graphics . . . 205

graphics backends . . . 271
graphics colors . . . 258
graphics line styles . . . 258
graphics marker styles . . . 258
graphics object properties . . . 249
graphics object, axes . . . 243
graphics object, figure . . . 243
graphics object, image . . . 243
graphics object, line . . . 243
graphics object, patch . . . 243
graphics object, root figure . . . 243
graphics object, surface . . . 243
graphics object, text . . . 243
graphics properties, default . . . 257
greater than operator . . . 113
group objects . . . 268, 269, 271

H

handle, function handles . . . 155
header comments . . . 608
help, on-line . . . 17
help, user-defined functions . . . 30
help, where to find . . . 625
Hermitian operator . . . 111
Hessenberg decomposition . . . 321
history . . . 1
history of commands . . . 22

I

`if` statement . . . 125
image graphics object . . . 243
image properties . . . 255
improving Octave . . . 622, 624
incorrect error messages . . . 622
incorrect output . . . 622, 623
incorrect results . . . 622, 623
increment operator . . . 117
infinity norm . . . 318
initialization . . . 16
inline, inline functions . . . 155
input conversions, for `scanf` . . . 197
input history . . . 22
installation trouble . . . 621
installing Octave . . . 627
introduction . . . 5
invalid input . . . 622

J

job hunting . . . 97

K

known causes of trouble . . . 621

L

less than operator . . . 113
line graphics object . . . 243
line properties . . . 251
line series . . . 267
line styles, graphics . . . 258
loadable function . . . 11
loading data . . . 180
logging commands and output . . . 26
logical expressions . . . 113
logical operators . . . 113
loop . . . 129
looping over structure elements . . . 131
LP . . . 401
LU decomposition . . . 322, 375
lvalue . . . 116

M

mapping function . . . 11
marker styles, graphics . . . 258
matching failure, in `scanf` . . . 197
matrices . . . 40
matrix multiplication . . . 111
maximum field width (`scanf`) . . . 197
messages, error . . . 27
mex . . . 580
mex-files . . . 580
minimum field width (`printf`) . . . 192
missing data . . . 34
mkoctfile . . . 557
multi-line comments . . . 30
multiplication . . . 111

N

negation . . . 111
NLP . . . 401
nonlinear equations . . . 331
nonlinear programming . . . 401
not operator . . . 113
numeric constant . . . 34, 39
numeric value . . . 34, 39

O

object groups . . . 260
oct . . . 557
oct-files . . . 557
Octave command options . . . 13
Octave development . . . 615
`octave-tags` . . . 637
ODE . . . 389
on-line help . . . 17
operator precedence . . . 118
operators, arithmetic . . . 111
operators, assignment . . . 115
operators, boolean . . . 113

operators, decrement . . . 117
operators, increment . . . 117
operators, logical . . . 113
operators, relational . . . 113
optimization . . . 401
options, Octave command . . . 13
or operator . . . 113
oregonator . . . 391
`otherwise` statement . . . 127
output conversions, for `printf` . . . 193

P

patch graphics object . . . 243
patch properties . . . 255
patches, submitting . . . 624
`persistent` statement . . . 100
persistent variables . . . 100
personal startup file . . . 16
plotting . . . 205
precision (`printf`) . . . 193
printing notation . . . 9
program, self contained . . . 28
project startup file . . . 16
prompt customization . . . 25

Q

QP . . . 401
QR factorization . . . 323
quadratic programming . . . 401
quitting octave . . . 5, 17
quiver group . . . 268
quotient . . . 111

R

range expressions . . . 43
`readline` customization . . . 24
relational operators . . . 113
reporting bugs . . . 621, 622
results, incorrect . . . 622, 623
root figure graphics object . . . 243

S

saving data . . . 180
scatter group . . . 269
Schur decomposition . . . 326
script files . . . 137
scripts . . . 28
self contained programs . . . 28
series objects . . . 264, 265, 267, 270
short-circuit evaluation . . . 114
side effect . . . 115
singular value decomposition . . . 326
site startup file . . . 16
speedups . . . 605
stair group . . . 269
standards of coding style . . . 605
startup . . . 16
startup files . . . 16
statements . . . 125
stem series . . . 270
strings . . . 35, 55
Structural Rank . . . 367
structure elements, looping over . . . 131
structures . . . 35, 77
submitting diffs . . . 624
submitting patches . . . 624
subtraction . . . 111
suggestions . . . 622
surface graphics object . . . 243
surface group . . . 271
surface properties . . . 256
`switch` statement . . . 127

T

TAGS . . . 637
test functions . . . 597
tests for equality . . . 113
text graphics object . . . 243
text properties . . . 252
tips . . . 605
transpose . . . 111
transpose, complex-conjugate . . . 111
troubleshooting . . . 621
`try` statement . . . 134

U

unary minus . . . 111
undefined behavior . . . 622
undefined function value . . . 622
`unwind_protect` statement . . . 134
`unwind_protect_cleanup` . . . 134
use of comments . . . 29
user-defined data types . . . 35
user-defined functions . . . 137
user-defined variables . . . 97

V

`varargin` . . . 141
`varargout` . . . 143
variable descriptions . . . 11
variable-length argument lists . . . 141
variable-length return lists . . . 143
variables, global . . . 99
variables, persistent . . . 100
variables, user-defined . . . 97
version startup file . . . 16

W

warranty 641
`while` statement 129
wrong answers 622, 623

Function Index

(

() 15, 536

-

-all 18

=

= 331, 368

A

abs 294
accumarray 300
acos 296
acosd 298
acosh 297
acot 296
acotd 299
acoth 297
acsc 296
acscd 299
acsch 297
addlistener 261
addpath 148
addproperty 260
addtodate 523
airy 307
all 273
allchild 245
amd 358
ancestor 245
angle 295
anova 422
any 273
arch_fit 479
arch_rnd 480
arch_test 480
area 221
arg 295
argnames 156
argv 15
arma_rnd 480
arrayfun 282
asctime 517
asec 296
asecd 299
asech 297
asin 296
asind 298
asinh 297
assert 600
assignin 123
atan 296
atan2 297
atand 298
atanh 297
atexit 17
autocor 480
autocov 480
autoload 151
autoreg_matrix 481
autumn 490
available_backends 272
axes 245
axis 221

B

backend 271
balance 315
bar 208
barh 209
bartlett 481
bartlett_test 422
base2dec 72
beep 163
beep_on_error 163
besselh 307
besseli 307
besselj 307
besselk 307
bessely 307
beta 308
betacdf 430
betainc 308
betainv 430
betaln 308
betapdf 430
betarnd 437
bicgstab 329
bicubic 462
bin2dec 70
bincoeff 308
binocdf 430
binoinv 430
binopdf 430
binornd 437
bitand 49
bitcmp 49
bitget 48
bitmax 49
bitor 49
bitset 48
bitshift 49
bitxor 49
blackman 481
blanks 57

blkdiag 282
bone 490
box 237
brighten 490
bsxfun 284
bug_report 621, 622
builtin 151
bunzip2 530
byte_size 561
bzip2 531

C

C 102
calendar 523
canonicalize_file_name 529
cart2pol 311
cart2sph 311
cast 33
cat 277
cauchy_cdf 431
cauchy_inv 431
cauchy_pdf 431
cauchy_rnd 437
caxis 223
ccolamd 359
cd 11, 540
ceil 301
cell 87
cell2mat 92
cell2struct 93
celldisp 86
cellfun 91
cellidx 91
cellstr 90
center 417
cgs 329
char 58, 59
chdir 11, 540
chi2cdf 431
chi2inv 431
chi2rnd 438
chisquare_pdf 431
chisquare_test_homogeneity 422
chisquare_test_independence 422
chol 320
chol2inv 320
choldelete 321
cholinsert 321
cholinv 320
cholshift 321
cholupdate 320
circshift 278
cla 248
clabel 237
class 33
clc 21
clear 104
clf 248
clock 519
cloglog 419
close 248
closereq 248
colamd 360
colloc 385
colon 506
colorbar 238
colormap 490
colperm 361
columns 35
comet 221
command 548
command_line_path 149
common_size 275
commutation_matrix 309
compan 446
compass 220
complement 442
completion_append_char 22
completion_matches 22
complex 39
computer 542
cond 316
condest 365
confirm_recursive_rmdir 525
conj 295
contour 213, 214
contour3 215
contourc 214
contourf 214
contrast 492
conv 447
conv2 447
convhull 471
convhulln 471
convn 447
cool 491
copper 491
copyfile 529
cor 416
cor_test 422
corrcoef 416
cos 296
cosd 298
cosh 297
cot 296
cotd 298
coth 297
cov 416
cplxpair 295
cputime 519
crash_dumps_octave_core 186
cross 301
csc 296
cscd 298
csch 297

cstrcat 60
csvread 185
csvwrite 185
csymamd 361
ctime 516
cummax 305
cummin 305
cumprod 300
cumsum 299
cumtrapz 385
cut 420
cylinder 235

D

daspk 391
daspk_options 392
dasrt 397
dasrt_options 398
dassl 395
dassl_options 396
date 519
datenum 521
datestr 521
datetick 524
datevec 523
dbclear 172
dbcont 171
dbdown 174
dblquad 386
dbquit 171
dbstack 174
dbstatus 172
dbstep 174
dbstop 172
dbtype 173
dbup 174
dbwhere 173
deal 143
deblank 64
debug_on_error 171
debug_on_interrupt 171
debug_on_warning 171
dec2base 71
dec2bin 70
dec2hex 71
deconv 447
default_save_options 183
del2 301
delaunay 463
delaunay3 463
delaunayn 464
delete 248
dellistener 262
demo 601
det 316
detrend 475
diag 282
diary 26
diff 274
diffpara 481
diffuse 229
dims 561
dir 540
discrete_cdf 431
discrete_inv 431
discrete_pdf 431
discrete_rnd 438
disp 175
dispatch 151
display 499
dlmread 185
dlmwrite 184
dmperm 362
dmult 316
do_string_escapes 74
doc_cache_file 19
dos 533
dot 316
double 39
drawnow 246
dsearch 467
dsearchn 468
dup2 536
duplication_matrix 309
durbinlevinson 481

E

e 311
echo 26
echo_executing_commands 27
edit 145
edit_history 23
EDITOR 24
eig 316
elem 561
ellipsoid 235
empirical_cdf 431
empirical_inv 431
empirical_pdf 431
empirical_rnd 438
endgrent 542
endpwent 541
eomday 524
eps 313
erf 309
erfc 309
erfinv 309
errno 165
errno_list 165
error 161
errorbar 216
etime 519
etree 352
etreeplot 352

eval ... 121
evalin ... 123
example ... 602
exec ... 535
EXEC_PATH ... 535
exist ... 104
exit ... 17
exp ... 293
expcdf ... 432
expinv ... 432
expm ... 328
expm1 ... 293
exppdf ... 432
exprnd ... 438
eye ... 284
ezcontour ... 224
ezcontourf ... 225
ezmesh ... 232
ezmeshc ... 233
ezplot ... 224
ezplot3 ... 232
ezpolar ... 225
ezsurf ... 233
ezsurfc ... 234

F

f_test_regression ... 423
factor ... 301
factorial ... 302
fail ... 601
false ... 51
fcdf ... 432
fclear ... 203
fclose ... 189
fcntl ... 538
fdisp ... 184
feather ... 220
feof ... 203
ferror ... 203
feval ... 122
fflush ... 178
fft ... 475
fft2 ... 477
fftconv ... 477
fftfilt ... 477
fftn ... 477
fftshift ... 481
fftw ... 475
fgetl ... 190
fgets ... 190
field ... 145
fieldnames ... 83
figure ... 239
file_in_loadpath ... 149
file_in_path ... 528
fileattrib ... 527
filemarker ... 529
fileparts ... 529
filesep ... 529
fill ... 246
filter ... 478
filter2 ... 478
find ... 274
find_dir_in_path ... 149
findall ... 257
findobj ... 256
findstr ... 64
finite ... 274
finv ... 432
fix ... 302
fixed_point_format ... 42
flag ... 491
flipdim ... 276
fliplr ... 275
flipud ... 275
floor ... 302
fmod ... 302
fnmatch ... 528
foo ... 10
fopen ... 188
fork ... 535
format ... 175
formula ... 157
fortran_vec ... 561
fpdf ... 432
fplot ... 223
fprintf ... 191
fputs ... 189
fractdiff ... 482
fread ... 199
freport ... 203
freqz ... 479
freqz_plot ... 479
frewind ... 204
frnd ... 438
fscanf ... 196
fseek ... 203
fsolve ... 331
fstat ... 527
ftell ... 203
full ... 348
fullfile ... 529
func2str ... 155
function_name ... 18
functions ... 155
fwrite ... 201
fzero ... 333

G

gamcdf ... 432
gaminv ... 432
gamma ... 309
gammainc ... 309
gammaln ... 311

gampdf 432
gamrnd 438
gca 244
gcbf 260
gcbo 260
gcd 302
gcf 244
genpath 148
genvarname 97
geocdf 432
geoinv 432
geopdf 433
geornd 439
get 245
getegid 539
getenv 540
geteuid 539
getfield 83
getgid 539
getgrent 542
getgrgid 542
getgrnam 542
getpgrp 539
getpid 539
getppid 539
getpwent 541
getpwnam 541
getpwuid 541
getrusage 544
getuid 539
ginput 242
givens 316
glob 528
glpk 401
gls 410
gmap40 493
gmtime 516
gplot 353
gradient 303
gray 491
gray2ind 489
grid 237
griddata 472
griddata3 473
griddatan 473
gtext 242
gunzip 530
gzip 530

H

hadamard 289
hamming 482
hankel 289
hanning 482
hess 321
hex2dec 71
hex2num 72
hggroup 260
hidden 227
hilb 290
hist 209
histc 418
history 23
history_file 24
history_size 24
history_timestamp_format_string 24
hold 247
home 21
horzcat 277
hot 491
hotelling_test 423
hotelling_test_2 423
housh 327
hsv 491
hsv2rgb 494
hurst 482
hygecdf 433
hygeinv 433
hygepdf 433
hygernd 439
hypot 303

I

I 312
idivide 47
ifft 476
ifftn 477
ifftshift 482
ignore_function_time_stamp 147
imag 295
image 488
IMAGE_PATH 485
image_viewer 488
imagesc 488
imfinfo 486
imread 485
imshow 487
imwrite 485
ind2gray 489
ind2rgb 490
ind2sub 109
index 65
Inf 312
inferiorto 509
info 18
info_file 18
info_program 19
inline 156
inpolygon 470
input 179
inputname 139
int16 45
int2str 61
int32 45

int64 45
int8 45
interp1 455
interp1q 457
interp2 460
interp3 460
interpft 457
interpn 461
intersect 442
intmax 45
intmin 46
intwarning 46
inv 317
inverse 317
invhilb 290
ipermute 277
iqr 420
is_absolute_filename 530
is_duplicate_entry 273
is_leap_year 520
is_rooted_relative_filename 530
isa 33
isalnum 75
isalpha 75
isascii 75
iscell 86
iscellstr 91
ischar 56
iscntrl 75
iscommand 157
iscomplex 52
isdebugmode 173
isdefinite 53
isdigit 75
isdir 528
isempty 36
isequal 113
isequalwithequalnans 113
isfield 83
isfigure 243
isfloat 52
isglobal 99
isgraph 75
ishandle 243
ishghandle 243
ishold 247
isieee 543
isinf 274
isinteger 45
isletter 75
islogical 53
islower 75
ismac 543
ismatrix 52
ismember 441
ismethod 499
isna 35
isnan 274
isnull 36
isnumeric 52
isobject 499
ispc 543
isprime 53
isprint 75
ispunct 75
israwcommand 158
isreal 52
isscalar 52
issorted 280
isspace 76
issparse 349
issquare 53
isstrprop 76
isstruct 83
issymmetric 53
isunix 543
isupper 76
isvarname 97
isvector 52
isxdigit 76

J

jet 491

K

kbhit 179
kendall 420
keyboard 173
kill 539
kolmogorov_smirnov_cdf 433
kolmogorov_smirnov_test 424
kolmogorov_smirnov_test_2 424
kron 329
kruskal_wallis_test 424
krylov 328
kurtosis 416

L

laplace_cdf 433
laplace_inv 433
laplace_pdf 433
laplace_rnd 439
lasterr 165
lasterror 164
lastwarn 167
lcm 303
legend 236
legendre 310
length 36
lgamma 311
license 543, 544
lin2mu 495
line 245

link 524
linkprop 263
linspace 289
list_primes 303
load 182
loadaudio 495
loadobj 502
localtime 516
log 293
log10 293
log1p 293
log2 293
logical 50
logistic_cdf 433
logistic_inv 434
logistic_pdf 434
logistic_regression 429
logistic_rnd 439
logit 419
loglog 208
loglogerr 217
logm 328
logncdf 434
logninv 434
lognpdf 434
lognrnd 439
logspace 289
lookfor 18
lookup 459
lower 74
ls 540
ls_command 540
lsode 389
lsode_options 390
lsqnonneg 411
lstat 526
lu 322
luinc 375

M

magic 290
mahalanobis 417
make_absolute_filename 530
makeinfo_program 19
manova 425
mark_as_command 157
mark_as_rawcommand 158
mat2cell 87
mat2str 60
matrix_type 317
max 304
max_recursion_depth 111
mcnemar_test 425
md5sum 545
mean 413
meansq 415
median 413
menu 179
mesh 227
meshc 227
meshgrid 229
meshz 227
methods 499
mex 581
mexext 581
mfilename 147
min 304
mislocked 153
mkdir 525
mkfifo 525
mkoctfile 557
mkpp 451
mkstemp 202
mktime 517
mlock 152
mod 306
mode 416
moment 417
more 178
movefile 529
mpoles 446
mu2lin 495
munlock 153

N

NA 34
name 17, 145
namelengthmax 98
NaN 313
nargchk 141
nargin 139
nargout 140
nargoutchk 141
native_float_format 184
nbincdf 434
nbininv 434
nbinpdf 434
nbinrnd 439
nchoosek 418, 614
ndgrid 229
ndims 35
nelem 561
newplot 247
news 18
newtroot 121
nextpow2 293
nnz 349
nonzeros 349
norm 318
normcdf 434
normest 365
norminv 435
normpdf 435
normrnd 439

now 515
nthroot 294
ntsc2rgb 494
null 319
num2cell 87
num2hex 72
num2str 61
numel 36
nzmax 349

O

ocean 491
octave_config_info 544
octave_core_file_limit 186
octave_core_file_name 186
octave_core_file_options 186
OCTAVE_HOME 543
OCTAVE_VERSION 543
ols 410
onenormest 365
ones 284
operator () 561
optimget 411
optimset 411
options 105
orderfields 83
orient 242
orth 319
output_max_field_width 41
output_precision 41

P

P_tmpdir 530
pack 531
page_output_immediately 178
page_screen_output 178
PAGER 178
PAGER_FLAGS 178
pareto 213
parseparams 142
pascal 290
patch 246
path 148
pathdef 148
pathsep 149
pause 520
pcg 371
pchip 482
pclose 534
pcolor 220
pcr 373
peaks 243
periodogram 482
perl 534
perms 418
permute 277
pi 312
pie 218
pink 492
pinv 319
pipe 536
pkg 547
planerot 317
playaudio 496
plot 205
plot3 230
plotmatrix 212
plotyy 207
poisscdf 435
poissinv 435
poisspdf 435
poissrnd 440
pol2cart 311
polar 218
poly 452
polyarea 470
polyder 450
polyderiv 449
polyfit 450
polygcd 448
polyint 450
polyinteg 450
polyout 452
polyreduce 452
polyval 445
polyvalm 445
popen 534
popen2 534
pow2 294
ppplot 421
ppval 451
prctile 414
prepad 281
primes 306
print 239
print_empty_dimensions 43
print_usage 163
printf 190
prism 492
probit 419
prod 299
program_name 15
prop_test_2 425
PS1 25
PS2 26
PS4 26
putenv 540
puts 190
pwd 541

Q

qp 407
qqplot 420

qr . . . 323
qrdelete . . . 324
qrinsert . . . 324
qrshift . . . 325
qrupdate . . . 324
quad . . . 381
quad_options . . . 382
quadgk . . . 383
quadl . . . 383, 384
quadv . . . 384
quantile . . . 414
quit . . . 17
quiver . . . 218, 219
quiver3 . . . 219
qz . . . 325
qzhess . . . 326

R

rainbow . . . 492
rand . . . 285
rande . . . 286
randg . . . 287
randn . . . 286
randp . . . 286
randperm . . . 288
range . . . 419
rank . . . 319
ranks . . . 419
rat . . . 187
rats . . . 187
rcond . . . 319
re_read_readline_init_file . . . 25
read_readline_init_file . . . 25
readdir . . . 525
readlink . . . 525
real . . . 295
reallog . . . 294
realmax . . . 314
realmin . . . 314
realpow . . . 294
realsqrt . . . 294
record . . . 496
rectangle_lw . . . 483
rectangle_sw . . . 483
rectint . . . 470
refresh . . . 247
refreshdata . . . 264
regexp . . . 67
regexpi . . . 69
regexprep . . . 69
regexptranslate . . . 70
rehash . . . 149
rem . . . 306
rename . . . 524
repmat . . . 285
reshape . . . 278
residue . . . 448, 449
resize . . . 278, 561
restoredefaultpath . . . 149
rethrow . . . 165
return . . . 144
rgb2hsv . . . 493
rgb2ind . . . 489
rgb2ntsc . . . 494
ribbon . . . 231
rindex . . . 65
rmdir . . . 525
rmfield . . . 83
rmpath . . . 148
roots . . . 446
rose . . . 213
rosser . . . 290
rot90 . . . 276
rotdim . . . 276
round . . . 306
roundb . . . 306
rows . . . 36
rref . . . 320
run . . . 122
run_count . . . 419
run_history . . . 23
run_test . . . 426
rundemos . . . 602

S

S . . . 102, 182
save . . . 180
save_header_format_string . . . 183
save_precision . . . 183
saveaudio . . . 495
saveobj . . . 502
savepath . . . 148
saving_history . . . 24
scanf . . . 196
scatter . . . 211
scatter3 . . . 212
schur . . . 326
sec . . . 296
secd . . . 298
sech . . . 297
SEEK_CUR . . . 204
SEEK_END . . . 204
SEEK_SET . . . 204
semilogx . . . 208
semilogxerr . . . 217
semilogy . . . 208
semilogyerr . . . 217
set . . . 245
setaudio . . . 496
setdiff . . . 442
setenv . . . 540
setfield . . . 83
setgrent . . . 542
setpwent . . . 541

setxor 443
shading 231
shg 248
shift 279
shiftdim 279
SIG 539
sighup_dumps_octave_core 186
sign 306
sign_test 426
sigterm_dumps_octave_core 186
silent_functions 139
sin 295
sinc 479
sind 298
sinetone 483
sinewave 483
single 44
sinh 296
size 36
size_equal 37
sizeof 37
skewness 417
sleep 520
slice 231
sombrero 242
sort 279
sortrows 280
source 155
spalloc 348
sparse 348
sparse_auto_mutate 355
spaugment 367
spconvert 349
spdiags 345
spearman 419
spectral_adf 483
spectral_xdf 483
specular 229
speed 602
spencer 483
speye 345
spfun 346
sph2cart 311
sphere 235
spinmap 493
spline 458
split_long_rows 41
spmax 346
spmin 346
spones 346
spparms 366
sprand 346
sprandn 346
sprandsym 347
sprank 367
spring 492
sprintf 191
spstats 350
spy 352
sqp 408
sqrt 294
sqrtm 328
squeeze 37
sscanf 196
stairs 210
stat 526
statistics 417
std 415
stderr 188
stdin 187
stdout 187
stem 210
stem3 211
stft 483
str 18
str2double 72
str2func 155
str2num 73
strcat 59
strchr 65
strcmp 62
strcmpi 63
strfind 65
strftime 517
string_fill_char 56
strjust 73
strmatch 66
strncmp 62
strncmpi 63
strptime 519
strrep 67
strsplit 67
strtok 66
strtrim 64
strtrunc 64
struct 82
struct_levels_to_print 78
struct2cell 85
structfun 84
strvcat 59
studentize 417
sub2ind 109
subplot 239
subsasgn 504
subsindex 505
subspace 326
subsref 503
substr 67
substruct 84
sum 299
summer 492
sumsq 300
superiorto 509
suppress_verbose_help_message 19
surf 228
surface 246

surfc ... 228
surfl ... 228
surfnorm ... 228
svd ... 326
svds ... 370
swapbytes ... 34
syl ... 329
sylvester_matrix ... 291
symamd ... 362
symbfact ... 367
symlink ... 524
symrcm ... 363
symvar ... 157
synthesis ... 484
system ... 533

T

t_test ... 426
t_test_2 ... 426
t_test_regression ... 427
table ... 419
tan ... 296
tand ... 298
tanh ... 297
tar ... 530
tcdf ... 435
tempdir ... 530
tempname ... 530
test ... 597
text ... 236
tic ... 520
tilde_expand ... 528
time ... 515
tinv ... 435
title ... 236
tmpfile ... 202
tmpnam ... 202
toascii ... 74
toc ... 520
toeplitz ... 291
tolower ... 74
toupper ... 74
tpdf ... 435
trace ... 320
trapz ... 385
treelayout ... 353
treeplot ... 353
triangle_lw ... 484
triangle_sw ... 484
tril ... 281
trimesh ... 465
triplequad ... 386
triplot ... 465
triu ... 281
trnd ... 440
true ... 51
tsearch ... 467
tsearchn ... 467
typecast ... 33
typeinfo ... 33

U

u_test ... 427
uint16 ... 45
uint32 ... 45
uint64 ... 45
uint8 ... 45
umask ... 526
uname ... 543
undo_string_escapes ... 74
unidcdf ... 435
unidinv ... 435
unidpdf ... 435
unidrnd ... 440
unifcdf ... 435
unifinv ... 436
unifpdf ... 436
unifrnd ... 440
union ... 442
unique ... 441
unix ... 533
unlink ... 525
unmark_command ... 157
unmark_rawcommand ... 158
unmkpp ... 451
unpack ... 531
untar ... 531
unwrap ... 479
unzip ... 531
upper ... 74
urlread ... 532
urlwrite ... 532
usage ... 163
usleep ... 521

V

validatestring ... 63
values ... 418
vander ... 291
var ... 415
var_test ... 427
vec ... 281
vech ... 281
vectorize ... 157
ver ... 544
version ... 544
vertcat ... 277
view ... 230
voronoi ... 468
voronoin ... 469

W

waitforbuttonpress 242
waitpid 536
warning 166
warranty 18
wavread 496
wavwrite 496
wblcdf 436
wblinv 436
wblpdf 436
wblrnd 440
WCOREDUMP 537
weekday 523
welch_test 427
WEXITSTATUS 537
what 106
which 105
white 492
who 102
whos 102
whos_line_format 103
wienrnd 440
WIFCONTINUED 537
WIFEXITED 537
WIFSIGNALED 537
WIFSTOPPED 537
wilcoxon_test 428
wilkinson 291
winter 492
WNOHANG 537
WSTOPSIG 538
WTERMSIG 538
WUNTRACED 538

X

xlabel 237
xlim 223
xor 273

Y

yes_or_no 179
ylabel 237
yulewalker 484

Z

z_test 428
z_test_2 428
zeros 285
zip 531
zlabel 237

Operator Index

!

! 114
!= 113

"

" 35, 55

&

& 114
&& 114

'

' 35, 55, 112

(

(.... 107

)

) 107

*

* 111
** 112
*= 117

+

+ 111, 112
++ 118
+= 117

,

, ... 40

-

- 111, 112
-- 118
-= 117

.

.' 112
.* 111
.** 112
.+ 111
./ 112
.^ 112
.\ 112

/

/ 112
/= 117

;

; 40

<

< 113
<= 113

=

= 115
== 113

>

> 113
>= 113

[

[.... 40

]

] 40

^

^ 112

\

\ 112

|

| 114
|| 115

~

~ 114
~= 113

C

colon 43
